# Eliminating Transition State Calculations for Faster and More Accurate Reactivity Prediction in Sulfa-Michael Additions Relevant to Human Health and the Environment

**DOI:** 10.1021/acsomega.2c03739

**Published:** 2022-07-21

**Authors:** Piers A. Townsend, Elliot H. E. Farrar, Matthew N. Grayson

**Affiliations:** † Centre for Sustainable Chemical Technologies, Department of Chemistry, University of Bath, Claverton Down, Bath BA2 7AY, U.K.; ‡ Department of Chemistry, University of Bath, Claverton Down, Bath BA2 7AY, U.K.

## Abstract

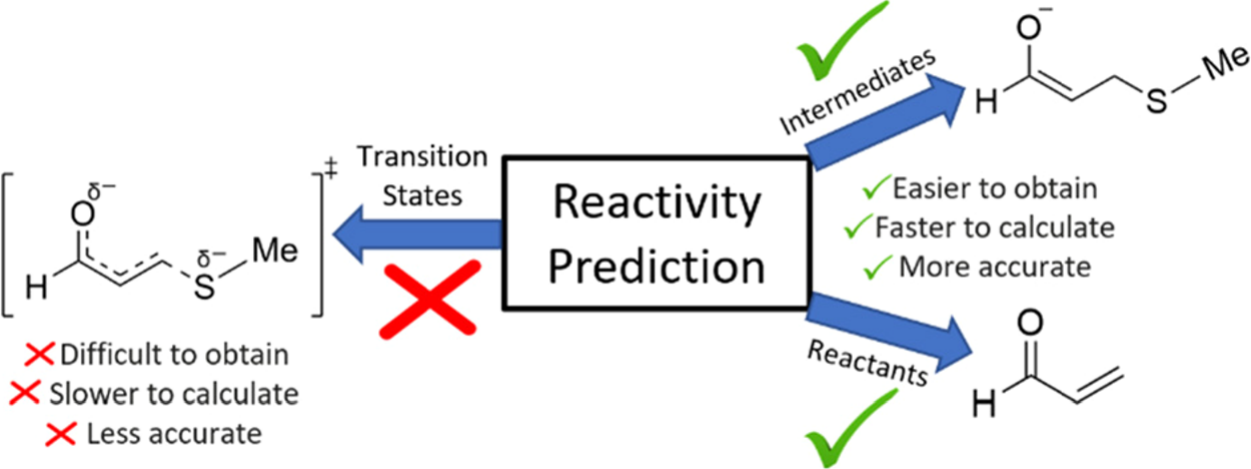

Fast and accurate computational approaches to predicting reactivity in sulfa-Michael additions are required for high-throughput screening in toxicology (e.g., predicting excess aquatic toxicity and skin sensitization), chemical synthesis, covalent drug design (e.g., targeting cysteine), and data set generation for machine learning. The kinetic glutathione chemoassay is a time-consuming in chemico method used to extract kinetic data in the form of log(*k*_GSH_) for organic electrophiles. In this work, we use density functional theory to compare the use of transition states (TSs) and enolate intermediate structures following C–S bond formation in the prediction of log(*k*_GSH_) for a diverse group of 1,4 Michael acceptors. Despite the widespread use of transition state calculations in the literature to predict sulfa-Michael reactivity, we observe that intermediate structures show much better performance for the prediction of log(*k*_GSH_), are faster to calculate, and easier to obtain than TSs. Furthermore, we show how linear combinations of atomic charges from the isolated Michael acceptors can further improve predictions, even when using inexpensive semiempirical quantum chemistry methods. Our models can be used widely in the chemical sciences (e.g., in the prediction of toxicity relevant to the environment and human health, synthesis planning, and the design of cysteine-targeting covalent inhibitors), and represent a low-cost, sustainable approach to reactivity assessment.

## Introduction

Michael addition reactions, characterized by nucleophilic attack at the β-carbon of an α,β-unsaturated carbonyl compound, have been widely used in synthesis for generating a variety of carbon-nucleophile bond types (e.g., C–S, C–N, and C–C bonds).^1−3^ The sulfa-Michael addition in particular is a highly important reaction given its extensive use in organic synthesis^4−6^ pharmacology,^7,8^ toxicology,^9,10^ and materials science.^11^ Therefore, the ability to assess and predict the reactivity of Michael acceptors (MAs) toward sulfur nucleophiles is of paramount importance across a range of disciplines. In chemical synthesis, predicting rates of reaction between MAs and a given nucleophile would provide low-cost, quantitative predictions for novel synthetic transformations. In toxicology, such predictions could be used in the chemical risk assessment of aquatic toxicity and skin sensitization, while aligning with the increasingly important “Green” toxicology approach.^12−14^ Lastly, in drug discovery, such predictions could be used in the design of cysteine-targeting, targeted covalent inhibitors (TCIs). TCIs are an emerging class of compounds in drug discovery and recently, three FDA-approved drugs (Afatanib, Ibrutinib, and Osimertinib) were designed to react irreversibly with a sulfur-containing cysteine residue through a hetero-Michael addition.^15,16^

**Figure 1:**
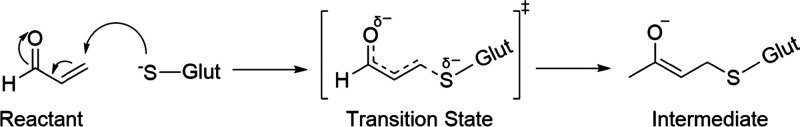
Generalized reaction mechanism between glutathione and a 1,4 Michael acceptor. “Glut” represents a glutathione residue minus its sulfur atom.

**Figure 2:**
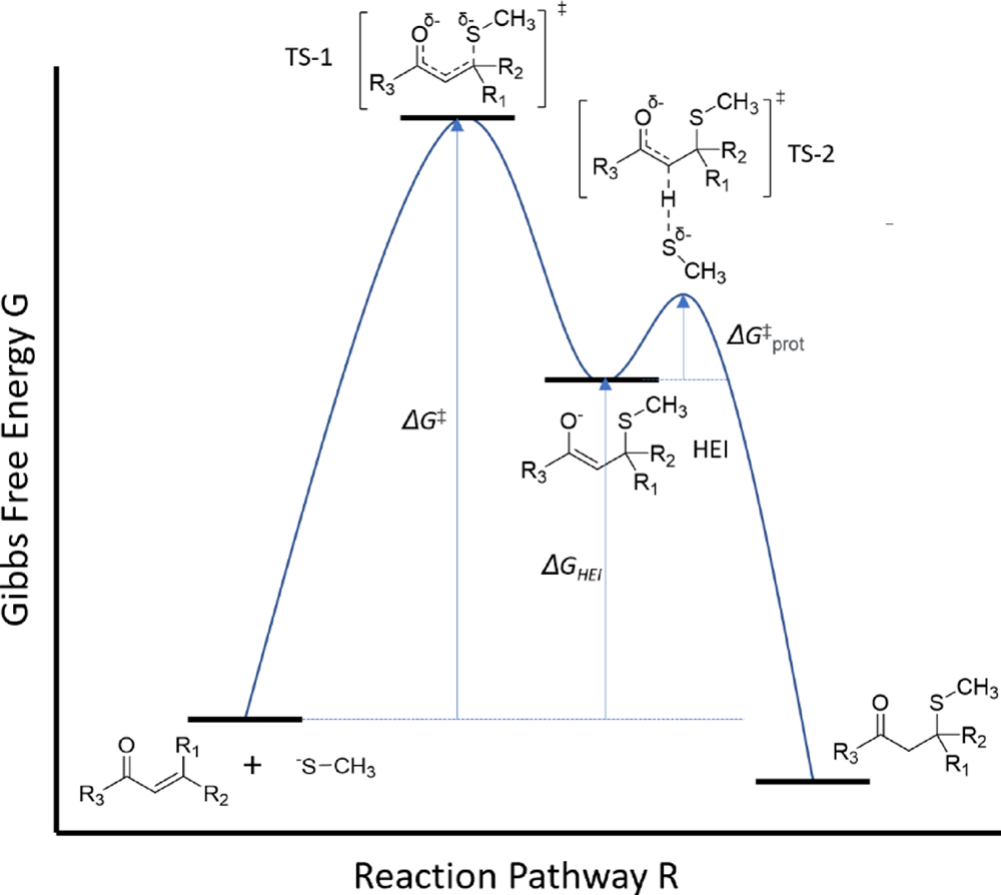
GSH-MA reaction pathway showing the structure of the reactants, intermediate, TS, and product.

In 2006, Schultz et al. presented a pioneering framework for modeling reactions between an electrophilic toxicant and a biological macromolecule, many of which are nucleophilic.^17^ This framework proposed the use of model nucleophiles to better understand reactive toxicity and the ultimate biological effects associated with its existence. Traditionally, MA reactivity has been assessed using chemoassays, with the kinetic glutathione chemoassay developed by Böhme et al. being the most widely used to examine sulfa-Michael additions.^18^ This method has been used extensively to obtain second-order rate constants [L mol^–1^ min^–1^] to assess the reactivity of different α,β-unsaturated carbonyl compounds and uses glutathione (GSH) as the nucleophile (Figure 1).^19^ In 2016, Schürmann and co-workers compiled, to the best of our knowledge, the largest known experimental kinetic glutathione assay rate data set for α,β-unsaturated carbonyl compounds.^20^ As we move toward a more sustainable future, it is vital that in silico approaches are developed that align with the principles of green chemistry. Predictive in silico approaches provide advantages in the design of safer chemicals and less hazardous syntheses, along with reducing harmful waste products that are commonly utilized in experimental methods. Thus, to reduce the environmental, financial, and time-based costs attached to chemoassays, there have been numerous attempts to develop quantum chemical transition state (TS) approaches to the prediction of MA reactivity data sets. In 2010, Cronin and co-workers performed TS calculations for 22 MAs reacting with methanethiolate.^21^ They presented a QSAR regression equation for the prediction of log(*k*_GSH_) that used the activation barrier Δ*E*^‡^_PCM_ for both the forward and backward reaction, and the solvent accessible surface area (SASA), resulting in an impressive squared Pearson correlation coefficient (*r*^2^) of 0.90 between their calculated and experimental values for log(*k*_GSH_):
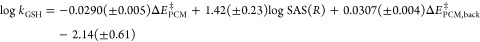


Although this work did provide strong regression statistics, multiple descriptors were required, and when only Δ*E*^‡^_PCM_ and the SASA were utilized as features, a relatively poor correlation (*r*^2^ = 0.51) was observed. In the same year, Schürmann and co-workers published a set of multidescriptor models based on ground state features such as a charge-limited local electrophilicity index, a σ-bond energy (obtained from natural bond order analysis), and SASAs for both the α- and β-carbons.^22^ Overall, their models showed strong statistics, with a four-descriptor model showing the best performance (*r*^2^ = 0.93). In 2011, Mulliner et al. used TS calculations to predict log(*k*_GSH_) for 35 α,β-unsaturated carbonyl compounds.^23^ They also performed calculations under the PCM solvent model to examine the effect of implicit solvation on the prediction of log(*k*_GSH_). For solvated and nonsolvated models, respectively, they obtained regression equations with *r*^2^ = 0.76 and *r*^2^ = 0.68 between calculated and experimental log(*k*_GSH_) values. Following this in 2013, the same authors calculated reaction barriers (Δ*E*^‡^) for a set of esters, and from Δ*E*^‡^, calculated log(*k*_GSH_) using their previously developed regression model from 2011. This study examined the use of log(*k*_GSH_) and hydrophobicity (in the form of log(*K*_ow_), where *K*_ow_ is the octanol–water partition coefficient) for the prediction of 50% growth inhibition of*T. pyriformis* (log(EC_50_)). It is thus clear that TSs have commonly been used in assessing MA reactivity, but far fewer studies have examined high energy intermediate structures (HEI, see Figure 2) and their ability to predict reactivity.^24^ In 2013, Enoch and Roberts performed DFT calculations on a data set of 26 MAs and calculated their Δ*E*_HEI_ values upon reaction with methanethiolate.^25^ With the full data set, their results showed a poor correlation (*r*^2^ = 0.02) between skin sensitization potency (pEC3) and intermediate energies upon linear regression analysis. Upon refining the data set and removing some problematic compounds from the analysis, their results showed improved correlation (*r*^2^ = 0.43) for a single descriptor model using intermediate energies. This was further improved by adding a SASA descriptor, resulting in a multivariate regression model with strong statistics (*r*^2^ = 0.79). Further in 2016, Ebbrell et al. developed an in silico profiler for the prediction of RC_50_ values using intermediate MA structures, where RC_50_ is the concentration of electrophiles needed to reduce the concentration of GSH by 50%. Their results showed that a single descriptor model using Δ*E*_HEI_ resulted in modest regression statistics (*n* = 54, *r*^2^ = 0.52). However, this model was greatly improved (*n* = 41, *r*^2^ = 0.87) by adopting a multivariate approach and including an additional SASA descriptor.^10^ In 2017, Ebbrell et al. presented a thorough analysis on how their previously developed fragment profiler could be used in the prediction of aquatic toxicity (toward*T. pyriformis*) and skin sensitization potential.^26^ This study highlighted the crucial role of Δ*E*_HEI_ regression models for the prediction of important toxicological endpoints such as EC_50_/pEC_50_. Thus, it is clear that intermediates and Δ*E*_HEI_ are of great use and importance in predictive toxicology. Furthermore, intermediate structures are particularly desirable from a practical standpoint; TS calculations are not only computationally costly but are challenging for both experts and nonexperts to perform.^27^ Compared with TSs, intermediate structure calculations involve optimization to a minimum, which is typically less costly, easier to automate (e.g., for data set generation in machine learning (ML)), and much easier to perform.^24^ In this work, we find that easily calculable intermediate energies perform better than TSs in the prediction of log(*k*_GSH_). Furthermore, we find that linear regression models built with very inexpensive features from semiempirical quantum mechanical (SQM) calculations can make reliable predictions of log(*k*_GSH_) comparable to methods using DFT calculations, even when only reactant-derived features are used.

## Materials and Methods

**Figure 3:**
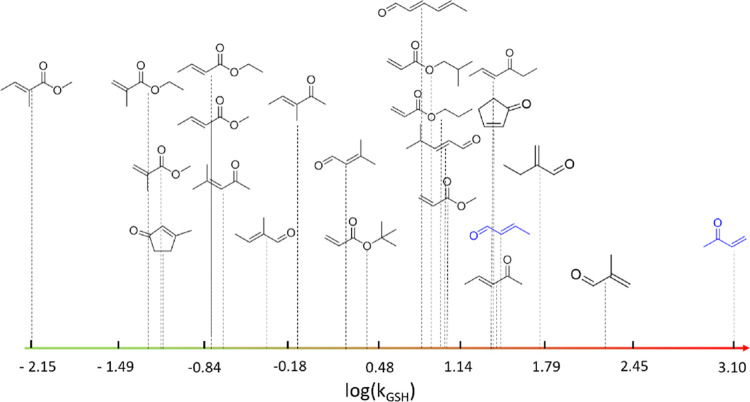
Visualizing log(*k*_GSH_) for the 23 compounds included in this study. Red indicates a higher reactivity with glutathione, while green indicates lower reactivity. Blue compounds indicate that minor truncation was performed (see the SI for full details).

Kinetic glutathione assay data (log(*k*_GSH_)) for 23 1,4 MAs were taken from the work published by Böhme et al., providing experimental rate data for nine esters, seven aldehydes, and seven ketones (see Figure 3 and Table S1 in the Supporting Information).^20^ DFT calculations were performed with Gaussian 16 (Rev. A.03)^28^ at the M06-2X/def2-TZVPP level of theory under the IEFPCM implicit solvation model (water), which has been used extensively for modeling organic reactions.^29−32^ Implicit solvation in water was chosen to simulate the experimental conditions used in the kinetic glutathione chemoassay. In line with previous studies, and to ensure computational feasibility, methanethiolate was used as a model nucleophile in all calculations.^10,25^ All regression models were developed via the Scikit-learn Python package.^33^ Activation barriers and intermediate energy differences were calculated according to:
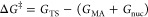

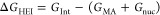
where Δ*G*^‡^ is the activation free energy, *G*_TS_ is the TS free energy, *G*_MA_ is the free energy of a MA, Δ*G*_HEI_ is the free energy difference between the intermediate and the reactant state, *G*_Int_ is the free energy of the intermediate structure, and *G*_nuc_ is the free energy of methanethiolate. For full computational details, see the Supporting Information.

## Results and Discussion

In total, across the data set, 94 reactant ground states, 226 TSs, and 229 intermediate structures were obtained and verified as either minima or first-order saddle points on the M06-2X/def2-TZVPP-IEFPCM (water) potential energy surface. Two compounds were minorly truncated in this work; 1-pentene-3-one to methyl vinyl ketone and trans-2-pentenal to but-2-enal. Previous literature demonstrates that conformational freedom is often neglected in the construction of QSAR models that use descriptors derived from quantum chemical methods.^34−36^ However, a more thorough exploration of chemical space is needed. To illustrate this, six reactant conformers were obtained for 2-ethylacrolein, and the lowest energy structure was 3.26 kcal/mol lower in energy than the highest energy conformation. Thus, if single conformations are examined, large errors can be introduced in the calculation of thermochemical data. A further example includes the two reactant conformers obtained for methyl methacrylate; a difference of 7.73 kcal/mol was obtained between the two conformers. Thus, it is quite clear that without direct consideration of molecular flexibility, calculated activation barriers and relative intermediate energies can vary considerably.

### TS Calculations

**Figure 4:**
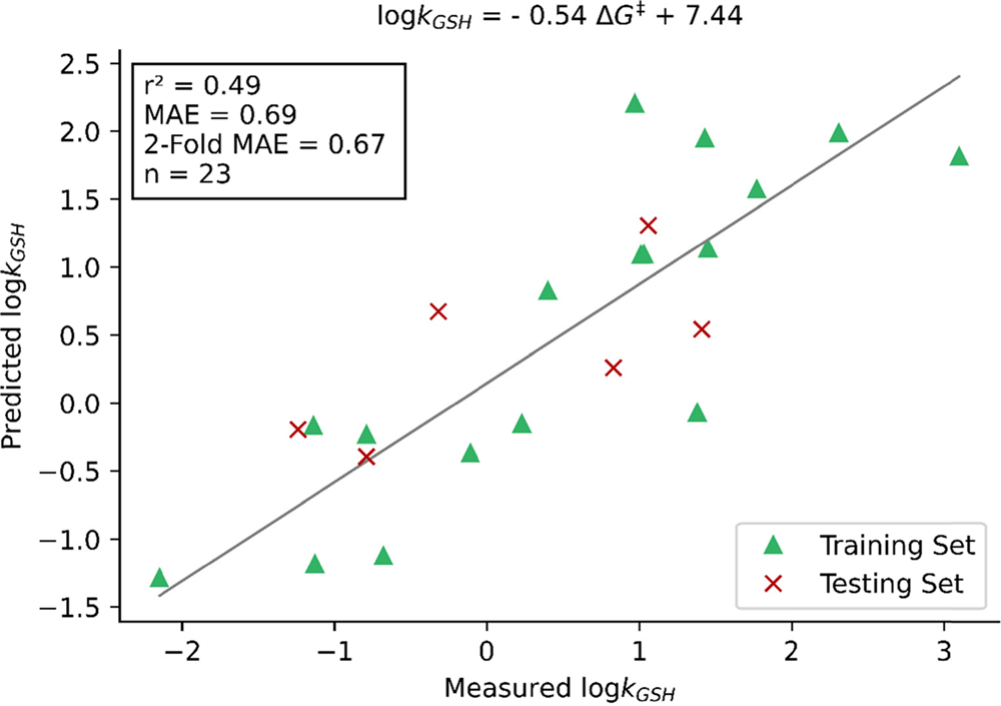
Linear regression of log(*k*_GSH_) on the activation energy derived from transition state structures (M06-2X/def2-TZVPP-IEFPCM (water)) was performed. Predicted log(*k*_GSH_) is plotted against measured log(*k*_GSH_).

Across the data set, experimental rate constants ranged from 3.1 to −2.15 log units (see Figure 3). The more positive a value of log(*k*_GSH_), the faster the reaction between an MA and methanethiolate, and thus, the corresponding MA is more reactive. Our calculated activation energies ranged from 9.7 to 16.1 kcal/mol across all compounds, with esters showing the largest range among the three groups. A single descriptor linear regression analysis showed a poor correlation between log(*k*_GSH_) and Δ*G*^‡^ (Figure 4), with a squared Pearson correlation coefficient (*r*^2^) of 0.49 between the predicted and measured log(*k*_GSH_). The resultant model demonstrates that TSs and free energies of activation provide a poor prediction of log(*k*_GSH_) in the MA data set, with a relatively large test set mean absolute error (MAE) of 0.69 log units. These results show good agreement with previous work by Schwöbel et al., where it was shown that at the B3LYP/6-31G** level of theory, no global model for esters, ketones, and aldehydes exists that utilizes activation energies derived from TSs.^21^ Additionally, it has been previously reported that DFT can prove problematic in the study of charged ionic TSs due to large errors in the calculated reactivity parameters.^37^ It is also known that most implicit solvent models define the solvent cavity as a set of overlapping spheres with fixed atomic radii. These radii are experimentally calculated, and for IEFPCM, the Bondi data set is used; for each atom, the atomic radius is calculated through multiplication of a constant (1.2) by the Bondi atomic van der Waals radius.^38,39^ These empirically determined radii use solvation free energy training sets for parameterization, and TSs are often not included in the initial training set, thereby introducing error when TSs are examined with implicit solvent models.^40^ Additionally, radii of the specific atoms involved in the bond-forming/breaking process (e.g., S–C from our study) are often poorly defined in TSs for this reason. It must also be noted that from a practical perspective, explicit solvation is too costly and would eliminate the easy-to-use nature of our models.

Therefore, our work, combined with previous literature, indicates that a poor correlation between log(*k*_GSH_) and activation energy is likely to be independent of both basis set and DFT functional. However, to further corroborate this, benchmarking studies could be performed to thoroughly examine the role that level of theory and solvation method plays in this problem. Upon examination of our results and previously published work, it is clear that single-descriptor Δ*G*^‡^ models do not provide a sufficient description of MA reactivity toward sulfur nucleophiles and should not be used for making predictions about the reactivity of MAs with glutathione.

### Intermediate Structures

**Figure 5:**
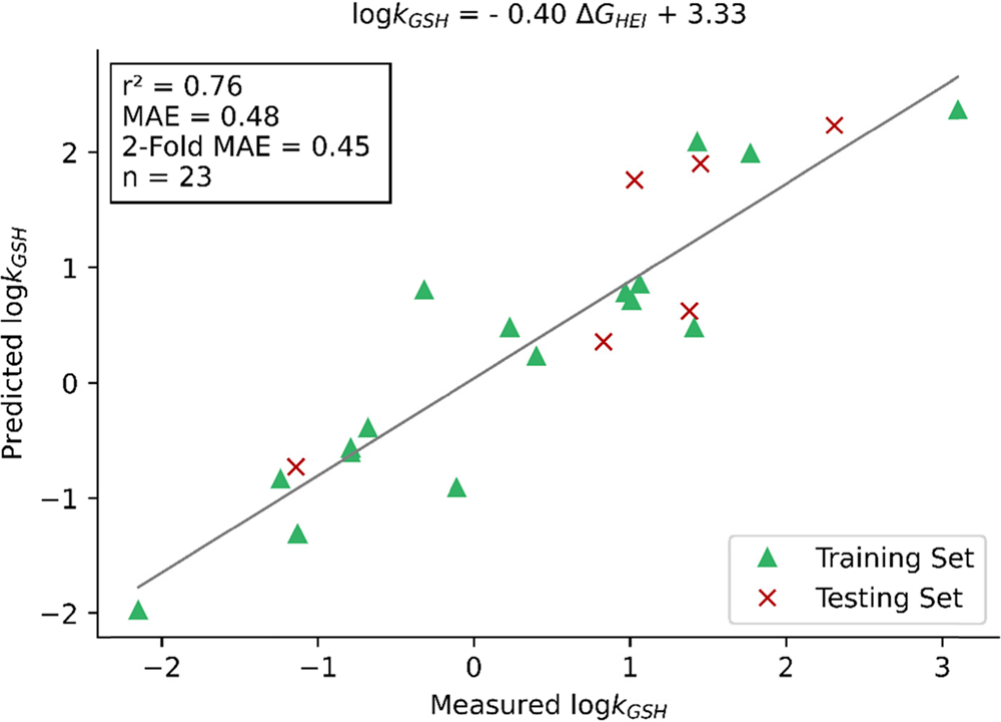
Linear regression of log(*k*_GSH_) on the intermediate energy differences (M06-2X/def2-TZVPP-IEFPCM (water)). Predicted log(*k*_GSH_) is plotted against measured log(*k*_GSH_).

The computed stability of the enolate intermediates after C–S bond formation, which closely resembles the TSs in line with Hammond’s postulate, may be used to accurately predict the reactivity of MAs toward thiol containing compounds.^41^ Across the data set, calculated intermediate energies Δ*G*_HEI_ varied from 2.4 to 13.3 kcal/mol. Intermediate energies for ketones ranged from 2.4 to 11.6 kcal/mol, aldehydes ranged from 2.8 to 7.5 kcal/mol, and esters ranged from 6.2 to 13.3 kcal/mol. A linear regression analysis with Δ*G*_HEI_ resulted in a significantly improved model compared to TS barriers (Figure 5), with *r*^2^_=_ 0.76 and a test set MAE of 0.48 log units. This is a noteworthy result; generally, it would be expected that TS barriers correlate more strongly with kinetic data such as log(*k*_GSH_), if the rate-determining step has been modeled. As highlighted by Schürmann and co-workers, the rate-determining step is the addition of methanethiolate to the MA, and the protonation step (see Figure 2, TS-2) is expected to be fast and not rate-limiting.^22^ However, to ensure we have focused our calculations on the rate-determining step (TS-1), we calculated reaction barriers of the protonation step (Δ*G*^‡^_prot_) for six structures in our data set; two esters, two ketones, and two aldehydes, with a fast and slow reacting compound being chosen for each (see Table S2). Methanethiol was the proton source as per the work by Northrop and co-workers.^42^ All calculated barriers for the protonation step were lower than the corresponding barrier for the addition of methanethiolate by an average value of 6.41 kcal/mol, thus confirming it highly likely that thiolate addition is the rate-determining step. From these results, it remains clear that, in line with the Hammond postulate, calculation of Δ*G*_HEI_ can provide a fast, high-throughput measure of 1,4 MA reactivity toward methanethiolate (and thus, thiol containing compounds), with our models permitting strong quantitative predictions to be made for initial, preliminary reactivity assessments. As detailed above, it is likely that the deficiencies of DFT TS modeling account for why our intermediate models perform better than those using TS-derived features.

A key advantage in the development of this model is its simplicity. A single variable regression model that uses calculated intermediate energies is very simple to use for both experts and nonexperts. TS searching is a nontrivial task, involving specialist knowledge and a high degree of human input to arrive at suitable structures. Optimization of only reactant and intermediate structures is significantly easier for the nonspecialist to make predictions. Minima, such as the intermediate structures presented here, converge more readily, thereby providing a great practical advantage for making predictions.^43^ Successfully obtaining a set of TSs for a single structure can take many rounds of re-optimization, while intermediate geometries can be readily obtained after a single calculation. A generalized workflow for obtaining intermediate structures was presented in one of our previous studies, and can be equally applied in the context of this study.^24^ Our model presents a fast, easy-to-use method for predicting the reactivity of 1,4 MAs with thiol containing compounds.

### Multivariate Models

**Figure 6:**
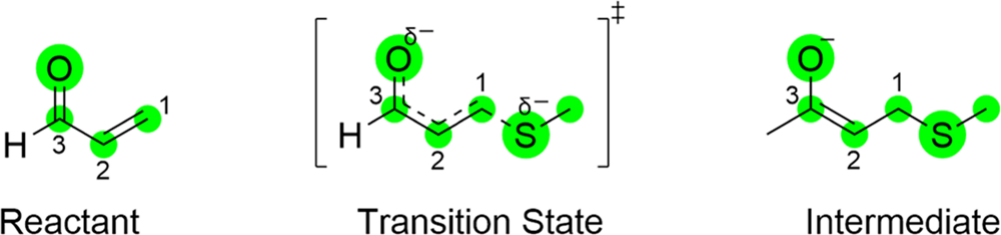
Mulliken and APT atomic charges were calculated for highlighted atoms only.

**Figure 7:**
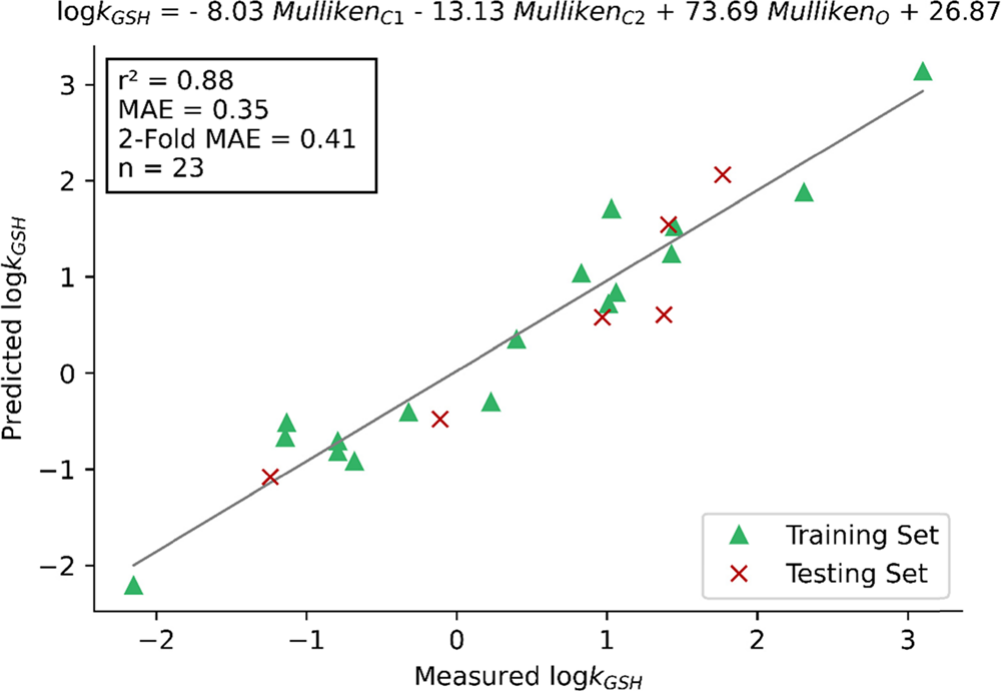
Linear regression of log(*k*_GSH_) on key atomic charges of the MA (M06-2X/def2-TZVPP-IEFPCM (water)). Predicted log(*k*_GSH_) is plotted against measured log(*k*_GSH_).

To see if the intermediate or TS models could be improved upon, a number of multivariate linear regression models were also generated using key Mulliken atomic charges, APT atomic charges (Figure 6), and several other chemical features (see Supporting Information) for all MAs, intermediates, and TSs. With the aim to create an easy-to-use method, Mulliken and APT charges, which are readily extracted from Gaussian output files and are thus very user friendly for the nonexpert performing calculations, were selected. The best results were obtained using MA features only, with *r*^2^ = 0.88 and an MAE of 0.35 log units via a three-descriptor model combining the Mulliken atomic charges of the α- and β-carbons and the carbonyl oxygen (Figure 7). Not only are these results a significant improvement on the Δ*G*_HEI_ model, but with fewer atoms and less conformational flexibility than the corresponding intermediates, calculations on reactant MA structures are even more trivial.

### Semiempirical Models

**Figure 8:**
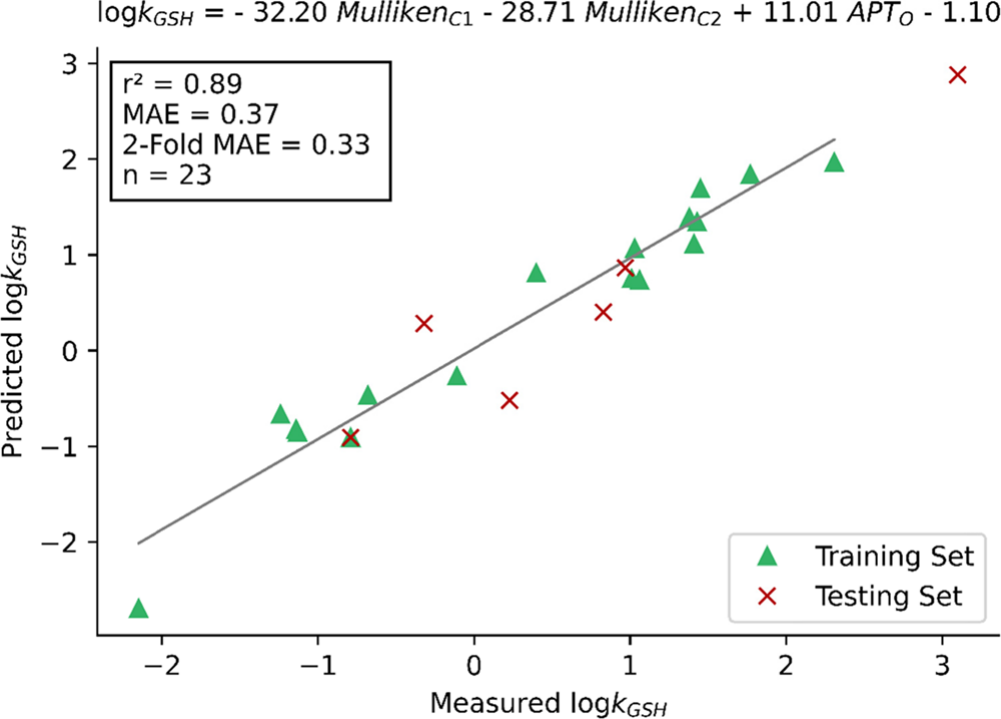
Linear regression of log(*k*_GSH_) on key atomic charges of the MA (AM1). Predicted log(*k*_GSH_) is plotted against measured log(*k*_GSH_).

Finally, we reoptimized all MAs and intermediates using the SQM AM1 method^44^ and generated multivariate linear models using the same chemical features as above. Because SQM calculations are typically orders of magnitude faster than DFT, regression models derived from SQM calculations could substantially reduce the computational expense of predictions. A result of particular significance is the negative relationship observed between log(*k*_GSH_) and the Mulliken charges (of C1 and C2) in the regression equation (see Figure 8). Although counterintuitive according to chemical intuition, previous literature indicates that MAs have a propensity to show interesting charge behavior on the α- and β-carbons. Spencer et al. used ^13^C NMR chemical shifts to examine the effect of electron-withdrawing groups (EWGs) at different positions on the aryl group of methyl cinnamates upon reaction with GSH.^45^ The presence of EWGs resulted in an increased rate of reaction with GSH, with the chemical shifts of the β-carbon being shifted slightly upfield (less positively charged). Additionally, although downfield shifts were observed for the α-carbon resonances with an increasing rate of reaction, no obvious statistical relationship was apparent between the rate of GSH addition and the α-carbon chemical shifts. When ortho-hydroxyl substitution on the aryl group was considered, a significant observation of an upfield shift of 3–6 ppm was observed at the β-carbon. These observations suggest that the electron distribution (and thus charge) at the α- and β-carbons can behave in an unusual way in the context of sulfa-Michael addition, and they agree with our results. Further to this point, the regression equation demonstrates a positive relationship between log(*k*_GSH_) and the APT charge on the carbonyl oxygen. It is possible that due to the highly electronegative nature of oxygen, an increasingly positive charge on the carbonyl oxygen is indicative of less overall electron density in the conjugated α,β-unsaturated carbonyl substructure. Thus, with lower electron density, the MA electrophile should become more receptive to nucleophilic attack, and the rate of reaction would increase as the charge becomes increasingly positive on oxygen. Similar to the model presented in the previous section, the best performing model utilized atomic charges on the α- and β-carbons and the carbonyl oxygen of the MA, with *r*^2^ = 0.89 and an MAE of 0.37 log units (Figure 8). This provides very similar performance to the DFT MA model, and is better than the single feature Δ*G*_HEI_ model but reduces the need for time-consuming DFT calculations. It is significant to note that a combination of models with differing levels of theory, opens up their use to a wider audience with differing computational resources.

## Conclusions

In this work, TS, intermediate, and reactant structures were explored in reactivity predictions (log(*k*_GSH_)) for a group of 23 sulfa-Michael additions. Such an approach to predicting sulfa-Michael reactivity is desirable for high-throughput screening in chemical synthesis, toxicology (aquatic toxicity and skin sensitization prediction), drug discovery (targeted covalent inhibitor design), and reactivity data set generation for ML. Further, our models provide a practical advantage in shifting the focus toward more sustainable approaches to chemical reactivity assessment. TSs were first considered, and activation free energies showed poor predictive performance in regression analyses toward log(*k*_GSH_) (*r*^2^ = 0.49, MAE = 0.69 log units). Intermediate enolate structures that follow the C–S bond-forming TSs were also considered and showed stronger predictive performance in regression analyses toward log(*k*_GSH_) (*r*^2^ = 0.76, MAE = 0.48 log units). Intermediate structures provide two key advantages over TSs: they are easier to compute and provide increased calculation speeds. Using linear combinations of purely reactant-derived chemical features, thus simplifying the calculations even further, resulted in noticeable improvements to our models with respective squared Pearson correlation coefficients and MAEs of 0.88 and 0.35 log units with DFT, and 0.89 and 0.37 log units with SQM. The models presented here are fast and easy-to-use methods for predicting log(*k*_GSH_).

## Supporting Information Available

All data supporting this study is provided in the Supporting Information. The Supporting Information is available free of charge at https://pubs.acs.org/doi/10.1021/acsomega.2c03739.

Full computational methods, model statistics and for each compound, energies, free energies, Cartesian coordinates, and the number of imaginary frequencies (PDF)

## Notes

All conformational searches were performed with the Maestro Suite using Schrodinger’s Macromodel (Ver. 12.3, https://www.schrodinger.com/products/macromodel). All DFT calculations were performed using Gaussian 16 (Rev A.03, https://gaussian.com/gaussian16/). Thermochemistry was obtained using the freely available GoodVibes package (Ver. 2.0.3, https://github.com/patonlab/GoodVibes/releases/tag/v2.0.3). The Cartesian coordinates of each structure have been included in the Supporting Information.

## References

[ref1] Yadav J. S.; Reddy B. V. S.; Baishya G. Green Protocol for Conjugate Addition of Thiols to α,β -Unsaturated Ketones Using a [Bmim] PF6/H2O System. J. Org. Chem. 2003, 68, 7098–7100. 10.1021/jo034335l. 12946157

[ref2] Krishna P. R.; Sreeshailam A.; Srinivas R. Recent Advances and Applications in Asymmetric Aza-Michael Addition Chemistry. Tetrahedron 2009, 65, 9657–9672. 10.1016/j.tet.2009.08.021.

[ref3] Sundararajan G.; Prabagaran N. A New Polymer-Anchored Chiral Catalyst for Asymmetric Michael Addition Reactions. Org. Lett. 2001, 3, 389–392. 10.1021/ol006898e. 11428021

[ref4] Chan J. W.; Hoyle C. E.; Lowe A. B.; Bowman M. Nucleophile-Initiated Thiol-Michael Reactions: Effect of Organocatalyst, Thiol, and Ene. Macromolecules 2010, 43, 6381–6388. 10.1021/ma101069c.

[ref5] Nair D. P.; Podgórski M.; Chatani S.; Gong T.; Xi W.; Fenoli C. R.; Bowman C. N. The Thiol-Michael Addition Click Reaction: A Powerful and Widely Used Tool in Materials Chemistry. Chem. Mater. 2014, 26, 724–744. 10.1021/cm402180t.

[ref6] Chauhan P.; Mahajan S.; Enders D. Organocatalytic Carbon-Sulfur Bond-Forming Reactions. Chem. Rev. 2014, 114, 8807–8864. 10.1021/cr500235v. 25144663

[ref7] Duplan V.; Hoshino M.; Li W.; Honda T.; Fujita M. In Situ Observation of Thiol Michael Addition to a Reversible Covalent Drug in a Crystalline Sponge. Angew. Chem. Int. Ed. 2016, 128, 5003–5007. 10.1002/ange.201509801. 26970084

[ref8] Sun Y.; Liu H.; Cheng L.; Zhu S.; Cai C.; Yang T.; Yang L.; Ding P. Thiol Michael Addition Reaction: A Facile Tool for Introducing Peptides into Polymer-Based Gene Delivery Systems. Polym. Int. 2018, 67, 25–31. 10.1002/pi.5490.

[ref9] Furuhama A.; Aoki Y.; Shiraishi H. Consideration of Reactivity to Acute Fish Toxicity of α,β-Unsaturated Carbonyl Ketones and Aldehydes. SAR QSAR Environ. Res. 2012, 23, 169–184. 10.1080/1062936X.2011.636381. 22150015

[ref10] Ebbrell D. J.; Madden J. C.; Cronin M. T. D.; Schultz T. W.; Enoch S. J. Development of a Fragment-Based in Silico Profiler for Michael Addition Thiol Reactivity. Chem. Res. Toxicol. 2016, 29, 1073–1081. 10.1021/acs.chemrestox.6b00099. 27100370

[ref11] Chatani S.; Wang C.; Podgórski M.; Bowman C. N. Triple Shape Memory Materials Incorporating Two Distinct Polymer Networks Formed by Selective Thiol-Michael Addition Reactions. Macromolecules 2014, 47, 4949–4954. 10.1021/ma501028a.

[ref12] Hahn M. E. Mechanistic Research in Aquatic Toxicology: Perspectives and Future Directions. Aquat. Toxicol. 2011, 105, 67–71. 10.1016/j.aquatox.2011.06.001. 22099346 PMC3220193

[ref13] Patlewicz G.; Aptula A. O.; Uriarte E.; Roberts D. W.; Kern P. S.; Gerberick G. F.; Kimber I.; Dearman R. J.; Ryan C. A.; Basketter D. A. An Evaluation of Selected Global (Q)SARs/Expert Systems for the Prediction of Skin Sensitisation Potential. SAR QSAR Environ. Res. 2007, 18, 515–541. 10.1080/10629360701427872. 17654336

[ref14] Townsend P. A.; Grayson M. N. Density Functional Theory in the Prediction of Mutagenicity: A Perspective. Chem. Res. Toxicol. 2021, 34, 179–188. 10.1021/acs.chemrestox.0c00113. 32643924 PMC7887799

[ref15] Jackson P. A.; Widen J. C.; Harki D. A.; Brummond K. M. Covalent Modifiers: A Chemical Perspective on the Reactivity of α,β-Unsaturated Carbonyls with Thiols via Hetero-Michael Addition Reactions. J. Med. Chem. 2017, 60, 839–885. 10.1021/acs.jmedchem.6b00788. 27996267 PMC5308545

[ref16] Baillie T. A. Targeted Covalent Inhibitors for Drug Design. Angew. Chem. Int. Ed. 2016, 55, 13408–13421. 10.1002/anie.201601091. 27539547

[ref17] Schultz T. W.; Carlson R. E.; Cronin M. T. D.; Hermens J. L. M.; Johnson R.; O’Brien P. J.; Roberts D. W.; Siraki A.; Wallace K. B.; Veith G. D. A Conceptual Framework for Predicting the Toxicity of Reactive Chemicals: Modeling Soft Electrophilicity. SAR QSAR Environ. Res. 2006, 17, 413–428. 10.1080/10629360600884371. 16920662

[ref18] Böhme A.; Thaens D.; Paschke A.; Schürmann G. Kinetic Glutathione Chemoassay to Quantify Thiol Reactivity of Organic Electrophiless - Application to α,β-Unsaturated Ketones, Acrylates, and Propiolates. Chem. Res. Toxicol. 2009, 22, 742–750. 10.1021/tx800492x. 19317512

[ref19] Schultz T. W.; Yarbrough J. W.; Johnson E. L. Structure-Activity Relationships for Reactivity of Carbonyl-Containing Compounds with Glutathione. SAR QSAR Environ. Res. 2005, 16, 313–322. 10.1080/10659360500204152. 16234173

[ref20] Böhme A.; Laqua A.; Schürmann G. Chemoavailability of Organic Electrophiles: Impact of Hydrophobicity and Reactivity on Their Aquatic Excess Toxicity. Chem. Res. Toxicol. 2016, 29, 952–962. 10.1021/acs.chemrestox.5b00398. 27096880

[ref21] Schwöbel J. A. H.; Madden J. C.; Cronin M. T. D. Examination of Michael Addition Reactivity towards Glutathione by Transition-State Calculations. SAR QSAR Environ. Res. 2010, 21, 693–710. 10.1080/1062936X.2010.528943. 21120757

[ref22] Schwöbel J. A. H.; Wondrousch D.; Koleva Y. K.; Madden J. C.; Cronin M. T. D.; Schürmann G. Prediction of Michael-Type Acceptor Reactivity toward Glutathione. Chem. Res. Toxicol. 2010, 23, 1576–1585. 10.1021/tx100172x. 20882991

[ref23] Mulliner D.; Wondrousch D.; Schürmann G. Predicting Michael-Acceptor Reactivity and Toxicity through Quantum Chemical Transition-State Calculations. Org. Biomol. Chem. 2011, 9, 8400–8412. 10.1039/c1ob06065a. 22048735

[ref24] Townsend P. A.; Grayson M. N. Reactivity Prediction in Aza-Michael Additions without Transition State Calculations: The Ames Test for Mutagenicity. Chem. Commun. 2020, 56, 13661–13664. 10.1039/d0cc05681b. 33073273

[ref25] Enoch S. J.; Roberts D. W. Predicting Skin Sensitization Potency for Michael Acceptors in the LLNA Using Quantum Mechanics Calculations. Chem. Res. Toxicol. 2013, 26, 767–774. 10.1021/tx4000655. 23611145

[ref26] Ebbrell D. J.; Madden J. C.; Cronin M. T. D.; Schultz T. W.; Enoch S. J. Validation of a Fragment-Based Profiler for Thiol Reactivity for the Prediction of Toxicity: Skin Sensitization and Tetrahymena Pyriformis. Chem. Res. Toxicol. 2017, 30, 604–613. 10.1021/acs.chemrestox.6b00361. 28045255

[ref27] Ayala P.; Schlegel H. A Combined Method for Determining Reaction Paths, Minima and Transition State Geometries. J. Chem. Phys. 1997, 107, 375–384. 10.1063/1.474398.

[ref28] Frisch M. J.; Trucks G. W.; Schlegel H. B.; Scuseria G. E.; Robb M. A.; Cheeseman J. R.; Scalmani G.; Barone V.; Mennucci B.; Petersson G. A.; Nakatsuji H.; Caricato M.; Li X.; Hratchian H. P.; Izmaylov A. F.; Bloino J.; Zheng J.; Sonnenberg J. L.; Hada M.; Ehara M.; Toyota K.; Fukuda R.; Hasegawa J.; Ishida M.; Nakajima T.; Honda Y.; Kitao O.; Nakai H.; Vreven T.; Montgomery J. A.; Peralta J. E.; Ogliaro F.; Bearpark M.; Heyd J. J.; Brothers E.; Kudin K. N.; Staroverov V. N.; Kobayashi R.; Normand J.; Raghavachari K.; Rendell J. C. A.; Burant S.; Iyengar S.; Tomasi J.; Cossi M.; Rega N.; Millam J. M.; Klene M.; Knox J. E.; Cross J. B.; Bakken V.; Adamo C.; Jaramillo J.; Gomperts R.; Stratmann R. E.; Yazyev O.; Austin A. J.; Cammi R.; Pomelli C.; Ochterski J. W.; Martin R. L.; Morokuma K.; Zakrzewski V. G.; Voth G. A.; Salvador P.; Dannenberg J. J.; Dapprich S.; Daniels A. D.; Farkas O.; Foresman J. B.; Ortiz J. V.; Cioslowski J.; Fox D. J. Gaussian 16, Revision A.03; Gaussian, Inc.: Wallingford, CT, 2016.

[ref29] Lam Y. H.; Grayson M. N.; Holland M. C.; Simon A.; Houk K. N. Theory and Modeling of Asymmetric Catalytic Reactions. Acc. Chem. Res. 2016, 49, 750–762. 10.1021/acs.accounts.6b00006. 26967569

[ref30] Fordham J. M.; Grayson M. N.; Aggarwal V. K. Vinylidene Homologation of Boronic Esters and Its Application to the Synthesis of the Proposed Structure of Machillene. Angew. Chem. Int. Ed. 2019, 131, 15412–15416. 10.1002/ange.201907617. 31365776

[ref31] Falcone B. N.; Grayson M. N.; Rodriguez J. B. Mechanistic Insights into a Chiral Phosphoric Acid-Catalyzed Asymmetric Pinacol Rearrangement. J. Org. Chem. 2018, 83, 14683–14687. 10.1021/acs.joc.8b02812. 30433780

[ref32] Townsend P. A.; Grayson M. N. Density Functional Theory Transition-State Modeling for the Prediction of Ames Mutagenicity in 1,4 Michael Acceptors. J. Chem. Inf. Model. 2019, 59, 5099–5103. 10.1021/acs.jcim.9b00966. 31774671

[ref33] Pedregosa F.; Varoquaux G.; Gramfort A.; Michel V.; Thirion B.; Grisel O.; Blondel M.; Prettenhofer P.; Weiss R.; Dubourg V.; Vanderplas J.; Passos A.; Cournapeau D.; Brucher M.; Perrot M.; Duchesnay É. Scikit-Learn: Machine Learning in Python. J. Mach. Learn. Res. 2011, 12, 2825–2830. 10.5555/1953048.2078195.

[ref34] Pasha F. A.; Srivastava H. K.; Singh P. P. Comparative QSAR Study of Phenol Derivatives with the Help of Density Functional Theory. Bioorg. Med. Chem. 2005, 13, 6823–6829. 10.1016/j.bmc.2005.07.064. 16169734

[ref35] Zhu M.; Ge F.; Zhu R.; Wang X.; Zheng X. A DFT-Based QSAR Study of the Toxicity of Quaternary Ammonium Compounds on Chlorella Vulgaris. Chemosphere 2010, 80, 46–52. 10.1016/j.chemosphere.2010.03.044. 20417544

[ref36] Trohalaki S.; Pachter R. Quantum Descriptors for Predictive Toxicology of Halogenated Aliphatic Hydrocarbons. SAR QSAR Environ. Res. 2003, 14, 131–143. 10.1080/1062936031000073153. 12747571

[ref37] Jorner K.; Tomberg A.; Bauer C.; Sköld C.; Norrby P. O. Organic Reactivity from Mechanism to Machine Learning. Nat. Rev. 2021, 5, 240 10.1038/s41570-021-00260-x. 37117288

[ref38] Bondi A. Van Der Waals Volumes and Radii. J. Phys. Chem. 1964, 68, 441–451. 10.1021/j100785a001.

[ref39] Gwee E. S. H.; Seeger Z. L.; Appadoo D. R. T.; Wood B. R.; Izgorodina E. I. Influence of DFT Functionals and Solvation Models on the Prediction of Far-Infrared Spectra of Pt-Based Anticancer Drugs: Why Do Different Complexes Require Different Levels of Theory?. ACS Omega 2019, 4, 5254–5269. 10.1021/acsomega.8b03455. 31459697 PMC6649127

[ref40] Hou G.; Zhu X.; Cui Q. An Implicit Solvent Model for SCC-DFTB with Charge-Dependent Radii. J. Chem. 2010, 6, 2303–2314. 10.1021/ct1001818. PMC2918909 20711513

[ref41] Agmon N. Quantitative Hammond Postulate. J. Chem. Soc. 1978, 74, 388–404. 10.1039/F29787400388.

[ref42] Northrop B. H.; Frayne S. H.; Choudhary U. Thiol-Maleimide “Click” Chemistry: Evaluating the Influence of Solvent, Initiator, and Thiol on the Reaction Mechanism, Kinetics, and Selectivity. Polym. Chem. 2015, 6, 3415–3430. 10.1039/c5py00168d.

[ref43] Grüber R.; Fleurat-Lessard P. Performance of Recent Density Functionals to Discriminate between Olefin and Nitrogen Binding to Palladium. Theor. Chem. Acc. 2014, 133, 1–10. 10.1007/s00214-014-1533-2.

[ref44] Dewar M. J. S.; Zoebisch E. G.; Healy E. F.; Stewart J. J. P. AM1: A New General Purpose Quantum Mechanical Molecular Model. J. Am. Chem. Soc. 1985, 107, 3902–3909. 10.1021/ja00299a024.

[ref45] Spencer S. R.; Xue L.; Klenz E. M.; Talalay P. The potency of inducers of NAD(P)H:(quinone-acceptor) oxidoreductase parallels their efficiency as substrates for glutathione transferases. Structural and electronic correlations. Biochem. J. 1991, 273, 711–717. 10.1042/bj2730711. 1900000 PMC1150218

