# Supplementary material for: Eliminating Transition State Calculations for Faster and More Accurate Reactivity Prediction in Sulfa-Michael Additions Relevant to Human Health and the Environment
Source: ACS Omega. 2022 Jul 21;7(30):26945–51. doi: 10.1021/acsomega.2c03739 (PMC9352231; doi:10.1021/acsomega.2c03739)
Supplement: Supplementary file 1 — ao2c03739_si_001.pdf [file ao2c03739_si_001.pdf]

Supplementary Information (SI)

Eliminating Transition State Calculations for Faster and More Accurate Reactivity Prediction in Sulfa-Michael Additions Relevant to Human Health and the Environment

Piers A. Townsend[a,b], Elliot H. E. Farrar[b] and Matthew N. Grayson[b,*]

[a]Centre for Sustainable Chemical Technologies, Department of Chemistry, University of Bath, Claverton Down, Bath, BA2 7AY, United Kingdom

[b]Department of Chemistry, University of Bath, Claverton Down, Bath, BA2 7AY, United Kingdom

Contents

Full Computational Methods S3

Full Regression Statistics S4–S5

Additional Figures S6

References S7

Aldehyde Structures – DFT .... S8 – S144

Ester Structures - DFT..S145 – S390

Ketone Structures – DFT ....S391 – S493

Aldehyde Structures – SQM ....S494 – S570

Ester Structures – SQM ....S571 – S711

Ketone Structures – SQM ....S712 – S767

Nucleophile Structures ....S768 – S769

Protonation Extension ....S770 – S828

1. Full Computational Methods

Kinetic glutathione assay data (log(k_{GSH})) for 23 1,4 Michael acceptors (MAs) was taken from work published by Bohme *et al.*, providing experimental rate data for: nine esters, seven aldehydes and seven ketones (see section 3, Table S1).[1] Minor truncation was performed on two compounds. 1-pentene-3-one was truncated to methyl vinyl ketone and trans-2-pentenal to but-2-enal. Conformational searches and redundant conformer eliminations were performed on all 23 MAs using Schrödinger's MacroModel (Ver. 12.3).[2] Molecular energies were calculated with the OPLS3e force field and the Polak-Ribière conjugate gradient (PRCG) algorithm was used for the optimization procedures.[3]

In 2011, it was reported by Krenske *et al* that the rate-determining step in the addition of thiols to α,β-unsaturated ketones is the addition of methanethiolate to the corresponding enone.[4] This paper also reported that M06-2X and a large basis set (triple-ζ) gave thermodynamic data within 1 kcal mol^{-1} of CBS-QB3 benchmark data. Therefore, structures obtained from MacroModel were optimised using DFT calculations performed with Gaussian 16 (Rev. A.03)[5] at the M06-2X/def2-TZVPP level of theory under the IEF-PCM implicit solvation model (water), which has been used extensively for modelling organic chemical reactions.[6–8] In line with previous studies, and to ensure computational feasibility, methanethiolate was used as a model nucleophile in all calculations.[9,10] Following DFT calculations, reactant and intermediate structures were optimised with the SQM AM1 method. Corrections to the free energy were obtained with GoodVibes using the quasi-harmonic approximation as described by Grimme.[11] In GoodVibes, a vibrational scaling factor of 1, a temperature of 298.15 K, and a concentration of 1 mol dm^{-3} were used. Electronic supplementary information was created using ESIGen.[12]

All regression models were developed via the Scikit-learn python package.[13] The 23 compounds were split at a 75:25 ratio into a training set of 17 compounds and a test set of 6 compounds and the training set used to fit an ordinary least squares linear regression model. This model was validated externally based on the log(k_{GSH}) values predicted using the test set. 2-fold cross validation was also performed within the training set in order to test the stability of the model and to identify any overfitting. Thus, the performance of a model could be assessed by its mean absolute error (MAE) for both external validation (test set predictions) and cross validation (training set). Pearson correlation coefficients (r^2) were also calculated between the predicted and experimental log(k_{GSH}) for each model, providing a further source of external validation (test set predictions, see section 2 below). Due to the small size of the dataset (n=23), MAEs and r^2 scores were calculated at 20 different random 75:25 train-test splitting's of the data and the scores averaged. For each model, a single train-test splitting was then located with individual MAEs and r^2 scores that closely matched the average scores. This ensured that the scores presented matched the average performance of the model. The average scores are included in the supplementary information, whilst the scores for the individual models are presented in the manuscript.

2. Full Regression Metrics

Linear regression of log(k_{GSH}) with the activation energy derived from transition state structures (M06-2X/def2-TZVPP-IEFPCM(water)).

Averaged over 20 train-test splits:
- Average MAE: 0.70
- Average 2-Fold MAE: 0.69
- Average r2: 0.65

Representative train-test split:
- MAE: 0.69
- 2-Fold MAE: 0.67
- r2: 0.49

Linear regression of log(kGSH) with the intermediate energy differences (M06-2X/def2-TZVPP-IEFPCM(water).

Averaged over 20 train-test splits:
- Average MAE: 0.48
- Average 2-Fold MAE: 0.50
- Average r2: 0.81

Representative train-test split:
- MAE: 0.48
- 2-Fold MAE: 0.45
- r2: 0.76

Linear regression of log(kGSH) with key atomic charges of the MA (M06-2X/def2-TZVPP-IEFPCM(water).

Averaged over 20 train-test splits:
- Average MAE: 0.32
- Average 2-Fold MAE: 0.41
- Average r2: 0.89

Representative train-test split:
- MAE: 0.35
- 2-Fold MAE: 0.41
- r2: 0.88

Linear regression of log(kGSH) with key atomic charges of the MA (AM1).

Averaged over 20 train-test splits:
- Average MAE: 0.33
- Average 2-Fold MAE: 0.40
- Average r2: 0.90

Representative train-test split:
- MAE: 0.37
- 2-Fold MAE: 0.33
- r2: 0.89

Multivariate Models

Except where noted, the following features were extracted for all MAs, intermediates, and TSs. Multivariate linear regression models were developed by combining up to 5 features from each subset (MAs, intermediates, TSs), fitting a model, and generating metrics (repeated over 20 train-test splits and averaged). No mixing of features occurred. For example, models were not fitted with a mixture of some TS features and some MA features.

**Feature**	**Source**
Activation energy (TSs only)	
Intermediate energy difference (intermediates only)	
Electronic Energy	Goodvibes
Gibbs Free Energy	Goodvibes
Enthalpy	Goodvibes
Entropy	Goodvibes
Quasiharmonic Entropy	Goodvibes
Zero-Point Energy	Goodvibes
Quasiharmonic Gibbs Free Energy	Goodvibes
Mulliken and APT atomic charges for key atoms, summed and not-summed	CCLIB[14]
Total Electrotopological State Index	RDKit[15,16]
Total Accessible Surface Area	RDKit[15,17]
Total Topological Polar Surface Area	RDKit[15,18]
Total Partial Equalization of Orbital Electronegativities (Partial Charges)	RDKit[15,19]
Molecular Chemical Potential	HSAB[20]
Molecular Electrophilic Index	HSAB[20]
Molecular Hardness	HSAB[20]
Molecular Softness	HSAB[20]

3. Additional Figures

Structure	CAS No	log(k_{GSH})	No of Conformations Found (TSs)	No of Conformations Found (HEI)	Activation Energy $\Delta G^{\ddagger}$ (kcal/mol)	Intermediate Energy Difference ΔG_{HEI} (kcal/mol)
**Ketones**						
1-pentene-3-one*	1629−58−9	**3.1**	6	6	10.37	2.42
3-pentene-2-one	625−33−2	**1.43**	7	7	10.12	3.12
2-cyclopentene-1-one	930−30−3	**1.41**	3	3	12.71	7.15
4-hexene-3-one	2497-21-4	**1.38**	19	19	13.96	6.78
3-methyl-3-pentene-2-one	565−62−8	**-0.11**	7	7	14.39	10.61
4-methyl-3-pentene-2-one	141−79−7	**-0.68**	6	6	15.78	9.33
3-methyl-2-cyclopentene-1-one	2758−18−1	**-1.13**	3	3	15.89	11.62
**Aldehydes**						
methacrylaldehyde	78−85−3	**2.31**	8	8	10.05	2.75
2-ethyl acrolein	922−63−4	**1.77**	11	11	10.81	3.37
trans-2-pentenal*	1576−87−0	**1.45**	8	8	11.61	3.59
4-methyl-2-pentenal	5362−56−1	**1.03**	14	14	11.69	3.94
trans,trans-2,4-hexadienal	142−83−6	**0.83**	11	11	13.23	7.45
3-methyl-2-butenal	107−86−8	**0.23**	8	8	13.99	7.14
trans-2-methyl-2-butenal	497−03−0	**-0.32**	7	7	12.47	6.33
**Esters**						
methyl acrylate	96-33-3	**1.06**	8	8	11.30	6.19
n-propyl acrylate	925-60-0	**1.01**	28	27	11.70	6.55
iso-butyl acrylate	106-63-8	**0.97**	21	22	9.65	6.38
tert-butyl acrylate	1663-39-4	**0.4**	8	8	12.19	7.77
ethyl crotonate	623-70-1	**-0.79**	12	14	14.44	9.76
methyl crotonate	623-43-8	**-0.79**	8	8	14.14	9.86
methyl methacrylate	80-62-6	**-1.14**	6	6	14.02	10.16
ethyl methacrylate	97-63-2	**-1.24**	12	12	14.07	10.43
methyl tiglate	6622-76-0	**-2.15**	6	6	16.08	13.28

Table S1. CAS No, kinetic glutathione chemoassay data, number of conformers, activation energies and intermediate energy differences for each structure included in this study. Compounds marked with an asterisk (*) have been truncated as described in the computational methods.

Compound Name	log(k_{GSH})	No. of TS Conformations	Barrier 1 (TS-1)	Barrier 2 (TS-2)
1-pentene-3-one	**3.1**	11	10.37	6.93
4-methyl-3-pentene-2-one	**-0.68**	11	15.78	7.42
methacrylaldehyde	**2.31**	8	10.05	6.80
3-methyl-2-butenal	**0.23**	9	13.99	8.06
methyl acrylate	**1.06**	12	11.30	5.65
methyl tiglate	**-2.15**	9	16.08	4.21

Table S*2*. For six compounds, activation barriers were calculated for the protonation step (TS-2). The number of TS conformers and TS barriers (TS-1 and TS-2) can be seen in the table.

4. References

(1) Böhme, A.; Laqua, A.; Schüürmann, G. Chemoavailability of Organic Electrophiles: Impact of Hydrophobicity and Reactivity on Their Aquatic Excess Toxicity. *Chem. Res. Toxicol.* **2016**, *29* (6), 952–962. https://doi.org/10.1021/acs.chemrestox.5b00398.

(2) Schrödinger. Macromodel. New York 2019.

(3) Roos, K.; Wu, C.; Damm, W.; Reboul, M.; Stevenson, J. M.; Lu, C.; Dahlgren, M. K.; Mondal, S.; Chen, W.; Wang, L.; Abel, R.; Friesner, R. A.; Harder, E. D. OPLS3e: Extending Force Field Coverage for Drug-Like Small Molecules. *J. Chem. Theory Comput.* **2019**, *15* (3), 1863–1874. https://doi.org/10.1021/acs.jctc.8b01026.

(4) Krenske, E. H.; Petter, R. C.; Zhu, Z.; Houk, K. N. Transition States and Energetics of Nucleophilic Additions of Thiols to Substituted α,β-Unsaturated Ketones: Substituent Effects Involve Enone Stabilization, Product Branching, and Solvation. *J. Org. Chem.* **2011**, *76* (12), 5074–5081. https://doi.org/10.1021/jo200761w.

(5) Frisch, M. J.; Trucks, G. W.; Schlegel, H. B.; Scuseria, G. E.; Robb, M. A.; Cheeseman, J. R.; Scalmani, G.; Barone, V.; Mennucci, B.; Petersson, G. A.; Nakatsuji, H.; Caricato, M.; Li, X.; Hratchian, H. P.; Izmaylov, A. F.; Bloino, J.; Zheng, J.; Sonnenberg, J. L.; Hada, M.; Ehara, M.; Toyota, K.; Fukuda, R.; Hasegawa, J.;

Ishida, M.; Nakajima, T.; Honda, Y.; Kitao, O.; Nakai, H.; Vreven, T.; Montgomery, J. A.; Peralta, J. E.; Ogliaro, F.; Bearpark, M.; Heyd, J. J.; Brothers, E.; Kudin, K. N.; Staroverov, V. N.; Kobayashi, R.; Normand, J.; Raghavachari, K.; A. Rendell, J. C.; Burant, S.; Iyengar, S.; Tomasi, J.; Cossi, M.; Rega, N.; Millam, J. M.; Klene, M.; Knox, J. E.; Cross, J. B.; Bakken, V.; Adamo, C.; Jaramillo, J.; Gomperts, R.; Stratmann, R. E.; Yazyev, O.; Austin, A. J.; Cammi, R.; Pomelli, C.; Ochterski, J. W.; Martin, R. L.; Morokuma, K.; Zakrzewski, V. G.; Voth, G. A.; Salvador, P.; Dannenberg, J. J.; Dapprich, S.; Daniels, A. D.; Farkas, O.; Foresman, J. B.; Ortiz, J. V.; Cioslowski, J.; Fox, D. J. Gaussian 16, Revision A.03. Gaussian, Inc.: Wallingford CT 2016.

(6) Lam, Y. H.; Grayson, M. N.; Holland, M. C.; Simon, A.; Houk, K. N. Theory and Modeling of Asymmetric Catalytic Reactions. *Acc. Chem. Res.* **2016**, *49* (4), 750–762. https://doi.org/10.1021/acs.accounts.6b00006.

(7) Fordham, J. M.; Grayson, M. N.; Aggarwal, V. K. Vinylidene Homologation of Boronic Esters and Its Application to the Synthesis of the Proposed Structure of Machillene. *Angew. Chemie Int. Ed.* **2019**, *131* (43), 15412–15416. https://doi.org/10.1002/ange.201907617.

(8) Falcone, B. N.; Grayson, M. N.; Rodriguez, J. B. Mechanistic Insights into a Chiral Phosphoric Acid-Catalyzed Asymmetric Pinacol Rearrangement. *J. Org. Chem.* **2018**, *83* (23), 14683–14687. https://doi.org/10.1021/acs.joc.8b02812.

(9) Enoch, S. J.; Roberts, D. W. Predicting Skin Sensitization Potency for Michael Acceptors in the LLNA Using Quantum Mechanics Calculations. *Chem. Res. Toxicol.* **2013**, *26* (5), 767–774. https://doi.org/10.1021/tx4000655.

(10) Ebbrell, D. J.; Madden, J. C.; Cronin, M. T. D.; Schultz, T. W.; Enoch, S. J. Development of a Fragment-Based in Silico Profiler for Michael Addition Thiol Reactivity. *Chem. Res. Toxicol.* **2016**, *29* (6), 1073–1081. https://doi.org/10.1021/acs.chemrestox.6b00099.

(11) Luchini, G.; Alegre-Requena, J. V.; Funes-Ardoiz, I.; Paton, R. S. GoodVibes: Automated Thermochemistry for Heterogeneous Computational Chemistry Data. *F1000Research* **2020**, *9* (May), 291. https://doi.org/10.12688/f1000research.22758.1.

(12) Rodríguez-Guerra Pedregal, J.; Gómez-Orellana, P.; Maréchal, J. D. ESIgen: Electronic Supporting Information Generator for Computational Chemistry Publications. *J. Chem. Inf. Model.* **2018**, *58* (3), 561–564. https://doi.org/10.1021/acs.jcim.7b00714.

(13) Pedregosa, F.; Varoquaux, G.; Gramfort, A.; Michel, V.; Thirion, B.; Grisel, O.; Blondel, M.; Prettenhofer, P.; Weiss, R.; Dubourg, V.; Vanderplas, J.; Passos, A.; Cournapeau, D.; Brucher, M.; Perrot, M.; Duchesnay, É. Scikit-Learn: Machine Learning in Python. *J. Mach. Learn. Res.* **2011**, *12* (85), 2825–2830. https://doi.org/10.5555/1953048.2078195.

(14) O'Boyle, N. M.; Tenderholt, A. L.; Langner, K. M. Cclib: A Library for Package-independent Computational Chemistry Algorithms. *J. Comput. Chem.* **2007**, *29* (5), 839–845. https://doi.org/10.1002/jcc.20823.

(15) RDKit: Open-Source Cheminformatics.

(16) Hall, L. H.; Mohney, B.; Kier, L. B. Electrotopological State Indices for Atom Types: A Novel Combination of Electronic, Topological, and Valence State Information. *Mol. Inform.* **1991**, *10* (1), 43–51. https://doi.org/10.1002/qsar.19910100108.

(17) Labute, P. ScienceDirect - Journal of Molecular Graphics and Modelling : A Widely Applicable Set of Descriptors. *J. Mol. Graph. Model.* **2000**, *3263* (Figure 1), 464–477.

(18) Ertl, P.; Rohde, B.; Selzer, P. Fast Calculation of Molecular Polar Surface Area as a Sum of Fragment-Based Contributions and Its Application to the Prediction of Drug Transport Properties. *J. Med. Chem.* **2000**, *43* (20), 3714–3717. https://doi.org/10.1021/jm000942e.

(19) Gasteiger, J.; Marsili, M. Iterative Partial Equalization of Orbital Electronegativity-a Rapid Access to Atomic Charges. *Tetrahedron* **1980**, *36* (22), 3219–3228. https://doi.org/10.1016/0040-4020(80)80168-2.

(20) LoPachin, R. M.; Gavin, T.; DeCaprio, A.; Barber, D. S. Application of the Hard and Soft, Acids and Bases (HSAB) Theory to Toxicant - Target Interactions. *Chem. Res. Toxicol.* **2012**, *25* (2), 239–251. https://doi.org/10.1021/tx2003257.

Created using ESIgen v0.0.5

ESIgen is scientific software, funded by public research grants and published as:

```
J Rodriguez-Guerra, P Gomez-Orellana, JD Marechal.
J. Chem. Inf. Model., 2018, 58 (3), pp 561564.
DOI: 10.1021/acs.jcim.7b00714.
```

If you make use of ESIgen in scientific publications, please cite us in the main text! References only mentioned in SI documents are not indexed by citation engines.

1_methylacrolein_conf2_min

Datum	Value
M06-2X/def2tzvpp-IEFPCM(water) Energy	-231.224444
M06-2X/def2tzvpp-IEFPCM(water) Free Energy (Quasiharmonic)	-231.159496
Number of Imaginary Frequencies	0

Frequencies (Top 3 out of 27)

```
  1.     162.8136 cm-1
  2.     186.7364 cm-1
  3.     267.3495 cm-1
```

M06-2X/def2tzvpp-IEFPCM(water) Molecular Geometry in Cartesian Coordinates

```
C        -1.488416      -0.947821      -0.000050
H        -1.295875      -2.013946      -0.000048
H        -2.522579      -0.629191      -0.000082
C        -0.483058      -0.074945      -0.000016
C         0.886248      -0.627889       0.000026
H         0.952205      -1.729573       0.000023
O         1.885483       0.051408       0.000061
C        -0.626254       1.413151      -0.000016
H        -0.137437       1.841181       0.875870
H        -0.137385       1.841186      -0.875871
H        -1.673908       1.704105      -0.000046
```

1_methylacrolein_HEI_1

Datum	Value
M06-2X/def2tzvpp-IEFPCM(water) Energy	-669.445937
M06-2X/def2tzvpp-IEFPCM(water) Free Energy (Quasiharmonic)	-669.346859
Number of Imaginary Frequencies	0

Frequencies (Top 3 out of 42)

```
1.      53.5027 cm-1
2.      98.2001 cm-1
3.     157.6711 cm-1
```

M06-2X/def2tzvpp-IEFPCM(water) Molecular Geometry in Cartesian Coordinates

```
C         -1.795566       -0.587764       -0.146618
C         -1.002628        0.501571        0.099758
C          0.156625        0.388712        1.017382
O         -1.701804       -1.778541        0.314185
H          0.249566        1.247960        1.687442
H          0.087078       -0.518081        1.616724
C          1.587141       -1.130589       -0.820198
H          1.618040       -2.026782       -0.203154
H          0.624220       -1.072722       -1.326297
H          2.388800       -1.168613       -1.554203
S          1.808656        0.351363        0.179546
H         -2.637992       -0.380029       -0.840503
C         -1.237374        1.824088       -0.569068
H         -1.385469        2.637055        0.152362
H         -2.123927        1.784727       -1.205592
H         -0.393569        2.126888       -1.200522
```

1_methylacrolein_HEI_2

Datum	Value
M06-2X/def2tzvpp-IEFPCM(water) Energy	-669.446447
M06-2X/def2tzvpp-IEFPCM(water) Free Energy (Quasiharmonic)	-669.348159
Number of Imaginary Frequencies	0

Frequencies (Top 3 out of 42)

```
1.      37.9712 cm-1
2.      80.5166 cm-1
3.     110.1660 cm-1
```

M06-2X/def2tzvpp-IEFPCM(water) Molecular Geometry in Cartesian Coordinates

```
C         -1.668595        0.665640        0.425978
C         -0.860345       -0.426995        0.249741
C          0.406965       -0.541433        1.004051
O         -2.772191        0.953715       -0.151915
H          0.456060        0.190401        1.812304
H          0.564669       -1.535271        1.433210
C          1.658696        1.368886       -0.587140
H          0.705627        1.412255       -1.111708
H          1.642734        2.071411        0.245489
H          2.464138        1.636058       -1.267346
S          1.956372       -0.306439       -0.001182
H         -1.296041        1.380926        1.190452
C         -1.180339       -1.491547       -0.757491
H         -2.206382       -1.380245       -1.107898
H         -0.523495       -1.452465       -1.634956
H         -1.070025       -2.497080       -0.336144
```

1_methylacrolein_HEI_3

Datum	Value
M06-2X/def2tzvpp-IEFPCM(water) Energy	-669.442564
M06-2X/def2tzvpp-IEFPCM(water) Free Energy (Quasiharmonic)	-669.343355
Number of Imaginary Frequencies	0

Frequencies (Top 3 out of 42)

```
1.      62.3601 cm-1
2.      82.7731 cm-1
3.     144.6139 cm-1
```

M06-2X/def2tzvpp-IEFPCM(water) Molecular Geometry in Cartesian Coordinates

```
C          2.235860       0.072582      -0.151597
C          0.964323       0.492579       0.134320
C         -0.027166      -0.461948       0.692018
O          2.756127      -1.090943      -0.019166
H          0.455038      -1.419112       0.884679
H         -0.484337      -0.114011       1.624474
C         -2.656329       0.387587       0.167788
H         -2.865360       0.259328       1.228514
H         -2.284933       1.393975      -0.010604
H         -3.577123       0.242388      -0.393249
S         -1.473263      -0.856007      -0.392850
H          2.892449       0.876486      -0.547250
C          0.516763       1.899130      -0.131382
H         -0.243814       1.955363      -0.920206
H          1.357471       2.519571      -0.449445
H          0.073107       2.370083       0.755144
```

1_methylacrolein_HEI_4_reopt

Datum	Value
M06-2X/def2tzvpp-IEFPCM(water) Energy	-669.443215
M06-2X/def2tzvpp-IEFPCM(water) Free Energy (Quasiharmonic)	-669.344318
Number of Imaginary Frequencies	0

Frequencies (Top 3 out of 42)

```
  1.      50.2597 cm-1
  2.      81.4307 cm-1
  3.     140.8306 cm-1
```

M06-2X/def2tzvpp-IEFPCM(water) Molecular Geometry in Cartesian Coordinates

```
C         -2.107557      -0.575483       0.046864
C         -0.940546       0.103268       0.277348
C          0.256388      -0.617723       0.769444
O         -3.225588      -0.132636      -0.390679
H          0.704115      -0.163043       1.659963
H          0.012127      -1.652967       1.011168
C          2.614986       0.743206       0.030581
H          2.038185       1.644847      -0.161594
H          2.899521       0.709405       1.080976
```

```
H          3.516634       0.760223       -0.578048
S          1.683582      -0.738121       -0.415370
H         -2.053188      -1.658118        0.290397
C         -0.810444       1.570429       -0.005274
H         -1.792758       2.008102       -0.182331
H         -0.339555       2.106325        0.827330
H         -0.194648       1.774066       -0.890285
```

1_methylacrolein_HEI_5

Datum	Value
M06-2X/def2tzvpp-IEFPCM(water) Energy	-669.444643
M06-2X/def2tzvpp-IEFPCM(water) Free Energy (Quasiharmonic)	-669.345843
Number of Imaginary Frequencies	0

Frequencies (Top 3 out of 42)

```
 1.      64.5044 cm-1
 2.      69.2061 cm-1
 3.     133.8647 cm-1
```

M06-2X/def2tzvpp-IEFPCM(water) Molecular Geometry in Cartesian Coordinates

```
C         -2.088236      -0.559327       -0.117311
C         -1.173400       0.409488        0.194917
C          0.165717       0.022546        0.711318
O         -1.969358      -1.831206       -0.033272
H          0.532826       0.713664        1.475398
H          0.144345      -0.987481        1.119428
C          2.917060      -0.282987        0.340933
H          3.072588       0.508170        1.072921
H          2.843618      -1.240922        0.853491
H          3.765749      -0.308710       -0.338955
S          1.428250       0.034702       -0.628735
H         -3.059344      -0.163550       -0.482730
C         -1.454308       1.871485        0.000777
H         -1.363486       2.442057        0.933019
H         -2.467571       2.022495       -0.377883
H         -0.766853       2.341455       -0.712555
```

1_methylacrolein_HEI_6_reopt

Datum	Value
M06-2X/def2tzvpp-IEFPCM(water) Energy	-669.443215
M06-2X/def2tzvpp-IEFPCM(water) Free Energy (Quasiharmonic)	-669.344319
Number of Imaginary Frequencies	0

Frequencies (Top 3 out of 42)

```
1.      50.1842 cm-1
2.      81.4468 cm-1
3.     140.8596 cm-1
```

M06-2X/def2tzvpp-IEFPCM(water) Molecular Geometry in Cartesian Coordinates

```
C          -2.107533       -0.575496        0.046809
C          -0.940528        0.103244        0.277350
C           0.256391       -0.617765        0.769455
O          -3.225551       -0.132618       -0.390737
H           0.704086       -0.163114        1.660006
H           0.012120       -1.653016        1.011137
C           2.614861        0.743327        0.030459
H           2.038048        1.644894       -0.162019
H           2.899221        0.709784        1.080911
H           3.516613        0.760253       -0.578019
S           1.683629       -0.738165       -0.415301
H          -2.053177       -1.658142        0.290291
C          -0.810450        1.570426       -0.005184
H          -1.792790        2.008126       -0.182036
H          -0.339408        2.106244        0.827383
H          -0.194810        1.774133       -0.890287
```

1_methylacrolein_HEI_7

Datum	Value
M06-2X/def2tzvpp-IEFPCM(water) Energy	-669.444692
M06-2X/def2tzvpp-IEFPCM(water) Free Energy (Quasiharmonic)	-669.346248
Number of Imaginary Frequencies	0

Frequencies (Top 3 out of 42)

```
1.      59.2241 cm-1
2.      72.6788 cm-1
3.     118.4455 cm-1
```

M06-2X/def2tzvpp-IEFPCM(water) Molecular Geometry in Cartesian Coordinates

```
C          1.962181       -0.818063        0.065785
C          1.029423        0.160290        0.288614
C         -0.344473       -0.205295        0.715228
O          3.175515       -0.703444       -0.319545
H         -0.401133       -1.251513        1.020228
H         -0.710956        0.416140        1.538988
C         -3.102648       -0.271184        0.271192
H         -3.109420       -1.275338        0.692517
H         -3.216270        0.458093        1.071867
H         -3.936691       -0.171399       -0.419736
S         -1.575236        0.024464       -0.643474
H          1.588436       -1.845949        0.262172
C          1.340174        1.615847        0.087960
H          2.413968        1.753878       -0.039338
H          0.845355        2.043307       -0.791747
H          1.018415        2.219349        0.944321
```

1_methylacrolein_HEI_8

Datum	Value
M06-2X/def2tzvpp-IEFPCM(water) Energy	-669.438669
M06-2X/def2tzvpp-IEFPCM(water) Free Energy (Quasiharmonic)	-669.34
Number of Imaginary Frequencies	0

Frequencies (Top 3 out of 42)

```
1.      60.7119 cm-1
2.      85.7641 cm-1
3.     149.4392 cm-1
```

M06-2X/def2tzvpp-IEFPCM(water) Molecular Geometry in Cartesian Coordinates

```
C          -1.553788       -0.874142        0.000006
C          -1.058044        0.396068       -0.000004
C           0.402268        0.743107       -0.000048
O          -2.781671       -1.258735        0.000019
H           0.660922        1.350963       -0.876380
H           0.661029        1.350892        0.876298
C           3.083992        0.263676        0.000048
H           3.154493        0.887857        0.889331
H           3.905052       -0.449485        0.000023
H           3.154521        0.887945       -0.889172
S           1.542400       -0.671883       -0.000026
H          -0.787050       -1.673338        0.000005
C          -1.965881        1.592882        0.000013
H          -1.809560        2.232935       -0.876888
H          -3.006028        1.269877       -0.000085
H          -1.809697        2.232813        0.877029
```

1_methylacrolein_min

Datum	Value
M06-2X/def2tzvpp-IEFPCM(water) Energy	-231.218956
M06-2X/def2tzvpp-IEFPCM(water) Free Energy (Quasiharmonic)	-231.154806
Number of Imaginary Frequencies	0

Frequencies (Top 3 out of 27)

```
  1.      88.3416 cm-1
  2.     158.9962 cm-1
  3.     288.8251 cm-1
```

M06-2X/def2tzvpp-IEFPCM(water) Molecular Geometry in Cartesian Coordinates

```
C           0.384897        1.454325       -0.000007
H          -0.564562        1.973557       -0.000008
H           1.290019        2.047147       -0.000010
C           0.422966        0.125783       -0.000001
C          -0.861391       -0.630657        0.000003
H          -0.756462       -1.730282        0.000008
O          -1.958598       -0.129084        0.000002
C           1.673998       -0.698832        0.000002
H           1.708908       -1.347060        0.877592
```

```
H           1.708907       -1.347067       -0.877584
H           2.559154       -0.067340       -0.000001
```

1_methylacrolein_TS_1

Datum	Value
M06-2X/def2tzvpp-IEFPCM(water) Energy	-669.429165
M06-2X/def2tzvpp-IEFPCM(water) Free Energy (Quasiharmonic)	-669.332207
Number of Imaginary Frequencies	1

Frequencies (Top 3 out of 42)

```
1.    -160.4792 cm-1
2.      66.9159 cm-1
3.      73.3202 cm-1
```

M06-2X/def2tzvpp-IEFPCM(water) Molecular Geometry in Cartesian Coordinates

```
C          -1.779610       -0.679458       -0.278568
C          -1.142745        0.511158        0.232828
C          -0.335587        0.407860        1.319810
O          -1.681290       -1.814503        0.177600
H           0.066465        1.286246        1.802068
H          -0.289221       -0.520547        1.868464
C           1.539673       -0.429006       -1.267330
H           1.770082       -1.461114       -1.532323
H           0.449732       -0.309333       -1.323453
H           1.980071        0.225185       -2.020187
S           2.111996       -0.015641        0.395356
H          -2.421074       -0.516818       -1.166555
C          -1.277437        1.798046       -0.533586
H          -1.474132        2.641808        0.128626
H          -2.090981        1.745551       -1.258523
H          -0.358320        2.023702       -1.083534
```

1_methylacrolein_TS_2

Datum	Value

Datum	Value
M06-2X/def2tzvpp-IEFPCM(water) Energy	-669.433075
M06-2X/def2tzvpp-IEFPCM(water) Free Energy (Quasiharmonic)	-669.33653
Number of Imaginary Frequencies	1

Frequencies (Top 3 out of 42)

```
1.    -186.9039 cm-1
2.      28.5678 cm-1
3.      74.8581 cm-1
```

M06-2X/def2tzvpp-IEFPCM(water) Molecular Geometry in Cartesian Coordinates

```
C          -1.666722        0.586483        0.622399
C          -0.968121       -0.583774        0.202601
C           0.074482       -1.030555        0.973662
O          -2.617990        1.115221        0.040742
H           0.246239       -0.581410        1.942369
H           0.474728       -2.023844        0.837044
C           1.527322        1.340978       -0.588757
H           0.433621        1.277441       -0.522964
H           1.845892        2.224661       -0.036460
H           1.787185        1.479118       -1.637941
S           2.244000       -0.173924        0.080155
H          -1.308684        1.021860        1.575472
C          -1.329073       -1.215272       -1.109084
H          -2.409959       -1.312964       -1.218519
H          -0.978072       -0.608100       -1.949666
H          -0.878347       -2.202912       -1.202683
```

1_methylacrolein_TS_3_reopt

Datum	Value
M06-2X/def2tzvpp-IEFPCM(water) Energy	-669.423398
M06-2X/def2tzvpp-IEFPCM(water) Free Energy (Quasiharmonic)	-669.327402
Number of Imaginary Frequencies	1

Frequencies (Top 3 out of 42)

```
1.    -226.3737 cm-1
2.      25.6547 cm-1
3.      72.8376 cm-1
```

M06-2X/def2tzvpp-IEFPCM(water) Molecular Geometry in Cartesian Coordinates

```
C          2.252621      -0.229025      -0.263867
C          1.144823       0.535661       0.230660
C          0.214566      -0.085750       1.016507
O          2.442710      -1.436200      -0.112445
H          0.424853      -1.074591       1.396367
H         -0.527032       0.492131       1.551289
C         -2.634899       0.641892      -0.015815
H         -2.939843       0.753491       1.025600
H         -1.981917       1.479479      -0.264946
H         -3.528654       0.713975      -0.634842
S         -1.782338      -0.944314      -0.285101
H          3.002610       0.355232      -0.832502
C          0.977870       1.952918      -0.244533
H          0.408167       2.002628      -1.177626
H          1.943291       2.426543      -0.431737
H          0.444367       2.555558       0.491866
```

1_methylacrolein_TS_4_reopt

Datum	Value
M06-2X/def2tzvpp-IEFPCM(water) Energy	-669.433075
M06-2X/def2tzvpp-IEFPCM(water) Free Energy (Quasiharmonic)	-669.336534
Number of Imaginary Frequencies	1

Frequencies (Top 3 out of 42)

```
1.    -186.9448 cm-1
2.      28.3226 cm-1
3.      75.0938 cm-1
```

M06-2X/def2tzvpp-IEFPCM(water) Molecular Geometry in Cartesian Coordinates

```
C        -1.666885     0.586019    -0.622749
C        -0.968056    -0.583903    -0.202359
C         0.074433    -1.031019    -0.973371
O        -2.618121     1.114993    -0.041272
H         0.474855    -2.024162    -0.836219
H         0.245975    -0.582407    -1.942364
C         1.527027     1.340852     0.589030
H         0.433348     1.277207     0.522971
H         1.786661     1.478477     1.638340
H         1.845590     2.224862     0.037252
S         2.244037    -0.173587    -0.080578
H        -1.309049     1.020857    -1.576145
C        -1.328593    -1.214619     1.109812
H        -2.409479    -1.311364     1.220034
H        -0.878604    -2.202590     1.203481
H        -0.976471    -0.607417     1.949909
```

1_methylacrolein_TS_5

Datum	Value
M06-2X/def2tzvpp-IEFPCM(water) Energy	-669.423712
M06-2X/def2tzvpp-IEFPCM(water) Free Energy (Quasiharmonic)	-669.328152
Number of Imaginary Frequencies	1

Frequencies (Top 3 out of 42)

```
1.    -202.4437 cm-1
2.      27.4233 cm-1
3.      58.9429 cm-1
```

M06-2X/def2tzvpp-IEFPCM(water) Molecular Geometry in Cartesian Coordinates

```
C        -2.127813    -0.759451    -0.155647
C        -1.372688     0.383551     0.268626
C        -0.135735     0.190073     0.816528
O        -1.767428    -1.936111    -0.119244
H         0.389147     1.006887     1.292498
H         0.169856    -0.808242     1.095521
C         2.895308    -0.287622     0.620369
H         2.521287     0.155655     1.546932
H         2.924200    -1.368780     0.757392
H         3.916794     0.062267     0.475798
```

```
S           1.834191       0.180170      -0.778878
H          -3.138117      -0.529346      -0.547240
C          -1.902793       1.759166      -0.028663
H          -1.677220       2.458483       0.777190
H          -2.985359       1.745131      -0.164623
H          -1.465890       2.169807      -0.944750
```

1_methylacrolein_TS_6_reopt

Datum	Value
M06-2X/def2tzvpp-IEFPCM(water) Energy	-669.433075
M06-2X/def2tzvpp-IEFPCM(water) Free Energy (Quasiharmonic)	-669.336531
Number of Imaginary Frequencies	1

Frequencies (Top 3 out of 42)

```
1.    -186.9112 cm-1
2.      28.5351 cm-1
3.      74.8778 cm-1
```

M06-2X/def2tzvpp-IEFPCM(water) Molecular Geometry in Cartesian Coordinates

```
C          -1.666826       0.586302      -0.622487
C          -0.968104      -0.583846      -0.202533
C           0.074418      -1.030701      -0.973643
O          -2.618052       1.115117      -0.040849
H           0.474774      -2.023926      -0.836885
H           0.246091      -0.581672      -1.942421
C           1.527154       1.340975       0.588777
H           0.433469       1.277398       0.522795
H           1.786837       1.478961       1.638027
H           1.845772       2.224762       0.036673
S           2.244013      -0.173786      -0.080269
H          -1.308912       1.021490      -1.575693
C          -1.328759      -1.215092       1.109350
H          -2.409665      -1.311624       1.219550
H          -0.878999      -2.203213       1.202546
H          -0.976467      -0.608369       1.949723
```

1_methylacrolein_TS_7_reopt

Datum	Value
M06-2X/def2tzvpp-IEFPCM(water) Energy	-669.427905
M06-2X/def2tzvpp-IEFPCM(water) Free Energy (Quasiharmonic)	-669.332021
Number of Imaginary Frequencies	1

Frequencies (Top 3 out of 42)

```
1.    -223.8011 cm-1
2.      48.7662 cm-1
3.      64.3882 cm-1
```

M06-2X/def2tzvpp-IEFPCM(water) Molecular Geometry in Cartesian Coordinates

```
C          1.990916       -0.894101        0.126105
C          1.179836        0.248374        0.358277
C         -0.091800        0.051568        0.847503
O          3.131717       -0.897468       -0.349092
H         -0.369175       -0.929409        1.211722
H         -0.648300        0.877598        1.268462
C         -3.153438       -0.227880        0.508109
H         -2.750504        0.115789        1.465339
H         -4.042451        0.363620        0.293376
H         -3.456958       -1.267260        0.629419
S         -1.901265       -0.041172       -0.793804
H          1.530770       -1.857160        0.421305
C          1.683063        1.600396       -0.054859
H          2.704372        1.767234        0.291019
H          1.698255        1.706942       -1.144105
H          1.049030        2.390999        0.346249
```

1_methylacrolein_TS_8_reopt

Datum	Value
M06-2X/def2tzvpp-IEFPCM(water) Energy	-669.427926
M06-2X/def2tzvpp-IEFPCM(water) Free Energy (Quasiharmonic)	-669.331687
Number of Imaginary Frequencies	1

Frequencies (Top 3 out of 42)

```
1.    -223.6741 cm-1
2.      47.6865 cm-1
3.      63.0025 cm-1
```

M06-2X/def2tzvpp-IEFPCM(water) Molecular Geometry in Cartesian Coordinates

```
C          2.016968       -0.852783        0.207117
C          1.171804        0.282539        0.332755
C         -0.093846        0.094305        0.838226
O          3.158522       -0.866983       -0.265214
H         -0.345896       -0.856214        1.291119
H         -0.677926        0.939016        1.176573
C         -3.138727       -0.032106        0.530234
H         -3.423566        1.012537        0.655673
H         -4.039509       -0.606065        0.317367
H         -2.736312       -0.386606        1.482907
S         -1.902846       -0.237735       -0.785168
H          1.584252       -1.797243        0.590533
C          1.634424        1.604833       -0.204276
H          0.994945        2.413966        0.148313
H          2.661122        1.818659        0.096333
H          1.616509        1.620855       -1.298753
```

2_crotonaldehyde_conf2_min

Datum	Value
M06-2X/def2tzvpp-IEFPCM(water) Energy	-231.22527
M06-2X/def2tzvpp-IEFPCM(water) Free Energy (Quasiharmonic)	-231.160531
Number of Imaginary Frequencies	0

Frequencies (Top 3 out of 27)

```
1.     126.4104 cm-1
2.     201.8334 cm-1
3.     215.7423 cm-1
```

M06-2X/def2tzvpp-IEFPCM(water) Molecular Geometry in Cartesian Coordinates

```
C          1.031183       0.388324      -0.000000
H          0.937836       1.471638       0.000004
C         -0.089004      -0.335612      -0.000006
H         -0.077761      -1.419603      -0.000009
C         -1.388835       0.330420      -0.000003
H         -1.349933       1.434379      -0.000007
O         -2.454642      -0.244905       0.000005
C          2.412755      -0.161467       0.000002
H          2.958722       0.196017      -0.874892
H          2.958698       0.195973       0.874929
H          2.412987      -1.249155      -0.000024
```

2_crotonaldehyde_HEI-1

Datum	Value
M06-2X/def2tzvpp-IEFPCM(water) Energy	-669.447041
M06-2X/def2tzvpp-IEFPCM(water) Free Energy (Quasiharmonic)	-669.347856
Number of Imaginary Frequencies	0

Frequencies (Top 3 out of 42)

```
  1.      41.3776 cm-1
  2.      76.1837 cm-1
  3.     160.6684 cm-1
```

M06-2X/def2tzvpp-IEFPCM(water) Molecular Geometry in Cartesian Coordinates

```
C         -2.090259       0.058779       0.390104
C         -0.892238       0.648011       0.700559
C          0.236079       0.750750      -0.255128
O         -2.455476      -0.488250      -0.703059
H         -0.753258       1.053837       1.697348
H         -0.122801       0.509511      -1.256328
C          0.672105      -2.011146       0.001769
H         -0.228935      -1.908131       0.605208
H          0.397324      -2.253790      -1.023247
H          1.298512      -2.803968       0.404559
S          1.601811      -0.472235       0.077744
H         -2.831776       0.081216       1.215152
C          0.912631       2.117565      -0.256241
H          1.264872       2.366161       0.746881
```

```
H          1.765137       2.151868       -0.936563
H          0.195845       2.881304       -0.558814
```

2_crotonaldehyde_HEI-2

Datum	Value
M06-2X/def2tzvpp-IEFPCM(water) Energy	-669.439061
M06-2X/def2tzvpp-IEFPCM(water) Free Energy (Quasiharmonic)	-669.340034
Number of Imaginary Frequencies	0

Frequencies (Top 3 out of 42)

```
  1.      53.0826 cm-1
  2.      98.2506 cm-1
  3.     152.5675 cm-1
```

M06-2X/def2tzvpp-IEFPCM(water) Molecular Geometry in Cartesian Coordinates

```
C          1.765393      -0.257856      -0.266316
C          1.021599       0.517816       0.574869
C         -0.450360       0.812291       0.482545
O          3.028781      -0.474621      -0.243485
H          1.533541       1.027615       1.389353
H         -0.883315       0.797328       1.488030
C         -1.121975      -1.907521       0.444332
H         -0.091787      -2.228664       0.310611
H         -1.320843      -1.755163       1.504208
H         -1.797126      -2.669955       0.061648
S         -1.445114      -0.391823      -0.471034
H          1.185791      -0.767503      -1.060483
C         -0.759173       2.182088      -0.129621
H         -1.825952       2.413884      -0.098441
H         -0.420854       2.213975      -1.166906
H         -0.220777       2.953719       0.421545
```

2_crotonaldehyde_HEI-3

Datum	Value

Datum	Value
M06-2X/def2tzvpp-IEFPCM(water) Energy	-669.443351
M06-2X/def2tzvpp-IEFPCM(water) Free Energy (Quasiharmonic)	-669.344361
Number of Imaginary Frequencies	0

Frequencies (Top 3 out of 42)

```
1.      40.4011 cm-1
2.      71.5523 cm-1
3.     168.9786 cm-1
```

M06-2X/def2tzvpp-IEFPCM(water) Molecular Geometry in Cartesian Coordinates

```
C          -2.183815       -0.133830       -0.290819
C          -1.022022        0.179187        0.363074
C           0.158007        0.755642       -0.329338
O          -3.264728       -0.623308        0.173558
H          -0.948705        0.007317        1.433961
H          -0.133537        1.079784       -1.331124
C           1.775477       -1.220644        0.881305
H           2.170601       -0.493627        1.588379
H           0.847424       -1.639978        1.266017
H           2.503542       -2.018908        0.755892
S           1.497659       -0.485618       -0.739588
H          -2.157390        0.087487       -1.381189
C           0.784587        1.929535        0.419192
H           1.639989        2.342270       -0.115038
H           1.118421        1.616058        1.410081
H           0.041540        2.716600        0.557477
```

2_crotonaldehyde_HEI-4

Datum	Value
M06-2X/def2tzvpp-IEFPCM(water) Energy	-669.442746
M06-2X/def2tzvpp-IEFPCM(water) Free Energy (Quasiharmonic)	-669.343168
Number of Imaginary Frequencies	0

Frequencies (Top 3 out of 42)

```
1.      62.2852 cm-1
2.      86.6997 cm-1
3.     155.7593 cm-1
```

M06-2X/def2tzvpp-IEFPCM(water) Molecular Geometry in Cartesian Coordinates

```
C         -1.993369      -0.092694       0.173908
C         -1.002727       0.133431      -0.743480
C          0.269698       0.860144      -0.487598
O         -3.090530      -0.729015       0.012647
H         -1.120607      -0.291997      -1.736947
H          0.509820       1.513435      -1.330642
C          1.404346      -1.330234       0.873840
H          1.680093      -0.858000       1.814253
H          0.337109      -1.557612       0.873194
H          1.968178      -2.252977       0.759994
S          1.762202      -0.272484      -0.538667
H         -1.818751       0.345119       1.175773
C          0.319469       1.692668       0.788282
H          1.259616       2.239122       0.848546
H          0.234427       1.068197       1.677345
H         -0.505386       2.406692       0.806277
```

2_crotonaldehyde_HEI-5

Datum	Value
M06-2X/def2tzvpp-IEFPCM(water) Energy	-669.445342
M06-2X/def2tzvpp-IEFPCM(water) Free Energy (Quasiharmonic)	-669.346054
Number of Imaginary Frequencies	0

Frequencies (Top 3 out of 42)

```
1.      42.7298 cm-1
2.      74.1069 cm-1
3.     160.8299 cm-1
```

M06-2X/def2tzvpp-IEFPCM(water) Molecular Geometry in Cartesian Coordinates

```
C        -2.171940     -0.225185      0.526842
C        -0.974558      0.404191      0.745732
C        -0.008566      0.695075     -0.343518
O        -2.645237     -0.667082     -0.571588
H        -0.719316      0.704813      1.755590
H        -0.546301      0.752362     -1.291785
C         1.925868     -0.927865      0.915558
H         2.558529     -0.084618      1.185968
H         1.146923     -1.059440      1.665364
H         2.537187     -1.826474      0.873112
S         1.173149     -0.704982     -0.705829
H        -2.797887     -0.348054      1.434322
C         0.778866      1.979684     -0.114372
H         1.342660      1.922885      0.818760
H         1.479273      2.175395     -0.925839
H         0.092428      2.824093     -0.030985
```

2_crotonaldehyde_HEI-6

Datum	Value
M06-2X/def2tzvpp-IEFPCM(water) Energy	-669.446501
M06-2X/def2tzvpp-IEFPCM(water) Free Energy (Quasiharmonic)	-669.347102
Number of Imaginary Frequencies	0

Frequencies (Top 3 out of 42)

```
1.      61.4940 cm-1
2.      74.0728 cm-1
3.     157.8426 cm-1
```

M06-2X/def2tzvpp-IEFPCM(water) Molecular Geometry in Cartesian Coordinates

```
C         2.394361     -0.166161     -0.187353
C         1.313484      0.605146     -0.526904
C         0.022249      0.554474      0.212180
O         2.495779     -1.020888      0.752971
H         1.392822      1.280258     -1.371718
H         0.196898      0.149488      1.211077
C        -2.513248     -0.719141      0.482288
H        -2.188154     -0.827045      1.516625
H        -3.124688      0.175048      0.384435
H        -3.112469     -1.586064      0.211551
```

```
S          -1.076881       -0.680796       -0.615932
H           3.289699       -0.000703       -0.820647
C          -0.666594        1.908485        0.306127
H          -0.838863        2.313224       -0.692794
H          -1.624569        1.852426        0.823355
H          -0.028327        2.606399        0.851227
```

2_crotonaldehyde_HEI-7

Datum	Value
M06-2X/def2tzvpp-IEFPCM(water) Energy	-669.445
M06-2X/def2tzvpp-IEFPCM(water) Free Energy (Quasiharmonic)	-669.345987
Number of Imaginary Frequencies	0

Frequencies (Top 3 out of 42)

```
1.      66.3520 cm-1
2.      77.1418 cm-1
3.     158.6728 cm-1
```

M06-2X/def2tzvpp-IEFPCM(water) Molecular Geometry in Cartesian Coordinates

```
C           2.289980       -0.253060        0.371236
C           1.258574        0.299781       -0.339834
C          -0.097661        0.502814        0.238505
O           3.486044       -0.491564        0.004462
H           1.409569        0.588152       -1.377437
H          -0.058463        0.365086        1.322029
C          -2.787173       -0.471063        0.400638
H          -2.642182       -0.274620        1.462482
H          -3.278398        0.380898       -0.063760
H          -3.423299       -1.347040        0.291324
S          -1.201089       -0.849157       -0.382484
H           2.020470       -0.514385        1.418838
C          -0.696003        1.866863       -0.082173
H          -1.698876        1.990436        0.326857
H          -0.745704        2.009625       -1.163125
H          -0.060350        2.648848        0.336601
```

2_crotonaldehyde_HEI-8

Datum	Value
M06-2X/def2tzvpp-IEFPCM(water) Energy	-669.442516
M06-2X/def2tzvpp-IEFPCM(water) Free Energy (Quasiharmonic)	-669.342601
Number of Imaginary Frequencies	0

Frequencies (Top 3 out of 42)

```
1.      78.4562 cm-1
2.      91.4444 cm-1
3.     179.3331 cm-1
```

M06-2X/def2tzvpp-IEFPCM(water) Molecular Geometry in Cartesian Coordinates

```
C          -1.944143       -0.408179       -0.529983
C          -0.922407        0.328764       -1.073463
C           0.195260        0.995486       -0.344503
O          -2.198091       -0.706656        0.683348
H          -0.898210        0.397118       -2.155403
H           0.540079        1.852672       -0.927569
C           1.146566       -1.511297        0.504344
H           1.014858       -1.353312        1.572760
H           0.193085       -1.789240        0.057283
H           1.875517       -2.303211        0.346975
S           1.756246       -0.027149       -0.315251
H          -2.652996       -0.788693       -1.296675
C          -0.129194        1.475270        1.066646
H          -0.338334        0.634563        1.722575
H           0.698372        2.057192        1.473021
H          -1.024071        2.100282        1.046005
```

2_crotonaldehyde_min

Datum	Value
M06-2X/def2tzvpp-IEFPCM(water) Energy	-231.221346
M06-2X/def2tzvpp-IEFPCM(water) Free Energy (Quasiharmonic)	-231.156905
Number of Imaginary Frequencies	0

Frequencies (Top 3 out of 27)

```
1.     127.4799 cm-1
2.     207.2556 cm-1
3.     210.2038 cm-1
```

M06-2X/def2tzvpp-IEFPCM(water) Molecular Geometry in Cartesian Coordinates

```
C          0.833395       -0.300960        0.000005
H          0.459425       -1.320761        0.000064
C         -0.062330        0.686072       -0.000036
H          0.243512        1.725503       -0.000095
C         -1.509582        0.414698       -0.000000
H         -2.159499        1.305108       -0.000036
O         -2.006005       -0.690078        0.000064
C          2.309102       -0.118985       -0.000026
H          2.747323       -0.603514        0.874603
H          2.747296       -0.603598       -0.874621
H          2.586470        0.932936       -0.000081
```

2_crotonaldehyde_TS-1-0

Datum	Value
M06-2X/def2tzvpp-IEFPCM(water) Energy	-669.430976
M06-2X/def2tzvpp-IEFPCM(water) Free Energy (Quasiharmonic)	-669.333262
Number of Imaginary Frequencies	1

Frequencies (Top 3 out of 42)

```
1.    -164.5178 cm-1
2.      72.4989 cm-1
3.      85.5952 cm-1
```

M06-2X/def2tzvpp-IEFPCM(water) Molecular Geometry in Cartesian Coordinates

```
C         -2.041327       -0.543778        0.258384
C         -1.188109        0.536885        0.652697
C         -0.338169        1.141408       -0.227801
O         -2.123236       -1.036449       -0.866288
H         -1.199129        0.824279        1.697810
```

```
H          -0.450576        0.886296       -1.272449
C           1.140758       -1.742975        0.580163
H           0.180565       -1.449047        1.023791
H           0.961387       -2.609209       -0.057334
H           1.799072       -2.047466        1.393667
S           1.820341       -0.345946       -0.341511
H          -2.679744       -0.954074        1.062669
C           0.367456        2.423800        0.081437
H           0.651045        2.472875        1.132125
H           1.258651        2.548773       -0.528766
H          -0.304497        3.262257       -0.126307
```

2_crotonaldehyde_TS-2-1_reopt

Datum	Value
M06-2X/def2tzvpp-IEFPCM(water) Energy	-669.428939
M06-2X/def2tzvpp-IEFPCM(water) Free Energy (Quasiharmonic)	-669.330865
Number of Imaginary Frequencies	1

Frequencies (Top 3 out of 42)

```
1.    -197.1535 cm-1
2.      64.4987 cm-1
3.      91.3577 cm-1
```

M06-2X/def2tzvpp-IEFPCM(water) Molecular Geometry in Cartesian Coordinates

```
C          -2.004658       -0.225343        0.182474
C          -1.127216        0.319156       -0.793124
C          -0.060881        1.135563       -0.492229
O          -2.975071       -0.956437       -0.045429
H          -1.307723        0.036803       -1.824450
H           0.405180        1.658087       -1.315076
C           1.317780       -1.405252        0.738126
H           0.223793       -1.343074        0.671748
H           1.598239       -2.438067        0.534562
H           1.610215       -1.168995        1.761417
S           2.051365       -0.269555       -0.459212
H          -1.799569        0.061146        1.229105
C           0.150331        1.748064        0.866344
H           1.079334        2.309987        0.891878
H           0.192597        0.990982        1.648411
H          -0.675479        2.424373        1.103692
```

2_crotonaldehyde_TS-3-1

Datum	Value
M06-2X/def2tzvpp-IEFPCM(water) Energy	-669.432504
M06-2X/def2tzvpp-IEFPCM(water) Free Energy (Quasiharmonic)	-669.335085
Number of Imaginary Frequencies	1

Frequencies (Top 3 out of 42)

```
  1.    -183.7478 cm-1
  2.      47.6205 cm-1
  3.      88.0625 cm-1
```

M06-2X/def2tzvpp-IEFPCM(water) Molecular Geometry in Cartesian Coordinates

```
C          2.025929      -0.222633       0.322221
C          1.102338       0.651269      -0.315544
C         -0.008035       1.107314       0.348423
O          3.071038      -0.668685      -0.155393
H          1.289700       0.935544      -1.345990
H         -0.077872       0.876989       1.404549
C         -0.924515      -1.988573      -0.121017
H         -1.244815      -2.619976      -0.949462
H          0.112390      -1.690132      -0.315834
H         -0.938716      -2.587955       0.789302
S         -1.945054      -0.504805       0.018098
H          1.752977      -0.494863       1.361371
C         -0.774008       2.308209      -0.116104
H         -1.771939       2.341073       0.314930
H         -0.859452       2.318991      -1.201891
H         -0.239963       3.213163       0.188733
```

2_crotonaldehyde_TS-4-0

Datum	Value
M06-2X/def2tzvpp-IEFPCM(water) Energy	-669.428939
M06-2X/def2tzvpp-IEFPCM(water) Free Energy (Quasiharmonic)	-669.330865

Datum	Value
Number of Imaginary Frequencies	1

Frequencies (Top 3 out of 42)

```
1.    -197.1970 cm-1
2.      64.4652 cm-1
3.      91.3721 cm-1
```

M06-2X/def2tzvpp-IEFPCM(water) Molecular Geometry in Cartesian Coordinates

```
C          -2.004616       -0.225299        0.182401
C          -1.127123        0.319075       -0.793197
C          -0.060777        1.135506       -0.492346
O          -2.975049       -0.956399       -0.045462
H          -1.307589        0.036629       -1.824505
H           0.405238        1.657958       -1.315262
C           1.317773       -1.405203        0.738348
H           1.610508       -1.169044        1.761573
H           0.223778       -1.342825        0.672239
H           1.598000       -2.438056        0.534660
S           2.051252       -0.269590       -0.459125
H          -1.799576        0.061303        1.229011
C           0.150342        1.748111        0.866200
H           1.079359        2.310005        0.891772
H           0.192528        0.991069        1.648313
H          -0.675468        2.424457        1.103446
```

2_crotonaldehyde_TS-5-0

Datum	Value
M06-2X/def2tzvpp-IEFPCM(water) Energy	-669.430976
M06-2X/def2tzvpp-IEFPCM(water) Free Energy (Quasiharmonic)	-669.333263
Number of Imaginary Frequencies	1

Frequencies (Top 3 out of 42)

```
1.    -164.3790 cm-1
2.      72.4706 cm-1
3.      85.5731 cm-1
```

M06-2X/def2tzvpp-IEFPCM(water) Molecular Geometry in Cartesian Coordinates

```
C          -2.041107       -0.544644        0.258355
C          -1.188432        0.536460        0.652745
C          -0.338882        1.141403       -0.227766
O          -2.122708       -1.037238       -0.866350
H          -1.199601        0.823825        1.697864
H          -0.451069        0.886082       -1.272387
C           1.141489       -1.742593        0.580133
H           1.799982       -2.047051        1.393506
H           0.181286       -1.448987        1.023938
H           0.962252       -2.608793       -0.057452
S           1.820682       -0.345293       -0.341462
H          -2.679304       -0.955360        1.062597
C           0.366263        2.424046        0.081407
H           0.649431        2.473468        1.132194
H           1.257644        2.549119       -0.528499
H          -0.305853        3.262259       -0.126813
```

2_crotonaldehyde_TS-6-1

Datum	Value
M06-2X/def2tzvpp-IEFPCM(water) Energy	-669.426361
M06-2X/def2tzvpp-IEFPCM(water) Free Energy (Quasiharmonic)	-669.329209
Number of Imaginary Frequencies	1

Frequencies (Top 3 out of 42)

```
  1.    -221.2025 cm-1
  2.      61.6153 cm-1
  3.      88.3603 cm-1
```

M06-2X/def2tzvpp-IEFPCM(water) Molecular Geometry in Cartesian Coordinates

```
C           2.484692       -0.375707       -0.130261
C           1.574201        0.688233       -0.393812
C           0.366532        0.794523        0.249806
O           2.313521       -1.314873        0.650780
```

```
H          1.847405        1.400536       -1.163329
H          0.210112        0.161533        1.114624
C         -2.519625       -0.544656        0.700370
H         -1.956525       -0.174148        1.562610
H         -3.317074        0.169768        0.494593
H         -2.978472       -1.489252        0.990976
S         -1.413952       -0.754075       -0.726200
H          3.434097       -0.330392       -0.695646
C         -0.442590        2.052532        0.176058
H         -0.419341        2.470987       -0.829558
H         -1.476779        1.893568        0.469924
H         -0.007613        2.792033        0.855808
```

2_crotonaldehyde_TS-7-0

Datum	Value
M06-2X/def2tzvpp-IEFPCM(water) Energy	-669.427889
M06-2X/def2tzvpp-IEFPCM(water) Free Energy (Quasiharmonic)	-669.330708
Number of Imaginary Frequencies	1

Frequencies (Top 3 out of 42)

```
1.    -231.3141 cm-1
2.      58.7215 cm-1
3.      84.2343 cm-1
```

M06-2X/def2tzvpp-IEFPCM(water) Molecular Geometry in Cartesian Coordinates

```
C          2.311382       -0.431483        0.368312
C          1.457324        0.521032       -0.237834
C          0.191318        0.747292        0.259643
O          3.455371       -0.735925        0.017068
H          1.797248        1.029507       -1.133824
H         -0.039906        0.327393        1.232849
C         -2.767705       -0.440908        0.629090
H         -2.318232        0.040674        1.502855
H         -3.480318        0.259619        0.193091
H         -3.320446       -1.310792        0.982368
S         -1.473408       -0.915830       -0.553753
H          1.875530       -0.934892        1.254896
C         -0.577209        1.975214       -0.127140
H         -1.631163        1.899609        0.126603
```

```
H          -0.482506       2.168459       -1.194873
H          -0.159305       2.834221        0.407116
```

2_crotonaldehyde_TS-8-0

Datum	Value
M06-2X/def2tzvpp-IEFPCM(water) Energy	-669.426801
M06-2X/def2tzvpp-IEFPCM(water) Free Energy (Quasiharmonic)	-669.32931
Number of Imaginary Frequencies	1

Frequencies (Top 3 out of 42)

```
  1.    -164.8974 cm-1
  2.      33.7760 cm-1
  3.      68.1241 cm-1
```

M06-2X/def2tzvpp-IEFPCM(water) Molecular Geometry in Cartesian Coordinates

```
C          -1.851664      -0.974747      -0.193688
C          -1.016642      -0.200989      -1.058995
C          -0.383925       0.971942      -0.735455
O          -2.163129      -0.733047       0.973127
H          -0.822456      -0.644212      -2.028258
H           0.145227       1.465105      -1.539412
C           1.704041      -1.464870       0.135839
H           1.757681      -1.895387       1.135744
H           0.701093      -1.669579      -0.261656
H           2.424768      -1.982315      -0.497319
S           1.979558       0.320527       0.158185
H          -2.248597      -1.897088      -0.660114
C          -0.745929       1.853330       0.422190
H          -0.848981       1.287024       1.341278
H          -0.001305       2.634436       0.557136
H          -1.710614       2.329954       0.217272
```

3_4-methyl-2-pentenal_1

Datum	Value

Datum	Value
M06-2X/def2tzvpp-IEFPCM(water) Energy	-309.842254
M06-2X/def2tzvpp-IEFPCM(water) Free Energy (Quasiharmonic)	-309.723854
Number of Imaginary Frequencies	0

Frequencies (Top 3 out of 45)

```
1.      77.0282 cm-1
2.     150.5143 cm-1
3.     153.7262 cm-1
```

M06-2X/def2tzvpp-IEFPCM(water) Molecular Geometry in Cartesian Coordinates

```
C          2.289949       -0.359574       -0.058781
C          0.956989        0.238206       -0.017555
C         -0.100594       -0.478274       -0.402997
C         -1.530482       -0.041565       -0.406826
C         -2.352749       -1.004162        0.458880
O          3.306627        0.209048        0.272222
C         -1.731375        1.401999        0.030064
H          2.326229       -1.402002       -0.422480
H          0.881571        1.260677        0.331574
H          0.074726       -1.499360       -0.737199
H         -1.881877       -0.151853       -1.438741
H         -3.410907       -0.751846        0.398378
H         -2.226264       -2.037124        0.135264
H         -2.041898       -0.931186        1.502257
H         -2.786740        1.665976       -0.027245
H         -1.406055        1.540235        1.063022
H         -1.172241        2.094319       -0.599316
```

3_4-methyl-2-pentenal_2

Datum	Value
M06-2X/def2tzvpp-IEFPCM(water) Energy	-309.841991
M06-2X/def2tzvpp-IEFPCM(water) Free Energy (Quasiharmonic)	-309.724126
Number of Imaginary Frequencies	0

Frequencies (Top 3 out of 45)

```
1.      55.6678 cm-1
2.     144.8724 cm-1
3.     153.5429 cm-1
```

M06-2X/def2tzvpp-IEFPCM(water) Molecular Geometry in Cartesian Coordinates

```
C          -1.444070       -1.873389        0.000000
C          -0.185809       -1.130521        0.000000
C          -0.197408        0.202975       -0.000000
C           1.002775        1.090996       -0.000000
C           1.002775        1.963360        1.258037
O          -1.527196       -3.081552        0.000000
C           1.002775        1.963360       -1.258037
H          -2.356094       -1.250250        0.000000
H           0.736229       -1.700756        0.000000
H          -1.161407        0.711773       -0.000000
H           1.897473        0.465226       -0.000000
H           1.873570        2.618953        1.258648
H           0.107866        2.587915        1.287333
H           1.026132        1.355999        2.162160
H           1.873570        2.618953       -1.258648
H           1.026132        1.355999       -2.162160
H           0.107866        2.587915       -1.287333
```

3_4-methyl-2-pentenal_3_2

Datum	Value
M06-2X/def2tzvpp-IEFPCM(water) Energy	-309.838276
M06-2X/def2tzvpp-IEFPCM(water) Free Energy (Quasiharmonic)	-309.720113
Number of Imaginary Frequencies	0

Frequencies (Top 3 out of 45)

```
1.      71.4940 cm-1
2.     119.9612 cm-1
3.     170.1273 cm-1
```

M06-2X/def2tzvpp-IEFPCM(water) Molecular Geometry in Cartesian Coordinates

```
C          2.370651        0.368677        0.211938
C          0.925911        0.648440        0.135694
C          0.065838       -0.266758       -0.312251
C         -1.418363       -0.123808       -0.422682
C         -2.093320       -1.235040        0.391195
O          2.892982       -0.673097       -0.117128
C         -1.938386        1.247937       -0.018953
H          2.992377        1.191238        0.602121
H          0.604590        1.629659        0.460483
H          0.472411       -1.228514       -0.616147
H         -1.664946       -0.299324       -1.475982
H         -3.173357       -1.199640        0.251552
H         -1.739331       -2.220376        0.088576
H         -1.880728       -1.105947        1.453661
H         -3.018400        1.292977       -0.154764
H         -1.723607        1.446969        1.032855
H         -1.486847        2.041052       -0.614974
```

3_4-methyl-2-pentenal_4

Datum	Value
M06-2X/def2tzvpp-IEFPCM(water) Energy	-309.838039
M06-2X/def2tzvpp-IEFPCM(water) Free Energy (Quasiharmonic)	-309.720504
Number of Imaginary Frequencies	0

Frequencies (Top 3 out of 45)

```
1.      57.1896 cm-1
2.      95.2675 cm-1
3.     148.8433 cm-1
```

M06-2X/def2tzvpp-IEFPCM(water) Molecular Geometry in Cartesian Coordinates

```
C          2.469499        0.000001        0.307252
C          1.076854        0.000001        0.787421
C          0.044448       -0.000000       -0.055195
C         -1.397191        0.000000        0.332806
C         -2.081029       -1.257691       -0.210126
O          2.798763       -0.000002       -0.858126
C         -2.081029        1.257690       -0.210129
H          3.242446        0.000003        1.093437
H          0.927234        0.000003        1.860567
```

```
H          0.254711      -0.000002      -1.123517
H         -1.465204       0.000001       1.422723
H         -3.136314      -1.258197       0.063096
H         -2.012447      -1.286811      -1.299268
H         -1.620543      -2.162125       0.185992
H         -3.136314       1.258197       0.063093
H         -1.620543       2.162126       0.185988
H         -2.012446       1.286809      -1.299270
```

3_4-methyl-2-pentenal_5

Datum	Value
M06-2X/def2tzvpp-IEFPCM(water) Energy	-309.842254
M06-2X/def2tzvpp-IEFPCM(water) Free Energy (Quasiharmonic)	-309.723854
Number of Imaginary Frequencies	0

Frequencies (Top 3 out of 45)

```
  1.      77.0283 cm-1
  2.     150.5142 cm-1
  3.     153.7262 cm-1
```

M06-2X/def2tzvpp-IEFPCM(water) Molecular Geometry in Cartesian Coordinates

```
C         -2.289949      -0.359574      -0.058780
C         -0.956990       0.238206      -0.017557
C          0.100594      -0.478275      -0.402996
C          1.530481      -0.041565      -0.406826
C          1.731374       1.401999       0.030065
O         -3.306628       0.209048       0.272222
C          2.352750      -1.004162       0.458879
H         -2.326229      -1.402004      -0.422476
H         -0.881573       1.260678       0.331569
H         -0.074726      -1.499362      -0.737195
H          1.881876      -0.151852      -1.438741
H          2.786740       1.665976      -0.027243
H          1.172241       2.094319      -0.599315
H          1.406054       1.540235       1.063023
H          3.410908      -0.751845       0.398376
H          2.041900      -0.931187       1.502256
H          2.226265      -2.037123       0.135262
```

3_4-methyl-2-pentenal_6_2

Datum	Value
M06-2X/def2tzvpp-IEFPCM(water) Energy	-309.838276
M06-2X/def2tzvpp-IEFPCM(water) Free Energy (Quasiharmonic)	-309.720113
Number of Imaginary Frequencies	0

Frequencies (Top 3 out of 45)

```
1.      71.4940 cm-1
2.     119.9612 cm-1
3.     170.1273 cm-1
```

M06-2X/def2tzvpp-IEFPCM(water) Molecular Geometry in Cartesian Coordinates

```
C          2.370651        0.368677        0.211938
C          0.925911        0.648440        0.135694
C          0.065838       -0.266758       -0.312251
C         -1.418363       -0.123808       -0.422682
C         -2.093320       -1.235040        0.391195
O          2.892982       -0.673097       -0.117128
C         -1.938386        1.247937       -0.018953
H          2.992377        1.191238        0.602121
H          0.604590        1.629659        0.460483
H          0.472411       -1.228514       -0.616147
H         -1.664946       -0.299324       -1.475982
H         -3.173357       -1.199640        0.251552
H         -1.739331       -2.220376        0.088575
H         -1.880728       -1.105947        1.453661
H         -3.018400        1.292977       -0.154764
H         -1.723607        1.446969        1.032855
H         -1.486847        2.041052       -0.614974
```

3_4methyl2pentenal_HEI_10_reopt

Datum	Value
M06-2X/def2tzvpp-IEFPCM(water) Energy	-748.056188
M06-2X/def2tzvpp-IEFPCM(water) Free Energy (Quasiharmonic)	-747.90247

Datum	Value
Number of Imaginary Frequencies	0

Frequencies (Top 3 out of 60)

```
1.      39.5306 cm-1
2.      97.7714 cm-1
3.     125.2402 cm-1
```

M06-2X/def2tzvpp-IEFPCM(water) Molecular Geometry in Cartesian Coordinates

```
C          -2.176302       -1.052080       -0.320348
C          -0.916938       -1.025317       -0.867469
C           0.114671        0.055473       -0.846495
O          -2.826719       -0.201864        0.372691
H           0.342545        0.369682       -1.871800
C           1.472742       -1.657695        1.045575
H           1.542629       -1.101139        1.976660
H           2.203646       -2.463272        1.048735
H           0.471498       -2.077006        0.944226
S           1.800785       -0.619712       -0.392991
H          -2.701892       -2.003975       -0.552165
H          -0.639385       -1.911912       -1.428764
C          -0.249692        1.321523       -0.058269
H          -1.281852        1.540795       -0.337070
C          -0.216978        1.112305        1.450782
H           0.814436        1.030129        1.800755
H          -0.675332        1.961800        1.960818
H          -0.765176        0.212445        1.725187
C           0.627634        2.504431       -0.454545
H           1.675168        2.301459       -0.222260
H           0.552447        2.716241       -1.522748
H           0.331639        3.403207        0.089367
```

3_4methyl2pentenal_HEI_11

Datum	Value
M06-2X/def2tzvpp-IEFPCM(water) Energy	-748.056228
M06-2X/def2tzvpp-IEFPCM(water) Free Energy (Quasiharmonic)	-747.903943
Number of Imaginary Frequencies	0

Frequencies (Top 3 out of 60)

```
1.      39.9568 cm-1
2.      75.1045 cm-1
3.      94.4170 cm-1
```

M06-2X/def2tzvpp-IEFPCM(water) Molecular Geometry in Cartesian Coordinates

```
C        -0.564340       1.878985       0.272468
C        -0.169342       1.029292      -0.722716
C         0.142327      -0.429650      -0.559314
O        -0.812737       3.132057       0.202199
H         0.073135      -0.901054      -1.544109
C        -2.570680      -1.065700      -0.466530
H        -3.386254      -1.582036       0.035113
H        -2.774135       0.002726      -0.495090
H        -2.477103      -1.446125      -1.482974
S        -1.067145      -1.375862       0.470545
H        -0.696276       1.397386       1.262619
H        -0.058315       1.432458      -1.725971
C         1.552208      -0.781652      -0.024096
H         1.618250      -1.874269       0.007575
C         2.615912      -0.273620      -0.992731
H         2.591834       0.816377      -1.040427
H         3.611231      -0.579450      -0.666764
H         2.453227      -0.660326      -2.000454
C         1.818477      -0.246142       1.377838
H         1.772826       0.843935       1.379727
H         1.088723      -0.614806       2.099791
H         2.811717      -0.546546       1.715129
```

3_4methyl2pentenal_HEI_12_reopt

Datum	Value
M06-2X/def2tzvpp-IEFPCM(water) Energy	-748.060377
M06-2X/def2tzvpp-IEFPCM(water) Free Energy (Quasiharmonic)	-747.90724
Number of Imaginary Frequencies	0

Frequencies (Top 3 out of 60)

```
   1.      41.7730 cm-1
   2.      60.5470 cm-1
   3.      97.6296 cm-1
```

M06-2X/def2tzvpp-IEFPCM(water) Molecular Geometry in Cartesian Coordinates

```
 C          -2.576931       -0.797694        0.024301
 C          -1.279604       -1.083768       -0.311626
 C          -0.138492       -0.273106        0.195880
 O          -3.016196        0.150039        0.756019
 H          -0.450237        0.216442        1.122357
 C           0.623046        2.496165        0.221492
 H           0.559148        3.435946       -0.323634
 H          -0.097193        2.516872        1.038950
 H           1.626250        2.388035        0.625101
 S           0.189909        1.174847       -0.935450
 H          -3.325350       -1.498129       -0.399055
 H          -1.091182       -1.924842       -0.965487
 C           1.128998       -1.098617        0.478383
 H           0.807072       -1.863655        1.193534
 C           1.677175       -1.810545       -0.754745
 H           1.966404       -1.080834       -1.513677
 H           2.563290       -2.391001       -0.493241
 H           0.950521       -2.491738       -1.194943
 C           2.235302       -0.279754        1.135499
 H           2.666656        0.423595        0.420500
 H           1.861477        0.287657        1.989846
 H           3.037200       -0.932300        1.483699
```

3_4methyl2pentenal_HEI_13

Datum	Value
M06-2X/def2tzvpp-IEFPCM(water) Energy	-748.059015
M06-2X/def2tzvpp-IEFPCM(water) Free Energy (Quasiharmonic)	-747.905512
Number of Imaginary Frequencies	0

Frequencies (Top 3 out of 60)

```
   1.      57.7081 cm-1
   2.      78.4632 cm-1
   3.      94.6954 cm-1
```

M06-2X/def2tzvpp-IEFPCM(water) Molecular Geometry in Cartesian Coordinates

```
C          0.458998      -2.233533       0.007936
C         -0.051461      -1.248743       0.812232
C         -0.070112       0.211483       0.518015
O          1.037198      -2.143571      -1.124532
H         -0.221508       0.759520       1.454137
C         -2.866205      -0.059650       0.407072
H         -3.803932       0.178970      -0.090220
H         -2.732547      -1.138726       0.436520
H         -2.894421       0.330058       1.424283
S         -1.540866       0.714952      -0.532420
H          0.352309      -3.252439       0.437284
H         -0.534545      -1.570343       1.727250
C          1.184568       0.797679      -0.156390
H          1.248838       0.376212      -1.160394
C          1.124816       2.322225      -0.248295
H          0.996120       2.756826       0.747237
H          2.053435       2.714545      -0.665568
H          0.303415       2.670175      -0.872821
C          2.439884       0.382052       0.606010
H          2.381457       0.709674       1.647927
H          2.562181      -0.698423       0.589748
H          3.322546       0.844215       0.160117
```

3_4methyl2pentenal_HEI_14

Datum	Value
M06-2X/def2tzvpp-IEFPCM(water) Energy	-748.063057
M06-2X/def2tzvpp-IEFPCM(water) Free Energy (Quasiharmonic)	-747.910899
Number of Imaginary Frequencies	0

Frequencies (Top 3 out of 60)

```
1.      10.4434 cm-1
2.      44.9594 cm-1
3.      78.4348 cm-1
```

M06-2X/def2tzvpp-IEFPCM(water) Molecular Geometry in Cartesian Coordinates

```
C          -2.558761       -0.266258        0.390151
C          -1.304541        0.169155        0.731347
C          -0.124037       -0.004601       -0.161284
O          -2.940897       -0.852931       -0.676055
H          -0.486003       -0.259892       -1.159714
C           2.182425       -1.607705       -0.728081
H           2.935585       -0.851983       -0.513024
H           2.629360       -2.593081       -0.612145
H           1.838473       -1.496965       -1.755997
S           0.785216       -1.507853        0.415433
H          -3.332590       -0.064093        1.158847
H          -1.167372        0.631980        1.700847
C           0.798807        1.219920       -0.291930
H           1.613269        0.945484       -0.968280
C           0.038595        2.372345       -0.945124
H          -0.759169        2.723000       -0.288914
H           0.708781        3.209141       -1.148093
H          -0.414394        2.061908       -1.888508
C           1.415211        1.665253        1.028944
H           0.642159        1.992819        1.726469
H           1.976628        0.857316        1.498847
H           2.092800        2.504819        0.867045
```

3_4methyl2pentenal_HEI_1

Datum	Value
M06-2X/def2tzvpp-IEFPCM(water) Energy	-748.062562
M06-2X/def2tzvpp-IEFPCM(water) Free Energy (Quasiharmonic)	-747.90903
Number of Imaginary Frequencies	0

Frequencies (Top 3 out of 60)

```
1.      52.3327 cm-1
2.      66.5114 cm-1
3.      82.2815 cm-1
```

M06-2X/def2tzvpp-IEFPCM(water) Molecular Geometry in Cartesian Coordinates

```
C           2.096709       -1.376211        0.281680
C           0.852486       -0.976812        0.697296
C          -0.092419       -0.238842       -0.178860
O           2.657521       -1.219048       -0.854043
```

```
H        0.312657    -0.241697    -1.193049
C        1.510978     2.026813    -0.009146
H        1.753596     2.010327    -1.070310
H        1.657217     3.032404     0.378889
H        2.155363     1.326919     0.520740
S       -0.208410     1.570986     0.265163
H        2.677239    -1.916763     1.057656
H        0.567681    -1.180130     1.722888
C       -1.516173    -0.823370    -0.234443
H       -1.368029    -1.872807    -0.507309
C       -2.237036    -0.790876     1.110485
H       -2.430550     0.237586     1.422258
H       -3.198384    -1.301474     1.037377
H       -1.655146    -1.278054     1.892284
C       -2.379550    -0.179250    -1.316565
H       -2.639323     0.847082    -1.053648
H       -1.859228    -0.158808    -2.275739
H       -3.308682    -0.736696    -1.444982
```

3_4methyl2pentenal_HEI_2_reopt

Datum	Value
M06-2X/def2tzvpp-IEFPCM(water) Energy	-748.062571
M06-2X/def2tzvpp-IEFPCM(water) Free Energy (Quasiharmonic)	-747.909681
Number of Imaginary Frequencies	0

Frequencies (Top 3 out of 60)

```
1.     56.3983 cm-1
2.     66.3749 cm-1
3.     68.4523 cm-1
```

M06-2X/def2tzvpp-IEFPCM(water) Molecular Geometry in Cartesian Coordinates

```
C       -1.718864    -1.657512    -0.289788
C       -0.656342    -0.957162    -0.794185
C        0.206238    -0.067494     0.021222
O       -2.167165    -1.688883     0.905788
H        0.038603    -0.271012     1.082339
C       -1.996212     1.678957     0.161661
H       -2.427513     2.629016    -0.146706
H       -2.454840     0.863752    -0.395000
H       -2.168317     1.534429     1.226440
```

```
S         -0.235522      1.735911      -0.205797
H         -2.245580     -2.280962      -1.041663
H         -0.431350     -1.046833      -1.852274
C          1.708032     -0.218144      -0.279247
H          1.846237     -0.033624      -1.350640
C          2.584573      0.760205       0.500901
H          2.386353      0.670737       1.572303
H          3.639794      0.538284       0.335397
H          2.410718      1.796018       0.214254
C          2.155697     -1.647099       0.024304
H          2.017192     -1.864660       1.086865
H          1.581757     -2.374746      -0.545883
H          3.213888     -1.774419      -0.208191
```

3_4methyl2pentenal_HEI_3

Datum	Value
M06-2X/def2tzvpp-IEFPCM(water) Energy	-748.06354
M06-2X/def2tzvpp-IEFPCM(water) Free Energy (Quasiharmonic)	-747.910087
Number of Imaginary Frequencies	0

Frequencies (Top 3 out of 60)

```
  1.      57.7480 cm-1
  2.      70.2200 cm-1
  3.      89.2619 cm-1
```

M06-2X/def2tzvpp-IEFPCM(water) Molecular Geometry in Cartesian Coordinates

```
C         -1.150831      1.907229       0.648074
C         -0.294913      0.856481       0.852243
C          0.144352     -0.047676      -0.239081
O         -1.683947      2.313234      -0.439019
H         -0.241323      0.345424      -1.181885
C         -2.343466     -1.307104      -0.219652
H         -2.593625     -0.960293      -1.221027
H         -2.932939     -2.190982       0.013154
H         -2.558155     -0.520108       0.502243
S         -0.602580     -1.749463      -0.106493
H         -1.394538      2.475719       1.569322
H          0.037928      0.656681       1.864107
C          1.666025     -0.235577      -0.390686
H          1.823674     -0.983316      -1.174606
```

```
C          2.309675       1.069373      -0.850880
H          2.174636       1.844144      -0.094484
H          3.379846       0.934257      -1.015677
H          1.863092       1.423876      -1.781305
C          2.337853      -0.736722       0.884626
H          2.254386       0.008008       1.677998
H          1.888876      -1.664311       1.241646
H          3.398815      -0.919585       0.708696
```

3_4methyl2pentenal_HEI_4

Datum	Value
M06-2X/def2tzvpp-IEFPCM(water) Energy	-748.055402
M06-2X/def2tzvpp-IEFPCM(water) Free Energy (Quasiharmonic)	-747.902079
Number of Imaginary Frequencies	0

Frequencies (Top 3 out of 60)

```
1.      56.8000 cm-1
2.      88.2384 cm-1
3.     114.1695 cm-1
```

M06-2X/def2tzvpp-IEFPCM(water) Molecular Geometry in Cartesian Coordinates

```
C         -2.372532      -0.002674      -0.050397
C         -1.215286      -0.639798      -0.408699
C          0.148821      -0.042451      -0.524209
O         -3.548884      -0.500095       0.035668
H          0.562784      -0.246285      -1.517956
C          1.397585      -2.517907      -0.193044
H          2.178335      -3.106207       0.283923
H          0.443576      -3.023026      -0.058650
H          1.619107      -2.431878      -1.256273
S          1.392009      -0.891688       0.583303
H         -2.278794       1.073729       0.185038
H         -1.293529      -1.695182      -0.657371
C          0.256858       1.474808      -0.290581
H         -0.598079       1.913561      -0.810689
C          0.174946       1.875364       1.182828
H          1.102189       1.620805       1.701051
H          0.033367       2.953172       1.274674
H         -0.645676       1.374805       1.694390
C          1.520925       2.055590      -0.919988
```

```
H          1.560895        1.847575       -1.990103
H          1.553168        3.137717       -0.783847
H          2.413671        1.631389       -0.457839
```

3_4methyl2pentenal_HEI_5_reopt

Datum	Value
M06-2X/def2tzvpp-IEFPCM(water) Energy	-748.059736
M06-2X/def2tzvpp-IEFPCM(water) Free Energy (Quasiharmonic)	-747.906472
Number of Imaginary Frequencies	0

Frequencies (Top 3 out of 60)

```
1.      64.3784 cm-1
2.      76.8199 cm-1
3.     104.0134 cm-1
```

M06-2X/def2tzvpp-IEFPCM(water) Molecular Geometry in Cartesian Coordinates

```
C          2.460871       -0.630212       -0.328559
C          1.269347       -0.386983        0.302577
C         -0.018340       -0.345090       -0.432416
O          3.634773       -0.695287        0.161970
H          0.158202       -0.703680       -1.450003
C         -0.097643        2.300370        0.686245
H         -0.341414        3.347333        0.516872
H         -0.603956        1.957645        1.586281
H          0.977739        2.195218        0.812199
S         -0.643118        1.397186       -0.774395
H          2.362380       -0.797966       -1.424209
H          1.269826       -0.197814        1.371865
C         -1.138207       -1.214546        0.173305
H         -0.672110       -2.192989        0.329158
C         -1.633014       -0.707492        1.523232
H         -2.158605        0.241648        1.399425
H         -2.331314       -1.419710        1.965095
H         -0.813510       -0.557615        2.226767
C         -2.310275       -1.402336       -0.784496
H         -2.819524       -0.453122       -0.959263
H         -1.975203       -1.790839       -1.748020
H         -3.037249       -2.103049       -0.370934
```

3_4methyl2pentenal_HEI_6_reopt

Datum	Value
M06-2X/def2tzvpp-IEFPCM(water) Energy	-748.056352
M06-2X/def2tzvpp-IEFPCM(water) Free Energy (Quasiharmonic)	-747.903411
Number of Imaginary Frequencies	0

Frequencies (Top 3 out of 60)

```
1.      58.9854 cm-1
2.      73.3153 cm-1
3.      77.3794 cm-1
```

M06-2X/def2tzvpp-IEFPCM(water) Molecular Geometry in Cartesian Coordinates

```
C         -1.930909      -0.936274      -0.368427
C         -1.197327      -0.241456       0.554169
C          0.260405       0.045724       0.452374
O         -3.182817      -1.187521      -0.363422
H          0.668489       0.197456       1.456868
C         -0.588412       2.736941       0.402507
H         -0.457363       3.743817       0.011745
H         -1.598636       2.392194       0.194108
H         -0.425998       2.750990       1.479748
S          0.623609       1.683610      -0.409816
H         -1.350179      -1.340232      -1.222750
H         -1.722986       0.189120       1.401408
C          1.136502      -1.020972      -0.232144
H          0.802826      -1.130029      -1.267587
C          2.615071      -0.630754      -0.249318
H          2.960962      -0.418964       0.766116
H          3.216656      -1.450372      -0.644319
H          2.808909       0.249698      -0.859638
C          0.987948      -2.369576       0.471287
H         -0.034831      -2.735724       0.432252
H          1.643687      -3.110127       0.011036
H          1.273595      -2.277215       1.522767
```

3_4methyl2pentenal_HEI_7_reopt

Datum	Value
M06-2X/def2tzvpp-IEFPCM(water) Energy	-748.059201
M06-2X/def2tzvpp-IEFPCM(water) Free Energy (Quasiharmonic)	-747.906398
Number of Imaginary Frequencies	0

Frequencies (Top 3 out of 60)

```
1.      54.3414 cm-1
2.      67.3312 cm-1
3.      74.2457 cm-1
```

M06-2X/def2tzvpp-IEFPCM(water) Molecular Geometry in Cartesian Coordinates

```
C         -2.501080       0.010601      -0.327930
C         -1.303547      -0.014386       0.331375
C          0.004059       0.120545      -0.363650
O         -3.684398      -0.097006       0.138257
H         -0.163030       0.451045      -1.394836
C          1.227576      -1.988613       1.044088
H          1.564389      -3.022683       1.036781
H          2.016919      -1.363610       1.459756
H          0.334415      -1.911346       1.663124
S          0.846407      -1.523410      -0.653603
H         -2.404178       0.150750      -1.427802
H         -1.293232      -0.130264       1.412875
C          0.955638       1.128361       0.313551
H          1.071040       0.817594       1.357539
C          2.338037       1.200546      -0.328031
H          2.247604       1.402318      -1.398932
H          2.920147       2.008792       0.116989
H          2.894529       0.272538      -0.211923
C          0.320793       2.519356       0.306291
H          0.166311       2.854691      -0.723221
H         -0.644432       2.521076       0.807817
H          0.973343       3.241242       0.799258
```

3_4methyl2pentenal_HEI_8_reopt

Datum	Value
M06-2X/def2tzvpp-IEFPCM(water) Energy	-748.059773
M06-2X/def2tzvpp-IEFPCM(water) Free Energy (Quasiharmonic)	-747.906799

Datum	Value
Number of Imaginary Frequencies	0

Frequencies (Top 3 out of 60)

```
1.      51.3662 cm-1
2.      60.7087 cm-1
3.     105.3849 cm-1
```

M06-2X/def2tzvpp-IEFPCM(water) Molecular Geometry in Cartesian Coordinates

```
C         -2.359251       -0.081134       -0.214321
C         -1.088021       -0.009818        0.291299
C          0.101520        0.155238       -0.575811
O         -3.464251       -0.222251        0.404049
H         -0.243360        0.323449       -1.599541
C          0.865473       -2.272294        0.774049
H          1.374370       -3.230655        0.691669
H          1.281654       -1.718905        1.613619
H         -0.195918       -2.439361        0.942529
S          1.119125       -1.413374       -0.789401
H         -2.408321        0.005736       -1.322661
H         -0.947091       -0.094979        1.364379
C          1.044873        1.322176       -0.217251
H          1.859103        1.307713       -0.948091
C          0.314267        2.655358       -0.354421
H         -0.464323        2.744990        0.404189
H          1.009409        3.487626       -0.232621
H         -0.160783        2.747399       -1.333131
C          1.661853        1.191294        1.170469
H          0.887163        1.169796        1.939379
H          2.258610        0.282922        1.254859
H          2.313215        2.041352        1.379339
```

3_4methyl2pentenal_HEI_9

Datum	Value
M06-2X/def2tzvpp-IEFPCM(water) Energy	-748.059537
M06-2X/def2tzvpp-IEFPCM(water) Free Energy (Quasiharmonic)	-747.905485
Number of Imaginary Frequencies	0

Frequencies (Top 3 out of 60)

```
1.      45.0618 cm-1
2.      94.5538 cm-1
3.     123.8491 cm-1
```

M06-2X/def2tzvpp-IEFPCM(water) Molecular Geometry in Cartesian Coordinates

```
C        -1.458115        1.732865        0.222941
C        -0.655907        1.024827        1.078796
C         0.122914       -0.218439        0.789867
O        -1.775475        1.511903       -0.993779
H         0.223121       -0.803675        1.711456
C         1.724336        1.146421       -1.058391
H         1.514055        0.506549       -1.913923
H         2.653949        1.683119       -1.234323
H         0.907274        1.853872       -0.921504
S         1.913118        0.181866        0.450879
H        -1.903936        2.632045        0.699482
H        -0.515857        1.450223        2.066538
C        -0.466681       -1.158200       -0.276384
H        -0.498860       -0.614995       -1.221144
C         0.355833       -2.432185       -0.447424
H         0.460748       -2.950484        0.510594
H        -0.139020       -3.113303       -1.141524
H         1.355566       -2.224949       -0.824797
C        -1.903134       -1.522091        0.096365
H        -1.932035       -1.993351        1.083456
H        -2.532778       -0.635830        0.110016
H        -2.313791       -2.233486       -0.622785
```

3_4methyl2pentenal_TS_10_reopt

Datum	Value
M06-2X/def2tzvpp-IEFPCM(water) Energy	-748.040295
M06-2X/def2tzvpp-IEFPCM(water) Free Energy (Quasiharmonic)	-747.887866
Number of Imaginary Frequencies	1

Frequencies (Top 3 out of 60)

```
1.    -194.3420 cm-1
2.      77.5287 cm-1
3.      91.0863 cm-1
```

M06-2X/def2tzvpp-IEFPCM(water) Molecular Geometry in Cartesian Coordinates

```
C          -2.014532       -1.353347       -0.350691
C          -0.925625       -0.876409       -1.137470
C          -0.227747        0.302171       -0.983804
O          -2.574692       -0.810992        0.606082
H           0.330735        0.641583       -1.845301
C           1.568307       -1.718459        0.796881
H           1.544695       -1.411519        1.843329
H           2.190743       -2.608826        0.711222
H           0.546798       -1.991887        0.502147
S           2.159217       -0.391593       -0.276506
H          -2.414200       -2.324723       -0.701973
H          -0.654269       -1.518332       -1.967568
C          -0.573181        1.377957        0.019783
H          -1.657911        1.509018       -0.065959
C          -0.286075        1.012609        1.476607
H           0.788141        1.043039        1.655343
H          -0.777916        1.728483        2.138505
H          -0.653861        0.017463        1.713445
C           0.113385        2.690596       -0.341518
H           1.196763        2.559039       -0.300013
H          -0.154452        3.020216       -1.346424
H          -0.162382        3.479164        0.359963
```

3_4methyl2pentenal_TS_11_reopt

Datum	Value
M06-2X/def2tzvpp-IEFPCM(water) Energy	-748.041224
M06-2X/def2tzvpp-IEFPCM(water) Free Energy (Quasiharmonic)	-747.889344
Number of Imaginary Frequencies	1

Frequencies (Top 3 out of 60)

```
1.    -176.7543 cm-1
2.      63.9120 cm-1
3.      78.7026 cm-1
```

M06-2X/def2tzvpp-IEFPCM(water) Molecular Geometry in Cartesian Coordinates

```
C          1.204245       1.670590       0.178634
C          0.585227       0.968873      -0.889103
C          0.231646      -0.365125      -0.912028
O          1.529997       2.864281       0.151548
H         -0.038910      -0.740456      -1.890609
C         -2.274319       0.889700       0.691790
H         -2.382016       0.585008       1.733433
H         -1.330342       1.441427       0.595600
H         -3.085339       1.576113       0.450486
S         -2.239357      -0.525533      -0.427480
H          1.424525       1.093482       1.088619
H          0.343751       1.571938      -1.759165
C          0.721785      -1.475110      -0.002606
H          0.117899      -2.343595      -0.266472
C          2.179178      -1.804358      -0.353411
H          2.832057      -0.972152      -0.085771
H          2.507090      -2.688272       0.196259
H          2.299004      -2.001121      -1.419471
C          0.569479      -1.271220       1.505512
H          1.393857      -0.696889       1.927045
H         -0.367912      -0.769327       1.740918
H          0.572630      -2.241984       2.003702
```

3_4methyl2pentenal_TS_12_reopt

Datum	Value
M06-2X/def2tzvpp-IEFPCM(water) Energy	-748.041188
M06-2X/def2tzvpp-IEFPCM(water) Free Energy (Quasiharmonic)	-747.890445
Number of Imaginary Frequencies	1

Frequencies (Top 3 out of 60)

```
1.    -182.4349 cm-1
2.      33.4077 cm-1
3.      83.6796 cm-1
```

M06-2X/def2tzvpp-IEFPCM(water) Molecular Geometry in Cartesian Coordinates

```
C          -2.802532       0.134759       -0.137864
C          -1.645017      -0.648923       -0.406871
C          -0.519469      -0.571060        0.382966
O          -2.960814       0.934556        0.789712
H          -0.625288      -0.016733        1.307934
C           2.144623       2.138274        0.103313
H           2.195318       3.210900       -0.086606
H           2.167461       1.990593        1.183462
H           3.040021       1.678376       -0.316012
S           0.618185       1.433826       -0.613178
H          -3.639110      -0.014640       -0.846279
H          -1.667525      -1.269944       -1.292376
C           0.565532      -1.616144        0.389640
H           0.130044      -2.427956        0.989749
C           0.889292      -2.179532       -0.988530
H           1.235215      -1.372706       -1.636590
H           1.679056      -2.927874       -0.912648
H           0.025104      -2.652789       -1.453233
C           1.833222      -1.166314        1.107018
H           2.398354      -0.478625        0.479881
H           1.599298      -0.655762        2.042864
H           2.463714      -2.026869        1.334961
```

3_4methyl2pentenal_TS_13

Datum	Value
M06-2X/def2tzvpp-IEFPCM(water) Energy	-748.044411
M06-2X/def2tzvpp-IEFPCM(water) Free Energy (Quasiharmonic)	-747.892723
Number of Imaginary Frequencies	1

Frequencies (Top 3 out of 60)

```
  1.    -145.8993 cm-1
  2.      59.7193 cm-1
  3.      69.5881 cm-1
```

M06-2X/def2tzvpp-IEFPCM(water) Molecular Geometry in Cartesian Coordinates

```
C           0.243068      -2.232001        0.080831
C           0.090694      -1.183936        1.043711
C           0.378872       0.138864        0.839937
O           0.659182      -2.133854       -1.072948
```

```
H          0.232962       0.796765       1.690056
C         -2.810332      -0.373019      -0.127761
H         -3.168403      -0.638115      -1.122655
H         -2.163034      -1.189103       0.219417
H         -3.669816      -0.323252       0.541070
S         -1.868674       1.168720      -0.147039
H         -0.064958      -3.230561       0.445145
H         -0.384203      -1.475862       1.972547
C          1.305478       0.686996      -0.217307
H          0.969701       0.336750      -1.191299
C          1.340233       2.210746      -0.199063
H          1.669573       2.570410       0.779395
H          2.042257       2.586706      -0.944523
H          0.357142       2.630548      -0.405592
C          2.717250       0.134805       0.027441
H          3.078410       0.433455       1.014215
H          2.724391      -0.951950      -0.030980
H          3.409730       0.530791      -0.717323
```

3_4methyl2pentenal_TS_14

Datum	Value
M06-2X/def2tzvpp-IEFPCM(water) Energy	-748.042991
M06-2X/def2tzvpp-IEFPCM(water) Free Energy (Quasiharmonic)	-747.891822
Number of Imaginary Frequencies	1

Frequencies (Top 3 out of 60)

```
1.    -201.7196 cm-1
2.      51.0479 cm-1
3.      83.7100 cm-1
```

M06-2X/def2tzvpp-IEFPCM(water) Molecular Geometry in Cartesian Coordinates

```
C         -2.689455      -0.492755       0.382201
C         -1.514358       0.273697       0.623679
C         -0.476185       0.305210      -0.277814
O         -2.914549      -1.215063      -0.593936
H         -0.679630      -0.114208      -1.255066
C          2.664176      -1.291338      -0.356551
H          3.009847      -0.431374       0.220513
H          3.355707      -2.114799      -0.175554
H          2.724693      -1.033635      -1.414652
```

```
S          0.970663       -1.758792        0.119737
H         -3.466937       -0.415006        1.165148
H         -1.446410        0.786604        1.574418
C          0.621431        1.334689       -0.241121
H          1.450043        0.958843       -0.842073
C          0.109204        2.616036       -0.914830
H         -0.702637        3.052870       -0.331352
H          0.913608        3.349288       -0.988589
H         -0.263581        2.416023       -1.920074
C          1.134710        1.632721        1.161287
H          0.363367        2.109697        1.768635
H          1.445421        0.713137        1.658049
H          1.985164        2.314177        1.115185
```

3_4methyl2pentenal_TS_1

Datum	Value
M06-2X/def2tzvpp-IEFPCM(water) Energy	-748.047486
M06-2X/def2tzvpp-IEFPCM(water) Free Energy (Quasiharmonic)	-747.895836
Number of Imaginary Frequencies	1

Frequencies (Top 3 out of 60)

```
1.    -144.4930 cm-1
2.      56.5960 cm-1
3.      84.1070 cm-1
```

M06-2X/def2tzvpp-IEFPCM(water) Molecular Geometry in Cartesian Coordinates

```
C          2.048478       -1.460567        0.154202
C          0.683161       -1.280083        0.543047
C         -0.244193       -0.778657       -0.326765
O          2.534883       -1.215531       -0.949883
H          0.067063       -0.684697       -1.359416
C          1.585179        1.825183        0.742146
H          2.485517        2.090775        0.187181
H          1.488928        2.507065        1.587059
H          1.730773        0.814889        1.146148
S          0.110800        1.844350       -0.300185
H          2.710191       -1.862972        0.943809
H          0.434952       -1.504813        1.572340
C         -1.732787       -0.842393       -0.103295
H         -1.984942       -1.888596       -0.326174
```

```
C          -2.152252       -0.562975        1.335152
H          -1.854775        0.450288        1.609510
H          -3.235096       -0.647566        1.434935
H          -1.698114       -1.260862        2.037787
C          -2.524160        0.016832       -1.084615
H          -2.423643        1.072221       -0.833888
H          -2.171427       -0.127404       -2.107125
H          -3.581847       -0.247729       -1.049382
```

3_4methyl2pentenal_TS_2

Datum	Value
M06-2X/def2tzvpp-IEFPCM(water) Energy	-748.047304
M06-2X/def2tzvpp-IEFPCM(water) Free Energy (Quasiharmonic)	-747.896043
Number of Imaginary Frequencies	1

Frequencies (Top 3 out of 60)

```
  1.    -142.7631 cm-1
  2.      46.2474 cm-1
  3.      77.1197 cm-1
```

M06-2X/def2tzvpp-IEFPCM(water) Molecular Geometry in Cartesian Coordinates

```
C           1.360982        2.017131       -0.061959
C           0.255378        1.289114       -0.606591
C          -0.476666        0.417968        0.146385
O           1.769388        1.965430        1.097843
H          -0.301620        0.420282        1.215696
C           2.383804       -1.155389       -0.669015
H           2.663875       -1.758810       -1.532385
H           2.154016       -0.145098       -1.032612
H           3.247443       -1.081160       -0.007624
S           0.935355       -1.829300        0.172357
H           1.879641        2.684762       -0.774761
H           0.051162        1.415093       -1.664082
C          -1.796744       -0.145517       -0.312531
H          -1.662674       -0.562273       -1.313771
C          -2.331930       -1.226688        0.618379
H          -2.447282       -0.826601        1.628911
H          -3.311383       -1.569899        0.282967
H          -1.659576       -2.081036        0.662705
C          -2.814659        1.001722       -0.404954
```

```
H          -2.939549        1.476944        0.570682
H          -2.489420        1.762835       -1.112634
H          -3.786400        0.620273       -0.721839
```

3_4methyl2pentenal_TS_3

Datum	Value
M06-2X/def2tzvpp-IEFPCM(water) Energy	-748.048293
M06-2X/def2tzvpp-IEFPCM(water) Free Energy (Quasiharmonic)	-747.896631
Number of Imaginary Frequencies	1

Frequencies (Top 3 out of 60)

```
1.    -159.1055 cm-1
2.      47.2820 cm-1
3.      77.8179 cm-1
```

M06-2X/def2tzvpp-IEFPCM(water) Molecular Geometry in Cartesian Coordinates

```
C          -1.151889        2.028077        0.457287
C          -0.059284        1.130452        0.680192
C           0.472363        0.382469       -0.333390
O          -1.719280        2.240707       -0.614316
H           0.159162        0.636351       -1.337849
C          -2.335243       -1.175895        0.555595
H          -3.283036       -0.941630        0.070234
H          -2.517701       -1.936536        1.314666
H          -1.990466       -0.269219        1.070494
S          -1.074830       -1.697188       -0.627267
H          -1.501520        2.571159        1.355106
H           0.280380        1.020326        1.702113
C           1.780745       -0.356442       -0.235750
H           1.741261       -1.186266       -0.942747
C           2.910697        0.582636       -0.680000
H           3.003046        1.421220        0.012290
H           3.861267        0.047550       -0.698069
H           2.726165        0.983418       -1.677362
C           2.060375       -0.915743        1.152192
H           2.209959       -0.112673        1.876212
H           1.229190       -1.536260        1.488123
H           2.967226       -1.521414        1.140830
```

3_4methyl2pentenal_TS_4_reopt

Datum	Value
M06-2X/def2tzvpp-IEFPCM(water) Energy	-748.039783
M06-2X/def2tzvpp-IEFPCM(water) Free Energy (Quasiharmonic)	-747.888584
Number of Imaginary Frequencies	1

Frequencies (Top 3 out of 60)

```
1.    -200.8048 cm-1
2.      41.0501 cm-1
3.      71.9174 cm-1
```

M06-2X/def2tzvpp-IEFPCM(water) Molecular Geometry in Cartesian Coordinates

```
C         -2.400588       -0.206089        0.115552
C         -1.258208       -0.572065       -0.642982
C         -0.102477        0.177295       -0.723081
O         -3.442346       -0.863692        0.213851
H          0.579897       -0.089109       -1.520732
C          1.451789       -2.453270       -0.330908
H          2.135340       -3.242099       -0.019168
H          0.432186       -2.810258       -0.177453
H          1.593082       -2.290381       -1.401472
S          1.746000       -0.930605        0.611181
H         -2.350239        0.768593        0.631561
H         -1.317154       -1.510461       -1.182775
C          0.027933        1.612439       -0.239605
H         -0.891806        2.117719       -0.550765
C          0.149854        1.789789        1.276105
H          1.157590        1.536262        1.603854
H         -0.046433        2.829670        1.543043
H         -0.546851        1.155481        1.821588
C          1.195181        2.303614       -0.939283
H          1.071280        2.294555       -2.022797
H          1.280026        3.341560       -0.615193
H          2.124955        1.787396       -0.694180
```

3_4methyl2pentenal_TS_5_reopt3

Datum	Value
M06-2X/def2tzvpp-IEFPCM(water) Energy	-748.048698
M06-2X/def2tzvpp-IEFPCM(water) Free Energy (Quasiharmonic)	-747.897228
Number of Imaginary Frequencies	1

Frequencies (Top 3 out of 60)

```
1.    -163.5265 cm-1
2.      50.0915 cm-1
3.      80.3001 cm-1
```

M06-2X/def2tzvpp-IEFPCM(water) Molecular Geometry in Cartesian Coordinates

```
C          2.265285      -0.875265      -0.453713
C          0.992204      -1.015273       0.158666
C         -0.146087      -0.607190      -0.499832
O          3.353788      -1.232215       0.006121
H         -0.019639      -0.317182      -1.537116
C          1.173885       2.234998       0.412764
H          1.698721       2.764726      -0.382396
H          1.226452       2.839195       1.318051
H          1.710080       1.299251       0.607153
S         -0.534615       1.855577      -0.030686
H          2.239650      -0.403369      -1.456758
H          0.954536      -1.431814       1.158388
C         -1.523755      -1.129265      -0.165800
H         -1.511812      -2.151632      -0.568064
C         -1.801785      -1.228604       1.329433
H         -1.753126      -0.235291       1.777045
H         -2.798498      -1.637918       1.498869
H         -1.086410      -1.874052       1.837747
C         -2.648013      -0.390198      -0.884576
H         -2.827759       0.582162      -0.426632
H         -2.407891      -0.232191      -1.937114
H         -3.571163      -0.968613      -0.828812
```

3_4methyl2pentenal_TS_6

Datum	Value
M06-2X/def2tzvpp-IEFPCM(water) Energy	-748.044861
M06-2X/def2tzvpp-IEFPCM(water) Free Energy (Quasiharmonic)	-747.893366

Datum	Value
Number of Imaginary Frequencies	1

Frequencies (Top 3 out of 60)

```
1.    -149.5598 cm-1
2.      42.6122 cm-1
3.      74.2561 cm-1
```

M06-2X/def2tzvpp-IEFPCM(water) Molecular Geometry in Cartesian Coordinates

```
C          0.183317        2.116694       -0.296001
C          0.188674        1.178224        0.770687
C         -0.403126       -0.063895        0.696587
O          0.694860        3.240162       -0.277618
H         -0.435588       -0.632171        1.618949
C          2.750220       -0.531045       -0.113083
H          3.274943       -0.545518       -1.067966
H          2.354267        0.480644        0.035724
H          3.473576       -0.730266        0.677525
S          1.377502       -1.704664       -0.096783
H         -0.333677        1.792498       -1.217778
H          0.747506        1.452429        1.658302
C         -1.495060       -0.444093       -0.283602
H         -1.146166       -0.271035       -1.302873
C         -1.894357       -1.909814       -0.144767
H         -2.231247       -2.110439        0.875555
H         -2.718133       -2.145646       -0.819376
H         -1.063224       -2.575274       -0.368481
C         -2.728728        0.435418       -0.033739
H         -2.512639        1.488895       -0.204316
H         -3.545789        0.134493       -0.691089
H         -3.068392        0.325786        0.998810
```

3_4methyl2pentenal_TS_7_reopt

Datum	Value
M06-2X/def2tzvpp-IEFPCM(water) Energy	-748.048605
M06-2X/def2tzvpp-IEFPCM(water) Free Energy (Quasiharmonic)	-747.897766
Number of Imaginary Frequencies	1

Frequencies (Top 3 out of 60)

```
1.    -153.0426 cm-1
2.      37.1393 cm-1
3.      65.1461 cm-1
```

M06-2X/def2tzvpp-IEFPCM(water) Molecular Geometry in Cartesian Coordinates

```
C         -1.839648       -1.434292        0.431906
C         -0.680211       -1.091526       -0.314322
C          0.349469       -0.392604        0.265667
O         -2.807852       -2.089036        0.038545
H          0.299853       -0.241007        1.339305
C         -1.910912        1.936210       -0.284592
H         -2.508156        2.292112        0.554841
H         -2.220491        2.478274       -1.177815
H         -2.145688        0.876951       -0.443102
S         -0.139198        2.106548        0.016570
H         -1.831003       -1.068460        1.478367
H         -0.634614       -1.385004       -1.358566
C          1.739308       -0.384521       -0.324894
H          1.661144       -0.090256       -1.374316
C          2.693786        0.560407        0.395283
H          2.730320        0.316406        1.460114
H          3.703435        0.456736       -0.003925
H          2.388161        1.599391        0.293769
C          2.300379       -1.813684       -0.268675
H          2.346929       -2.162156        0.765726
H          1.676402       -2.506734       -0.830706
H          3.310660       -1.838682       -0.679409
```

3_4methyl2pentenal_TS_8_reopt2

Datum	Value
M06-2X/def2tzvpp-IEFPCM(water) Energy	-748.049605
M06-2X/def2tzvpp-IEFPCM(water) Free Energy (Quasiharmonic)	-747.898555
Number of Imaginary Frequencies	1

Frequencies (Top 3 out of 60)

```
1.    -178.1133 cm-1
2.      29.0902 cm-1
3.      75.6837 cm-1
```

M06-2X/def2tzvpp-IEFPCM(water) Molecular Geometry in Cartesian Coordinates

```
C         -1.417716      1.814705     -0.249452
C         -0.296054      1.134322      0.297739
C          0.397243      0.212283     -0.450909
O         -2.102193      2.678108      0.305682
H          0.122487      0.139598     -1.497745
C         -2.308063     -1.502510      0.057582
H         -2.910559     -1.616586     -0.843690
H         -2.806295     -2.030721      0.870033
H         -2.286659     -0.438208      0.319995
S         -0.612722     -2.079328     -0.172728
H         -1.663134      1.527233     -1.291515
H         -0.024353      1.351749      1.324284
C          1.819205     -0.196205     -0.153803
H          1.982615     -1.177581     -0.601634
C          2.765644      0.795368     -0.843880
H          2.652441      1.791858     -0.413815
H          3.802026      0.481139     -0.712172
H          2.562651      0.861202     -1.913440
C          2.130559     -0.281542      1.334460
H          2.086163      0.704707      1.800122
H          1.421573     -0.936385      1.840259
H          3.137231     -0.672148      1.487089
```

3_4methyl2pentenal_TS_9

Datum	Value
M06-2X/def2tzvpp-IEFPCM(water) Energy	-748.044411
M06-2X/def2tzvpp-IEFPCM(water) Free Energy (Quasiharmonic)	-747.892722
Number of Imaginary Frequencies	1

Frequencies (Top 3 out of 60)

```
1.    -145.9067 cm-1
2.      59.7340 cm-1
3.      69.5769 cm-1
```

M06-2X/def2tzvpp-IEFPCM(water) Molecular Geometry in Cartesian Coordinates

```
C          -0.243050        2.232039        0.080805
C          -0.090703        1.183978        1.043689
C          -0.378813       -0.138802        0.839926
O          -0.659039        2.133869       -1.073023
H          -0.233057       -0.796634        1.690127
C           2.810245        0.372958       -0.127783
H           3.167939        0.638256       -1.122756
H           3.669967        0.323038        0.540725
H           2.163058        1.188976        0.219813
S           1.868500       -1.168735       -0.146960
H           0.064840        3.230627        0.445160
H           0.384092        1.475935        1.972563
C          -1.305424       -0.686982       -0.217298
H          -0.969706       -0.336642       -1.191276
C          -1.340050       -2.210733       -0.199184
H          -1.669213       -2.570530        0.779284
H          -2.042153       -2.586677       -0.944578
H          -0.356955       -2.630431       -0.405905
C          -2.717215       -0.134880        0.027534
H          -3.078341       -0.433641        1.014287
H          -2.724403        0.951881       -0.030774
H          -3.409693       -0.530815       -0.717258
```

4_3-methyl-2-butenal_1

Datum	Value
M06-2X/def2tzvpp-IEFPCM(water) Energy	-270.536544
M06-2X/def2tzvpp-IEFPCM(water) Free Energy (Quasiharmonic)	-270.445858
Number of Imaginary Frequencies	0

Frequencies (Top 3 out of 36)

```
1.     109.1233 cm-1
2.     127.1964 cm-1
3.     188.3092 cm-1
```

M06-2X/def2tzvpp-IEFPCM(water) Molecular Geometry in Cartesian Coordinates

```
C          1.455132       0.732593       0.000000
C          0.000000       0.669388       0.000000
O          2.073261       1.778579       0.000000
C         -0.729093      -0.457990       0.000000
C         -2.224660      -0.389977       0.000000
H         -2.619612      -0.909325       0.875731
H          2.006431      -0.219031       0.000000
H         -2.619612      -0.909325      -0.875731
H         -2.588354       0.634490       0.000000
C         -0.178195      -1.850977       0.000000
H         -0.499841       1.631626       0.000000
H         -0.554376      -2.384401       0.875119
H         -0.554376      -2.384401      -0.875119
H          0.904553      -1.906487       0.000000
```

4_3-methyl-2-butenal_2

Datum	Value
M06-2X/def2tzvpp-IEFPCM(water) Energy	-270.533343
M06-2X/def2tzvpp-IEFPCM(water) Free Energy (Quasiharmonic)	-270.442682
Number of Imaginary Frequencies	0

Frequencies (Top 3 out of 36)

```
1.      45.7040 cm-1
2.     158.7467 cm-1
3.     206.6602 cm-1
```

M06-2X/def2tzvpp-IEFPCM(water) Molecular Geometry in Cartesian Coordinates

```
C          1.638163      -0.639888      -0.005787
C          0.211152      -0.949676      -0.001293
O          2.131375       0.470672       0.010782
C         -0.779466      -0.040936      -0.001606
C         -2.209336      -0.477601       0.004757
H         -2.719828      -0.075603      -0.873426
H          2.299437      -1.523464      -0.025395
H         -2.717105      -0.057610       0.876109
H         -2.312842      -1.559805       0.016025
C         -0.549501       1.441027      -0.006843
H         -0.036212      -2.004258       0.000150
H         -1.495564       1.976663      -0.048626
```

```
H          -0.005270        1.746894        0.887151
H           0.070311        1.734244       -0.853614
```

4_3methyl2butenal_HEI_1_reopt

Datum	Value
M06-2X/def2tzvpp-IEFPCM(water) Energy	-708.751636
M06-2X/def2tzvpp-IEFPCM(water) Free Energy (Quasiharmonic)	-708.62551
Number of Imaginary Frequencies	0

Frequencies (Top 3 out of 51)

```
1.      57.7972 cm-1
2.      70.8871 cm-1
3.     161.7503 cm-1
```

M06-2X/def2tzvpp-IEFPCM(water) Molecular Geometry in Cartesian Coordinates

```
C          -2.177944       -0.228024       -0.221621
C          -0.997152       -0.198924        0.470778
C           0.189792        0.650022        0.160623
O          -3.212735       -0.939879        0.012802
C           1.701109       -1.776240        0.265656
H           2.055466       -1.532833        1.265797
H           0.748288       -2.297848        0.330830
H           2.431792       -2.422583       -0.216089
S           1.537268       -0.304429       -0.756789
H          -2.239622        0.460554       -1.085877
H          -0.900445       -0.858684        1.330162
C          -0.092030        1.819352       -0.780611
H          -0.452819        1.484318       -1.751851
H          -0.847548        2.470934       -0.338493
H           0.816872        2.403083       -0.938095
C           0.813039        1.186546        1.450695
H           1.712829        1.768182        1.246086
H           0.088676        1.825538        1.960926
H           1.071226        0.372852        2.129679
```

4_3methyl2butenal_HEI_2_reopt

Datum	Value
M06-2X/def2tzvpp-IEFPCM(water) Energy	-708.753779
M06-2X/def2tzvpp-IEFPCM(water) Free Energy (Quasiharmonic)	-708.627524
Number of Imaginary Frequencies	0

Frequencies (Top 3 out of 51)

```
1.      73.6207 cm-1
2.      94.3094 cm-1
3.     158.3724 cm-1
```

M06-2X/def2tzvpp-IEFPCM(water) Molecular Geometry in Cartesian Coordinates

```
C          2.085757       -0.159826        0.144805
C          1.011266        0.362886       -0.523863
C         -0.315730        0.705144        0.057990
O          3.229405       -0.481056       -0.327979
C         -0.978167       -2.055117        0.220675
H          0.103343       -2.066181        0.076035
H         -1.208913       -2.141606        1.280479
H         -1.420667       -2.895625       -0.309074
S         -1.633273       -0.536764       -0.486070
H          1.942036       -0.307822        1.232471
H          1.102405        0.506246       -1.599007
C         -0.344196        0.771528        1.582329
H         -0.101547       -0.186135        2.040618
H          0.385202        1.504369        1.933785
H         -1.335016        1.071869        1.924416
C         -0.840140        2.022711       -0.516139
H         -1.857444        2.230648       -0.177678
H         -0.191980        2.837737       -0.189254
H         -0.833032        1.999214       -1.606609
```

4_3methyl2butenal_HEI_3

Datum	Value
M06-2X/def2tzvpp-IEFPCM(water) Energy	-708.751636
M06-2X/def2tzvpp-IEFPCM(water) Free Energy (Quasiharmonic)	-708.625506
Number of Imaginary Frequencies	0

Frequencies (Top 3 out of 51)

```
1.      58.0269 cm-1
2.      70.8933 cm-1
3.     161.9189 cm-1
```

M06-2X/def2tzvpp-IEFPCM(water) Molecular Geometry in Cartesian Coordinates

```
C        -2.178043      -0.228208      -0.221573
C        -0.997237      -0.199096       0.470796
C         0.189665       0.649932       0.160661
O        -3.212787      -0.940139       0.012814
C         1.702057      -1.775626       0.265774
H         2.058137      -1.531773       1.265187
H         0.749166      -2.296899       0.332747
H         2.431730      -2.422486      -0.216807
S         1.537005      -0.304306      -0.757175
H        -2.239775       0.460439      -1.085777
H        -0.900441      -0.858909       1.330119
C        -0.092315       1.819465      -0.780272
H        -0.453120       1.484634      -1.751579
H        -0.847889       2.470848      -0.337958
H         0.816517       2.403327      -0.937661
C         0.813048       1.186150       1.450784
H         1.712907       1.767712       1.246253
H         0.088789       1.825141       1.961162
H         1.071138       0.372272       2.129589
```

4_3methyl2butenal_HEI_4

Datum	Value
M06-2X/def2tzvpp-IEFPCM(water) Energy	-708.7526
M06-2X/def2tzvpp-IEFPCM(water) Free Energy (Quasiharmonic)	-708.626411
Number of Imaginary Frequencies	0

Frequencies (Top 3 out of 51)

```
1.      61.7198 cm-1
2.      79.3981 cm-1
3.     164.6308 cm-1
```

M06-2X/def2tzvpp-IEFPCM(water) Molecular Geometry in Cartesian Coordinates

```
C          2.362695      -0.192805      -0.253665
C          1.251735      -0.014626       0.525857
C         -0.091936       0.465390       0.067663
O          3.516683      -0.609385       0.104009
C         -2.855508      -0.446549      -0.173463
H         -3.215994       0.137158       0.670610
H         -2.919506       0.145768      -1.084321
H         -3.490394      -1.323532      -0.287454
S         -1.173888      -1.055653       0.102442
H          2.241682       0.067603      -1.321932
H          1.324707      -0.262403       1.582903
C         -0.105480       1.044064      -1.343379
H          0.221710       0.315173      -2.082170
H         -1.109578       1.375535      -1.610960
H          0.557908       1.910422      -1.393351
C         -0.659918       1.496667       1.042896
H         -0.680391       1.098063       2.057554
H         -0.021218       2.382486       1.033896
H         -1.669724       1.806406       0.768618
```

4_3methyl2butenal_HEI_5

Datum	Value
M06-2X/def2tzvpp-IEFPCM(water) Energy	-708.753445
M06-2X/def2tzvpp-IEFPCM(water) Free Energy (Quasiharmonic)	-708.626703
Number of Imaginary Frequencies	0

Frequencies (Top 3 out of 51)

```
  1.      82.4592 cm-1
  2.      96.7297 cm-1
  3.     184.8644 cm-1
```

M06-2X/def2tzvpp-IEFPCM(water) Molecular Geometry in Cartesian Coordinates

```
C         -2.055218       0.188164      -0.631575
C         -0.789274       0.657291      -0.881876
C          0.383445       0.697560       0.042016
```

```
O          -2.565646       -0.327944        0.415996
C           0.346517       -2.142286        0.037815
H           0.082905       -2.258162        1.087263
H          -0.553264       -1.946552       -0.543651
H           0.823671       -3.054702       -0.313697
S           1.506477       -0.789247       -0.209463
H          -2.726494        0.295277       -1.510983
H          -0.601157        1.008384       -1.891827
C           0.009890        0.766965        1.520629
H          -0.543343       -0.113725        1.832383
H           0.908263        0.874727        2.131052
H          -0.635263        1.632821        1.688071
C           1.292977        1.878100       -0.302662
H           1.569111        1.863707       -1.357666
H           0.761506        2.809801       -0.100653
H           2.205578        1.865178        0.297060
```

4_3methyl2butenal_HEI_6

Datum	Value
M06-2X/def2tzvpp-IEFPCM(water) Energy	-708.753076
M06-2X/def2tzvpp-IEFPCM(water) Free Energy (Quasiharmonic)	-708.626992
Number of Imaginary Frequencies	0

Frequencies (Top 3 out of 51)

```
  1.      48.0186 cm-1
  2.      92.4239 cm-1
  3.     161.3385 cm-1
```

M06-2X/def2tzvpp-IEFPCM(water) Molecular Geometry in Cartesian Coordinates

```
C           2.004689       -0.660063        0.624645
C           0.873806        0.018288        1.006569
C          -0.099853        0.715667        0.114595
O           2.486714       -0.867633       -0.535309
C          -1.822185       -1.531727        0.426014
H          -1.006148       -1.858437        1.069756
H          -2.576485       -1.018054        1.019158
H          -2.276756       -2.399169       -0.047687
S          -1.161307       -0.481836       -0.877587
H           2.567552       -1.084158        1.483473
H           0.641463        0.011520        2.066017
```

```
C          0.569327       1.569016       -0.963616
H          1.268219       0.969266       -1.541381
H         -0.174550       2.014236       -1.629080
H          1.123050       2.376737       -0.480291
C         -1.039189       1.589214        0.943478
H         -1.578324       0.997168        1.684374
H         -0.455326       2.340601        1.480644
H         -1.765068       2.098369        0.308776
```

4_3methyl2butenal_HEI_7_reopt

Datum	Value
M06-2X/def2tzvpp-IEFPCM(water) Energy	-708.7526
M06-2X/def2tzvpp-IEFPCM(water) Free Energy (Quasiharmonic)	-708.626411
Number of Imaginary Frequencies	0

Frequencies (Top 3 out of 51)

```
  1.      61.7116 cm-1
  2.      79.3979 cm-1
  3.     164.6184 cm-1
```

M06-2X/def2tzvpp-IEFPCM(water) Molecular Geometry in Cartesian Coordinates

```
C          2.362704      -0.192802        0.253633
C          1.251731      -0.014609       -0.525867
C         -0.091943       0.465374       -0.067648
O          3.516704      -0.609331       -0.104071
C         -2.855502      -0.446494        0.173359
H         -2.919511       0.146005        1.084094
H         -3.215939       0.137053       -0.670841
H         -3.490414      -1.323436        0.287520
S         -1.173885      -1.055679       -0.102379
H          2.241696       0.067547        1.321917
H          1.324701      -0.262299       -1.582936
C         -0.659974       1.496652       -1.042852
H         -0.680505       1.098052       -2.057510
H         -1.669763       1.806378       -0.768498
H         -0.021278       2.382474       -1.033876
C         -0.105457       1.044012        1.343404
H         -1.109513       1.375599        1.611002
H          0.558047       1.910279        1.393406
H          0.221645       0.315060        2.082178
```

4_3methyl2butenal_HEI_8_reopt

Datum	Value
M06-2X/def2tzvpp-IEFPCM(water) Energy	-708.752908
M06-2X/def2tzvpp-IEFPCM(water) Free Energy (Quasiharmonic)	-708.626869
Number of Imaginary Frequencies	0

Frequencies (Top 3 out of 51)

```
1.      51.9651 cm-1
2.      58.6345 cm-1
3.     152.8767 cm-1
```

M06-2X/def2tzvpp-IEFPCM(water) Molecular Geometry in Cartesian Coordinates

```
C          2.354399       -0.199083       -0.382324
C          1.294497        0.633089       -0.644030
C         -0.038010        0.632696        0.046419
O          2.474304       -1.139251        0.467820
C         -2.517317       -0.876759        0.261808
H         -2.355335       -1.139185        1.305049
H         -3.053819        0.068167        0.200606
H         -3.128295       -1.650066       -0.200760
S         -0.961827       -0.822356       -0.660967
H          3.233889        0.005645       -1.029494
H          1.414714        1.337842       -1.459492
C         -0.802057        1.910721       -0.291870
H         -0.888236        2.037678       -1.371045
H         -1.803399        1.909696        0.140690
H         -0.262878        2.770353        0.113556
C          0.065093        0.487284        1.563172
H          0.602629       -0.420217        1.823661
H          0.617618        1.342104        1.961051
H         -0.921720        0.482001        2.030048
```

4_3methyl2butenal_TS_1_reopt

Datum	Value

Datum	Value
M06-2X/def2tzvpp-IEFPCM(water) Energy	-708.741325
M06-2X/def2tzvpp-IEFPCM(water) Free Energy (Quasiharmonic)	-708.616614
Number of Imaginary Frequencies	1

Frequencies (Top 3 out of 51)

```
1.    -208.3234 cm-1
2.      79.4082 cm-1
3.      94.3472 cm-1
```

M06-2X/def2tzvpp-IEFPCM(water) Molecular Geometry in Cartesian Coordinates

```
C          2.091595       -0.312211        0.135513
C          1.111889        0.466904       -0.527016
C         -0.021653        0.987881        0.071106
O          3.118188       -0.776402       -0.380040
C         -0.971028       -2.048610        0.231938
H         -1.146313       -2.945413       -0.361552
H          0.101493       -1.823010        0.177943
H         -1.219216       -2.273275        1.269673
S         -1.898024       -0.635394       -0.399832
H          1.923986       -0.491021        1.211961
H          1.271403        0.647537       -1.585630
C         -0.195263        0.950653        1.569719
H         -0.055401       -0.048159        1.977945
H          0.539595        1.613560        2.037203
H         -1.190431        1.293469        1.841439
C         -0.712364        2.136297       -0.613557
H         -1.751339        2.219963       -0.300768
H         -0.199161        3.064620       -0.340270
H         -0.670794        2.033772       -1.696529
```

4_3methyl2butenal_TS_2_reopt

Datum	Value
M06-2X/def2tzvpp-IEFPCM(water) Energy	-708.741325
M06-2X/def2tzvpp-IEFPCM(water) Free Energy (Quasiharmonic)	-708.616613
Number of Imaginary Frequencies	1

Frequencies (Top 3 out of 51)

```
1.    -208.3351 cm-1
2.      79.3728 cm-1
3.      94.3455 cm-1
```

M06-2X/def2tzvpp-IEFPCM(water) Molecular Geometry in Cartesian Coordinates

```
C          2.091663       -0.312286        0.135415
C          1.111932        0.466829       -0.527074
C         -0.021599        0.987824        0.071105
O          3.118218       -0.776514       -0.380189
C         -0.971242       -2.048662        0.231754
H          0.101265       -1.822867        0.178376
H         -1.219822       -2.273988        1.269250
H         -1.146140       -2.945127       -0.362358
S         -1.898152       -0.635218       -0.399596
H          1.924106       -0.491063        1.211876
H          1.271402        0.647432       -1.585700
C         -0.195077        0.950664        1.569738
H         -0.055212       -0.048139        1.977989
H          0.539847        1.613568        2.037128
H         -1.190209        1.293521        1.841538
C         -0.712272        2.136283       -0.613533
H         -1.751226        2.220028       -0.300696
H         -0.198990        3.064573       -0.340284
H         -0.670764        2.033744       -1.696507
```

4_3methyl2butenal_TS_3_reopt

Datum	Value
M06-2X/def2tzvpp-IEFPCM(water) Energy	-708.73922
M06-2X/def2tzvpp-IEFPCM(water) Free Energy (Quasiharmonic)	-708.61578
Number of Imaginary Frequencies	1

Frequencies (Top 3 out of 51)

```
1.    -210.6124 cm-1
2.      15.7090 cm-1
3.      75.1336 cm-1
```

M06-2X/def2tzvpp-IEFPCM(water) Molecular Geometry in Cartesian Coordinates

```
C          -2.082746       -0.298087       -0.305528
C          -1.090275        0.214245        0.562133
C          -0.001767        0.978383        0.164866
O          -3.060104       -0.981335        0.029789
C           1.153915       -2.059656        0.364790
H           1.619361       -2.226351        1.336527
H           0.079636       -1.911308        0.521262
H           1.284422       -2.960137       -0.234675
S           1.822939       -0.606791       -0.475078
H          -1.963669       -0.057435       -1.376294
H          -1.173355       -0.065800        1.607578
C           0.023787        1.709745       -1.153746
H          -0.352371        1.115031       -1.980366
H          -0.591980        2.611075       -1.067927
H           1.040520        2.018459       -1.391800
C           0.758012        1.688065        1.257615
H           1.738476        2.012467        0.917483
H           0.187671        2.574466        1.556948
H           0.879546        1.052698        2.133423
```

4_3methyl2butenal_TS_4_reopt

Datum	Value
M06-2X/def2tzvpp-IEFPCM(water) Energy	-708.73642
M06-2X/def2tzvpp-IEFPCM(water) Free Energy (Quasiharmonic)	-708.613138
Number of Imaginary Frequencies	1

Frequencies (Top 3 out of 51)

```
1.    -235.2005 cm-1
2.      54.8442 cm-1
3.      67.8513 cm-1
```

M06-2X/def2tzvpp-IEFPCM(water) Molecular Geometry in Cartesian Coordinates

```
C           2.414797       -0.399566        0.257266
C           1.426443        0.231917       -0.529194
C           0.237243        0.756203       -0.041389
```

```
O         3.475591     -0.885893     -0.161149
C        -3.046160     -0.438674      0.135939
H        -3.017866      0.261844      0.973572
H        -3.423375      0.096258     -0.736380
H        -3.763776     -1.220658      0.386268
S        -1.395698     -1.137779     -0.174330
H         2.216121     -0.450810      1.341675
H         1.595122      0.246991     -1.601410
C        -0.534950      1.681387     -0.943164
H        -0.469764      1.368814     -1.983398
H        -1.581974      1.745766     -0.652497
H        -0.100487      2.683847     -0.859069
C         0.028025      1.032606      1.424735
H         0.396037      0.238043      2.066070
H         0.553769      1.957565      1.684158
H        -1.029762      1.180711      1.634321
```

4_3methyl2butenal_TS_5_reopt

Datum	Value
M06-2X/def2tzvpp-IEFPCM(water) Energy	-708.738578
M06-2X/def2tzvpp-IEFPCM(water) Free Energy (Quasiharmonic)	-708.614633
Number of Imaginary Frequencies	1

Frequencies (Top 3 out of 51)

```
  1.    -183.9125 cm-1
  2.      15.1941 cm-1
  3.      80.8369 cm-1
```

M06-2X/def2tzvpp-IEFPCM(water) Molecular Geometry in Cartesian Coordinates

```
C        -1.926815     -0.844339     -0.513329
C        -1.020163      0.169400     -0.932439
C        -0.269408      1.004050     -0.126553
O        -2.236090     -1.180910      0.634706
C         1.350938     -1.910937     -0.296308
H         1.183029     -2.633469      0.502791
H         0.403641     -1.779481     -0.835200
H         2.073488     -2.333670     -0.994178
S         1.887617     -0.307479      0.334513
H        -2.400093     -1.385497     -1.356236
H        -0.866025      0.226371     -2.004484
```

```
C         -0.610177       1.229303       1.321957
H         -0.852743       0.304986       1.831539
H          0.210299       1.730396       1.832316
H         -1.489074       1.883234       1.368299
C          0.422147       2.171688      -0.779098
H          0.748183       1.932921      -1.789560
H         -0.284267       3.007283      -0.833893
H          1.281281       2.498893      -0.196626
```

4_3methyl2butenal_TS_6_reopt

Datum	Value
M06-2X/def2tzvpp-IEFPCM(water) Energy	-708.738577
M06-2X/def2tzvpp-IEFPCM(water) Free Energy (Quasiharmonic)	-708.614637
Number of Imaginary Frequencies	1

Frequencies (Top 3 out of 51)

```
1.    -184.0066 cm-1
2.      15.0399 cm-1
3.      80.8497 cm-1
```

M06-2X/def2tzvpp-IEFPCM(water) Molecular Geometry in Cartesian Coordinates

```
C         -1.927107      -0.843703      -0.513374
C         -1.020134       0.169745      -0.932451
C         -0.269047       1.004064      -0.126532
O         -2.236455      -1.180218       0.634657
C          1.350319      -1.911366      -0.296184
H          0.403252      -1.779689      -0.835421
H          2.072964      -2.334563      -0.993676
H          1.181867      -2.633579       0.503090
S          1.887396      -0.307909       0.334374
H         -2.400595      -1.384663      -1.356290
H         -0.866022       0.226740      -2.004499
C         -0.609659       1.229292       1.322017
H         -0.852255       0.304973       1.831578
H          0.210917       1.730279       1.832324
H         -1.488507       1.883275       1.368483
C          0.422928       2.171492      -0.778998
H          0.748818       1.932702      -1.789497
H         -0.283158       3.007375      -0.833666
H          1.282222       2.498297      -0.196539
```

4_3methyl2butenal_TS_7_reopt

Datum	Value
M06-2X/def2tzvpp-IEFPCM(water) Energy	-708.736359
M06-2X/def2tzvpp-IEFPCM(water) Free Energy (Quasiharmonic)	-708.612899
Number of Imaginary Frequencies	1

Frequencies (Top 3 out of 51)

```
1.    -234.3194 cm-1
2.      56.3730 cm-1
3.      70.7858 cm-1
```

M06-2X/def2tzvpp-IEFPCM(water) Molecular Geometry in Cartesian Coordinates

```
C          2.379981       -0.443584        0.298332
C          1.448510        0.263359       -0.493328
C          0.239670        0.768542       -0.033496
O          3.459222       -0.912547       -0.091423
C         -2.987843       -0.510669        0.320556
H         -2.976073       -0.571307        1.409372
H         -3.187469        0.527297        0.044497
H         -3.822499       -1.111923       -0.041278
S         -1.413399       -1.074476       -0.394456
H          2.113402       -0.576327        1.361197
H          1.682318        0.362942       -1.548630
C         -0.461305        1.776399       -0.903905
H         -0.331282        1.548580       -1.959868
H         -1.524126        1.835636       -0.677598
H         -0.022401        2.761421       -0.709287
C         -0.053240        0.933458        1.435381
H         -1.117498        1.102395        1.590677
H          0.484796        1.813279        1.803699
H          0.246809        0.074976        2.028659
```

4_3methyl2butenal_TS_8_reopt

Datum	Value

Datum	Value
M06-2X/def2tzvpp-IEFPCM(water) Energy	-708.73459
M06-2X/def2tzvpp-IEFPCM(water) Free Energy (Quasiharmonic)	-708.611249
Number of Imaginary Frequencies	1

Frequencies (Top 3 out of 51)

```
1.    -233.8710 cm-1
2.      56.1389 cm-1
3.      71.9870 cm-1
```

M06-2X/def2tzvpp-IEFPCM(water) Molecular Geometry in Cartesian Coordinates

```
C          2.460041       -0.478915       -0.427609
C          1.473430        0.503226       -0.704775
C          0.361564        0.813677        0.062156
O          2.530718       -1.261383        0.527791
C         -2.805428       -0.594268        0.441923
H         -2.625700       -1.093167        1.394839
H         -2.954437        0.469217        0.644501
H         -3.737396       -0.981718        0.029072
S         -1.421876       -0.840393       -0.712099
H          3.258363       -0.528627       -1.193891
H          1.582530        1.006093       -1.659086
C         -0.413709        2.047886       -0.309448
H         -0.416895        2.210610       -1.385121
H         -1.440156        1.998431        0.048883
H          0.064432        2.910786        0.167443
C          0.261918        0.443797        1.515293
H          0.623329       -0.559328        1.705812
H          0.885801        1.143614        2.084101
H         -0.762496        0.549022        1.869451
```

5_trans-2-methyl-2-butenal_1

Datum	Value
M06-2X/def2tzvpp-IEFPCM(water) Energy	-270.538087
M06-2X/def2tzvpp-IEFPCM(water) Free Energy (Quasiharmonic)	-270.447305
Number of Imaginary Frequencies	0

Frequencies (Top 3 out of 36)

```
1.     108.7589 cm-1
2.     122.1360 cm-1
3.     131.1117 cm-1
```

M06-2X/def2tzvpp-IEFPCM(water) Molecular Geometry in Cartesian Coordinates

```
C          1.312579       -0.734221        0.000069
C          0.062951        0.040129       -0.000018
O          2.418535       -0.241163        0.000094
C         -1.069661       -0.673486       -0.000004
C         -2.470035       -0.169541       -0.000047
H          1.189450       -1.831499        0.000115
H         -0.965311       -1.755549        0.000062
C          0.198028        1.530427       -0.000085
H         -3.000560       -0.550929        0.874504
H         -2.533251        0.914581       -0.000286
H         -3.000664       -0.551334       -0.874356
H         -0.764555        2.032662       -0.000412
H          0.761448        1.855832        0.875341
H          0.761987        1.855698       -0.875211
```

5_trans-2-methyl-2-butenal_2

Datum	Value
M06-2X/def2tzvpp-IEFPCM(water) Energy	-270.532018
M06-2X/def2tzvpp-IEFPCM(water) Free Energy (Quasiharmonic)	-270.442103
Number of Imaginary Frequencies	0

Frequencies (Top 3 out of 36)

```
1.      89.6715 cm-1
2.     106.4637 cm-1
3.     123.8075 cm-1
```

M06-2X/def2tzvpp-IEFPCM(water) Molecular Geometry in Cartesian Coordinates

```
C          1.553772        0.082458        0.000067
C          0.091055        0.335848       -0.000016
O          2.082802       -1.004616        0.000135
C         -0.725378       -0.721320       -0.000029
C         -2.214258       -0.728253       -0.000082
H          2.179440        0.993649        0.000063
H         -0.246130       -1.695813        0.000016
C         -0.305178        1.783055       -0.000057
H         -2.582505       -1.268902        0.874134
H         -2.648686        0.267239       -0.000231
H         -2.582452       -1.269155       -0.874163
H         -1.382445        1.917659       -0.000488
H          0.099788        2.290694        0.877460
H          0.100505        2.290829       -0.877164
```

5_trans2methyl2butenal_HEI_1_reopt

Datum	Value
M06-2X/def2tzvpp-IEFPCM(water) Energy	-708.75467
M06-2X/def2tzvpp-IEFPCM(water) Free Energy (Quasiharmonic)	-708.628993
Number of Imaginary Frequencies	0

Frequencies (Top 3 out of 51)

```
1.      49.7297 cm-1
2.      78.5463 cm-1
3.     137.5390 cm-1
```

M06-2X/def2tzvpp-IEFPCM(water) Molecular Geometry in Cartesian Coordinates

```
C         -1.966139       -0.379978       -0.057541
C         -0.917825        0.433351        0.286242
C          0.250223        0.555189       -0.625402
O         -2.140427       -1.080491       -1.115304
C          0.988574       -1.920886        0.481498
H          0.314174       -1.764023        1.320847
H          0.425766       -2.340865       -0.349694
H          1.784834       -2.604079        0.769569
S          1.751513       -0.364454       -0.005888
H         -2.772455       -0.415382        0.706112
C          0.729689        1.980888       -0.880167
H          1.028273        2.466672        0.049599
```

```
H          1.583097        2.000219       -1.560383
H         -0.077354        2.565369       -1.324305
H         -0.005596        0.078322       -1.570821
C         -0.859001        1.105516        1.626966
H         -0.000104        0.764378        2.219855
H         -0.773692        2.195987        1.564806
H         -1.760870        0.884114        2.201471
```

5_trans2methyl2butenal_HEI_2

Datum	Value
M06-2X/def2tzvpp-IEFPCM(water) Energy	-708.755606
M06-2X/def2tzvpp-IEFPCM(water) Free Energy (Quasiharmonic)	-708.630275
Number of Imaginary Frequencies	0

Frequencies (Top 3 out of 51)

```
1.      38.4957 cm-1
2.      77.8926 cm-1
3.     133.7383 cm-1
```

M06-2X/def2tzvpp-IEFPCM(water) Molecular Geometry in Cartesian Coordinates

```
C         -1.774222       -0.511772       -0.610394
C         -0.887792        0.332741        0.006332
C          0.440680        0.586473       -0.604037
O         -2.948217       -0.860627       -0.241155
C          1.294382       -1.991993        0.096364
H          0.299594       -2.100277        0.524749
H          1.267726       -2.285251       -0.952587
H          1.995657       -2.630564        0.629004
S          1.850526       -0.289962        0.268825
H         -1.398882       -0.936362       -1.566192
C          0.856378        2.054748       -0.637922
H          0.926672        2.461099        0.372271
H          1.823104        2.187280       -1.125031
H          0.108930        2.634578       -1.181286
H          0.458059        0.179784       -1.617678
C         -1.202669        0.966826        1.330503
H         -2.114482        0.531950        1.739099
H         -1.354397        2.050313        1.266012
H         -0.395209        0.809719        2.054604
```

5_trans2methyl2butenal_HEI_3

Datum	Value
M06-2X/def2tzvpp-IEFPCM(water) Energy	-708.753544
M06-2X/def2tzvpp-IEFPCM(water) Free Energy (Quasiharmonic)	-708.627407
Number of Imaginary Frequencies	0

Frequencies (Top 3 out of 51)

```
1.      61.1096 cm-1
2.      78.9349 cm-1
3.     138.7183 cm-1
```

M06-2X/def2tzvpp-IEFPCM(water) Molecular Geometry in Cartesian Coordinates

```
C          2.134114       -0.724772        0.012877
C          1.244096        0.312284       -0.070909
C         -0.116269        0.165993        0.530429
O          1.985120       -1.872337        0.561244
C         -2.835710       -0.563495        0.076362
H         -2.687770       -1.141873        0.987893
H         -3.272566        0.401139        0.324549
H         -3.520890       -1.103671       -0.573744
S         -1.268695       -0.387593       -0.808042
H          3.115885       -0.515533       -0.462081
C         -0.643843        1.423093        1.209495
H         -0.766049        2.235003        0.491522
H         -1.604641        1.249330        1.693689
H          0.064581        1.748740        1.974464
H         -0.091515       -0.659610        1.242900
C          1.577864        1.584400       -0.798099
H          0.871766        1.801529       -1.608872
H          1.575849        2.467047       -0.148436
H          2.572001        1.513062       -1.244088
```

5_trans2methyl2butenal_HEI_4

Datum	Value
M06-2X/def2tzvpp-IEFPCM(water) Energy	-708.752508

Datum	Value
M06-2X/def2tzvpp-IEFPCM(water) Free Energy (Quasiharmonic)	-708.626562
Number of Imaginary Frequencies	0

Frequencies (Top 3 out of 51)

```
1.      61.6042 cm-1
2.      79.1990 cm-1
3.     117.2525 cm-1
```

M06-2X/def2tzvpp-IEFPCM(water) Molecular Geometry in Cartesian Coordinates

```
C         -1.781358        0.456369        0.452595
C         -0.876732       -0.013469       -0.461942
C          0.432137        0.646806       -0.727101
O         -2.906596       -0.059182        0.792638
C          1.357067       -0.856653        1.471620
H          1.219701        0.033573        2.083290
H          0.424894       -1.417736        1.440567
H          2.142601       -1.469410        1.908870
S          1.877813       -0.426175       -0.196521
H         -1.506426        1.405342        0.947070
C          0.630468        2.046913       -0.159417
H          0.644716        2.040007        0.930625
H          1.575867        2.461225       -0.506107
H         -0.177997        2.706915       -0.478436
H          0.615808        0.683543       -1.806741
C         -1.123142       -1.310021       -1.177521
H         -2.180678       -1.568771       -1.137480
H         -0.561834       -2.145890       -0.739241
H         -0.819537       -1.256221       -2.228584
```

5_trans2methyl2butenal_HEI_5

Datum	Value
M06-2X/def2tzvpp-IEFPCM(water) Energy	-708.748893
M06-2X/def2tzvpp-IEFPCM(water) Free Energy (Quasiharmonic)	-708.62345
Number of Imaginary Frequencies	0

Frequencies (Top 3 out of 51)

```
1.      54.5171 cm-1
2.      69.8699 cm-1
3.      99.2142 cm-1
```

M06-2X/def2tzvpp-IEFPCM(water) Molecular Geometry in Cartesian Coordinates

```
C          1.637405        0.250749       -0.753187
C          0.891074        0.033912        0.372258
C         -0.439433        0.691751        0.575118
O          2.772639       -0.247855       -1.080102
C         -1.558638       -1.772329       -0.226209
H         -0.540973       -2.024726       -0.520272
H         -1.723307       -2.063836        0.810209
H         -2.264076       -2.302601       -0.862190
S         -1.830701       -0.009285       -0.457280
H          1.179003        0.953610       -1.477832
C         -0.494953        2.198555        0.329992
H         -0.220973        2.441615       -0.695755
H         -1.495112        2.595015        0.516792
H          0.205877        2.701690        0.996486
H         -0.755354        0.507734        1.604591
C          1.348121       -0.924734        1.437965
H          2.344582       -1.296278        1.200403
H          0.686042       -1.792862        1.541901
H          1.392949       -0.455379        2.427343
```

5_trans2methyl2butenal_HEI_6_reopt

Datum	Value
M06-2X/def2tzvpp-IEFPCM(water) Energy	-708.750557
M06-2X/def2tzvpp-IEFPCM(water) Free Energy (Quasiharmonic)	-708.624244
Number of Imaginary Frequencies	0

Frequencies (Top 3 out of 51)

```
1.      67.5161 cm-1
2.      83.0977 cm-1
3.     130.5373 cm-1
```

M06-2X/def2tzvpp-IEFPCM(water) Molecular Geometry in Cartesian Coordinates

```
C          -2.196706       0.327898       -0.469861
C          -1.024092       0.011249        0.162884
C           0.196647       0.827939       -0.089269
O          -3.333608      -0.258707       -0.406329
C           2.499878      -0.878875        0.242599
H           3.053201      -0.151579        0.832626
H           1.874132      -1.480864        0.897586
H           3.208205      -1.530831       -0.264737
S           1.521251      -0.079149       -1.048076
H          -2.130345       1.228072       -1.117855
C           0.815477       1.469664        1.151191
H           1.080423       0.723099        1.899654
H           1.714945       2.037101        0.907540
H           0.086483       2.145022        1.600899
H          -0.063244       1.623931       -0.791061
C          -0.922641      -1.201984        1.041717
H          -1.921395      -1.575252        1.267462
H          -0.416440      -0.999458        1.990963
H          -0.368500      -2.018541        0.561207
```

5_trans2methyl2butenal_HEI_7_reopt

Datum	Value
M06-2X/def2tzvpp-IEFPCM(water) Energy	-708.75341
M06-2X/def2tzvpp-IEFPCM(water) Free Energy (Quasiharmonic)	-708.628124
Number of Imaginary Frequencies	0

Frequencies (Top 3 out of 51)

```
  1.      51.6769 cm-1
  2.      57.0751 cm-1
  3.     123.8825 cm-1
```

M06-2X/def2tzvpp-IEFPCM(water) Molecular Geometry in Cartesian Coordinates

```
C          -2.027540      -0.677132       -0.576330
C          -1.114180       0.175088       -0.015000
C           0.277390       0.232479       -0.553080
```

```
O          -3.253400       -0.867572       -0.264610
C           3.019610       -0.501381       -0.159930
H           2.973240       -0.710641       -1.228280
H           3.408360        0.502059       -0.002090
H           3.692601       -1.217451        0.307300
S           1.389090       -0.720631        0.589970
H          -1.623189       -1.274982       -1.421400
C           0.810560        1.646769       -0.752380
H           0.862630        2.178579        0.198590
H           1.803800        1.652329       -1.200550
H           0.137810        2.195858       -1.414440
H           0.319580       -0.306841       -1.502080
C          -1.473910        1.055788        1.149110
H          -2.443620        0.756368        1.546630
H          -1.541720        2.116628        0.880720
H          -0.739330        0.989108        1.958590
```

5_trans2methyl2butenal_TS_1_reopt

Datum	Value
M06-2X/def2tzvpp-IEFPCM(water) Energy	-708.741157
M06-2X/def2tzvpp-IEFPCM(water) Free Energy (Quasiharmonic)	-708.617051
Number of Imaginary Frequencies	1

Frequencies (Top 3 out of 51)

```
1.    -175.5889 cm-1
2.      65.7512 cm-1
3.      83.2168 cm-1
```

M06-2X/def2tzvpp-IEFPCM(water) Molecular Geometry in Cartesian Coordinates

```
C          -1.694488       -1.053723       -0.181189
C          -1.151531        0.221389        0.175749
C          -0.304855        0.847739       -0.704733
O          -1.509672       -1.674754       -1.231438
C           1.508555       -1.420185        0.875834
H           0.422187       -1.361265        1.025743
H           1.732088       -2.398185        0.449306
H           1.980204       -1.355258        1.856371
S           2.028354       -0.071550       -0.203928
H          -2.352611       -1.505440        0.587545
C           0.072996        2.291358       -0.578868
```

```
H         0.302908     2.558297     0.451069
H         0.933356     2.532830    -1.199176
H        -0.767312     2.913108    -0.906522
H        -0.235153     0.414915    -1.693015
C        -1.419583     0.777002     1.546712
H        -0.491033     0.881056     2.118013
H        -1.886144     1.764090     1.514308
H        -2.081337     0.117207     2.109668
```

5_trans2methyl2butenal_TS_2_reopt

Datum	Value
M06-2X/def2tzvpp-IEFPCM(water) Energy	-708.743966
M06-2X/def2tzvpp-IEFPCM(water) Free Energy (Quasiharmonic)	-708.62048
Number of Imaginary Frequencies	1

Frequencies (Top 3 out of 51)

```
1.    -192.0929 cm-1
2.      35.8207 cm-1
3.      89.8274 cm-1
```

M06-2X/def2tzvpp-IEFPCM(water) Molecular Geometry in Cartesian Coordinates

```
C        -1.748642    -0.581553    -0.696017
C        -0.986565     0.421397    -0.036054
C         0.131712     0.911033    -0.681325
O        -2.786421    -1.120090    -0.291930
C         1.252896    -1.941056     0.329144
H         0.173078    -1.749339     0.310392
H         1.477682    -2.688419    -0.431553
H         1.496576    -2.360762     1.304726
S         2.118577    -0.385200     0.038950
H        -1.349378    -0.873400    -1.687297
C         0.732139     2.241667    -0.336659
H         0.828724     2.367425     0.740173
H         1.713845     2.364606    -0.789343
H         0.080024     3.038133    -0.710105
H         0.278897     0.582805    -1.703368
C        -1.367263     0.866531     1.345718
H        -2.244350     0.318339     1.685434
H        -1.594892     1.933991     1.392018
H        -0.551729     0.682424     2.052326
```

5_trans2methyl2butenal_TS_3

Datum	Value
M06-2X/def2tzvpp-IEFPCM(water) Energy	-708.735535
M06-2X/def2tzvpp-IEFPCM(water) Free Energy (Quasiharmonic)	-708.61188
Number of Imaginary Frequencies	1

Frequencies (Top 3 out of 51)

```
1.    -227.2017 cm-1
2.      58.2972 cm-1
3.      91.5900 cm-1
```

M06-2X/def2tzvpp-IEFPCM(water) Molecular Geometry in Cartesian Coordinates

```
C          2.052977       -1.048834        0.057882
C          1.426713        0.229584        0.014287
C          0.185144        0.387618        0.596879
O          1.605236       -2.088321        0.556909
C         -2.894495       -0.386823        0.407078
H         -2.401418       -0.506307        1.376208
H         -3.484031        0.529389        0.444165
H         -3.578678       -1.225252        0.280797
S         -1.657160       -0.343433       -0.922422
H          3.056040       -1.085955       -0.411937
C         -0.371455        1.748829        0.886093
H         -0.297986        2.401078        0.017477
H         -1.410782        1.702945        1.201856
H          0.211091        2.206458        1.693654
H         -0.154246       -0.421123        1.232240
C          2.089537        1.347719       -0.742545
H          1.498441        1.653766       -1.612303
H          2.234124        2.240317       -0.129235
H          3.069605        1.037622       -1.107493
```

5_trans2methyl2butenal_TS_4

Datum	Value

Datum	Value
M06-2X/def2tzvpp-IEFPCM(water) Energy	-708.74078
M06-2X/def2tzvpp-IEFPCM(water) Free Energy (Quasiharmonic)	-708.616445
Number of Imaginary Frequencies	1

Frequencies (Top 3 out of 51)

```
1.    -212.8168 cm-1
2.      57.4163 cm-1
3.      91.6501 cm-1
```

M06-2X/def2tzvpp-IEFPCM(water) Molecular Geometry in Cartesian Coordinates

```
C        -1.737901        0.254599        0.703929
C        -1.009257        0.124744       -0.505812
C         0.112019        0.895936       -0.758796
O        -2.733113       -0.415269        1.023959
C         1.525329       -0.908701        1.346019
H         2.023265       -0.309790        2.108348
H         0.445649       -0.717752        1.410195
H         1.686073       -1.960895        1.577672
S         2.092472       -0.501718       -0.317114
H        -1.397138        1.044234        1.394638
C         0.518383        2.077742        0.082723
H         0.665116        1.800462        1.125964
H         1.449868        2.499808       -0.284303
H        -0.251911        2.852821        0.048685
H         0.448710        0.930409       -1.786922
C        -1.424103       -0.954080       -1.464280
H        -2.502980       -0.953637       -1.627475
H        -1.162123       -1.945971       -1.081286
H        -0.926004       -0.831483       -2.426061
```

5_trans2methyl2butenal_TS_5_reopt

Datum	Value
M06-2X/def2tzvpp-IEFPCM(water) Energy	-708.74078
M06-2X/def2tzvpp-IEFPCM(water) Free Energy (Quasiharmonic)	-708.616444
Number of Imaginary Frequencies	1

Frequencies (Top 3 out of 51)

```
1.    -212.7943 cm-1
2.      57.5735 cm-1
3.      91.6194 cm-1
```

M06-2X/def2tzvpp-IEFPCM(water) Molecular Geometry in Cartesian Coordinates

```
C          1.737903        0.254355       -0.703985
C          1.009235        0.124966        0.505798
C         -0.112040        0.896269        0.758464
O          2.733113       -0.415650       -1.023743
C         -1.525164       -0.909456       -1.345618
H         -0.445466       -0.718597       -1.409749
H         -1.685927       -1.961774       -1.576694
H         -2.022968       -0.310959       -2.108359
S         -2.092503       -0.501498        0.317210
H          1.397168        1.043733       -1.394997
C         -0.518374        2.077756       -0.083515
H         -0.665107        1.800070       -1.126647
H         -1.449859        2.499975        0.283337
H          0.251924        2.852844       -0.049775
H         -0.448765        0.931131        1.786566
C          1.424004       -0.953553        1.464640
H          2.502871       -0.953063        1.627910
H          1.162049       -1.945564        1.081939
H          0.925836       -0.830645        2.426344
```

5_trans2methyl2butenal_TS_6_reopt

Datum	Value
M06-2X/def2tzvpp-IEFPCM(water) Energy	-708.737389
M06-2X/def2tzvpp-IEFPCM(water) Free Energy (Quasiharmonic)	-708.614079
Number of Imaginary Frequencies	1

Frequencies (Top 3 out of 51)

```
1.    -245.0999 cm-1
2.      27.2601 cm-1
3.      83.4120 cm-1
```

M06-2X/def2tzvpp-IEFPCM(water) Molecular Geometry in Cartesian Coordinates

```
C          -2.180592       -0.658954       -0.387013
C          -1.201631        0.309540       -0.072426
C          -0.074102        0.376043       -0.882087
O          -3.232565       -0.898790        0.227050
C           2.855281       -0.055067        0.774371
H           3.289270        0.677063        0.093156
H           2.385326        0.484039        1.597251
H           3.667978       -0.653783        1.185777
S           1.662137       -1.128573       -0.081330
H          -1.957627       -1.247693       -1.299039
C           0.777934        1.609572       -0.931812
H           1.085817        1.927789        0.063633
H           1.668954        1.458154       -1.536478
H           0.193052        2.425382       -1.368936
H          -0.111473       -0.198770       -1.800164
C          -1.324810        1.139001        1.172093
H          -2.282927        0.949801        1.653282
H          -1.251729        2.209838        0.968919
H          -0.532797        0.894856        1.888724
```

5_trans2methyl2butenal_TS_7_reopt

Datum	Value
M06-2X/def2tzvpp-IEFPCM(water) Energy	-708.738142
M06-2X/def2tzvpp-IEFPCM(water) Free Energy (Quasiharmonic)	-708.61449
Number of Imaginary Frequencies	2

Frequencies (Top 3 out of 51)

```
  1.    -231.7576 cm-1
  2.     -11.7553 cm-1
  3.      66.1223 cm-1
```

M06-2X/def2tzvpp-IEFPCM(water) Molecular Geometry in Cartesian Coordinates

```
C          -2.116060       -0.727250       -0.549130
C          -1.262524        0.297543       -0.073546
C          -0.056736        0.484771       -0.731428
```

```
O          -3.218829       -1.061321       -0.093032
C           3.251550       -0.275331        0.066532
H           3.484491       -0.212885       -0.996933
H           3.327352        0.726627        0.490139
H           4.012211       -0.897289        0.539196
S           1.592900       -0.975621        0.340400
H          -1.727288       -1.263633       -1.437337
C           0.722404        1.757877       -0.600270
H           0.853920        2.036255        0.444561
H           1.701975        1.682185       -1.066171
H           0.170176        2.564942       -1.093595
H           0.069551       -0.049570       -1.665008
C          -1.620189        1.076249        1.158845
H          -2.582476        0.742833        1.544116
H          -1.684580        2.150206        0.967422
H          -0.871768        0.937686        1.945444
```

6_2ethylacrolein_1

Datum	Value
M06-2X/def2tzvpp-IEFPCM(water) Energy	-270.531295
M06-2X/def2tzvpp-IEFPCM(water) Free Energy (Quasiharmonic)	-270.439235
Number of Imaginary Frequencies	0

Frequencies (Top 3 out of 36)

```
1.      95.3467 cm-1
2.     170.7274 cm-1
3.     207.4475 cm-1
```

M06-2X/def2tzvpp-IEFPCM(water) Molecular Geometry in Cartesian Coordinates

```
C           1.310085       -0.452009       -0.129494
O           1.149757       -1.627067        0.102718
C           0.290050        0.588786        0.117403
C           0.638347        1.846849       -0.147302
C          -1.050992        0.154693        0.628937
C          -1.895121       -0.494931       -0.469810
H           2.265584       -0.085257       -0.543169
H          -0.040331        2.673703        0.018235
H           1.621695        2.081105       -0.537450
H          -0.903122       -0.560239        1.440170
H          -1.573829        1.018200        1.039448
```

```
H          -2.852440       -0.828771       -0.071676
H          -2.087730        0.215610       -1.273634
H          -1.382094       -1.358145       -0.892075
```

6_2ethylacrolein_2

Datum	Value
M06-2X/def2tzvpp-IEFPCM(water) Energy	-270.531295
M06-2X/def2tzvpp-IEFPCM(water) Free Energy (Quasiharmonic)	-270.439235
Number of Imaginary Frequencies	0

Frequencies (Top 3 out of 36)

```
  1.      95.3453 cm-1
  2.     170.7257 cm-1
  3.     207.4476 cm-1
```

M06-2X/def2tzvpp-IEFPCM(water) Molecular Geometry in Cartesian Coordinates

```
C          -1.310088       -0.452001       -0.129495
O          -1.149770       -1.627060        0.102718
C          -0.290046        0.588787        0.117404
C          -0.638334        1.846852       -0.147302
C           1.050993        0.154684        0.628938
C           1.895119       -0.494939       -0.469811
H          -2.265583       -0.085241       -0.543173
H           0.040347        2.673702        0.018238
H          -1.621681        2.081113       -0.537451
H           1.573834        1.018188        1.039453
H           0.903117       -0.560251        1.440168
H           2.852433       -0.828794       -0.071675
H           1.382084       -1.358144       -0.892087
H           2.087740        0.215607       -1.273627
```

6_2ethylacrolein_3

Datum	Value
M06-2X/def2tzvpp-IEFPCM(water) Energy	-270.532199

Datum	Value
M06-2X/def2tzvpp-IEFPCM(water) Free Energy (Quasiharmonic)	-270.440101
Number of Imaginary Frequencies	0

Frequencies (Top 3 out of 36)

```
1.      94.6321 cm-1
2.     171.2586 cm-1
3.     215.7651 cm-1
```

M06-2X/def2tzvpp-IEFPCM(water) Molecular Geometry in Cartesian Coordinates

```
C          1.590656        0.175891       -0.000000
O          2.097329       -0.920862        0.000002
C          0.131847        0.412032       -0.000007
C         -0.267919        1.682388        0.000002
C         -0.755735       -0.796686       -0.000002
C         -2.244360       -0.490955        0.000002
H          2.216190        1.084892        0.000004
H         -1.312391        1.962621        0.000004
H          0.458243        2.486371        0.000001
H         -0.493861       -1.407551       -0.867749
H         -0.493855       -1.407542        0.867750
H         -2.529538        0.083239        0.882147
H         -2.529536        0.083260       -0.882131
H         -2.820809       -1.414415       -0.000010
```

6_2ethylacrolein_4

Datum	Value
M06-2X/def2tzvpp-IEFPCM(water) Energy	-270.526283
M06-2X/def2tzvpp-IEFPCM(water) Free Energy (Quasiharmonic)	-270.434897
Number of Imaginary Frequencies	0

Frequencies (Top 3 out of 36)

```
1.      68.4703 cm-1
2.     124.2051 cm-1
3.     210.5525 cm-1
```

M06-2X/def2tzvpp-IEFPCM(water) Molecular Geometry in Cartesian Coordinates

```
C          1.027082      -0.745879       0.216094
O          2.167950      -0.756447      -0.177122
C          0.118369       0.434282       0.171922
C          0.607805       1.608760      -0.213605
C         -1.307818       0.186805       0.570835
C         -2.060129      -0.618122      -0.490717
H          0.588447      -1.657597       0.660228
H         -0.011331       2.495876      -0.245796
H          1.647133       1.702382      -0.500829
H         -1.325064      -0.359357       1.517818
H         -1.807014       1.140026       0.741241
H         -3.080110      -0.824678      -0.169759
H         -2.097941      -0.066494      -1.429908
H         -1.569559      -1.573666      -0.683197
```

6_2ethylacrolein_5

Datum	Value
M06-2X/def2tzvpp-IEFPCM(water) Energy	-270.526283
M06-2X/def2tzvpp-IEFPCM(water) Free Energy (Quasiharmonic)	-270.434897
Number of Imaginary Frequencies	0

Frequencies (Top 3 out of 36)

```
1.      68.4702 cm-1
2.     124.2054 cm-1
3.     210.5523 cm-1
```

M06-2X/def2tzvpp-IEFPCM(water) Molecular Geometry in Cartesian Coordinates

```
C         -1.027084      -0.745878       0.216094
O         -2.167951      -0.756444      -0.177124
C         -0.118368       0.434281       0.171924
C         -0.607802       1.608760      -0.213604
C          1.307818       0.186801       0.570837
C          2.060130      -0.618120      -0.490719
H         -0.588452      -1.657596       0.660231
```

```
H          0.011336       2.495875      -0.245794
H         -1.647130       1.702384      -0.500829
H          1.807014       1.140020       0.741249
H          1.325063      -0.359366       1.517816
H          3.080109      -0.824682      -0.169759
H          1.569558      -1.573662      -0.683207
H          2.097946      -0.066485      -1.429906
```

6_2ethylacrolein_6

Datum	Value
M06-2X/def2tzvpp-IEFPCM(water) Energy	-270.527037
M06-2X/def2tzvpp-IEFPCM(water) Free Energy (Quasiharmonic)	-270.435495
Number of Imaginary Frequencies	0

Frequencies (Top 3 out of 36)

```
1.     73.1261 cm-1
2.    112.3255 cm-1
3.    232.7511 cm-1
```

M06-2X/def2tzvpp-IEFPCM(water) Molecular Geometry in Cartesian Coordinates

```
C         -1.419323      -0.616328       0.000000
O         -2.504471      -0.088817       0.000000
C         -0.115308       0.106272      -0.000000
C         -0.122566       1.435613      -0.000000
C          1.105552      -0.770304      -0.000000
C          2.427074      -0.021733       0.000000
H         -1.341727      -1.718465      -0.000000
H          0.788940       2.017154      -0.000000
H         -1.063328       1.970431       0.000000
H          1.051039      -1.432506       0.869684
H          1.051039      -1.432505      -0.869685
H          3.261081      -0.721128       0.000000
H          2.518073       0.613222      -0.881867
H          2.518072       0.613221       0.881868
```

6_2ethylacrolein_HEI_10_reopt

Datum	Value
M06-2X/def2tzvpp-IEFPCM(water) Energy	-708.746647
M06-2X/def2tzvpp-IEFPCM(water) Free Energy (Quasiharmonic)	-708.62038
Number of Imaginary Frequencies	0

Frequencies (Top 3 out of 51)

```
1.      42.3216 cm-1
2.      62.2812 cm-1
3.      88.3811 cm-1
```

M06-2X/def2tzvpp-IEFPCM(water) Molecular Geometry in Cartesian Coordinates

```
C          1.245352       -1.370579       -0.224901
O          2.355402       -1.522765       -0.846384
C          0.771041       -0.279661        0.450207
C         -0.557408       -0.331083        1.146137
C          1.605645        0.964483        0.610315
C          1.145558        2.176480       -0.204059
H          0.548922       -2.236546       -0.223974
H         -0.720487        0.588273        1.712314
H         -0.645030       -1.156035        1.858595
H          1.639905        1.261217        1.666238
H          2.630564        0.717977        0.326262
H          1.791366        3.041198       -0.035921
H          1.153412        1.947523       -1.271208
H          0.127611        2.465482        0.063724
C         -1.954456        0.801144       -1.016816
H         -2.101609        1.746911       -0.497779
H         -0.979060        0.797520       -1.500881
H         -2.732648        0.680227       -1.767228
S         -2.068160       -0.589771        0.117227
```

6_2ethylacrolein_HEI_11_reopt

Datum	Value
M06-2X/def2tzvpp-IEFPCM(water) Energy	-708.74867
M06-2X/def2tzvpp-IEFPCM(water) Free Energy (Quasiharmonic)	-708.622145
Number of Imaginary Frequencies	0

Frequencies (Top 3 out of 51)

```
1.      66.0674 cm-1
2.      71.2197 cm-1
3.     135.5803 cm-1
```

M06-2X/def2tzvpp-IEFPCM(water) Molecular Geometry in Cartesian Coordinates

```
C         -2.220021       -0.244154       -0.037067
O         -2.672552       -1.411048       -0.305459
C         -0.971236        0.130061        0.386757
C          0.050472       -0.912178        0.657970
C         -0.628685        1.584254        0.582511
C         -0.078893        2.293390       -0.662919
H         -2.926331        0.607624       -0.146712
H         -0.418759       -1.893701        0.605417
H          0.514184       -0.806808        1.644030
H         -1.520244        2.129721        0.908182
H          0.098469        1.685120        1.397349
H          0.217207        3.322908       -0.447068
H         -0.843259        2.316994       -1.441761
H          0.782784        1.765529       -1.072225
C          2.647731        0.100686        0.315483
H          2.253956        1.115095        0.340262
H          2.853590       -0.232938        1.331383
H          3.575090        0.094465       -0.253402
S          1.499845       -1.032999       -0.492387
```

6_2ethylacrolein_HEI_1_reopt

Datum	Value
M06-2X/def2tzvpp-IEFPCM(water) Energy	-708.751352
M06-2X/def2tzvpp-IEFPCM(water) Free Energy (Quasiharmonic)	-708.624592
Number of Imaginary Frequencies	0

Frequencies (Top 3 out of 51)

```
1.      58.1745 cm-1
2.      66.7213 cm-1
3.      92.2488 cm-1
```

M06-2X/def2tzvpp-IEFPCM(water) Molecular Geometry in Cartesian Coordinates

```
C         -1.048424      1.364741     -0.025295
O         -0.392727      2.455703     -0.197420
C         -0.708911      0.093848     -0.407164
C          0.568339     -0.137604     -1.129254
C         -1.537110     -1.134650     -0.128642
C         -2.849506     -0.917888      0.615232
H         -2.022344      1.452219      0.489984
H          0.969347      0.797130     -1.517471
H          0.458483     -0.844300     -1.956833
H         -1.758737     -1.647699     -1.075107
H         -0.935260     -1.854805      0.441474
H         -3.367492     -1.866461      0.760014
H         -2.681124     -0.474212      1.597634
H         -3.515806     -0.252349      0.064276
C          2.087252      0.363716      1.144394
H          2.580273      1.245851      0.740265
H          1.097408      0.636766      1.507906
H          2.681924     -0.042773      1.959300
S          1.910332     -0.911748     -0.115482
```

6_2ethylacrolein_HEI_2

Datum	Value
M06-2X/def2tzvpp-IEFPCM(water) Energy	-708.753478
M06-2X/def2tzvpp-IEFPCM(water) Free Energy (Quasiharmonic)	-708.627384
Number of Imaginary Frequencies	0

Frequencies (Top 3 out of 51)

```
1.     25.3831 cm-1
2.     69.2392 cm-1
3.     87.5653 cm-1
```

M06-2X/def2tzvpp-IEFPCM(water) Molecular Geometry in Cartesian Coordinates

```
C          0.961022     -1.708862      0.182958
O          0.378202     -2.710489     -0.360735
C          0.785315     -0.368017     -0.043610
```

```
C          -0.226749       0.087488       -1.031066
C           1.516751       0.665501        0.765483
C           2.253884       1.717061       -0.067164
H           1.734069      -1.933184        0.948795
H          -0.630311      -0.767622       -1.570720
H           0.161203       0.803270       -1.760230
H           0.822268       1.198033        1.430673
H           2.231917       0.155771        1.417228
H           1.555431       2.291755       -0.678463
H           2.792120       2.424431        0.566098
H           2.972139       1.244017       -0.739709
C          -2.178003      -0.176549        0.944525
H          -2.950930       0.290535        1.550502
H          -1.327562      -0.441543        1.571373
H          -2.577383      -1.076549        0.479795
S          -1.655118       1.012204       -0.302264
```

6_2ethylacrolein_HEI_3_reopt

Datum	Value
M06-2X/def2tzvpp-IEFPCM(water) Energy	-708.751683
M06-2X/def2tzvpp-IEFPCM(water) Free Energy (Quasiharmonic)	-708.625381
Number of Imaginary Frequencies	0

Frequencies (Top 3 out of 51)

```
1.      45.9582 cm-1
2.      75.4815 cm-1
3.      88.5131 cm-1
```

M06-2X/def2tzvpp-IEFPCM(water) Molecular Geometry in Cartesian Coordinates

```
C          -1.611886      -1.087776        0.191875
O          -1.312601      -2.323316        0.332857
C          -0.929534      -0.092325       -0.458852
C           0.327316      -0.414294       -1.177766
C          -1.434512       1.327145       -0.437201
C          -0.911899       2.185381        0.723039
H          -2.568461      -0.747324        0.645595
H           0.371261       0.041078       -2.171048
H           0.427988      -1.493512       -1.282735
H          -1.178668       1.823934       -1.380518
H          -2.527964       1.322172       -0.386019
```

```
H          -1.224148       1.753233       1.675813
H          -1.294362       3.207873       0.673655
H           0.177502       2.225131       0.715816
C           1.701815      -0.432607       1.261715
H           0.702953      -0.175859       1.614670
H           1.833008      -1.512591       1.295462
H           2.448529       0.041488       1.894881
S           1.905336       0.199235      -0.410956
```

6_2ethylacrolein_HEI_4_reopt

Datum	Value
M06-2X/def2tzvpp-IEFPCM(water) Energy	-708.74784
M06-2X/def2tzvpp-IEFPCM(water) Free Energy (Quasiharmonic)	-708.621306
Number of Imaginary Frequencies	0

Frequencies (Top 3 out of 51)

```
1.      51.6127 cm-1
2.      68.7305 cm-1
3.     118.6205 cm-1
```

M06-2X/def2tzvpp-IEFPCM(water) Molecular Geometry in Cartesian Coordinates

```
C          -1.849233      -0.926036      -0.085076
O          -1.904784      -2.210069      -0.063489
C          -0.797545      -0.110617       0.237936
C           0.480087      -0.719532       0.691553
C          -0.814638       1.391600       0.126053
C          -2.153494       2.035889      -0.215089
H          -2.769542      -0.399081      -0.395915
H           0.345052      -1.789500       0.837718
H           0.853239      -0.287147       1.625774
H          -0.083897       1.712106      -0.629744
H          -0.448826       1.827577       1.066927
H          -2.059587       3.122010      -0.237401
H          -2.514665       1.712787      -1.192171
H          -2.917449       1.777728       0.519997
C           2.723322       0.908900       0.175597
H           2.067870       1.775078       0.120144
H           3.030397       0.750328       1.207930
H           3.607770       1.091576      -0.431183
S           1.912307      -0.568384      -0.473125
```

6_2ethylacrolein_HEI_5_reopt

Datum	Value
M06-2X/def2tzvpp-IEFPCM(water) Energy	-708.750409
M06-2X/def2tzvpp-IEFPCM(water) Free Energy (Quasiharmonic)	-708.623966
Number of Imaginary Frequencies	0

Frequencies (Top 3 out of 51)

```
1.      46.3403 cm-1
2.      80.1312 cm-1
3.     116.1938 cm-1
```

M06-2X/def2tzvpp-IEFPCM(water) Molecular Geometry in Cartesian Coordinates

```
C         -2.215912       -0.343211       -0.244514
O         -2.705190       -1.460907        0.145725
C         -0.964011        0.176131       -0.039470
C          0.037560       -0.610846        0.727721
C         -0.556457        1.498818       -0.623847
C         -0.104946        2.538175        0.406531
H         -2.884962        0.321742       -0.831064
H         -0.443070       -1.499281        1.134113
H          0.492022       -0.064528        1.559238
H         -1.399384        1.904497       -1.190840
H          0.255866        1.366159       -1.351898
H          0.744844        2.176024        0.988449
H         -0.913031        2.758752        1.106201
H          0.198974        3.471815       -0.070535
C          2.660082        0.102124       -0.027024
H          3.603153       -0.214945       -0.467411
H          2.322068        1.008275       -0.524449
H          2.816906        0.304721        1.031500
S          1.481890       -1.250820       -0.234093
```

6_2ethylacrolein_HEI_6

Datum	Value

Datum	Value
M06-2X/def2tzvpp-IEFPCM(water) Energy	-708.75369
M06-2X/def2tzvpp-IEFPCM(water) Free Energy (Quasiharmonic)	-708.627786
Number of Imaginary Frequencies	0

Frequencies (Top 3 out of 51)

```
1.      49.5605 cm-1
2.      70.7235 cm-1
3.      84.1861 cm-1
```

M06-2X/def2tzvpp-IEFPCM(water) Molecular Geometry in Cartesian Coordinates

```
C          0.970522       -1.454678       -0.383340
O          1.917653       -2.121459        0.158777
C          0.636559       -0.136657       -0.209937
C         -0.503172        0.446218       -0.954705
C          1.399353        0.755015        0.728642
C          2.423736        1.651121        0.023732
H          0.317834       -1.995227       -1.102512
H         -0.818469       -0.200839       -1.775242
H         -0.286460        1.433168       -1.372339
H          0.700965        1.391370        1.284024
H          1.916528        0.136785        1.465321
H          2.930841        2.320137        0.722363
H          3.180365        1.043095       -0.474761
H          1.941458        2.268139       -0.737154
C         -2.441452       -0.925644        0.487206
H         -2.732104       -1.492046       -0.396942
H         -1.577591       -1.398988        0.951600
H         -3.270286       -0.907391        1.191032
S         -2.034848        0.772576        0.051551
```

6_2ethylacrolein_HEI_7

Datum	Value
M06-2X/def2tzvpp-IEFPCM(water) Energy	-708.750136
M06-2X/def2tzvpp-IEFPCM(water) Free Energy (Quasiharmonic)	-708.624003
Number of Imaginary Frequencies	0

Frequencies (Top 3 out of 51)

```
1.      50.9865 cm-1
2.      69.6115 cm-1
3.      73.6690 cm-1
```

M06-2X/def2tzvpp-IEFPCM(water) Molecular Geometry in Cartesian Coordinates

```
C        -1.464276      1.279920     -0.074037
O        -0.971776      2.465505     -0.094588
C        -0.847106      0.113045      0.288201
C         0.573458      0.150470      0.731692
C        -1.492798     -1.249952      0.260854
C        -2.940476     -1.304010     -0.212341
H        -2.521902      1.178156     -0.377547
H         0.774138     -0.563564      1.535765
H         0.851836      1.150819      1.060670
H        -1.442228     -1.698517      1.262719
H        -0.900219     -1.923123     -0.373105
H        -3.590311     -0.700166      0.422827
H        -3.039428     -0.930840     -1.232770
H        -3.310922     -2.329316     -0.192149
C         3.280926     -0.335666      0.263175
H         3.248188     -1.083382      1.054295
H         3.496122      0.639927      0.696435
H         4.071602     -0.594980     -0.437331
S         1.717436     -0.299494     -0.638024
```

6_2ethylacrolein_HEI_8

Datum	Value
M06-2X/def2tzvpp-IEFPCM(water) Energy	-708.749671
M06-2X/def2tzvpp-IEFPCM(water) Free Energy (Quasiharmonic)	-708.623237
Number of Imaginary Frequencies	0

Frequencies (Top 3 out of 51)

```
1.      47.3676 cm-1
2.      52.0017 cm-1
3.      99.9024 cm-1
```

M06-2X/def2tzvpp-IEFPCM(water) Molecular Geometry in Cartesian Coordinates

```
C          1.217199       1.433479       -0.029129
O          2.243474       1.740017        0.668611
C          0.617806       0.212308       -0.188691
C         -0.561702       0.070345       -1.082501
C          1.184742      -1.024085        0.459530
C          2.199333      -1.759030       -0.424472
H          0.725873       2.255752       -0.594172
H         -0.598305      -0.918623       -1.544566
H         -0.564925       0.809472       -1.885119
H          1.671531      -0.747866        1.398421
H          0.384342      -1.723954        0.715979
H          2.577331      -2.665946        0.053151
H          1.743274      -2.045595       -1.374528
H          3.048674      -1.111322       -0.646203
C         -2.119568      -0.690820        1.149315
H         -2.034641      -1.744772        0.888450
H         -1.257200      -0.389640        1.742187
H         -3.026283      -0.539592        1.730869
S         -2.240270       0.340548       -0.321104
```

6_2ethylacrolein_HEI_9

Datum	Value
M06-2X/def2tzvpp-IEFPCM(water) Energy	-708.752205
M06-2X/def2tzvpp-IEFPCM(water) Free Energy (Quasiharmonic)	-708.626445
Number of Imaginary Frequencies	0

Frequencies (Top 3 out of 51)

```
1.     40.6516 cm-1
2.     59.5642 cm-1
3.     86.1327 cm-1
```

M06-2X/def2tzvpp-IEFPCM(water) Molecular Geometry in Cartesian Coordinates

```
C         -1.532784      -1.453451       -0.120118
O         -1.072975      -2.571914        0.296833
C         -0.977808      -0.203845       -0.027941
```

```
C          0.350972       -0.032846        0.620200
C         -1.653819        1.008705       -0.606621
C         -2.120477        2.033559        0.432422
H         -2.522069       -1.469780       -0.625007
H          0.573892       -0.878649        1.269711
H          0.427627        0.889780        1.200419
H         -2.518850        0.681961       -1.191006
H         -0.987356        1.521391       -1.312439
H         -2.855373        1.586694        1.104085
H         -1.286118        2.385652        1.041594
H         -2.574810        2.907503       -0.038777
C          3.101474        0.421242        0.435608
H          2.947933        1.365873        0.955236
H          3.247668       -0.373692        1.165272
H          3.990337        0.499363       -0.186502
S          1.695973        0.051189       -0.634909
```

6_2ethylacrolein_TS_10_reopt

Datum	Value
M06-2X/def2tzvpp-IEFPCM(water) Energy	-708.741213
M06-2X/def2tzvpp-IEFPCM(water) Free Energy (Quasiharmonic)	-708.615924
Number of Imaginary Frequencies	1

Frequencies (Top 3 out of 51)

```
1.    -186.4154 cm-1
2.      73.8370 cm-1
3.      87.3594 cm-1
```

M06-2X/def2tzvpp-IEFPCM(water) Molecular Geometry in Cartesian Coordinates

```
C         -1.449309       -1.253680        0.077139
O         -2.471866       -1.134509        0.758117
C         -0.841640       -0.238981       -0.721185
C          0.278777       -0.566970       -1.443195
C         -1.397671        1.157270       -0.679422
C         -1.015825        1.897813        0.605399
H         -0.942956       -2.238036        0.036001
H          0.620633        0.069487       -2.245846
H          0.588039       -1.601716       -1.499103
H         -1.031952        1.715714       -1.543718
H         -2.486853        1.119406       -0.758163
```

```
H          -1.429373       2.907239       0.619786
H          -1.392997       1.361524       1.476819
H           0.069424       1.964813       0.693343
C           1.670919      -0.307363       1.405413
H           1.819471       0.522736       2.095823
H           0.588837      -0.462155       1.307942
H           2.105022      -1.204405       1.846201
S           2.362258       0.049558      -0.222433
```

6_2ethylacrolein_TS_11_reopt

Datum	Value
M06-2X/def2tzvpp-IEFPCM(water) Energy	-708.731764
M06-2X/def2tzvpp-IEFPCM(water) Free Energy (Quasiharmonic)	-708.607797
Number of Imaginary Frequencies	1

Frequencies (Top 3 out of 51)

```
1.    -223.3808 cm-1
2.      48.4183 cm-1
3.      71.2113 cm-1
```

M06-2X/def2tzvpp-IEFPCM(water) Molecular Geometry in Cartesian Coordinates

```
C          -2.183047      -0.588327      -0.144863
O          -2.283946      -1.788981      -0.403557
C          -1.101111       0.061854       0.532222
C          -0.080371      -0.698254       1.038534
C          -1.071581       1.567651       0.541095
C          -0.613525       2.156605      -0.796020
H          -3.008549       0.086729      -0.448636
H          -0.201606      -1.770407       1.087266
H           0.639022      -0.262666       1.718847
H          -2.064995       1.960271       0.778121
H          -0.404304       1.908863       1.336295
H          -0.561616       3.245809      -0.758198
H          -1.307296       1.879196      -1.592118
H           0.369004       1.767214      -1.065641
C           2.692794       0.507339       0.276668
H           1.976213       1.322444       0.399065
H           3.093625       0.263116       1.261155
H           3.514201       0.872711      -0.338819
S           1.898057      -0.937665      -0.489669
```

6_2ethylacrolein_TS_1

Datum	Value
M06-2X/def2tzvpp-IEFPCM(water) Energy	-708.734214
M06-2X/def2tzvpp-IEFPCM(water) Free Energy (Quasiharmonic)	-708.609492
Number of Imaginary Frequencies	1

Frequencies (Top 3 out of 51)

```
1.    -149.1867 cm-1
2.      40.6311 cm-1
3.      65.9663 cm-1
```

M06-2X/def2tzvpp-IEFPCM(water) Molecular Geometry in Cartesian Coordinates

```
C         -1.194081        1.264713        0.402080
O         -0.669064        2.365219        0.557307
C         -0.819758        0.282644       -0.590168
C          0.199740        0.586178       -1.432623
C         -1.420101       -1.107079       -0.612481
C         -2.536253       -1.382005        0.388983
H         -2.029800        0.988997        1.066096
H          0.614932        1.582285       -1.445436
H          0.457786       -0.067488       -2.252772
H         -1.788235       -1.320943       -1.619071
H         -0.605928       -1.819863       -0.436418
H         -2.895884       -2.404081        0.275863
H         -2.190923       -1.264270        1.417321
H         -3.384816       -0.712174        0.242598
C          1.546063       -0.676067        1.328346
H          1.977225       -0.117082        2.159056
H          0.492521       -0.376572        1.243146
H          1.564005       -1.736402        1.582334
S          2.405498       -0.342779       -0.224749
```

6_2ethylacrolein_TS_2

Datum	Value

Datum	Value
M06-2X/def2tzvpp-IEFPCM(water) Energy	-708.736714
M06-2X/def2tzvpp-IEFPCM(water) Free Energy (Quasiharmonic)	-708.612756
Number of Imaginary Frequencies	1

Frequencies (Top 3 out of 51)

```
1.    -150.0792 cm-1
2.      30.6711 cm-1
3.      52.3600 cm-1
```

M06-2X/def2tzvpp-IEFPCM(water) Molecular Geometry in Cartesian Coordinates

```
C          0.869585       -1.627611        0.277608
O          0.273230       -2.561809       -0.249762
C          0.879019       -0.253631       -0.163741
C          0.160119        0.087672       -1.265339
C          1.595072        0.780542        0.666124
C          2.811223        1.388986       -0.031991
H          1.471467       -1.826211        1.185998
H         -0.276497       -0.682130       -1.883370
H          0.222775        1.079091       -1.688943
H          0.891188        1.578566        0.923597
H          1.908487        0.331233        1.612091
H          2.520221        1.851834       -0.975849
H          3.285105        2.152176        0.586291
H          3.552119        0.619629       -0.253261
C         -1.974306        0.152525        1.253570
H         -2.096253        0.941050        1.996394
H         -0.946427       -0.225653        1.339048
H         -2.647611       -0.666080        1.508477
S         -2.257168        0.760630       -0.423110
```

6_2ethylacrolein_TS_3_reopt

Datum	Value
M06-2X/def2tzvpp-IEFPCM(water) Energy	-708.736628
M06-2X/def2tzvpp-IEFPCM(water) Free Energy (Quasiharmonic)	-708.611779
Number of Imaginary Frequencies	1

Frequencies (Top 3 out of 51)

```
1.    -176.8316 cm-1
2.      40.4011 cm-1
3.      68.1290 cm-1
```

M06-2X/def2tzvpp-IEFPCM(water) Molecular Geometry in Cartesian Coordinates

```
C         -1.490241        1.193859       -0.397538
O         -1.030703        2.328063       -0.522331
C         -1.067505        0.185430        0.537395
C         -0.067422        0.476728        1.418813
C         -1.712168       -1.176374        0.464839
C         -1.103260       -2.089731       -0.601743
H         -2.334827        0.891211       -1.049021
H          0.158490       -0.190167        2.237861
H          0.279399        1.494694        1.508145
H         -1.627654       -1.659425        1.440388
H         -2.782425       -1.065068        0.268094
H         -1.237442       -1.663573       -1.597515
H         -1.569426       -3.075966       -0.590039
H         -0.031910       -2.207122       -0.433364
C          1.649235       -0.069669       -1.296844
H          0.558269        0.035616       -1.307398
H          2.074409        0.775894       -1.837815
H          1.894380       -0.983989       -1.837471
S          2.225783       -0.131129        0.414203
```

6_2ethylacrolein_TS_4_reopt2

Datum	Value
M06-2X/def2tzvpp-IEFPCM(water) Energy	-708.736714
M06-2X/def2tzvpp-IEFPCM(water) Free Energy (Quasiharmonic)	-708.612755
Number of Imaginary Frequencies	1

Frequencies (Top 3 out of 51)

```
1.    -150.0580 cm-1
2.      30.6804 cm-1
3.      52.3396 cm-1
```

M06-2X/def2tzvpp-IEFPCM(water) Molecular Geometry in Cartesian Coordinates

```
C          -0.869534        1.627624        0.277545
O          -0.273218        2.561783       -0.249946
C          -0.879016        0.253615       -0.163704
C          -0.160204       -0.087778       -1.265334
C          -1.594983       -0.780497        0.666312
C          -2.811260       -1.388935       -0.031588
H          -1.471327        1.826293        1.185978
H           0.276331        0.681974       -1.883485
H          -0.222901       -1.079234       -1.688847
H          -1.908241       -0.331136        1.612307
H          -0.891083       -1.578529        0.923716
H          -3.285080       -2.152069        0.586809
H          -3.552159       -0.619562       -0.252794
H          -2.520416       -1.851848       -0.975463
C           1.974348       -0.152556        1.253448
H           0.946499        0.225691        1.338967
H           2.096261       -0.941122        1.996235
H           2.647717        0.665992        1.508368
S           2.257127       -0.760597       -0.423268
```

6_2ethylacrolein_TS_5_reopt3

Datum	Value
M06-2X/def2tzvpp-IEFPCM(water) Energy	-708.735787
M06-2X/def2tzvpp-IEFPCM(water) Free Energy (Quasiharmonic)	-708.610591
Number of Imaginary Frequencies	1

Frequencies (Top 3 out of 51)

```
1.    -157.9454 cm-1
2.      46.8608 cm-1
3.      80.2895 cm-1
```

M06-2X/def2tzvpp-IEFPCM(water) Molecular Geometry in Cartesian Coordinates

```
C           1.423551       -1.346452        0.381686
O           0.926702       -2.456874        0.210889
C           1.143237       -0.150281       -0.374351
```

```
C          0.239254      -0.223791      -1.389443
C          1.831560       1.137919       0.014350
C          0.876350       2.289408       0.325822
H          2.169477      -1.219240       1.190474
H         -0.110269      -1.187490      -1.724053
H          0.072740       0.614017      -2.051457
H          2.507256       1.453106      -0.786066
H          2.465212       0.950097       0.885089
H          0.222572       2.039712       1.161410
H          0.241028       2.509437      -0.532322
H          1.429965       3.194283       0.578883
C         -1.661581      -0.055675       1.290728
H         -2.089836      -0.939425       1.764347
H         -0.568238      -0.150721       1.333235
H         -1.937103       0.818486       1.881225
S         -2.183166       0.104122      -0.430040
```

6_2ethylacrolein_TS_6

Datum	Value
M06-2X/def2tzvpp-IEFPCM(water) Energy	-708.73997
M06-2X/def2tzvpp-IEFPCM(water) Free Energy (Quasiharmonic)	-708.61589
Number of Imaginary Frequencies	1

Frequencies (Top 3 out of 51)

```
1.    -178.2163 cm-1
2.      32.0492 cm-1
3.      44.5987 cm-1
```

M06-2X/def2tzvpp-IEFPCM(water) Molecular Geometry in Cartesian Coordinates

```
C          1.018062      -1.325609      -0.578804
O          1.862150      -1.977163       0.042585
C          0.689256       0.046752      -0.374476
C         -0.259873       0.615751      -1.187179
C          1.360450       0.808547       0.734234
C          2.718526       1.382477       0.325540
H          0.450684      -1.819387      -1.392047
H         -0.620089       0.067853      -2.047177
H         -0.368614       1.689093      -1.236038
H          0.703008       1.618929       1.057756
H          1.497840       0.145234       1.591688
```

```
H          3.191631        1.917174        1.150359
H          3.389163        0.583298        0.008322
H          2.604201        2.076284       -0.508605
C         -2.130495       -0.864151        0.936387
H         -2.683315       -1.750181        0.625130
H         -1.058667       -1.089025        0.859137
H         -2.353592       -0.670723        1.985165
S         -2.501563        0.566634       -0.098036
```

6_2ethylacrolein_TS_7_reopt

Datum	Value
M06-2X/def2tzvpp-IEFPCM(water) Energy	-708.731083
M06-2X/def2tzvpp-IEFPCM(water) Free Energy (Quasiharmonic)	-708.608764
Number of Imaginary Frequencies	1

Frequencies (Top 3 out of 51)

```
1.    -186.6098 cm-1
2.      11.1587 cm-1
3.      25.0467 cm-1
```

M06-2X/def2tzvpp-IEFPCM(water) Molecular Geometry in Cartesian Coordinates

```
C         -1.534615        1.556723       -0.108662
O         -0.936154        2.557255        0.286245
C         -1.112585        0.191946        0.024537
C          0.063810       -0.075883        0.667095
C         -1.921659       -0.898864       -0.629353
C         -2.564654       -1.866168        0.364702
H         -2.505327        1.692651       -0.624932
H          0.339511       -1.091248        0.916481
H          0.556440        0.706567        1.225884
H         -1.281455       -1.465234       -1.313571
H         -2.702263       -0.447340       -1.246877
H         -1.805114       -2.334016        0.992542
H         -3.257915       -1.337264        1.020045
H         -3.113498       -2.658271       -0.146217
C          3.167833       -0.497774        0.700444
H          2.523249       -0.642427        1.572632
H          3.850612        0.320462        0.927405
H          3.757257       -1.405131        0.572554
S          2.158685       -0.141042       -0.768655
```

6_2ethylacrolein_TS_8_reopt

Datum	Value
M06-2X/def2tzvpp-IEFPCM(water) Energy	-708.73997
M06-2X/def2tzvpp-IEFPCM(water) Free Energy (Quasiharmonic)	-708.615915
Number of Imaginary Frequencies	1

Frequencies (Top 3 out of 51)

```
1.    -178.2804 cm-1
2.      30.7166 cm-1
3.      44.2395 cm-1
```

M06-2X/def2tzvpp-IEFPCM(water) Molecular Geometry in Cartesian Coordinates

```
C         -1.018215        1.325444        0.579034
O         -1.862352        1.976824       -0.042483
C         -0.689091       -0.046838        0.374768
C          0.260171       -0.615596        1.187524
C         -1.359951       -0.808745       -0.734069
C         -2.718238       -1.382492       -0.325816
H         -0.451039        1.819362        1.392335
H          0.369124       -1.688915        1.236431
H          0.620122       -0.067618        2.047583
H         -1.496961       -0.145549       -1.591677
H         -0.702452       -1.619227       -1.057232
H         -3.191074       -1.917264       -1.150742
H         -2.604309       -2.076191        0.508473
H         -3.388922       -0.583202       -0.008982
C          2.129511        0.863370       -0.937709
H          2.352045        0.668601       -1.986359
H          1.057652        1.087958       -0.860104
H          2.682181        1.750032       -0.628003
S          2.501708       -0.565840        0.098485
```

6_2ethylacrolein_TS_9_reopt

Datum	Value

Datum	Value
M06-2X/def2tzvpp-IEFPCM(water) Energy	-708.731081
M06-2X/def2tzvpp-IEFPCM(water) Free Energy (Quasiharmonic)	-708.608217
Number of Imaginary Frequencies	1

Frequencies (Top 3 out of 51)

```
1.    -198.2196 cm-1
2.      21.8719 cm-1
3.      44.3025 cm-1
```

M06-2X/def2tzvpp-IEFPCM(water) Molecular Geometry in Cartesian Coordinates

```
C          1.590429        1.534109       -0.075713
O          1.025995        2.548072        0.334660
C          1.117992        0.183437        0.030744
C         -0.066626       -0.052240        0.670411
C          1.880795       -0.922677       -0.652357
C          2.450453       -1.964804        0.310182
H          2.567940        1.644233       -0.585091
H         -0.528087        0.735711        1.247787
H         -0.381157       -1.061243        0.898973
H          2.696675       -0.489315       -1.236349
H          1.224897       -1.424488       -1.371481
H          3.158802       -1.503414        0.999483
H          1.655007       -2.417002        0.904001
H          2.965001       -2.763517       -0.225458
C         -3.110269       -0.614251        0.676881
H         -2.611195       -0.331241        1.607679
H         -4.116795       -0.198202        0.703186
H         -3.193683       -1.700988        0.661389
S         -2.176376        0.008965       -0.752644
```

7_transtrans24hexadienal_1

Datum	Value
M06-2X/def2tzvpp-IEFPCM(water) Energy	-308.624168
M06-2X/def2tzvpp-IEFPCM(water) Free Energy (Quasiharmonic)	-308.528847
Number of Imaginary Frequencies	0

Frequencies (Top 3 out of 39)

```
1.     72.3362 cm-1
2.    113.1949 cm-1
3.    170.0887 cm-1
```

M06-2X/def2tzvpp-IEFPCM(water) Molecular Geometry in Cartesian Coordinates

```
C        -2.584532     0.333031     0.000000
C        -1.321014    -0.388965    -0.000001
C        -0.162188     0.287487    -0.000001
C         1.150431    -0.318567    -0.000001
C         2.272680     0.406504     0.000001
C         3.650815    -0.156300     0.000000
O        -3.678052    -0.192196     0.000001
H        -2.495133     1.433941    -0.000001
H        -1.355495    -1.472178    -0.000002
H        -0.197126     1.374428     0.000000
H         1.202933    -1.402736     0.000003
H         2.186407     1.489703     0.000001
H         4.203838     0.189899     0.875302
H         3.638004    -1.244521     0.000003
H         4.203836     0.189895    -0.875304
```

7_transtrans24hexadienal_2

Datum	Value
M06-2X/def2tzvpp-IEFPCM(water) Energy	-308.620617
M06-2X/def2tzvpp-IEFPCM(water) Free Energy (Quasiharmonic)	-308.525656
Number of Imaginary Frequencies	0

Frequencies (Top 3 out of 39)

```
1.    101.1821 cm-1
2.    114.2937 cm-1
3.    115.8993 cm-1
```

M06-2X/def2tzvpp-IEFPCM(water) Molecular Geometry in Cartesian Coordinates

```
C          2.719912       0.269816       -0.000001
C          1.348841       0.788966        0.000001
C          0.288084      -0.031674       -0.000001
C         -1.089464       0.405291        0.000000
C         -2.112793      -0.454338       -0.000001
C         -3.550927      -0.068756        0.000000
O          3.015528      -0.906945        0.000002
H          3.516008       1.032082       -0.000007
H          1.225873       1.864956        0.000003
H          0.469015      -1.102946       -0.000003
H         -1.278131       1.474431        0.000001
H         -1.891255      -1.518083       -0.000001
H         -4.056366      -0.481619        0.875224
H         -3.674913       1.012530        0.000001
H         -4.056369      -0.481619       -0.875222
```

7_transtrans24hexadienal_3

Datum	Value
M06-2X/def2tzvpp-IEFPCM(water) Energy	-308.624168
M06-2X/def2tzvpp-IEFPCM(water) Free Energy (Quasiharmonic)	-308.52885
Number of Imaginary Frequencies	0

Frequencies (Top 3 out of 39)

```
1.      72.6298 cm-1
2.     113.5176 cm-1
3.     170.2949 cm-1
```

M06-2X/def2tzvpp-IEFPCM(water) Molecular Geometry in Cartesian Coordinates

```
C         -2.585041       0.332858       -0.000023
C         -1.321064      -0.388932       -0.000032
C         -0.162102       0.287405       -0.000026
C          1.150675      -0.318539        0.000040
C          2.272891       0.406634        0.000111
C          3.651262      -0.156348       -0.000051
O         -3.678600      -0.192126        0.000021
H         -2.495643       1.433933       -0.000055
H         -1.355131      -1.472160       -0.000016
H         -0.196723       1.374408       -0.000032
H          1.203140      -1.402713        0.000076
```

```
H          2.186360       1.489809       0.000099
H          4.204111       0.189847      -0.875472
H          4.204614       0.189997       0.874978
H          3.638343      -1.244581       0.000137
```

7_transtrans24hexadienal_4

Datum	Value
M06-2X/def2tzvpp-IEFPCM(water) Energy	-308.619041
M06-2X/def2tzvpp-IEFPCM(water) Free Energy (Quasiharmonic)	-308.524236
Number of Imaginary Frequencies	0

Frequencies (Top 3 out of 39)

```
1.      80.3627 cm-1
2.     109.3520 cm-1
3.     126.9007 cm-1
```

M06-2X/def2tzvpp-IEFPCM(water) Molecular Geometry in Cartesian Coordinates

```
C          2.515296       0.144962       0.054983
C          1.093624      -0.135462      -0.092545
C          0.206325       0.863086       0.023087
C         -1.244259       0.760170      -0.071492
C         -1.936734      -0.369079       0.091531
C         -3.420029      -0.471501      -0.002214
O          3.392329      -0.689582      -0.018618
H          2.767419       1.203677       0.244091
H          0.805564      -1.158298      -0.303608
H          0.601262       1.860860       0.194916
H         -1.781086       1.684128      -0.256808
H         -1.402919      -1.285673       0.325052
H         -3.838493      -0.847123       0.933418
H         -3.873357       0.491514      -0.229621
H         -3.702364      -1.185483      -0.778594
```

7_transtrans24hexadienal_5

Datum	Value

Datum	Value
M06-2X/def2tzvpp-IEFPCM(water) Energy	-308.619041
M06-2X/def2tzvpp-IEFPCM(water) Free Energy (Quasiharmonic)	-308.524236
Number of Imaginary Frequencies	0

Frequencies (Top 3 out of 39)

```
1.      80.3624 cm-1
2.     109.3520 cm-1
3.     126.9007 cm-1
```

M06-2X/def2tzvpp-IEFPCM(water) Molecular Geometry in Cartesian Coordinates

```
C          -2.515296        0.144962        0.054982
C          -1.093624       -0.135461       -0.092545
C          -0.206325        0.863086        0.023087
C           1.244259        0.760170       -0.071492
C           1.936734       -0.369079        0.091531
C           3.420029       -0.471501       -0.002213
O          -3.392329       -0.689582       -0.018618
H          -2.767419        1.203677        0.244090
H          -0.805564       -1.158298       -0.303607
H          -0.601262        1.860860        0.194915
H           1.781086        1.684128       -0.256807
H           1.402919       -1.285673        0.325051
H           3.702364       -1.185483       -0.778594
H           3.873357        0.491514       -0.229620
H           3.838493       -0.847124        0.933418
```

7_transtrans24hexadienal_6

Datum	Value
M06-2X/def2tzvpp-IEFPCM(water) Energy	-308.615228
M06-2X/def2tzvpp-IEFPCM(water) Free Energy (Quasiharmonic)	-308.520463
Number of Imaginary Frequencies	0

Frequencies (Top 3 out of 39)

```
1.      60.2935 cm-1
2.     122.1685 cm-1
3.     142.7241 cm-1
```

M06-2X/def2tzvpp-IEFPCM(water) Molecular Geometry in Cartesian Coordinates

```
C          2.521383      -0.551417      -0.034368
C          1.057877      -0.560962      -0.149314
C          0.347171       0.570313      -0.047899
C         -1.102818       0.693588      -0.121398
C         -1.962842      -0.297959       0.121274
C         -3.445427      -0.167978       0.048315
O          3.195863       0.435791       0.168690
H          3.010949      -1.532134      -0.149941
H          0.588639      -1.518479      -0.336583
H          0.906269       1.490811       0.089805
H         -1.490810       1.678721      -0.357706
H         -1.576067      -1.270986       0.410136
H         -3.900572      -0.411395       1.010188
H         -3.744345       0.838714      -0.238004
H         -3.853037      -0.875089      -0.677071
```

7_transtrans24hexadienal_7

Datum	Value
M06-2X/def2tzvpp-IEFPCM(water) Energy	-308.615228
M06-2X/def2tzvpp-IEFPCM(water) Free Energy (Quasiharmonic)	-308.520463
Number of Imaginary Frequencies	0

Frequencies (Top 3 out of 39)

```
1.      60.2935 cm-1
2.     122.1685 cm-1
3.     142.7241 cm-1
```

M06-2X/def2tzvpp-IEFPCM(water) Molecular Geometry in Cartesian Coordinates

```
C         -2.521383       -0.551417       -0.034368
C         -1.057877       -0.560962       -0.149314
C         -0.347171        0.570313       -0.047899
C          1.102818        0.693588       -0.121398
C          1.962842       -0.297959        0.121274
C          3.445427       -0.167978        0.048315
O         -3.195863        0.435791        0.168690
H         -3.010949       -1.532134       -0.149941
H         -0.588639       -1.518479       -0.336583
H         -0.906269        1.490811        0.089805
H          1.490810        1.678721       -0.357706
H          1.576067       -1.270986        0.410135
H          3.744345        0.838714       -0.238004
H          3.900572       -0.411396        1.010188
H          3.853037       -0.875089       -0.677071
```

7_transtrans24hexadienal_HEI_10

Datum	Value
M06-2X/def2tzvpp-IEFPCM(water) Energy	-746.838593
M06-2X/def2tzvpp-IEFPCM(water) Free Energy (Quasiharmonic)	-746.710027
Number of Imaginary Frequencies	0

Frequencies (Top 3 out of 54)

```
1.      53.8882 cm-1
2.      67.3390 cm-1
3.      86.3989 cm-1
```

M06-2X/def2tzvpp-IEFPCM(water) Molecular Geometry in Cartesian Coordinates

```
C         -2.661727        0.648249        0.394123
C         -1.563496        0.476372       -0.406208
C         -0.261901       -0.015595        0.123886
C          0.914936        0.761886       -0.372604
C          1.890249        1.240818        0.391251
C          3.060186        2.030346       -0.107731
O         -3.826843        1.055068        0.083744
H         -2.480545        0.397696        1.462765
H         -1.627047        0.690432       -1.469682
H         -0.272686       -0.012608        1.216553
H          0.944638        0.926494       -1.448176
```

```
H          1.847125       1.058567       1.462661
H          4.001452       1.536356       0.141665
H          3.015246       2.157370      -1.188913
H          3.092411       3.019475       0.353438
C          1.516124      -2.217378       0.439880
H          1.451902      -2.035959       1.512102
H          1.701352      -3.276144       0.270416
H          2.336187      -1.635864       0.024119
S         -0.047595      -1.800909      -0.361405
```

7_transtrans24hexadienal_HEI_11

Datum	Value
M06-2X/def2tzvpp-IEFPCM(water) Energy	-746.834771
M06-2X/def2tzvpp-IEFPCM(water) Free Energy (Quasiharmonic)	-746.705423
Number of Imaginary Frequencies	0

Frequencies (Top 3 out of 54)

```
1.      54.7698 cm-1
2.      80.6596 cm-1
3.     101.7824 cm-1
```

M06-2X/def2tzvpp-IEFPCM(water) Molecular Geometry in Cartesian Coordinates

```
C         -2.419515       0.279058      -0.154526
C         -1.630488      -0.616069       0.518829
C         -0.147789      -0.483979       0.687278
C          0.350520       0.936350       0.657240
C          1.142039       1.513876      -0.236700
C          1.570673       2.947917      -0.184666
O         -3.675632       0.221106      -0.368785
H         -1.881968       1.163466      -0.548954
H         -2.084986      -1.516286       0.922162
H          0.136541      -0.895884       1.661935
H         -0.014715       1.534778       1.490231
H          1.503534       0.925760      -1.074984
H          2.658696       3.032624      -0.149213
H          1.158444       3.452536       0.688704
H          1.243500       3.485776      -1.076952
C          2.376697      -1.643655       0.134304
H          2.878043      -0.686297       0.009695
H          2.922209      -2.409772      -0.412609
```

```
H          2.360279      -1.909909       1.190486
S          0.692042      -1.596665      -0.517549
```

7_transtrans24hexadienal_HEI_1

Datum	Value
M06-2X/def2tzvpp-IEFPCM(water) Energy	-746.83439
M06-2X/def2tzvpp-IEFPCM(water) Free Energy (Quasiharmonic)	-746.704981
Number of Imaginary Frequencies	0

Frequencies (Top 3 out of 54)

```
1.      63.1028 cm-1
2.      68.0144 cm-1
3.     105.7739 cm-1
```

M06-2X/def2tzvpp-IEFPCM(water) Molecular Geometry in Cartesian Coordinates

```
C         -2.592552      -0.840803      -0.194326
C         -1.339762      -0.571266       0.289806
C         -0.207294      -0.177683      -0.595824
C          1.038180      -0.977701      -0.295623
C          2.262126      -0.547563      -0.015966
C          3.420018      -1.443753       0.300036
O         -3.644172      -1.177172       0.438802
H         -2.674124      -0.750022      -1.300620
H         -1.150412      -0.635361       1.357188
H         -0.499048      -0.380563      -1.631443
H          0.854861      -2.050973      -0.288136
H          2.457565       0.520319      -0.015252
H          3.131415      -2.493578       0.257868
H          4.241076      -1.284438      -0.401828
H          3.815279      -1.236406       1.296606
C          0.180737       2.041108       1.094003
H          1.000032       1.516681       1.582656
H          0.339164       3.113582       1.182425
H         -0.760123       1.774433       1.571331
S          0.114311       1.651854      -0.663365
```

7_transtrans24hexadienal_HEI_2

Datum	Value
M06-2X/def2tzvpp-IEFPCM(water) Energy	-746.83069
M06-2X/def2tzvpp-IEFPCM(water) Free Energy (Quasiharmonic)	-746.701697
Number of Imaginary Frequencies	0

Frequencies (Top 3 out of 54)

```
1.      52.3705 cm-1
2.      68.3430 cm-1
3.      98.6020 cm-1
```

M06-2X/def2tzvpp-IEFPCM(water) Molecular Geometry in Cartesian Coordinates

```
C        -2.284946     -0.992051     -0.084089
C        -0.925440     -1.134967     -0.075881
C         0.095426     -0.151950     -0.585724
C         1.378557     -0.255239      0.186335
C         2.552665     -0.582880     -0.340169
C         3.831546     -0.708111      0.427704
O        -3.171731     -1.831842      0.294457
H        -2.647455     -0.015615     -0.460999
H        -0.510459     -2.065438      0.304371
H         0.341341     -0.305281     -1.643828
H         1.304498     -0.077404      1.257232
H         2.606338     -0.777303     -1.408929
H         4.586333     -0.017440      0.046235
H         3.676874     -0.496985      1.485318
H         4.248778     -1.712670      0.334800
C        -0.945667      1.903361      1.074909
H        -0.117690      1.727483      1.758451
H        -1.234888      2.950476      1.136465
H        -1.789152      1.275182      1.349780
S        -0.456470      1.606297     -0.633316
```

7_transtrans24hexadienal_HEI_3

Datum	Value
M06-2X/def2tzvpp-IEFPCM(water) Energy	-746.836456
M06-2X/def2tzvpp-IEFPCM(water) Free Energy (Quasiharmonic)	-746.707235
Number of Imaginary Frequencies	0

Frequencies (Top 3 out of 54)

```
1.      51.6442 cm-1
2.      58.2323 cm-1
3.      79.4054 cm-1
```

M06-2X/def2tzvpp-IEFPCM(water) Molecular Geometry in Cartesian Coordinates

```
C          2.317603       1.105414      -0.225537
C          1.114427       0.612300       0.206406
C          0.182437      -0.091684      -0.703507
C         -1.261860       0.327860      -0.668525
C         -1.804989       1.198022       0.175232
C         -3.256335       1.566374       0.192875
O          3.224056       1.706836       0.435643
H          2.502858       0.952559      -1.312008
H          0.847312       0.708640       1.254099
H          0.546935      -0.005478      -1.731047
H         -1.904252      -0.164177      -1.395191
H         -1.166721       1.691838       0.901045
H         -3.809586       1.031125      -0.578321
H         -3.706999       1.336239       1.160531
H         -3.390751       2.637921       0.031895
C         -0.231284      -2.068517       1.250162
H         -1.165706      -1.546502       1.453560
H         -0.346619      -3.120586       1.500358
H          0.565364      -1.640450       1.855537
S          0.179732      -1.964652      -0.499265
```

7_transtrans24hexadienal_HEI_4

Datum	Value
M06-2X/def2tzvpp-IEFPCM(water) Energy	-746.837695
M06-2X/def2tzvpp-IEFPCM(water) Free Energy (Quasiharmonic)	-746.707671
Number of Imaginary Frequencies	0

Frequencies (Top 3 out of 54)

```
1.      70.7266 cm-1
2.      76.5618 cm-1
3.     101.7503 cm-1
```

M06-2X/def2tzvpp-IEFPCM(water) Molecular Geometry in Cartesian Coordinates

```
C          -2.056974       -1.570763        0.415619
C          -0.841663       -1.015681        0.724878
C          -0.051198       -0.209273       -0.246755
C           1.380423       -0.660703       -0.318995
C           2.475187        0.002013        0.035996
C           3.861899       -0.559898       -0.035914
O          -2.706225       -1.518983       -0.679863
H          -2.513459       -2.144237        1.248225
H          -0.435631       -1.155317        1.720177
H          -0.507538       -0.338215       -1.231328
H           1.488026       -1.682919       -0.676649
H           2.389650        1.018881        0.407988
H           4.497578        0.046167       -0.684469
H           4.333910       -0.567811        0.948589
H           3.853436       -1.579549       -0.420107
C          -1.899332        1.844675       -0.049753
H          -2.400972        1.128298        0.598723
H          -2.125273        2.858473        0.273176
H          -2.239330        1.707761       -1.074491
S          -0.118790        1.617382        0.073663
```

7_transtrans24hexadienal_HEI_5

Datum	Value
M06-2X/def2tzvpp-IEFPCM(water) Energy	-746.837712
M06-2X/def2tzvpp-IEFPCM(water) Free Energy (Quasiharmonic)	-746.709319
Number of Imaginary Frequencies	0

Frequencies (Top 3 out of 54)

```
1.      51.2689 cm-1
2.      59.7409 cm-1
3.      84.2701 cm-1
```

M06-2X/def2tzvpp-IEFPCM(water) Molecular Geometry in Cartesian Coordinates

```
C          -1.931376        1.512562        0.285924
C          -0.937829        0.926741       -0.454722
C           0.065546        0.015041        0.139056
C           1.465689        0.282984       -0.323780
C           2.519999        0.406669        0.473465
C           3.913736        0.696903        0.012102
O          -2.857963        2.297568       -0.094253
H          -1.895192        1.256531        1.367792
H          -0.894043        1.095675       -1.527764
H           0.022398        0.062273        1.229382
H           1.592001        0.387111       -1.399807
H           2.375109        0.298559        1.545925
H           3.957507        0.787097       -1.072733
H           4.286215        1.624988        0.450172
H           4.600304       -0.094123        0.320076
C          -1.907766       -1.985061        0.335009
H          -2.544234       -1.227068       -0.118292
H          -2.258156       -2.975676        0.053461
H          -1.943605       -1.886286        1.419081
S          -0.222662       -1.802791       -0.269725
```

7_transtrans24hexadienal_HEI_6

Datum	Value
M06-2X/def2tzvpp-IEFPCM(water) Energy	-746.837339
M06-2X/def2tzvpp-IEFPCM(water) Free Energy (Quasiharmonic)	-746.708011
Number of Imaginary Frequencies	0

Frequencies (Top 3 out of 54)

```
1.      51.6892 cm-1
2.      57.9151 cm-1
3.      90.7455 cm-1
```

M06-2X/def2tzvpp-IEFPCM(water) Molecular Geometry in Cartesian Coordinates

```
C           2.134965        1.529574        0.512917
C           1.036877        0.780228        0.849445
C           0.116268        0.224402       -0.173899
C          -1.306655        0.172626        0.285591
C          -2.334774        0.717974       -0.354059
C          -3.755116        0.678186        0.117171
```

```
O         2.548936       1.843623      -0.650048
H         2.716806       1.905306       1.378627
H         0.829959       0.588275       1.895244
H         0.190844       0.812201      -1.090520
H        -1.483220      -0.341761       1.228835
H        -2.147929       1.236233      -1.291732
H        -4.151056       1.685634       0.260010
H        -4.398421       0.188869      -0.617122
H        -3.840644       0.139384       1.060405
C         0.734181      -2.374134       0.757883
H         1.413119      -1.853843       1.431393
H        -0.239530      -2.487693       1.231051
H         1.134839      -3.360714       0.536176
S         0.614329      -1.477125      -0.799517
```

7_transtrans24hexadienal_HEI_7

Datum	Value
M06-2X/def2tzvpp-IEFPCM(water) Energy	-746.837944
M06-2X/def2tzvpp-IEFPCM(water) Free Energy (Quasiharmonic)	-746.708558
Number of Imaginary Frequencies	0

Frequencies (Top 3 out of 54)

```
1.     62.6222 cm-1
2.     73.8669 cm-1
3.     96.0449 cm-1
```

M06-2X/def2tzvpp-IEFPCM(water) Molecular Geometry in Cartesian Coordinates

```
C        -2.641964       1.120193      -0.215126
C        -1.312772       1.034534      -0.542518
C        -0.355573       0.173846       0.217206
C         0.961715       0.868560       0.432636
C         2.123658       0.630351      -0.162669
C         3.371706       1.426384       0.065207
O        -3.268648       0.528042       0.721295
H        -3.224783       1.806644      -0.862172
H        -0.934007       1.604558      -1.381839
H        -0.802432      -0.045830       1.190596
H         0.899816       1.695377       1.138585
H         2.190848      -0.192130      -0.868956
H         3.205211       2.223724       0.789038
```

```
H          4.181515       0.792479       0.432123
H          3.723571       1.877002      -0.865171
C          0.770405      -2.397923       0.565223
H          0.323143      -2.334594       1.556454
H          0.769458      -3.437484       0.244007
H          1.794803      -2.034413       0.606398
S         -0.217563      -1.457209      -0.619323
```

7_transtrans24hexadienal_HEI_8

Datum	Value
M06-2X/def2tzvpp-IEFPCM(water) Energy	-746.838725
M06-2X/def2tzvpp-IEFPCM(water) Free Energy (Quasiharmonic)	-746.708962
Number of Imaginary Frequencies	0

Frequencies (Top 3 out of 54)

```
1.      55.3335 cm-1
2.      77.6082 cm-1
3.      88.6896 cm-1
```

M06-2X/def2tzvpp-IEFPCM(water) Molecular Geometry in Cartesian Coordinates

```
C          1.368395       1.945961       0.356852
C          0.408388       1.014695       0.659504
C          0.003308      -0.044915      -0.289635
C         -1.470154      -0.300331      -0.448434
C         -2.446500       0.371737       0.150054
C         -3.905318       0.093745      -0.038176
O          2.030226       2.091576      -0.723179
H          1.574821       2.664330       1.176371
H         -0.035855       1.026234       1.647899
H          0.433554       0.179246      -1.268962
H         -1.733937      -1.115898      -1.119252
H         -2.185356       1.189016       0.814856
H         -4.065296      -0.737266      -0.724458
H         -4.382556      -0.149002       0.913409
H         -4.422444       0.970586      -0.433033
C          2.472190      -1.322691       0.034133
H          2.666680      -0.426394       0.621313
H          3.031206      -2.161303       0.443322
H          2.775325      -1.154374      -0.997589
S          0.720012      -1.722937       0.135360
```

7_transtrans24hexadienal_HEI_9

Datum	Value
M06-2X/def2tzvpp-IEFPCM(water) Energy	-746.834781
M06-2X/def2tzvpp-IEFPCM(water) Free Energy (Quasiharmonic)	-746.705081
Number of Imaginary Frequencies	0

Frequencies (Top 3 out of 54)

```
1.      54.1778 cm-1
2.      64.6646 cm-1
3.      91.7277 cm-1
```

M06-2X/def2tzvpp-IEFPCM(water) Molecular Geometry in Cartesian Coordinates

```
C          1.113208        2.038350       -0.298211
C          0.935294        0.965688       -1.135520
C          0.114992       -0.251934       -0.869776
C         -1.077870       -0.045851        0.012770
C         -2.327663       -0.296006       -0.361776
C         -3.535143       -0.090416        0.500255
O          0.651387        2.263158        0.867923
H          1.765732        2.822048       -0.738240
H          1.475802        0.982123       -2.074930
H         -0.229019       -0.678890       -1.814589
H         -0.873753        0.357844        0.997814
H         -2.508087       -0.674718       -1.365729
H         -3.254750        0.292420        1.481163
H         -4.230978        0.616052        0.042826
H         -4.083311       -1.024525        0.640664
C          1.874740       -0.911843        1.242245
H          1.144447       -0.756088        2.033554
H          2.658163       -1.575653        1.601509
H          2.304603        0.044418        0.949042
S          1.127169       -1.684890       -0.202274
```

7_transtrans24hexadienal_TS_10

Datum	Value
M06-2X/def2tzvpp-IEFPCM(water) Energy	-746.825243
M06-2X/def2tzvpp-IEFPCM(water) Free Energy (Quasiharmonic)	-746.698111
Number of Imaginary Frequencies	1

Frequencies (Top 3 out of 54)

```
1.    -203.8656 cm-1
2.      40.4172 cm-1
3.      72.0519 cm-1
```

M06-2X/def2tzvpp-IEFPCM(water) Molecular Geometry in Cartesian Coordinates

```
C         -2.868991        0.439742        0.367412
C         -1.679587        0.704300       -0.342267
C         -0.440809        0.455889        0.235211
C          0.785249        1.022528       -0.314120
C          1.870389        1.293662        0.411918
C          3.122511        1.909817       -0.123267
O         -4.030918        0.603325       -0.029309
H         -2.709658        0.052532        1.394303
H         -1.745515        1.055144       -1.366411
H         -0.421297        0.238133        1.296823
H          0.776554        1.253234       -1.376110
H          1.854435        1.059408        1.473317
H          3.981012        1.255795        0.043535
H          3.039971        2.104077       -1.191859
H          3.342945        2.851567        0.383286
C          1.826338       -2.164916        0.249756
H          1.878921       -2.663580        1.216997
H          2.369720       -2.767332       -0.478188
H          2.338511       -1.201165        0.334681
S          0.115697       -1.876910       -0.275235
```

7_transtrans24hexadienal_TS_11_reopt

Datum	Value
M06-2X/def2tzvpp-IEFPCM(water) Energy	-746.814263
M06-2X/def2tzvpp-IEFPCM(water) Free Energy (Quasiharmonic)	-746.687558
Number of Imaginary Frequencies	1

Frequencies (Top 3 out of 54)

```
1.    -230.6307 cm-1
2.      27.7112 cm-1
3.      57.3747 cm-1
```

M06-2X/def2tzvpp-IEFPCM(water) Molecular Geometry in Cartesian Coordinates

```
C         -2.519707       -0.065623       -0.156748
C         -1.597327       -0.863380        0.565494
C         -0.314116       -0.470606        0.906789
C          0.148306        0.932817        0.824661
C          0.421333        1.621606       -0.275457
C          0.858106        3.050959       -0.297370
O         -3.653363       -0.419610       -0.507410
H         -2.188220        0.957739       -0.395018
H         -1.923690       -1.862800        0.831786
H          0.177795       -1.045275        1.681825
H          0.269956        1.419182        1.791192
H          0.354115        1.106759       -1.229548
H          1.838315        3.150982       -0.768232
H          0.913781        3.463850        0.709287
H          0.165946        3.660188       -0.882324
C          2.795874       -0.786964        0.138083
H          2.968752        0.166556       -0.361631
H          3.694548       -1.396206        0.043717
H          2.640302       -0.579477        1.199825
S          1.347531       -1.637342       -0.549645
```

7_transtrans24hexadienal_TS_1_reopt

Datum	Value
M06-2X/def2tzvpp-IEFPCM(water) Energy	-746.828331
M06-2X/def2tzvpp-IEFPCM(water) Free Energy (Quasiharmonic)	-746.700822
Number of Imaginary Frequencies	1

Frequencies (Top 3 out of 54)

```
1.    -173.5738 cm-1
2.      54.0718 cm-1
3.      71.3456 cm-1
```

M06-2X/def2tzvpp-IEFPCM(water) Molecular Geometry in Cartesian Coordinates

```
C          -2.150344       -1.386974       -0.334630
C          -0.957939       -1.106072        0.376111
C           0.116237       -0.499272       -0.249838
C           1.464534       -0.565887        0.316514
C           2.577962       -0.522687       -0.412059
C           3.963496       -0.625864        0.138210
O          -3.158357       -1.956786        0.097985
H          -2.135316       -1.062662       -1.394747
H          -0.906636       -1.366709        1.427970
H           0.066810       -0.389356       -1.326577
H           1.529209       -0.687463        1.394634
H           2.486457       -0.398353       -1.488364
H           3.949011       -0.751162        1.220215
H           4.498086       -1.470835       -0.300289
H           4.544026        0.268591       -0.097717
C          -1.856073        2.047895        0.120888
H          -2.161728        2.677640        0.955753
H          -2.328986        2.423195       -0.786307
H          -2.237518        1.036378        0.311189
S          -0.061488        1.958012       -0.026926
```

7_transtrans24hexadienal_TS_2_reopt

Datum	Value
M06-2X/def2tzvpp-IEFPCM(water) Energy	-746.828331
M06-2X/def2tzvpp-IEFPCM(water) Free Energy (Quasiharmonic)	-746.700823
Number of Imaginary Frequencies	1

Frequencies (Top 3 out of 54)

```
1.    -173.5988 cm-1
2.      53.8751 cm-1
3.      71.3407 cm-1
```

M06-2X/def2tzvpp-IEFPCM(water) Molecular Geometry in Cartesian Coordinates

```
C          2.150496       -1.386814        0.334656
C          0.958133       -1.105845       -0.376104
C         -0.116124       -0.499211        0.249902
C         -1.464410       -0.565940       -0.316474
C         -2.577892       -0.522866        0.412023
C         -3.963370       -0.626128       -0.138370
O          3.158562       -1.956541       -0.097972
H          2.135395       -1.062653        1.394821
H          0.906902       -1.366293       -1.428013
H         -0.066716       -0.389535        1.326664
H         -1.529020       -0.687491       -1.394600
H         -2.486482       -0.398548        1.488336
H         -4.543909        0.268378        0.097344
H         -3.948781       -0.751610       -1.220352
H         -4.498019       -1.471012        0.300223
C          1.855757        2.047851       -0.121256
H          2.237152        1.036325       -0.311530
H          2.161072        2.677487       -0.956323
H          2.328984        2.423278        0.785725
S          0.061212        1.957983        0.027202
```

7_transtrans24hexadienal_TS_3

Datum	Value
M06-2X/def2tzvpp-IEFPCM(water) Energy	-746.826415
M06-2X/def2tzvpp-IEFPCM(water) Free Energy (Quasiharmonic)	-746.698285
Number of Imaginary Frequencies	1

Frequencies (Top 3 out of 54)

```
  1.    -207.2817 cm-1
  2.      50.4696 cm-1
  3.      70.6866 cm-1
```

M06-2X/def2tzvpp-IEFPCM(water) Molecular Geometry in Cartesian Coordinates

```
C          2.475052        0.940885       -0.237570
C          1.100289        0.882224        0.074546
C          0.205586        0.323135       -0.828037
C         -1.249384        0.508653       -0.753750
C         -1.901911        1.139816        0.223116
C         -3.387966        1.297863        0.275633
```

```
O          3.385956        1.389493        0.470846
H          2.733477        0.539235       -1.238568
H          0.782395        1.225164        1.051360
H          0.589396        0.120561       -1.820049
H         -1.819369        0.081376       -1.571525
H         -1.340620        1.575450        1.043488
H         -3.863253        0.848948       -0.595848
H         -3.799493        0.827179        1.171346
H         -3.670095        2.351847        0.319058
C         -0.042790       -1.784347        1.306599
H         -0.867001       -1.106068        1.546521
H         -0.192307       -2.714940        1.851846
H          0.885372       -1.325645        1.650816
S          0.017537       -2.086777       -0.471152
```

7_transtrans24hexadienal_TS_4_reopt

Datum	Value
M06-2X/def2tzvpp-IEFPCM(water) Energy	-746.825805
M06-2X/def2tzvpp-IEFPCM(water) Free Energy (Quasiharmonic)	-746.697552
Number of Imaginary Frequencies	1

Frequencies (Top 3 out of 54)

```
1.    -150.2527 cm-1
2.      48.7696 cm-1
3.      74.9158 cm-1
```

M06-2X/def2tzvpp-IEFPCM(water) Molecular Geometry in Cartesian Coordinates

```
C         -1.661197       -1.866867        0.301472
C         -0.390260       -1.244894        0.469398
C          0.160655       -0.468976       -0.523238
C          1.554302       -0.007530       -0.535536
C          2.524376       -0.466967        0.253922
C          3.941371        0.007369        0.213673
O         -2.383791       -1.806334       -0.697389
H         -2.012185       -2.455811        1.169134
H          0.087502       -1.345636        1.435693
H         -0.335052       -0.495884       -1.483538
H          1.796545        0.747155       -1.276935
H          2.292940       -1.241395        0.978746
H          4.237748        0.428707        1.176835
```

```
H           4.625960       -0.818480        0.010087
H           4.081263        0.769505       -0.551981
C          -2.037024        1.495137        0.914473
H          -3.062442        1.546548        0.548583
H          -1.859479        0.472944        1.275181
H          -1.938095        2.169005        1.764874
S          -0.837357        1.874398       -0.377034
```

7_transtrans24hexadienal_TS_5

Datum	Value
M06-2X/def2tzvpp-IEFPCM(water) Energy	-746.828331
M06-2X/def2tzvpp-IEFPCM(water) Free Energy (Quasiharmonic)	-746.700822
Number of Imaginary Frequencies	1

Frequencies (Top 3 out of 54)

```
1.    -173.6150 cm-1
2.      54.0252 cm-1
3.      71.3459 cm-1
```

M06-2X/def2tzvpp-IEFPCM(water) Molecular Geometry in Cartesian Coordinates

```
C          -2.150567        1.386799        0.334653
C          -0.958235        1.105840       -0.376143
C           0.116066        0.499240        0.249844
C           1.464365        0.566017       -0.316530
C           2.577818        0.522896        0.412012
C           3.963331        0.626177       -0.138290
O          -3.158691        1.956437       -0.097958
H          -2.135380        1.062745        1.394849
H          -0.907083        1.366208       -1.428075
H           0.066700        0.389650        1.326616
H           1.528999        0.687588       -1.394651
H           2.486350        0.398538        1.488317
H           3.948816        0.751585       -1.220282
H           4.497908        1.471117        0.300284
H           4.543892       -0.268286        0.097531
C          -1.855589       -2.048185       -0.121126
H          -2.237270       -1.036649       -0.310864
H          -2.160814       -2.677462       -0.956499
H          -2.328619       -2.424228        0.785696
S          -0.061069       -1.957812        0.027139
```

7_transtrans24hexadienal_TS_6_reopt

Datum	Value
M06-2X/def2tzvpp-IEFPCM(water) Energy	-746.82759
M06-2X/def2tzvpp-IEFPCM(water) Free Energy (Quasiharmonic)	-746.699552
Number of Imaginary Frequencies	1

Frequencies (Top 3 out of 54)

```
1.    -167.2593 cm-1
2.      64.6068 cm-1
3.      74.1039 cm-1
```

M06-2X/def2tzvpp-IEFPCM(water) Molecular Geometry in Cartesian Coordinates

```
C         -2.028910       -1.679916        0.227778
C         -0.790071       -1.209275        0.751429
C          0.165365       -0.634750       -0.055301
C          1.529590       -0.400378        0.407849
C          2.585558       -0.343083       -0.402499
C          3.996757       -0.144384        0.045612
O         -2.392180       -1.633141       -0.951075
H         -2.710640       -2.131494        0.971941
H         -0.637837       -1.277582        1.821877
H          0.029171       -0.720579       -1.125058
H          1.669366       -0.304001        1.481629
H          2.420611       -0.440314       -1.472713
H          4.630162       -0.975012       -0.272199
H          4.421493        0.759609       -0.396323
H          4.057842       -0.059106        1.129829
C         -2.110846        1.720503        0.430572
H         -2.220180        0.733268        0.900731
H         -2.262618        2.472710        1.204207
H         -2.900530        1.824136       -0.313990
S         -0.465254        1.833200       -0.297124
```

7_transtrans24hexadienal_TS_7_reopt

Datum	Value
M06-2X/def2tzvpp-IEFPCM(water) Energy	-746.825058
M06-2X/def2tzvpp-IEFPCM(water) Free Energy (Quasiharmonic)	-746.697095
Number of Imaginary Frequencies	1

Frequencies (Top 3 out of 54)

```
1.    -185.3946 cm-1
2.      33.1332 cm-1
3.      63.9940 cm-1
```

M06-2X/def2tzvpp-IEFPCM(water) Molecular Geometry in Cartesian Coordinates

```
C          2.420591       -1.222613       -0.527066
C          1.004908       -1.080234       -0.522322
C          0.338607       -0.581216        0.577002
C         -1.116672       -0.608409        0.740975
C         -1.996686       -0.998643       -0.182135
C         -3.478345       -0.993217        0.014623
O          3.196512       -0.921640        0.385320
H          2.843931       -1.650276       -1.454825
H          0.481334       -1.345666       -1.430603
H          0.915384       -0.485798        1.486055
H         -1.487314       -0.264069        1.700556
H         -1.640372       -1.337529       -1.149824
H         -3.741416       -0.666072        1.019914
H         -3.963470       -0.325861       -0.701639
H         -3.901305       -1.986649       -0.148357
C         -0.386559        1.900258       -1.129173
H         -1.471754        1.809687       -1.073183
H         -0.143706        2.796237       -1.699118
H          0.000136        1.035176       -1.677950
S          0.363837        1.956149        0.513437
```

7_transtrans24hexadienal_TS_8

Datum	Value
M06-2X/def2tzvpp-IEFPCM(water) Energy	-746.825805
M06-2X/def2tzvpp-IEFPCM(water) Free Energy (Quasiharmonic)	-746.697552
Number of Imaginary Frequencies	1

Frequencies (Top 3 out of 54)

```
1.    -150.2141 cm-1
2.      48.8089 cm-1
3.      74.9406 cm-1
```

M06-2X/def2tzvpp-IEFPCM(water) Molecular Geometry in Cartesian Coordinates

```
C          1.661036        1.866910        0.301515
C          0.390071        1.244918        0.469412
C         -0.160790        0.469067       -0.523296
C         -1.554355        0.007353       -0.535458
C         -2.524532        0.466962        0.253778
C         -3.941430       -0.007685        0.213738
O          2.383552        1.806481       -0.697393
H          2.012082        2.455747        1.169224
H         -0.087620        1.345456        1.435763
H          0.334947        0.496156       -1.483574
H         -1.796457       -0.747686       -1.276541
H         -2.293244        1.241742        0.978273
H         -4.081246       -0.769921       -0.551830
H         -4.237586       -0.429017        1.176973
H         -4.626230        0.817993        0.010177
C          2.037274       -1.495016        0.914483
H          1.859565       -0.472912        1.275361
H          1.938443       -2.169053        1.764764
H          3.062707       -1.546208        0.548605
S          0.837661       -1.874325       -0.377068
```

7_transtrans24hexadienal_TS_9

Datum	Value
M06-2X/def2tzvpp-IEFPCM(water) Energy	-746.823513
M06-2X/def2tzvpp-IEFPCM(water) Free Energy (Quasiharmonic)	-746.695729
Number of Imaginary Frequencies	1

Frequencies (Top 3 out of 54)

```
1.    -173.9639 cm-1
2.      49.2037 cm-1
3.      69.7490 cm-1
```

M06-2X/def2tzvpp-IEFPCM(water) Molecular Geometry in Cartesian Coordinates

```
C          1.168410        2.114141       -0.035225
C          0.842226        1.167849       -1.051076
C         -0.133566        0.195636       -0.974358
C         -1.291105        0.238773       -0.082351
C         -2.447697       -0.350830       -0.385883
C         -3.674494       -0.305844        0.466276
O          0.682391        2.210206        1.094739
H          1.965905        2.825265       -0.323370
H          1.486111        1.182553       -1.921444
H         -0.276829       -0.415036       -1.857550
H         -1.189243        0.809857        0.831390
H         -2.517078       -0.916146       -1.312464
H         -3.506053        0.283550        1.366730
H         -4.516702        0.126884       -0.077540
H         -3.978747       -1.311705        0.764494
C          2.634981       -1.125787        0.307193
H          3.001808       -1.101083        1.333133
H          3.348988       -1.681803       -0.300458
H          2.611789       -0.091455       -0.063291
S          0.969900       -1.812260        0.208437
```

Created using ESIgen v0.0.5

ESIgen is scientific software, funded by public research grants and published as:

```
J Rodriguez-Guerra, P Gomez-Orellana, JD Marechal.
J. Chem. Inf. Model., 2018, 58 (3), pp 561564.
DOI: 10.1021/acs.jcim.7b00714.
```

If you make use of ESIgen in scientific publications, please cite us in the main text! References only mentioned in SI documents are not indexed by citation engines.

1_methylacrylate_1

Datum	Value
M06-2X/def2tzvpp-IEFPCM(water) Energy	-306.469104
M06-2X/def2tzvpp-IEFPCM(water) Free Energy (Quasiharmonic)	-306.399991
Number of Imaginary Frequencies	0

Frequencies (Top 3 out of 30)

```
  1.      80.7656 cm-1
  2.     160.2674 cm-1
  3.     195.0324 cm-1
```

M06-2X/def2tzvpp-IEFPCM(water) Molecular Geometry in Cartesian Coordinates

```
C         -2.476962       -0.006325        0.000003
C         -1.316811       -0.645028       -0.000009
C         -0.040162        0.110489        0.000002
O          1.009225       -0.713357       -0.000013
O          0.061530        1.312988        0.000021
C          2.294302       -0.087679       -0.000003
H          3.018354       -0.894679       -0.000013
H          2.411071        0.529802       -0.887953
H          2.411069        0.529779        0.887962
H         -2.508004        1.075665        0.000020
H         -3.415308       -0.542627       -0.000005
H         -1.245427       -1.723726       -0.000026
```

1_methylacrylate_2

Datum	Value
M06-2X/def2tzvpp-IEFPCM(water) Energy	-306.468483
M06-2X/def2tzvpp-IEFPCM(water) Free Energy (Quasiharmonic)	-306.399337
Number of Imaginary Frequencies	0

Frequencies (Top 3 out of 30)

```
1.      82.5695 cm-1
2.     172.8395 cm-1
3.     185.6286 cm-1
```

M06-2X/def2tzvpp-IEFPCM(water) Molecular Geometry in Cartesian Coordinates

```
C         -2.153096       -0.765729       -0.000031
C         -1.489811        0.381469        0.000010
C         -0.011465        0.477417        0.000017
O          0.601820       -0.705129       -0.000023
O          0.581920        1.529935        0.000056
C          2.030018       -0.665791       -0.000021
H          2.391924       -0.151988       -0.887941
H          2.391922       -0.152050        0.887937
H          2.355729       -1.699935       -0.000056
H         -1.635042       -1.714906       -0.000061
H         -3.234218       -0.781858       -0.000034
H         -1.994120        1.338091        0.000041
```

1_methylacrylate_3

Datum	Value
M06-2X/def2tzvpp-IEFPCM(water) Energy	-306.460181
M06-2X/def2tzvpp-IEFPCM(water) Free Energy (Quasiharmonic)	-306.390271
Number of Imaginary Frequencies	1

Frequencies (Top 3 out of 30)

```
1.     -25.4437 cm-1
2.     139.1780 cm-1
3.     252.3368 cm-1
```

M06-2X/def2tzvpp-IEFPCM(water) Molecular Geometry in Cartesian Coordinates

```
C        -2.137839      -0.758454       0.003226
C        -0.812182      -0.766893      -0.004752
C        -0.069244       0.523731      -0.001633
O         1.268172       0.493218      -0.000117
O        -0.614944       1.599487      -0.000532
C         1.992738      -0.738761       0.002456
H         1.768593      -1.320110       0.894567
H         1.777397      -1.318609      -0.892799
H         3.040571      -0.458560       0.007825
H        -2.683613       0.175710       0.012298
H        -2.702684      -1.680033       0.001086
H        -0.266941      -1.697777      -0.013564
```

1_methylacrylate_HEI_1_reopt

Datum	Value
M06-2X/def2tzvpp-IEFPCM(water) Energy	-744.684095
M06-2X/def2tzvpp-IEFPCM(water) Free Energy (Quasiharmonic)	-744.581103
Number of Imaginary Frequencies	0

Frequencies (Top 3 out of 45)

```
1.      44.7959 cm-1
2.      78.8121 cm-1
3.     105.7983 cm-1
```

M06-2X/def2tzvpp-IEFPCM(water) Molecular Geometry in Cartesian Coordinates

```
C         1.039376      -0.873494      -0.849006
C        -0.053655      -1.334729       0.026657
C        -1.250960      -0.668336       0.203676
O        -1.389293       0.476726      -0.589112
```

```
O        -2.207128      -0.967962       0.955479
C        -2.590799       1.197238      -0.425108
H        -2.717249       1.541066       0.602354
H        -2.524926       2.057134      -1.088864
H        -3.462200       0.598783      -0.694062
H         0.681832      -0.199077      -1.624318
H         1.571211      -1.693333      -1.334424
H         0.106630      -2.208264       0.645092
S         2.444521       0.031742      -0.004941
C         1.521067       1.398293       0.715839
H         0.660345       1.000728       1.252822
H         2.174599       1.922115       1.409775
H         1.178620       2.089032      -0.052592
```

1_methylacrylate_HEI_2

Datum	Value
M06-2X/def2tzvpp-IEFPCM(water) Energy	-744.685464
M06-2X/def2tzvpp-IEFPCM(water) Free Energy (Quasiharmonic)	-744.58318
Number of Imaginary Frequencies	0

Frequencies (Top 3 out of 45)

```
1.      35.6100 cm-1
2.      66.9105 cm-1
3.      96.9908 cm-1
```

M06-2X/def2tzvpp-IEFPCM(water) Molecular Geometry in Cartesian Coordinates

```
C         1.183137      -1.099269       0.497142
C        -0.095081      -1.069628      -0.234520
C        -1.135533      -0.263142       0.180491
O        -2.257779      -0.339622      -0.656425
O        -1.205653       0.490096       1.177248
C        -3.373319       0.426198      -0.258067
H        -4.145157       0.255733      -1.006062
H        -3.745194       0.119862       0.720784
H        -3.139307       1.490740      -0.215946
H         1.635096      -2.091210       0.545309
H         1.056831      -0.733217       1.515522
H        -0.204629      -1.637910      -1.147652
S         2.572683      -0.085144      -0.235459
C         1.787265       1.534745      -0.211919
```

```
H          1.717319        1.921885        0.803081
H          2.383382        2.213340       -0.817872
H          0.787372        1.445859       -0.635158
```

1_methylacrylate_HEI_3_reopt

Datum	Value
M06-2X/def2tzvpp-IEFPCM(water) Energy	-744.683474
M06-2X/def2tzvpp-IEFPCM(water) Free Energy (Quasiharmonic)	-744.581113
Number of Imaginary Frequencies	0

Frequencies (Top 3 out of 45)

```
1.      37.4404 cm-1
2.      70.8745 cm-1
3.      95.7013 cm-1
```

M06-2X/def2tzvpp-IEFPCM(water) Molecular Geometry in Cartesian Coordinates

```
C          0.870181       -0.062729        1.100380
C         -0.073867        0.952819        0.597169
C         -1.323229        0.658925        0.084216
O         -1.631625       -0.704953        0.108011
O         -2.185868        1.439827       -0.376770
C         -2.919188       -1.052653       -0.353261
H         -3.065648       -0.762338       -1.394091
H         -2.994396       -2.134560       -0.264930
H         -3.701999       -0.584864        0.245213
H          0.364154       -0.930059        1.521466
H          1.537472        0.341393        1.862801
H          0.233533        1.988643        0.560670
S          2.021638       -0.842733       -0.153542
C          2.885084        0.645792       -0.683176
H          3.463142        1.070169        0.137134
H          3.558804        0.381932       -1.495059
H          2.164790        1.381489       -1.038426
```

1_methylacrylate_HEI_4

Datum	Value
M06-2X/def2tzvpp-IEFPCM(water) Energy	-744.684881
M06-2X/def2tzvpp-IEFPCM(water) Free Energy (Quasiharmonic)	-744.58279
Number of Imaginary Frequencies	0

Frequencies (Top 3 out of 45)

```
1.      42.6312 cm-1
2.      60.9345 cm-1
3.      84.9518 cm-1
```

M06-2X/def2tzvpp-IEFPCM(water) Molecular Geometry in Cartesian Coordinates

```
C        -1.069403       -0.593244        0.945556
C         0.118795        0.271835        0.846925
C         1.254007       -0.164245        0.193318
O         2.284055        0.783685        0.173644
O         1.466365       -1.268158       -0.355042
C         3.484180        0.374073       -0.444844
H         4.169608        1.216043       -0.371461
H         3.921456       -0.491980        0.054284
H         3.332428        0.122023       -1.495249
H        -1.658537       -0.394318        1.841585
H        -0.800055       -1.649549        0.941577
H         0.090020        1.278173        1.238579
S        -2.305348       -0.490927       -0.457398
C        -2.736445        1.250802       -0.305730
H        -1.837088        1.860517       -0.381043
H        -3.413748        1.508555       -1.116610
H        -3.228685        1.445820        0.646539
```

1_methylacrylate_HEI_5_reopt3

Datum	Value
M06-2X/def2tzvpp-IEFPCM(water) Energy	-744.685041
M06-2X/def2tzvpp-IEFPCM(water) Free Energy (Quasiharmonic)	-744.581803
Number of Imaginary Frequencies	0

Frequencies (Top 3 out of 45)

```
1.      36.2445 cm-1
2.      63.4740 cm-1
3.      98.6268 cm-1
```

M06-2X/def2tzvpp-IEFPCM(water) Molecular Geometry in Cartesian Coordinates

```
C          0.963611      -0.521287       1.088712
C         -0.380973      -0.643781       0.486384
C         -1.188192       0.467002       0.350687
O         -2.451551       0.328898      -0.232664
O         -0.948820       1.646463       0.705407
C         -2.868816      -0.936559      -0.686585
H         -2.206848      -1.323449      -1.465295
H         -3.867052      -0.808802      -1.100277
H         -2.910516      -1.662724       0.128994
H          1.052656       0.403274       1.657134
H          1.215623      -1.353317       1.748376
H         -0.683923      -1.611409       0.120225
S          2.386567      -0.535888      -0.109023
C          1.947700       0.898814      -1.104262
H          2.104076       1.822588      -0.550239
H          2.573745       0.899688      -1.993680
H          0.900155       0.820337      -1.392447
```

1_methylacrylate_HEI_6

Datum	Value
M06-2X/def2tzvpp-IEFPCM(water) Energy	-744.684949
M06-2X/def2tzvpp-IEFPCM(water) Free Energy (Quasiharmonic)	-744.583013
Number of Imaginary Frequencies	0

Frequencies (Top 3 out of 45)

```
1.      51.5583 cm-1
2.      61.2358 cm-1
3.      82.4448 cm-1
```

M06-2X/def2tzvpp-IEFPCM(water) Molecular Geometry in Cartesian Coordinates

```
C        -1.021142      -0.417776       0.772622
C         0.321814      -0.894089       0.381916
C         1.363296      -0.004678       0.215682
O         2.559517      -0.619784      -0.173657
O         1.370984       1.236494       0.374876
C         3.662595       0.241396      -0.352009
H         3.471004       0.988986      -1.123024
H         4.496231      -0.387480      -0.658053
H         3.922612       0.762143       0.570590
H        -1.569600      -1.140581       1.378967
H        -0.963208       0.522797       1.321166
H         0.481691      -1.941019       0.167721
S        -2.103778      -0.084279      -0.694445
C        -3.625796       0.416747       0.139063
H        -3.458165       1.308749       0.740991
H        -4.374866       0.636792      -0.618350
H        -3.993858      -0.385210       0.777715
```

1_methylacrylate_HEI_7

Datum	Value
M06-2X/def2tzvpp-IEFPCM(water) Energy	-744.683508
M06-2X/def2tzvpp-IEFPCM(water) Free Energy (Quasiharmonic)	-744.581474
Number of Imaginary Frequencies	0

Frequencies (Top 3 out of 45)

```
1.     48.7548 cm-1
2.     64.8197 cm-1
3.     85.4350 cm-1
```

M06-2X/def2tzvpp-IEFPCM(water) Molecular Geometry in Cartesian Coordinates

```
C        -0.874293      -0.501422       0.765139
C         0.347337      -1.214304       0.331215
C         1.543130      -0.592985       0.028515
O         1.516399       0.795642       0.198070
O         2.617095      -1.108068      -0.357777
C         2.734955       1.468188      -0.033791
H         2.541252       2.523344       0.148684
H         3.520991       1.122689       0.639149
H         3.080718       1.335521      -1.059510
```

```
H          -0.644403       0.440902        1.260408
H          -1.489461      -1.102582        1.437137
H           0.301948      -2.283450        0.175677
S          -1.985383      -0.071895       -0.655671
C          -3.362018       0.687349        0.233625
H          -3.026492       1.566039        0.782693
H          -4.113476       0.989847       -0.492378
H          -3.807561      -0.023541        0.928324
```

1_methylacrylate_HEI_8

Datum	Value
M06-2X/def2tzvpp-IEFPCM(water) Energy	-744.684708
M06-2X/def2tzvpp-IEFPCM(water) Free Energy (Quasiharmonic)	-744.581017
Number of Imaginary Frequencies	0

Frequencies (Top 3 out of 45)

```
1.      60.2485 cm-1
2.      71.4733 cm-1
3.      96.9943 cm-1
```

M06-2X/def2tzvpp-IEFPCM(water) Molecular Geometry in Cartesian Coordinates

```
C          -0.922844      -0.523615        0.981867
C           0.363955       0.121206        0.646300
C           1.329903      -0.595326       -0.031157
O           2.542716       0.014978       -0.354943
O           1.269876      -1.788973       -0.410400
C           2.752074       1.360163        0.003917
H           2.018344       2.019819       -0.466206
H           3.746859       1.628096       -0.345786
H           2.703771       1.498873        1.086760
H          -0.810887      -1.600485        1.104075
H          -1.363698      -0.118762        1.893799
H           0.501107       1.158091        0.903746
S          -2.270783      -0.391644       -0.296323
C          -2.442643       1.400772       -0.319850
H          -1.488078       1.861774       -0.569715
H          -3.176992       1.663676       -1.077667
H          -2.781310       1.767990        0.648431
```

1_methylacrylate_TS_1_reopt

Datum	Value
M06-2X/def2tzvpp-IEFPCM(water) Energy	-744.675795
M06-2X/def2tzvpp-IEFPCM(water) Free Energy (Quasiharmonic)	-744.573834
Number of Imaginary Frequencies	1

Frequencies (Top 3 out of 45)

```
1.    -197.5075 cm-1
2.      35.7684 cm-1
3.      60.6869 cm-1
```

M06-2X/def2tzvpp-IEFPCM(water) Molecular Geometry in Cartesian Coordinates

```
C         -0.776248        1.271174       -0.836549
C          0.164355        1.395509        0.163366
C          1.317395        0.557329        0.278639
O          1.404303       -0.396441       -0.690245
O          2.188432        0.641199        1.139168
C          2.542559       -1.242180       -0.625403
H          2.563783       -1.794655        0.313075
H          2.453953       -1.931584       -1.459894
H          3.462587       -0.666120       -0.714813
H         -0.525578        0.715886       -1.726490
H         -1.498474        2.061713       -0.974142
H          0.031771        2.103838        0.969435
S         -2.515794       -0.280045       -0.293589
C         -1.671733       -1.046191        1.102137
H         -0.793602       -0.436112        1.351884
H         -2.311515       -1.089243        1.982434
H         -1.330063       -2.054922        0.871404
```

1_methylacrylate_TS_2

Datum	Value
M06-2X/def2tzvpp-IEFPCM(water) Energy	-744.676976
M06-2X/def2tzvpp-IEFPCM(water) Free Energy (Quasiharmonic)	-744.575035

Datum	Value
Number of Imaginary Frequencies	1

Frequencies (Top 3 out of 45)

```
1.    -183.1926 cm-1
2.      36.8285 cm-1
3.      62.1603 cm-1
```

M06-2X/def2tzvpp-IEFPCM(water) Molecular Geometry in Cartesian Coordinates

```
C          0.973655       -1.522416       -0.111344
C         -0.156295       -1.014446       -0.709225
C         -1.174331       -0.392904        0.081279
O         -2.219571        0.056454       -0.671535
O         -1.180453       -0.247386        1.297699
C         -3.271470        0.687298        0.043345
H         -4.006305        0.984214       -0.699139
H         -3.722572        0.001433        0.759242
H         -2.907516        1.563864        0.577846
H          1.645114       -2.143533       -0.683997
H          0.953082       -1.710428        0.951282
H         -0.269405       -0.986731       -1.783498
S          2.681662        0.148159        0.191234
C          1.673852        1.559343       -0.297695
H          1.470615        2.225757        0.540164
H          2.145515        2.135342       -1.092758
H          0.712609        1.185738       -0.676360
```

1_methylacrylate_TS_3

Datum	Value
M06-2X/def2tzvpp-IEFPCM(water) Energy	-744.675796
M06-2X/def2tzvpp-IEFPCM(water) Free Energy (Quasiharmonic)	-744.573833
Number of Imaginary Frequencies	1

Frequencies (Top 3 out of 45)

```
1.    -197.4724 cm-1
2.      35.7148 cm-1
```

```
   3.      60.7509 cm-1
```

M06-2X/def2tzvpp-IEFPCM(water) Molecular Geometry in Cartesian Coordinates

```
C          0.776154        1.270970        0.836846
C         -0.164375        1.395449       -0.163126
C         -1.317410        0.557294       -0.278580
O         -1.404329       -0.396664        0.690119
O         -2.188431        0.641317       -1.139113
C         -2.542659       -1.242297        0.625185
H         -2.564012       -1.794563       -0.313411
H         -2.454040       -1.931890        1.459518
H         -3.462631       -0.666177        0.714802
H          0.525373        0.715623        1.726718
H          1.498396        2.061470        0.974572
H         -0.031733        2.103887       -0.969088
S          2.515598       -0.280340        0.293930
C          1.672278       -1.045297       -1.102893
H          2.312335       -1.087193       -1.983047
H          1.330893       -2.054389       -0.873330
H          0.793999       -0.435264       -1.352252
```

1_methylacrylate_TS_4_reopt

Datum	Value
M06-2X/def2tzvpp-IEFPCM(water) Energy	-744.676976
M06-2X/def2tzvpp-IEFPCM(water) Free Energy (Quasiharmonic)	-744.575035
Number of Imaginary Frequencies	1

Frequencies (Top 3 out of 45)

```
   1.    -183.2223 cm-1
   2.      36.7422 cm-1
   3.      62.1898 cm-1
```

M06-2X/def2tzvpp-IEFPCM(water) Molecular Geometry in Cartesian Coordinates

```
C          0.973727       -1.522436       -0.110974
C         -0.156310       -1.014789       -0.708972
```

```
C          -1.174265       -0.392935        0.081364
O          -2.219550        0.056181       -0.671547
O          -1.180315       -0.246990        1.297718
C          -3.271381        0.687319        0.043172
H          -4.006167        0.984159       -0.699392
H          -3.722588        0.001669        0.759213
H          -2.907333        1.563960        0.577487
H           1.645156       -2.143769       -0.683432
H           0.953169       -1.710134        0.951712
H          -0.269471       -0.987431       -1.783249
S           2.681641        0.148260        0.190980
C           1.673581        1.559350       -0.297690
H           0.712504        1.185643       -0.676667
H           1.470020        2.225434        0.540352
H           2.145265        2.135720       -1.092471
```

1_methylacrylate_TS_5_reopt

Datum	Value
M06-2X/def2tzvpp-IEFPCM(water) Energy	-744.672055
M06-2X/def2tzvpp-IEFPCM(water) Free Energy (Quasiharmonic)	-744.569857
Number of Imaginary Frequencies	1

Frequencies (Top 3 out of 45)

```
1.    -156.2791 cm-1
2.      34.9206 cm-1
3.      50.6356 cm-1
```

M06-2X/def2tzvpp-IEFPCM(water) Molecular Geometry in Cartesian Coordinates

```
C           0.653206       -1.272157       -0.948381
C          -0.481016       -0.526058       -0.853946
C          -1.230030       -0.524642        0.383220
O          -2.363429        0.220732        0.468546
O          -0.928009       -1.152386        1.385228
C          -2.806916        0.994076       -0.637777
H          -2.075480        1.758115       -0.901668
H          -3.727382        1.473423       -0.318310
H          -3.009408        0.364151       -1.503530
H           0.872411       -2.013616       -0.197030
H           1.165531       -1.367597       -1.892585
H          -0.776222        0.117276       -1.666883
```

```
S          2.667395      0.055107     -0.032344
C          1.675794      1.478676      0.467326
H          1.684803      1.622663      1.547720
H          2.020055      2.399077     -0.003838
H          0.632651      1.318655      0.160775
```

1_methylacrylate_TS_6_reopt

Datum	Value
M06-2X/def2tzvpp-IEFPCM(water) Energy	-744.672076
M06-2X/def2tzvpp-IEFPCM(water) Free Energy (Quasiharmonic)	-744.570571
Number of Imaginary Frequencies	2

Frequencies (Top 3 out of 45)

```
1.    -222.3206 cm-1
2.      -4.1784 cm-1
3.      38.6979 cm-1
```

M06-2X/def2tzvpp-IEFPCM(water) Molecular Geometry in Cartesian Coordinates

```
C          0.801005      0.895433      0.498157
C         -0.453855      1.079573     -0.033651
C         -1.481273      0.112130      0.192824
O         -2.659181      0.451534     -0.406031
O         -1.393325     -0.923909      0.841304
C         -3.736643     -0.452305     -0.212025
H         -3.496027     -1.437050     -0.610332
H         -4.583307     -0.032868     -0.747440
H         -3.977526     -0.550336      0.845740
H          1.512217      1.708589      0.481911
H          0.920449      0.189302      1.308284
H         -0.671809      1.898463     -0.703667
S          2.372858     -0.426317     -0.740896
C          3.683855     -0.130873      0.479115
H          4.280785     -1.026399      0.645725
H          4.351808      0.672015      0.168468
H          3.239193      0.154605      1.436938
```

1_methylacrylate_TS_7_reopt

Datum	Value
M06-2X/def2tzvpp-IEFPCM(water) Energy	-744.67108
M06-2X/def2tzvpp-IEFPCM(water) Free Energy (Quasiharmonic)	-744.570567
Number of Imaginary Frequencies	1

Frequencies (Top 3 out of 45)

```
1.    -222.6992 cm-1
2.      21.4457 cm-1
3.      49.9683 cm-1
```

M06-2X/def2tzvpp-IEFPCM(water) Molecular Geometry in Cartesian Coordinates

```
C         -0.669978       -0.761509        0.739626
C          0.487415       -1.318569        0.242845
C          1.679322       -0.572821       -0.013479
O          1.578454        0.751554        0.289903
O          2.729756       -1.023361       -0.459514
C          2.748721        1.526555        0.076529
H          2.495578        2.541493        0.368612
H          3.575619        1.159728        0.683253
H          3.044511        1.503915       -0.971347
H         -0.628019        0.201545        1.227259
H         -1.459463       -1.415407        1.082740
H          0.517093       -2.353302       -0.068622
S         -2.230055        0.197361       -0.800387
C         -3.448942        0.441078        0.521823
H         -4.086563        1.300060        0.318079
H         -4.085870       -0.434220        0.648628
H         -2.936910        0.624461        1.470424
```

1_methylacrylate_TS_8_reopt

Datum	Value
M06-2X/def2tzvpp-IEFPCM(water) Energy	-744.672055
M06-2X/def2tzvpp-IEFPCM(water) Free Energy (Quasiharmonic)	-744.569858
Number of Imaginary Frequencies	1

Frequencies (Top 3 out of 45)

```
1.    -156.2834 cm-1
2.      34.8289 cm-1
3.      50.6087 cm-1
```

M06-2X/def2tzvpp-IEFPCM(water) Molecular Geometry in Cartesian Coordinates

```
C          0.653058       -1.272234       -0.948358
C         -0.481154       -0.526132       -0.853833
C         -1.229994       -0.524614        0.383438
O         -2.363381        0.220764        0.468863
O         -0.927837       -1.152285        1.385452
C         -2.806998        0.994065       -0.637438
H         -2.075579        1.758074       -0.901462
H         -3.727410        1.473448       -0.317871
H         -3.009621        0.364104       -1.503134
H          0.872382       -2.013599       -0.196948
H          1.165233       -1.367787       -1.892632
H         -0.776495        0.117108       -1.666797
S          2.667419        0.055130       -0.032826
C          1.675876        1.478653        0.467093
H          0.632721        1.318717        0.160543
H          1.684917        1.622471        1.547510
H          2.020158        2.399116       -0.003935
```

2_tert-butylacrylate_1

Datum	Value
M06-2X/def2tzvpp-IEFPCM(water) Energy	-424.402886
M06-2X/def2tzvpp-IEFPCM(water) Free Energy (Quasiharmonic)	-424.254255
Number of Imaginary Frequencies	0

Frequencies (Top 3 out of 57)

```
1.      65.9392 cm-1
2.      86.3067 cm-1
3.     135.2275 cm-1
```

M06-2X/def2tzvpp-IEFPCM(water) Molecular Geometry in Cartesian Coordinates

```
C          3.473369       0.195015      -0.000000
C          2.268878       0.745238       0.000000
C          1.047083      -0.104774      -0.000000
O         -0.046208       0.654047       0.000001
O          1.057747      -1.312329      -0.000001
C         -1.389415       0.075262       0.000000
C         -2.288478       1.301331       0.000002
C         -1.605051      -0.742835      -1.264997
C         -1.605051      -0.742838       1.264996
H          3.584402      -0.881767      -0.000001
H          4.369460       0.799456       0.000000
H          2.119222       1.815928       0.000001
H         -2.655135      -1.029248      -1.323432
H         -1.364673      -0.144117      -2.143926
H         -0.996588      -1.642712      -1.266947
H         -3.332427       0.990762       0.000001
H         -2.104093       1.907821       0.886324
H         -2.104093       1.907823      -0.886320
H         -2.655135      -1.029250       1.323431
H         -0.996588      -1.642715       1.266944
H         -1.364672      -0.144121       2.143926
```

2_tert-butylacrylate_2

Datum	Value
M06-2X/def2tzvpp-IEFPCM(water) Energy	-424.402309
M06-2X/def2tzvpp-IEFPCM(water) Free Energy (Quasiharmonic)	-424.253871
Number of Imaginary Frequencies	0

Frequencies (Top 3 out of 57)

```
1.      46.5934 cm-1
2.      97.3909 cm-1
3.     119.6199 cm-1
```

M06-2X/def2tzvpp-IEFPCM(water) Molecular Geometry in Cartesian Coordinates

```
C         -2.830945       1.169512      -0.000002
C         -2.473274      -0.106303      -0.000001
C         -1.063511      -0.576574       0.000000
O         -0.185476       0.419796      -0.000001
O         -0.782025      -1.752804       0.000002
```

```
C          1.257322       0.180170       0.000000
C          1.833875       1.586906      -0.000002
C          1.664079      -0.562211       1.264624
C          1.664080      -0.562214      -1.264622
H         -2.092236       1.959305      -0.000002
H         -3.874175       1.454147      -0.000002
H         -3.201192      -0.906538       0.000000
H          2.752293      -0.586422       1.323138
H          1.286055      -0.039984       2.143905
H          1.291396      -1.582663       1.265736
H          2.921916       1.537966      -0.000001
H          1.508226       2.130811      -0.886344
H          1.508225       2.130814       0.886338
H          2.752294      -0.586426      -1.323135
H          1.291397      -1.582667      -1.265730
H          1.286057      -0.039991      -2.143904
```

2_tert-butylacrylate_3

Datum	Value
M06-2X/def2tzvpp-IEFPCM(water) Energy	-424.390976
M06-2X/def2tzvpp-IEFPCM(water) Free Energy (Quasiharmonic)	-424.241606
Number of Imaginary Frequencies	1

Frequencies (Top 3 out of 57)

```
  1.     -13.0175 cm-1
  2.      67.5495 cm-1
  3.     117.3289 cm-1
```

M06-2X/def2tzvpp-IEFPCM(water) Molecular Geometry in Cartesian Coordinates

```
C          2.972020      -0.888454       0.000959
C          1.657588      -0.715065      -0.000644
C          1.096064       0.666021      -0.000463
O         -0.221986       0.869902      -0.000744
O          1.810934       1.641055      -0.000175
C         -1.310778      -0.106578      -0.000012
C         -1.298708      -0.938737      -1.275503
C         -1.296968      -0.938928       1.275341
C         -2.540428       0.793407       0.000887
H          3.643146      -0.040265       0.002457
H          3.401711      -1.880357       0.000821
```

```
H          0.997835      -1.564131      -0.002125
H         -0.478879      -1.652558       1.320077
H         -1.237130      -0.283017       2.143583
H         -2.228957      -1.500445       1.335195
H         -2.230604      -1.500553      -1.334000
H         -1.240414      -0.282677      -2.143734
H         -0.480453      -1.652088      -1.321631
H         -3.443281       0.184627       0.001348
H         -2.543106       1.426777       0.887122
H         -2.544196       1.427039      -0.885156
```

2_tertbutylacrylate_HEI_1_reopt

Datum	Value
M06-2X/def2tzvpp-IEFPCM(water) Energy	-862.61594
M06-2X/def2tzvpp-IEFPCM(water) Free Energy (Quasiharmonic)	-862.433315
Number of Imaginary Frequencies	0

Frequencies (Top 3 out of 72)

```
1.      33.6536 cm-1
2.      58.6897 cm-1
3.      71.1736 cm-1
```

M06-2X/def2tzvpp-IEFPCM(water) Molecular Geometry in Cartesian Coordinates

```
C          1.991132       0.715458      -0.979528
C          1.126388       1.568758      -0.145519
C         -0.197507       1.298035       0.153114
O         -0.692281       0.171775      -0.511053
O         -0.964066       1.954401       0.896437
C         -1.933627      -0.439394      -0.138658
C         -1.996866      -1.682559      -1.019968
C         -1.921676      -0.858546       1.328711
C         -3.124532       0.462524      -0.454941
H          1.414045       0.094148      -1.660819
H          2.712725       1.284704      -1.567422
H          1.559341       2.435004       0.337885
H         -2.838323      -1.401473       1.564753
H         -1.074271      -1.519568       1.517786
H         -1.845255       0.011545       1.975453
H         -2.909733      -2.244014      -0.819322
H         -1.139432      -2.327442      -0.824387
```

```
H          -1.986113       -1.399875       -2.073278
H          -4.053261       -0.095640       -0.323013
H          -3.130087        1.334748        0.190988
H          -3.069965        0.792255       -1.494010
C           1.872837       -1.370464        0.882276
H           1.264129       -1.995299        0.230451
H           2.382829       -1.993502        1.613768
H           1.228079       -0.655911        1.393335
S           3.114949       -0.468248       -0.056135
```

2_tertbutylacrylate_HEI_2_reopt

Datum	Value
M06-2X/def2tzvpp-IEFPCM(water) Energy	-862.617443
M06-2X/def2tzvpp-IEFPCM(water) Free Energy (Quasiharmonic)	-862.434634
Number of Imaginary Frequencies	0

Frequencies (Top 3 out of 72)

```
1.      38.6737 cm-1
2.      48.1383 cm-1
3.      61.8116 cm-1
```

M06-2X/def2tzvpp-IEFPCM(water) Molecular Geometry in Cartesian Coordinates

```
C           2.299763       -1.211609        0.079350
C           0.925878       -1.160503       -0.456763
C          -0.130519       -0.748527        0.325474
O          -1.371425       -0.872757       -0.328142
O          -0.117775       -0.342884        1.511276
C          -2.358317        0.156096       -0.161729
C          -3.068670        0.040753        1.184847
C          -1.732440        1.536302       -0.336776
C          -3.355731       -0.104896       -1.284687
H           2.289248       -1.229708        1.168629
H           2.870790       -2.072834       -0.271511
H           0.744223       -1.431617       -1.487043
H          -2.510149        2.300887       -0.322067
H          -1.023277        1.740715        0.463613
H          -1.211488        1.593569       -1.294313
H          -2.382561        0.258431        1.997603
H          -3.910162        0.735360        1.220816
H          -3.455062       -0.971976        1.312141
```

```
H          -4.171409        0.617628       -1.245804
H          -2.863627       -0.026320       -2.254480
H          -3.775172       -1.107080       -1.188070
C           2.433244        1.569415        0.262050
H           2.481255        1.593579        1.349189
H           2.829661        2.499352       -0.139541
H           1.397182        1.442328       -0.051001
S           3.407806        0.207539       -0.399614
```

2_tertbutylacrylate_HEI_3

Datum	Value
M06-2X/def2tzvpp-IEFPCM(water) Energy	-862.61508
M06-2X/def2tzvpp-IEFPCM(water) Free Energy (Quasiharmonic)	-862.432617
Number of Imaginary Frequencies	0

Frequencies (Top 3 out of 72)

```
1.      39.3772 cm-1
2.      42.2028 cm-1
3.      60.2225 cm-1
```

M06-2X/def2tzvpp-IEFPCM(water) Molecular Geometry in Cartesian Coordinates

```
C          -1.784034        0.307639       -1.167524
C          -1.017228        1.373441       -0.491562
C           0.324679        1.289339       -0.173848
O           0.923975        0.113014       -0.641221
O           1.034146        2.133340        0.421500
C           2.023289       -0.477245        0.066468
C           2.137928       -1.870797       -0.541477
C           3.319747        0.292411       -0.171535
C           1.712933       -0.592127        1.555752
H          -2.632606        0.710539       -1.721933
H          -1.176490       -0.274654       -1.858124
H          -1.529303        2.279838       -0.197703
H           4.160520       -0.258093        0.254780
H           3.492031        0.403107       -1.243559
H           3.264865        1.278187        0.280333
H           1.215310       -2.430044       -0.380002
H           2.317252       -1.799221       -1.614904
H           2.964005       -2.418231       -0.086831
H           2.494262       -1.169229        2.052164
```

```
H          1.654006      0.392247      2.013835
H          0.761462     -1.107711      1.698331
C         -3.551305      0.022732      0.967544
H         -2.935375      0.787924      1.437932
H         -4.340089      0.497907      0.385266
H         -4.001387     -0.600467      1.736744
S         -2.523093     -1.020956     -0.078842
```

2_tertbutylacrylate_HEI_4_reopt

Datum	Value
M06-2X/def2tzvpp-IEFPCM(water) Energy	-862.616928
M06-2X/def2tzvpp-IEFPCM(water) Free Energy (Quasiharmonic)	-862.43426
Number of Imaginary Frequencies	0

Frequencies (Top 3 out of 72)

```
  1.      39.3121 cm-1
  2.      48.6453 cm-1
  3.      76.5104 cm-1
```

M06-2X/def2tzvpp-IEFPCM(water) Molecular Geometry in Cartesian Coordinates

```
C          2.186903     -1.089926     -0.477085
C          0.894003     -0.979983      0.225951
C         -0.090322     -0.122521     -0.217016
O         -1.181044     -0.045483      0.668076
O         -0.109795      0.580835     -1.254203
C         -2.515407      0.037785      0.145550
C         -3.400841     -0.256644      1.351038
C         -2.829114      1.435570     -0.383380
C         -2.737994     -1.021612     -0.928793
H          2.570283     -2.110443     -0.525133
H          2.106145     -0.708038     -1.494270
H          0.744902     -1.514972      1.153418
H         -3.891239      1.509394     -0.624781
H         -2.600038      2.179030      0.382100
H         -2.238864      1.651102     -1.268638
H         -4.453585     -0.209731      1.071040
H         -3.186825     -1.250693      1.744600
H         -3.218425      0.475598      2.138565
H         -2.108445     -0.827922     -1.795175
H         -2.502498     -2.011161     -0.533419
```

```
H           -3.782080       -1.017042       -1.244156
C            2.937593        1.489140        0.277705
H            1.924761        1.465100        0.678065
H            3.563677        2.121328        0.903265
H            2.919009        1.887775       -0.735078
S            3.613187       -0.179563        0.304674
```

2_tertbutylacrylate_HEI_5

Datum	Value
M06-2X/def2tzvpp-IEFPCM(water) Energy	-862.614766
M06-2X/def2tzvpp-IEFPCM(water) Free Energy (Quasiharmonic)	-862.432771
Number of Imaginary Frequencies	0

Frequencies (Top 3 out of 72)

```
1.      18.0610 cm-1
2.      41.5752 cm-1
3.      59.1559 cm-1
```

M06-2X/def2tzvpp-IEFPCM(water) Molecular Geometry in Cartesian Coordinates

```
C           -1.689989       -0.135950        1.042030
C           -0.992826        1.087206        0.598764
C            0.254497        1.104980        0.005752
O            0.785888       -0.174270       -0.201582
O            0.917377        2.099815       -0.370848
C            2.197123       -0.399854       -0.064440
C            2.319186       -1.918512       -0.011067
C            2.972423        0.130509       -1.267672
C            2.712763        0.205836        1.236784
H           -0.994761       -0.923502        1.328106
H           -2.355193        0.055682        1.885402
H           -1.505065        2.037257        0.675662
H            4.014079       -0.190187       -1.205838
H            2.545299       -0.271235       -2.188074
H            2.927088        1.214605       -1.303153
H            3.364940       -2.213661        0.079165
H            1.910865       -2.359797       -0.921083
H            1.768742       -2.313336        0.843570
H            3.756043       -0.073943        1.388359
H            2.638622        1.291016        1.209886
H            2.130009       -0.170146        2.079812
```

```
C          -3.928551       0.350700       -0.539341
H          -3.384661       1.232337       -0.875080
H          -4.613875       0.032836       -1.321663
H          -4.498127       0.595049        0.356758
S          -2.774868      -0.993552       -0.216078
```

2_tertbutylacrylate_HEI_6

Datum	Value
M06-2X/def2tzvpp-IEFPCM(water) Energy	-862.616393
M06-2X/def2tzvpp-IEFPCM(water) Free Energy (Quasiharmonic)	-862.43404
Number of Imaginary Frequencies	0

Frequencies (Top 3 out of 72)

```
1.      25.9747 cm-1
2.      44.7451 cm-1
3.      56.3557 cm-1
```

M06-2X/def2tzvpp-IEFPCM(water) Molecular Geometry in Cartesian Coordinates

```
C           2.215100      -0.909691       -0.740487
C           0.895754      -0.331977       -1.062235
C          -0.233243      -0.754820       -0.397143
O          -1.416350      -0.165386       -0.878355
O          -0.318212      -1.604391        0.519744
C          -2.398568       0.287989        0.066654
C          -1.736188       1.082617        1.187714
C          -3.304466       1.201856       -0.750019
C          -3.217426      -0.872954        0.626022
H           2.125740      -1.937503       -0.388054
H           2.898774      -0.895897       -1.590239
H           0.815604       0.458115       -1.794012
H          -4.108724       1.595910       -0.128085
H          -2.733496       2.037593       -1.155460
H          -3.746657       0.648158       -1.579162
H          -2.498670       1.507312        1.841700
H          -1.084148       0.442042        1.779489
H          -1.145441       1.900136        0.770765
H          -2.595716      -1.516215        1.241464
H          -4.047267      -0.487712        1.221676
H          -3.629785      -1.462568       -0.194602
C           3.303672       1.558683       -0.070953
```

```
H          2.314189       1.951404      -0.301231
H          3.786166       2.205349       0.658255
H          3.905280       1.532674      -0.978942
S          3.154552      -0.086924       0.644250
```

2_tertbutylacrylate_HEI_7

Datum	Value
M06-2X/def2tzvpp-IEFPCM(water) Energy	-862.615288
M06-2X/def2tzvpp-IEFPCM(water) Free Energy (Quasiharmonic)	-862.433379
Number of Imaginary Frequencies	0

Frequencies (Top 3 out of 72)

```
1.      37.8453 cm-1
2.      43.0621 cm-1
3.      56.8470 cm-1
```

M06-2X/def2tzvpp-IEFPCM(water) Molecular Geometry in Cartesian Coordinates

```
C         -1.795706       0.782493      -0.734376
C         -0.745707       1.708152      -0.249937
C          0.590784       1.383234      -0.132623
O          0.894196       0.105183      -0.622021
O          1.524327       2.097867       0.302321
C          1.935992      -0.675934      -0.019586
C          1.697730      -2.079080      -0.566044
C          3.318543      -0.189561      -0.446754
C          1.798531      -0.687531       1.499557
H         -2.614264       1.310426      -1.226431
H         -1.403471       0.037030      -1.424250
H         -1.033634       2.703418       0.062326
H          4.080912      -0.890527      -0.101725
H          3.370760      -0.136052      -1.535525
H          3.519189       0.795433      -0.036238
H          2.449277      -2.770313      -0.183735
H          0.710069      -2.434970      -0.269820
H          1.752798      -2.073062      -1.655318
H          2.519955      -1.383430       1.929853
H          1.975867       0.304180       1.909345
H          0.795914      -1.014844       1.780860
C         -3.798190      -1.132009      -0.284456
H         -4.345866      -1.759157       0.415523
```

```
H          -4.500447       -0.473555       -0.793890
H          -3.303146       -1.767968       -1.017065
S          -2.583372       -0.170599        0.644312
```

2_tertbutylacrylate_HEI_8_reopt

Datum	Value
M06-2X/def2tzvpp-IEFPCM(water) Energy	-862.616417
M06-2X/def2tzvpp-IEFPCM(water) Free Energy (Quasiharmonic)	-862.434929
Number of Imaginary Frequencies	0

Frequencies (Top 3 out of 72)

```
1.      18.7681 cm-1
2.      40.8049 cm-1
3.      53.8019 cm-1
```

M06-2X/def2tzvpp-IEFPCM(water) Molecular Geometry in Cartesian Coordinates

```
C          -2.131571       -0.564184        0.795230
C          -0.732774       -0.196248        1.109210
C           0.305327       -0.619837        0.309589
O           1.568577       -0.277202        0.823712
O           0.250560       -1.288189       -0.748993
C           2.585968        0.194929       -0.073070
C           3.629407        0.816503        0.848060
C           3.220671       -0.949701       -0.859572
C           2.022860        1.259137       -1.009128
H          -2.176957       -1.489544        0.219904
H          -2.749945       -0.677804        1.687206
H          -0.521404        0.423023        1.968548
H           4.095189       -0.584932       -1.401778
H           3.545835       -1.732914       -0.172626
H           2.509141       -1.373115       -1.562099
H           4.466734        1.205194        0.267862
H           3.190444        1.633944        1.420697
H           4.008198        0.068094        1.545204
H           1.565697        2.063688       -0.430797
H           2.824936        1.682752       -1.614726
H           1.273242        0.829879       -1.671613
C          -4.604431       -0.029082       -0.409724
H          -5.070409       -0.164858        0.565303
H          -5.223362        0.636032       -1.007846
```

```
H          -4.531711       -0.992535       -0.912329
S          -2.970717        0.720446       -0.235139
```

2_tertbutylacrylate_TS_1_reopt

Datum	Value
M06-2X/def2tzvpp-IEFPCM(water) Energy	-862.608577
M06-2X/def2tzvpp-IEFPCM(water) Free Energy (Quasiharmonic)	-862.426568
Number of Imaginary Frequencies	1

Frequencies (Top 3 out of 72)

```
1.    -207.5432 cm-1
2.      43.8929 cm-1
3.      46.2867 cm-1
```

M06-2X/def2tzvpp-IEFPCM(water) Molecular Geometry in Cartesian Coordinates

```
C           1.797103        0.973513       -1.182995
C           1.041807        1.654744       -0.245373
C          -0.262500        1.248085        0.178376
O          -0.683726        0.104966       -0.428473
O          -0.961659        1.843522        0.995635
C          -1.964700       -0.495679       -0.133799
C          -1.973702       -1.736155       -1.017600
C          -2.047541       -0.905328        1.332074
C          -3.106465        0.432834       -0.530477
H           1.298664        0.292407       -1.854731
H           2.669457        1.460756       -1.593369
H           1.450104        2.504190        0.285134
H          -2.945913       -1.503844        1.488081
H          -1.181142       -1.513196        1.596750
H          -2.083563       -0.035780        1.981762
H          -2.901443       -2.290249       -0.877392
H          -1.135430       -2.385573       -0.763499
H          -1.890313       -1.453819       -2.067287
H          -4.051629       -0.106312       -0.455021
H          -3.144120        1.305869        0.114474
H          -2.980306        0.757232       -1.564351
C           2.248887       -0.838139        1.327786
H           1.636609       -1.734740        1.420928
H           2.954288       -0.809413        2.156986
```

```
H           1.590637        0.036899        1.411043
S           3.092491       -0.757224       -0.261548
```

2_tertbutylacrylate_TS_2

Datum	Value
M06-2X/def2tzvpp-IEFPCM(water) Energy	-862.609398
M06-2X/def2tzvpp-IEFPCM(water) Free Energy (Quasiharmonic)	-862.427887
Number of Imaginary Frequencies	1

Frequencies (Top 3 out of 72)

```
1.    -203.1837 cm-1
2.      24.5195 cm-1
3.      41.2576 cm-1
```

M06-2X/def2tzvpp-IEFPCM(water) Molecular Geometry in Cartesian Coordinates

```
C          -2.156400       -1.499056        0.258673
C          -0.950859       -1.106068        0.804678
C           0.124667       -0.686297       -0.040656
O           1.221263       -0.317453        0.683472
O           0.113996       -0.649819       -1.267032
C           2.414412        0.198195        0.051032
C           3.055943       -0.856372       -0.843360
C           2.109028        1.483525       -0.710116
C           3.330306        0.501782        1.229525
H          -2.174410       -1.782152       -0.783027
H          -2.881292       -1.996672        0.885129
H          -0.818992       -1.007371        1.872608
H           3.044499        1.943209       -1.031191
H           1.492567        1.287965       -1.582785
H           1.589482        2.186479       -0.056954
H           2.436794       -1.064247       -1.710961
H           4.030887       -0.499985       -1.178211
H           3.204713       -1.779383       -0.281189
H           4.278545        0.902487        0.872232
H           2.867580        1.235223        1.890008
H           3.527578       -0.406203        1.799591
C          -2.419777        1.628276        0.160276
H          -2.150714        2.152382       -0.756553
H          -2.791902        2.354805        0.881266
```

```
H          -1.508833        1.174961        0.574836
S          -3.617031        0.315799       -0.136664
```

2_tertbutylacrylate_TS_3

Datum	Value
M06-2X/def2tzvpp-IEFPCM(water) Energy	-862.608577
M06-2X/def2tzvpp-IEFPCM(water) Free Energy (Quasiharmonic)	-862.426568
Number of Imaginary Frequencies	1

Frequencies (Top 3 out of 72)

```
1.    -207.5367 cm-1
2.      43.8975 cm-1
3.      46.2941 cm-1
```

M06-2X/def2tzvpp-IEFPCM(water) Molecular Geometry in Cartesian Coordinates

```
C          -1.797097        0.973549       -1.182953
C          -1.041795        1.654741       -0.245318
C           0.262524        1.248081        0.178403
O           0.683706        0.104924       -0.428396
O           0.961717        1.843553        0.995613
C           1.964714       -0.495694       -0.133804
C           1.973693       -1.736166       -1.017609
C           3.106435        0.432843       -0.530554
C           2.047657       -0.905334        1.332062
H          -2.669443        1.460812       -1.593318
H          -1.298690        0.292411       -1.854677
H          -1.450064        2.504212        0.285171
H           4.051592       -0.106349       -0.455349
H           2.980094        0.757386       -1.564359
H           3.144243        1.305790        0.114507
H           1.135392       -2.385554       -0.763529
H           1.890339       -1.453825       -2.067298
H           2.901411       -2.290292       -0.877382
H           2.946177       -1.503632        1.488059
H           2.083484       -0.035776        1.981748
H           1.181412       -1.513414        1.596749
C          -2.248871       -0.838214        1.327728
H          -1.590736        0.036901        1.411079
H          -2.954229       -0.809706        2.156972
```

```
H          -1.636466       -1.734746        1.420709
S          -3.092591       -0.757179       -0.261535
```

2_tertbutylacrylate_TS_4

Datum	Value
M06-2X/def2tzvpp-IEFPCM(water) Energy	-862.609398
M06-2X/def2tzvpp-IEFPCM(water) Free Energy (Quasiharmonic)	-862.427885
Number of Imaginary Frequencies	1

Frequencies (Top 3 out of 72)

```
1.    -203.1495 cm-1
2.      24.5833 cm-1
3.      41.2776 cm-1
```

M06-2X/def2tzvpp-IEFPCM(water) Molecular Geometry in Cartesian Coordinates

```
C           2.156396       -1.499079        0.258804
C           0.950848       -1.106030        0.804735
C          -0.124667       -0.686279       -0.040636
O          -1.221275       -0.317426        0.683462
O          -0.113958       -0.649807       -1.267014
C          -2.414433        0.198196        0.051013
C          -3.330399        0.501642        1.229485
C          -2.109063        1.483590       -0.710029
C          -3.055874       -0.856325       -0.843501
H           2.881289       -1.996585        0.885344
H           2.174483       -1.782248       -0.782873
H           0.818947       -1.007295        1.872658
H          -3.044513        1.943012       -1.031539
H          -1.589990        2.186706       -0.056666
H          -1.492168        1.288148       -1.582419
H          -4.278566        0.902515        0.872187
H          -3.527817       -0.406431        1.799360
H          -2.867656        1.234909        1.890148
H          -2.436748       -1.063998       -1.711163
H          -3.204496       -1.779438       -0.281458
H          -4.030881       -0.500010       -1.178243
C           2.419821        1.628235        0.160470
H           1.508869        1.174847        0.574930
H           2.791975        2.354576        0.881637
```

```
  H          2.150757        2.152583       -0.756219
  S          3.617038        0.315805       -0.136832
```

2_tertbutylacrylate_TS_5_reopt

Datum	Value
M06-2X/def2tzvpp-IEFPCM(water) Energy	-862.608577
M06-2X/def2tzvpp-IEFPCM(water) Free Energy (Quasiharmonic)	-862.426571
Number of Imaginary Frequencies	1

Frequencies (Top 3 out of 72)

```
    1.    -207.5527 cm-1
    2.      43.8507 cm-1
    3.      46.1623 cm-1
```

M06-2X/def2tzvpp-IEFPCM(water) Molecular Geometry in Cartesian Coordinates

```
  C          1.797111        0.973626       -1.182895
  C          1.041802        1.654781       -0.245236
  C         -0.262507        1.248081        0.178477
  O         -0.683702        0.104983       -0.428425
  O         -0.961688        1.843479        0.995744
  C         -1.964696       -0.495669       -0.133847
  C         -1.973697       -1.736055       -1.017774
  C         -2.047577       -0.905468        1.331980
  C         -3.106437        0.432902       -0.530460
  H          1.298698        0.292518       -1.854647
  H          2.669454        1.460906       -1.593248
  H          1.450082        2.504208        0.285315
  H         -2.946014       -1.503904        1.487924
  H         -1.181246       -1.513462        1.596588
  H         -2.083509       -0.035993        1.981770
  H         -2.901435       -2.290166       -0.877617
  H         -1.135423       -2.385498       -0.763741
  H         -1.890315       -1.453613       -2.067433
  H         -4.051605       -0.106253       -0.455128
  H         -3.144122        1.305848        0.114608
  H         -2.980220        0.757440       -1.564282
  C          2.248671       -0.838470        1.327614
  H          1.590468        0.036587        1.411023
  H          1.636313       -1.735059        1.420351
```

```
H          2.953907       -0.810057        2.156965
S          3.092629       -0.757098       -0.261510
```

2_tertbutylacrylate_TS_6_reopt

Datum	Value
M06-2X/def2tzvpp-IEFPCM(water) Energy	-862.609398
M06-2X/def2tzvpp-IEFPCM(water) Free Energy (Quasiharmonic)	-862.427882
Number of Imaginary Frequencies	1

Frequencies (Top 3 out of 72)

```
1.    -203.1607 cm-1
2.      24.6706 cm-1
3.      41.2816 cm-1
```

M06-2X/def2tzvpp-IEFPCM(water) Molecular Geometry in Cartesian Coordinates

```
C         -2.156332       -1.499065        0.258658
C         -0.950848       -1.105946        0.804705
C          0.124630       -0.686044       -0.040639
O          1.221205       -0.317122        0.683470
O          0.113901       -0.649512       -1.267008
C          2.414507        0.198157        0.051014
C          2.109473        1.483366       -0.710477
C          3.330362        0.501822        1.229517
C          3.055880       -0.856756       -0.843083
H         -2.174282       -1.782138       -0.783049
H         -2.881202       -1.996750        0.885084
H         -0.818991       -1.007273        1.872638
H          4.278657        0.902392        0.872223
H          2.867662        1.235398        1.889867
H          3.527518       -0.406099        1.799725
H          3.045077        1.942733       -1.031618
H          1.493017        1.287724       -1.583131
H          1.590065        2.186621       -0.057529
H          2.436741       -1.064704       -1.710674
H          4.030916       -0.500647       -1.177960
H          3.204424       -1.779666       -0.280687
C         -2.420015        1.628214        0.160532
H         -1.509179        1.174930        0.575359
H         -2.150706        2.152347       -0.756208
```

```
H          -2.792388        2.354711        0.881428
S          -3.617132        0.315688       -0.136783
```

2_tertbutylacrylate_TS_7_reopt

Datum	Value
M06-2X/def2tzvpp-IEFPCM(water) Energy	-862.603501
M06-2X/def2tzvpp-IEFPCM(water) Free Energy (Quasiharmonic)	-862.423177
Number of Imaginary Frequencies	1

Frequencies (Top 3 out of 72)

```
1.    -233.9947 cm-1
2.      25.3806 cm-1
3.      32.5538 cm-1
```

M06-2X/def2tzvpp-IEFPCM(water) Molecular Geometry in Cartesian Coordinates

```
C          -1.688319        0.928356       -0.797237
C          -0.687404        1.754437       -0.325180
C           0.639210        1.311502       -0.025874
O           0.831943       -0.009633       -0.288836
O           1.538141        2.021311        0.418835
C           2.090654       -0.670712       -0.027450
C           1.819683       -2.108155       -0.452161
C           3.205880       -0.079378       -0.881623
C           2.425819       -0.625556        1.458838
H          -2.595403        1.377327       -1.177669
H          -1.419881       -0.011906       -1.257026
H          -0.898161        2.780544       -0.056875
H           4.097703       -0.700425       -0.788728
H           2.905374       -0.068708       -1.930252
H           3.444608        0.933079       -0.569743
H           2.706971       -2.719860       -0.291218
H           0.996683       -2.524295        0.129380
H           1.555129       -2.147317       -1.509019
H           3.285283       -1.268093        1.653699
H           2.659642        0.385740        1.778471
H           1.581421       -0.997936        2.040830
C          -4.014818       -1.030854       -0.488123
H          -4.974803       -0.522354       -0.570065
H          -3.518401       -0.970585       -1.461029
```

```
H          -4.201236       -2.082553       -0.275967
S          -2.958114       -0.277120        0.779880
```

2_tertbutylacrylate_TS_8_reopt

Datum	Value
M06-2X/def2tzvpp-IEFPCM(water) Energy	-862.604234
M06-2X/def2tzvpp-IEFPCM(water) Free Energy (Quasiharmonic)	-862.423904
Number of Imaginary Frequencies	1

Frequencies (Top 3 out of 72)

```
1.    -235.9450 cm-1
2.      34.5894 cm-1
3.      41.4428 cm-1
```

M06-2X/def2tzvpp-IEFPCM(water) Molecular Geometry in Cartesian Coordinates

```
C           1.974457       -1.076030        0.061863
C           0.705821       -1.089571       -0.475554
C          -0.356442       -0.371991        0.160564
O          -1.528686       -0.495537       -0.524948
O          -0.273088        0.282853        1.194250
C          -2.750603        0.131559       -0.072478
C          -3.764154       -0.277278       -1.133390
C          -3.172991       -0.418682        1.284910
C          -2.607800        1.649203       -0.052484
H           2.090811       -0.788020        1.097813
H           2.716072       -1.768557       -0.310343
H           0.502411       -1.552023       -1.430419
H          -4.172571       -0.054473        1.525222
H          -3.206014       -1.508504        1.250436
H          -2.485947       -0.108166        2.066569
H          -4.741746        0.142317       -0.897604
H          -3.454609        0.087042       -2.113049
H          -3.850922       -1.363087       -1.175745
H          -2.244078        2.000935       -1.018934
H          -3.584513        2.099426        0.128911
H          -1.920107        1.970233        0.724316
C           4.794468        0.014975        0.420052
H           4.410124       -0.601244        1.237670
H           5.425122       -0.615109       -0.206690
```

```
H          5.417078        0.794393        0.856851
S          3.411034        0.724575       -0.515644
```

3_methylcrotonate_1

Datum	Value
M06-2X/def2tzvpp-IEFPCM(water) Energy	-345.784109
M06-2X/def2tzvpp-IEFPCM(water) Free Energy (Quasiharmonic)	-345.688853
Number of Imaginary Frequencies	0

Frequencies (Top 3 out of 39)

```
1.      73.6615 cm-1
2.     146.0981 cm-1
3.     159.5768 cm-1
```

M06-2X/def2tzvpp-IEFPCM(water) Molecular Geometry in Cartesian Coordinates

```
C          1.871906        0.283441        0.000002
C          0.770252       -0.459914       -0.000002
C         -0.566043        0.168318       -0.000000
O         -1.534744       -0.753625       -0.000004
O         -0.790902        1.355779        0.000004
C         -2.872637       -0.253728       -0.000002
H         -3.516873       -1.125991       -0.000003
H         -3.049539        0.349974        0.887649
H         -3.049540        0.349977       -0.887650
H          1.754526        1.362842        0.000007
C          3.260286       -0.252874        0.000001
H          0.805396       -1.541425       -0.000006
H          3.803356        0.108356        0.875070
H          3.271897       -1.340784       -0.000004
H          3.803358        0.108364       -0.875063
```

3_methylcrotonate_2

Datum	Value
M06-2X/def2tzvpp-IEFPCM(water) Energy	-345.783427

Datum	Value
M06-2X/def2tzvpp-IEFPCM(water) Free Energy (Quasiharmonic)	-345.688127
Number of Imaginary Frequencies	0

Frequencies (Top 3 out of 39)

```
1.      85.3381 cm-1
2.     139.7822 cm-1
3.     161.0840 cm-1
```

M06-2X/def2tzvpp-IEFPCM(water) Molecular Geometry in Cartesian Coordinates

```
C          1.664156      -0.400580      -0.000004
C          0.836520       0.640264       0.000004
C         -0.633754       0.524921       0.000005
O         -1.078546      -0.734111      -0.000004
O         -1.375323       1.480870       0.000013
C         -2.497419      -0.892965      -0.000005
H         -2.676728      -1.962471      -0.000011
H         -2.928733      -0.435156       0.887702
H         -2.928733      -0.435147      -0.887708
H          1.241780      -1.399512      -0.000011
C          3.149552      -0.295272      -0.000005
H          1.203816       1.658578       0.000011
H          3.562600      -0.799937      -0.875125
H          3.480016       0.741313       0.000003
H          3.562601      -0.799950       0.875107
```

3_methylcrotonate_3

Datum	Value
M06-2X/def2tzvpp-IEFPCM(water) Energy	-345.775611
M06-2X/def2tzvpp-IEFPCM(water) Free Energy (Quasiharmonic)	-345.680396
Number of Imaginary Frequencies	0

Frequencies (Top 3 out of 39)

```
1.      51.1125 cm-1
2.     135.6901 cm-1
```

```
3.     169.7836 cm-1
```

M06-2X/def2tzvpp-IEFPCM(water) Molecular Geometry in Cartesian Coordinates

```
C          1.724753        0.213489        0.000005
C          0.490686       -0.285024       -0.000009
C         -0.672681        0.631716       -0.000000
O         -1.905048        0.103742        0.000001
O         -0.577324        1.836078        0.000005
C         -2.116810       -1.308103        0.000001
H         -3.193526       -1.440717        0.000011
H         -1.695993       -1.765901       -0.893209
H         -1.695977       -1.765903        0.893203
H          1.836642        1.292957        0.000022
C          2.968701       -0.601743       -0.000003
H          0.335163       -1.353541       -0.000026
H          3.574942       -0.359561        0.874750
H          2.754886       -1.668371       -0.000020
H          3.574949       -0.359534       -0.874743
```

3_methylcrotonate_HEI_1

Datum	Value
M06-2X/def2tzvpp-IEFPCM(water) Energy	-783.995001
M06-2X/def2tzvpp-IEFPCM(water) Free Energy (Quasiharmonic)	-783.866194
Number of Imaginary Frequencies	0

Frequencies (Top 3 out of 54)

```
1.      60.2649 cm-1
2.      70.6865 cm-1
3.      81.3294 cm-1
```

M06-2X/def2tzvpp-IEFPCM(water) Molecular Geometry in Cartesian Coordinates

```
C          1.139143        0.749575        0.304819
C         -0.149978        0.776021       -0.405889
C         -1.291098        0.221226        0.137687
O         -2.409533        0.333180       -0.700323
```

```
O          -1.450314       -0.338024        1.245316
C          -3.617320       -0.179911       -0.182475
H          -3.920365        0.339603        0.728046
H          -4.370891       -0.026019       -0.952339
H          -3.541116       -1.244672        0.041956
H           0.970193        0.546792        1.362418
C           1.960768        2.023511        0.140976
H          -0.191609        1.198003       -1.402067
H           1.423298        2.866289        0.576273
H           2.936490        1.946856        0.624018
H           2.119989        2.236557       -0.917803
S           2.305295       -0.628154       -0.244250
C           1.219122       -2.050317       -0.062503
H           1.693239       -2.902965       -0.543837
H           1.041031       -2.279616        0.986376
H           0.269967       -1.832239       -0.550668
```

3_methylcrotonate_HEI_2

Datum	Value
M06-2X/def2tzvpp-IEFPCM(water) Energy	-783.991845
M06-2X/def2tzvpp-IEFPCM(water) Free Energy (Quasiharmonic)	-783.863401
Number of Imaginary Frequencies	0

Frequencies (Top 3 out of 54)

```
1.      37.8713 cm-1
2.      46.8032 cm-1
3.      83.2227 cm-1
```

M06-2X/def2tzvpp-IEFPCM(water) Molecular Geometry in Cartesian Coordinates

```
C          -0.799975        0.535410       -0.647598
C           0.177098        0.918394        0.392286
C           1.479812        0.463530        0.455629
O           1.817066       -0.416030       -0.578188
O           2.367251        0.739982        1.294586
C           3.155402       -0.863273       -0.597414
H           3.413004       -1.406255        0.312521
H           3.242660       -1.529714       -1.453193
H           3.856307       -0.035133       -0.711274
H          -0.295126        0.274118       -1.577501
C          -1.828321        1.630025       -0.912548
```

```
H         -0.148435      1.575515      1.188659
H         -2.559689      1.328292     -1.661682
H         -2.362754      1.882371      0.005361
H         -1.323862      2.534734     -1.256214
S         -1.720162     -1.082081     -0.316690
C         -2.491552     -0.689371      1.262631
H         -1.743621     -0.258406      1.928377
H         -3.322940      0.003376      1.147069
H         -2.862285     -1.615507      1.695822
```

3_methylcrotonate_HEI_3

Datum	Value
M06-2X/def2tzvpp-IEFPCM(water) Energy	-783.988922
M06-2X/def2tzvpp-IEFPCM(water) Free Energy (Quasiharmonic)	-783.860235
Number of Imaginary Frequencies	0

Frequencies (Top 3 out of 54)

```
1.      31.4725 cm-1
2.      64.1989 cm-1
3.      85.9684 cm-1
```

M06-2X/def2tzvpp-IEFPCM(water) Molecular Geometry in Cartesian Coordinates

```
C         -0.955382      0.555704     -0.815677
C          0.125486     -0.413010     -1.116850
C          1.352240     -0.537145     -0.488184
O          1.581012      0.393171      0.525679
O          2.258740     -1.369223     -0.722039
C          2.852314      0.342333      1.136833
H          3.653086      0.496595      0.412600
H          2.869820      1.144029      1.872395
H          3.022978     -0.611708      1.636669
H         -1.701527      0.474365     -1.608246
C         -0.576110      2.029971     -0.685120
H         -0.094479     -1.187218     -1.839655
H         -1.466368      2.652075     -0.577101
H          0.071893      2.192777      0.172579
H         -0.038716      2.346271     -1.580126
S         -1.963114      0.187479      0.735173
C         -2.456951     -1.503402      0.369784
H         -3.108026     -1.539416     -0.503201
```

```
H         -1.572152       -2.111044        0.186619
H         -2.994278       -1.894681        1.230862
```

3_methylcrotonate_HEI_4

Datum	Value
M06-2X/def2tzvpp-IEFPCM(water) Energy	-783.994467
M06-2X/def2tzvpp-IEFPCM(water) Free Energy (Quasiharmonic)	-783.865886
Number of Imaginary Frequencies	0

Frequencies (Top 3 out of 54)

```
1.      42.9921 cm-1
2.      55.8435 cm-1
3.      79.4335 cm-1
```

M06-2X/def2tzvpp-IEFPCM(water) Molecular Geometry in Cartesian Coordinates

```
C          0.903260        0.600634        0.287067
C         -0.447233        0.773049       -0.295485
C         -1.521547        0.026486        0.141213
O         -2.712956        0.325395       -0.532535
O         -1.562642       -0.844276        1.038733
C         -3.859945       -0.365224       -0.087921
H         -4.685035       -0.019385       -0.707481
H         -3.751558       -1.444706       -0.199757
H         -4.080716       -0.151029        0.958991
H          0.827074        0.207996        1.303004
C          1.722479        1.883657        0.284350
H         -0.582129        1.477660       -1.105141
H          2.731832        1.732748        0.667504
H          1.796732        2.279030       -0.730249
H          1.230897        2.633599        0.906987
S          1.808987       -0.730742       -0.649187
C          3.339402       -0.913013        0.298766
H          3.110900       -0.980105        1.362246
H          3.817791       -1.839001       -0.013897
H          4.026702       -0.087420        0.127252
```

3_methylcrotonate_HEI_5_reopt3

Datum	Value
M06-2X/def2tzvpp-IEFPCM(water) Energy	-783.988159
M06-2X/def2tzvpp-IEFPCM(water) Free Energy (Quasiharmonic)	-783.85847
Number of Imaginary Frequencies	0

Frequencies (Top 3 out of 54)

```
1.      46.4774 cm-1
2.      72.9766 cm-1
3.      88.4450 cm-1
```

M06-2X/def2tzvpp-IEFPCM(water) Molecular Geometry in Cartesian Coordinates

```
C          1.109704      -1.053900       0.088031
C         -0.109914      -1.113017      -0.754981
C         -1.328022      -0.488703      -0.561172
O         -1.451177       0.205721       0.643667
O         -2.321929      -0.491439      -1.326613
C         -2.645571       0.935375       0.817144
H         -2.579151       1.400944       1.798689
H         -2.756522       1.711065       0.057034
H         -3.524479       0.291624       0.778789
H          1.738233      -1.908350      -0.169992
C          0.940253      -1.049480       1.606018
H         -0.005854      -1.603788      -1.714417
H          1.902800      -1.209948       2.091861
H          0.523084      -0.109801       1.958026
H          0.257869      -1.847022       1.906487
S          2.296348       0.341664      -0.376200
C          1.193437       1.759959      -0.269692
H          1.738139       2.628834      -0.633083
H          0.319764       1.586196      -0.896587
H          0.870075       1.937959       0.753883
```

3_methylcrotonate_HEI_6

Datum	Value
M06-2X/def2tzvpp-IEFPCM(water) Energy	-783.994667
M06-2X/def2tzvpp-IEFPCM(water) Free Energy (Quasiharmonic)	-783.864234
Number of Imaginary Frequencies	0

Frequencies (Top 3 out of 54)

```
1.      54.6686 cm-1
2.      78.5247 cm-1
3.     101.5193 cm-1
```

M06-2X/def2tzvpp-IEFPCM(water) Molecular Geometry in Cartesian Coordinates

```
C          0.962432        0.731130        0.419627
C         -0.417351        0.595028       -0.092916
C         -1.345409       -0.184093        0.566497
O         -2.640897       -0.291339        0.050492
O         -1.194100       -0.837637        1.626835
C         -2.965388        0.375219       -1.146154
H         -2.848941        1.457422       -1.048415
H         -2.346472        0.031085       -1.978478
H         -4.007593        0.147513       -1.360367
H          1.004803        0.383056        1.451177
C          1.511245        2.150908        0.322983
H         -0.651902        1.099734       -1.018030
H          2.549293        2.212024        0.654263
H          1.460182        2.507068       -0.707788
H          0.909839        2.819270        0.939590
S          2.217831       -0.322096       -0.490965
C          1.456794       -1.944431       -0.330822
H          1.959467       -2.622000       -1.017558
H          1.548826       -2.326543        0.683972
H          0.403234       -1.865859       -0.596830
```

3_methylcrotonate_HEI_7

Datum	Value
M06-2X/def2tzvpp-IEFPCM(water) Energy	-783.993038
M06-2X/def2tzvpp-IEFPCM(water) Free Energy (Quasiharmonic)	-783.864016
Number of Imaginary Frequencies	0

Frequencies (Top 3 out of 54)

```
1.      48.9703 cm-1
2.      70.5437 cm-1
3.      84.6283 cm-1
```

M06-2X/def2tzvpp-IEFPCM(water) Molecular Geometry in Cartesian Coordinates

```
C          0.785376       0.576758       0.384364
C         -0.434748       1.115828      -0.263262
C         -1.673870       0.507050      -0.244086
O         -1.702570      -0.691479       0.476546
O         -2.743628       0.891840      -0.769462
C         -2.960837      -1.323424       0.569412
H         -3.356567      -1.580025      -0.413689
H         -2.804517      -2.232909       1.146033
H         -3.692127      -0.693818       1.078098
H          0.525990      -0.069569       1.223675
C          1.734863       1.672767       0.849107
H         -0.346343       2.030782      -0.835530
H          2.656681       1.272670       1.271518
H          1.995296       2.322705       0.011800
H          1.246359       2.280428       1.613153
S          1.665422      -0.553615      -0.806535
C          3.019013      -1.212516       0.197878
H          3.811786      -0.482274       0.344835
H          2.638212      -1.534406       1.167026
H          3.429292      -2.077411      -0.319504
```

3_methylcrotonate_HEI_8_reopt

Datum	Value
M06-2X/def2tzvpp-IEFPCM(water) Energy	-783.990291
M06-2X/def2tzvpp-IEFPCM(water) Free Energy (Quasiharmonic)	-783.860872
Number of Imaginary Frequencies	0

Frequencies (Top 3 out of 54)

```
1.      64.1062 cm-1
2.      73.0587 cm-1
3.      91.6012 cm-1
```

M06-2X/def2tzvpp-IEFPCM(water) Molecular Geometry in Cartesian Coordinates

```
C        -1.156367     0.894023    -0.531480
C         0.208162     0.480034    -0.934116
C         1.248815     0.203382    -0.070354
O         2.414538    -0.191219    -0.755623
O         1.311563     0.258671     1.177261
C         3.539477    -0.454065     0.051828
H         3.352514    -1.265149     0.757268
H         4.341905    -0.743332    -0.624291
H         3.846869     0.426216     0.618894
H        -1.610131     1.464069    -1.344912
C        -1.267972     1.718303     0.747362
H         0.380251     0.296839    -1.986214
H        -2.287407     2.080394     0.880040
H        -0.979768     1.132207     1.616130
H        -0.593231     2.575087     0.694303
S        -2.389212    -0.533121    -0.438286
C        -1.552630    -1.595192     0.750838
H        -2.018178    -2.577552     0.711738
H        -0.503235    -1.673790     0.468718
H        -1.627923    -1.203568     1.763329
```

3_methylcrotonate_TS_1

Datum	Value
M06-2X/def2tzvpp-IEFPCM(water) Energy	-783.987906
M06-2X/def2tzvpp-IEFPCM(water) Free Energy (Quasiharmonic)	-783.859366
Number of Imaginary Frequencies	1

Frequencies (Top 3 out of 54)

```
1.    -198.8273 cm-1
2.      57.2731 cm-1
3.      63.9246 cm-1
```

M06-2X/def2tzvpp-IEFPCM(water) Molecular Geometry in Cartesian Coordinates

```
C         1.011671     1.077530     0.268122
C        -0.169313     0.924959    -0.435399
C        -1.310666     0.332202     0.177189
O        -2.380355     0.252441    -0.672639
O        -1.410681    -0.080023     1.329199
C        -3.556322    -0.330757    -0.134696
```

```
H          -3.922737        0.240224        0.717611
H          -4.293320       -0.317224       -0.932543
H          -3.373124       -1.356572        0.183464
H           0.934626        0.969472        1.341637
C           2.063464        2.038895       -0.210519
H          -0.231807        1.179953       -1.485103
H           1.750109        3.062087        0.015484
H           3.022221        1.863104        0.273905
H           2.201150        1.958210       -1.288501
S           2.330142       -0.897922        0.155653
C           1.063200       -2.022176       -0.459568
H           1.424062       -2.601281       -1.308524
H           0.713515       -2.711925        0.308151
H           0.209114       -1.422551       -0.799274
```

3_methylcrotonate_TS_2

Datum	Value
M06-2X/def2tzvpp-IEFPCM(water) Energy	-783.986773
M06-2X/def2tzvpp-IEFPCM(water) Free Energy (Quasiharmonic)	-783.858417
Number of Imaginary Frequencies	1

Frequencies (Top 3 out of 54)

```
1.    -211.3018 cm-1
2.      31.6181 cm-1
3.      56.7624 cm-1
```

M06-2X/def2tzvpp-IEFPCM(water) Molecular Geometry in Cartesian Coordinates

```
C          -0.906469        1.017228       -0.384916
C           0.091814        1.103555        0.571932
C           1.394880        0.549137        0.418127
O           1.588043       -0.087157       -0.774994
O           2.311468        0.604463        1.236068
C           2.865044       -0.675463       -0.962684
H           3.063138       -1.427558       -0.199661
H           2.841562       -1.141181       -1.943821
H           3.653161        0.075597       -0.927265
H          -0.593743        0.743803       -1.382266
C          -2.085098        1.949649       -0.320227
H          -0.112056        1.550595        1.536800
H          -2.907243        1.605534       -0.945347
```

```
H         -2.446164      2.048419      0.703292
H         -1.783492      2.941094     -0.669572
S         -2.113234     -1.010833     -0.198058
C         -0.895314     -1.942755      0.748924
H         -0.183787     -1.231477      1.185274
H         -1.364757     -2.490155      1.565174
H         -0.340113     -2.647903      0.130792
```

3_methylcrotonate_TS_3_reopt

Datum	Value
M06-2X/def2tzvpp-IEFPCM(water) Energy	-783.981629
M06-2X/def2tzvpp-IEFPCM(water) Free Energy (Quasiharmonic)	-783.852733
Number of Imaginary Frequencies	1

Frequencies (Top 3 out of 54)

```
  1.    -201.4286 cm-1
  2.      35.2728 cm-1
  3.      71.1061 cm-1
```

M06-2X/def2tzvpp-IEFPCM(water) Molecular Geometry in Cartesian Coordinates

```
C          0.818835      0.980229      0.836074
C         -0.190475      0.127029      1.275071
C         -1.396688     -0.241172      0.611114
O         -1.560979      0.299399     -0.630348
O         -2.262171     -0.988268      1.065495
C         -2.763979     -0.043034     -1.301429
H         -3.634645      0.280636     -0.732715
H         -2.732623      0.472740     -2.256944
H         -2.829853     -1.118362     -1.461517
H          1.562757      1.177531      1.597440
C          0.623907      2.154174     -0.089885
H         -0.032887     -0.415450      2.197625
H          1.584059      2.612482     -0.323222
H          0.135867      1.871800     -1.015925
H          0.001679      2.903898      0.408552
S          2.412000     -0.162521     -0.469123
C          1.762286     -1.832862     -0.279709
H          2.438424     -2.477434      0.280773
H          0.813766     -1.767185      0.268779
H          1.563335     -2.295546     -1.245482
```

3_methylcrotonate_TS_4

Datum	Value
M06-2X/def2tzvpp-IEFPCM(water) Energy	-783.983201
M06-2X/def2tzvpp-IEFPCM(water) Free Energy (Quasiharmonic)	-783.855557
Number of Imaginary Frequencies	1

Frequencies (Top 3 out of 54)

```
1.    -247.1072 cm-1
2.      41.0215 cm-1
3.      56.3149 cm-1
```

M06-2X/def2tzvpp-IEFPCM(water) Molecular Geometry in Cartesian Coordinates

```
C          0.710980        0.836518        0.309397
C         -0.563496        0.946625       -0.216044
C         -1.600002        0.053436        0.176184
O         -2.779076        0.304139       -0.471713
O         -1.533031       -0.859235        0.994255
C         -3.869798       -0.530930       -0.117409
H         -4.708793       -0.208391       -0.727319
H         -3.643896       -1.576961       -0.320376
H         -4.117425       -0.424562        0.938296
H          0.809877        0.247573        1.214113
C          1.673869        1.981675        0.166049
H         -0.778936        1.660199       -0.999871
H          2.694370        1.694439        0.408085
H          1.652762        2.377935       -0.848817
H          1.376097        2.785467        0.846091
S          2.023179       -0.852132       -0.752883
C          3.324406       -0.908299        0.511367
H          2.935984       -0.518857        1.457193
H          3.660494       -1.929189        0.688303
H          4.189704       -0.306924        0.232829
```

3_methylcrotonate_TS_5

Datum	Value
M06-2X/def2tzvpp-IEFPCM(water) Energy	-783.981629
M06-2X/def2tzvpp-IEFPCM(water) Free Energy (Quasiharmonic)	-783.852732
Number of Imaginary Frequencies	1

Frequencies (Top 3 out of 54)

```
1.    -201.3974 cm-1
2.      35.3289 cm-1
3.      71.1130 cm-1
```

M06-2X/def2tzvpp-IEFPCM(water) Molecular Geometry in Cartesian Coordinates

```
C        -0.818768       0.980189      -0.836189
C         0.190482       0.126884      -1.275054
C         1.396721      -0.241255      -0.611067
O         1.561031       0.299431       0.630332
O         2.262183      -0.988397      -1.065402
C         2.764046      -0.042937       1.301425
H         2.732772       0.473041       2.256832
H         2.829852      -1.118237       1.461739
H         3.634699       0.280557       0.732594
H        -1.562726       1.177364      -1.597552
C        -0.623887       2.154188       0.089702
H         0.032874      -0.415711      -2.197536
H        -1.584060       2.612443       0.323048
H        -0.135796       1.871883       1.015738
H        -0.001724       2.903930      -0.408788
S        -2.412023      -0.162433       0.469214
C        -1.762452      -1.832821       0.279711
H        -2.438516      -2.477249      -0.281029
H        -0.813791      -1.767181      -0.268542
H        -1.563789      -2.295665       1.245466
```

3_methylcrotonate_TS_6

Datum	Value
M06-2X/def2tzvpp-IEFPCM(water) Energy	-783.9836
M06-2X/def2tzvpp-IEFPCM(water) Free Energy (Quasiharmonic)	-783.855044
Number of Imaginary Frequencies	1

Frequencies (Top 3 out of 54)

```
1.    -172.3500 cm-1
2.      17.5161 cm-1
3.      54.4248 cm-1
```

M06-2X/def2tzvpp-IEFPCM(water) Molecular Geometry in Cartesian Coordinates

```
C          0.761052        1.165619        0.349467
C         -0.468998        0.803333       -0.141018
C         -1.352258        0.007194        0.662478
O         -2.564623       -0.358506        0.152454
O         -1.122529       -0.385436        1.799358
C         -2.935410        0.018362       -1.164102
H         -2.970396        1.102617       -1.270806
H         -2.249082       -0.399096       -1.901614
H         -3.928485       -0.390629       -1.326413
H          0.927747        1.014390        1.406274
C          1.573180        2.242018       -0.306033
H         -0.732194        1.059347       -1.155917
H          2.620294        2.188167       -0.017214
H          1.507273        2.176198       -1.391549
H          1.186387        3.219563       -0.002675
S          2.377158       -0.704332       -0.055053
C          1.162303       -1.935225       -0.570140
H          1.447540       -2.412878       -1.506959
H          1.015333       -2.710865        0.181516
H          0.199049       -1.433773       -0.732197
```

3_methylcrotonate_TS_7

Datum	Value
M06-2X/def2tzvpp-IEFPCM(water) Energy	-783.981995
M06-2X/def2tzvpp-IEFPCM(water) Free Energy (Quasiharmonic)	-783.854066
Number of Imaginary Frequencies	1

Frequencies (Top 3 out of 54)

```
1.    -248.6699 cm-1
2.      52.5395 cm-1
3.      72.5029 cm-1
```

M06-2X/def2tzvpp-IEFPCM(water) Molecular Geometry in Cartesian Coordinates

```
C          0.598040        0.834971        0.424058
C         -0.572200        1.237499       -0.196204
C         -1.777571        0.480064       -0.206719
O         -1.704988       -0.690607        0.491267
O         -2.826900        0.794478       -0.764350
C         -2.894586       -1.462604        0.531448
H         -3.205031       -1.755428       -0.470635
H         -2.661460       -2.345616        1.119559
H         -3.705651       -0.907460        1.001323
H          0.521189        0.038468        1.153858
C          1.673185        1.853588        0.686089
H         -0.589245        2.144292       -0.786909
H          2.621095        1.393075        0.954122
H          1.825517        2.485308       -0.188696
H          1.360899        2.496537        1.514477
S          1.878372       -0.686187       -0.891473
C          3.018459       -1.192852        0.426949
H          3.951885       -0.630764        0.397717
H          2.553465       -1.020844        1.402199
H          3.256524       -2.253552        0.357490
```

3_methylcrotonate_TS_8_reopt

Datum	Value
M06-2X/def2tzvpp-IEFPCM(water) Energy	-783.983668
M06-2X/def2tzvpp-IEFPCM(water) Free Energy (Quasiharmonic)	-783.854279
Number of Imaginary Frequencies	2

Frequencies (Top 3 out of 54)

```
1.    -196.0135 cm-1
2.     -18.0050 cm-1
3.      60.4262 cm-1
```

M06-2X/def2tzvpp-IEFPCM(water) Molecular Geometry in Cartesian Coordinates

```
C          0.994902       1.028900       0.727328
C         -0.225059       0.427302       1.008388
C         -1.282729       0.262646       0.068091
O         -2.367938      -0.363378       0.627491
O         -1.318359       0.595105      -1.112649
C         -3.469429      -0.581409      -0.238368
H         -3.186589      -1.210625      -1.081780
H         -4.228399      -1.081150       0.356890
H         -3.860338       0.360827      -0.621055
H          1.633088       1.155837       1.592290
C          1.178051       2.105772      -0.309799
H         -0.363703      -0.059312       1.963630
H          2.234437       2.329402      -0.449100
H          0.742586       1.822074      -1.262061
H          0.679615       3.018092       0.033318
S          2.513202      -0.543313      -0.201071
C          1.464019      -2.006247      -0.115324
H          1.878417      -2.771600       0.539967
H          0.487549      -1.703182       0.284818
H          1.303942      -2.442953      -1.100421
```

4_methylmethacrylate_1

Datum	Value
M06-2X/def2tzvpp-IEFPCM(water) Energy	-345.781977
M06-2X/def2tzvpp-IEFPCM(water) Free Energy (Quasiharmonic)	-345.686401
Number of Imaginary Frequencies	0

Frequencies (Top 3 out of 39)

```
 1.      55.5385 cm-1
 2.     133.7581 cm-1
 3.     184.2183 cm-1
```

M06-2X/def2tzvpp-IEFPCM(water) Molecular Geometry in Cartesian Coordinates

```
C         -1.352467       1.569923      -0.000002
C         -1.143122       0.257927      -0.000000
C          0.244063      -0.293814       0.000000
O          1.198880       0.635868      -0.000000
O          0.481885      -1.478367       0.000001
C          2.541692       0.149287      -0.000000
```

```
H          3.176131       1.028650      -0.000000
H          2.724467      -0.452268       0.887849
H          2.724468      -0.452268      -0.887850
H         -0.532955       2.273560      -0.000003
H         -2.359078       1.967186      -0.000002
C         -2.233525      -0.768580       0.000001
H         -2.155157      -1.412840       0.876034
H         -3.208684      -0.287635       0.000001
H         -2.155157      -1.412843      -0.876030
```

4_methylmethacrylate_2

Datum	Value
M06-2X/def2tzvpp-IEFPCM(water) Energy	-345.781618
M06-2X/def2tzvpp-IEFPCM(water) Free Energy (Quasiharmonic)	-345.686015
Number of Imaginary Frequencies	0

Frequencies (Top 3 out of 39)

```
1.      46.3408 cm-1
2.     146.4326 cm-1
3.     172.9723 cm-1
```

M06-2X/def2tzvpp-IEFPCM(water) Molecular Geometry in Cartesian Coordinates

```
C         -2.107648      -0.923423       0.000000
C         -1.186849       0.033369       0.000001
C          0.246950      -0.394877       0.000001
O          1.076821       0.648847      -0.000003
O          0.633141      -1.538554       0.000005
C          2.471255       0.337820      -0.000003
H          2.988866       1.290546      -0.000006
H          2.727641      -0.236124       0.887795
H          2.727640      -0.236128      -0.887800
H         -1.820874      -1.965858       0.000000
H         -3.162218      -0.682545      -0.000000
C         -1.495808       1.499864       0.000000
H         -1.068072       1.987185      -0.876210
H         -2.572003       1.656879       0.000001
H         -1.068071       1.987185       0.876210
```

4_methylmethacrylate_3

Datum	Value
M06-2X/def2tzvpp-IEFPCM(water) Energy	-345.769113
M06-2X/def2tzvpp-IEFPCM(water) Free Energy (Quasiharmonic)	-345.674082
Number of Imaginary Frequencies	0

Frequencies (Top 3 out of 39)

```
1.      48.2441 cm-1
2.     120.7213 cm-1
3.     172.4030 cm-1
```

M06-2X/def2tzvpp-IEFPCM(water) Molecular Geometry in Cartesian Coordinates

```
C          -0.705098       -1.467959       -0.813545
C          -0.838914       -0.363613       -0.090615
C           0.188558        0.724030       -0.186450
O           1.486408        0.439721       -0.047005
O          -0.130942        1.863143       -0.418706
C           1.948615       -0.781960        0.540165
H           2.897525       -0.540428        1.008825
H           1.250179       -1.148441        1.287318
H           2.101372       -1.537208       -0.226342
H           0.170859       -1.655609       -1.419706
H          -1.491203       -2.211437       -0.836361
C          -2.046728       -0.030009        0.731333
H          -2.506105        0.889268        0.368633
H          -2.774301       -0.836735        0.683883
H          -1.770652        0.134747        1.774114
```

4_methylmethacrylate_HEI_1_reopt

Datum	Value
M06-2X/def2tzvpp-IEFPCM(water) Energy	-783.991114
M06-2X/def2tzvpp-IEFPCM(water) Free Energy (Quasiharmonic)	-783.863091
Number of Imaginary Frequencies	0

Frequencies (Top 3 out of 54)

```
1.      32.6006 cm-1
2.      59.7670 cm-1
3.      87.6195 cm-1
```

M06-2X/def2tzvpp-IEFPCM(water) Molecular Geometry in Cartesian Coordinates

```
C        -1.165592        0.436021        1.059507
C         0.071501        0.803286        0.347978
C         1.125295       -0.088915        0.359941
O         2.218253        0.314203       -0.423086
O         1.230740       -1.181696        0.971361
C         3.363221       -0.504891       -0.345757
H         3.158637       -1.521148       -0.684092
H         4.108846       -0.050945       -0.996016
H         3.755903       -0.557219        0.671167
H        -0.994652       -0.375600        1.762922
H        -1.620852        1.276349        1.591282
C         0.042949        2.040276       -0.503920
H        -0.486889        2.852064        0.007104
H         1.045561        2.395399       -0.735903
H        -0.478739        1.894499       -1.460260
C        -1.758288       -1.402223       -0.977777
H        -0.791981       -1.026411       -1.314241
H        -1.607383       -2.294507       -0.372877
H        -2.375169       -1.649298       -1.838786
S        -2.586234       -0.106535       -0.041020
```

4_methylmethacrylate_HEI_2

Datum	Value
M06-2X/def2tzvpp-IEFPCM(water) Energy	-783.991178
M06-2X/def2tzvpp-IEFPCM(water) Free Energy (Quasiharmonic)	-783.863254
Number of Imaginary Frequencies	0

Frequencies (Top 3 out of 54)

```
1.      32.2468 cm-1
2.      44.8667 cm-1
3.      76.7734 cm-1
```

M06-2X/def2tzvpp-IEFPCM(water) Molecular Geometry in Cartesian Coordinates

```
C        -0.983207       0.298279      -1.076062
C         0.031138       1.020860      -0.286742
C         1.253403       0.465553       0.035180
O         1.468671      -0.810843      -0.510241
O         2.180374       0.953677       0.728845
C         2.716090      -1.398448      -0.220144
H         2.714039      -2.374534      -0.702107
H         3.545475      -0.805921      -0.609647
H         2.863925      -1.525938       0.853272
H        -0.561356      -0.522112      -1.650043
H        -1.526535       0.957270      -1.759297
C        -0.361956       2.349620       0.290231
H         0.500279       2.852534       0.725898
H        -0.787659       3.012913      -0.472281
H        -1.123439       2.263973       1.077273
C        -1.445615      -1.521087       1.001641
H        -2.101635      -1.868026       1.796800
H        -1.048717      -2.376857       0.458505
H        -0.620263      -0.952465       1.429356
S        -2.394099      -0.443261      -0.085074
```

4_methylmethacrylate_HEI_3

Datum	Value
M06-2X/def2tzvpp-IEFPCM(water) Energy	-783.988881
M06-2X/def2tzvpp-IEFPCM(water) Free Energy (Quasiharmonic)	-783.860743
Number of Imaginary Frequencies	0

Frequencies (Top 3 out of 54)

```
1.     42.3674 cm-1
2.     61.4810 cm-1
3.     77.9003 cm-1
```

M06-2X/def2tzvpp-IEFPCM(water) Molecular Geometry in Cartesian Coordinates

```
C        -1.061221      -0.284539       1.078202
C         0.116624       0.448510       0.571912
```

```
C          1.220177      -0.284478       0.190491
O          2.281984       0.507266      -0.270979
O          1.387604      -1.529091       0.206828
C          3.461798      -0.187419      -0.609330
H          3.290912      -0.910882      -1.407468
H          4.173828       0.563099      -0.947538
H          3.878232      -0.718746       0.248046
H         -0.784051      -1.215554       1.572912
H         -1.645324       0.319194       1.775718
C          0.080519       1.952577       0.464679
H         -0.809972       2.342162       0.964185
H          0.946793       2.427844       0.933862
H          0.047874       2.329948      -0.564680
C         -2.684732       0.697731      -0.973402
H         -3.085135       1.405519      -0.247881
H         -1.786197       1.113462      -1.424602
H         -3.428157       0.522527      -1.747882
S         -2.309656      -0.878641      -0.186049
```

4_methylmethacrylate_HEI_4_reopt

Datum	Value
M06-2X/def2tzvpp-IEFPCM(water) Energy	-783.988774
M06-2X/def2tzvpp-IEFPCM(water) Free Energy (Quasiharmonic)	-783.860216
Number of Imaginary Frequencies	0

Frequencies (Top 3 out of 54)

```
1.      45.8043 cm-1
2.      70.8177 cm-1
3.      93.2099 cm-1
```

M06-2X/def2tzvpp-IEFPCM(water) Molecular Geometry in Cartesian Coordinates

```
C          0.806889      -0.215120       1.073685
C         -0.084132       0.775729       0.435670
C         -1.337955       0.440061      -0.028516
O         -1.682831      -0.907246       0.151493
O         -2.183117       1.188599      -0.578630
C         -2.995613      -1.259728      -0.223103
H         -3.094700      -2.325672      -0.026561
H         -3.742790      -0.715708       0.357570
H         -3.180370      -1.067864      -1.280554
```

```
H          0.260097      -1.006738       1.583257
H          1.478088       0.256830       1.794432
C          0.379642       2.198321       0.254287
H         -0.345154       2.922237       0.640450
H          1.319503       2.364458       0.785903
H          0.557218       2.478109      -0.791727
C          2.815419       0.170233      -0.835927
H          3.549568      -0.246177      -1.522102
H          2.114036       0.788672      -1.392126
H          3.327729       0.783051      -0.094576
S          1.948679      -1.196188      -0.043341
```

4_methylmethacrylate_HEI_5

Datum	Value
M06-2X/def2tzvpp-IEFPCM(water) Energy	-783.989792
M06-2X/def2tzvpp-IEFPCM(water) Free Energy (Quasiharmonic)	-783.861989
Number of Imaginary Frequencies	0

Frequencies (Top 3 out of 54)

```
1.      48.9600 cm-1
2.      55.5320 cm-1
3.      77.2881 cm-1
```

M06-2X/def2tzvpp-IEFPCM(water) Molecular Geometry in Cartesian Coordinates

```
C         -1.037550       0.117362       0.760124
C          0.288263       0.636186       0.356711
C          1.320430      -0.264655       0.201831
O          2.536104       0.312348      -0.198136
O          1.307210      -1.509501       0.379585
C          3.621519      -0.577135      -0.330647
H          3.853660      -1.077270       0.610652
H          3.428816      -1.342744      -1.083346
H          4.474690       0.024518      -0.638770
H         -0.960803      -0.844994       1.263669
H         -1.575224       0.817304       1.406795
C          0.392018       2.105430       0.057925
H          0.015194       2.713567       0.889586
H          1.422116       2.403757      -0.124883
H         -0.193494       2.402911      -0.822085
C         -3.693263      -0.560467       0.157617
```

```
H          -3.566421       -1.458005        0.761297
H          -4.008757        0.262587        0.797497
H          -4.462898       -0.742334       -0.589223
S          -2.157618       -0.145151       -0.696508
```

4_methylmethacrylate_HEI_6

Datum	Value
M06-2X/def2tzvpp-IEFPCM(water) Energy	-783.989833
M06-2X/def2tzvpp-IEFPCM(water) Free Energy (Quasiharmonic)	-783.861945
Number of Imaginary Frequencies	0

Frequencies (Top 3 out of 54)

```
1.      50.0531 cm-1
2.      57.8527 cm-1
3.      80.5713 cm-1
```

M06-2X/def2tzvpp-IEFPCM(water) Molecular Geometry in Cartesian Coordinates

```
C          -0.861140        0.153080        0.743148
C           0.332313        0.907000        0.297453
C           1.530257        0.289633        0.005411
O           1.512954       -1.104274        0.184274
O           2.607993        0.803130       -0.386364
C           2.746704       -1.761286        0.002669
H           3.502411       -1.408378        0.706855
H           2.558843       -2.819013        0.178428
H           3.133883       -1.626068       -1.007848
H          -1.462027        0.729155        1.453477
H          -0.610948       -0.804937        1.192910
C           0.170960        2.384894        0.084374
H           1.116666        2.833733       -0.215949
H          -0.567281        2.622557       -0.692385
H          -0.168275        2.899293        0.992295
C          -3.424692       -0.884543        0.252203
H          -4.200539       -1.153087       -0.461397
H          -3.134239       -1.773568        0.810220
H          -3.820341       -0.139645        0.941342
S          -2.017634       -0.217722       -0.662049
```

4_methylmethacrylate_TS_1

Datum	Value
M06-2X/def2tzvpp-IEFPCM(water) Energy	-783.984906
M06-2X/def2tzvpp-IEFPCM(water) Free Energy (Quasiharmonic)	-783.856529
Number of Imaginary Frequencies	1

Frequencies (Top 3 out of 54)

```
1.    -226.9363 cm-1
2.      50.3544 cm-1
3.      59.7216 cm-1
```

M06-2X/def2tzvpp-IEFPCM(water) Molecular Geometry in Cartesian Coordinates

```
C         -1.022812        0.822892        1.118926
C          0.108629        0.965619        0.326522
C          1.129713       -0.022813        0.443896
O          2.175011        0.185149       -0.418252
O          1.158650       -0.984957        1.212201
C          3.233640       -0.754966       -0.345502
H          2.882642       -1.758917       -0.582312
H          3.970505       -0.439263       -1.078833
H          3.681076       -0.766800        0.647681
H         -0.959515        0.166405        1.973731
H         -1.674861        1.676609        1.243759
C          0.169640        2.014736       -0.750654
H         -0.580161        2.784172       -0.561329
H          1.144074        2.501722       -0.800152
H         -0.032329        1.606410       -1.747671
C         -1.627243       -1.203835       -1.154048
H         -0.634072       -0.740381       -1.112544
H         -1.514935       -2.267929       -0.949531
H         -2.016104       -1.079427       -2.163824
S         -2.680313       -0.388371        0.059037
```

4_methylmethacrylate_TS_2

Datum	Value
M06-2X/def2tzvpp-IEFPCM(water) Energy	-783.985082

Datum	Value
M06-2X/def2tzvpp-IEFPCM(water) Free Energy (Quasiharmonic)	-783.857114
Number of Imaginary Frequencies	1

Frequencies (Top 3 out of 54)

```
1.    -230.3728 cm-1
2.      51.3551 cm-1
3.      66.6325 cm-1
```

M06-2X/def2tzvpp-IEFPCM(water) Molecular Geometry in Cartesian Coordinates

```
C         -0.795335        0.475986       -1.286773
C          0.104954        1.065783       -0.408033
C          1.286459        0.389229        0.012536
O          1.470437       -0.840796       -0.562475
O          2.121938        0.816539        0.810808
C          2.642755       -1.534412       -0.171050
H          2.628546       -2.479028       -0.707684
H          3.538976       -0.973385       -0.434001
H          2.648991       -1.718958        0.902898
H         -0.466307       -0.365705       -1.875675
H         -1.519374        1.114572       -1.775099
C         -0.227025        2.360630        0.279496
H          0.649782        3.001186        0.382437
H         -0.983482        2.906959       -0.285513
H         -0.626560        2.205687        1.289115
C         -1.546382       -0.991403        1.333940
H         -2.139164       -0.731782        2.209910
H         -1.228576       -2.029838        1.420954
H         -0.651545       -0.357646        1.328368
S         -2.461428       -0.685805       -0.187691
```

4_methylmethacrylate_TS_3_reopt

Datum	Value
M06-2X/def2tzvpp-IEFPCM(water) Energy	-783.984906
M06-2X/def2tzvpp-IEFPCM(water) Free Energy (Quasiharmonic)	-783.856525
Number of Imaginary Frequencies	1

Frequencies (Top 3 out of 54)

```
1.    -226.7038 cm-1
2.      50.3637 cm-1
3.      59.7119 cm-1
```

M06-2X/def2tzvpp-IEFPCM(water) Molecular Geometry in Cartesian Coordinates

```
C          1.022633        0.823060       -1.118917
C         -0.108671        0.965650       -0.326466
C         -1.129748       -0.022877       -0.443931
O         -2.175027        0.185046        0.418167
O         -1.158587       -0.984991       -1.212272
C         -3.233525       -0.755247        0.345577
H         -2.882334       -1.759112        0.582458
H         -3.970385       -0.439584        1.078926
H         -3.681018       -0.767237       -0.647574
H          0.959504        0.166305       -1.973520
H          1.674745        1.676727       -1.243721
C         -0.170164        2.014857        0.750599
H          0.579988        2.784079        0.561809
H         -1.144521        2.502096        0.799288
H          0.030887        1.606518        1.747760
C          1.627272       -1.203702        1.153952
H          2.015761       -1.079003        2.163838
H          0.634008       -0.740434        1.111976
H          1.515167       -2.267868        0.949684
S          2.680645       -0.388336       -0.058935
```

4_methylmethacrylate_TS_4_reopt

Datum	Value
M06-2X/def2tzvpp-IEFPCM(water) Energy	-783.985082
M06-2X/def2tzvpp-IEFPCM(water) Free Energy (Quasiharmonic)	-783.857112
Number of Imaginary Frequencies	1

Frequencies (Top 3 out of 54)

```
1.    -230.5716 cm-1
2.      51.3820 cm-1
3.      66.6349 cm-1
```

M06-2X/def2tzvpp-IEFPCM(water) Molecular Geometry in Cartesian Coordinates

```
C         -0.795672        0.475729       -1.286543
C          0.104865        1.065673       -0.407996
C          1.286366        0.389249        0.012444
O          1.470379       -0.840867       -0.562483
O          2.121980        0.816638        0.810603
C          2.642752       -1.534321       -0.170962
H          2.628476       -2.479162       -0.707203
H          3.538946       -0.973413       -0.434271
H          2.649173       -1.718426        0.903062
H         -0.466491       -0.365778       -1.875637
H         -1.519521        1.114444       -1.774995
C         -0.226992        2.360608        0.279427
H          0.649563        3.001731        0.381078
H         -0.984407        2.906291       -0.284923
H         -0.625227        2.205878        1.289596
C         -1.546400       -0.991356        1.334025
H         -1.228600       -2.029786        1.421070
H         -0.651606       -0.357543        1.328501
H         -2.139301       -0.731719        2.209906
S         -2.461212       -0.685761       -0.187720
```

4_methylmethacrylate_TS_5_reopt

Datum	Value
M06-2X/def2tzvpp-IEFPCM(water) Energy	-783.979739
M06-2X/def2tzvpp-IEFPCM(water) Free Energy (Quasiharmonic)	-783.853053
Number of Imaginary Frequencies	1

Frequencies (Top 3 out of 54)

```
1.    -251.7318 cm-1
2.      35.0702 cm-1
3.      53.5001 cm-1
```

M06-2X/def2tzvpp-IEFPCM(water) Molecular Geometry in Cartesian Coordinates

```
```

```
C          0.878539       0.368486       -0.834486
C         -0.390039       0.746858       -0.428634
C         -1.363510      -0.281504       -0.246701
O         -2.562311       0.197833        0.207559
O         -1.229815      -1.487015       -0.452237
C         -3.587362      -0.765930        0.385271
H         -3.821665      -1.266264       -0.553687
H         -3.295666      -1.517115        1.118284
H         -4.457359      -0.220728        0.740397
H          1.004498      -0.608730       -1.279743
H          1.555152       1.130080       -1.201289
C         -0.679408       2.156396        0.010814
H          0.114953       2.821912       -0.329684
H         -1.623235       2.530221       -0.388217
H         -0.738797       2.252243        1.100621
C          3.674224      -0.613438       -0.368399
H          3.475511      -1.599180       -0.789571
H          3.692461       0.107545       -1.188938
H          4.663726      -0.629184        0.085743
S          2.410798      -0.147034        0.846020
```

4_methylmethacrylate_TS_6

Datum	Value
M06-2X/def2tzvpp-IEFPCM(water) Energy	-783.979973
M06-2X/def2tzvpp-IEFPCM(water) Free Energy (Quasiharmonic)	-783.853631
Number of Imaginary Frequencies	1

Frequencies (Top 3 out of 54)

```
1.    -257.3413 cm-1
2.      32.3660 cm-1
3.      58.6917 cm-1
```

M06-2X/def2tzvpp-IEFPCM(water) Molecular Geometry in Cartesian Coordinates

```
C         -0.710746       0.381996        0.815353
C          0.453389       0.983814        0.364932
C          1.606577       0.213685        0.034613
O          1.481538      -1.128162        0.271350
O          2.659535       0.654611       -0.425482
C          2.623956      -1.915606       -0.019834
```

```
H          3.477376      -1.608388       0.583820
H          2.353253      -2.940424       0.218525
H          2.894665      -1.839373      -1.072256
H         -1.469074       1.011557       1.264907
H         -0.663445      -0.621518       1.212394
C          0.483945       2.441024      -0.001789
H          1.402008       2.925863       0.333532
H          0.423147       2.598648      -1.084883
H         -0.359952       2.963324       0.450519
C         -3.463875      -0.728855       0.466323
H         -4.328380      -1.217238       0.019532
H         -3.024209      -1.415793       1.191799
H         -3.812662       0.153783       1.004179
S         -2.248799      -0.279899      -0.802912
```

5_methyltiglate_1

Datum	Value
M06-2X/def2tzvpp-IEFPCM(water) Energy	-385.094038
M06-2X/def2tzvpp-IEFPCM(water) Free Energy (Quasiharmonic)	-384.97239
Number of Imaginary Frequencies	0

Frequencies (Top 3 out of 48)

```
1.      49.6713 cm-1
2.     124.2600 cm-1
3.     133.7189 cm-1
```

M06-2X/def2tzvpp-IEFPCM(water) Molecular Geometry in Cartesian Coordinates

```
C          1.705103      -0.633504      -0.000020
C          0.684477       0.226314       0.000008
C         -0.682110      -0.374401      -0.000009
O         -1.636593       0.560915       0.000017
O         -0.935981      -1.556432      -0.000039
C         -2.981280       0.081538      -0.000006
H         -3.612202       0.963581       0.000078
H         -3.167178      -0.519565      -0.887526
H         -3.167154      -0.519720       0.887413
H          1.447008      -1.687159      -0.000053
C          3.162405      -0.323801      -0.000013
C          0.783835       1.723307       0.000057
H          0.288074       2.142495       0.875740
```

```
H          1.817775      2.054128      0.000051
H          0.288047      2.142553     -0.875583
H          3.636073     -0.774452     -0.874332
H          3.379460      0.740216      0.000108
H          3.636111     -0.774661      0.874176
```

5_methyltiglate_2

Datum	Value
M06-2X/def2tzvpp-IEFPCM(water) Energy	-385.094139
M06-2X/def2tzvpp-IEFPCM(water) Free Energy (Quasiharmonic)	-384.97299
Number of Imaginary Frequencies	0

Frequencies (Top 3 out of 48)

```
1.      48.4999 cm-1
2.     110.5146 cm-1
3.     115.5529 cm-1
```

M06-2X/def2tzvpp-IEFPCM(water) Molecular Geometry in Cartesian Coordinates

```
C          1.361073     -0.852407      0.000010
C          0.724579      0.321389     -0.000011
C         -0.764157      0.354631     -0.000005
O         -1.348809     -0.846546      0.000020
O         -1.401171      1.383270     -0.000021
C         -2.775897     -0.843582      0.000033
H         -3.153243     -0.340003      0.887582
H         -3.153259     -0.340049     -0.887535
H         -3.075099     -1.886021      0.000063
H          0.750890     -1.747546      0.000033
C          2.833478     -1.086421      0.000008
C          1.362116      1.678742     -0.000039
H          1.046479      2.247044      0.875292
H          2.445565      1.617780     -0.000013
H          1.046517      2.246993     -0.875418
H          3.113356     -1.677318     -0.874244
H          3.418106     -0.171604     -0.000071
H          3.113380     -1.677183      0.874345
```

5_methyltiglate_3

Datum	Value
M06-2X/def2tzvpp-IEFPCM(water) Energy	-385.080926
M06-2X/def2tzvpp-IEFPCM(water) Free Energy (Quasiharmonic)	-384.960136
Number of Imaginary Frequencies	0

Frequencies (Top 3 out of 48)

```
1.      50.7450 cm-1
2.      99.1487 cm-1
3.     118.0540 cm-1
```

M06-2X/def2tzvpp-IEFPCM(water) Molecular Geometry in Cartesian Coordinates

```
C         -1.160761       -0.628756       -0.406362
C         -0.475904        0.407925        0.076416
C          0.974018        0.531829       -0.265739
O          1.782617       -0.535510       -0.197770
O          1.444910        1.577328       -0.642746
C          1.495260       -1.677000        0.616040
H          2.450042       -2.004457        1.017088
H          0.820513       -1.423377        1.428695
H          1.063366       -2.471442        0.012302
H         -0.609846       -1.384991       -0.956048
C         -2.630086       -0.871317       -0.311677
C         -1.036970        1.589616        0.811402
H         -0.912813        2.493817        0.215581
H         -2.090717        1.463698        1.038574
H         -0.499556        1.745839        1.747910
H         -2.816397       -1.832364        0.171177
H         -3.156985       -0.099065        0.240585
H         -3.061172       -0.935982       -1.312221
```

5_methyltiglate_4

Datum	Value
M06-2X/def2tzvpp-IEFPCM(water) Energy	-385.081265
M06-2X/def2tzvpp-IEFPCM(water) Free Energy (Quasiharmonic)	-384.959662

Datum	Value
Number of Imaginary Frequencies	0

Frequencies (Top 3 out of 48)

```
1.      63.5213 cm-1
2.     114.5190 cm-1
3.     142.4851 cm-1
```

M06-2X/def2tzvpp-IEFPCM(water) Molecular Geometry in Cartesian Coordinates

```
C        -1.527943        0.507370       -0.377373
C        -0.466410       -0.019315        0.231970
C         0.800884        0.769975        0.172470
O         1.943835        0.123913       -0.107703
O         0.858182        1.956103        0.384644
C         1.940445       -1.165533       -0.725190
H         2.824568       -1.199050       -1.354375
H         1.999308       -1.947156        0.028439
H         1.053600       -1.303417       -1.340328
H        -1.400690        1.462374       -0.876147
C        -2.885614       -0.104649       -0.450992
C        -0.477270       -1.315221        1.004684
H         0.402078       -1.401187        1.640586
H        -1.354365       -1.355581        1.648142
H        -0.503862       -2.187206        0.350070
H        -3.184039       -0.230846       -1.493054
H        -2.935294       -1.072777        0.040996
H        -3.621986        0.558950        0.006725
```

5_methyltiglate_HEI_1

Datum	Value
M06-2X/def2tzvpp-IEFPCM(water) Energy	-823.299953
M06-2X/def2tzvpp-IEFPCM(water) Free Energy (Quasiharmonic)	-823.144884
Number of Imaginary Frequencies	0

Frequencies (Top 3 out of 63)

```
1.      49.2463 cm-1
2.      52.1951 cm-1
3.      82.4748 cm-1
```

M06-2X/def2tzvpp-IEFPCM(water) Molecular Geometry in Cartesian Coordinates

```
C         -1.125600        0.465283       -0.635048
C          0.138742        0.707966        0.092471
C          1.266271        0.027787       -0.321977
O          2.417623        0.308927        0.432515
O          1.391954       -0.786136       -1.271779
C          3.595510       -0.347763        0.022176
H          3.488480       -1.432699        0.054325
H          3.885862       -0.065043       -0.990946
H          4.374884       -0.040765        0.717432
H         -0.910994       -0.038064       -1.575695
C         -1.966775        1.711613       -0.893118
C          0.100294        1.601399        1.300953
H          1.016850        1.521123        1.881173
H         -0.037707        2.662054        1.056451
H         -0.733189        1.331195        1.961778
H         -1.389158        2.430450       -1.476626
H         -2.879070        1.472827       -1.441807
H         -2.251528        2.192493        0.043929
S         -2.304050       -0.708950        0.262256
C         -1.209688       -2.114164        0.522923
H         -1.681007       -2.783965        1.238953
H         -1.023832       -2.649356       -0.406072
H         -0.263923       -1.752102        0.924836
```

5_methyltiglate_HEI_2

Datum	Value
M06-2X/def2tzvpp-IEFPCM(water) Energy	-823.300044
M06-2X/def2tzvpp-IEFPCM(water) Free Energy (Quasiharmonic)	-823.144707
Number of Imaginary Frequencies	0

Frequencies (Top 3 out of 63)

```
1.      43.3060 cm-1
2.      57.8526 cm-1
3.      92.3374 cm-1
```

M06-2X/def2tzvpp-IEFPCM(water) Molecular Geometry in Cartesian Coordinates

```
C          0.976682      -0.254071      -0.725029
C         -0.035673      -0.930042       0.114665
C         -1.358484      -0.533461       0.138329
O         -1.664123       0.520935      -0.738130
O         -2.304445      -0.995535       0.824563
C         -2.992795       0.987565      -0.696245
H         -3.054746       1.799502      -1.418791
H         -3.706925       0.208658      -0.966542
H         -3.258302       1.364768       0.292926
H          0.496451       0.347775      -1.491800
C          1.989821      -1.192820      -1.373870
C          0.429061      -1.997167       1.065432
H         -0.363288      -2.243684       1.770776
H          0.727310      -2.929422       0.570172
H          1.300847      -1.666256       1.643934
H          1.468768      -1.912907      -2.006841
H          2.706763      -0.645715      -1.987678
H          2.545927      -1.751040      -0.619401
S          2.046076       0.986878       0.225147
C          0.762683       2.036495       0.925187
H          1.220235       2.675979       1.676952
H          0.294983       2.653420       0.160256
H          0.005527       1.406684       1.391410
```

5_methyltiglate_HEI_3_reopt

Datum	Value
M06-2X/def2tzvpp-IEFPCM(water) Energy	-823.298578
M06-2X/def2tzvpp-IEFPCM(water) Free Energy (Quasiharmonic)	-823.143052
Number of Imaginary Frequencies	0

Frequencies (Top 3 out of 63)

```
1.     55.1829 cm-1
2.     65.5468 cm-1
3.     85.6906 cm-1
```

M06-2X/def2tzvpp-IEFPCM(water) Molecular Geometry in Cartesian Coordinates

```
C          0.922559       0.234411       0.553382
C         -0.414884       0.628651       0.032275
C         -1.465236      -0.242556       0.227289
O         -2.691119       0.205744      -0.292223
O         -1.464373      -1.359179       0.807040
C         -3.791229      -0.651851      -0.090972
H         -3.634513      -1.628778      -0.550276
H         -3.997645      -0.804476       0.969577
H         -4.646945      -0.166787      -0.557350
H          0.802193      -0.544352       1.305756
C          1.737208       1.386188       1.128292
C         -0.527391       1.916988      -0.736069
H          0.244057       1.992913      -1.512600
H         -1.493669       1.992789      -1.229740
H         -0.411813       2.809959      -0.108724
H          1.176149       1.860421       1.936776
H          2.690822       1.047788       1.533332
H          1.939536       2.140852       0.367361
S          1.871905      -0.590143      -0.823743
C          3.433310      -1.037416      -0.026830
H          4.075502      -0.172780       0.125790
H          3.238275      -1.518017       0.931656
H          3.945472      -1.746266      -0.674420
```

5_methyltiglate_HEI_4

Datum	Value
M06-2X/def2tzvpp-IEFPCM(water) Energy	-823.294761
M06-2X/def2tzvpp-IEFPCM(water) Free Energy (Quasiharmonic)	-823.139482
Number of Imaginary Frequencies	0

Frequencies (Top 3 out of 63)

```
1.      20.9913 cm-1
2.      71.8347 cm-1
3.      84.1060 cm-1
```

M06-2X/def2tzvpp-IEFPCM(water) Molecular Geometry in Cartesian Coordinates

```
C          -0.849911        0.615040        0.502768
C           0.316162       -0.293639        0.348219
C           1.535483        0.276545        0.047034
O           2.560048       -0.655893       -0.190219
O           1.844015        1.494305       -0.038811
C           3.852168       -0.116167       -0.351505
H           3.910284        0.548822       -1.213867
H           4.170340        0.442684        0.530380
H           4.521108       -0.961711       -0.502017
H          -0.482161        1.639354        0.463516
C          -1.656003        0.434502        1.786583
C           0.080832       -1.777883        0.303874
H           1.002744       -2.331727        0.470760
H          -0.633908       -2.101025        1.067634
H          -0.326864       -2.118272       -0.659266
H          -1.009887        0.635878        2.642303
H          -2.510089        1.112475        1.826567
H          -2.031349       -0.583732        1.889406
S          -2.024828        0.608586       -0.965437
C          -3.155214       -0.748025       -0.581270
H          -3.777312       -0.898443       -1.461355
H          -2.612630       -1.667050       -0.372091
H          -3.796627       -0.504164        0.263038
```

5_methyltiglate_HEI_5

Datum	Value
M06-2X/def2tzvpp-IEFPCM(water) Energy	-823.294385
M06-2X/def2tzvpp-IEFPCM(water) Free Energy (Quasiharmonic)	-823.138231
Number of Imaginary Frequencies	0

Frequencies (Top 3 out of 63)

```
1.      36.1335 cm-1
2.      70.0752 cm-1
3.      90.3460 cm-1
```

M06-2X/def2tzvpp-IEFPCM(water) Molecular Geometry in Cartesian Coordinates

```
C           0.632106       -0.471027        0.544044
C          -0.285681        0.650932        0.211401
C          -1.621011        0.449557       -0.068798
```

```
O          -2.056200       -0.883006        0.062892
O          -2.485252        1.295417       -0.414918
C          -3.434593       -1.100867       -0.132440
H          -3.594204       -2.171660       -0.017997
H          -4.038643       -0.567502        0.603916
H          -3.758900       -0.790931       -1.126364
H           0.059191       -1.392684        0.607389
C           1.411622       -0.303714        1.847304
C           0.299864        2.015344       -0.019609
H          -0.488701        2.765900       -0.054470
H           1.003259        2.311762        0.765797
H           0.852030        2.083663       -0.968177
H           0.704735       -0.253977        2.676721
H           2.094893       -1.136854        2.020392
H           1.994926        0.616932        1.854632
S           1.834992       -0.902026       -0.836156
C           3.248286        0.176212       -0.510814
H           3.922530        0.073630       -1.358837
H           2.941621        1.216450       -0.428400
H           3.775448       -0.120219        0.393566
```

5_methyltiglate_HEI_6

Datum	Value
M06-2X/def2tzvpp-IEFPCM(water) Energy	-823.298384
M06-2X/def2tzvpp-IEFPCM(water) Free Energy (Quasiharmonic)	-823.14299
Number of Imaginary Frequencies	0

Frequencies (Top 3 out of 63)

```
1.      45.8415 cm-1
2.      61.1048 cm-1
3.      86.4789 cm-1
```

M06-2X/def2tzvpp-IEFPCM(water) Molecular Geometry in Cartesian Coordinates

```
C           0.764701        0.178909        0.555434
C          -0.435817        0.880471        0.023563
C          -1.661867        0.259216       -0.089382
O          -1.672823       -1.081221        0.333266
O          -2.746802        0.731895       -0.513514
C          -2.933159       -1.712227        0.334585
H          -2.766720       -2.727668        0.689815
```

```
H         -3.637520      -1.209119       0.999429
H         -3.371870      -1.745507      -0.663246
H          0.468803      -0.700347       1.124138
C          1.664700       1.058928       1.415231
C         -0.274181       2.289396      -0.476763
H          0.571736       2.385868      -1.168521
H         -1.173121       2.598904      -1.008128
H         -0.098260       3.021581       0.321345
H          2.509313       0.502816       1.821614
H          2.055251       1.899444       0.840628
H          1.088364       1.458212       2.252871
S          1.739799      -0.503658      -0.883469
C          3.141150      -1.313143      -0.074453
H          3.867769      -0.594138       0.297515
H          2.789024      -1.932488       0.750298
H          3.624296      -1.953718      -0.809555
```

5_methyltiglate_TS_1

Datum	Value
M06-2X/def2tzvpp-IEFPCM(water) Energy	-823.29476
M06-2X/def2tzvpp-IEFPCM(water) Free Energy (Quasiharmonic)	-823.140365
Number of Imaginary Frequencies	1

Frequencies (Top 3 out of 63)

```
1.    -213.8843 cm-1
2.      40.2382 cm-1
3.      55.6738 cm-1
```

M06-2X/def2tzvpp-IEFPCM(water) Molecular Geometry in Cartesian Coordinates

```
C         -1.012455       0.784983      -0.688348
C          0.141159       0.833068       0.093246
C          1.284089       0.133855      -0.391960
O          2.380513       0.222771       0.429214
O          1.375127      -0.505317      -1.442467
C          3.542423      -0.453533      -0.018522
H          3.359253      -1.522452      -0.123718
H          3.880715      -0.062132      -0.977336
H          4.302766      -0.282393       0.738697
H         -0.866865       0.422881      -1.696788
C         -2.082159       1.834211      -0.544938
```

```
C          0.099653       1.455588       1.462663
H          1.038603       1.319481       1.992327
H         -0.111000       2.528263       1.429647
H         -0.694552       0.999441       2.066651
H         -1.714627       2.785129      -0.943663
H         -2.981349       1.560765      -1.095346
H         -2.354117       1.989499       0.497828
S         -2.314171      -1.037552      -0.070450
C         -1.060977      -1.984245       0.811238
H         -1.403237      -2.262737       1.807189
H         -0.772140      -2.887416       0.274615
H         -0.172222      -1.350690       0.922844
```

5_methyltiglate_TS_2

Datum	Value
M06-2X/def2tzvpp-IEFPCM(water) Energy	-823.294927
M06-2X/def2tzvpp-IEFPCM(water) Free Energy (Quasiharmonic)	-823.140417
Number of Imaginary Frequencies	1

Frequencies (Top 3 out of 63)

```
1.    -224.8927 cm-1
2.      47.1365 cm-1
3.      64.7576 cm-1
```

M06-2X/def2tzvpp-IEFPCM(water) Molecular Geometry in Cartesian Coordinates

```
C          0.852983      -0.540859      -0.854271
C         -0.072728      -0.999384       0.085840
C         -1.395842      -0.476267       0.131402
O         -1.654069       0.483105      -0.816272
O         -2.294238      -0.802452       0.911510
C         -2.962129       1.026424      -0.804770
H         -2.993092       1.756240      -1.609362
H         -3.712360       0.255238      -0.976532
H         -3.176701       1.515569       0.145180
H          0.445374       0.025688      -1.678365
C          2.040550      -1.392145      -1.221697
C          0.355747      -1.939539       1.178674
H         -0.456386      -2.096094       1.885557
H          0.664594      -2.918562       0.801549
H          1.210862      -1.531402       1.731081
```

```
 H          1.702151      -2.262224      -1.792952
 H          2.749408      -0.837917      -1.835598
 H          2.563486      -1.753064      -0.337710
 S          2.036636       1.255920      -0.018246
 C          0.872777       1.774454       1.254681
 H          1.375928       1.931265       2.207817
 H          0.342192       2.686880       0.984204
 H          0.136685       0.972328       1.386003
```

5_methyltiglate_TS_3

Datum	Value
M06-2X/def2tzvpp-IEFPCM(water) Energy	-823.289187
M06-2X/def2tzvpp-IEFPCM(water) Free Energy (Quasiharmonic)	-823.134588
Number of Imaginary Frequencies	1

Frequencies (Top 3 out of 63)

```
1.    -256.5346 cm-1
2.      57.8545 cm-1
3.      60.9238 cm-1
```

M06-2X/def2tzvpp-IEFPCM(water) Molecular Geometry in Cartesian Coordinates

```
 C          0.769545       0.510130       0.605734
 C         -0.507087       0.778438       0.112446
 C         -1.491573      -0.237272       0.273517
 O         -2.712636       0.080233      -0.267394
 O         -1.352986      -1.325162       0.836449
 C         -3.730756      -0.891988      -0.107164
 H         -3.457666      -1.830782      -0.587863
 H         -3.928511      -1.085016       0.946743
 H         -4.618801      -0.480910      -0.579634
 H          0.839053      -0.308967       1.311519
 C          1.734107       1.641327       0.838469
 C         -0.762489       2.019185      -0.701498
 H         -0.152610       2.034916      -1.613258
 H         -1.803875       2.088250      -1.003043
 H         -0.516601       2.930436      -0.149579
 H          1.353042       2.277394       1.644648
 H          2.718172       1.280315       1.128931
 H          1.841856       2.261388      -0.049883
 S          2.043862      -0.729369      -0.907179
```

```
C          3.403219      -1.131773       0.225897
H          4.211890      -0.403486       0.167277
H          3.034211      -1.145617       1.255073
H          3.813219      -2.116870       0.007096
```

5_methyltiglate_TS_4_reopt

Datum	Value
M06-2X/def2tzvpp-IEFPCM(water) Energy	-823.29476
M06-2X/def2tzvpp-IEFPCM(water) Free Energy (Quasiharmonic)	-823.140366
Number of Imaginary Frequencies	1

Frequencies (Top 3 out of 63)

```
1.    -213.8492 cm-1
2.      40.2952 cm-1
3.      55.7054 cm-1
```

M06-2X/def2tzvpp-IEFPCM(water) Molecular Geometry in Cartesian Coordinates

```
C         -1.012441       0.784977      -0.688383
C          0.141190       0.833125       0.093171
C          1.284123       0.133849      -0.391984
O          2.380506       0.222722       0.429240
O          1.375184      -0.505328      -1.442481
C          3.542409      -0.453637      -0.018432
H          3.359233      -1.522571      -0.123473
H          3.880678      -0.062373      -0.977308
H          4.302768      -0.282391       0.738748
H         -0.866922       0.422779      -1.696797
C         -2.082193       1.834150      -0.544937
C          0.099770       1.455846       1.462501
H         -0.111975       2.528297       1.429296
H         -0.693660       0.999060       2.067012
H          1.039129       1.320831       1.991715
H         -1.714655       2.785150      -0.943465
H         -2.981306       1.560745      -1.095490
H         -2.354277       1.989251       0.497822
S         -2.314267      -1.037534      -0.070295
C         -1.060980      -1.984340       0.811136
H         -0.772222      -2.887462       0.274383
H         -0.172198      -1.350825       0.922666
H         -1.403102      -2.262935       1.807105
```

5_methyltiglate_TS_5_reopt2

Datum	Value
M06-2X/def2tzvpp-IEFPCM(water) Energy	-823.294927
M06-2X/def2tzvpp-IEFPCM(water) Free Energy (Quasiharmonic)	-823.140419
Number of Imaginary Frequencies	1

Frequencies (Top 3 out of 63)

```
1.    -224.9045 cm-1
2.      47.0836 cm-1
3.      64.7386 cm-1
```

M06-2X/def2tzvpp-IEFPCM(water) Molecular Geometry in Cartesian Coordinates

```
C         -0.852958        0.540871       -0.854256
C          0.072744        0.999371        0.085871
C          1.395857        0.476229        0.131447
O          1.654083       -0.483107       -0.816263
O          2.294246        0.802376        0.911578
C          2.962147       -1.026416       -0.804804
H          2.993118       -1.756160       -1.609461
H          3.712371       -0.255210       -0.976498
H          3.176720       -1.515643        0.145103
H         -0.445336       -0.025675       -1.678345
C         -2.040476        1.392221       -1.221711
C         -0.355735        1.939533        1.178697
H         -0.664780        2.918476        0.801527
H         -1.210721        1.531325        1.731253
H          0.456465        2.096271        1.885461
H         -1.702001        2.262360       -1.792827
H         -2.749281        0.838079       -1.835750
H         -2.563500        1.753046       -0.337737
S         -2.036650       -1.255882       -0.018308
C         -0.872911       -1.774509        1.254687
H         -0.342499       -2.687069        0.984324
H         -0.136663       -0.972515        1.385951
H         -1.376122       -1.931119        2.207825
```

5_methyltiglate_TS_6

Datum	Value
M06-2X/def2tzvpp-IEFPCM(water) Energy	-823.289318
M06-2X/def2tzvpp-IEFPCM(water) Free Energy (Quasiharmonic)	-823.135108
Number of Imaginary Frequencies	1

Frequencies (Top 3 out of 63)

```
1.    -262.7267 cm-1
2.      51.7949 cm-1
3.      66.2136 cm-1
```

M06-2X/def2tzvpp-IEFPCM(water) Molecular Geometry in Cartesian Coordinates

```
C          0.621193        0.473646        0.637072
C         -0.558196        0.989190        0.095764
C         -1.715579        0.175366       -0.052576
O         -1.578824       -1.095414        0.446395
O         -2.790231        0.503532       -0.561399
C         -2.724466       -1.922798        0.350730
H         -2.444065       -2.878486        0.785427
H         -3.564071       -1.499082        0.901273
H         -3.024152       -2.062976       -0.687230
H          0.534383       -0.454687        1.186789
C          1.666977        1.420690        1.164090
C         -0.581733        2.367616       -0.506924
H          0.134940        2.460663       -1.332014
H         -1.570796        2.591260       -0.900394
H         -0.325878        3.145739        0.217286
H          2.575494        0.898431        1.455853
H          1.925658        2.178990        0.427109
H          1.271291        1.934864        2.046499
S          1.907488       -0.608754       -0.969384
C          3.103285       -1.372960        0.161810
H          3.981790       -0.745655        0.311421
H          2.635912       -1.539319        1.136078
H          3.433252       -2.339117       -0.217717
```

7_isobutylacrylate_1

Datum	Value

Datum	Value
M06-2X/def2tzvpp-IEFPCM(water) Energy	-424.398624
M06-2X/def2tzvpp-IEFPCM(water) Free Energy (Quasiharmonic)	-424.24911
Number of Imaginary Frequencies	0

Frequencies (Top 3 out of 57)

```
1.      51.8686 cm-1
2.      60.5986 cm-1
3.      80.0092 cm-1
```

M06-2X/def2tzvpp-IEFPCM(water) Molecular Geometry in Cartesian Coordinates

```
C          1.295189      -0.243411       0.145602
C          2.541934       0.562178       0.157949
C          3.625037       0.137228      -0.474723
O          1.161425      -1.307455      -0.408312
O          0.327920       0.361317       0.838688
C         -0.947850      -0.296367       0.894955
C         -1.790669      -0.013333      -0.338472
C         -2.031974       1.481967      -0.510319
C         -3.106396      -0.774976      -0.216977
H          2.513474       1.496393       0.701258
H          4.539861       0.712780      -0.474729
H          3.614761      -0.804129      -1.009006
H         -0.788610      -1.366051       1.023536
H         -1.429701       0.107280       1.785209
H         -1.244519      -0.389892      -1.206714
H         -2.621989       1.674141      -1.406549
H         -2.581952       1.877830       0.346889
H         -1.094813       2.031095      -0.595840
H         -3.730450      -0.602831      -1.093511
H         -2.939952      -1.848557      -0.122813
H         -3.662493      -0.438663       0.661177
```

7_isobutylacrylate_2

Datum	Value
M06-2X/def2tzvpp-IEFPCM(water) Energy	-424.398624
M06-2X/def2tzvpp-IEFPCM(water) Free Energy (Quasiharmonic)	-424.24911

Datum	Value
Number of Imaginary Frequencies	0

Frequencies (Top 3 out of 57)

```
1.      51.8688 cm-1
2.      60.5986 cm-1
3.      80.0092 cm-1
```

M06-2X/def2tzvpp-IEFPCM(water) Molecular Geometry in Cartesian Coordinates

```
C         -1.295189       -0.243411        0.145602
C         -2.541935        0.562178        0.157948
C         -3.625037        0.137227       -0.474724
O         -1.161425       -1.307455       -0.408310
O         -0.327920        0.361318        0.838687
C          0.947850       -0.296367        0.894955
C          1.790669       -0.013333       -0.338472
C          3.106396       -0.774977       -0.216977
C          2.031976        1.481967       -0.510319
H         -2.513474        1.496394        0.701256
H         -4.539861        0.712779       -0.474730
H         -3.614761       -0.804130       -1.009005
H          1.429700        0.107280        1.785209
H          0.788609       -1.366050        1.023536
H          1.244518       -0.389891       -1.206714
H          3.730449       -0.602833       -1.093512
H          3.662493       -0.438665        0.661176
H          2.939950       -1.848558       -0.122814
H          2.621991        1.674140       -1.406549
H          1.094815        2.031096       -0.595839
H          2.581955        1.877830        0.346888
```

7_isobutylacrylate_3

Datum	Value
M06-2X/def2tzvpp-IEFPCM(water) Energy	-424.398575
M06-2X/def2tzvpp-IEFPCM(water) Free Energy (Quasiharmonic)	-424.249314
Number of Imaginary Frequencies	0

Frequencies (Top 3 out of 57)

```
1.      49.7953 cm-1
2.      60.2993 cm-1
3.     102.4427 cm-1
```

M06-2X/def2tzvpp-IEFPCM(water) Molecular Geometry in Cartesian Coordinates

```
C          -1.375996        0.285378        0.000003
C          -2.300652       -0.875789        0.000003
C          -3.612320       -0.692879       -0.000005
O          -1.713505        1.444705       -0.000001
O          -0.103907       -0.110218        0.000010
C           0.891763        0.925979        0.000010
C           2.261459        0.274208       -0.000002
C           2.484437       -0.556065       -1.260357
C           2.484455       -0.556076        1.260341
H          -1.849590       -1.858355        0.000009
H          -4.297937       -1.528413       -0.000005
H          -4.026841        0.307053       -0.000010
H           0.745190        1.546157       -0.885260
H           0.745201        1.546145        0.885291
H           2.973996        1.103794       -0.000003
H           3.497806       -0.956873       -1.279341
H           1.789667       -1.396264       -1.293059
H           2.337697        0.042756       -2.160134
H           3.497825       -0.956884        1.279308
H           2.337727        0.042736        2.160126
H           1.789687       -1.396277        1.293045
```

7_isobutylacrylate_4

Datum	Value
M06-2X/def2tzvpp-IEFPCM(water) Energy	-424.398068
M06-2X/def2tzvpp-IEFPCM(water) Free Energy (Quasiharmonic)	-424.248614
Number of Imaginary Frequencies	0

Frequencies (Top 3 out of 57)

```
1.      41.7586 cm-1
2.      54.8351 cm-1
3.      90.4747 cm-1
```

M06-2X/def2tzvpp-IEFPCM(water) Molecular Geometry in Cartesian Coordinates

```
C         -1.306599      -0.632626      -0.073016
C         -2.664362      -0.081386      -0.297447
C         -3.059072       1.105835       0.139536
O         -0.954762      -1.703475      -0.509364
O         -0.524805       0.163784       0.655343
C          0.823138      -0.274169       0.887266
C          1.736631       0.046934      -0.284779
C          1.753973       1.543275      -0.575595
C          3.136231      -0.471699       0.028449
H         -3.316835      -0.732974      -0.862559
H         -4.060132       1.465005      -0.055471
H         -2.393427       1.747349       0.700647
H          1.141544       0.268498       1.777139
H          0.817061      -1.342478       1.098817
H          1.352294      -0.483053      -1.159628
H          0.755505       1.918864      -0.797914
H          2.140380       2.092139       0.286647
H          2.397120       1.760371      -1.428552
H          3.536915       0.023195       0.916145
H          3.813575      -0.269651      -0.800871
H          3.132897      -1.546736       0.211280
```

7_isobutylacrylate_5

Datum	Value
M06-2X/def2tzvpp-IEFPCM(water) Energy	-424.398068
M06-2X/def2tzvpp-IEFPCM(water) Free Energy (Quasiharmonic)	-424.248614
Number of Imaginary Frequencies	0

Frequencies (Top 3 out of 57)

```
1.      41.7586 cm-1
2.      54.8352 cm-1
3.      90.4747 cm-1
```

M06-2X/def2tzvpp-IEFPCM(water) Molecular Geometry in Cartesian Coordinates

```
C          1.306598      -0.632626      -0.073016
C          2.664362      -0.081386      -0.297447
C          3.059072       1.105836       0.139536
O          0.954762      -1.703475      -0.509364
O          0.524805       0.163784       0.655344
C         -0.823138      -0.274169       0.887266
C         -1.736631       0.046935      -0.284779
C         -1.753973       1.543276      -0.575594
C         -3.136231      -0.471699       0.028448
H          3.316835      -0.732975      -0.862559
H          4.060132       1.465005      -0.055472
H          2.393427       1.747349       0.700646
H         -0.817061      -1.342479       1.098816
H         -1.141544       0.268496       1.777139
H         -1.352293      -0.483052      -1.159628
H         -2.397119       1.760372      -1.428551
H         -2.140381       2.092139       0.286648
H         -0.755505       1.918865      -0.797912
H         -3.813575      -0.269651      -0.800873
H         -3.132897      -1.546737       0.211278
H         -3.536916       0.023193       0.916145
```

7_isobutylacrylate_6

Datum	Value
M06-2X/def2tzvpp-IEFPCM(water) Energy	-424.398166
M06-2X/def2tzvpp-IEFPCM(water) Free Energy (Quasiharmonic)	-424.248959
Number of Imaginary Frequencies	0

Frequencies (Top 3 out of 57)

```
1.      42.4624 cm-1
2.      59.3915 cm-1
3.     109.4919 cm-1
```

M06-2X/def2tzvpp-IEFPCM(water) Molecular Geometry in Cartesian Coordinates

```
C         -1.513521      -0.564884       0.000002
C         -2.563082       0.482041      -0.000001
C         -2.303148       1.781640      -0.000001
O         -1.762511      -1.747998      -0.000003
O         -0.276823      -0.076277       0.000010
```

```
C          0.796667      -1.030382       0.000011
C          2.106919      -0.265993      -0.000001
C          2.256443       0.581347       1.259935
C          2.256428       0.581333      -1.259949
H         -3.573458       0.096298      -0.000004
H         -3.103462       2.508694      -0.000004
H         -1.286264       2.150780       0.000001
H          0.701634      -1.660151       0.885529
H          0.701625      -1.660164      -0.885497
H          2.888111      -1.031202      -0.000001
H          3.231574       1.067797       1.279969
H          1.491565       1.358737       1.290293
H          2.160014      -0.026839       2.160242
H          3.231559       1.067783      -1.280000
H          2.159988      -0.026864      -2.160247
H          1.491549       1.358722      -1.290307
```

7_isobutylacrylate_7

Datum	Value
M06-2X/def2tzvpp-IEFPCM(water) Energy	-424.398465
M06-2X/def2tzvpp-IEFPCM(water) Free Energy (Quasiharmonic)	-424.249459
Number of Imaginary Frequencies	0

Frequencies (Top 3 out of 57)

```
1.      43.2215 cm-1
2.      63.0639 cm-1
3.     104.6317 cm-1
```

M06-2X/def2tzvpp-IEFPCM(water) Molecular Geometry in Cartesian Coordinates

```
C         -1.551253      -0.632794       0.085951
C         -2.782795       0.184269      -0.027365
C         -2.782851       1.478362      -0.312986
O         -1.564294      -1.807345       0.371865
O         -0.434823       0.050092      -0.155269
C          0.798695      -0.676008      -0.053633
C          1.941009       0.279492      -0.333494
C          3.250931      -0.501936      -0.331603
C          1.974961       1.413740       0.684616
H         -3.696553      -0.366576       0.149298
H         -3.708852       2.032436      -0.381009
```

```
H          -1.860008        2.015602       -0.484956
H           0.878150       -1.096031        0.951863
H           0.780173       -1.499136       -0.768958
H           1.783096        0.700552       -1.330180
H           3.425436       -0.952098        0.648191
H           3.242947       -1.299374       -1.075234
H           4.089429        0.158776       -0.549650
H           2.131205        1.013572        1.689358
H           2.792230        2.100971        0.465575
H           1.043506        1.978571        0.684014
```

7_isobutylacrylate_8

Datum	Value
M06-2X/def2tzvpp-IEFPCM(water) Energy	-424.397552
M06-2X/def2tzvpp-IEFPCM(water) Free Energy (Quasiharmonic)	-424.247897
Number of Imaginary Frequencies	0

Frequencies (Top 3 out of 57)

```
1.      40.8545 cm-1
2.      72.5298 cm-1
3.      85.8777 cm-1
```

M06-2X/def2tzvpp-IEFPCM(water) Molecular Geometry in Cartesian Coordinates

```
C          -1.306779        0.509030       -0.286904
C          -2.657407        0.119773        0.186255
C          -3.024099       -1.131028        0.425502
O          -0.987796        1.660223       -0.467388
O          -0.500955       -0.533961       -0.487770
C           0.852173       -0.291240       -0.903143
C           1.815919       -0.468387        0.258525
C           3.242341       -0.321146       -0.261430
C           1.532290        0.524003        1.380052
H          -3.330406        0.953962        0.330522
H          -4.021428       -1.359857        0.774995
H          -2.339367       -1.955036        0.279364
H           1.051885       -1.022839       -1.684325
H           0.926832        0.712162       -1.320465
H           1.684306       -1.483316        0.643671
H           3.455238       -1.038968       -1.054321
H           3.401286        0.683131       -0.660374
```

```
H           3.960708       -0.479310        0.542385
H           1.645192        1.548581        1.019698
H           2.228430        0.373797        2.205427
H           0.520710        0.411562        1.771551
```

7_isobutylacrylate_HEI_10_reopt

Datum	Value
M06-2X/def2tzvpp-IEFPCM(water) Energy	-862.6132
M06-2X/def2tzvpp-IEFPCM(water) Free Energy (Quasiharmonic)	-862.429663
Number of Imaginary Frequencies	0

Frequencies (Top 3 out of 72)

```
1.      25.9963 cm-1
2.      54.6172 cm-1
3.      69.4906 cm-1
```

M06-2X/def2tzvpp-IEFPCM(water) Molecular Geometry in Cartesian Coordinates

```
C           0.147524        1.439355        0.247521
C           1.528709        1.445859        0.207612
C           2.352113        0.673462       -0.741368
O          -0.605876        2.093992        1.003213
O          -0.439145        0.597656       -0.709969
C          -1.849413        0.552121       -0.802701
C          -2.413313       -0.744402       -0.233912
C          -3.898957       -0.845224       -0.561929
C          -2.176298       -0.838278        1.268186
H           2.026167        2.056839        0.949365
H           1.777157        0.355562       -1.608499
H           3.221551        1.228601       -1.097873
H          -2.286366        1.405560       -0.282440
H          -2.100067        0.622092       -1.864965
H          -1.888465       -1.570706       -0.724155
H          -4.323332       -1.770869       -0.171958
H          -4.073594       -0.818777       -1.638558
H          -4.444813       -0.012597       -0.111233
H          -2.493076       -1.808855        1.653239
H          -1.122947       -0.695986        1.508832
H          -2.743160       -0.062835        1.788447
S           3.154935       -0.877642       -0.071409
C           1.675431       -1.775800        0.419029
```

```
H           1.038375       -1.106022        0.995550
H           1.972973       -2.620671        1.035927
H           1.126034       -2.134819       -0.449722
```

7_isobutylacrylate_HEI_11

Datum	Value
M06-2X/def2tzvpp-IEFPCM(water) Energy	-862.614553
M06-2X/def2tzvpp-IEFPCM(water) Free Energy (Quasiharmonic)	-862.43118
Number of Imaginary Frequencies	0

Frequencies (Top 3 out of 72)

```
1.      29.1820 cm-1
2.      52.0397 cm-1
3.      60.7476 cm-1
```

M06-2X/def2tzvpp-IEFPCM(water) Molecular Geometry in Cartesian Coordinates

```
C           0.029542       -1.159823        0.234005
C          -1.133702       -0.819127        0.889142
C          -2.442309       -0.989080        0.230731
O           0.169516       -1.609451       -0.925610
O           1.182293       -0.998038        1.023263
C           2.391410       -0.808704        0.314328
C           2.503373        0.585049       -0.293949
C           3.800652        0.692527       -1.086908
C           2.422774        1.663234        0.780153
H          -1.075658       -0.387091        1.877416
H          -3.250236       -1.158522        0.943463
H          -2.432204       -1.818177       -0.477022
H           3.192106       -0.952368        1.045220
H           2.493986       -1.563627       -0.466635
H           1.658582        0.706611       -0.978440
H           4.661709        0.544653       -0.429830
H           3.848084       -0.058150       -1.877175
H           3.898960        1.676281       -1.546902
H           1.496550        1.581961        1.348656
H           3.259424        1.566783        1.477533
H           2.468126        2.659604        0.338183
S          -3.032526        0.433516       -0.826133
C          -3.100515        1.705087        0.446890
H          -3.373303        2.646389       -0.024573
```

```
H          -3.843938        1.455623        1.203302
H          -2.123591        1.808709        0.917351
```

7_isobutylacrylate_HEI_12

Datum	Value
M06-2X/def2tzvpp-IEFPCM(water) Energy	-862.61287
M06-2X/def2tzvpp-IEFPCM(water) Free Energy (Quasiharmonic)	-862.429751
Number of Imaginary Frequencies	0

Frequencies (Top 3 out of 72)

```
1.      18.5018 cm-1
2.      40.8544 cm-1
3.      55.4328 cm-1
```

M06-2X/def2tzvpp-IEFPCM(water) Molecular Geometry in Cartesian Coordinates

```
C          -0.072990        1.259361        0.120751
C           0.998807        1.208243       -0.749262
C           1.635280       -0.036315       -1.219255
O          -0.670341        2.269701        0.556543
O          -0.506886       -0.004838        0.548792
C          -1.776988       -0.107127        1.163905
C          -2.838453       -0.598695        0.183024
C          -2.481288       -1.976231       -0.366092
C          -3.047694        0.400698       -0.950099
H           1.424012        2.157217       -1.048772
H           0.965877       -0.889550       -1.126605
H           1.968979        0.023103       -2.256562
H          -1.671510       -0.829014        1.978042
H          -2.062064        0.856525        1.583829
H          -3.769190       -0.681632        0.754147
H          -1.547115       -1.924570       -0.928320
H          -2.351726       -2.702042        0.438713
H          -3.259703       -2.345371       -1.035183
H          -2.124521        0.518636       -1.522106
H          -3.826297        0.056159       -1.632314
H          -3.330037        1.381492       -0.567127
S           3.216348       -0.534959       -0.351083
C           2.599387       -0.668875        1.334781
H           3.452921       -0.767472        2.001797
```

```
H           2.042526        0.234016        1.581746
H           1.947730       -1.533407        1.446843
```

7_isobutylacrylate_HEI_13_reopt

Datum	Value
M06-2X/def2tzvpp-IEFPCM(water) Energy	-862.613682
M06-2X/def2tzvpp-IEFPCM(water) Free Energy (Quasiharmonic)	-862.430789
Number of Imaginary Frequencies	0

Frequencies (Top 3 out of 72)

```
1.      29.5364 cm-1
2.      37.0532 cm-1
3.      55.9963 cm-1
```

M06-2X/def2tzvpp-IEFPCM(water) Molecular Geometry in Cartesian Coordinates

```
C          -0.378130        1.559289       -0.044705
C          -1.662643        1.381771        0.432682
C          -2.147687        0.149328        1.080865
O           0.100236        2.548094       -0.648293
O           0.475392        0.482837        0.214337
C           1.760502        0.538696       -0.368284
C           2.509862       -0.737369       -0.022190
C           2.682994       -0.886515        1.485029
C           3.861165       -0.741012       -0.728801
H          -2.365915        2.179597        0.233622
H          -2.884556        0.341668        1.862035
H          -1.335320       -0.427069        1.519334
H           2.315163        1.406677        0.004159
H           1.677923        0.646457       -1.453900
H           1.916824       -1.579382       -0.392566
H           3.263482       -0.048763        1.880710
H           1.720059       -0.901947        1.993570
H           3.215701       -1.807112        1.727000
H           3.746982       -0.660287       -1.810614
H           4.470233        0.100523       -0.389717
H           4.410349       -1.657641       -0.512466
S          -3.073214       -1.050886       -0.019319
C          -1.788958       -1.380300       -1.236884
H          -2.236281       -1.928759       -2.062850
```

```
H          -1.390322       -0.433041       -1.598984
H          -0.980549       -1.967526       -0.804863
```

7_isobutylacrylate_HEI_14

Datum	Value
M06-2X/def2tzvpp-IEFPCM(water) Energy	-862.61448
M06-2X/def2tzvpp-IEFPCM(water) Free Energy (Quasiharmonic)	-862.431677
Number of Imaginary Frequencies	0

Frequencies (Top 3 out of 72)

```
1.      20.9879 cm-1
2.      44.3110 cm-1
3.      48.9233 cm-1
```

M06-2X/def2tzvpp-IEFPCM(water) Molecular Geometry in Cartesian Coordinates

```
C           0.002278       -0.181689       -0.068750
C          -1.034964        0.611830        0.375917
C          -2.170174        0.022559        1.108068
O           0.174784       -1.408993        0.102341
O           0.957452        0.525206       -0.816858
C           2.209317       -0.106249       -0.998204
C           3.086389       -0.060681        0.247913
C           3.339742        1.375322        0.691384
C           4.396106       -0.790713       -0.026990
H          -1.044096        1.660027        0.114194
H          -2.632216        0.727479        1.800246
H          -1.869850       -0.861389        1.670694
H           2.069286       -1.140617       -1.315327
H           2.701747        0.441312       -1.806918
H           2.546679       -0.586690        1.039693
H           3.947732        1.402867        1.596779
H           3.874511        1.924165       -0.088679
H           2.403885        1.896652        0.891405
H           5.037983       -0.789846        0.854505
H           4.220966       -1.827458       -0.318310
H           4.942494       -0.300901       -0.837293
S          -3.589894       -0.620646        0.074790
C          -4.055033        0.922872       -0.727555
H          -4.411074        1.646825        0.004755
```

```
H          -3.198375        1.335115       -1.258777
H          -4.851221        0.713596       -1.438169
```

7_isobutylacrylate_HEI_15

Datum	Value
M06-2X/def2tzvpp-IEFPCM(water) Energy	-862.614713
M06-2X/def2tzvpp-IEFPCM(water) Free Energy (Quasiharmonic)	-862.430954
Number of Imaginary Frequencies	0

Frequencies (Top 3 out of 72)

```
1.      35.1935 cm-1
2.      52.1688 cm-1
3.      67.4290 cm-1
```

M06-2X/def2tzvpp-IEFPCM(water) Molecular Geometry in Cartesian Coordinates

```
C           0.075673       -0.000996       -0.005666
C           1.057662       -0.746704       -0.624392
C           2.256817       -1.190393        0.108079
O          -0.010610        0.351701        1.190676
O          -0.940751        0.396108       -0.895052
C          -2.144227        0.903938       -0.354625
C          -3.251546       -0.143888       -0.348174
C          -4.560290        0.496081        0.101378
C          -2.884511       -1.327794        0.538702
H           0.976059       -0.940019       -1.685017
H           2.587252       -2.193952       -0.164609
H           2.084799       -1.169781        1.183524
H          -1.972675        1.269712        0.659099
H          -2.443472        1.746947       -0.983007
H          -3.370288       -0.501382       -1.375939
H          -4.466312        0.876740        1.121436
H          -4.840261        1.329783       -0.544463
H          -5.374401       -0.229263        0.090446
H          -1.935462       -1.769744        0.233220
H          -2.784974       -1.004442        1.577224
H          -3.654300       -2.099829        0.494707
S           3.808535       -0.190161       -0.181029
C           3.214138        1.424529        0.349056
H           3.110492        1.468899        1.431831
```

```
H          3.928805       2.176904       0.023279
H          2.246769       1.610882      -0.116398
```

7_isobutylacrylate_HEI_16

Datum	Value
M06-2X/def2tzvpp-IEFPCM(water) Energy	-862.615065
M06-2X/def2tzvpp-IEFPCM(water) Free Energy (Quasiharmonic)	-862.431104
Number of Imaginary Frequencies	0

Frequencies (Top 3 out of 72)

```
1.      32.0581 cm-1
2.      60.7387 cm-1
3.      67.8910 cm-1
```

M06-2X/def2tzvpp-IEFPCM(water) Molecular Geometry in Cartesian Coordinates

```
C         -0.084204      -1.027471       0.207853
C         -1.214661      -1.178678      -0.567483
C         -2.563324      -1.051930       0.013773
O          0.004731      -0.760433       1.426363
O          1.101474      -1.251773      -0.517705
C          2.333628      -0.913999       0.086682
C          2.907820       0.375521      -0.488559
C          4.289574       0.630598       0.102642
C          1.970654       1.549728      -0.234505
H         -1.102641      -1.385229      -1.622669
H         -2.528138      -1.153285       1.098140
H         -3.273687      -1.784330      -0.373574
H          2.203154      -0.813539       1.165223
H          3.024425      -1.739775      -0.104725
H          3.008168       0.237749      -1.569815
H          4.221328       0.760721       1.185525
H          4.967939      -0.201154      -0.094016
H          4.733089       1.535247      -0.314347
H          0.974186       1.343533      -0.627479
H          1.877683       1.735131       0.838175
H          2.346179       2.459422      -0.705342
S         -3.457150       0.553335      -0.320710
C         -2.282470       1.694802       0.426835
H         -1.279112       1.416436       0.105245
```

```
H          -2.512302        2.701298        0.084743
H          -2.337614        1.660636        1.513582
```

7_isobutylacrylate_HEI_17_reopt

Datum	Value
M06-2X/def2tzvpp-IEFPCM(water) Energy	-862.612374
M06-2X/def2tzvpp-IEFPCM(water) Free Energy (Quasiharmonic)	-862.428421
Number of Imaginary Frequencies	0

Frequencies (Top 3 out of 72)

```
1.      30.9906 cm-1
2.      44.0641 cm-1
3.      65.9065 cm-1
```

M06-2X/def2tzvpp-IEFPCM(water) Molecular Geometry in Cartesian Coordinates

```
C          -0.126524        1.197632       -0.088496
C           0.992870        0.961557        0.686659
C           1.548543       -0.379298        0.949808
O          -0.675518        2.290264       -0.350764
O          -0.671208        0.028509       -0.639715
C          -1.980169        0.088684       -1.175987
C          -3.014411       -0.450691       -0.191151
C          -3.062228        0.397997        1.075420
C          -2.743446       -1.913844        0.144134
H           1.504302        1.830323        1.077795
H           2.060566       -0.427240        1.911931
H           0.782979       -1.153942        0.933817
H          -2.216018        1.115173       -1.453290
H          -1.976702       -0.529257       -2.077422
H          -3.983644       -0.383169       -0.696348
H          -2.095110        0.368842        1.582888
H          -3.286734        1.439869        0.846662
H          -3.818531        0.022058        1.766004
H          -1.773346       -2.013537        0.634332
H          -3.504979       -2.308734        0.817924
H          -2.732199       -2.530959       -0.755971
S           2.806765       -1.031107       -0.273137
C           4.050018        0.258294       -0.087238
H           4.462693        0.255161        0.921190
```

```
H          4.849361        0.070328       -0.800261
H          3.605015        1.230617       -0.294109
```

7_isobutylacrylate_HEI_18_reopt

Datum	Value
M06-2X/def2tzvpp-IEFPCM(water) Energy	-862.613371
M06-2X/def2tzvpp-IEFPCM(water) Free Energy (Quasiharmonic)	-862.431212
Number of Imaginary Frequencies	0

Frequencies (Top 3 out of 72)

```
1.      26.5271 cm-1
2.      37.4094 cm-1
3.      49.5275 cm-1
```

M06-2X/def2tzvpp-IEFPCM(water) Molecular Geometry in Cartesian Coordinates

```
C         -0.042014        1.755394       -0.030793
C          1.312404        1.729163        0.241181
C          2.028723        0.538860        0.749321
O         -0.732007        2.697615       -0.483364
O         -0.694120        0.554901        0.262848
C         -2.082408        0.504925        0.007529
C         -2.591422       -0.884174        0.355131
C         -1.907953       -1.951604       -0.492203
C         -4.104963       -0.931029        0.175113
H          1.877265        2.619197        0.000096
H          2.892553        0.803595        1.361400
H          1.379757       -0.112857        1.333051
H         -2.605687        1.261021        0.599255
H         -2.283903        0.720113       -1.047447
H         -2.357180       -1.068598        1.408048
H         -2.122847       -1.784259       -1.551072
H         -0.826932       -1.929629       -0.359107
H         -2.268724       -2.947120       -0.230228
H         -4.370502       -0.735867       -0.866753
H         -4.497684       -1.912896        0.440252
H         -4.604842       -0.186120        0.795697
S          2.705434       -0.517387       -0.617097
C          3.524856       -1.803746        0.350619
H          2.804195       -2.332262        0.973210
```

```
H          3.983587       -2.510791       -0.337063
H          4.299671       -1.372182        0.982960
```

7_isobutylacrylate_HEI_19

Datum	Value
M06-2X/def2tzvpp-IEFPCM(water) Energy	-862.614281
M06-2X/def2tzvpp-IEFPCM(water) Free Energy (Quasiharmonic)	-862.432315
Number of Imaginary Frequencies	0

Frequencies (Top 3 out of 72)

```
1.      26.9915 cm-1
2.      30.8994 cm-1
3.      45.5740 cm-1
```

M06-2X/def2tzvpp-IEFPCM(water) Molecular Geometry in Cartesian Coordinates

```
C         -0.053914       -0.493658        0.284177
C         -1.062362        0.136884        0.984291
C         -2.468298       -0.290289        0.830351
O         -0.133748       -1.461294       -0.505943
O          1.212644        0.048268        0.531085
C          2.300096       -0.559891       -0.135140
C          3.575820        0.181862        0.227712
C          3.520433        1.634649       -0.231126
C          4.772960       -0.536863       -0.385463
H         -0.823309        0.989457        1.603472
H         -3.058088       -0.146741        1.737216
H         -2.533149       -1.338183        0.535799
H          2.379641       -1.612743        0.150278
H          2.151443       -0.527053       -1.219969
H          3.673619        0.160308        1.317336
H          3.422794        1.680094       -1.319111
H          2.670580        2.155732        0.207167
H          4.431720        2.166238        0.045944
H          4.838873       -1.569683       -0.040882
H          4.692271       -0.548898       -1.475188
H          5.703882       -0.032008       -0.126371
S         -3.355316        0.663503       -0.488711
C         -4.998345       -0.076642       -0.366511
H         -5.414378        0.077271        0.628330
```

```
H          -5.645691       0.402229       -1.097837
H          -4.954664      -1.144175       -0.577693
```

7_isobutylacrylate_HEI_1

Datum	Value
M06-2X/def2tzvpp-IEFPCM(water) Energy	-862.613852
M06-2X/def2tzvpp-IEFPCM(water) Free Energy (Quasiharmonic)	-862.430516
Number of Imaginary Frequencies	0

Frequencies (Top 3 out of 72)

```
1.      32.3512 cm-1
2.      53.3114 cm-1
3.      61.4203 cm-1
```

M06-2X/def2tzvpp-IEFPCM(water) Molecular Geometry in Cartesian Coordinates

```
C          -0.375813       1.635447        0.038375
C          -1.677258       1.436915       -0.379282
C          -2.150981       0.221177       -1.065535
O           0.094043       2.585630        0.707059
O           0.510190       0.637065       -0.385085
C           1.785324       0.622491        0.220850
C           2.642396      -0.436084       -0.453155
C           2.005108      -1.817195       -0.356633
C           4.034309      -0.432285        0.169963
H          -2.401882       2.179997       -0.072721
H          -2.941980       0.418426       -1.790511
H          -1.343903      -0.297640       -1.578413
H           2.259492       1.602129        0.139025
H           1.689526       0.391357        1.290448
H           2.730047      -0.164458       -1.509710
H           1.872959      -2.098100        0.692060
H           1.027250      -1.833543       -0.836197
H           2.635864      -2.571122       -0.829705
H           3.977919      -0.697900        1.228424
H           4.682255      -1.158419       -0.321362
H           4.504155       0.549210        0.093357
S          -2.961470      -1.064491        0.030382
C          -1.613141      -1.364749        1.184822
H          -1.237771      -0.408724        1.549957
```

```
H          -2.003236       -1.945182        2.018002
H          -0.800702       -1.910025        0.709006
```

7_isobutylacrylate_HEI_20

Datum	Value
M06-2X/def2tzvpp-IEFPCM(water) Energy	-862.614093
M06-2X/def2tzvpp-IEFPCM(water) Free Energy (Quasiharmonic)	-862.430949
Number of Imaginary Frequencies	0

Frequencies (Top 3 out of 72)

```
1.      27.7662 cm-1
2.      34.9707 cm-1
3.      59.6233 cm-1
```

M06-2X/def2tzvpp-IEFPCM(water) Molecular Geometry in Cartesian Coordinates

```
C          -0.002991        0.028437       -0.147228
C           1.055326        0.068364        0.737893
C           2.203731        0.961234        0.502691
O          -0.180761        0.707811       -1.180541
O          -0.972853       -0.926601        0.204581
C          -2.222312       -0.887117       -0.456059
C          -3.295792       -0.221145        0.397162
C          -4.646274       -0.330315       -0.301967
C          -2.943328        1.232242        0.690700
H           1.066866       -0.616865        1.572979
H           2.686320        1.272403        1.429914
H           1.914375        1.856275       -0.047951
H          -2.124065       -0.361297       -1.407056
H          -2.507167       -1.922874       -0.658406
H          -3.345912       -0.766122        1.345183
H          -4.913998       -1.370382       -0.494703
H          -5.436618        0.116787        0.301722
H          -4.621137        0.194279       -1.260307
H          -3.682223        1.684861        1.353792
H          -1.962945        1.311588        1.161772
H          -2.918417        1.809150       -0.236240
S           3.597134        0.280764       -0.544962
C           4.038789       -1.131798        0.480507
H           4.414561       -0.804068        1.449311
```

```
H          3.167183       -1.768456       0.625393
H          4.815046       -1.696595      -0.030539
```

7_isobutylacrylate_HEI_21

Datum	Value
M06-2X/def2tzvpp-IEFPCM(water) Energy	-862.613502
M06-2X/def2tzvpp-IEFPCM(water) Free Energy (Quasiharmonic)	-862.430037
Number of Imaginary Frequencies	0

Frequencies (Top 3 out of 72)

```
1.      34.9832 cm-1
2.      41.3798 cm-1
3.      52.1070 cm-1
```

M06-2X/def2tzvpp-IEFPCM(water) Molecular Geometry in Cartesian Coordinates

```
C          0.084050       -0.244806      -0.273986
C         -0.871625        0.274631       0.575993
C         -2.051282       -0.522384       0.956235
O          0.134514       -1.383649      -0.785228
O          1.101515        0.675202      -0.579464
C          2.292410        0.181854      -1.164576
C          3.391445       -0.030968      -0.126238
C          2.995721       -1.101372       0.885766
C          3.747570        1.275317       0.576210
H         -0.778640        1.298033       0.909268
H         -2.434608       -0.256870       1.942175
H         -1.833425       -1.590385       0.951418
H          2.084538       -0.748386      -1.692010
H          2.616547        0.933115      -1.889211
H          4.270502       -0.378964      -0.679040
H          2.103200       -0.789431       1.432942
H          2.769881       -2.047293       0.393931
H          3.796203       -1.266103       1.608615
H          4.047823        2.041978      -0.140205
H          2.886646        1.650237       1.131821
H          4.566903        1.129071       1.281347
S         -3.540124       -0.411348      -0.170938
C         -3.852133        1.355977      -0.026483
H         -4.129747        1.617522       0.994145
```

```
H          -2.960326        1.911002       -0.314179
H          -4.668680        1.616120       -0.695996
```

7_isobutylacrylate_HEI_22

Datum	Value
M06-2X/def2tzvpp-IEFPCM(water) Energy	-862.61469
M06-2X/def2tzvpp-IEFPCM(water) Free Energy (Quasiharmonic)	-862.430663
Number of Imaginary Frequencies	0

Frequencies (Top 3 out of 72)

```
1.      36.7471 cm-1
2.      44.5457 cm-1
3.      79.7286 cm-1
```

M06-2X/def2tzvpp-IEFPCM(water) Molecular Geometry in Cartesian Coordinates

```
C          -0.253357        0.933039       -0.125883
C          -0.889002       -0.081648       -0.812188
C          -2.309159        0.056872       -1.198392
O          -0.727635        2.039704        0.227626
O           1.087455        0.782238        0.238491
C           1.774785       -0.408058       -0.075890
C           3.205770       -0.305253        0.426057
C           3.946637        0.843666       -0.248006
C           3.923427       -1.629988        0.190429
H          -0.388244       -1.008101       -1.042723
H          -2.525578       -0.347319       -2.188696
H          -2.616334        1.101460       -1.181774
H           1.276205       -1.266602        0.387179
H           1.779208       -0.570551       -1.161450
H           3.163571       -0.114378        1.502521
H           4.966048        0.924786        0.131220
H           4.001931        0.672976       -1.326413
H           3.439765        1.792652       -0.080538
H           4.947336       -1.587659        0.562199
H           3.414345       -2.455457        0.689616
H           3.965766       -1.855473       -0.877934
S          -3.529040       -0.861907       -0.135128
C          -3.162420       -0.105615        1.457074
H          -3.641361       -0.698443        2.233093
```

```
H          -2.083388       -0.107685        1.606182
H          -3.533278        0.916676        1.501430
```

7_isobutylacrylate_HEI_2_reopt3

Datum	Value
M06-2X/def2tzvpp-IEFPCM(water) Energy	-862.613682
M06-2X/def2tzvpp-IEFPCM(water) Free Energy (Quasiharmonic)	-862.430792
Number of Imaginary Frequencies	0

Frequencies (Top 3 out of 72)

```
1.      29.5556 cm-1
2.      36.9103 cm-1
3.      55.9776 cm-1
```

M06-2X/def2tzvpp-IEFPCM(water) Molecular Geometry in Cartesian Coordinates

```
C           0.378187        1.559293       -0.044908
C           1.662711        1.381832        0.432465
C           2.147765        0.149485        1.080831
O          -0.100150        2.547957       -0.648751
O          -0.475397        0.482975        0.214500
C          -1.760335        0.538498       -0.368535
C          -2.509798       -0.737367       -0.021929
C          -3.860867       -0.741445       -0.728985
C          -2.683428       -0.885603        1.485322
H           2.366006        2.179585        0.233198
H           2.884689        0.341935        1.861920
H           1.335416       -0.426828        1.519444
H          -2.315109        1.406693        0.003231
H          -1.677420        0.645623       -1.454191
H          -1.916631       -1.579597       -0.391603
H          -4.410118       -1.657945       -0.512274
H          -4.470051        0.100293       -0.390615
H          -3.746327       -0.661379       -1.810810
H          -1.720663       -0.900703        1.994193
H          -3.264067       -0.047624        1.880301
H          -3.216194       -1.806065        1.727676
S           3.073174       -1.050939       -0.019227
C           1.788722       -1.380661       -1.236501
H           1.390001       -0.433495       -1.598751
```

```
H          2.235918       -1.929298       -2.062419
H          0.980402       -1.967810       -0.804209
```

7_isobutylacrylate_HEI_3

Datum	Value
M06-2X/def2tzvpp-IEFPCM(water) Energy	-862.614797
M06-2X/def2tzvpp-IEFPCM(water) Free Energy (Quasiharmonic)	-862.432348
Number of Imaginary Frequencies	0

Frequencies (Top 3 out of 72)

```
1.      20.1296 cm-1
2.      32.8254 cm-1
3.      71.1114 cm-1
```

M06-2X/def2tzvpp-IEFPCM(water) Molecular Geometry in Cartesian Coordinates

```
C         -0.235451       -0.719991       -0.027162
C         -1.252910       -0.605821       -0.953326
C         -2.640944       -0.953545       -0.604953
O         -0.281476       -1.145500        1.149301
O          1.005991       -0.299471       -0.522693
C          2.119349       -0.474245        0.328948
C          3.352365        0.097671       -0.351045
C          4.584896       -0.200615        0.495964
C          3.205897        1.596295       -0.589151
H         -1.031247       -0.189926       -1.926448
H         -2.668824       -1.594856        0.275391
H         -3.182452       -1.453708       -1.409545
H          1.955596        0.035068        1.284279
H          2.266801       -1.536314        0.547211
H          3.460938       -0.403428       -1.317706
H          4.494814        0.270183        1.477998
H          4.716555       -1.272864        0.646795
H          5.485966        0.189042        0.021724
H          2.328069        1.816294       -1.194843
H          3.099721        2.118383        0.365555
H          4.084269        1.998959       -1.095201
S         -3.775434        0.483809       -0.224052
C         -2.861745        1.215485        1.143645
H         -2.937445        0.601267        2.039195
```

```
H          -1.815126        1.308299        0.855412
H          -3.275551        2.201031        1.344630
```

7_isobutylacrylate_HEI_4

Datum	Value
M06-2X/def2tzvpp-IEFPCM(water) Energy	-862.613222
M06-2X/def2tzvpp-IEFPCM(water) Free Energy (Quasiharmonic)	-862.430036
Number of Imaginary Frequencies	0

Frequencies (Top 3 out of 72)

```
1.      42.5294 cm-1
2.      44.3282 cm-1
3.      68.6162 cm-1
```

M06-2X/def2tzvpp-IEFPCM(water) Molecular Geometry in Cartesian Coordinates

```
C          -0.177773        1.557013       -0.015660
C          -1.477640        1.356075        0.411025
C          -1.936872        0.143422        1.113423
O           0.301212        2.533436       -0.635890
O           0.686063        0.512071        0.320795
C           2.028204        0.633745       -0.103732
C           2.783884       -0.620631        0.302072
C           4.255160       -0.470211       -0.069521
C           2.181336       -1.862222       -0.345412
H          -2.198155        2.116912        0.145015
H          -1.156892       -0.305844        1.726386
H          -2.796414        0.343887        1.754333
H           2.072614        0.758359       -1.191064
H           2.491332        1.517044        0.344528
H           2.704480       -0.718077        1.389127
H           4.824316       -1.349341        0.233416
H           4.364857       -0.358219       -1.150963
H           4.700892        0.404036        0.406881
H           2.717626       -2.761260       -0.038916
H           1.131954       -1.976246       -0.076563
H           2.246535       -1.786656       -1.434187
S          -2.482971       -1.298706        0.048308
C          -3.821978       -0.485582       -0.839102
H          -4.218960       -1.181826       -1.574174
```

```
H          -4.618927       -0.195386       -0.155102
H          -3.441850        0.398192       -1.349455
```

7_isobutylacrylate_HEI_5_reopt2

Datum	Value
M06-2X/def2tzvpp-IEFPCM(water) Energy	-862.613408
M06-2X/def2tzvpp-IEFPCM(water) Free Energy (Quasiharmonic)	-862.430009
Number of Imaginary Frequencies	0

Frequencies (Top 3 out of 72)

```
1.      28.0450 cm-1
2.      50.4420 cm-1
3.      69.7126 cm-1
```

M06-2X/def2tzvpp-IEFPCM(water) Molecular Geometry in Cartesian Coordinates

```
C          -0.101867       -1.427328       -0.221692
C          -1.481012       -1.499949       -0.197299
C          -2.356324       -0.732442        0.707910
O           0.692190       -2.070167       -0.947781
O           0.442192       -0.530227        0.710210
C           1.853420       -0.492875        0.806424
C           2.503009        0.436285       -0.212303
C           4.018978        0.296311       -0.132071
C           2.075248        1.881554        0.010683
H          -1.937167       -2.153975       -0.929106
H          -3.200203       -1.315824        1.080502
H          -1.810365       -0.345099        1.565986
H           2.074302       -0.130224        1.815097
H           2.261749       -1.498205        0.701277
H           2.165540        0.109913       -1.198836
H           4.373699        0.558854        0.868268
H           4.336569       -0.725732       -0.343753
H           4.512075        0.959918       -0.843177
H           2.493165        2.537722       -0.754200
H           0.990336        1.975984       -0.011654
H           2.427493        2.234214        0.984020
S          -3.227440        0.740951       -0.045119
C          -1.784711        1.710279       -0.508799
H          -1.070656        1.057770       -1.012289
```

```
H          -2.099505        2.501506       -1.185441
H          -1.313499        2.150107        0.368653
```

7_isobutylacrylate_HEI_6_reopt2

Datum	Value
M06-2X/def2tzvpp-IEFPCM(water) Energy	-862.613915
M06-2X/def2tzvpp-IEFPCM(water) Free Energy (Quasiharmonic)	-862.430834
Number of Imaginary Frequencies	0

Frequencies (Top 3 out of 72)

```
1.      20.9442 cm-1
2.      35.3471 cm-1
3.      38.5519 cm-1
```

M06-2X/def2tzvpp-IEFPCM(water) Molecular Geometry in Cartesian Coordinates

```
C           0.028026        1.114386       -0.396749
C           1.128379        0.749184       -1.144975
C           1.816427       -0.552243       -1.045283
O          -0.613405        2.191385       -0.427998
O          -0.392308        0.131118        0.512877
C          -1.741088        0.200161        0.930959
C          -2.716741       -0.254975       -0.148288
C          -4.142521       -0.162997        0.382796
C          -2.392094       -1.667719       -0.618621
H           1.545326        1.508710       -1.793882
H           1.168428       -1.316653       -0.620871
H           2.181387       -0.912762       -2.008535
H          -1.816791       -0.469045        1.792809
H          -1.988042        1.212407        1.254138
H          -2.607961        0.431866       -0.992302
H          -4.272355       -0.826549        1.241739
H          -4.384634        0.851782        0.702216
H          -4.864369       -0.460024       -0.378617
H          -2.491092       -2.375300        0.209230
H          -3.072240       -1.982017       -1.411550
H          -1.371134       -1.728602       -0.995632
S           3.380413       -0.580288       -0.022267
C           2.714961       -0.016400        1.552310
H           2.082190       -0.776748        2.006140
```

```
H           3.549792        0.204909        2.213517
H           2.128492        0.886217        1.385946
```

7_isobutylacrylate_HEI_7_reopt

Datum	Value
M06-2X/def2tzvpp-IEFPCM(water) Energy	-862.614223
M06-2X/def2tzvpp-IEFPCM(water) Free Energy (Quasiharmonic)	-862.431989
Number of Imaginary Frequencies	0

Frequencies (Top 3 out of 72)

```
1.      19.3613 cm-1
2.      35.7226 cm-1
3.      55.2159 cm-1
```

M06-2X/def2tzvpp-IEFPCM(water) Molecular Geometry in Cartesian Coordinates

```
C           0.146903       -0.722046        0.244336
C           1.222287       -0.206833        0.940480
C           2.573311       -0.762627        0.752783
O           0.127803       -1.667830       -0.574547
O          -1.060687       -0.076846        0.531680
C          -2.210070       -0.556313       -0.135540
C          -3.401298        0.298428        0.264797
C          -4.670464       -0.276328       -0.355229
C          -3.203938        1.752234       -0.149477
H           1.072237        0.646415        1.585723
H           2.544434       -1.821554        0.495264
H           3.200063       -0.644088        1.637370
H          -2.069836       -0.509428       -1.220861
H          -2.391601       -1.603328        0.123857
H          -3.490667        0.252477        1.354410
H          -4.602398       -0.259631       -1.445741
H          -4.836001       -1.308414       -0.043494
H          -5.543517        0.309820       -0.067449
H          -2.302782        2.171374        0.295566
H          -3.110739        1.822902       -1.236480
H          -4.055088        2.363010        0.154294
S           3.593194       -0.041960       -0.642612
C           3.642884        1.666743       -0.077730
H           4.190766        1.748925        0.860494
```

```
H          4.142054        2.265518       -0.835992
H          2.627353        2.035232        0.061252
```

7_isobutylacrylate_HEI_8

Datum	Value
M06-2X/def2tzvpp-IEFPCM(water) Energy	-862.61516
M06-2X/def2tzvpp-IEFPCM(water) Free Energy (Quasiharmonic)	-862.431855
Number of Imaginary Frequencies	0

Frequencies (Top 3 out of 72)

```
1.      30.5233 cm-1
2.      41.1038 cm-1
3.      54.6499 cm-1
```

M06-2X/def2tzvpp-IEFPCM(water) Molecular Geometry in Cartesian Coordinates

```
C         -0.068893        0.078219       -0.002981
C         -1.013994       -0.913488       -0.160193
C         -2.196424       -0.988943        0.717043
O          0.005183        0.969962        0.872938
O          0.924659        0.048148       -0.998423
C          2.136332        0.709380       -0.693691
C          3.006391       -0.068191        0.287384
C          4.276161        0.722833        0.579438
C          3.332645       -1.456873       -0.248794
H         -0.921316       -1.594796       -0.994778
H         -2.031925       -0.425812        1.635088
H         -2.473824       -2.008944        0.988129
H          2.665716        0.810924       -1.645458
H          1.935049        1.706942       -0.300379
H          2.432928       -0.172591        1.212432
H          4.856082        0.863641       -0.336470
H          4.047092        1.708571        0.987156
H          4.908984        0.198127        1.295871
H          2.422725       -2.018102       -0.459658
H          3.907470       -1.380280       -1.175819
H          3.927935       -2.023228        0.468883
S         -3.791379       -0.342230       -0.005928
C         -3.287428        1.350332       -0.356075
H         -2.330283        1.331242       -0.876056
```

```
  H         -4.041035        1.806551       -0.993931
  H         -3.191020        1.928935        0.560933
```

7_isobutylacrylate_HEI_9_reopt

Datum	Value
M06-2X/def2tzvpp-IEFPCM(water) Energy	-862.612336
M06-2X/def2tzvpp-IEFPCM(water) Free Energy (Quasiharmonic)	-862.428848
Number of Imaginary Frequencies	0

Frequencies (Top 3 out of 72)

```
  1.      30.1691 cm-1
  2.      60.5368 cm-1
  3.      68.9161 cm-1
```

M06-2X/def2tzvpp-IEFPCM(water) Molecular Geometry in Cartesian Coordinates

```
  C         -0.096524        1.589997        0.125913
  C         -1.461244        1.505459       -0.071194
  C         -2.129111        0.497012       -0.914037
  O          0.528938        2.407000        0.840432
  O          0.637712        0.633014       -0.591559
  C          2.047667        0.751133       -0.634885
  C          2.714809       -0.523698       -0.131034
  C          2.333017       -1.726553       -0.987266
  C          2.382323       -0.766480        1.336767
  H         -2.071471        2.190058        0.503579
  H         -2.975709        0.897358       -1.474640
  H         -1.437970        0.044402       -1.621582
  H          2.338049        0.929101       -1.675957
  H          2.360281        1.602834       -0.033712
  H          3.793265       -0.359612       -0.224507
  H          1.258128       -1.903043       -0.927635
  H          2.591260       -1.563590       -2.035074
  H          2.843885       -2.628293       -0.646960
  H          1.304631       -0.889904        1.457589
  H          2.871213       -1.669251        1.705511
  H          2.698192        0.072689        1.958035
  S         -2.934634       -0.925925       -0.001786
  C         -1.505811       -1.537104        0.903821
  H         -1.011288       -0.692754        1.383645
```

```
H          -1.851554       -2.238331        1.660022
H          -0.800734       -2.035577        0.240767
```

7_isobutylacrylate_TS_10_reopt

Datum	Value
M06-2X/def2tzvpp-IEFPCM(water) Energy	-862.605245
M06-2X/def2tzvpp-IEFPCM(water) Free Energy (Quasiharmonic)	-862.421887
Number of Imaginary Frequencies	1

Frequencies (Top 3 out of 72)

```
1.    -202.7255 cm-1
2.      26.2208 cm-1
3.      54.0200 cm-1
```

M06-2X/def2tzvpp-IEFPCM(water) Molecular Geometry in Cartesian Coordinates

```
C           0.033203        1.540348        0.316018
C           1.451327        1.607303        0.147996
C           2.125017        1.043917       -0.918284
O          -0.596954        1.981687        1.271855
O          -0.604136        0.933535       -0.728099
C          -1.999987        0.674727       -0.623982
C          -2.257165       -0.801284       -0.356516
C          -3.758836       -1.063789       -0.375483
C          -1.633914       -1.237445        0.963758
H           1.988417        2.055185        0.972241
H           1.577762        0.801986       -1.815814
H           3.161577        1.304986       -1.069329
H          -2.431384        1.286419        0.168211
H          -2.440563        0.967794       -1.577985
H          -1.788926       -1.367363       -1.167410
H          -3.968996       -2.121643       -0.217684
H          -4.204819       -0.766950       -1.325746
H          -4.253465       -0.502132        0.420481
H          -1.781352       -2.305955        1.125936
H          -0.561836       -1.036678        0.980592
H          -2.092655       -0.699090        1.796257
S           2.650648       -1.244182       -0.581909
C           2.279057       -1.292544        1.180910
H           1.735137       -0.375186        1.443717
```

```
H           3.181406       -1.340839        1.789614
H           1.645824       -2.142783        1.430910
```

7_isobutylacrylate_TS_11_reopt

Datum	Value
M06-2X/def2tzvpp-IEFPCM(water) Energy	-862.606932
M06-2X/def2tzvpp-IEFPCM(water) Free Energy (Quasiharmonic)	-862.423946
Number of Imaginary Frequencies	1

Frequencies (Top 3 out of 72)

```
1.    -194.6938 cm-1
2.      31.8772 cm-1
3.      50.7309 cm-1
```

M06-2X/def2tzvpp-IEFPCM(water) Molecular Geometry in Cartesian Coordinates

```
C          -0.120667       -1.212803       -0.021102
C          -1.319538       -1.302908        0.753549
C          -2.548038       -1.297520        0.132351
O          -0.036271       -1.159185       -1.242472
O           1.001579       -1.196946        0.759375
C           2.240491       -0.951323        0.101503
C           2.455815        0.527954       -0.189177
C           3.785432        0.707662       -0.913077
C           2.408860        1.351945        1.092336
H          -1.225413       -1.260861        1.829229
H          -3.434054       -1.534043        0.701626
H          -2.591175       -1.533852       -0.920052
H           3.008098       -1.309399        0.790204
H           2.291728       -1.532822       -0.818862
H           1.646426        0.851825       -0.848654
H           4.611061        0.363951       -0.284964
H           3.812134        0.142109       -1.845407
H           3.960168        1.757764       -1.147580
H           1.459833        1.220879        1.611957
H           3.212257        1.048318        1.768580
H           2.535585        2.413090        0.874912
S          -3.331994        0.928632       -0.300655
C          -1.791691        1.770930        0.104221
H          -1.312708        2.194946       -0.778294
```

```
H          -1.946940        2.566189        0.832205
H          -1.101537        1.039228        0.546738
```

7_isobutylacrylate_TS_12_reopt

Datum	Value
M06-2X/def2tzvpp-IEFPCM(water) Energy	-862.604452
M06-2X/def2tzvpp-IEFPCM(water) Free Energy (Quasiharmonic)	-862.421364
Number of Imaginary Frequencies	1

Frequencies (Top 3 out of 72)

```
1.    -194.4538 cm-1
2.      29.1393 cm-1
3.      40.2873 cm-1
```

M06-2X/def2tzvpp-IEFPCM(water) Molecular Geometry in Cartesian Coordinates

```
C           0.103576        1.176761        0.078485
C          -0.957203        1.091413        1.035574
C          -1.476220       -0.099163        1.496766
O           0.623621        2.212907       -0.321636
O           0.513586       -0.041493       -0.378256
C           1.679899       -0.106896       -1.196016
C           2.888029       -0.559421       -0.384636
C           2.671304       -1.953529        0.195386
C           3.224571        0.443569        0.714139
H          -1.382731        2.039345        1.334812
H          -0.910280       -1.006445        1.354242
H          -2.121977       -0.086721        2.361885
H           1.457671       -0.834516       -1.977522
H           1.859990        0.864506       -1.652008
H           3.723946       -0.600074       -1.089838
H           1.833305       -1.945804        0.894552
H           2.451862       -2.679579       -0.588876
H           3.557110       -2.291100        0.734260
H           2.400386        0.515593        1.427814
H           4.113552        0.129267        1.261964
H           3.401475        1.437942        0.304512
S          -3.257637       -0.930305        0.126523
C          -2.933763        0.143067       -1.284565
H          -3.840709        0.640883       -1.625007
```

```
H          -2.219704       0.916879       -0.973638
H          -2.500541      -0.401400       -2.123176
```

7_isobutylacrylate_TS_14_reopt

Datum	Value
M06-2X/def2tzvpp-IEFPCM(water) Energy	-862.606259
M06-2X/def2tzvpp-IEFPCM(water) Free Energy (Quasiharmonic)	-862.423554
Number of Imaginary Frequencies	1

Frequencies (Top 3 out of 72)

```
1.    -188.5405 cm-1
2.      34.3549 cm-1
3.      39.3019 cm-1
```

M06-2X/def2tzvpp-IEFPCM(water) Molecular Geometry in Cartesian Coordinates

```
C          -0.066561       0.242282        0.084560
C          -1.088791       1.141976       -0.355071
C          -2.151795       1.441750        0.465808
O          -0.005593      -0.329540        1.166470
O           0.901249       0.056964       -0.861902
C           2.009978      -0.763168       -0.506167
C           3.068189      -0.003870        0.282082
C           3.588032       1.192561       -0.506467
C           4.200635      -0.958450        0.644190
H          -1.035413       1.502590       -1.372278
H          -2.810121       2.256743        0.207075
H          -2.061812       1.229537        1.520355
H           1.660584      -1.627403        0.058442
H           2.430522      -1.105076       -1.453972
H           2.597796       0.352613        1.201712
H           4.327754       1.746113        0.072817
H           4.066354       0.857976       -1.430666
H           2.780618       1.874877       -0.770612
H           4.969924      -0.445478        1.221471
H           3.838951      -1.802056        1.233486
H           4.669887      -1.353718       -0.260192
S          -3.945290      -0.147106        0.261307
C          -3.050494      -1.332443       -0.759217
H          -3.596985      -1.573064       -1.670175
```

```
H          -2.090370       -0.884649       -1.049554
H          -2.843452       -2.258539       -0.223659
```

7_isobutylacrylate_TS_15

Datum	Value
M06-2X/def2tzvpp-IEFPCM(water) Energy	-862.60588
M06-2X/def2tzvpp-IEFPCM(water) Free Energy (Quasiharmonic)	-862.422716
Number of Imaginary Frequencies	1

Frequencies (Top 3 out of 72)

```
1.    -185.7311 cm-1
2.      37.0086 cm-1
3.      47.6835 cm-1
```

M06-2X/def2tzvpp-IEFPCM(water) Molecular Geometry in Cartesian Coordinates

```
C           0.054393       -0.240771       -0.049438
C           1.083312       -0.824858       -0.855504
C           2.148744       -1.465671       -0.266967
O          -0.013596       -0.252386        1.172806
O          -0.905638        0.367432       -0.809493
C          -2.041032        0.927255       -0.156834
C          -3.267455        0.042198       -0.326885
C          -4.479810        0.744005        0.275545
C          -3.054692       -1.329321        0.302527
H           1.032494       -0.667856       -1.923402
H           2.813132       -2.061416       -0.873703
H           2.061844       -1.765260        0.766385
H          -1.822294        1.073735        0.900872
H          -2.212127        1.899109       -0.621767
H          -3.433109       -0.086684       -1.400637
H          -4.337721        0.895720        1.348114
H          -4.646889        1.718928       -0.184402
H          -5.380595        0.145571        0.139498
H          -2.184307       -1.831419       -0.121318
H          -2.897023       -1.232207        1.378765
H          -3.925683       -1.965482        0.141003
S           3.938922        0.049102        0.287936
C           3.026004        1.567908       -0.039697
H           2.811078        2.120869        0.874336
```

```
H           3.565860        2.222441       -0.722700
H           2.069687        1.303484       -0.511011
```

7_isobutylacrylate_TS_16

Datum	Value
M06-2X/def2tzvpp-IEFPCM(water) Energy	-862.606336
M06-2X/def2tzvpp-IEFPCM(water) Free Energy (Quasiharmonic)	-862.423369
Number of Imaginary Frequencies	1

Frequencies (Top 3 out of 72)

```
1.    -190.9112 cm-1
2.      28.9688 cm-1
3.      46.3142 cm-1
```

M06-2X/def2tzvpp-IEFPCM(water) Molecular Geometry in Cartesian Coordinates

```
C          -0.074828       -1.109294        0.122423
C          -1.266721       -1.357649       -0.630352
C          -2.492474       -1.387993       -0.004437
O           0.000182       -0.900346        1.326571
O           1.043092       -1.143074       -0.664543
C           2.313445       -0.914190       -0.061927
C           2.841333        0.476384       -0.383537
C           4.262916        0.608058        0.152486
C           1.934818        1.560138        0.186659
H          -1.172945       -1.446227       -1.703116
H          -2.518238       -1.507261        1.067964
H          -3.350825       -1.767301       -0.537400
H           2.238840       -1.049700        1.017051
H           2.983676       -1.672421       -0.469470
H           2.865551        0.575181       -1.472890
H           4.271576        0.496082        1.239270
H           4.922199       -0.150994       -0.270690
H           4.676655        1.588082       -0.085109
H           0.915194        1.464121       -0.187073
H           1.900334        1.489565        1.275939
H           2.304091        2.551060       -0.080416
S          -3.489863        0.787586        0.179263
C          -2.066684        1.709843       -0.426392
H          -1.324673        0.990002       -0.799095
```

```
H          -2.334163        2.372778        -1.248240
H          -1.596487        2.301241         0.359297
```

7_isobutylacrylate_TS_17

Datum	Value
M06-2X/def2tzvpp-IEFPCM(water) Energy	-862.604452
M06-2X/def2tzvpp-IEFPCM(water) Free Energy (Quasiharmonic)	-862.421377
Number of Imaginary Frequencies	1

Frequencies (Top 3 out of 72)

```
1.    -194.4723 cm-1
2.      28.7388 cm-1
3.      40.0847 cm-1
```

M06-2X/def2tzvpp-IEFPCM(water) Molecular Geometry in Cartesian Coordinates

```
C          -0.103593        1.176708         0.078642
C           0.957171        1.091135         1.035732
C           1.476162       -0.099552         1.496675
O          -0.623589        2.212958        -0.321298
O          -0.513671       -0.041440        -0.378318
C          -1.680003       -0.106649        -1.196062
C          -2.888099       -0.559354        -0.384735
C          -3.224686        0.443465         0.714184
C          -2.671289       -1.953530         0.195095
H           1.382702        2.038997         1.335184
H           2.121872       -0.087306         2.361833
H           0.910199       -1.006788         1.353947
H          -1.860107        0.864858        -1.651826
H          -1.457805       -0.834092        -1.977741
H          -3.724023       -0.599963        -1.089931
H          -2.400478        0.515477         1.427832
H          -3.401703        1.437874         0.304691
H          -4.113616        0.129014         1.262007
H          -1.833337       -1.945835         0.894319
H          -3.557102       -2.291258         0.733858
H          -2.451728       -2.679447        -0.589259
S           3.257623       -0.930397         0.126378
C           2.934150        0.143537        -1.284369
H           3.841182        0.641522        -1.624336
```

```
H          2.501195       -0.400607       -2.123328
H          2.219968        0.917196       -0.973339
```

7_isobutylacrylate_TS_18_reopt

Datum	Value
M06-2X/def2tzvpp-IEFPCM(water) Energy	-862.601412
M06-2X/def2tzvpp-IEFPCM(water) Free Energy (Quasiharmonic)	-862.420067
Number of Imaginary Frequencies	1

Frequencies (Top 3 out of 72)

```
1.    -226.4948 cm-1
2.      23.6928 cm-1
3.      37.8301 cm-1
```

M06-2X/def2tzvpp-IEFPCM(water) Molecular Geometry in Cartesian Coordinates

```
C         -0.221664        1.815384       -0.049440
C          1.173055        1.912829        0.245071
C          1.914611        0.874290        0.766753
O         -0.922460        2.703929       -0.525896
O         -0.763053        0.603581        0.253371
C         -2.154418        0.442814        0.003105
C         -2.549407       -0.976155        0.368053
C         -1.795161       -1.994545       -0.479549
C         -4.057204       -1.135257        0.203739
H          1.645078        2.836659       -0.058664
H          2.908382        1.077693        1.140154
H          1.404759        0.051971        1.247915
H         -2.718131        1.169065        0.592549
H         -2.362440        0.635661       -1.053276
H         -2.291587       -1.131348        1.419724
H         -2.037736       -1.856663       -1.536422
H         -0.717022       -1.888669       -0.362858
H         -2.073710       -3.011397       -0.201116
H         -4.346803       -0.967861       -0.836334
H         -4.370766       -2.142117        0.479067
H         -4.604792       -0.426150        0.825682
S          2.850305       -0.703158       -0.746392
C          3.675654       -1.623331        0.582155
H          3.606749       -2.698365        0.422822
```

```
H          4.728695       -1.356544        0.664983
H          3.195754       -1.397656        1.538910
```

7_isobutylacrylate_TS_1_reopt

Datum	Value
M06-2X/def2tzvpp-IEFPCM(water) Energy	-862.60647
M06-2X/def2tzvpp-IEFPCM(water) Free Energy (Quasiharmonic)	-862.423413
Number of Imaginary Frequencies	1

Frequencies (Top 3 out of 72)

```
1.    -196.9032 cm-1
2.      37.4793 cm-1
3.      58.0061 cm-1
```

M06-2X/def2tzvpp-IEFPCM(water) Molecular Geometry in Cartesian Coordinates

```
C         -0.289308        1.647677        0.094431
C         -1.624379        1.574317       -0.411979
C         -2.055257        0.592413       -1.280937
O          0.147277        2.497959        0.865942
O          0.522195        0.654595       -0.361936
C          1.857903        0.644489        0.127446
C          2.575609       -0.562118       -0.448657
C          1.899320       -1.861529       -0.026143
C          4.035016       -0.534900       -0.006760
H         -2.314808        2.294853        0.004116
H         -3.015013        0.708838       -1.762141
H         -1.323484        0.035920       -1.844775
H          2.360634        1.570833       -0.158216
H          1.846468        0.593277        1.220311
H          2.536412       -0.481841       -1.538908
H          1.915296       -1.956692        1.062821
H          0.860713       -1.893707       -0.354106
H          2.419991       -2.722660       -0.446280
H          4.104594       -0.599536        1.081645
H          4.580557       -1.379962       -0.426425
H          4.532401        0.382580       -0.323589
S         -2.768157       -1.386939       -0.170533
C         -2.047387       -0.988924        1.432043
H         -1.762453        0.071223        1.428039
```

```
H          -2.761372       -1.142014        2.240022
H          -1.154314       -1.579074        1.637289
```

7_isobutylacrylate_TS_20_reopt2

Datum	Value
M06-2X/def2tzvpp-IEFPCM(water) Energy	-862.60588
M06-2X/def2tzvpp-IEFPCM(water) Free Energy (Quasiharmonic)	-862.422715
Number of Imaginary Frequencies	1

Frequencies (Top 3 out of 72)

```
    1.    -185.7784 cm-1
    2.      37.0520 cm-1
    3.      47.7003 cm-1
```

M06-2X/def2tzvpp-IEFPCM(water) Molecular Geometry in Cartesian Coordinates

```
C           0.054431       -0.240799       -0.049455
C           1.083320       -0.824820       -0.855587
C           2.148842       -1.465579       -0.267100
O          -0.013500       -0.252418        1.172782
O          -0.905689        0.367357       -0.809456
C          -2.041022        0.927191       -0.156702
C          -3.267494        0.042193       -0.326729
C          -4.479819        0.744084        0.275660
C          -3.054824       -1.329330        0.302709
H           1.032406       -0.667856       -1.923485
H           2.813161       -2.061363       -0.873880
H           2.061954       -1.765229        0.766238
H          -1.822214        1.073629        0.900996
H          -2.212122        1.899074       -0.621575
H          -3.433137       -0.086710       -1.400480
H          -4.646834        1.719005       -0.184315
H          -5.380634        0.145696        0.139612
H          -4.337741        0.895818        1.348228
H          -3.925828       -1.965460        0.141127
H          -2.184433       -1.831469       -0.121077
H          -2.897228       -1.232220        1.378960
S           3.938951        0.049080        0.287631
C           3.025972        1.567950       -0.039538
H           3.565811        2.222708       -0.722338
```

```
H          2.069671        1.303626       -0.510934
H          2.811024        2.120625        0.874662
```

7_isobutylacrylate_TS_21_reopt

Datum	Value
M06-2X/def2tzvpp-IEFPCM(water) Energy	-862.605388
M06-2X/def2tzvpp-IEFPCM(water) Free Energy (Quasiharmonic)	-862.422339
Number of Imaginary Frequencies	1

Frequencies (Top 3 out of 72)

```
1.    -188.6273 cm-1
2.      34.4227 cm-1
3.      43.7481 cm-1
```

M06-2X/def2tzvpp-IEFPCM(water) Molecular Geometry in Cartesian Coordinates

```
C          0.023818       -0.159680       -0.106403
C         -0.875923       -0.595932        0.918075
C         -1.953794       -1.392268        0.605423
O         -0.042344       -0.424034       -1.299985
O          1.024695        0.626028        0.393651
C          2.058587        1.056581       -0.489393
C          3.325987        0.232853       -0.294326
C          3.093334       -1.234872       -0.637706
C          3.871221        0.384197        1.122290
H         -0.721528       -0.212913        1.916571
H         -2.508623       -1.870728        1.397746
H         -1.953509       -1.907147       -0.343145
H          1.708614        0.989768       -1.517536
H          2.249139        2.101794       -0.242344
H          4.058780        0.644168       -0.995452
H          2.350098       -1.669458        0.034661
H          2.730825       -1.351950       -1.658806
H          4.016255       -1.805410       -0.528333
H          4.059423        1.431181        1.364773
H          3.156986       -0.008995        1.847327
H          4.805820       -0.165449        1.238150
S         -3.885239       -0.116479       -0.048085
C         -3.054499        1.480700       -0.130794
H         -3.559932        2.229582        0.477571
```

```
H          -2.034280        1.361450        0.258641
H          -2.985420        1.852345       -1.152793
```

7_isobutylacrylate_TS_22

Datum	Value
M06-2X/def2tzvpp-IEFPCM(water) Energy	-862.602044
M06-2X/def2tzvpp-IEFPCM(water) Free Energy (Quasiharmonic)	-862.419668
Number of Imaginary Frequencies	1

Frequencies (Top 3 out of 72)

```
1.    -152.2033 cm-1
2.      23.7336 cm-1
3.      40.3353 cm-1
```

M06-2X/def2tzvpp-IEFPCM(water) Molecular Geometry in Cartesian Coordinates

```
C          -0.224094        1.040174        0.161615
C          -0.864743        0.447851       -0.990482
C          -2.131470        0.825053       -1.321278
O          -0.732248        1.876436        0.892372
O           1.040801        0.661508        0.482119
C           1.739775       -0.314962       -0.285370
C           3.130649       -0.479501        0.299751
C           3.922590        0.820081        0.213082
C           3.849786       -1.610494       -0.427802
H          -0.373529       -0.342087       -1.535250
H          -2.579791        0.478292       -2.238789
H          -2.562068        1.705293       -0.871380
H           1.198153       -1.263780       -0.252968
H           1.811930        0.011674       -1.327408
H           3.013222       -0.754554        1.351651
H           4.912344        0.697840        0.653580
H           4.052959        1.111962       -0.832041
H           3.413696        1.630984        0.731948
H           4.846926       -1.759163       -0.014103
H           3.303642       -2.550848       -0.345968
H           3.960195       -1.373001       -1.488368
S          -3.841756       -0.556813       -0.005814
C          -2.598008       -1.423884        0.973625
H          -2.688579       -2.505682        0.878633
```

```
H          -1.597449       -1.141290        0.617744
H          -2.658886       -1.166102        2.030960
```

7_isobutylacrylate_TS_2

Datum	Value
M06-2X/def2tzvpp-IEFPCM(water) Energy	-862.605656
M06-2X/def2tzvpp-IEFPCM(water) Free Energy (Quasiharmonic)	-862.423295
Number of Imaginary Frequencies	1

Frequencies (Top 3 out of 72)

```
1.    -199.0610 cm-1
2.      28.9611 cm-1
3.      42.1131 cm-1
```

M06-2X/def2tzvpp-IEFPCM(water) Molecular Geometry in Cartesian Coordinates

```
C          -0.309637        1.564078       -0.033698
C          -1.592539        1.429903       -0.651034
C          -1.970664        0.320784       -1.379664
O           0.089880        2.532838        0.606711
O           0.494463        0.481491       -0.214727
C           1.784431        0.530544        0.382531
C           2.496141       -0.778357        0.093840
C           3.839226       -0.791735        0.815717
C           2.676970       -0.990164       -1.405018
H          -2.291369        2.227563       -0.440632
H          -2.872608        0.369831       -1.971308
H          -1.207025       -0.339921       -1.758674
H           2.350497        1.371876       -0.028475
H           1.682491        0.689400        1.458039
H           1.873701       -1.585520        0.491668
H           4.361023       -1.732555        0.641071
H           4.476620        0.016737        0.450016
H           3.716304       -0.665325        1.891998
H           1.719177       -0.988665       -1.923741
H           3.293912       -0.191917       -1.825664
H           3.174659       -1.939676       -1.604345
S          -2.866125       -1.425211       -0.027169
C          -2.331962       -0.749333        1.555017
H          -1.990323        0.281394        1.392439
```

```
H          -3.148835       -0.722565        2.274865
H          -1.506758       -1.316228        1.985429
```

7_isobutylacrylate_TS_3

Datum	Value
M06-2X/def2tzvpp-IEFPCM(water) Energy	-862.606529
M06-2X/def2tzvpp-IEFPCM(water) Free Energy (Quasiharmonic)	-862.424176
Number of Imaginary Frequencies	1

Frequencies (Top 3 out of 72)

```
1.    -186.7244 cm-1
2.      27.1821 cm-1
3.      41.0682 cm-1
```

M06-2X/def2tzvpp-IEFPCM(water) Molecular Geometry in Cartesian Coordinates

```
C          -0.215736       -0.876500       -0.048053
C          -1.249590       -0.804544       -1.034179
C          -2.526823       -1.222879       -0.734492
O          -0.319785       -1.290116        1.101296
O           0.984214       -0.425474       -0.513281
C           2.072489       -0.455082        0.403709
C           3.308083        0.089461       -0.288488
C           4.504481       -0.039163        0.648663
C           3.107249        1.536775       -0.723323
H          -1.009403       -0.339747       -1.979642
H          -2.667876       -1.858229        0.126834
H          -3.244940       -1.348257       -1.530195
H           1.829254        0.152422        1.280625
H           2.235784       -1.479829        0.743327
H           3.488243       -0.524783       -1.175476
H           4.344102        0.550654        1.554215
H           4.671322       -1.075381        0.945023
H           5.411460        0.327603        0.168318
H           2.258360        1.634897       -1.398460
H           2.923614        2.169057        0.149148
H           3.995379        1.914708       -1.230675
S          -3.852649        0.501927        0.269894
C          -2.532910        1.717737        0.434490
H          -2.298124        1.925697        1.477914
```

```
H          -1.629788        1.314427       -0.044467
H          -2.783897        2.655818       -0.058878
```

7_isobutylacrylate_TS_4

Datum	Value
M06-2X/def2tzvpp-IEFPCM(water) Energy	-862.60647
M06-2X/def2tzvpp-IEFPCM(water) Free Energy (Quasiharmonic)	-862.423411
Number of Imaginary Frequencies	1

Frequencies (Top 3 out of 72)

```
1.    -196.9031 cm-1
2.      37.4937 cm-1
3.      58.0789 cm-1
```

M06-2X/def2tzvpp-IEFPCM(water) Molecular Geometry in Cartesian Coordinates

```
C          -0.289320        1.647743       -0.094523
C          -1.624371        1.574421        0.411947
C          -2.055201        0.592564        1.280938
O           0.147214        2.497927       -0.866179
O           0.522229        0.654748        0.361955
C           1.857910        0.644584       -0.127497
C           2.575504       -0.562213        0.448354
C           4.034936       -0.535004        0.006542
C           1.899121       -1.861469        0.025512
H          -2.314828        2.294857       -0.004274
H          -1.323412        0.036152        1.844841
H          -3.014960        0.708931        1.762157
H           1.846413        0.593573       -1.220367
H           2.360767        1.570817        0.158311
H           2.536247       -0.482184        1.538621
H           4.580333       -1.380340        0.425843
H           4.104559       -0.599149       -1.081890
H           4.532439        0.382258        0.323816
H           2.419825       -2.722753        0.445295
H           0.860563       -1.893719        0.353622
H           1.914927       -1.956281       -1.063485
S          -2.768073       -1.386971        0.171042
C          -2.047193       -0.989180       -1.431530
H          -1.154031       -1.579233       -1.636652
```

```
H          -2.761130       -1.142480       -2.239511
H          -1.762407        0.071009       -1.427668
```

7_isobutylacrylate_TS_5_reopt

Datum	Value
M06-2X/def2tzvpp-IEFPCM(water) Energy	-862.611414
M06-2X/def2tzvpp-IEFPCM(water) Free Energy (Quasiharmonic)	-862.427134
Number of Imaginary Frequencies	1

Frequencies (Top 3 out of 72)

```
1.    -118.0126 cm-1
2.      35.5520 cm-1
3.      51.7955 cm-1
```

M06-2X/def2tzvpp-IEFPCM(water) Molecular Geometry in Cartesian Coordinates

```
C           0.142657        1.580894       -0.162995
C           1.519032        1.496137       -0.090455
C           2.250861        0.589060        0.808567
O          -0.550583        2.252350       -0.960733
O          -0.526884        0.835980        0.825635
C          -1.921421        0.665146        0.666800
C          -2.254569       -0.574145       -0.156140
C          -3.743853       -0.598287       -0.479845
C          -1.834409       -1.843121        0.577733
H           2.072766        2.059350       -0.830664
H           3.148545        1.030877        1.244622
H           1.617622        0.237114        1.618172
H          -2.335187        0.553653        1.673590
H          -2.354982        1.552270        0.206964
H          -1.690330       -0.497079       -1.091463
H          -4.332404       -0.607066        0.441291
H          -4.039052        0.277155       -1.059705
H          -4.007261       -1.489861       -1.049869
H          -1.954453       -2.724528       -0.053940
H          -0.793353       -1.789762        0.896607
H          -2.453353       -1.980758        1.468327
S           2.972228       -0.953165        0.005233
C           1.567776       -1.515699       -0.985439
H           1.711063       -1.284723       -2.038288
```

```
H          1.443738       -2.589390       -0.864387
H          0.674286       -1.003159       -0.633553
```

7_isobutylacrylate_TS_6

Datum	Value
M06-2X/def2tzvpp-IEFPCM(water) Energy	-862.60529
M06-2X/def2tzvpp-IEFPCM(water) Free Energy (Quasiharmonic)	-862.42249
Number of Imaginary Frequencies	1

Frequencies (Top 3 out of 72)

```
1.    -190.3994 cm-1
2.      32.1608 cm-1
3.      40.3573 cm-1
```

M06-2X/def2tzvpp-IEFPCM(water) Molecular Geometry in Cartesian Coordinates

```
C         -0.026686       -0.932750       -0.621886
C         -1.197439       -0.596547       -1.372615
C         -1.803639        0.640211       -1.330188
O          0.567454       -2.006018       -0.660202
O          0.410336        0.076277        0.184103
C          1.643533       -0.125016        0.867479
C          2.850240        0.161722       -0.015219
C          4.124257       -0.090513        0.783466
C          2.809915        1.587345       -0.553351
H         -1.627731       -1.410207       -1.940024
H         -1.252582        1.481009       -0.939427
H         -2.550958        0.880568       -2.071340
H          1.624026        0.574749        1.705279
H          1.686706       -1.142016        1.256363
H          2.815183       -0.536715       -0.855019
H          4.171529        0.577733        1.646859
H          4.170334       -1.117495        1.148141
H          5.007613        0.092730        0.171648
H          2.846582        2.304564        0.270741
H          3.663436        1.779778       -1.204239
H          1.898279        1.770652       -1.121733
S         -3.422452        0.826373        0.427610
C         -2.918868       -0.659198        1.315077
H         -2.409952       -0.425200        2.249745
```

```
H          -3.769711       -1.302824        1.534526
H          -2.223724       -1.222891        0.678941
```

7_isobutylacrylate_TS_7_reopt

Datum	Value
M06-2X/def2tzvpp-IEFPCM(water) Energy	-862.606529
M06-2X/def2tzvpp-IEFPCM(water) Free Energy (Quasiharmonic)	-862.424175
Number of Imaginary Frequencies	1

Frequencies (Top 3 out of 72)

```
1.    -186.7526 cm-1
2.      27.1841 cm-1
3.      41.0917 cm-1
```

M06-2X/def2tzvpp-IEFPCM(water) Molecular Geometry in Cartesian Coordinates

```
C           0.215754       -0.876629        0.048049
C           1.249600       -0.804699        1.034160
C           2.526889       -1.222806        0.734401
O           0.319819       -1.290088       -1.101355
O          -0.984234       -0.425748        0.513342
C          -2.072500       -0.455303       -0.403654
C          -3.308050        0.089433        0.288472
C          -4.504343       -0.038714       -0.648879
C          -3.106922        1.536617        0.723605
H           1.009373       -0.340036        1.979680
H           2.668009       -1.858112       -0.126951
H           3.245015       -1.348208        1.530096
H          -1.829187        0.152088       -1.280625
H          -2.235914       -1.480063       -0.743181
H          -3.488507       -0.524927        1.175320
H          -4.343845        0.551540       -1.554125
H          -4.671171       -1.074784       -0.945763
H          -5.411372        0.327833       -0.168459
H          -2.258254        1.634378        1.399070
H          -2.922768        2.168977       -0.148701
H          -3.995126        1.914745        1.230681
S           3.852492        0.502086       -0.269940
C           2.532626        1.717764       -0.434422
H           2.783501        2.655840        0.059011
```

```
H          2.297802        1.925760       -1.477830
H          1.629563        1.314311        0.044524
```

7_isobutylacrylate_TS_8_reopt

Datum	Value
M06-2X/def2tzvpp-IEFPCM(water) Energy	-862.606259
M06-2X/def2tzvpp-IEFPCM(water) Free Energy (Quasiharmonic)	-862.423555
Number of Imaginary Frequencies	1

Frequencies (Top 3 out of 72)

```
1.    -188.5456 cm-1
2.      34.3439 cm-1
3.      39.2820 cm-1
```

M06-2X/def2tzvpp-IEFPCM(water) Molecular Geometry in Cartesian Coordinates

```
C         -0.066564        0.242373       -0.084472
C         -1.088832        1.142052        0.355098
C         -2.151814        1.441762       -0.465836
O         -0.005572       -0.329503       -1.166352
O          0.901248        0.057128        0.862002
C          2.009942       -0.763083        0.506329
C          3.068135       -0.003944       -0.282095
C          4.200516       -0.958626       -0.644135
C          3.588078        1.192578        0.506249
H         -1.035511        1.502692        1.372299
H         -2.061770        1.229546       -1.520377
H         -2.810194        2.256724       -0.207141
H          2.430525       -1.104856        1.454165
H          1.660494       -1.627397       -0.058128
H          2.597698        0.352424       -1.201747
H          4.669789       -1.353806        0.260275
H          3.838764       -1.802288       -1.233309
H          4.969806       -0.445766       -1.221515
H          2.780706        1.874956        0.770360
H          4.066459        0.858104        1.430458
H          4.327774        1.746027       -0.073167
S         -3.945208       -0.147204       -0.261447
C         -3.050449       -1.332434        0.759235
H         -2.090341       -0.884602        1.049568
```

```
H          -3.596974       -1.572975        1.670193
H          -2.843374       -2.258577        0.223773
```

7_isobutylacrylate_TS_9_reopt

Datum	Value
M06-2X/def2tzvpp-IEFPCM(water) Energy	-862.605619
M06-2X/def2tzvpp-IEFPCM(water) Free Energy (Quasiharmonic)	-862.421613
Number of Imaginary Frequencies	1

Frequencies (Top 3 out of 72)

```
1.    -203.9619 cm-1
2.      40.1835 cm-1
3.      62.8819 cm-1
```

M06-2X/def2tzvpp-IEFPCM(water) Molecular Geometry in Cartesian Coordinates

```
C           0.192919        1.597396        0.141208
C          -1.227928        1.723368        0.054329
C          -2.003382        1.083400       -0.895889
O           0.916788        2.175606        0.946921
O           0.717941        0.759958       -0.800283
C           2.123346        0.522554       -0.804929
C           2.461999       -0.820331       -0.168483
C           1.745812       -1.964017       -0.880534
C           2.151792       -0.826497        1.324425
H          -1.682659        2.321056        0.832157
H          -3.021299        1.414857       -1.036401
H          -1.523864        0.695345       -1.780678
H           2.419939        0.521758       -1.854773
H           2.632608        1.334343       -0.288927
H           3.541069       -0.947233       -0.301640
H           0.664296       -1.862704       -0.768157
H           1.976405       -1.973863       -1.947160
H           2.041210       -2.925662       -0.459502
H           1.085488       -0.661877        1.489799
H           2.416009       -1.787760        1.767389
H           2.697626       -0.041031        1.847006
S          -2.714773       -1.067581       -0.247322
C          -1.796335       -1.155016        1.298481
H          -1.274983       -0.197275        1.437834
```

```
H          -2.454065       -1.310744        2.152563
H          -1.048581       -1.947581        1.282885
```

8_ethylmethacrylate_1

Datum	Value
M06-2X/def2tzvpp-IEFPCM(water) Energy	-385.093777
M06-2X/def2tzvpp-IEFPCM(water) Free Energy (Quasiharmonic)	-384.971744
Number of Imaginary Frequencies	0

Frequencies (Top 3 out of 48)

```
  1.      35.8850 cm-1
  2.      84.9653 cm-1
  3.     131.7870 cm-1
```

M06-2X/def2tzvpp-IEFPCM(water) Molecular Geometry in Cartesian Coordinates

```
C           0.344125       -0.560003        0.000006
C           1.612188        0.235987       -0.000002
C           1.518264        1.731599        0.000012
C           2.755364       -0.439683       -0.000021
O           0.280986       -1.766216        0.000012
O          -0.733536        0.223748        0.000003
C          -2.008782       -0.439326        0.000006
C          -3.075968        0.626124       -0.000015
H           2.513299        2.170674        0.000009
H           0.975648        2.086921       -0.876080
H           0.975661        2.086906        0.876118
H           3.707292        0.074105       -0.000028
H           2.757521       -1.520823       -0.000030
H          -2.067647       -1.076047       -0.882188
H          -2.067656       -1.076023        0.882216
H          -4.058552        0.156840       -0.000013
H          -2.993161        1.254513        0.885516
H          -2.993152        1.254486       -0.885564
```

8_ethylmethacrylate_2

Datum	Value
M06-2X/def2tzvpp-IEFPCM(water) Energy	-385.093248
M06-2X/def2tzvpp-IEFPCM(water) Free Energy (Quasiharmonic)	-384.9707
Number of Imaginary Frequencies	0

Frequencies (Top 3 out of 48)

```
1.      45.3074 cm-1
2.      83.2996 cm-1
3.     120.1157 cm-1
```

M06-2X/def2tzvpp-IEFPCM(water) Molecular Geometry in Cartesian Coordinates

```
C         -0.182047       -0.407029       -0.176780
C         -1.589890        0.043912        0.063191
C         -1.874670        1.514790        0.100777
C         -2.510671       -0.898196        0.230860
O          0.174901       -1.559356       -0.232953
O          0.646692        0.626478       -0.329767
C          2.036676        0.326318       -0.547883
C          2.741551        0.044610        0.760574
H         -2.932840        1.688160        0.282927
H         -1.296818        2.002592        0.885835
H         -1.596239        1.988752       -0.840505
H         -3.546384       -0.641078        0.407981
H         -2.242359       -1.944871        0.193451
H          2.109532       -0.516997       -1.231435
H          2.435385        1.214683       -1.030398
H          3.800855       -0.128303        0.573832
H          2.644602        0.893523        1.435998
H          2.325835       -0.839857        1.239643
```

8_ethylmethacrylate_3

Datum	Value
M06-2X/def2tzvpp-IEFPCM(water) Energy	-385.093595
M06-2X/def2tzvpp-IEFPCM(water) Free Energy (Quasiharmonic)	-384.971085
Number of Imaginary Frequencies	0

Frequencies (Top 3 out of 48)

```
1.      45.2374 cm-1
2.      82.5778 cm-1
3.     110.1466 cm-1
```

M06-2X/def2tzvpp-IEFPCM(water) Molecular Geometry in Cartesian Coordinates

```
C         -0.176615       -0.271849       -0.186268
C         -1.557548        0.243982        0.056454
C         -2.612813       -0.810401        0.190645
C         -1.791060        1.548982        0.143772
O          0.076709       -1.451823       -0.252927
O          0.740669        0.684649       -0.327333
C          2.098708        0.261717       -0.540600
C          2.768126       -0.100118        0.766781
H         -3.584835       -0.355903        0.366519
H         -2.663906       -1.420402       -0.711560
H         -2.380467       -1.483891        1.015895
H         -2.792324        1.918965        0.321945
H         -0.996526        2.273523        0.041470
H          2.100910       -0.575187       -1.235995
H          2.579463        1.116669       -1.008663
H          3.809704       -0.361232        0.582451
H          2.741062        0.743600        1.455059
H          2.275104       -0.952622        1.230252
```

8_ethylmethacrylate_4

Datum	Value
M06-2X/def2tzvpp-IEFPCM(water) Energy	-385.081566
M06-2X/def2tzvpp-IEFPCM(water) Free Energy (Quasiharmonic)	-384.960304
Number of Imaginary Frequencies	0

Frequencies (Top 3 out of 48)

```
1.      24.9000 cm-1
2.      65.2684 cm-1
3.     104.7491 cm-1
```

M06-2X/def2tzvpp-IEFPCM(water) Molecular Geometry in Cartesian Coordinates

```
C          0.386290       0.770959      -0.254732
C          1.279119      -0.415654      -0.045178
C          2.320609      -0.251985       1.019893
C          1.199334      -1.456357      -0.864021
O          0.844306       1.881367      -0.363669
O         -0.934722       0.607526      -0.354631
C         -1.605019      -0.590335       0.088575
C         -3.014752      -0.199713       0.459712
H          2.970184      -1.123390       1.054629
H          2.921815       0.635847       0.824534
H          1.855236      -0.119864       1.998071
H          1.907006      -2.271942      -0.790877
H          0.450432      -1.518906      -1.642071
H         -1.599773      -1.317995      -0.720695
H         -1.070652      -1.010877       0.938357
H         -3.563530      -1.084133       0.780342
H         -3.011475       0.522982       1.274068
H         -3.529405       0.235646      -0.395447
```

8_ethylmethacrylate_5

Datum	Value
M06-2X/def2tzvpp-IEFPCM(water) Energy	-385.081108
M06-2X/def2tzvpp-IEFPCM(water) Free Energy (Quasiharmonic)	-384.958971
Number of Imaginary Frequencies	0

Frequencies (Top 3 out of 48)

```
  1.      49.0212 cm-1
  2.      90.2755 cm-1
  3.     111.4752 cm-1
```

M06-2X/def2tzvpp-IEFPCM(water) Molecular Geometry in Cartesian Coordinates

```
C          0.283461       0.887410       0.120636
C          1.069977      -0.387930       0.187563
C          1.931378      -0.671091      -1.006414
C          1.086082      -1.098055       1.308402
O          0.811139       1.920793      -0.207545
O         -1.005999       0.907854       0.472939
C         -1.837308      -0.270216       0.429891
```

```
C          -1.993706      -0.781085      -0.984103
H           1.328014      -0.784249      -1.908691
H           2.614707       0.160471      -1.178645
H           2.507333      -1.580673      -0.853567
H           1.732171      -1.960781       1.406466
H           0.483569      -0.829597       2.166131
H          -1.443017      -1.036020       1.091020
H          -2.787772       0.073396       0.829173
H          -2.359738       0.009412      -1.637822
H          -1.046748      -1.152824      -1.376072
H          -2.708941      -1.602516      -0.996993
```

8_ethylmethacrylate_6

Datum	Value
M06-2X/def2tzvpp-IEFPCM(water) Energy	-385.08076
M06-2X/def2tzvpp-IEFPCM(water) Free Energy (Quasiharmonic)	-384.959031
Number of Imaginary Frequencies	0

Frequencies (Top 3 out of 48)

```
1.      44.3308 cm-1
2.      95.9299 cm-1
3.     109.4089 cm-1
```

M06-2X/def2tzvpp-IEFPCM(water) Molecular Geometry in Cartesian Coordinates

```
C          -0.388829       0.888799      -0.156737
C          -1.086533      -0.442064      -0.142481
C          -1.849651      -0.740204       1.115363
C          -1.060855      -1.216982      -1.216372
O          -0.918979       1.884110      -0.580719
O           0.832614       0.979432       0.371281
C           1.578463      -0.188468       0.764440
C           2.450082      -0.666360      -0.372963
H          -1.197059      -0.728669       1.990288
H          -2.614672       0.021178       1.276701
H          -2.329536      -1.713832       1.046948
H          -1.598113      -2.156519      -1.231621
H          -0.515155      -0.938821      -2.108541
H           2.176174       0.139248       1.611260
H           0.901589      -0.969886       1.103034
H           1.840983      -1.004328      -1.210755
```

```
H          3.104776      0.134501      -0.713145
H          3.065868     -1.499532      -0.036169
```

8_ethylmethacrylate_HEI_10_reopt

Datum	Value
M06-2X/def2tzvpp-IEFPCM(water) Energy	-823.300393
M06-2X/def2tzvpp-IEFPCM(water) Free Energy (Quasiharmonic)	-823.144423
Number of Imaginary Frequencies	0

Frequencies (Top 3 out of 63)

```
1.      35.2152 cm-1
2.      52.5087 cm-1
3.      68.5606 cm-1
```

M06-2X/def2tzvpp-IEFPCM(water) Molecular Geometry in Cartesian Coordinates

```
C          0.839409     -0.258718      -0.009594
C         -0.251323      0.422232       0.486583
C         -0.338194      1.927759       0.452967
C         -1.369650     -0.368231       1.040907
O          1.041113     -1.498331      -0.055170
O          1.827838      0.589261      -0.538401
C          3.087771      0.001977      -0.806398
C          3.909031     -0.173897       0.457937
H         -1.182290      2.267888       1.058321
H          0.560387      2.405472       0.852673
H         -0.486022      2.348677      -0.549143
H         -1.898693      0.172432       1.828797
H         -1.031283     -1.323306       1.441684
H          2.954949     -0.956243      -1.307668
H          3.588941      0.685639      -1.492682
H          4.890708     -0.587350       0.222951
H          4.050824      0.784888       0.957737
H          3.400546     -0.853550       1.140759
C         -3.245228      0.720083      -0.723072
H         -2.430371      1.217833      -1.244198
H         -3.571054      1.339275       0.112514
H         -4.078151      0.581208      -1.408747
S         -2.719437     -0.902346      -0.141276
```

8_ethylmethacrylate_HEI_11

Datum	Value
M06-2X/def2tzvpp-IEFPCM(water) Energy	-823.30149
M06-2X/def2tzvpp-IEFPCM(water) Free Energy (Quasiharmonic)	-823.147346
Number of Imaginary Frequencies	0

Frequencies (Top 3 out of 63)

```
1.      25.9999 cm-1
2.      49.3823 cm-1
3.      52.0689 cm-1
```

M06-2X/def2tzvpp-IEFPCM(water) Molecular Geometry in Cartesian Coordinates

```
C         -1.070293        0.963445       -0.007700
C          0.248925        1.208775        0.311305
C          0.825633        2.584753        0.138577
C          1.171099        0.134791        0.743920
O         -1.952304        1.772956       -0.390624
O         -1.447857       -0.382943        0.126803
C         -2.815169       -0.677659       -0.080365
C         -2.994795       -2.170067        0.096206
H          0.049009        3.290087       -0.153725
H          1.286044        2.960063        1.061144
H          1.609879        2.622690       -0.628672
H          0.652433       -0.715918        1.180076
H          1.910706        0.501565        1.462017
H         -3.434117       -0.126741        0.632565
H         -3.122885       -0.366489       -1.080619
H         -2.386242       -2.717603       -0.623719
H         -4.038324       -2.445563       -0.055812
H         -2.699964       -2.478201        1.099421
C          3.299955       -1.622503        0.238354
H          3.895169       -1.048377        0.947213
H          3.965849       -2.100453       -0.476745
H          2.745059       -2.391578        0.773963
S          2.173534       -0.539550       -0.666770
```

8_ethylmethacrylate_HEI_12_reopt2

Datum	Value
M06-2X/def2tzvpp-IEFPCM(water) Energy	-823.300333
M06-2X/def2tzvpp-IEFPCM(water) Free Energy (Quasiharmonic)	-823.144765
Number of Imaginary Frequencies	0

Frequencies (Top 3 out of 63)

```
1.      36.9777 cm-1
2.      43.0164 cm-1
3.      61.9617 cm-1
```

M06-2X/def2tzvpp-IEFPCM(water) Molecular Geometry in Cartesian Coordinates

```
C          0.983136        0.907178       -0.148228
C         -0.368750        1.076218       -0.341100
C         -1.036658        2.263598        0.304129
C         -1.171535        0.130520       -1.148594
O          1.761579        1.621371        0.532610
O          1.535436       -0.187623       -0.840406
C          2.732720       -0.719371       -0.305030
C          2.467341       -1.534358        0.947983
H         -1.105589        2.195432        1.396963
H         -0.506382        3.197347        0.092556
H         -2.058191        2.377746       -0.066119
H         -0.599690       -0.336078       -1.948345
H         -2.038316        0.624580       -1.592343
H          3.439491        0.082858       -0.094590
H          3.151133       -1.350899       -1.089402
H          1.767637       -2.343096        0.735193
H          3.392761       -1.967708        1.329158
H          2.038270       -0.898919        1.722998
C         -2.765275       -0.509036        1.056805
H         -3.518822        0.162342        0.645852
H         -3.254798       -1.260104        1.672725
H         -2.068685        0.060704        1.668322
S         -1.878801       -1.356418       -0.262900
```

8_ethylmethacrylate_HEI_1_reopt

Datum	Value
M06-2X/def2tzvpp-IEFPCM(water) Energy	-823.302755

Datum	Value
M06-2X/def2tzvpp-IEFPCM(water) Free Energy (Quasiharmonic)	-823.147707
Number of Imaginary Frequencies	0

Frequencies (Top 3 out of 63)

```
1.      40.5086 cm-1
2.      54.4829 cm-1
3.      71.0200 cm-1
```

M06-2X/def2tzvpp-IEFPCM(water) Molecular Geometry in Cartesian Coordinates

```
C         -0.646088      -0.137978      -0.567278
C          0.389029       0.771846      -0.473490
C          0.317153       2.022538       0.354939
C          1.700970       0.401203      -1.032530
O         -0.673133      -1.246423      -1.160007
O         -1.814768       0.266039       0.095325
C         -2.958983      -0.543303      -0.095936
C         -4.086496       0.054489       0.717664
H          0.900831       2.827817      -0.104871
H         -0.706014       2.377361       0.466585
H          0.726655       1.892533       1.366959
H          2.203134       1.229864      -1.539717
H          1.617313      -0.435933      -1.721049
H         -3.224300      -0.578612      -1.155998
H         -2.754948      -1.568299       0.218715
H         -4.992777      -0.539556       0.600749
H         -3.825427       0.078450       1.775803
H         -4.298909       1.073318       0.393529
C          2.064211      -1.318454       1.157393
H          1.085098      -0.908664       1.404748
H          2.608867      -1.545165       2.071143
H          1.935096      -2.230084       0.576956
S          2.996863      -0.083250       0.237458
```

8_ethylmethacrylate_HEI_2

Datum	Value
M06-2X/def2tzvpp-IEFPCM(water) Energy	-823.302939
M06-2X/def2tzvpp-IEFPCM(water) Free Energy (Quasiharmonic)	-823.148171

Datum	Value
Number of Imaginary Frequencies	0

Frequencies (Top 3 out of 63)

```
1.      28.7281 cm-1
2.      50.4827 cm-1
3.      76.5432 cm-1
```

M06-2X/def2tzvpp-IEFPCM(water) Molecular Geometry in Cartesian Coordinates

```
C          0.595790       1.122257       0.042816
C         -0.721349       1.097923      -0.369137
C         -1.661089       2.179409       0.079229
C         -1.299605      -0.045382      -1.098899
O          1.182065       1.984772       0.744450
O          1.363749       0.040151      -0.417510
C          2.628075      -0.131186       0.191002
C          3.261840      -1.369581      -0.405169
H         -1.108106       3.044978       0.442059
H         -2.311165       2.513890      -0.737616
H         -2.330258       1.857065       0.888697
H         -0.548705      -0.649845      -1.599977
H         -2.045746       0.263816      -1.835900
H          3.255893       0.745688       0.022673
H          2.510780      -0.240958       1.273262
H          4.243775      -1.541539       0.035498
H          3.383714      -1.257093      -1.482665
H          2.642269      -2.247256      -0.219645
C         -1.000179      -1.736179       1.109351
H         -0.516839      -0.842760       1.504548
H         -0.253929      -2.360876       0.621439
H         -1.463459      -2.290653       1.922340
S         -2.290352      -1.243588      -0.046587
```

8_ethylmethacrylate_HEI_3_reopt

Datum	Value
M06-2X/def2tzvpp-IEFPCM(water) Energy	-823.302744
M06-2X/def2tzvpp-IEFPCM(water) Free Energy (Quasiharmonic)	-823.147082
Number of Imaginary Frequencies	0

Frequencies (Top 3 out of 63)

```
1.      37.2471 cm-1
2.      50.0240 cm-1
3.      61.9410 cm-1
```

M06-2X/def2tzvpp-IEFPCM(water) Molecular Geometry in Cartesian Coordinates

```
C          0.770749        0.386434        0.609652
C         -0.357152        1.030510        0.150015
C         -0.375867        2.006750       -0.990607
C         -1.653909        0.715575        0.783311
O          0.866035       -0.482975        1.514202
O          1.966687        0.808046       -0.007114
C          3.059134       -0.086922        0.081868
C          2.891919       -1.269054       -0.855952
H         -0.772547        2.985147       -0.689488
H          0.619378        2.166999       -1.398378
H         -1.018225        1.660918       -1.810408
H         -2.277842        1.601977        0.931386
H         -1.522443        0.217293        1.741381
H          3.938594        0.496369       -0.193484
H          3.181066       -0.431386        1.108388
H          3.754263       -1.934179       -0.795975
H          1.999552       -1.833239       -0.585070
H          2.790548       -0.928559       -1.886993
C         -1.717210       -1.820320       -0.384084
H         -0.717262       -1.483315       -0.657207
H         -2.117883       -2.458103       -1.168767
H         -1.668357       -2.380281        0.548004
S         -2.784788       -0.378628       -0.222832
```

8_ethylmethacrylate_HEI_4_reopt

Datum	Value
M06-2X/def2tzvpp-IEFPCM(water) Energy	-823.302663
M06-2X/def2tzvpp-IEFPCM(water) Free Energy (Quasiharmonic)	-823.147019
Number of Imaginary Frequencies	0

Frequencies (Top 3 out of 63)

```
1.      46.0448 cm-1
2.      50.2502 cm-1
3.      68.7838 cm-1
```

M06-2X/def2tzvpp-IEFPCM(water) Molecular Geometry in Cartesian Coordinates

```
C          -0.756225       -0.195854       -0.160218
C           0.261530        0.728903       -0.266712
C           0.344248        1.972340        0.571456
C           1.431420        0.395615       -1.102516
O          -0.896375       -1.290225       -0.763918
O          -1.751264        0.161293        0.769850
C          -3.005562       -0.477634        0.619718
C          -3.797028        0.102780       -0.538361
H           0.793819        2.794669        0.003533
H          -0.638025        2.298635        0.908067
H           0.969471        1.846653        1.466706
H           1.775193        1.235518       -1.713371
H           1.225700       -0.451771       -1.752480
H          -2.866442       -1.549678        0.485026
H          -3.532037       -0.313067        1.560519
H          -4.770458       -0.382502       -0.619528
H          -3.956241        1.172047       -0.395661
H          -3.256716       -0.048166       -1.472608
C           2.344310       -1.293887        0.943844
H           1.416696       -0.935303        1.389931
H           3.079469       -1.476687        1.724238
H           2.151380       -2.220009        0.405177
S           2.990822       -0.020779       -0.152517
```

8_ethylmethacrylate_HEI_5_reopt

Datum	Value
M06-2X/def2tzvpp-IEFPCM(water) Energy	-823.300555
M06-2X/def2tzvpp-IEFPCM(water) Free Energy (Quasiharmonic)	-823.14554
Number of Imaginary Frequencies	0

Frequencies (Top 3 out of 63)

```
1.      35.8449 cm-1
2.      44.6652 cm-1
3.      71.4394 cm-1
```

M06-2X/def2tzvpp-IEFPCM(water) Molecular Geometry in Cartesian Coordinates

```
C        -0.741290      -0.474590       0.309503
C         0.330927       0.309162       0.680325
C         0.239767       1.813800       0.732531
C         1.599593      -0.367750       1.015762
O        -0.805196      -1.725257       0.205148
O        -1.896903       0.265195       0.022322
C        -3.053144      -0.477848      -0.313654
C        -4.173788       0.504561      -0.576142
H         0.132147       2.290569      -0.249445
H        -0.605265       2.163686       1.333022
H         1.146061       2.228601       1.180440
H         1.435415      -1.366886       1.418973
H         2.189249       0.205459       1.734191
H        -2.863346      -1.094224      -1.195075
H        -3.316925      -1.155522       0.501645
H        -5.087423      -0.027603      -0.840713
H        -4.371840       1.108645       0.309363
H        -3.915312       1.173222      -1.397303
C         2.991922       0.968128      -1.004859
H         2.035829       1.362786      -1.342599
H         3.686347       0.943276      -1.841727
H         3.394769       1.615467      -0.226125
S         2.787073      -0.713734      -0.391575
```

8_ethylmethacrylate_HEI_6

Datum	Value
M06-2X/def2tzvpp-IEFPCM(water) Energy	-823.302694
M06-2X/def2tzvpp-IEFPCM(water) Free Energy (Quasiharmonic)	-823.147184
Number of Imaginary Frequencies	0

Frequencies (Top 3 out of 63)

```
1.      29.2893 cm-1
2.      41.5547 cm-1
3.      74.5564 cm-1
```

M06-2X/def2tzvpp-IEFPCM(water) Molecular Geometry in Cartesian Coordinates

```
C        -0.832912      0.594089      0.340951
C         0.338636      0.999909     -0.262949
C         0.910799      2.350856      0.054522
C         1.145104      0.109492     -1.119882
O        -1.591956      1.252839      1.096386
O        -1.194830     -0.739884      0.067400
C        -2.560441     -1.063930      0.250214
C        -3.425940     -0.508587     -0.866433
H         0.169981      2.975523      0.551373
H         1.790267      2.297623      0.709955
H         1.239085      2.873157     -0.851903
H         1.572194      0.635931     -1.978487
H         0.578543     -0.743426     -1.482807
H        -2.602675     -2.153723      0.258404
H        -2.907277     -0.698056      1.216360
H        -4.467819     -0.798778     -0.725339
H        -3.091587     -0.885469     -1.833536
H        -3.368016      0.579450     -0.875700
C         1.930672     -1.453305      1.067877
H         1.405701     -2.348232      0.738759
H         1.225212     -0.781665      1.556177
H         2.716580     -1.730452      1.766833
S         2.687412     -0.593542     -0.321636
```

8_ethylmethacrylate_HEI_7_reopt

Datum	Value
M06-2X/def2tzvpp-IEFPCM(water) Energy	-823.300484
M06-2X/def2tzvpp-IEFPCM(water) Free Energy (Quasiharmonic)	-823.145279
Number of Imaginary Frequencies	0

Frequencies (Top 3 out of 63)

```
1.      43.1518 cm-1
2.      66.3112 cm-1
3.      73.5543 cm-1
```

M06-2X/def2tzvpp-IEFPCM(water) Molecular Geometry in Cartesian Coordinates

```
C          0.793855       0.984655      -0.031979
C         -0.501866       0.936475       0.436619
C         -1.355963       2.166936       0.269820
C         -1.064169      -0.273119       1.071592
O          1.377906       1.954364      -0.576889
O          1.518433      -0.202915       0.136559
C          2.877733      -0.170572      -0.253742
C          3.464381      -1.540821       0.007564
H         -2.302024       2.051515       0.803989
H         -0.866849       3.064306       0.662676
H         -1.610401       2.393949      -0.773204
H         -1.838927      -0.018928       1.798147
H         -0.308797      -0.874834       1.574348
H          2.960875       0.091748      -1.310464
H          3.415203       0.595139       0.311067
H          4.514656      -1.563460      -0.282772
H          3.394423      -1.795142       1.065244
H          2.932757      -2.301540      -0.564299
C         -3.100726      -0.475288      -0.836456
H         -3.768075      -0.037039      -0.094726
H         -3.684025      -1.081895      -1.525664
H         -2.604159       0.319643      -1.388996
S         -1.880302      -1.539041      -0.045826
```

8_ethylmethacrylate_HEI_8

Datum	Value
M06-2X/def2tzvpp-IEFPCM(water) Energy	-823.301403
M06-2X/def2tzvpp-IEFPCM(water) Free Energy (Quasiharmonic)	-823.146944
Number of Imaginary Frequencies	0

Frequencies (Top 3 out of 63)

```
1.      42.4194 cm-1
2.      52.4373 cm-1
3.      62.7799 cm-1
```

M06-2X/def2tzvpp-IEFPCM(water) Molecular Geometry in Cartesian Coordinates

```
C         -0.837121      -0.322257       0.311205
C          0.177715       0.603077       0.436546
C          0.021918       2.075240       0.178280
```

```
C          1.534665       0.110011       0.760489
O         -0.778866      -1.569810       0.461714
O         -2.085286       0.228023      -0.018049
C         -3.165759      -0.680149      -0.110118
C         -4.406568       0.114206      -0.456079
H          0.550532       2.407929      -0.724947
H         -1.023670       2.351079       0.058911
H          0.428820       2.674161       1.002418
H          1.506856      -0.865299       1.243713
H          2.087456       0.808574       1.395764
H         -2.962904      -1.433541      -0.874577
H         -3.296538      -1.210202       0.836100
H         -5.267727      -0.548634      -0.539618
H         -4.616771       0.855736       0.314795
H         -4.278895       0.633397      -1.406066
C          4.165501      -0.511852       0.009318
H          4.503299       0.296468       0.656550
H          4.899541      -0.663429      -0.778976
H          4.077917      -1.427512       0.592499
S          2.585200      -0.092130      -0.756483
```

8_ethylmethacrylate_HEI_9_reopt2

Datum	Value
M06-2X/def2tzvpp-IEFPCM(water) Energy	-823.300163
M06-2X/def2tzvpp-IEFPCM(water) Free Energy (Quasiharmonic)	-823.145506
Number of Imaginary Frequencies	0

Frequencies (Top 3 out of 63)

```
1.       9.3964 cm-1
2.      39.8771 cm-1
3.      62.1654 cm-1
```

M06-2X/def2tzvpp-IEFPCM(water) Molecular Geometry in Cartesian Coordinates

```
C          0.872471      -0.001726      -0.624169
C         -0.316292       0.692562      -0.562534
C         -0.399532       2.078290       0.028557
C         -1.529859       0.047014      -1.103127
O          1.091523      -1.155018      -1.072059
O          1.964261       0.730819      -0.126630
C          3.183888       0.028358       0.025969
```

```
C           3.195492       -0.816708        1.286858
H          -0.207188        2.114191        1.107351
H           0.308086        2.776691       -0.428614
H          -1.400091        2.490154       -0.122867
H          -1.295944       -0.662263       -1.896887
H          -2.243306        0.778089       -1.489258
H           3.370511       -0.594062       -0.848578
H           3.958139        0.794978        0.076228
H           4.160519       -1.310550        1.408437
H           3.015331       -0.194711        2.164338
H           2.419239       -1.578510        1.230597
C          -2.866026        0.223577        1.345772
H          -1.932062        0.577441        1.777695
H          -3.467040       -0.244003        2.122522
H          -3.415002        1.068496        0.930530
S          -2.534895       -1.008160        0.073379
```

8_ethylmethacrylate_TS_10_reopt

Datum	Value
M06-2X/def2tzvpp-IEFPCM(water) Energy	-823.296238
M06-2X/def2tzvpp-IEFPCM(water) Free Energy (Quasiharmonic)	-823.140805
Number of Imaginary Frequencies	1

Frequencies (Top 3 out of 63)

```
1.    -224.8647 cm-1
2.      51.4355 cm-1
3.      66.4367 cm-1
```

M06-2X/def2tzvpp-IEFPCM(water) Molecular Geometry in Cartesian Coordinates

```
C           0.755269       -0.017236        0.305071
C          -0.293924        0.949489        0.317833
C          -0.335706        2.059681       -0.697215
C          -1.359744        0.738821        1.182746
O           0.859042       -1.017110        1.017099
O           1.722770        0.265696       -0.625898
C           2.859060       -0.592948       -0.661896
C           3.875138       -0.215456        0.397304
H          -1.085519        2.797606       -0.408283
H           0.621595        2.573286       -0.790145
H          -0.603669        1.704001       -1.698874
```

```
H          -2.012617        1.571138        1.407646
H          -1.223619        0.033434        1.988634
H           2.539071       -1.626689       -0.542382
H           3.277579       -0.468238       -1.659438
H           4.756369       -0.852128        0.315955
H           4.187555        0.821301        0.272849
H           3.448695       -0.338010        1.391457
C          -2.107453       -1.157959       -1.157863
H          -1.117580       -0.685586       -1.157001
H          -2.567849       -0.977239       -2.128304
H          -1.972742       -2.231137       -1.027594
S          -3.078600       -0.431049        0.174374
```

8_ethylmethacrylate_TS_11

Datum	Value
M06-2X/def2tzvpp-IEFPCM(water) Energy	-823.291891
M06-2X/def2tzvpp-IEFPCM(water) Free Energy (Quasiharmonic)	-823.138658
Number of Imaginary Frequencies	1

Frequencies (Top 3 out of 63)

```
1.    -258.2122 cm-1
2.      26.7295 cm-1
3.      48.7110 cm-1
```

M06-2X/def2tzvpp-IEFPCM(water) Molecular Geometry in Cartesian Coordinates

```
C          -1.193619        0.910841        0.013757
C           0.116088        1.325157        0.394083
C           0.490654        2.750735        0.099852
C           1.065384        0.410341        0.824832
O          -2.077818        1.641658       -0.434572
O          -1.439330       -0.423885        0.184257
C          -2.748853       -0.873613       -0.147121
C          -2.798920       -2.362025        0.108630
H          -0.277378        3.450519        0.432689
H           1.424496        3.008870        0.600925
H           0.635486        2.930399       -0.972028
H           0.737923       -0.560757        1.167172
H           1.949976        0.790330        1.321570
H          -3.482400       -0.341905        0.461071
H          -2.959835       -0.643818       -1.192454
```

```
H          -2.062387       -2.881827       -0.503478
H          -3.787097       -2.748958       -0.137538
H          -2.594496       -2.580245        1.156416
C           3.304229       -1.529931        0.436279
H           3.620801       -0.897386        1.268214
H           4.193122       -1.986510        0.003957
H           2.672470       -2.323816        0.835998
S           2.416045       -0.546632       -0.801366
```

8_ethylmethacrylate_TS_12_reopt

Datum	Value
M06-2X/def2tzvpp-IEFPCM(water) Energy	-823.2968
M06-2X/def2tzvpp-IEFPCM(water) Free Energy (Quasiharmonic)	-823.142245
Number of Imaginary Frequencies	1

Frequencies (Top 3 out of 63)

```
1.    -234.6241 cm-1
2.      31.9289 cm-1
3.      32.3961 cm-1
```

M06-2X/def2tzvpp-IEFPCM(water) Molecular Geometry in Cartesian Coordinates

```
C           0.849516        0.931878       -0.063083
C          -0.516754        1.268994       -0.287967
C          -1.147119        2.257549        0.652805
C          -1.295326        0.577832       -1.208894
O           1.593395        1.453702        0.769524
O           1.330874       -0.051364       -0.890396
C           2.650808       -0.520125       -0.632891
C           2.665741       -1.531146        0.496393
H          -1.395253        1.807055        1.621913
H          -0.488029        3.102127        0.856547
H          -2.076014        2.644325        0.231210
H          -0.801085       -0.017532       -1.960298
H          -2.222685        1.032813       -1.530509
H           3.303424        0.322112       -0.409464
H           2.977084       -0.978082       -1.565426
H           2.014220       -2.372709        0.260872
H           3.676609       -1.909202        0.650860
H           2.321723       -1.070088        1.421330
C          -1.332994       -1.397096        1.181496
```

```
H          -1.892721       -1.365875        2.115434
H          -0.774288       -2.331188        1.137594
H          -0.614097       -0.568360        1.187235
S          -2.416642       -1.190089       -0.242342
```

8_ethylmethacrylate_TS_1

Datum	Value
M06-2X/def2tzvpp-IEFPCM(water) Energy	-823.296732
M06-2X/def2tzvpp-IEFPCM(water) Free Energy (Quasiharmonic)	-823.14183
Number of Imaginary Frequencies	1

Frequencies (Top 3 out of 63)

```
1.    -227.1755 cm-1
2.      42.8545 cm-1
3.      56.0946 cm-1
```

M06-2X/def2tzvpp-IEFPCM(water) Molecular Geometry in Cartesian Coordinates

```
C          -0.648057       -0.093216        0.669581
C           0.381458       -1.019357        0.326308
C           0.220257       -1.955994       -0.840377
C           1.599354       -0.908125        0.984499
O          -0.611263        0.780987        1.537058
O          -1.782254       -0.254957       -0.081525
C          -2.865410        0.622664        0.207703
C          -3.991959        0.288623       -0.742413
H           1.006844       -2.711713       -0.820364
H          -0.740349       -2.471803       -0.828741
H           0.292801       -1.441719       -1.805874
H           2.286155       -1.742430        0.940849
H           1.626142       -0.341281        1.902956
H          -3.171963        0.494032        1.246970
H          -2.538085        1.656334        0.086937
H          -4.843780        0.941034       -0.553655
H          -3.676165        0.424583       -1.776374
H          -4.313206       -0.744294       -0.611448
C           1.853207        1.352931       -1.126231
H           0.886130        0.849088       -1.008413
H           2.112539        1.343835       -2.184070
H           1.740417        2.386581       -0.801200
S           3.074223        0.464022       -0.144142
```

8_ethylmethacrylate_TS_2

Datum	Value
M06-2X/def2tzvpp-IEFPCM(water) Energy	-823.296957
M06-2X/def2tzvpp-IEFPCM(water) Free Energy (Quasiharmonic)	-823.142367
Number of Imaginary Frequencies	1

Frequencies (Top 3 out of 63)

```
1.    -237.7302 cm-1
2.      37.5853 cm-1
3.      45.6897 cm-1
```

M06-2X/def2tzvpp-IEFPCM(water) Molecular Geometry in Cartesian Coordinates

```
C          0.689195        1.039675        0.045855
C         -0.618736        1.147366       -0.509179
C         -1.485099        2.272794       -0.016605
C         -1.140268        0.155248       -1.331537
O          1.221886        1.848843        0.808323
O          1.377056       -0.079037       -0.341997
C          2.687884       -0.232452        0.190448
C          3.242000       -1.537959       -0.331213
H         -0.934329        3.212495        0.040067
H         -2.335913        2.417159       -0.683956
H         -1.888886        2.080893        0.985078
H         -0.459330       -0.540837       -1.795317
H         -2.012112        0.397422       -1.924815
H          3.308832        0.611625       -0.114067
H          2.641186       -0.230337        1.280585
H          4.247701       -1.697228        0.056178
H          3.289121       -1.529353       -1.419767
H          2.615503       -2.372974       -0.018395
C         -1.489619       -1.173512        1.440899
H         -0.921647       -0.237751        1.377859
H         -0.798286       -1.974970        1.699315
H         -2.222973       -1.071373        2.239604
S         -2.289534       -1.453136       -0.149061
```

8_ethylmethacrylate_TS_3

Datum	Value
M06-2X/def2tzvpp-IEFPCM(water) Energy	-823.296624
M06-2X/def2tzvpp-IEFPCM(water) Free Energy (Quasiharmonic)	-823.141276
Number of Imaginary Frequencies	1

Frequencies (Top 3 out of 63)

```
 1.    -226.8481 cm-1
 2.      44.4419 cm-1
 3.      55.3320 cm-1
```

M06-2X/def2tzvpp-IEFPCM(water) Molecular Geometry in Cartesian Coordinates

```
C          0.755426      -0.524470      -0.551609
C         -0.408079      -1.205821      -0.085931
C         -0.420145      -1.888886       1.254938
C         -1.579765      -1.055106      -0.816213
O          0.868086       0.141602      -1.581640
O          1.833510      -0.684209       0.282673
C          3.015932       0.038665      -0.047860
C          2.929858       1.477557       0.420852
H         -1.316862      -2.502931       1.349725
H          0.445432      -2.536251       1.398943
H         -0.427180      -1.177939       2.089443
H         -2.391735      -1.746823      -0.637226
H         -1.499049      -0.697390      -1.831473
H          3.822726      -0.486343       0.461161
H          3.187510      -0.010776      -1.121842
H          3.859371       2.002255       0.198976
H          2.111972       1.990802      -0.082840
H          2.760470       1.517274       1.496921
C         -1.498923       1.624122       0.748108
H         -0.626046       0.961640       0.799249
H         -1.775253       1.898124       1.765427
H         -1.209637       2.524932       0.207610
S         -2.832519       0.737995      -0.077254
```

8_ethylmethacrylate_TS_4

Datum	Value

Datum	Value
M06-2X/def2tzvpp-IEFPCM(water) Energy	-823.296238
M06-2X/def2tzvpp-IEFPCM(water) Free Energy (Quasiharmonic)	-823.140804
Number of Imaginary Frequencies	1

Frequencies (Top 3 out of 63)

```
1.    -224.8127 cm-1
2.      51.4406 cm-1
3.      66.4346 cm-1
```

M06-2X/def2tzvpp-IEFPCM(water) Molecular Geometry in Cartesian Coordinates

```
C          0.755276       -0.017351        0.305015
C         -0.293895        0.949410        0.317851
C         -0.335555        2.059728       -0.697069
C         -1.359691        0.738737        1.182776
O          0.858996       -1.017304        1.016932
O          1.722802        0.265667       -0.625895
C          2.859158       -0.592891       -0.661925
C          3.875154       -0.215453        0.397377
H         -1.085596        2.797474       -0.408276
H          0.621682        2.573527       -0.789615
H         -0.603117        1.704139       -1.698863
H         -2.012525        1.571061        1.407757
H         -1.223617        0.033252        1.988587
H          2.539239       -1.626672       -0.542565
H          3.277728       -0.468018       -1.659425
H          4.756410       -0.852089        0.316032
H          4.187542        0.821325        0.273028
H          3.448658       -0.338100        1.391494
C         -2.107598       -1.157766       -1.157951
H         -1.117734       -0.685367       -1.157070
H         -2.568021       -0.976985       -2.128367
H         -1.972849       -2.230950       -1.027771
S         -3.078704       -0.430999        0.174395
```

8_ethylmethacrylate_TS_5_reopt

Datum	Value
M06-2X/def2tzvpp-IEFPCM(water) Energy	-823.296732

Datum	Value
M06-2X/def2tzvpp-IEFPCM(water) Free Energy (Quasiharmonic)	-823.141832
Number of Imaginary Frequencies	1

Frequencies (Top 3 out of 63)

```
1.    -227.1311 cm-1
2.      42.8469 cm-1
3.      56.0697 cm-1
```

M06-2X/def2tzvpp-IEFPCM(water) Molecular Geometry in Cartesian Coordinates

```
C        -0.647998        0.093444        0.669596
C         0.381499        1.019547        0.326184
C         0.220383        1.955874       -0.840758
C         1.599375        0.908364        0.984408
O        -0.611235       -0.780542        1.537285
O        -1.782155        0.254950       -0.081622
C        -2.865250       -0.622693        0.207765
C        -3.991888       -0.288790       -0.742289
H         0.293740        1.441439       -1.806119
H        -0.740538        2.471094       -0.829710
H         1.006505        2.712069       -0.820504
H         1.626077        0.341691        1.902971
H         2.286262        1.742587        0.940581
H        -2.537881       -1.656358        0.087057
H        -3.171715       -0.493987        1.247047
H        -4.843679       -0.941196       -0.553378
H        -4.313143        0.744136       -0.611413
H        -3.676188       -0.424863       -1.776263
C         1.852953       -1.353064       -1.126122
H         0.885890       -0.849230       -1.008158
H         1.740201       -2.386753       -0.801203
H         2.112176       -1.343851       -2.183987
S         3.074061       -0.464259       -0.144058
```

8_ethylmethacrylate_TS_6

Datum	Value
M06-2X/def2tzvpp-IEFPCM(water) Energy	-823.296363
M06-2X/def2tzvpp-IEFPCM(water) Free Energy (Quasiharmonic)	-823.141501

Datum	Value
Number of Imaginary Frequencies	1

Frequencies (Top 3 out of 63)

```
1.    -229.4294 cm-1
2.      35.3037 cm-1
3.      55.1186 cm-1
```

M06-2X/def2tzvpp-IEFPCM(water) Molecular Geometry in Cartesian Coordinates

```
C         -0.842406        0.595473        0.172133
C          0.357153        1.066446       -0.437191
C          0.923976        2.363232        0.069435
C          1.072220        0.286406       -1.337977
O         -1.515518        1.205724        1.004713
O         -1.237599       -0.648710       -0.248475
C         -2.473270       -1.139797        0.262539
C         -3.656840       -0.579096       -0.500245
H          0.145404        3.110648        0.224818
H          1.446293        2.244224        1.026736
H          1.646327        2.765851       -0.642102
H          1.801131        0.779979       -1.966908
H          0.580051       -0.557834       -1.794535
H         -2.417454       -2.221099        0.146277
H         -2.549101       -0.904931        1.323050
H         -4.584404       -1.009752       -0.122300
H         -3.574671       -0.817737       -1.560536
H         -3.703780        0.502378       -0.384860
C          2.000888       -0.949944        1.350394
H          1.622431       -1.929240        1.640996
H          1.162647       -0.242790        1.354743
H          2.726538       -0.619856        2.092532
S          2.714576       -0.963267       -0.303896
```

8_ethylmethacrylate_TS_7_reopt

Datum	Value
M06-2X/def2tzvpp-IEFPCM(water) Energy	-823.296958
M06-2X/def2tzvpp-IEFPCM(water) Free Energy (Quasiharmonic)	-823.142366
Number of Imaginary Frequencies	1

Frequencies (Top 3 out of 63)

```
1.    -237.7303 cm-1
2.      37.5553 cm-1
3.      45.5261 cm-1
```

M06-2X/def2tzvpp-IEFPCM(water) Molecular Geometry in Cartesian Coordinates

```
C        -0.689340       1.039735       0.045878
C         0.618640       1.147468      -0.509066
C         1.484863       2.273024      -0.016537
C         1.140258       0.155386      -1.331443
O        -1.222060       1.848754       0.808448
O        -1.377201      -0.078879      -0.342248
C        -2.687877      -0.232642       0.190456
C        -3.241769      -1.538251      -0.331197
H         2.335217       2.418026      -0.684336
H         0.933725       3.212463       0.040833
H         1.889335       2.080901       0.984829
H         2.012045       0.397686      -1.924756
H         0.459369      -0.540757      -1.795202
H        -2.640947      -0.230604       1.280585
H        -3.309102       0.611303      -0.113859
H        -4.247382      -1.697779       0.056312
H        -3.289026      -1.529596      -1.419745
H        -2.615028      -2.373133      -0.018505
C         1.489782      -1.173747       1.440881
H         2.223143      -1.071139       2.239520
H         0.798921      -1.975571       1.699418
H         0.921259      -0.238323       1.377812
S         2.289702      -1.453020      -0.149146
```

8_ethylmethacrylate_TS_8

Datum	Value
M06-2X/def2tzvpp-IEFPCM(water) Energy	-823.291618
M06-2X/def2tzvpp-IEFPCM(water) Free Energy (Quasiharmonic)	-823.138217
Number of Imaginary Frequencies	1

Frequencies (Top 3 out of 63)

```
1.    -257.9744 cm-1
2.      31.2376 cm-1
3.      41.0854 cm-1
```

M06-2X/def2tzvpp-IEFPCM(water) Molecular Geometry in Cartesian Coordinates

```
C          -0.881701       -0.284614        0.412432
C           0.099659        0.745660        0.523856
C          -0.216277        2.150132        0.086880
C           1.394819        0.369399        0.842590
O          -0.732685       -1.487345        0.629138
O          -2.103423        0.186535        0.015669
C          -3.143658       -0.777158       -0.116223
C          -4.385496       -0.050399       -0.577579
H          -0.330505        2.236646       -0.999607
H          -1.140525        2.523761        0.529422
H           0.591139        2.821849        0.381631
H           1.549122       -0.601606        1.292430
H           2.094801        1.134553        1.154800
H          -2.841881       -1.541431       -0.833749
H          -3.306243       -1.271920        0.842496
H          -5.207260       -0.755846       -0.695260
H          -4.681805        0.706060        0.148483
H          -4.211177        0.438419       -1.535689
C           4.156642       -0.579903        0.190739
H           4.242845        0.179222        0.971442
H           5.103693       -0.618817       -0.345080
H           3.996832       -1.544191        0.673678
S           2.792868       -0.171183       -0.932476
```

8_ethylmethacrylate_TS_9_reopt2

Datum	Value
M06-2X/def2tzvpp-IEFPCM(water) Energy	-823.296624
M06-2X/def2tzvpp-IEFPCM(water) Free Energy (Quasiharmonic)	-823.141276
Number of Imaginary Frequencies	1

Frequencies (Top 3 out of 63)

```
1.    -226.8158 cm-1
2.      44.4284 cm-1
3.      55.3154 cm-1
```

M06-2X/def2tzvpp-IEFPCM(water) Molecular Geometry in Cartesian Coordinates

```
C         -0.755354       0.524530       0.551704
C          0.408123       1.205904       0.085987
C          0.420128       1.888974      -1.254880
C          1.579840       1.055104       0.816150
O         -0.867964      -0.141514       1.581767
O         -1.833456       0.684199      -0.282558
C         -3.015886      -0.038663       0.047978
C         -2.929791      -1.477581      -0.420651
H          0.427608       1.178016      -2.089376
H         -0.445703       2.535960      -1.399017
H          1.316597       2.503401      -1.349538
H          1.499202       0.697290       1.831382
H          2.391845       1.746771       0.637136
H         -3.187521       0.010845       1.121948
H         -3.822658       0.486311      -0.461112
H         -3.859364      -2.002229      -0.198902
H         -2.760238      -1.517347      -1.496692
H         -2.112006      -1.990840       0.083189
C          1.498656      -1.624145      -0.748183
H          0.625828      -0.961587      -0.799212
H          1.209370      -2.524910      -0.207610
H          1.774800      -1.898206      -1.765536
S          2.832456      -0.738105       0.076940
```

---

ethylcrotonate_1

Datum	Value
M06-2X/def2tzvpp-IEFPCM(water) Energy	-385.096208
M06-2X/def2tzvpp-IEFPCM(water) Free Energy (Quasiharmonic)	-384.974384
Number of Imaginary Frequencies	0

Frequencies (Top 3 out of 48)

```
1.      62.3410 cm-1
2.      89.9852 cm-1
3.     120.9564 cm-1
```

M06-2X/def2tzvpp-IEFPCM(water) Molecular Geometry in Cartesian Coordinates

```
C         -0.005849        0.380346       -0.000001
C          1.228916       -0.430574       -0.000002
C          2.424209        0.150112        0.000004
C          3.723952       -0.574926        0.000003
O         -0.056631        1.588425        0.000013
O         -1.094641       -0.394330       -0.000019
C         -2.361818        0.282173       -0.000023
C         -3.440869       -0.771633        0.000024
H          1.111378       -1.506289       -0.000010
H          2.458753        1.235305        0.000008
H          4.312208       -0.293002        0.875035
H          3.583743       -1.653843        0.000001
H          4.312208       -0.293000       -0.875029
H         -2.415690        0.919728        0.882068
H         -2.415713        0.919672       -0.882152
H         -4.418596       -0.292256        0.000019
H         -3.364684       -1.401061       -0.885397
H         -3.364664       -1.401005        0.885482
```

---

ethylcrotonate_2

Datum	Value
M06-2X/def2tzvpp-IEFPCM(water) Energy	-385.095703
M06-2X/def2tzvpp-IEFPCM(water) Free Energy (Quasiharmonic)	-384.973593
Number of Imaginary Frequencies	0

Frequencies (Top 3 out of 48)

```
1.      57.9966 cm-1
2.      90.3543 cm-1
3.     128.1822 cm-1
```

M06-2X/def2tzvpp-IEFPCM(water) Molecular Geometry in Cartesian Coordinates

```
C          0.103620        0.146018       -0.231043
C         -1.227323       -0.465125       -0.035237
C         -2.315458        0.291258        0.064474
C         -3.697361       -0.225497        0.261317
```

```
O          0.326256       1.330677      -0.323815
O          1.056769      -0.789590      -0.301271
C          2.409430      -0.333921      -0.473212
C          3.013473       0.087637       0.848354
H         -1.269473      -1.544852       0.022449
H         -2.190784       1.367735      -0.002424
H         -4.125184       0.183244       1.178433
H         -4.341763       0.102349      -0.556284
H         -3.717715      -1.311991       0.314493
H          2.934474      -1.187188      -0.894564
H          2.416477       0.481865      -1.193298
H          2.983354      -0.735596       1.560925
H          4.053345       0.376377       0.698944
H          2.474787       0.937138       1.264101
```

---

ethylcrotonate_3

Datum	Value
M06-2X/def2tzvpp-IEFPCM(water) Energy	-385.095551
M06-2X/def2tzvpp-IEFPCM(water) Free Energy (Quasiharmonic)	-384.973823
Number of Imaginary Frequencies	0

Frequencies (Top 3 out of 48)

```
1.      75.1575 cm-1
2.      86.7729 cm-1
3.     116.7384 cm-1
```

M06-2X/def2tzvpp-IEFPCM(water) Molecular Geometry in Cartesian Coordinates

```
C          0.040576       0.898475      -0.000000
C         -1.410827       0.632417      -0.000001
C         -1.944138      -0.585765       0.000001
C         -3.406939      -0.865248       0.000000
O          0.508672       2.014909      -0.000001
O          0.793492      -0.203223       0.000002
C          2.216138      -0.004415       0.000003
C          2.865173      -1.365943      -0.000003
H         -2.026988       1.522581      -0.000003
H         -1.279549      -1.442877       0.000002
H         -3.676715      -1.459000       0.875119
H         -3.992539       0.051714      -0.000002
H         -3.676714      -1.459003      -0.875118
```

```
H          2.486829       0.575030      -0.882230
H          2.486829       0.575022       0.882241
H          3.948286      -1.253144      -0.000001
H          2.576671      -1.930474       0.885479
H          2.576672      -1.930466      -0.885490
```

---

ethylcrotonate_4

Datum	Value
M06-2X/def2tzvpp-IEFPCM(water) Energy	-385.095037
M06-2X/def2tzvpp-IEFPCM(water) Free Energy (Quasiharmonic)	-384.972999
Number of Imaginary Frequencies	0

Frequencies (Top 3 out of 48)

```
1.      56.5065 cm-1
2.     108.5598 cm-1
3.     113.0448 cm-1
```

M06-2X/def2tzvpp-IEFPCM(water) Molecular Geometry in Cartesian Coordinates

```
C          0.151746       0.683455      -0.105173
C         -1.311470       0.639027       0.082298
C         -2.043215      -0.466339      -0.022715
C         -3.519350      -0.520060       0.167220
O          0.799001       1.700398       0.005118
O          0.691175      -0.498897      -0.412728
C          2.117466      -0.541731      -0.587284
C          2.826839      -0.639468       0.745288
H         -1.759092       1.595598       0.320300
H         -1.543367      -1.398591      -0.261882
H         -4.001397      -0.901777      -0.734484
H         -3.770388      -1.212809       0.972363
H         -3.931172       0.459400       0.401467
H          2.291653      -1.426259      -1.194441
H          2.430675       0.340019      -1.142506
H          2.491363      -1.519693       1.292127
H          3.900738      -0.723845       0.581810
H          2.637483       0.246643       1.348327
```

---

ethylcrotonate_5

Datum	Value
M06-2X/def2tzvpp-IEFPCM(water) Energy	-385.096208
M06-2X/def2tzvpp-IEFPCM(water) Free Energy (Quasiharmonic)	-384.974382
Number of Imaginary Frequencies	0

Frequencies (Top 3 out of 48)

```
1.      62.6219 cm-1
2.      90.2648 cm-1
3.     121.0773 cm-1
```

M06-2X/def2tzvpp-IEFPCM(water) Molecular Geometry in Cartesian Coordinates

```
C          0.005940        0.380069        0.000008
C         -1.229080       -0.430568        0.000041
C         -2.424312        0.150313        0.000022
C         -3.724213       -0.574642       -0.000042
O          0.057028        1.588103       -0.000115
O          1.094624       -0.394736        0.000141
C          2.361687        0.282223        0.000165
C          3.441119       -0.771291       -0.000174
H         -1.111714       -1.506299        0.000054
H         -2.458861        1.235505        0.000001
H         -4.312583       -0.292644        0.874885
H         -4.312249       -0.292883       -0.875269
H         -3.584103       -1.653592        0.000158
H          2.415122        0.919979       -0.881820
H          2.415290        0.919575        0.882433
H          4.418762       -0.291727       -0.000132
H          3.365207       -1.400954        0.885107
H          3.365070       -1.400523       -0.885750
```

---

ethylcrotonate_6

Datum	Value
M06-2X/def2tzvpp-IEFPCM(water) Energy	-385.087261
M06-2X/def2tzvpp-IEFPCM(water) Free Energy (Quasiharmonic)	-384.965131

Datum	Value
Number of Imaginary Frequencies	0

Frequencies (Top 3 out of 48)

```
1.      42.2302 cm-1
2.      88.2279 cm-1
3.     137.4599 cm-1
```

M06-2X/def2tzvpp-IEFPCM(water) Molecular Geometry in Cartesian Coordinates

```
C         -0.187096        0.970841       -0.004797
C          0.757037       -0.160441       -0.170066
C          2.053508        0.020038        0.069987
C          3.094108       -1.035055       -0.059568
O          0.160070        2.081063        0.324520
O         -1.491626        0.771046       -0.237133
C         -2.036563       -0.511111       -0.587120
C         -2.195784       -1.398682        0.627417
H          0.394667       -1.128714       -0.480836
H          2.380335        1.005718        0.384472
H          3.855410       -0.723826       -0.777140
H          3.605471       -1.176141        0.894355
H          2.671356       -1.984862       -0.379967
H         -3.005779       -0.276119       -1.019732
H         -1.432787       -0.974069       -1.365726
H         -2.820402       -0.907010        1.371778
H         -2.673645       -2.333425        0.336603
H         -1.233448       -1.631964        1.081982
```

---

ethylcrotonate_7

Datum	Value
M06-2X/def2tzvpp-IEFPCM(water) Energy	-385.088063
M06-2X/def2tzvpp-IEFPCM(water) Free Energy (Quasiharmonic)	-384.966662
Number of Imaginary Frequencies	0

Frequencies (Top 3 out of 48)

```
1.      28.8296 cm-1
2.     121.7028 cm-1
3.     125.3858 cm-1
```

M06-2X/def2tzvpp-IEFPCM(water) Molecular Geometry in Cartesian Coordinates

```
C          0.023271        0.852102       -0.000020
C         -0.951766       -0.263608       -0.000051
C         -2.256766       -0.002201        0.000034
C         -3.328237       -1.033721        0.000013
O         -0.295141        2.018334        0.000005
O          1.331209        0.560867       -0.000022
C          1.815198       -0.792273       -0.000014
C          3.322460       -0.723460        0.000040
H         -0.601054       -1.284828       -0.000141
H         -2.566473        1.037800        0.000126
H         -3.968906       -0.908044       -0.874679
H         -2.920385       -2.042251       -0.000086
H         -3.968810       -0.908179        0.874795
H          1.448086       -1.307220        0.887779
H          1.448151       -1.307207       -0.887843
H          3.733503       -1.731742        0.000046
H          3.681225       -0.201480       -0.885602
H          3.681162       -0.201496        0.885718
```

---

ethylcrotonate_HEI_10_reopt

Datum	Value
M06-2X/def2tzvpp-IEFPCM(water) Energy	-823.305967
M06-2X/def2tzvpp-IEFPCM(water) Free Energy (Quasiharmonic)	-823.150045
Number of Imaginary Frequencies	0

Frequencies (Top 3 out of 63)

```
1.      33.3109 cm-1
2.      53.4500 cm-1
3.      71.7888 cm-1
```

M06-2X/def2tzvpp-IEFPCM(water) Molecular Geometry in Cartesian Coordinates

```
C         -1.103431       -0.163512       -0.054099
C         -0.049611        0.603158       -0.502739
C          1.258119        0.608099        0.193281
C          1.978429        1.945949        0.100771
O         -1.168831       -0.892872        0.960901
O         -2.232331       -0.089782       -0.887749
C         -3.454731       -0.540882       -0.332269
C         -4.044811        0.472178        0.631761
H          1.125399        0.324859        1.239201
H          2.107089        2.230929       -0.944949
H          1.383439        2.717279        0.593391
H          2.961049        1.922229        0.572231
H         -4.122011       -0.692442       -1.181209
H         -3.308541       -1.498552        0.166351
H         -4.203991        1.428258        0.132241
H         -5.003231        0.121458        1.016491
H         -3.368801        0.624128        1.472541
C          3.786909       -0.687971        0.563151
H          3.475089       -0.651771        1.606771
H          4.355049       -1.601651        0.400621
H          4.424999        0.165889        0.345991
S          2.332379       -0.739241       -0.511899
H         -0.159541        1.176368       -1.413619
```

---

ethylcrotonate_HEI_11

Datum	Value
M06-2X/def2tzvpp-IEFPCM(water) Energy	-823.305113
M06-2X/def2tzvpp-IEFPCM(water) Free Energy (Quasiharmonic)	-823.148734
Number of Imaginary Frequencies	0

Frequencies (Top 3 out of 63)

```
1.     39.6797 cm-1
2.     52.3751 cm-1
3.     64.2216 cm-1
```

M06-2X/def2tzvpp-IEFPCM(water) Molecular Geometry in Cartesian Coordinates

```
C          0.928666       -0.908769       -0.145831
C          0.027810       -0.095538        0.509971
```

```
C          -1.381885       -0.518169        0.672021
C          -2.021624        0.039902        1.938101
O           0.709076       -2.032122       -0.658534
O           2.257469       -0.501902       -0.282430
C           2.667916        0.743626        0.242490
C           4.142204        0.912801       -0.052320
H          -1.446390       -1.607052        0.671829
H          -1.984753        1.131003        1.937818
H          -3.062714       -0.266455        2.035437
H          -1.469174       -0.305557        2.813615
H           2.089187        1.552714       -0.214155
H           2.485567        0.775053        1.321008
H           4.498212        1.865780        0.338207
H           4.719543        0.112999        0.411057
H           4.322929        0.891122       -1.126790
C          -2.302108        1.670970       -0.857690
H          -2.715373        2.016749       -1.802417
H          -1.243144        1.926466       -0.817086
H          -2.829957        2.160985       -0.041867
S          -2.485799       -0.120009       -0.796202
H           0.310618        0.869596        0.899836
```

---

ethylcrotonate_HEI_12_reopt

Datum	Value
M06-2X/def2tzvpp-IEFPCM(water) Energy	-823.306455
M06-2X/def2tzvpp-IEFPCM(water) Free Energy (Quasiharmonic)	-823.14988
Number of Imaginary Frequencies	0

Frequencies (Top 3 out of 63)

```
1.      42.2425 cm-1
2.      70.5028 cm-1
3.      90.4644 cm-1
```

M06-2X/def2tzvpp-IEFPCM(water) Molecular Geometry in Cartesian Coordinates

```
C           0.811451       -0.259370        0.777279
C          -0.005168        0.593260        0.062779
C          -1.437088        0.732571        0.402079
C          -1.951687        2.163551        0.289279
O           0.501801       -0.988880        1.750099
O           2.163811       -0.361861        0.436669
```

```
C           2.681502        0.406449       -0.629861
C           4.159492        0.106098       -0.750631
H          -1.614128        0.350221        1.406739
H          -1.423147        2.800351        0.999019
H          -1.769147        2.554071       -0.713771
H          -3.021887        2.230392        0.492039
H           2.520812        1.471629       -0.437381
H           2.162022        0.156729       -1.560191
H           4.679902        0.366437        0.170839
H           4.320811       -0.952902       -0.950921
H           4.595332        0.681667       -1.566871
C          -1.864440       -1.906899       -0.496511
H          -0.790789       -1.845289       -0.667461
H          -2.048660       -2.301219        0.500619
H          -2.317910       -2.563589       -1.235921
S          -2.583919       -0.269598       -0.692191
H           0.360212        1.157050       -0.782291
```

---

ethylcrotonate_HEI_14_reopt

Datum	Value
M06-2X/def2tzvpp-IEFPCM(water) Energy	-823.303614
M06-2X/def2tzvpp-IEFPCM(water) Free Energy (Quasiharmonic)	-823.148205
Number of Imaginary Frequencies	0

Frequencies (Top 3 out of 63)

```
1.      27.6040 cm-1
2.      46.5268 cm-1
3.      57.7844 cm-1
```

M06-2X/def2tzvpp-IEFPCM(water) Molecular Geometry in Cartesian Coordinates

```
C           0.948669        1.113304       -0.246348
C          -0.386129        1.191057        0.102179
C          -1.129353        0.141279        0.828508
C          -2.288193        0.705768        1.642986
O           1.645886        1.949058       -0.866512
O           1.569067       -0.065632        0.175193
C           2.942189       -0.205601       -0.140604
C           3.399406       -1.548507        0.385423
H          -0.465652       -0.426800        1.480301
H          -2.845118       -0.078260        2.154987
```

```
H         -1.908920      1.410941      2.384503
H         -2.979407      1.250689      0.997088
H          3.086702     -0.140947     -1.221023
H          3.518175      0.605234      0.310774
H          2.827150     -2.355432     -0.072463
H          4.454288     -1.702913      0.159337
H          3.267425     -1.604155      1.466018
C         -2.826648     -0.319102     -1.388640
H         -3.746016      0.016625     -0.912705
H         -3.079089     -0.962184     -2.228595
H         -2.265948      0.541554     -1.754029
S         -1.788647     -1.260132     -0.256793
H         -0.924522      2.071166     -0.225972
```

---

ethylcrotonate_HEI_1

Datum	Value
M06-2X/def2tzvpp-IEFPCM(water) Energy	-823.305634
M06-2X/def2tzvpp-IEFPCM(water) Free Energy (Quasiharmonic)	-823.14866
Number of Imaginary Frequencies	0

Frequencies (Top 3 out of 63)

```
1.     65.0236 cm-1
2.     80.0896 cm-1
3.     82.9878 cm-1
```

M06-2X/def2tzvpp-IEFPCM(water) Molecular Geometry in Cartesian Coordinates

```
C         -0.879636     -1.049409      0.397043
C          0.491532     -1.145731      0.528670
C          1.484577     -0.561220     -0.391153
C          2.718143     -1.436930     -0.584384
O         -1.765165     -1.500778      1.160427
O         -1.295206     -0.386165     -0.767021
C         -2.666053     -0.038275     -0.843298
C         -2.978252      1.204290     -0.029576
H          1.031898     -0.341681     -1.356314
H          3.467631     -0.955399     -1.214589
H          3.176490     -1.661617      0.380702
H          2.429596     -2.381270     -1.046756
H         -2.862854      0.140641     -1.900892
H         -3.282877     -0.873217     -0.513175
```

```
H         -4.033262        1.465802       -0.121611
H         -2.754702        1.026959        1.021965
H         -2.381657        2.048625       -0.375583
C          0.651203        2.030212        0.355011
H         -0.056122        1.442446        0.939859
H          0.210807        2.256763       -0.614276
H          0.877580        2.958000        0.876200
S          2.179596        1.100126        0.172996
H          0.857827       -1.650157        1.415404
```

---

ethylcrotonate_HEI_2_reopt

Datum	Value
M06-2X/def2tzvpp-IEFPCM(water) Energy	-823.305476
M06-2X/def2tzvpp-IEFPCM(water) Free Energy (Quasiharmonic)	-823.150072
Number of Imaginary Frequencies	0

Frequencies (Top 3 out of 63)

```
1.      30.0474 cm-1
2.      51.5710 cm-1
3.      60.5105 cm-1
```

M06-2X/def2tzvpp-IEFPCM(water) Molecular Geometry in Cartesian Coordinates

```
C         -0.788875       -1.131876       -0.487271
C          0.575301       -1.267076       -0.312869
C          1.398633       -0.439957        0.587050
C          2.559055       -1.202126        1.217113
O         -1.541915       -1.763372       -1.264653
O         -1.373991       -0.169254        0.341073
C         -2.738351        0.122367        0.104006
C         -3.143619        1.224185        1.058814
H          0.784293        0.008939        1.365334
H          3.195585       -0.553279        1.821037
H          2.172719       -1.999277        1.852817
H          3.175820       -1.659594        0.441148
H         -2.879065        0.436963       -0.933308
H         -3.349975       -0.769165        0.256605
H         -2.540166        2.116858        0.893602
H         -4.191723        1.484522        0.912156
H         -3.009774        0.905751        2.092716
C          0.783342        1.830841       -0.990897
```

```
H           0.134453        2.262853       -0.231562
H           0.223391        1.089929       -1.560704
H           1.136928        2.614549       -1.657539
S           2.220229        1.043015       -0.248034
H           1.078171       -1.984414       -0.950789
```

---

ethylcrotonate_HEI_3

Datum	Value
M06-2X/def2tzvpp-IEFPCM(water) Energy	-823.305034
M06-2X/def2tzvpp-IEFPCM(water) Free Energy (Quasiharmonic)	-823.148644
Number of Imaginary Frequencies	0

Frequencies (Top 3 out of 63)

```
   1.      32.5518 cm-1
   2.      62.4814 cm-1
   3.      68.6626 cm-1
```

M06-2X/def2tzvpp-IEFPCM(water) Molecular Geometry in Cartesian Coordinates

```
C           0.954281       -0.202177        0.832738
C          -0.297217       -0.784737        0.829590
C          -1.242034       -0.775047       -0.302116
C          -2.024135       -2.076246       -0.449610
O           1.793710       -0.173629        1.762992
O           1.293020        0.431579       -0.372006
C           2.660958        0.751344       -0.554897
C           3.475986       -0.468373       -0.944015
H          -0.720086       -0.549028       -1.230149
H          -2.765693       -2.020111       -1.248427
H          -1.337679       -2.894305       -0.669551
H          -2.542496       -2.312927        0.481571
H           2.681810        1.494009       -1.353093
H           3.063352        1.204470        0.350277
H           3.083712       -0.915033       -1.858085
H           4.517814       -0.194449       -1.114788
H           3.438875       -1.210561       -0.147349
C          -1.556700        2.016809        0.033250
H          -2.208969        2.845070        0.301749
H          -1.015203        2.261933       -0.878279
H          -0.843938        1.834635        0.836538
```

```
S          -2.577731        0.552890       -0.189506
H          -0.628485       -1.222998        1.764144
```

---

ethylcrotonate_HEI_4

Datum	Value
M06-2X/def2tzvpp-IEFPCM(water) Energy	-823.304432
M06-2X/def2tzvpp-IEFPCM(water) Free Energy (Quasiharmonic)	-823.148569
Number of Imaginary Frequencies	0

Frequencies (Top 3 out of 63)

```
1.      32.9298 cm-1
2.      41.1005 cm-1
3.      73.6421 cm-1
```

M06-2X/def2tzvpp-IEFPCM(water) Molecular Geometry in Cartesian Coordinates

```
C           1.229621        0.706873       -0.513107
C          -0.017060        1.225520       -0.227303
C          -1.107124        0.489875        0.458436
C          -2.015389        1.405585        1.268140
O           2.189146        1.262265       -1.096299
O           1.399707       -0.616272       -0.082872
C           2.721560       -1.125963       -0.099052
C           3.526935       -0.643718        1.093312
H          -0.700754       -0.292176        1.100524
H          -1.440224        1.879334        2.065931
H          -2.422907        2.191098        0.629186
H          -2.847692        0.868608        1.723370
H           2.614865       -2.210867       -0.072330
H           3.216716       -0.849853       -1.029116
H           3.036972       -0.927262        2.025286
H           4.525392       -1.082700        1.082915
H           3.624337        0.440858        1.063604
C          -3.290111       -1.355626        0.210146
H          -4.074998       -0.715713        0.607442
H          -2.767517       -1.842510        1.033306
H          -3.744252       -2.121442       -0.415436
S          -2.112127       -0.434004       -0.808327
H          -0.227327        2.223467       -0.591522
```

ethylcrotonate_HEI_5_reopt

Datum	Value
M06-2X/def2tzvpp-IEFPCM(water) Energy	-823.306753
M06-2X/def2tzvpp-IEFPCM(water) Free Energy (Quasiharmonic)	-823.150531
Number of Imaginary Frequencies	0

Frequencies (Top 3 out of 63)

```
1.      43.8838 cm-1
2.      57.4572 cm-1
3.      75.8027 cm-1
```

M06-2X/def2tzvpp-IEFPCM(water) Molecular Geometry in Cartesian Coordinates

```
C         -0.880384       -0.653758        0.261636
C          0.296865       -0.898316       -0.412076
C          1.611412       -0.695674        0.222122
C          2.637962       -1.769344       -0.122601
O         -1.052322       -0.242944        1.431463
O         -2.022143       -0.957159       -0.503253
C         -3.236697       -0.370520       -0.070919
C         -3.301649        1.105606       -0.418991
H          1.488393       -0.629422        1.303064
H          3.616203       -1.555308        0.311448
H          2.297307       -2.735982        0.249234
H          2.754440       -1.847160       -1.205245
H         -4.025400       -0.919044       -0.586700
H         -3.362873       -0.513436        1.002041
H         -4.259013        1.530397       -0.114842
H         -2.505190        1.644685        0.093934
H         -3.186635        1.250534       -1.493676
C          1.147730        2.090259        0.165720
H          1.048727        2.204902        1.243517
H          0.206922        1.726964       -0.247401
H          1.398602        3.051217       -0.278161
S          2.450998        0.922230       -0.250312
H          0.256831       -1.212723       -1.447251
```

ethylcrotonate_HEI_6

Datum	Value
M06-2X/def2tzvpp-IEFPCM(water) Energy	-823.306617
M06-2X/def2tzvpp-IEFPCM(water) Free Energy (Quasiharmonic)	-823.151889
Number of Imaginary Frequencies	0

Frequencies (Top 3 out of 63)

```
1.      30.8699 cm-1
2.      43.3354 cm-1
3.      57.3077 cm-1
```

M06-2X/def2tzvpp-IEFPCM(water) Molecular Geometry in Cartesian Coordinates

```
C          0.823678       -0.205695        0.359394
C         -0.256282       -0.757949       -0.299698
C         -1.611335       -0.750394        0.275389
C         -2.392504       -2.037274        0.031195
O          0.872757        0.336714        1.486566
O          2.015639       -0.302344       -0.372281
C          3.179860        0.212379        0.245840
C          4.339917       -0.008645       -0.700333
H         -1.556049       -0.543354        1.344198
H         -1.889406       -2.871249        0.521063
H         -3.413900       -1.975565        0.410921
H         -2.438369       -2.253434       -1.037923
H          3.054585        1.275977        0.461508
H          3.356012       -0.291616        1.198734
H          4.168914        0.505887       -1.645956
H          5.261670        0.374080       -0.262492
H          4.471987       -1.070871       -0.906121
C         -1.707722        2.050834       -0.099584
H         -1.680571        2.302774        0.958854
H         -0.697086        1.841381       -0.448944
H         -2.123280        2.886756       -0.658081
S         -2.735153        0.605616       -0.401907
H         -0.112902       -1.165117       -1.292740
```

---

ethylcrotonate_HEI_7

Datum	Value
M06-2X/def2tzvpp-IEFPCM(water) Energy	-823.306541

Datum	Value
M06-2X/def2tzvpp-IEFPCM(water) Free Energy (Quasiharmonic)	-823.149716
Number of Imaginary Frequencies	0

Frequencies (Top 3 out of 63)

```
1.      42.0942 cm-1
2.      72.5037 cm-1
3.      81.2184 cm-1
```

M06-2X/def2tzvpp-IEFPCM(water) Molecular Geometry in Cartesian Coordinates

```
C          0.905540       0.081508       0.039042
C         -0.169801      -0.599580      -0.490426
C         -1.412432      -0.808878       0.273166
C         -2.030701      -2.189220       0.078382
O          1.051609       0.561871       1.185758
O          1.958024       0.241778      -0.880324
C          3.223170       0.570661      -0.335006
C          3.900861      -0.638784       0.282783
H         -1.224436      -0.629901       1.331836
H         -2.980817      -2.290771       0.605818
H         -2.207042      -2.377636      -0.982376
H         -1.345657      -2.953475       0.446169
H          3.811362       0.952054      -1.170222
H          3.117321       1.364237       0.404092
H          4.887918      -0.372586       0.662803
H          3.302784      -1.019462       1.110332
H          4.019254      -1.430906      -0.457084
C         -1.949645       1.957022       0.062476
H         -1.807193       2.181487       1.117961
H         -0.980477       1.904573      -0.432582
H         -2.552619       2.739222      -0.393488
S         -2.799653       0.387714      -0.164409
H         -0.124965      -0.935831      -1.518691
```

---

ethylcrotonate_HEI_8

Datum	Value
M06-2X/def2tzvpp-IEFPCM(water) Energy	-823.305817
M06-2X/def2tzvpp-IEFPCM(water) Free Energy (Quasiharmonic)	-823.15029

Datum	Value
Number of Imaginary Frequencies	0

Frequencies (Top 3 out of 63)

```
1.      28.1445 cm-1
2.      46.9717 cm-1
3.      55.4236 cm-1
```

M06-2X/def2tzvpp-IEFPCM(water) Molecular Geometry in Cartesian Coordinates

```
C          -1.135004       -0.472706        0.319501
C          -0.021000       -0.888428       -0.375305
C           1.347565       -0.675866        0.152261
C           2.313216       -1.792693       -0.219380
O          -1.200108        0.131211        1.414137
O          -2.341620       -0.828168       -0.309988
C          -3.491463       -0.104349        0.090847
C          -3.512307        1.290621       -0.507227
H           1.314793       -0.553578        1.236599
H           2.342781       -1.917795       -1.303248
H           3.327372       -1.598948        0.130474
H           1.975113       -2.731690        0.222887
H          -4.342013       -0.685482       -0.266580
H          -3.542302       -0.052532        1.177994
H          -3.481456        1.240892       -1.595995
H          -4.418468        1.821098       -0.212359
H          -2.649968        1.858819       -0.158998
C           3.570293        1.114051        0.393814
H           3.901167        2.146034        0.296146
H           3.436407        0.889122        1.451718
H           4.333190        0.459542       -0.022060
S           1.990701        0.953206       -0.473933
H          -0.141809       -1.374902       -1.333914
```

---

ethylcrotonate_HEI_9_reopt

Datum	Value
M06-2X/def2tzvpp-IEFPCM(water) Energy	-823.306125
M06-2X/def2tzvpp-IEFPCM(water) Free Energy (Quasiharmonic)	-823.150664
Number of Imaginary Frequencies	0

Frequencies (Top 3 out of 63)

```
1.      44.4110 cm-1
2.      48.1958 cm-1
3.      64.6996 cm-1
```

M06-2X/def2tzvpp-IEFPCM(water) Molecular Geometry in Cartesian Coordinates

```
C          1.023152       -0.004562        0.306510
C         -0.008208        0.773791       -0.176525
C         -1.398108        0.600561        0.303849
C         -2.205596        1.890552        0.267037
O          0.985312       -0.910438        1.169346
O          2.263212        0.302852       -0.266955
C          3.376858       -0.428545        0.211074
C          4.600689        0.055558       -0.535568
H         -1.400101        0.187000        1.314668
H         -2.204015        2.305025       -0.742694
H         -3.240989        1.741223        0.573706
H         -1.754862        2.624891        0.937385
H          3.494208       -0.277500        1.286809
H          3.223230       -1.497955        0.051092
H          5.485918       -0.483060       -0.197874
H          4.487142       -0.107452       -1.607354
H          4.760054        1.120234       -0.364933
C         -3.829137       -0.903886        0.133816
H         -3.661881       -0.990741        1.207314
H         -4.288618       -1.823647       -0.222416
H         -4.505797       -0.074643       -0.060923
S         -2.245836       -0.704411       -0.719316
H          0.192997        1.507076       -0.946003
```

---

ethylcrotonate_TS_10_UNCON_m062x

Datum	Value
M06-2X/def2tzvpp-IEFPCM(water) Energy	-823.295752
M06-2X/def2tzvpp-IEFPCM(water) Free Energy (Quasiharmonic)	-823.139981
Number of Imaginary Frequencies	1

Frequencies (Top 3 out of 63)

```
   1.    -175.0856 cm-1
   2.      36.8416 cm-1
   3.      62.3980 cm-1
```

M06-2X/def2tzvpp-IEFPCM(water) Molecular Geometry in Cartesian Coordinates

```
 C          0.820003        0.204286        0.900024
 C         -0.005793        0.866258       -0.068900
 C         -1.313028        1.159377        0.233330
 C         -2.107029        2.120710       -0.599619
 O          0.472796       -0.113708        2.031230
 O          2.107889       -0.107369        0.573120
 C          2.624683        0.179513       -0.724468
 C          4.056627       -0.300311       -0.760970
 H         -1.603485        1.070484        1.270330
 H         -1.835681        3.144347       -0.324473
 H         -1.893516        1.989499       -1.659942
 H         -3.176638        2.000887       -0.442722
 H          2.570581        1.253339       -0.913524
 H          2.024600       -0.333674       -1.479028
 H          4.488667       -0.101557       -1.740893
 H          4.652561        0.214918       -0.008705
 H          4.106079       -1.371829       -0.571482
 C         -1.360815       -2.022611       -0.441838
 H         -1.485003       -2.618465       -1.345475
 H         -1.260768       -2.699303        0.406914
 H         -0.422742       -1.460676       -0.539955
 S         -2.716644       -0.851919       -0.228139
 H          0.368276        1.068037       -1.060978
```

---

ethylcrotonate_TS_11_UNCON_m062x_reopt

Datum	Value
M06-2X/def2tzvpp-IEFPCM(water) Energy	-823.295752
M06-2X/def2tzvpp-IEFPCM(water) Free Energy (Quasiharmonic)	-823.139982
Number of Imaginary Frequencies	1

Frequencies (Top 3 out of 63)

```
   1.    -175.1679 cm-1
   2.      36.8576 cm-1
   3.      62.4158 cm-1
```

M06-2X/def2tzvpp-IEFPCM(water) Molecular Geometry in Cartesian Coordinates

```
C          0.820027        0.204374        0.899973
C         -0.005811        0.866153       -0.069013
C         -1.313092        1.159233        0.233248
C         -2.107047        2.120701       -0.599599
O          0.472869       -0.113459        2.031251
O          2.107929       -0.107287        0.573104
C          2.624702        0.179437       -0.724521
C          4.056655       -0.300365       -0.760967
H         -1.603416        1.070454        1.270297
H         -1.835595        3.144292       -0.324384
H         -1.893595        1.989555       -1.659942
H         -3.176664        2.000970       -0.442666
H          2.570573        1.253237       -0.913718
H          2.024624       -0.333859       -1.479014
H          4.488693       -0.101737       -1.740916
H          4.652582        0.214972       -0.008770
H          4.106123       -1.371857       -0.571331
C         -1.360934       -2.022763       -0.441525
H         -1.484885       -2.618452       -1.345304
H         -1.261359       -2.699592        0.407169
H         -0.422733       -1.460964       -0.539179
S         -2.716622       -0.851847       -0.228222
H          0.368221        1.067885       -1.061114
```

---

ethylcrotonate_TS_12_UNCON_m062x_reopt

Datum	Value
M06-2X/def2tzvpp-IEFPCM(water) Energy	-823.294376
M06-2X/def2tzvpp-IEFPCM(water) Free Energy (Quasiharmonic)	-823.138582
Number of Imaginary Frequencies	1

Frequencies (Top 3 out of 63)

```
1.    -182.5303 cm-1
2.      38.0472 cm-1
3.      48.3092 cm-1
```

M06-2X/def2tzvpp-IEFPCM(water) Molecular Geometry in Cartesian Coordinates

```
C          0.901368      -0.374582       0.844322
C          0.223690       0.638598       0.089497
C         -0.999934       1.104353       0.512344
C         -1.570969       2.380784      -0.031310
O          0.504458      -0.853166       1.901127
O          2.088268      -0.878382       0.391710
C          2.669261      -0.453010      -0.839454
C          3.434955       0.846297      -0.691946
H         -1.295544       0.840858       1.517728
H         -1.085282       3.229906       0.458744
H         -1.393668       2.464308      -1.103099
H         -2.641186       2.452637       0.150166
H          1.903878      -0.389172      -1.613391
H          3.347941      -1.258153      -1.116230
H          3.905413       1.108596      -1.639458
H          2.777074       1.661681      -0.395120
H          4.214833       0.737878       0.061198
C         -1.708036      -1.747879      -0.846020
H         -0.669546      -1.391728      -0.854053
H         -1.951237      -2.081971      -1.854006
H         -1.766124      -2.604198      -0.174091
S         -2.776261      -0.392584      -0.322582
H          0.629818       0.995722      -0.844367
```

---

ethylcrotonate_TS_1_UNCON_m062x

Datum	Value
M06-2X/def2tzvpp-IEFPCM(water) Energy	-823.298556
M06-2X/def2tzvpp-IEFPCM(water) Free Energy (Quasiharmonic)	-823.142803
Number of Imaginary Frequencies	1

Frequencies (Top 3 out of 63)

```
1.    -208.4252 cm-1
2.      35.0037 cm-1
3.      52.7533 cm-1
```

M06-2X/def2tzvpp-IEFPCM(water) Molecular Geometry in Cartesian Coordinates

```
C          -0.921355       -1.010606        0.433737
C           0.479507       -1.248238        0.544244
C           1.407937       -0.906711       -0.424753
C           2.766347       -1.552458       -0.420401
O          -1.771656       -1.305846        1.272043
O          -1.288390       -0.415510       -0.740743
C          -2.667170       -0.086914       -0.895884
C          -3.006269        1.217416       -0.203435
H           1.016942       -0.668083       -1.403324
H           3.473013       -1.010644       -1.046779
H           3.168475       -1.602803        0.591190
H           2.684820       -2.573481       -0.803609
H          -3.281328       -0.901260       -0.515490
H          -2.819625       -0.005477       -1.970825
H          -4.057005        1.462647       -0.359686
H          -2.823231        1.135840        0.866963
H          -2.399171        2.029627       -0.602532
C           0.758204        1.946892        0.801144
H           0.189934        1.086394        1.175203
H           0.088416        2.566665        0.205951
H           1.095219        2.525000        1.660643
S           2.152253        1.327417       -0.158992
H           0.804661       -1.668541        1.487852
```

---

ethylcrotonate_TS_2_UNCON_m062x

Datum	Value
M06-2X/def2tzvpp-IEFPCM(water) Energy	-823.298791
M06-2X/def2tzvpp-IEFPCM(water) Free Energy (Quasiharmonic)	-823.143494
Number of Imaginary Frequencies	1

Frequencies (Top 3 out of 63)

```
1.    -209.7581 cm-1
2.      46.8149 cm-1
3.      59.3251 cm-1
```

M06-2X/def2tzvpp-IEFPCM(water) Molecular Geometry in Cartesian Coordinates

```
C          -0.853290       -1.079014        0.537818
C           0.526132       -1.357938        0.316910
C           1.284397       -0.775247       -0.686461
```

```
C          2.588929      -1.403403      -1.094755
O         -1.565235      -1.573953       1.410855
O         -1.378770      -0.172517      -0.336910
C         -2.745130       0.180310      -0.144393
C         -3.097043       1.220125      -1.182445
H          0.737464      -0.297484      -1.486383
H          3.199972      -0.720377      -1.682367
H          3.160538      -1.714121      -0.220414
H          2.390687      -2.290999      -1.702045
H         -2.879716       0.567792       0.866648
H         -3.370014      -0.708510      -0.243476
H         -4.137315       1.522843      -1.068690
H         -2.466462       2.101917      -1.070851
H         -2.961563       0.822428      -2.187760
C          1.002752       1.640180       1.338744
H          0.531248       0.691355       1.620979
H          0.217901       2.343078       1.059805
H          1.524308       2.028880       2.212285
S          2.148417       1.318144      -0.015214
H          0.989835      -2.015438       1.041630
```

---

ethylcrotonate_TS_3_UNCON_m062x_reopt

Datum	Value
M06-2X/def2tzvpp-IEFPCM(water) Energy	-823.298097
M06-2X/def2tzvpp-IEFPCM(water) Free Energy (Quasiharmonic)	-823.142494
Number of Imaginary Frequencies	1

Frequencies (Top 3 out of 63)

```
1.    -209.3744 cm-1
2.      38.5523 cm-1
3.      56.2011 cm-1
```

M06-2X/def2tzvpp-IEFPCM(water) Molecular Geometry in Cartesian Coordinates

```
C          0.977688       0.355824       0.738795
C         -0.296065       0.994541       0.757329
C         -1.151942       1.059736      -0.329865
C         -2.252920       2.084339      -0.353356
O          1.762869       0.282339       1.682680
O          1.296169      -0.202482      -0.467379
C          2.582971      -0.805882      -0.582680
```

```
C           3.659787       0.225270       -0.853676
H          -0.722957       0.826182       -1.293673
H          -2.999167       1.858074       -1.113009
H          -2.750553       2.141308        0.614495
H          -1.829123       3.067987       -0.574532
H           2.494773      -1.500799       -1.416142
H           2.802879      -1.369859        0.322240
H           4.623427      -0.266518       -0.987888
H           3.430979       0.785681       -1.759961
H           3.737503       0.920180       -0.019278
C          -1.541982      -1.952348        0.575198
H          -0.834624      -1.336086        1.143068
H          -0.974695      -2.675542       -0.010202
H          -2.162965      -2.493199        1.288021
S          -2.534332      -0.859458       -0.459061
H          -0.603691       1.386177        1.718957
```

---

ethylcrotonate_TS_4_UNCON_m062x

Datum	Value
M06-2X/def2tzvpp-IEFPCM(water) Energy	-823.29963
M06-2X/def2tzvpp-IEFPCM(water) Free Energy (Quasiharmonic)	-823.144172
Number of Imaginary Frequencies	1

Frequencies (Top 3 out of 63)

```
  1.    -207.8675 cm-1
  2.      34.4622 cm-1
  3.      50.7735 cm-1
```

M06-2X/def2tzvpp-IEFPCM(water) Molecular Geometry in Cartesian Coordinates

```
C          -0.868902      -0.729322        0.218718
C           0.346663      -1.075907       -0.436898
C           1.556889      -1.010617        0.232321
C           2.761965      -1.728676       -0.308623
O          -1.009619      -0.381000        1.387969
O          -1.956300      -0.830702       -0.609977
C          -3.208965      -0.398490       -0.084229
C          -3.342994       1.109800       -0.150375
H           1.493658      -0.960661        1.310924
H           2.665571      -2.799921       -0.110311
H           2.844097      -1.591352       -1.386553
```

```
H        3.682865      -1.379742       0.155090
H       -3.319060      -0.754562       0.938807
H       -3.960204      -0.882130      -0.706301
H       -4.322767       1.416986       0.215786
H       -2.578090       1.584173       0.462762
H       -3.235333       1.456677      -1.178055
C        0.959296       2.048354      -0.393212
H        0.498294       2.637251       0.399590
H        0.232673       1.298222      -0.731222
H        1.177391       2.705866      -1.233693
S        2.438661       1.185831       0.166718
H        0.305967      -1.301332      -1.494464
```

---

ethylcrotonate_TS_5_UNCON_m062x

Datum	Value
M06-2X/def2tzvpp-IEFPCM(water) Energy	-823.299725
M06-2X/def2tzvpp-IEFPCM(water) Free Energy (Quasiharmonic)	-823.144422
Number of Imaginary Frequencies	1

Frequencies (Top 3 out of 63)

```
1.    -200.9380 cm-1
2.      45.8099 cm-1
3.      67.8415 cm-1
```

M06-2X/def2tzvpp-IEFPCM(water) Molecular Geometry in Cartesian Coordinates

```
C       -0.827004      -0.389511       0.379558
C        0.263275      -0.936306      -0.356068
C        1.514302      -1.065205       0.221478
C        2.536470      -1.988580      -0.380786
O       -0.819275       0.000682       1.544292
O       -1.979272      -0.329951      -0.355606
C       -3.125209       0.199431       0.304918
C       -4.271490       0.179908      -0.679069
H        1.541761      -0.982745       1.299709
H        2.559934      -1.885915      -1.465419
H        3.535271      -1.793903       0.005889
H        2.275304      -3.023894      -0.143997
H       -2.911501       1.213687       0.645472
H       -3.348082      -0.404002       1.186128
H       -5.170598       0.577803      -0.210003
```

```
H          -4.039942       0.789144       -1.552201
H          -4.477122      -0.837357       -1.010756
C           1.397996       2.045409       -0.418559
H           1.108042       2.692029        0.409253
H           0.532722       1.429799       -0.694870
H           1.652872       2.668694       -1.274620
S           2.756052       0.943380        0.016657
H           0.102844      -1.174137       -1.399424
```

---

ethylcrotonate_TS_6_UNCON_m062x

Datum	Value
M06-2X/def2tzvpp-IEFPCM(water) Energy	-823.299209
M06-2X/def2tzvpp-IEFPCM(water) Free Energy (Quasiharmonic)	-823.143711
Number of Imaginary Frequencies	1

Frequencies (Top 3 out of 63)

```
1.    -202.2296 cm-1
2.      35.9166 cm-1
3.      50.0899 cm-1
```

M06-2X/def2tzvpp-IEFPCM(water) Molecular Geometry in Cartesian Coordinates

```
C           0.920147       0.159769        0.064137
C          -0.196744       0.831469       -0.511285
C          -1.325021       1.106066        0.241279
C          -2.307370       2.147561       -0.217347
O           1.034007      -0.235945        1.220895
O           1.936648      -0.025721       -0.835670
C           3.130166      -0.634367       -0.347541
C           4.026007       0.371711        0.346676
H          -1.204798       1.021053        1.312977
H          -2.502323       2.052990       -1.285278
H          -3.254282       2.072530        0.314223
H          -1.892629       3.143154       -0.035601
H           3.620323      -1.043906       -1.229246
H           2.873429      -1.453259        0.322483
H           4.951539      -0.108605        0.664762
H           4.277827       1.188330       -0.329706
H           3.528285       0.781330        1.224003
C          -1.684542      -1.989964       -0.385498
H          -0.798186      -1.478091       -0.781137
```

```
H          -2.134649       -2.557576       -1.198809
H          -1.361293       -2.686624        0.387549
S          -2.817599       -0.740552        0.248653
H          -0.162759        1.057362       -1.568995
```

---

ethylcrotonate_TS_7_UNCON_m062x

Datum	Value
M06-2X/def2tzvpp-IEFPCM(water) Energy	-823.294503
M06-2X/def2tzvpp-IEFPCM(water) Free Energy (Quasiharmonic)	-823.139634
Number of Imaginary Frequencies	1

Frequencies (Top 3 out of 63)

```
1.    -244.7558 cm-1
2.      41.5160 cm-1
3.      57.9074 cm-1
```

M06-2X/def2tzvpp-IEFPCM(water) Molecular Geometry in Cartesian Coordinates

```
C           1.190146        0.437151        0.292141
C           0.076968        1.113936       -0.282241
C          -1.203757        0.920649        0.200965
C          -2.290482        1.890499       -0.169844
O           1.179361       -0.340534        1.241864
O           2.366870        0.750570       -0.334902
C           3.546785        0.108777        0.143604
C           3.700952       -1.280316       -0.442083
H          -1.286067        0.470646        1.183738
H          -2.137488        2.825321        0.377766
H          -2.256091        2.117647       -1.235034
H          -3.280585        1.513458        0.075084
H           3.525285        0.071988        1.231590
H           4.366766        0.753148       -0.169188
H           3.723455       -1.234264       -1.530704
H           4.632234       -1.730482       -0.097831
H           2.872563       -1.915689       -0.133611
C          -3.601978       -1.094446        0.555893
H          -3.334335       -0.510805        1.441827
H          -3.825589       -2.108551        0.884812
H          -4.509918       -0.663828        0.133742
S          -2.226423       -1.072950       -0.627995
H           0.250892        1.720823       -1.160590
```

ethylcrotonate_TS_8_UNCON_m062x

Datum	Value
M06-2X/def2tzvpp-IEFPCM(water) Energy	-823.294976
M06-2X/def2tzvpp-IEFPCM(water) Free Energy (Quasiharmonic)	-823.140441
Number of Imaginary Frequencies	1

Frequencies (Top 3 out of 63)

```
1.    -244.0623 cm-1
2.      27.8887 cm-1
3.      65.6806 cm-1
```

M06-2X/def2tzvpp-IEFPCM(water) Molecular Geometry in Cartesian Coordinates

```
C          1.101814        0.054690        0.337928
C          0.089042        0.941408       -0.126156
C         -1.213634        0.836682        0.324864
C         -2.167023        1.977677        0.105373
O          0.981933       -0.851565        1.157436
O          2.318817        0.305503       -0.233365
C          3.401835       -0.514164        0.197256
C          4.639964       -0.064389       -0.542558
H         -1.367100        0.259257        1.229445
H         -1.913287        2.791534        0.791239
H         -2.082374        2.359440       -0.911756
H         -3.200554        1.692928        0.287189
H          3.526003       -0.413996        1.276608
H          3.172148       -1.559812       -0.012250
H          5.494138       -0.668328       -0.238620
H          4.506535       -0.172993       -1.618423
H          4.861065        0.979907       -0.324164
C         -3.859728       -0.885398        0.353060
H         -3.548326       -0.485704        1.322736
H         -4.228156       -1.897385        0.516632
H         -4.686736       -0.275284       -0.010166
S         -2.452691       -0.872950       -0.793521
H          0.350083        1.647083       -0.903298
```

ethylcrotonate_TS_9_UNCON_m062x

Datum	Value
M06-2X/def2tzvpp-IEFPCM(water) Energy	-823.294536
M06-2X/def2tzvpp-IEFPCM(water) Free Energy (Quasiharmonic)	-823.139461
Number of Imaginary Frequencies	1

Frequencies (Top 3 out of 63)

```
1.    -249.3636 cm-1
2.      49.4722 cm-1
3.      57.4567 cm-1
```

M06-2X/def2tzvpp-IEFPCM(water) Molecular Geometry in Cartesian Coordinates

```
C          1.172701        0.024252       -0.029577
C          0.115476        0.920727       -0.353435
C         -1.115164        0.830125        0.271335
C         -2.073538        1.985329        0.193060
O          1.157400       -0.874350        0.806867
O          2.295990        0.254899       -0.779194
C          3.443071       -0.540828       -0.489578
C          4.213038        0.002793        0.697093
H         -1.148613        0.250176        1.186500
H         -1.716714        2.789360        0.843831
H         -2.124021        2.375764       -0.823027
H         -3.076223        1.711789        0.512866
H          4.049307       -0.503432       -1.393140
H          3.136696       -1.571036       -0.315688
H          3.598929       -0.030450        1.595373
H          5.109417       -0.593996        0.866786
H          4.515996        1.033709        0.514998
C         -3.739957       -0.870870        0.674416
H         -3.281176       -0.490696        1.591949
H         -4.090527       -1.882451        0.875218
H         -4.605544       -0.247558        0.450134
S         -2.522719       -0.851098       -0.671653
H          0.275090        1.622834       -1.160606
```

---

n-propylacrylate_10

Datum	Value

Datum	Value
M06-2X/def2tzvpp-IEFPCM(water) Energy	-385.087549
M06-2X/def2tzvpp-IEFPCM(water) Free Energy (Quasiharmonic)	-384.964642
Number of Imaginary Frequencies	0

Frequencies (Top 3 out of 48)

```
1.      40.4598 cm-1
2.      82.5976 cm-1
3.     109.8485 cm-1
```

M06-2X/def2tzvpp-IEFPCM(water) Molecular Geometry in Cartesian Coordinates

```
C          -0.845880        0.537374       -0.243512
C          -2.192323        0.218306        0.290927
C          -2.638915       -1.014935        0.480115
O          -0.450649        1.671718       -0.373987
O          -0.136692       -0.545876       -0.559278
C           1.205605       -0.373423       -1.048135
C           2.206149       -0.662527        0.052879
C           2.103631        0.305497        1.224095
H          -2.790025        1.088540        0.525629
H          -3.628547       -1.192013        0.878165
H          -2.028897       -1.875888        0.243824
H           1.305874       -1.083067       -1.866258
H           1.316677        0.638805       -1.431139
H           2.064834       -1.688816        0.397138
H           3.201489       -0.611797       -0.393001
H           2.251687        1.333801        0.893272
H           2.852540        0.077369        1.981263
H           1.123493        0.244586        1.699012
```

---

n-propylacrylate_11

Datum	Value
M06-2X/def2tzvpp-IEFPCM(water) Energy	-385.079724
M06-2X/def2tzvpp-IEFPCM(water) Free Energy (Quasiharmonic)	-384.956632
Number of Imaginary Frequencies	0

Frequencies (Top 3 out of 48)

```
1.      38.0769 cm-1
2.      75.8175 cm-1
3.     103.1965 cm-1
```

M06-2X/def2tzvpp-IEFPCM(water) Molecular Geometry in Cartesian Coordinates

```
C          0.961983       -0.615594        0.084011
C          1.450336        0.788797        0.188927
C          2.599476        1.124848       -0.380141
O          1.619971       -1.498690       -0.410062
O         -0.246117       -0.924630        0.565897
C         -1.160555        0.059159        1.079769
C         -1.829655        0.847889       -0.029001
C         -2.602221       -0.041899       -0.994507
H          0.867620        1.522432        0.723651
H          2.978367        2.135351       -0.319291
H          3.182502        0.391746       -0.921572
H         -1.898267       -0.530367        1.621042
H         -0.654559        0.700068        1.799539
H         -1.081675        1.430343       -0.571279
H         -2.501836        1.565184        0.445703
H         -3.091789        0.553771       -1.763241
H         -1.938551       -0.750585       -1.489177
H         -3.368825       -0.610589       -0.466402
```

---

n-propylacrylate_12

Datum	Value
M06-2X/def2tzvpp-IEFPCM(water) Energy	-385.080544
M06-2X/def2tzvpp-IEFPCM(water) Free Energy (Quasiharmonic)	-384.958206
Number of Imaginary Frequencies	0

Frequencies (Top 3 out of 48)

```
1.      21.0228 cm-1
2.      81.4392 cm-1
3.     110.4477 cm-1
```

M06-2X/def2tzvpp-IEFPCM(water) Molecular Geometry in Cartesian Coordinates

```
C          1.059935       0.515707      -0.114407
C          1.814912      -0.745845       0.126298
C          3.126430      -0.699206       0.314041
O          1.588379       1.599787      -0.162446
O         -0.263217       0.447112      -0.287176
C         -0.983671      -0.797046      -0.241592
C         -2.448912      -0.478735      -0.438934
C         -3.024567       0.376012       0.682300
H          1.288541      -1.687486       0.144198
H          3.698165      -1.599623       0.489553
H          3.653686       0.245375       0.295352
H         -0.822394      -1.271389       0.728092
H         -0.617334      -1.453477      -1.030812
H         -2.980932      -1.429533      -0.500686
H         -2.572587       0.018826      -1.402443
H         -2.917510      -0.127235       1.644468
H         -2.511930       1.335113       0.744131
H         -4.083765       0.568915       0.518888
```

---

n-propylacrylate_13

Datum	Value
M06-2X/def2tzvpp-IEFPCM(water) Energy	-385.079839
M06-2X/def2tzvpp-IEFPCM(water) Free Energy (Quasiharmonic)	-384.956947
Number of Imaginary Frequencies	0

Frequencies (Top 3 out of 48)

```
1.      40.8245 cm-1
2.      69.3681 cm-1
3.      81.9272 cm-1
```

M06-2X/def2tzvpp-IEFPCM(water) Molecular Geometry in Cartesian Coordinates

```
C          1.222904      -0.549258      -0.015991
C          1.245744       0.922285       0.222640
C          2.367374       1.599321       0.020238
O          2.191828      -1.163774      -0.390193
O          0.090861      -1.227692       0.198411
C         -1.132445      -0.596058       0.604984
C         -1.850653       0.053608      -0.560364
```

```
C        -3.165229     0.675199    -0.106651
H         0.352810     1.428950     0.553864
H         2.411960     2.666826     0.183959
H         3.264089     1.095411    -0.314941
H        -0.942972     0.108635     1.414694
H        -1.731446    -1.408427     1.012014
H        -1.210133     0.814277    -1.011494
H        -2.029045    -0.705637    -1.323314
H        -3.692172     1.125389    -0.945847
H        -3.818603    -0.076685     0.337421
H        -2.992186     1.452403     0.638762
```

---

n-propylacrylate_14

Datum	Value
M06-2X/def2tzvpp-IEFPCM(water) Energy	-385.080361
M06-2X/def2tzvpp-IEFPCM(water) Free Energy (Quasiharmonic)	-384.958587
Number of Imaginary Frequencies	0

Frequencies (Top 3 out of 48)

```
1.      10.9342 cm-1
2.      79.7609 cm-1
3.     127.4168 cm-1
```

M06-2X/def2tzvpp-IEFPCM(water) Molecular Geometry in Cartesian Coordinates

```
C         1.266279    -0.531540     0.000003
C         1.705502     0.891795     0.000007
C         3.000161     1.177090    -0.000008
O         2.039039    -1.458490    -0.000002
O        -0.043571    -0.798380     0.000005
C        -1.039355     0.237007     0.000005
C        -2.393824    -0.434287    -0.000005
C        -3.512798     0.599060    -0.000004
H         0.967036     1.678376     0.000024
H         3.347031     2.200852    -0.000005
H         3.739151     0.386797    -0.000024
H        -0.919603     0.858490    -0.888988
H        -0.919611     0.858480     0.889007
H        -2.468110    -1.076391    -0.878868
H        -2.468117    -1.076402     0.878850
H        -4.487294     0.114240    -0.000010
```

```
H          -3.455005        1.237883        0.882036
H          -3.454998        1.237892       -0.882036
```

n-propylacrylate_15

Datum	Value
M06-2X/def2tzvpp-IEFPCM(water) Energy	-385.078628
M06-2X/def2tzvpp-IEFPCM(water) Free Energy (Quasiharmonic)	-384.955611
Number of Imaginary Frequencies	0

Frequencies (Top 3 out of 48)

```
1.      31.0134 cm-1
2.      66.5485 cm-1
3.     124.7589 cm-1
```

M06-2X/def2tzvpp-IEFPCM(water) Molecular Geometry in Cartesian Coordinates

```
C          -0.977091       -0.589510       -0.163726
C          -1.245325        0.801965        0.297730
C          -2.491586        1.252525        0.325256
O          -1.793280       -1.239041       -0.771356
O           0.210143       -1.148199        0.086520
C           1.231560       -0.570877        0.917466
C           2.364931       -0.032724        0.067593
C           1.978875        1.167051       -0.786589
H          -0.424579        1.436806        0.591256
H          -2.713792        2.261019        0.644626
H          -3.316142        0.616995        0.030752
H           1.579256       -1.392420        1.540388
H           0.811380        0.188424        1.574002
H           3.180597        0.233221        0.742392
H           2.729342       -0.844165       -0.565005
H           2.811186        1.472744       -1.418604
H           1.137499        0.934361       -1.441652
H           1.702160        2.020366       -0.165838
```

n-propylacrylate_1

Datum	Value
M06-2X/def2tzvpp-IEFPCM(water) Energy	-385.088513
M06-2X/def2tzvpp-IEFPCM(water) Free Energy (Quasiharmonic)	-384.965766
Number of Imaginary Frequencies	0

Frequencies (Top 3 out of 48)

```
1.      49.4875 cm-1
2.      68.8495 cm-1
3.      98.9218 cm-1
```

M06-2X/def2tzvpp-IEFPCM(water) Molecular Geometry in Cartesian Coordinates

```
C        -0.908008      -0.263968       0.108678
C        -2.044021       0.674965       0.287464
C        -3.184052       0.484614      -0.359113
O        -0.921081      -1.242771      -0.597781
O         0.142149       0.110992       0.841345
C         1.330272      -0.695549       0.747979
C         2.154874      -0.326202      -0.469950
C         2.609635       1.127488      -0.454427
H        -1.888105       1.506218       0.960895
H        -4.019445       1.159856      -0.239275
H        -3.302141      -0.359450      -1.026422
H         1.875363      -0.483917       1.665925
H         1.041687      -1.744485       0.731355
H         3.020125      -0.991787      -0.488064
H         1.573811      -0.536726      -1.368834
H         1.755089       1.803826      -0.448651
H         3.212965       1.356397      -1.331722
H         3.209909       1.336206       0.432498
```

---

n-propylacrylate_2

Datum	Value
M06-2X/def2tzvpp-IEFPCM(water) Energy	-385.089061
M06-2X/def2tzvpp-IEFPCM(water) Free Energy (Quasiharmonic)	-384.966519
Number of Imaginary Frequencies	0

Frequencies (Top 3 out of 48)

```
1.      52.5424 cm-1
2.      71.0189 cm-1
3.     107.4987 cm-1
```

M06-2X/def2tzvpp-IEFPCM(water) Molecular Geometry in Cartesian Coordinates

```
C          1.053463        0.267226        0.009646
C          2.037396       -0.826592       -0.187264
C          3.326182       -0.624048        0.040564
O          1.319618        1.387292        0.373660
O         -0.184214       -0.140734       -0.268551
C         -1.230917        0.833060       -0.116331
C         -2.543061        0.160360       -0.447986
C         -2.888977       -0.968070        0.514899
H          1.647718       -1.778546       -0.520147
H          4.053442       -1.411483       -0.098105
H          3.679692        0.343314        0.373565
H         -1.216964        1.198041        0.911920
H         -1.022701        1.671143       -0.780988
H         -2.500739       -0.211677       -1.473346
H         -3.317634        0.928650       -0.421294
H         -3.848767       -1.416262        0.262087
H         -2.132476       -1.751489        0.485514
H         -2.949315       -0.595763        1.538745
```

---

n-propylacrylate_3

Datum	Value
M06-2X/def2tzvpp-IEFPCM(water) Energy	-385.08853
M06-2X/def2tzvpp-IEFPCM(water) Free Energy (Quasiharmonic)	-384.965702
Number of Imaginary Frequencies	0

Frequencies (Top 3 out of 48)

```
1.      58.9574 cm-1
2.      73.3697 cm-1
3.      98.4617 cm-1
```

M06-2X/def2tzvpp-IEFPCM(water) Molecular Geometry in Cartesian Coordinates

```
C          1.019649       0.033078      -0.179198
C          2.339683      -0.503558       0.236909
C          3.392904       0.294116       0.328398
O          0.796768       1.184639      -0.464368
O          0.097213      -0.931093      -0.204931
C         -1.238190      -0.550040      -0.573466
C         -1.998333       0.019808       0.607394
C         -3.426300       0.371872       0.210122
H          2.389095      -1.560895       0.456717
H          4.360283      -0.082047       0.629866
H          3.304340       1.348618       0.100731
H         -1.702232      -1.467740      -0.929236
H         -1.189738       0.167222      -1.392018
H         -1.998681      -0.715989       1.413246
H         -1.476844       0.905682       0.971752
H         -3.979698       0.771049       1.058355
H         -3.436744       1.122058      -0.581514
H         -3.958113      -0.507988      -0.154458
```

---

n-propylacrylate_4

Datum	Value
M06-2X/def2tzvpp-IEFPCM(water) Energy	-385.088906
M06-2X/def2tzvpp-IEFPCM(water) Free Energy (Quasiharmonic)	-384.96649
Number of Imaginary Frequencies	0

Frequencies (Top 3 out of 48)

```
 1.     53.4974 cm-1
 2.     80.7653 cm-1
 3.    105.3513 cm-1
```

M06-2X/def2tzvpp-IEFPCM(water) Molecular Geometry in Cartesian Coordinates

```
C          1.161988       0.199749       0.000002
C          2.327519      -0.718988       0.000001
C          3.562610      -0.241129      -0.000005
O          1.224169       1.405438      -0.000002
O          0.013326      -0.476915       0.000007
C         -1.190790       0.305012       0.000008
C         -2.366335      -0.643938      -0.000005
```

```
C         -3.682266       0.123411       -0.000004
H          2.113030      -1.778603        0.000005
H          4.420988      -0.897916       -0.000006
H          3.737946       0.827000       -0.000009
H         -1.193175       0.945762        0.883074
H         -1.193167       0.945778       -0.883047
H         -2.303040      -1.287716        0.878713
H         -2.303033      -1.287703       -0.878733
H         -4.530008      -0.559391       -0.000013
H         -3.762930       0.759947        0.881940
H         -3.762923       0.759961       -0.881938
```

---

n-propylacrylate_5

Datum	Value
M06-2X/def2tzvpp-IEFPCM(water) Energy	-385.088173
M06-2X/def2tzvpp-IEFPCM(water) Free Energy (Quasiharmonic)	-384.965056
Number of Imaginary Frequencies	0

Frequencies (Top 3 out of 48)

```
1.      56.1421 cm-1
2.      70.2291 cm-1
3.     105.4688 cm-1
```

M06-2X/def2tzvpp-IEFPCM(water) Molecular Geometry in Cartesian Coordinates

```
C         -0.830504      -0.179995        0.244940
C         -2.051396      -0.272455       -0.595648
C         -3.136815       0.420580       -0.286518
O         -0.725443       0.496374        1.238629
O          0.140390      -0.952648       -0.247684
C          1.414785      -0.969656        0.420571
C          2.416633      -0.123537       -0.339491
C          2.041683       1.351754       -0.384880
H         -2.002263      -0.923824       -1.457059
H         -4.032185       0.363740       -0.889269
H         -3.148316       1.063494        0.584200
H          1.716006      -2.014616        0.437377
H          1.285177      -0.613146        1.440252
H          3.384700      -0.252094        0.148695
H          2.516635      -0.520022       -1.351652
H          2.794287       1.925865       -0.923596
```

```
H           1.087160        1.498959       -0.891895
H           1.952902        1.761689        0.621540
```

---

n-propylacrylate_6

Datum	Value
M06-2X/def2tzvpp-IEFPCM(water) Energy	-385.087967
M06-2X/def2tzvpp-IEFPCM(water) Free Energy (Quasiharmonic)	-384.965037
Number of Imaginary Frequencies	0

Frequencies (Top 3 out of 48)

```
1.      49.0213 cm-1
2.      62.2427 cm-1
3.     123.5831 cm-1
```

M06-2X/def2tzvpp-IEFPCM(water) Molecular Geometry in Cartesian Coordinates

```
C          -0.952743       -0.598466       -0.068811
C          -2.235620        0.103973       -0.311777
C          -2.494669        1.332542        0.112169
O          -0.719410       -1.702720       -0.501791
O          -0.095811        0.103388        0.671188
C           1.194578       -0.483684        0.916947
C           2.134025       -0.262460       -0.252465
C           2.355568        1.213627       -0.556797
H          -2.953272       -0.471892       -0.880140
H          -3.445096        1.803714       -0.097142
H          -1.765138        1.897513        0.676144
H           1.557414        0.023593        1.808905
H           1.064610       -1.542602        1.130569
H           1.739000       -0.779258       -1.128213
H           3.081777       -0.742682       -0.001039
H           3.049456        1.340895       -1.386458
H           1.418405        1.701528       -0.824548
H           2.767771        1.730656        0.311158
```

---

n-propylacrylate_7

Datum	Value
M06-2X/def2tzvpp-IEFPCM(water) Energy	-385.08856
M06-2X/def2tzvpp-IEFPCM(water) Free Energy (Quasiharmonic)	-384.966042
Number of Imaginary Frequencies	0

Frequencies (Top 3 out of 48)

```
1.      49.4234 cm-1
2.      71.1791 cm-1
3.     111.8923 cm-1
```

M06-2X/def2tzvpp-IEFPCM(water) Molecular Geometry in Cartesian Coordinates

```
C          1.189306       -0.572939        0.071623
C          2.315637        0.390670        0.055736
C          2.170142        1.688041       -0.172079
O          1.333410       -1.749568        0.309414
O          0.010125       -0.019365       -0.198606
C         -1.129716       -0.894825       -0.188242
C         -2.353851       -0.061715       -0.491012
C         -2.620762        1.001804        0.566385
H          3.281071       -0.054646        0.253874
H          3.023460        2.352078       -0.168833
H          1.197037        2.118387       -0.366261
H         -0.974539       -1.676235       -0.931638
H         -1.197771       -1.363292        0.794961
H         -3.202242       -0.744510       -0.561727
H         -2.234363        0.398744       -1.473504
H         -3.521688        1.567079        0.332334
H         -2.754312        0.544427        1.547982
H         -1.789468        1.703210        0.631884
```

---

n-propylacrylate_8

Datum	Value
M06-2X/def2tzvpp-IEFPCM(water) Energy	-385.087927
M06-2X/def2tzvpp-IEFPCM(water) Free Energy (Quasiharmonic)	-384.965053
Number of Imaginary Frequencies	0

Frequencies (Top 3 out of 48)

```
1.      56.7943 cm-1
2.      76.5565 cm-1
3.     107.6175 cm-1
```

M06-2X/def2tzvpp-IEFPCM(water) Molecular Geometry in Cartesian Coordinates

```
C         -1.034349        0.533367        0.090749
C         -2.451013        0.234256       -0.227549
C         -2.963997       -0.987617       -0.231868
O         -0.583984        1.655015        0.077950
O         -0.318148       -0.548390        0.394949
C          1.072215       -0.349370        0.697064
C          1.908259       -0.258067       -0.563525
C          3.383991       -0.097121       -0.220829
H         -3.043509        1.107935       -0.462663
H         -4.005165       -1.152025       -0.472855
H         -2.358601       -1.852003        0.004017
H          1.355060       -1.217097        1.289685
H          1.179071        0.548468        1.304767
H          1.752118       -1.161584       -1.155236
H          1.563237        0.588043       -1.158907
H          3.553174        0.814118        0.354174
H          3.741003       -0.939611        0.373051
H          3.990032       -0.041943       -1.123474
```

---

n-propylacrylate_9

Datum	Value
M06-2X/def2tzvpp-IEFPCM(water) Energy	-385.088297
M06-2X/def2tzvpp-IEFPCM(water) Free Energy (Quasiharmonic)	-384.966106
Number of Imaginary Frequencies	0

Frequencies (Top 3 out of 48)

```
1.      55.6198 cm-1
2.      71.0846 cm-1
3.     111.5755 cm-1
```

M06-2X/def2tzvpp-IEFPCM(water) Molecular Geometry in Cartesian Coordinates

```
C          1.247984       0.591030      -0.000002
C          2.556125      -0.105558      -0.000011
C          2.689170      -1.424019       0.000004
O          1.144938       1.795548      -0.000006
O          0.202370      -0.232993       0.000011
C         -1.095715       0.380185       0.000023
C         -2.132429      -0.718794      -0.000015
C         -3.539649      -0.135426      -0.000006
H          3.409360       0.559006      -0.000029
H          3.667952      -1.883452      -0.000003
H          1.826428      -2.076032       0.000022
H         -1.185327       1.014304      -0.883215
H         -1.185335       1.014249       0.883300
H         -1.983070      -1.348162      -0.878777
H         -1.983081      -1.348213       0.878713
H         -4.288035      -0.925874      -0.000035
H         -3.705143       0.484584       0.881892
H         -3.705131       0.484637      -0.881870
```

---

n-propylacrylate_HEI_10_reopt

Datum	Value
M06-2X/def2tzvpp-IEFPCM(water) Energy	-823.302968
M06-2X/def2tzvpp-IEFPCM(water) Free Energy (Quasiharmonic)	-823.147333
Number of Imaginary Frequencies	0

Frequencies (Top 3 out of 63)

```
1.      26.2349 cm-1
2.      40.6139 cm-1
3.      52.3465 cm-1
```

M06-2X/def2tzvpp-IEFPCM(water) Molecular Geometry in Cartesian Coordinates

```
C          0.599963       1.538777       0.124672
C         -0.697565       1.575364       0.597748
C         -1.520195       0.371257       0.847810
O          1.372152       2.495303      -0.115924
O          1.080474       0.245033      -0.094576
C          2.443334       0.126159      -0.454970
C          2.770012      -1.349337      -0.577820
```

```
C          2.599800      -2.103867       0.734755
H         -2.240988       0.521159       1.653428
H         -0.909912      -0.498420       1.089194
H          2.631902       0.644543      -1.397514
H          3.075414       0.592643       0.306624
H          2.134047      -1.790219      -1.349089
H          3.800644      -1.437388      -0.928554
H          1.566863      -2.053920       1.077861
H          2.871753      -3.153655       0.627193
H          3.232326      -1.670433       1.511929
S         -2.533044      -0.114320      -0.627844
C         -3.414322      -1.541101       0.043052
H         -4.020185      -1.249459       0.899950
H         -2.712897      -2.317678       0.345029
H         -4.066698      -1.936443      -0.732450
H         -1.140746       2.552182       0.734431
```

---

n-propylacrylate_HEI_11

Datum	Value
M06-2X/def2tzvpp-IEFPCM(water) Energy	-823.30297
M06-2X/def2tzvpp-IEFPCM(water) Free Energy (Quasiharmonic)	-823.145789
Number of Imaginary Frequencies	0

Frequencies (Top 3 out of 63)

```
1.      35.6856 cm-1
2.      55.8024 cm-1
3.      72.6429 cm-1
```

M06-2X/def2tzvpp-IEFPCM(water) Molecular Geometry in Cartesian Coordinates

```
C         -0.418929      -0.733407       0.679414
C          0.676265      -1.342126       0.096788
C          1.491866      -0.753356      -0.981632
O         -1.139452      -1.164140       1.608352
O         -0.722162       0.522747       0.132009
C         -1.962946       1.119425       0.463335
C         -2.972270       0.940907      -0.660153
C         -3.318159      -0.521297      -0.910063
H          1.857697      -1.497001      -1.691504
H          0.942255       0.002522      -1.538637
H         -2.340651       0.688991       1.389945
```

```
H          -1.767119        2.181566        0.624561
H          -3.873520        1.504846       -0.405951
H          -2.565380        1.387964       -1.570599
H          -2.417535       -1.093489       -1.137987
H          -4.009134       -0.627578       -1.746405
H          -3.778805       -0.966883       -0.027634
S           3.088957        0.067601       -0.452545
C           2.426674        1.305967        0.673391
H           3.256246        1.741161        1.226138
H           1.900358        2.089121        0.130981
H           1.738033        0.822555        1.365221
H           0.992161       -2.280922        0.533219
```

---

n-propylacrylate_HEI_12

Datum	Value
M06-2X/def2tzvpp-IEFPCM(water) Energy	-823.305326
M06-2X/def2tzvpp-IEFPCM(water) Free Energy (Quasiharmonic)	-823.148069
Number of Imaginary Frequencies	0

Frequencies (Top 3 out of 63)

```
1.      30.8490 cm-1
2.      50.0767 cm-1
3.      76.7432 cm-1
```

M06-2X/def2tzvpp-IEFPCM(water) Molecular Geometry in Cartesian Coordinates

```
C          -0.385781       -1.096610        0.065437
C           0.685745       -1.049878       -0.799334
C           2.072774       -1.081433       -0.302025
O          -0.394333       -1.199779        1.313979
O          -1.626309       -1.059584       -0.597664
C          -2.719802       -0.585356        0.167690
C          -2.612695        0.902404        0.468589
C          -2.477743        1.747144       -0.791770
H           2.750939       -1.666298       -0.925628
H           2.112914       -1.478525        0.711589
H          -3.603595       -0.779389       -0.443797
H          -2.808435       -1.152718        1.094641
H          -3.500354        1.200956        1.031952
H          -1.750945        1.060599        1.119894
H          -1.575699        1.472064       -1.338711
```

```
H          -2.419079        2.809180       -0.553493
H          -3.331551        1.597424       -1.455796
S           2.939243        0.571382       -0.242989
C           1.787684        1.466789        0.811783
H           1.841056        1.112256        1.839534
H           2.045888        2.522841        0.778626
H           0.776456        1.321918        0.430822
H           0.498558       -0.905880       -1.854535
```

---

n-propylacrylate_HEI_13

Datum	Value
M06-2X/def2tzvpp-IEFPCM(water) Energy	-823.304846
M06-2X/def2tzvpp-IEFPCM(water) Free Energy (Quasiharmonic)	-823.14802
Number of Imaginary Frequencies	0

Frequencies (Top 3 out of 63)

```
 1.      36.6770 cm-1
 2.      48.4137 cm-1
 3.      78.1101 cm-1
```

M06-2X/def2tzvpp-IEFPCM(water) Molecular Geometry in Cartesian Coordinates

```
C           0.317767        0.179245        0.103605
C          -0.540318       -0.850430       -0.218834
C          -1.759274       -1.105119        0.570874
O           0.285054        0.959448        1.082436
O           1.351750        0.345851       -0.835939
C           2.513449        1.011172       -0.373362
C           3.371290        0.115773        0.507400
C           3.851091       -1.133729       -0.219880
H          -1.981630       -2.165602        0.699552
H          -1.682417       -0.651171        1.558330
H           3.067327        1.290543       -1.272323
H           2.237734        1.919272        0.162272
H           4.225957        0.698210        0.860473
H           2.788026       -0.160593        1.388326
H           3.003135       -1.723244       -0.568657
H           4.458238       -1.764685        0.429059
H           4.454107       -0.867843       -1.090367
S          -3.352033       -0.467638       -0.164210
C          -2.938165        1.280893       -0.278896
```

```
H          -2.914893        1.744063        0.705962
H          -1.961278        1.381736       -0.750386
H          -3.692241        1.770394       -0.891047
H          -0.359006       -1.418094       -1.121252
```

---

n-propylacrylate_HEI_14

Datum	Value
M06-2X/def2tzvpp-IEFPCM(water) Energy	-823.304755
M06-2X/def2tzvpp-IEFPCM(water) Free Energy (Quasiharmonic)	-823.14879
Number of Imaginary Frequencies	0

Frequencies (Top 3 out of 63)

```
1.      29.2539 cm-1
2.      41.9626 cm-1
3.      69.4278 cm-1
```

M06-2X/def2tzvpp-IEFPCM(water) Molecular Geometry in Cartesian Coordinates

```
C           0.184943       -0.744058        0.058877
C          -0.782679       -0.621263       -0.918618
C          -2.191724       -0.943677       -0.636767
O           0.069578       -1.149620        1.237648
O           1.459775       -0.364789       -0.379550
C           2.515359       -0.500990        0.552244
C           3.789930       -0.004908       -0.102373
C           3.737226        1.476979       -0.451845
H          -2.699012       -1.450137       -1.459560
H          -2.273582       -1.569176        0.251434
H           2.301015        0.080703        1.453606
H           2.619074       -1.545548        0.856096
H           3.980408       -0.596448       -1.000949
H           4.617351       -0.196665        0.584327
H           4.672329        1.812795       -0.899505
H           2.931603        1.679650       -1.156460
H           3.558401        2.076223        0.443002
S          -3.325323        0.514816       -0.339256
C          -2.474773        1.262547        1.060137
H          -2.628426        0.684419        1.969702
H          -2.864160        2.268176        1.201635
H          -1.409686        1.309782        0.834817
H          -0.504668       -0.223341       -1.884777
```

n-propylacrylate_HEI_15

Datum	Value
M06-2X/def2tzvpp-IEFPCM(water) Energy	-823.304838
M06-2X/def2tzvpp-IEFPCM(water) Free Energy (Quasiharmonic)	-823.147063
Number of Imaginary Frequencies	0

Frequencies (Top 3 out of 63)

```
1.      49.3756 cm-1
2.      65.8180 cm-1
3.      88.5353 cm-1
```

M06-2X/def2tzvpp-IEFPCM(water) Molecular Geometry in Cartesian Coordinates

```
C          0.403403      -0.941866       0.218284
C         -0.706690      -1.185870      -0.563593
C         -2.066486      -1.152496       0.004484
O          0.461897      -0.656880       1.434532
O          1.608349      -1.083477      -0.495246
C          2.807092      -0.650694       0.121730
C          3.245777       0.699692      -0.423515
C          2.234710       1.800879      -0.133234
H         -2.717774      -1.938225      -0.382233
H         -2.035516      -1.241131       1.090127
H          2.665325      -0.599202       1.200828
H          3.565846      -1.405637      -0.095077
H          4.215734       0.949417       0.014289
H          3.398475       0.610954      -1.502002
H          1.262877       1.544967      -0.558148
H          2.550747       2.753519      -0.558364
H          2.101013       1.932703       0.941312
S         -3.071778       0.380700      -0.350655
C         -2.001296       1.606102       0.419687
H         -0.974543       1.411333       0.111585
H         -2.306089       2.593660       0.081002
H         -2.068227       1.559130       1.505407
H         -0.570441      -1.394319      -1.615592
```

n-propylacrylate_HEI_16_reopt

Datum	Value
M06-2X/def2tzvpp-IEFPCM(water) Energy	-823.305058
M06-2X/def2tzvpp-IEFPCM(water) Free Energy (Quasiharmonic)	-823.148299
Number of Imaginary Frequencies	0

Frequencies (Top 3 out of 63)

```
1.      30.7961 cm-1
2.      49.3253 cm-1
3.      78.2586 cm-1
```

M06-2X/def2tzvpp-IEFPCM(water) Molecular Geometry in Cartesian Coordinates

```
C          0.184504      -0.996457       0.225922
C         -0.988638      -1.219416      -0.461953
C         -2.300894      -0.986131       0.167884
O          0.350290      -0.598766       1.401306
O          1.330169      -1.309825      -0.530166
C          2.545340      -0.738816      -0.085491
C          2.648384       0.737090      -0.432897
C          3.973891       1.328518       0.028571
H         -3.051953      -1.732268      -0.096910
H         -2.211584      -0.963432       1.253380
H          2.659417      -0.875515       0.991186
H          3.338290      -1.296451      -0.588272
H          2.536680       0.854348      -1.513317
H          1.814132       1.260685       0.038674
H          4.083096       1.239187       1.110678
H          4.051861       2.384076      -0.229174
H          4.814975       0.808557      -0.433680
S         -3.158742       0.602151      -0.308833
C         -1.904229       1.779467       0.221607
H         -2.139573       2.749250      -0.210966
H         -0.933410       1.440722      -0.140084
H         -1.878042       1.864163       1.306614
H         -0.937844      -1.524544      -1.497780
```

---

n-propylacrylate_HEI_17_reopt

Datum	Value

Datum	Value
M06-2X/def2tzvpp-IEFPCM(water) Energy	-823.30476
M06-2X/def2tzvpp-IEFPCM(water) Free Energy (Quasiharmonic)	-823.148158
Number of Imaginary Frequencies	0

Frequencies (Top 3 out of 63)

```
1.      39.1238 cm-1
2.      48.0679 cm-1
3.      79.3053 cm-1
```

M06-2X/def2tzvpp-IEFPCM(water) Molecular Geometry in Cartesian Coordinates

```
C        -0.192830      -0.043859       0.480773
C         0.709375      -1.005726       0.072148
C         2.074372      -1.054142       0.623422
O        -0.054799       0.853624       1.342595
O        -1.424292      -0.130360      -0.181525
C        -2.430893       0.772545       0.233611
C        -3.679851       0.495732      -0.580321
C        -4.249411      -0.896075      -0.336783
H         2.447970      -2.068169       0.774254
H         2.128576      -0.525993       1.574969
H        -2.097509       1.802909       0.090575
H        -2.639069       0.642181       1.300079
H        -3.448688       0.627295      -1.639984
H        -4.423561       1.252905      -0.322248
H        -3.527569      -1.663703      -0.613279
H        -5.157857      -1.059969      -0.915997
H        -4.494431      -1.031023       0.718481
S         3.417515      -0.310359      -0.444516
C         2.772251       1.364236      -0.585976
H         3.326816       1.880895      -1.366083
H         1.718429       1.311581      -0.857104
H         2.877656       1.905524       0.352592
H         0.423649      -1.691071      -0.713805
```

---

n-propylacrylate_HEI_18

Datum	Value
M06-2X/def2tzvpp-IEFPCM(water) Energy	-823.304797

Datum	Value
M06-2X/def2tzvpp-IEFPCM(water) Free Energy (Quasiharmonic)	-823.148557
Number of Imaginary Frequencies	0

Frequencies (Top 3 out of 63)

```
1.      36.6580 cm-1
2.      63.5029 cm-1
3.      74.7329 cm-1
```

M06-2X/def2tzvpp-IEFPCM(water) Molecular Geometry in Cartesian Coordinates

```
C         -0.090500       -0.443199        0.370556
C          0.954265       -1.093834       -0.254676
C          2.308502       -1.088453        0.324182
O         -0.097371        0.180569        1.455993
O         -1.295382       -0.532779       -0.340344
C         -2.418458        0.101372        0.237560
C         -3.611603       -0.121790       -0.669336
C         -4.865621        0.537495       -0.109617
H          2.840744       -2.032748        0.198873
H          2.274303       -0.854763        1.387712
H         -2.617810       -0.305489        1.233045
H         -2.232195        1.172605        0.359437
H         -3.385227        0.278810       -1.659759
H         -3.771400       -1.195473       -0.788891
H         -4.724074        1.613881       -0.000201
H         -5.722770        0.377516       -0.762318
H         -5.112669        0.133102        0.873232
S          3.517755        0.127589       -0.423170
C          2.602504        1.652777       -0.145652
H          1.579352        1.517188       -0.494429
H          3.081630        2.446753       -0.714055
H          2.591932        1.917758        0.909996
H          0.781605       -1.559107       -1.215206
```

---

n-propylacrylate_HEI_19_reopt

Datum	Value
M06-2X/def2tzvpp-IEFPCM(water) Energy	-823.304782
M06-2X/def2tzvpp-IEFPCM(water) Free Energy (Quasiharmonic)	-823.149138

Datum	Value
Number of Imaginary Frequencies	0

Frequencies (Top 3 out of 63)

```
1.      10.0902 cm-1
2.      45.8621 cm-1
3.      62.3465 cm-1
```

M06-2X/def2tzvpp-IEFPCM(water) Molecular Geometry in Cartesian Coordinates

```
C         -0.224058      -0.110744       0.051919
C          0.805215      -0.968736       0.376603
C          1.984906      -1.115632      -0.495016
O         -0.376776       0.591037      -0.973374
O         -1.214167      -0.044336       1.050081
C         -2.459643       0.503927       0.665369
C         -3.311687      -0.483806      -0.114483
C         -4.651922       0.125453      -0.505271
H          2.356954      -2.139773      -0.552916
H          1.765722      -0.776276      -1.506727
H         -2.964840       0.778324       1.593929
H         -2.308811       1.409387       0.075789
H         -2.759644      -0.788986      -1.005386
H         -3.465367      -1.377617       0.494470
H         -5.210423       0.439037       0.378599
H         -5.268733      -0.584287      -1.055255
H         -4.509282       1.004323      -1.136258
S          3.515217      -0.184356       0.033741
C          2.846159       1.487058       0.036558
H          3.560284       2.140573       0.532466
H          2.673987       1.841841      -0.978019
H          1.904972       1.488322       0.585048
H          0.775433      -1.483890       1.326675
```

---

n-propylacrylate_HEI_1_reopt

Datum	Value
M06-2X/def2tzvpp-IEFPCM(water) Energy	-823.30311
M06-2X/def2tzvpp-IEFPCM(water) Free Energy (Quasiharmonic)	-823.146099
Number of Imaginary Frequencies	0

Frequencies (Top 3 out of 63)

```
1.      44.4926 cm-1
2.      61.0683 cm-1
3.      75.2642 cm-1
```

M06-2X/def2tzvpp-IEFPCM(water) Molecular Geometry in Cartesian Coordinates

```
C          0.339511       -1.392043       -0.230036
C         -1.034891       -1.525844       -0.190364
C         -1.935091       -0.793218        0.719190
O          1.152425       -2.003627       -0.962388
O          0.854109       -0.467903        0.690745
C          2.266047       -0.368968        0.771394
C          2.845836        0.586905       -0.259437
C          2.388995        2.024615       -0.050719
H         -2.753546       -1.409617        1.095270
H         -1.401129       -0.384852        1.575144
H          2.713579       -1.356112        0.663837
H          2.475808        0.002756        1.777514
H          2.557424        0.233227       -1.250423
H          3.936001        0.529989       -0.201197
H          1.302374        2.090781       -0.094842
H          2.799562        2.688779       -0.811260
H          2.707924        2.394530        0.925806
S         -2.865324        0.646346       -0.028082
C         -1.460663        1.671114       -0.489133
H         -0.991260        2.104032        0.392717
H         -0.732514        1.054754       -1.017079
H         -1.810337        2.467683       -1.142021
H         -1.469435       -2.200617       -0.916370
```

---

n-propylacrylate_HEI_20_reopt

Datum	Value
M06-2X/def2tzvpp-IEFPCM(water) Energy	-823.296058
M06-2X/def2tzvpp-IEFPCM(water) Free Energy (Quasiharmonic)	-823.139571
Number of Imaginary Frequencies	0

Frequencies (Top 3 out of 63)

```
   1.      21.0489 cm-1
   2.      52.1114 cm-1
   3.      76.2079 cm-1
```

M06-2X/def2tzvpp-IEFPCM(water) Molecular Geometry in Cartesian Coordinates

```
C          0.987887        1.418241       -0.109080
C         -0.353657        1.557836        0.171448
C         -1.464401        0.806381       -0.460409
O          1.931800        2.145299        0.278951
O          1.375738        0.383668       -0.989221
C          1.058122       -0.960972       -0.659646
C          2.319008       -1.732663       -0.309084
C          2.976344       -1.199234        0.956763
H         -2.253605        1.464579       -0.832758
H         -1.120453        0.196673       -1.295227
H          0.560710       -1.417630       -1.520452
H          0.359276       -0.985669        0.179237
H          3.013603       -1.671993       -1.150336
H          2.055298       -2.786559       -0.189081
H          3.192222       -0.135959        0.847469
H          3.905652       -1.723328        1.179287
H          2.309067       -1.316979        1.813401
S         -2.316867       -0.319596        0.728270
C         -3.676769       -0.896211       -0.309683
H         -4.283196       -0.056114       -0.645433
H         -3.297394       -1.437637       -1.174912
H         -4.296245       -1.565087        0.283468
H         -0.614575        2.357240        0.853316
```

---

n-propylacrylate_HEI_22

Datum	Value
M06-2X/def2tzvpp-IEFPCM(water) Energy	-823.296867
M06-2X/def2tzvpp-IEFPCM(water) Free Energy (Quasiharmonic)	-823.140117
Number of Imaginary Frequencies	0

Frequencies (Top 3 out of 63)

```
   1.      30.0579 cm-1
   2.      44.6862 cm-1
   3.      87.5201 cm-1
```

M06-2X/def2tzvpp-IEFPCM(water) Molecular Geometry in Cartesian Coordinates

```
C        -0.947143      1.317946      0.073572
C         0.383686      1.509312     -0.216832
C         1.524330      0.830888      0.444765
O        -1.935842      1.933198     -0.389904
O        -1.255478      0.345335      1.057761
C        -1.130639     -1.006609      0.638973
C        -2.041336     -1.361058     -0.526079
C        -3.515340     -1.152194     -0.209517
H         2.306898      1.529067      0.752386
H         1.204525      0.275029      1.325410
H        -1.395520     -1.608526      1.511764
H        -0.093371     -1.228965      0.374548
H        -1.850530     -2.404714     -0.789225
H        -1.754789     -0.759685     -1.392782
H        -3.815218     -1.750785      0.653214
H        -4.147388     -1.436486     -1.050974
H        -3.697582     -0.103491      0.019652
S         2.369879     -0.359846     -0.683536
C         3.749612     -0.860542      0.367567
H         4.362528     -1.565593     -0.189408
H         4.357458      0.002225      0.636474
H         3.386514     -1.344055      1.273216
H         0.609937      2.258798     -0.965241
```

---

n-propylacrylate_HEI_23

Datum	Value
M06-2X/def2tzvpp-IEFPCM(water) Energy	-823.297094
M06-2X/def2tzvpp-IEFPCM(water) Free Energy (Quasiharmonic)	-823.140476
Number of Imaginary Frequencies	0

Frequencies (Top 3 out of 63)

```
  1.     34.8166 cm-1
  2.     55.0050 cm-1
  3.     80.0592 cm-1
```

M06-2X/def2tzvpp-IEFPCM(water) Molecular Geometry in Cartesian Coordinates

```
C         -1.034973        1.572181       -0.046648
C          0.294852        1.632613       -0.391153
C          1.414693        0.985466        0.333872
O         -1.998456        2.174152       -0.575717
O         -1.374444        0.776286        1.077593
C         -1.325580       -0.626372        0.872610
C         -2.237111       -1.111573       -0.242252
C         -2.143753       -2.624350       -0.396613
H          2.251962        1.666528        0.505309
H          1.096829        0.594163        1.299627
H         -1.629398       -1.076796        1.820542
H         -0.301814       -0.950936        0.661349
H         -1.947995       -0.620781       -1.174344
H         -3.263962       -0.809064       -0.026482
H         -1.122021       -2.925634       -0.635521
H         -2.793610       -2.985176       -1.192844
H         -2.431821       -3.130663        0.526402
S          2.135421       -0.418648       -0.622822
C          3.527406       -0.844125        0.445167
H          4.073361       -1.662517       -0.018632
H          4.195552        0.008454        0.558435
H          3.176356       -1.161993        1.425721
H          0.539822        2.246248       -1.249316
```

---

n-propylacrylate_HEI_24

Datum	Value
M06-2X/def2tzvpp-IEFPCM(water) Energy	-823.302987
M06-2X/def2tzvpp-IEFPCM(water) Free Energy (Quasiharmonic)	-823.146319
Number of Imaginary Frequencies	0

Frequencies (Top 3 out of 63)

```
1.      30.7752 cm-1
2.      44.5708 cm-1
3.      55.7230 cm-1
```

M06-2X/def2tzvpp-IEFPCM(water) Molecular Geometry in Cartesian Coordinates

```
C          0.466026        1.082777       -0.150424
C         -0.660719        0.719393       -0.861211
C         -1.288266       -0.614541       -0.791742
O          1.077863        2.175854       -0.158560
O          0.949451        0.067725        0.687370
C          2.312652        0.161132        1.062466
C          3.243356       -0.201320       -0.084628
C          2.986482       -1.602577       -0.623845
H         -1.786293       -0.881103       -1.725142
H         -0.566453       -1.395258       -0.556164
H          2.436055       -0.549574        1.882300
H          2.534090        1.162302        1.431518
H          4.273444       -0.115064        0.269882
H          3.114944        0.536991       -0.878538
H          3.666701       -1.846087       -1.439792
H          1.965438       -1.687044       -0.997092
H          3.118911       -2.351194        0.159906
S         -2.599423       -0.868082        0.517826
C         -3.765634        0.393001       -0.022679
H         -4.158761        0.157603       -1.011220
H         -4.587367        0.427375        0.688843
H         -3.273242        1.363977       -0.049798
H         -1.128596        1.490555       -1.458030
```

---

n-propylacrylate_HEI_25

Datum	Value
M06-2X/def2tzvpp-IEFPCM(water) Energy	-823.304482
M06-2X/def2tzvpp-IEFPCM(water) Free Energy (Quasiharmonic)	-823.146932
Number of Imaginary Frequencies	0

Frequencies (Top 3 out of 63)

```
1.      45.5239 cm-1
2.      50.8916 cm-1
3.      79.2493 cm-1
```

M06-2X/def2tzvpp-IEFPCM(water) Molecular Geometry in Cartesian Coordinates

```
C         -0.359024        0.056609        0.003383
C          0.589906       -0.693318       -0.661061
C          1.772169       -1.224997        0.039095
```

```
O          -0.428603        0.341004        1.218897
O          -1.357446        0.547604       -0.857470
C          -2.529293        1.096838       -0.282573
C          -3.669908        0.090999       -0.291327
C          -3.387409       -1.123838        0.582909
H           2.058826       -2.226677       -0.285268
H           1.606408       -1.250300        1.115345
H          -2.794711        1.967173       -0.886155
H          -2.320354        1.428356        0.734428
H          -3.849363       -0.224916       -1.322047
H          -4.577898        0.596934        0.047218
H          -2.469108       -1.619938        0.265233
H          -4.200823       -1.847652        0.529964
H          -3.259782       -0.828364        1.624837
S           3.363976       -0.277680       -0.210860
C           2.842818        1.329417        0.411078
H           3.583256        2.069045        0.115126
H           1.878189        1.578240       -0.030232
H           2.753838        1.320636        1.496082
H           0.500744       -0.818776       -1.731211
```

---

n-propylacrylate_HEI_26

Datum	Value
M06-2X/def2tzvpp-IEFPCM(water) Energy	-823.296874
M06-2X/def2tzvpp-IEFPCM(water) Free Energy (Quasiharmonic)	-823.139927
Number of Imaginary Frequencies	0

Frequencies (Top 3 out of 63)

```
1.      45.7248 cm-1
2.      49.7497 cm-1
3.      76.5644 cm-1
```

M06-2X/def2tzvpp-IEFPCM(water) Molecular Geometry in Cartesian Coordinates

```
C          -0.910053       -1.327867        0.076071
C           0.429124       -1.513584       -0.176792
C           1.543434       -0.789558        0.481895
O          -1.883057       -1.967051       -0.389028
O          -1.242441       -0.324400        1.021359
C          -1.155935        1.008701        0.538264
C          -2.153324        1.305700       -0.570655
```

```
C          -3.597893        1.140762       -0.120439
H           2.323982       -1.460679        0.849448
H           1.190403       -0.191160        1.320921
H          -0.140860        1.218366        0.189555
H          -1.358701        1.649204        1.400367
H          -1.944906        0.645131       -1.415790
H          -1.976011        2.327780       -0.915156
H          -4.295623        1.344371       -0.932778
H          -3.827961        1.824463        0.699300
H          -3.764706        0.122915        0.228770
S           2.408346        0.348804       -0.684165
C           3.756603        0.916318        0.373814
H           4.359826        0.073492        0.708420
H           3.367308        1.449455        1.239804
H           4.382110        1.590963       -0.206356
H           0.683845       -2.286386       -0.891458
```

---

n-propylacrylate_HEI_27_reopt

Datum	Value
M06-2X/def2tzvpp-IEFPCM(water) Energy	-823.296381
M06-2X/def2tzvpp-IEFPCM(water) Free Energy (Quasiharmonic)	-823.139405
Number of Imaginary Frequencies	0

Frequencies (Top 3 out of 63)

```
1.      32.8653 cm-1
2.      55.3591 cm-1
3.      85.5520 cm-1
```

M06-2X/def2tzvpp-IEFPCM(water) Molecular Geometry in Cartesian Coordinates

```
C          -0.716524        1.300004       -0.064584
C           0.659055        1.289240       -0.016124
C           1.548409        0.526963       -0.919409
O          -1.508887        2.009633        0.597818
O          -1.327116        0.446202       -1.018004
C          -1.340563       -0.931587       -0.673302
C          -2.122214       -1.226005        0.597485
C          -3.586173       -0.821104        0.500447
H           2.363140        1.140647       -1.310165
H           1.004164        0.120230       -1.770600
H          -1.804822       -1.442152       -1.520648
```

```
H          -0.318875       -1.306256       -0.568833
H          -2.035395       -2.296888        0.798730
H          -1.644195       -0.707343        1.432332
H          -3.663891        0.250882        0.326683
H          -4.127236       -1.061259        1.415776
H          -4.077480       -1.339051       -0.325878
S           2.413941       -0.951945       -0.195223
C           3.336432       -0.115212        1.106049
H           3.918917       -0.862569        1.639799
H           4.011554        0.626827        0.680966
H           2.653101        0.368969        1.801304
H           1.115461        1.958605        0.702224
```

---

n-propylacrylate_HEI_2

Datum	Value
M06-2X/def2tzvpp-IEFPCM(water) Energy	-823.303018
M06-2X/def2tzvpp-IEFPCM(water) Free Energy (Quasiharmonic)	-823.146391
Number of Imaginary Frequencies	0

Frequencies (Top 3 out of 63)

```
1.      47.5073 cm-1
2.      54.2514 cm-1
3.      64.6931 cm-1
```

M06-2X/def2tzvpp-IEFPCM(water) Molecular Geometry in Cartesian Coordinates

```
C          -0.837496       -1.387925       -0.076233
C           0.506780       -1.679795       -0.193907
C           1.590529       -0.930325        0.479319
O          -1.807527       -1.965231       -0.618774
O          -1.109785       -0.321811        0.793609
C          -2.443868        0.154016        0.821232
C          -2.745094        1.092009       -0.337484
C          -1.868151        2.337469       -0.328092
H           2.429534       -1.570746        0.758237
H           1.239130       -0.416868        1.372739
H          -2.541394        0.691000        1.767613
H          -3.139841       -0.683830        0.821155
H          -2.611259        0.539207       -1.269072
H          -3.799272        1.375386       -0.281348
H          -2.094160        2.990605       -1.170903
```

```
H          -0.813947        2.065918       -0.384470
H          -2.017405        2.909627        0.589818
S           2.323306        0.376917       -0.610897
C           3.634854        0.994298        0.466712
H           3.216713        1.404251        1.384897
H           4.333801        0.196137        0.713503
H           4.169465        1.781141       -0.060810
H           0.768924       -2.494649       -0.854970
```

---

n-propylacrylate_HEI_3

Datum	Value
M06-2X/def2tzvpp-IEFPCM(water) Energy	-823.303798
M06-2X/def2tzvpp-IEFPCM(water) Free Energy (Quasiharmonic)	-823.147099
Number of Imaginary Frequencies	0

Frequencies (Top 3 out of 63)

```
1.      34.0767 cm-1
2.      61.5237 cm-1
3.      62.3333 cm-1
```

M06-2X/def2tzvpp-IEFPCM(water) Molecular Geometry in Cartesian Coordinates

```
C           0.152483        1.578857        0.062461
C          -1.151584        1.502414       -0.387369
C          -1.720481        0.339812       -1.092653
O           0.690620        2.484074        0.742116
O           0.952915        0.501342       -0.333729
C           2.233817        0.414851        0.259681
C           2.979229       -0.741894       -0.376295
C           2.284505       -2.082694       -0.178136
H          -2.466236        0.614209       -1.840220
H          -0.952385       -0.253869       -1.583749
H           2.132185        0.250433        1.338857
H           2.780706        1.348202        0.121152
H           3.980880       -0.771923        0.058426
H           3.103408       -0.539504       -1.442834
H           2.877578       -2.900719       -0.586616
H           1.312317       -2.089372       -0.669810
H           2.123237       -2.279941        0.883847
S          -2.685227       -0.864205       -0.028474
C          -1.409689       -1.285556        1.169960
```

```
H         -0.963960      -0.366519       1.550538
H         -1.877603      -1.829981       1.987162
H         -0.633212      -1.900360       0.719387
H         -1.811236       2.308551      -0.093544
```

---

n-propylacrylate_HEI_4_reopt

Datum	Value
M06-2X/def2tzvpp-IEFPCM(water) Energy	-823.303592
M06-2X/def2tzvpp-IEFPCM(water) Free Energy (Quasiharmonic)	-823.14754
Number of Imaginary Frequencies	0

Frequencies (Top 3 out of 63)

```
1.      20.2783 cm-1
2.      44.1834 cm-1
3.      61.4059 cm-1
```

M06-2X/def2tzvpp-IEFPCM(water) Molecular Geometry in Cartesian Coordinates

```
C          0.154503       1.490006      -0.037317
C         -1.089673       1.371181      -0.627310
C         -1.637756       0.115206      -1.171927
O          0.682396       2.507622       0.469687
O          0.896327       0.306200      -0.037473
C          2.189888       0.363946       0.530698
C          2.795437      -1.024374       0.458473
C          2.959924      -1.524848      -0.971002
H         -2.255097       0.269682      -2.058289
H         -0.851712      -0.592792      -1.428239
H          2.812058       1.078688      -0.016932
H          2.132931       0.707385       1.565905
H          3.766094      -0.994878       0.958393
H          2.166692      -1.714169       1.026772
H          1.993532      -1.590956      -1.469883
H          3.423340      -2.510889      -0.995249
H          3.588767      -0.843239      -1.547100
S         -2.814988      -0.824394      -0.058945
C         -1.725033      -1.108157       1.345370
H         -1.250792      -0.166761       1.621090
H         -2.326166      -1.472602       2.175396
H         -0.956242      -1.840022       1.103599
H         -1.717115       2.252524      -0.611964
```

---

n-propylacrylate_HEI_6_reopt

Datum	Value
M06-2X/def2tzvpp-IEFPCM(water) Energy	-823.303628
M06-2X/def2tzvpp-IEFPCM(water) Free Energy (Quasiharmonic)	-823.147029
Number of Imaginary Frequencies	0

Frequencies (Top 3 out of 63)

```
1.      19.5731 cm-1
2.      44.5186 cm-1
3.      64.6063 cm-1
```

M06-2X/def2tzvpp-IEFPCM(water) Molecular Geometry in Cartesian Coordinates

```
C         -0.414400        1.077059       -0.388395
C          0.647093        0.671720       -1.171015
C          1.360619       -0.611176       -1.020688
O         -1.071749        2.142849       -0.459116
O         -0.769884        0.157559        0.611247
C         -2.109496        0.219857        1.067584
C         -3.086643       -0.333352        0.041614
C         -2.771998       -1.772804       -0.344612
H          1.664413       -1.042024       -1.976318
H          0.755015       -1.346679       -0.494563
H         -2.134771       -0.390036        1.973174
H         -2.371966        1.244489        1.329965
H         -4.095207       -0.263752        0.456495
H         -3.059480        0.307659       -0.841872
H         -3.483563       -2.153640       -1.077026
H         -2.805997       -2.426157        0.529599
H         -1.772205       -1.841365       -0.774366
S          2.990346       -0.541577       -0.109993
C          2.417263        0.096583        1.472649
H          1.778816        0.961148        1.295495
H          3.286623        0.395497        2.053961
H          1.856367       -0.658427        2.020250
H          1.014863        1.387930       -1.894774
```

---

n-propylacrylate_HEI_8

Datum	Value
M06-2X/def2tzvpp-IEFPCM(water) Energy	-823.30361
M06-2X/def2tzvpp-IEFPCM(water) Free Energy (Quasiharmonic)	-823.147606
Number of Imaginary Frequencies	0

Frequencies (Top 3 out of 63)

```
1.      23.7525 cm-1
2.      55.2818 cm-1
3.      62.2719 cm-1
```

M06-2X/def2tzvpp-IEFPCM(water) Molecular Geometry in Cartesian Coordinates

```
C          0.054534        1.475726        0.116495
C          1.379934        1.433415       -0.271457
C          1.989698        0.327936       -1.033020
O         -0.527284        2.340304        0.812618
O         -0.712356        0.410494       -0.366823
C         -2.032355        0.308299        0.127244
C         -2.681021       -0.908814       -0.500397
C         -4.115635       -1.085283       -0.019836
H          2.778510        0.661560       -1.708716
H          1.253559       -0.219004       -1.618555
H         -2.603789        1.209229       -0.110721
H         -2.022112        0.212180        1.217962
H         -2.658051       -0.801174       -1.587021
H         -2.089434       -1.793643       -0.255442
H         -4.151285       -1.210559        1.063503
H         -4.580598       -1.959776       -0.473074
H         -4.721436       -0.214098       -0.273981
S          2.886112       -0.969660       -0.021827
C          1.528178       -1.510427        1.028914
H          0.781893       -2.056621        0.454844
H          1.935442       -2.154827        1.804835
H          1.060903       -0.638177        1.485186
H          2.015735        2.227964        0.096407
```

---

n-propylacrylate_HEI_9

Datum	Value

Datum	Value
M06-2X/def2tzvpp-IEFPCM(water) Energy	-823.303315
M06-2X/def2tzvpp-IEFPCM(water) Free Energy (Quasiharmonic)	-823.146745
Number of Imaginary Frequencies	0

Frequencies (Top 3 out of 63)

```
1.      21.7513 cm-1
2.      50.5084 cm-1
3.      68.9146 cm-1
```

M06-2X/def2tzvpp-IEFPCM(water) Molecular Geometry in Cartesian Coordinates

```
C          0.228927        0.960356        0.372645
C         -0.973687        1.367988       -0.170399
C         -1.803776        0.550729       -1.074820
O          0.975963        1.584398        1.161812
O          0.631398       -0.320137       -0.035323
C          1.947725       -0.712010        0.302243
C          2.985166       -0.142952       -0.650833
C          4.389731       -0.602490       -0.281757
H         -2.307728        1.142069       -1.841047
H         -1.218666       -0.219477       -1.572955
H          1.955298       -1.803084        0.251049
H          2.180902       -0.412863        1.324985
H          2.920131        0.945835       -0.619402
H          2.740065       -0.455886       -1.668385
H          5.134002       -0.206238       -0.971457
H          4.461845       -1.691498       -0.301620
H          4.654937       -0.271031        0.723568
S         -3.248085       -0.342324       -0.287529
C         -2.358363       -1.326452        0.928929
H         -1.683694       -0.677259        1.485654
H         -3.085539       -1.767237        1.607180
H         -1.782285       -2.115657        0.449536
H         -1.353133        2.324413        0.165387
```

---

n-propylacrylate_TS_10_UNCON_m062x_reopt

Datum	Value
M06-2X/def2tzvpp-IEFPCM(water) Energy	-823.294629

Datum	Value
M06-2X/def2tzvpp-IEFPCM(water) Free Energy (Quasiharmonic)	-823.138262
Number of Imaginary Frequencies	1

Frequencies (Top 3 out of 63)

```
1.    -193.9425 cm-1
2.      34.3736 cm-1
3.      39.7041 cm-1
```

M06-2X/def2tzvpp-IEFPCM(water) Molecular Geometry in Cartesian Coordinates

```
C        -0.460433        0.845650        0.416290
C         0.621387        1.392985       -0.343796
C         1.287341        0.703731       -1.335108
O        -1.103298        1.440823        1.275010
O        -0.738673       -0.451676        0.099206
C        -1.889850       -1.065966        0.676393
C        -3.019370       -1.129120       -0.336087
C        -3.497320        0.249095       -0.773929
H         1.944242        1.248838       -1.996074
H         0.828436       -0.174060       -1.761259
H        -1.581452       -2.068690        0.970738
H        -2.190912       -0.511847        1.563512
H        -2.682095       -1.701440       -1.203073
H        -3.842002       -1.689664        0.113627
H        -2.681348        0.817729       -1.222585
H        -3.871689        0.819147        0.076959
H        -4.295771        0.171037       -1.511187
S         3.113866       -0.596380       -0.505651
C         2.710857       -0.422366        1.242058
H         1.900111        0.312342        1.337088
H         2.370821       -1.360769        1.679152
H         3.561832       -0.059647        1.817018
H         0.938058        2.381872       -0.042162
```

---

n-propylacrylate_TS_11_UNCON_m062x

Datum	Value
M06-2X/def2tzvpp-IEFPCM(water) Energy	-823.296727
M06-2X/def2tzvpp-IEFPCM(water) Free Energy (Quasiharmonic)	-823.140393

Datum	Value
Number of Imaginary Frequencies	1

Frequencies (Top 3 out of 63)

```
1.    -191.7471 cm-1
2.      41.1841 cm-1
3.      48.7538 cm-1
```

M06-2X/def2tzvpp-IEFPCM(water) Molecular Geometry in Cartesian Coordinates

```
C          -0.333162       -1.134016        0.032013
C           0.780240       -1.225104       -0.861301
C           2.057021       -1.378750       -0.370235
O          -0.304430       -1.207069        1.255279
O          -1.514636       -0.956662       -0.631327
C          -2.667102       -0.680622        0.162527
C          -2.712774        0.776628        0.589297
C          -2.730934        1.733454       -0.596013
H           2.862259       -1.620930       -1.046882
H           2.178642       -1.727661        0.643898
H          -3.515445       -0.915359       -0.480700
H          -2.683993       -1.341632        1.027385
H          -1.851037        0.981025        1.227245
H          -3.605709        0.917860        1.202346
H          -1.833900        1.612643       -1.203664
H          -2.775690        2.770290       -0.264281
H          -3.596432        1.545620       -1.234064
S           3.074700        0.721317        0.184953
C           1.579800        1.718502        0.052157
H           0.786255        1.097855       -0.387013
H           1.234030        2.069873        1.024262
H           1.727808        2.580385       -0.597198
H           0.592012       -1.061737       -1.912862
```

---

n-propylacrylate_TS_12_UNCON_m062x

Datum	Value
M06-2X/def2tzvpp-IEFPCM(water) Energy	-823.296536
M06-2X/def2tzvpp-IEFPCM(water) Free Energy (Quasiharmonic)	-823.140925
Number of Imaginary Frequencies	1

Frequencies (Top 3 out of 63)

```
1.    -190.1303 cm-1
2.      28.0083 cm-1
3.      49.0193 cm-1
```

M06-2X/def2tzvpp-IEFPCM(water) Molecular Geometry in Cartesian Coordinates

```
C          0.199249       -0.897648        0.042587
C         -0.779120       -0.838550       -0.998765
C         -2.077403       -1.229479       -0.754977
O          0.027666       -1.281149        1.194606
O          1.427744       -0.471173       -0.367465
C          2.468089       -0.495067        0.606670
C          3.717121        0.077940       -0.028227
C          3.560974        1.541325       -0.421572
H         -2.755216       -1.364285       -1.583887
H         -2.271357       -1.844554        0.110705
H          2.165149        0.096216        1.473847
H          2.627725       -1.521276        0.941341
H          4.533459       -0.032414        0.688170
H          3.978287       -0.523486       -0.901535
H          3.316930        2.149865        0.450960
H          2.760803        1.664127       -1.150627
H          4.479496        1.932007       -0.858009
S         -3.415842        0.535637        0.144208
C         -2.085478        1.734447        0.341789
H         -1.168801        1.306926       -0.087673
H         -2.299460        2.665125       -0.182041
H         -1.893694        1.961524        1.390022
H         -0.483718       -0.399204       -1.940756
```

---

n-propylacrylate_TS_13_UNCON_m062x

Datum	Value
M06-2X/def2tzvpp-IEFPCM(water) Energy	-823.296048
M06-2X/def2tzvpp-IEFPCM(water) Free Energy (Quasiharmonic)	-823.139897
Number of Imaginary Frequencies	1

Frequencies (Top 3 out of 63)

```
1.    -185.1824 cm-1
2.      35.3465 cm-1
3.      44.3511 cm-1
```

M06-2X/def2tzvpp-IEFPCM(water) Molecular Geometry in Cartesian Coordinates

```
C          0.334230      -0.161132       0.123142
C         -0.600101      -0.978203      -0.588789
C         -1.675857      -1.537164       0.063308
O          0.308080       0.116821       1.316415
O          1.324209       0.319008      -0.686306
C          2.349416       1.096249      -0.069076
C          3.415975       0.223636       0.569010
C          4.103170      -0.692329      -0.435579
H         -2.261156      -2.296647      -0.431967
H         -1.649836      -1.595425       1.140834
H          2.778623       1.688502      -0.877673
H          1.905378       1.766653       0.664924
H          4.148793       0.884347       1.037769
H          2.957266      -0.361881       1.367021
H          4.578868      -0.111314      -1.227715
H          3.384013      -1.366415      -0.900649
H          4.870774      -1.298194       0.044658
S         -3.565654      -0.059682       0.152365
C         -2.711422       1.401581      -0.465931
H         -2.593160       2.162806       0.304738
H         -1.710669       1.100374      -0.804190
H         -3.232678       1.843528      -1.314078
H         -0.476524      -1.063890      -1.658892
```

---

n-propylacrylate_TS_14_UNCON_m062x

Datum	Value
M06-2X/def2tzvpp-IEFPCM(water) Energy	-823.296497
M06-2X/def2tzvpp-IEFPCM(water) Free Energy (Quasiharmonic)	-823.1404
Number of Imaginary Frequencies	1

Frequencies (Top 3 out of 63)

```
1.    -187.6321 cm-1
2.      36.6578 cm-1
3.      61.9783 cm-1
```

M06-2X/def2tzvpp-IEFPCM(water) Molecular Geometry in Cartesian Coordinates

```
C          0.173921      -1.063058       0.114872
C         -1.050143      -1.379478      -0.554769
C         -2.245029      -1.339474       0.126346
O          0.311470      -0.770823       1.296772
O          1.252950      -1.122909      -0.721909
C          2.505609      -0.698172      -0.192238
C          2.641161       0.813278      -0.219577
C          4.001271       1.254772       0.304905
H         -3.134932      -1.738627      -0.335911
H         -2.224432      -1.371755       1.204938
H          2.625422      -1.075400       0.823481
H          3.260470      -1.159801      -0.828754
H          2.499990       1.159236      -1.245637
H          1.841424       1.246751       0.383216
H          4.143982       0.931814       1.337159
H          4.102155       2.338719       0.276191
H          4.807710       0.827128      -0.293018
S         -3.183878       0.873623       0.178955
C         -1.733775       1.725874      -0.467110
H         -1.985867       2.361881      -1.314639
H         -1.245055       2.335610       0.292609
H         -1.012803       0.972664      -0.813633
H         -1.009479      -1.538788      -1.622758
```

---

n-propylacrylate_TS_15_UNCON_m062x

Datum	Value
M06-2X/def2tzvpp-IEFPCM(water) Energy	-823.296181
M06-2X/def2tzvpp-IEFPCM(water) Free Energy (Quasiharmonic)	-823.139947
Number of Imaginary Frequencies	1

Frequencies (Top 3 out of 63)

```
1.    -189.5306 cm-1
2.      37.4187 cm-1
3.      50.8367 cm-1
```

M06-2X/def2tzvpp-IEFPCM(water) Molecular Geometry in Cartesian Coordinates

```
C          0.411492      -1.032445       0.130026
C         -0.742747      -1.344155      -0.656768
C         -1.977469      -1.481960      -0.062174
O          0.445152      -0.851577       1.340906
O          1.546110      -0.965764      -0.629880
C          2.782297      -0.646972       0.008143
C          3.187507       0.784436      -0.293700
C          2.225120       1.808291       0.292926
H         -2.791867      -1.908586      -0.627310
H         -2.018921      -1.637576       1.005081
H          2.688690      -0.806460       1.080925
H          3.517184      -1.344896      -0.392535
H          4.192610       0.938471       0.105463
H          3.254744       0.908589      -1.376827
H          2.187039       1.726266       1.379745
H          1.214499       1.653252      -0.086868
H          2.528302       2.823322       0.037503
S         -3.137163       0.601518       0.159917
C         -1.789694       1.646535      -0.418177
H         -0.980045       0.995018      -0.776067
H         -2.098378       2.284607      -1.245287
H         -1.389084       2.275641       0.376582
H         -0.619287      -1.395584      -1.728950
```

---

n-propylacrylate_TS_16_UNCON_m062x

Datum	Value
M06-2X/def2tzvpp-IEFPCM(water) Energy	-823.296181
M06-2X/def2tzvpp-IEFPCM(water) Free Energy (Quasiharmonic)	-823.139947
Number of Imaginary Frequencies	1

Frequencies (Top 3 out of 63)

```
1.    -189.5297 cm-1
2.      37.4113 cm-1
3.      50.8273 cm-1
```

M06-2X/def2tzvpp-IEFPCM(water) Molecular Geometry in Cartesian Coordinates

```
C          0.411487       -1.032443        0.130027
C         -0.742742       -1.344145       -0.656785
C         -1.977470       -1.481962       -0.062208
O          0.445130       -0.851580        1.340907
O          1.546116       -0.965766       -0.629863
C          2.782295       -0.646975        0.008177
C          3.187523        0.784422       -0.293694
C          2.225127        1.808299        0.292879
H         -2.791861       -1.908577       -0.627362
H         -2.018936       -1.637596        1.005044
H          2.688663       -0.806433        1.080962
H          3.517183       -1.344917       -0.392467
H          4.192617        0.938462        0.105490
H          3.254789        0.908544       -1.376823
H          2.187028        1.726317        1.379700
H          1.214512        1.653245       -0.086927
H          2.528313        2.823320        0.037422
S         -3.137169        0.601516        0.159917
C         -1.789689        1.646540       -0.418139
H         -2.098370        2.284653       -1.245219
H         -1.389066        2.275607        0.376645
H         -0.980051        0.995030       -0.776064
H         -0.619268       -1.395562       -1.728967
```

---

n-propylacrylate_TS_17_UNCON_m062x

Datum	Value
M06-2X/def2tzvpp-IEFPCM(water) Energy	-823.296474
M06-2X/def2tzvpp-IEFPCM(water) Free Energy (Quasiharmonic)	-823.140846
Number of Imaginary Frequencies	1

Frequencies (Top 3 out of 63)

```
1.    -198.0046 cm-1
2.      27.4894 cm-1
3.      45.2616 cm-1
```

M06-2X/def2tzvpp-IEFPCM(water) Molecular Geometry in Cartesian Coordinates

```
C          0.218471       -0.483343        0.372513
C         -0.691294       -1.043991       -0.578528
C         -1.923312       -1.509633       -0.175566
```

```
O          0.040996       -0.356807        1.579091
O          1.386079       -0.073017       -0.200550
C          2.352963        0.510253        0.669366
C          3.580196        0.844659       -0.151436
C          4.256213       -0.390668       -0.732540
H         -2.518501       -2.094304       -0.860311
H         -2.073313       -1.724024        0.871597
H          1.929556        1.402867        1.132875
H          2.598368       -0.196269        1.465422
H          3.295350        1.532374       -0.950489
H          4.275547        1.382044        0.496267
H          5.145561       -0.122038       -1.301481
H          3.579580       -0.928182       -1.395924
H          4.558818       -1.073198        0.063539
S         -3.577228        0.218945       -0.097022
C         -2.454085        1.594959       -0.398812
H         -1.461692        1.186128       -0.633400
H         -2.776566        2.199640       -1.245416
H         -2.356209        2.240980        0.473094
H         -0.412371       -1.003965       -1.621735
```

---

n-propylacrylate_TS_18_UNCON_m062x

Datum	Value
M06-2X/def2tzvpp-IEFPCM(water) Energy	-823.296473
M06-2X/def2tzvpp-IEFPCM(water) Free Energy (Quasiharmonic)	-823.140802
Number of Imaginary Frequencies	1

Frequencies (Top 3 out of 63)

```
1.    -190.2879 cm-1
2.      35.5518 cm-1
3.      56.3993 cm-1
```

M06-2X/def2tzvpp-IEFPCM(water) Molecular Geometry in Cartesian Coordinates

```
C          0.110711       -0.712318        0.164784
C         -0.950505       -1.079247       -0.722276
C         -2.189540       -1.417021       -0.227802
O          0.068007       -0.661721        1.388849
O          1.262329       -0.407735       -0.499578
C          2.378818       -0.032554        0.300600
C          3.543380        0.255751       -0.621323
```

```
C          4.782494       0.660616       0.166520
H         -2.915717      -1.876330      -0.880697
H         -2.272964      -1.672564       0.817706
H          2.124239       0.848040       0.894990
H          2.621003      -0.840337       0.994777
H          3.750281      -0.633213      -1.220122
H          3.259453       1.050225      -1.314041
H          4.592535       1.556354       0.759491
H          5.619015       0.870022      -0.498339
H          5.087108      -0.133651       0.849440
S         -3.608472       0.495254       0.084571
C         -2.324714       1.739482      -0.142042
H         -1.420364       1.240681      -0.516255
H         -2.618701       2.491779      -0.872726
H         -2.072228       2.241814       0.791505
H         -0.764677      -0.999489      -1.783799
```

---

n-propylacrylate_TS_19_UNCON_m062x

Datum	Value
M06-2X/def2tzvpp-IEFPCM(water) Energy	-823.296128
M06-2X/def2tzvpp-IEFPCM(water) Free Energy (Quasiharmonic)	-823.140015
Number of Imaginary Frequencies	1

Frequencies (Top 3 out of 63)

```
1.    -189.7273 cm-1
2.      42.3735 cm-1
3.      52.5300 cm-1
```

M06-2X/def2tzvpp-IEFPCM(water) Molecular Geometry in Cartesian Coordinates

```
C         -0.245252      -0.366061       0.098606
C          0.851548      -1.006696       0.757681
C          1.909080      -1.503044       0.030354
O         -0.378731      -0.189195      -1.106604
O         -1.198436       0.062468       0.978610
C         -2.365086       0.667791       0.428259
C         -3.383610      -0.363851      -0.019891
C         -4.645237       0.305485      -0.549667
H          2.628955      -2.147305       0.511150
H          1.774857      -1.660317      -1.028938
H         -2.775781       1.282837       1.229156
```

```
H          -2.086061        1.315518       -0.403132
H          -2.932818       -0.989970       -0.790906
H          -3.624798       -1.009963        0.826593
H          -5.381029       -0.432937       -0.864719
H          -4.418300        0.940096       -1.407596
H          -5.105522        0.931950        0.216085
S           3.596642        0.154962       -0.392444
C           2.672054        1.561187        0.251928
H           2.405365        2.268412       -0.532893
H           1.743087        1.187129        0.703790
H           3.229720        2.091676        1.022669
H           0.852419       -1.011557        1.838180
```

---

n-propylacrylate_TS_1_UNCON_m062x

Datum	Value
M06-2X/def2tzvpp-IEFPCM(water) Energy	-823.296286
M06-2X/def2tzvpp-IEFPCM(water) Free Energy (Quasiharmonic)	-823.139308
Number of Imaginary Frequencies	1

Frequencies (Top 3 out of 63)

```
1.    -202.1746 cm-1
2.      48.0960 cm-1
3.      57.9816 cm-1
```

M06-2X/def2tzvpp-IEFPCM(water) Molecular Geometry in Cartesian Coordinates

```
C           0.587763       -1.480089        0.183356
C          -0.792512       -1.807984        0.018023
C          -1.604022       -1.232185       -0.941083
O           1.327751       -1.900521        1.069364
O           1.061766       -0.627792       -0.771860
C           2.350288       -0.058020       -0.554249
C           2.252103        1.171464        0.332345
C           1.438293        2.287370       -0.311152
H          -2.582017       -1.655974       -1.114127
H          -1.147538       -0.768686       -1.801087
H           3.013963       -0.804239       -0.122579
H           2.716236        0.213711       -1.544894
H           1.804198        0.874422        1.283774
H           3.264973        1.517952        0.549776
H           0.431219        1.941739       -0.549601
```

```
H          1.910533       2.623344      -1.236286
H          1.351479       3.147054       0.353426
S         -2.522360       0.835877      -0.245112
C         -1.592419       0.988446       1.289479
H         -0.947963       1.866775       1.289801
H         -2.249519       1.036054       2.156943
H         -0.955935       0.099332       1.394727
H         -1.204977      -2.453021       0.781583
```

---

n-propylacrylate_TS_20_UNCON_m062x

Datum	Value
M06-2X/def2tzvpp-IEFPCM(water) Energy	-823.280924
M06-2X/def2tzvpp-IEFPCM(water) Free Energy (Quasiharmonic)	-823.125582
Number of Imaginary Frequencies	1

Frequencies (Top 3 out of 63)

```
1.    -231.7627 cm-1
2.      27.8383 cm-1
3.      37.3630 cm-1
```

M06-2X/def2tzvpp-IEFPCM(water) Molecular Geometry in Cartesian Coordinates

```
C         -1.423935       1.281843      -0.120285
C         -0.160134       1.801755       0.306970
C          1.071046       1.512718      -0.239658
O         -2.501410       1.775707       0.194731
O         -1.476077       0.243291      -1.013722
C         -0.686857      -0.938221      -0.831128
C         -1.548299      -2.054371      -0.270950
C         -2.049718      -1.732878       1.131186
H          1.876931       2.216391      -0.095848
H          1.133908       0.934565      -1.153056
H         -0.293178      -1.204035      -1.813097
H          0.148388      -0.745839      -0.158029
H         -2.388591      -2.236154      -0.944344
H         -0.945377      -2.965656      -0.257485
H         -2.670482      -0.836270       1.122618
H         -1.209480      -1.552668       1.805508
H         -2.642518      -2.550323       1.540260
S          2.463483      -0.135804       0.836933
C          3.111355      -0.717494      -0.755036
```

```
H          2.341348       -1.245365       -1.321182
H          3.457647        0.124072       -1.357874
H          3.952214       -1.394324       -0.610810
H         -0.237393        2.556366        1.077735
```

---

n-propylacrylate_TS_21_UNCON_m062x

Datum	Value
M06-2X/def2tzvpp-IEFPCM(water) Energy	-823.286504
M06-2X/def2tzvpp-IEFPCM(water) Free Energy (Quasiharmonic)	-823.130025
Number of Imaginary Frequencies	1

Frequencies (Top 3 out of 63)

```
1.    -196.7059 cm-1
2.      30.8511 cm-1
3.      63.0580 cm-1
```

M06-2X/def2tzvpp-IEFPCM(water) Molecular Geometry in Cartesian Coordinates

```
C         -0.934059        1.491399        0.197691
C          0.484815        1.699154        0.219895
C          1.384707        1.345171       -0.762291
O         -1.717355        2.083408        0.931539
O         -1.498033        0.675918       -0.746081
C         -0.937633       -0.602084       -1.063113
C         -1.924037       -1.685020       -0.669914
C         -2.172139       -1.708736        0.832507
H          2.315571        1.886467       -0.822817
H          1.031203        0.946742       -1.702227
H         -0.741488       -0.623498       -2.136759
H          0.007619       -0.742967       -0.542569
H         -2.861750       -1.527571       -1.207093
H         -1.520578       -2.643405       -1.005053
H         -2.572292       -0.751795        1.169197
H         -1.241220       -1.890858        1.373076
H         -2.881382       -2.488697        1.107265
S          2.652671       -0.636804       -0.241878
C          1.789642       -1.041246        1.287570
H          1.002979       -0.290040        1.454487
H          1.314662       -2.021114        1.238005
H          2.458429       -1.022682        2.146824
H          0.820844        2.291861        1.059982
```

---

n-propylacrylate_TS_22_UNCON_m062x

Datum	Value
M06-2X/def2tzvpp-IEFPCM(water) Energy	-823.280737
M06-2X/def2tzvpp-IEFPCM(water) Free Energy (Quasiharmonic)	-823.125298
Number of Imaginary Frequencies	1

Frequencies (Top 3 out of 63)

```
1.    -223.1260 cm-1
2.      26.1672 cm-1
3.      42.5501 cm-1
```

M06-2X/def2tzvpp-IEFPCM(water) Molecular Geometry in Cartesian Coordinates

```
C          1.250611        1.331741        0.098797
C         -0.029792        1.765899       -0.367269
C         -1.238806        1.407351        0.181883
O          2.313364        1.840134       -0.234948
O          1.308956        0.338569        1.052449
C          0.784766       -0.950441        0.703152
C          1.493970       -1.543438       -0.500434
C          2.994752       -1.698297       -0.294971
H         -2.104257        2.022734       -0.012277
H         -1.261886        0.870203        1.121011
H          0.956550       -1.564572        1.587599
H         -0.285409       -0.895706        0.506673
H          1.032912       -2.512022       -0.707876
H          1.293172       -0.913733       -1.372562
H          3.469524       -2.142662       -1.169241
H          3.202072       -2.339176        0.563996
H          3.455416       -0.728105       -0.111651
S         -2.515018       -0.412939       -0.808431
C         -3.282247       -0.752625        0.800155
H         -2.568664       -1.213137        1.486819
H         -3.638883        0.173440        1.256014
H         -4.133430       -1.423655        0.695133
H          0.005079        2.482647       -1.176626
```

n-propylacrylate_TS_23_UNCON_m062x

Datum	Value
M06-2X/def2tzvpp-IEFPCM(water) Energy	-823.280607
M06-2X/def2tzvpp-IEFPCM(water) Free Energy (Quasiharmonic)	-823.124984
Number of Imaginary Frequencies	1

Frequencies (Top 3 out of 63)

```
1.    -237.7369 cm-1
2.      33.0504 cm-1
3.      50.5078 cm-1
```

M06-2X/def2tzvpp-IEFPCM(water) Molecular Geometry in Cartesian Coordinates

```
C         -2.001499       -0.908009       -0.017992
C         -0.941058       -1.871085       -0.034748
C          0.293039       -1.800093        0.580837
O         -3.152495       -1.174244       -0.352911
O         -1.814089        0.364645        0.443467
C         -0.565355        1.048693        0.332074
C         -0.813611        2.456776       -0.167690
C          0.497972        3.227357       -0.257206
H          0.814464       -2.727456        0.765204
H          0.508979       -1.022459        1.299560
H         -0.093562        1.085528        1.318189
H          0.101840        0.519115       -0.348290
H         -1.291393        2.404519       -1.147962
H         -1.508951        2.963630        0.504192
H          0.340136        4.236998       -0.633165
H          1.197746        2.723171       -0.926631
H          0.971973        3.304331        0.722820
S          2.174439       -1.063354       -0.698285
C          2.872007       -0.083458        0.660836
H          2.827303       -0.643361        1.597127
H          3.915939        0.159954        0.468435
H          2.323300        0.850526        0.795900
H         -1.215093       -2.785133       -0.543927
```

n-propylacrylate_TS_24_UNCON_m062x

Datum	Value
M06-2X/def2tzvpp-IEFPCM(water) Energy	-823.285149
M06-2X/def2tzvpp-IEFPCM(water) Free Energy (Quasiharmonic)	-823.128957
Number of Imaginary Frequencies	1

Frequencies (Top 3 out of 63)

```
1.    -200.8378 cm-1
2.      34.0236 cm-1
3.      43.7972 cm-1
```

M06-2X/def2tzvpp-IEFPCM(water) Molecular Geometry in Cartesian Coordinates

```
C         -0.480049        1.691898        0.135819
C          0.890081        1.598848       -0.278761
C          1.448978        0.760844       -1.222967
O         -0.925616        2.653237        0.755428
O         -1.402443        0.742886       -0.200564
C         -1.068789       -0.637308       -0.366247
C         -2.109512       -1.475802        0.349392
C         -3.511695       -1.296369       -0.217231
H          2.375971        1.069729       -1.680634
H          0.825126        0.147527       -1.853931
H         -1.070572       -0.873996       -1.434178
H         -0.074933       -0.835370        0.026378
H         -1.802727       -2.521073        0.266535
H         -2.093159       -1.221748        1.411656
H         -4.232429       -1.922690        0.307302
H         -3.536675       -1.567185       -1.274330
H         -3.836200       -0.260248       -0.128097
S          2.580630       -1.124501       -0.282897
C          2.068116       -0.849372        1.422408
H          1.450254       -1.664186        1.799694
H          2.922970       -0.725991        2.085451
H          1.474055        0.076013        1.460878
H          1.509932        2.365820        0.166246
```

---

n-propylacrylate_TS_25_UNCON_m062x

Datum	Value
M06-2X/def2tzvpp-IEFPCM(water) Energy	-823.291258

Datum	Value
M06-2X/def2tzvpp-IEFPCM(water) Free Energy (Quasiharmonic)	-823.136363
Number of Imaginary Frequencies	1

Frequencies (Top 3 out of 63)

```
1.    -221.3390 cm-1
2.      34.5866 cm-1
3.      49.1308 cm-1
```

M06-2X/def2tzvpp-IEFPCM(water) Molecular Geometry in Cartesian Coordinates

```
C          0.497808       -0.789976        0.208781
C         -0.657176       -1.353626       -0.417844
C         -1.912433       -1.103366        0.085436
O          0.520828       -0.074880        1.203637
O          1.655017       -1.142194       -0.425948
C          2.868519       -0.604390        0.092902
C          3.135793        0.796512       -0.425463
C          4.455515        1.336389        0.110460
H         -2.756434       -1.674408       -0.273948
H         -1.999535       -0.734170        1.098038
H          2.836671       -0.608581        1.182541
H          3.651720       -1.286925       -0.237346
H          3.152158        0.770474       -1.516852
H          2.310682        1.444237       -0.126632
H          4.656245        2.336810       -0.270009
H          5.287627        0.693512       -0.181152
H          4.440519        1.388597        1.200105
S         -3.055023        0.882777       -0.630411
C         -4.343185        0.631059        0.622890
H         -5.293057        1.057453        0.303695
H         -4.070063        1.082318        1.576699
H         -4.497469       -0.438506        0.789145
H         -0.514492       -1.894277       -1.342191
```

---

n-propylacrylate_TS_26_UNCON_m062x

Datum	Value
M06-2X/def2tzvpp-IEFPCM(water) Energy	-823.29573
M06-2X/def2tzvpp-IEFPCM(water) Free Energy (Quasiharmonic)	-823.139636

Datum	Value
Number of Imaginary Frequencies	1

Frequencies (Top 3 out of 63)

```
1.    -190.4109 cm-1
2.      33.0459 cm-1
3.      40.3239 cm-1
```

M06-2X/def2tzvpp-IEFPCM(water) Molecular Geometry in Cartesian Coordinates

```
C          0.384173       -0.111023        0.033183
C         -0.580155       -0.693207        0.916837
C         -1.603952       -1.464577        0.416938
O          0.417613       -0.217724       -1.186061
O          1.322169        0.618057        0.709050
C          2.411105        1.176391       -0.024724
C          3.666443        0.344707        0.169599
C          3.529895       -1.070505       -0.376361
H         -2.209372       -2.048392        1.093114
H         -1.509789       -1.855575       -0.584609
H          2.144258        1.239060       -1.078212
H          2.554012        2.183095        0.366862
H          3.905735        0.314040        1.234891
H          4.490869        0.862649       -0.325648
H          4.441269       -1.643944       -0.209640
H          3.325321       -1.055110       -1.447248
H          2.708260       -1.597283        0.111080
S         -3.508833       -0.149841       -0.243338
C         -2.723007        1.459550       -0.043056
H         -2.577362        1.964613       -0.997549
H         -3.301284        2.111663        0.610235
H         -1.736865        1.308967        0.416914
H         -0.518979       -0.437020        1.964794
```

---

n-propylacrylate_TS_27_UNCON_m062x

Datum	Value
M06-2X/def2tzvpp-IEFPCM(water) Energy	-823.286476
M06-2X/def2tzvpp-IEFPCM(water) Free Energy (Quasiharmonic)	-823.129522
Number of Imaginary Frequencies	1

Frequencies (Top 3 out of 63)

```
1.    -181.3012 cm-1
2.      60.8337 cm-1
3.      64.9417 cm-1
```

M06-2X/def2tzvpp-IEFPCM(water) Molecular Geometry in Cartesian Coordinates

```
C          0.799288        1.434898        0.195528
C         -0.611953        1.670001        0.299670
C         -1.532407        1.376569       -0.675269
O          1.635042        1.893569        0.960075
O          1.256515        0.725865       -0.894379
C          0.925099       -0.668589       -0.936140
C          1.645796       -1.449293        0.148557
C          3.161349       -1.339291        0.054503
H         -2.490574        1.870480       -0.660738
H         -1.196496        1.036013       -1.643720
H         -0.154766       -0.803483       -0.859974
H          1.248424       -1.003201       -1.922555
H          1.302869       -1.098584        1.125763
H          1.330980       -2.492639        0.066177
H          3.649067       -1.931778        0.827956
H          3.474058       -0.302287        0.171297
H          3.515932       -1.693010       -0.915429
S         -2.753393       -0.725169       -0.245154
C         -1.759628       -1.221964        1.174824
H         -1.203893       -2.140368        0.983587
H         -1.030489       -0.427398        1.388270
H         -2.370683       -1.364582        2.065082
H         -0.917856        2.184077        1.201142
```

---

n-propylacrylate_TS_28_UNCON_m062x

Datum	Value
M06-2X/def2tzvpp-IEFPCM(water) Energy	-823.280737
M06-2X/def2tzvpp-IEFPCM(water) Free Energy (Quasiharmonic)	-823.125294
Number of Imaginary Frequencies	1

Frequencies (Top 3 out of 63)

```
1.    -223.1178 cm-1
2.      26.4036 cm-1
3.      42.4995 cm-1
```

M06-2X/def2tzvpp-IEFPCM(water) Molecular Geometry in Cartesian Coordinates

```
C          1.250599        1.331740        0.098764
C         -0.029813        1.765859       -0.367310
C         -1.238849        1.407272        0.181791
O          2.313338        1.840139       -0.235026
O          1.308988        0.338637        1.052484
C          0.784893       -0.950440        0.703292
C          1.494043       -1.543439       -0.500324
C          2.994850       -1.698206       -0.294983
H         -2.104319        2.022590       -0.012502
H         -1.261972        0.870218        1.120972
H          0.956813       -1.564510        1.587756
H         -0.285303       -0.895825        0.506906
H          1.033027       -2.512060       -0.707687
H          1.293132       -0.913787       -1.372463
H          3.202278       -2.338925        0.564079
H          3.455507       -0.727971       -0.111885
H          3.469548       -2.142715       -1.169220
S         -2.514952       -0.413138       -0.808413
C         -3.282553       -0.752352        0.800095
H         -4.134244       -1.422722        0.694972
H         -2.569382       -1.213427        1.486803
H         -3.638531        0.173968        1.255961
H          0.005052        2.482563       -1.176707
```

---

n-propylacrylate_TS_2_UNCON_m062x

Datum	Value
M06-2X/def2tzvpp-IEFPCM(water) Energy	-823.290649
M06-2X/def2tzvpp-IEFPCM(water) Free Energy (Quasiharmonic)	-823.134391
Number of Imaginary Frequencies	1

Frequencies (Top 3 out of 63)

```
1.    -246.6090 cm-1
2.      48.7749 cm-1
3.      61.2303 cm-1
```

M06-2X/def2tzvpp-IEFPCM(water) Molecular Geometry in Cartesian Coordinates

```
C         -0.918703      -1.453437      -0.045137
C          0.370059      -2.059324      -0.155795
C          1.498848      -1.547064       0.450520
O         -1.952629      -1.852929      -0.573763
O         -0.924292      -0.335293       0.735493
C         -2.141658       0.402938       0.813355
C         -2.324024       1.314110      -0.388218
C         -1.175761       2.301979      -0.553776
H          2.404311      -2.136202       0.436373
H          1.387989      -0.897390       1.307217
H         -2.981650      -0.282388       0.914791
H         -2.053082       0.993994       1.725511
H         -2.425833       0.697802      -1.282788
H         -3.268059       1.848647      -0.258908
H         -0.232614       1.777483      -0.714712
H         -1.065849       2.921170       0.339108
H         -1.346907       2.963622      -1.402692
S          2.540597       0.311577      -0.620267
C          2.209316       1.448457       0.758379
H          1.182972       1.321408       1.106926
H          2.885009       1.266724       1.594503
H          2.334915       2.483338       0.441105
H          0.436157      -2.883612      -0.851954
```

---

n-propylacrylate_TS_3_UNCON_m062x

Datum	Value
M06-2X/def2tzvpp-IEFPCM(water) Energy	-823.296424
M06-2X/def2tzvpp-IEFPCM(water) Free Energy (Quasiharmonic)	-823.14028
Number of Imaginary Frequencies	1

Frequencies (Top 3 out of 63)

```
1.    -202.9832 cm-1
2.      34.7653 cm-1
3.      46.0316 cm-1
```

M06-2X/def2tzvpp-IEFPCM(water) Molecular Geometry in Cartesian Coordinates

```
C        -0.281936       1.580021       0.090925
C         1.047510       1.662135      -0.428224
C         1.583942       0.727445      -1.290714
O        -0.810483       2.378816       0.860248
O        -0.972431       0.493484      -0.350273
C        -2.291793       0.322452       0.158728
C        -2.853791      -0.955359      -0.427132
C        -2.064863      -2.191706      -0.014546
H         2.518377       0.950507      -1.784011
H         0.917273       0.082251      -1.840588
H        -2.252533       0.266201       1.249446
H        -2.902161       1.185566      -0.110463
H        -3.890356      -1.043734      -0.095903
H        -2.875299      -0.866339      -1.515423
H        -2.510629      -3.097395      -0.424370
H        -2.042893      -2.288605       1.072555
H        -1.034770      -2.131536      -0.366117
S         2.533243      -1.138847      -0.165629
C         1.758013      -0.823392       1.430153
H         0.927211      -1.502013       1.623926
H         2.475814      -0.907932       2.244791
H         1.367765       0.202480       1.427571
H         1.651127       2.464115      -0.026292
```

---

n-propylacrylate_TS_4_UNCON_m062x

Datum	Value
M06-2X/def2tzvpp-IEFPCM(water) Energy	-823.295637
M06-2X/def2tzvpp-IEFPCM(water) Free Energy (Quasiharmonic)	-823.139891
Number of Imaginary Frequencies	1

Frequencies (Top 3 out of 63)

```
1.    -199.8551 cm-1
2.      27.6314 cm-1
3.      47.7665 cm-1
```

M06-2X/def2tzvpp-IEFPCM(water) Molecular Geometry in Cartesian Coordinates

```
C          -0.226691        1.461752       -0.130895
C           0.992785        1.372390       -0.872147
C           1.425844        0.216133       -1.487902
O          -0.667538        2.467998        0.418470
O          -0.915740        0.289891       -0.071665
C          -2.145213        0.304567        0.648044
C          -2.728356       -1.091509        0.598231
C          -3.063911       -1.540670       -0.818179
H           2.244184        0.275127       -2.189892
H           0.714628       -0.572617       -1.676251
H          -1.959968        0.620995        1.675494
H          -2.824998        1.027925        0.191319
H          -2.021431       -1.785998        1.057709
H          -3.627540       -1.097957        1.217349
H          -3.505089       -2.536735       -0.821419
H          -2.168917       -1.564388       -1.439139
H          -3.776031       -0.854943       -1.280773
S           2.688213       -1.195977       -0.043754
C           2.197336       -0.399917        1.496557
H           1.714589        0.555374        1.252435
H           1.488654       -1.003646        2.063024
H           3.058309       -0.188673        2.129183
H           1.607657        2.261578       -0.855653
```

---

n-propylacrylate_TS_5_UNCON_m062x

Datum	Value
M06-2X/def2tzvpp-IEFPCM(water) Energy	-823.295437
M06-2X/def2tzvpp-IEFPCM(water) Free Energy (Quasiharmonic)	-823.139675
Number of Imaginary Frequencies	1

Frequencies (Top 3 out of 63)

```
1.    -198.9504 cm-1
2.      30.1126 cm-1
3.      43.5154 cm-1
```

M06-2X/def2tzvpp-IEFPCM(water) Molecular Geometry in Cartesian Coordinates

```
C          -0.205262        1.364325        0.291273
C           1.207636        1.580940        0.267481
C           2.037524        1.104209       -0.726355
```

```
O          -0.980837        1.767714        1.153414
O          -0.664265        0.648971       -0.776143
C          -2.049012        0.314747       -0.785356
C          -2.333074       -0.921834        0.046894
C          -3.811844       -1.284690        0.006747
H           3.051962        1.470560       -0.776249
H           1.607266        0.811215       -1.670749
H          -2.636024        1.160519       -0.427742
H          -2.296015        0.133643       -1.831537
H          -1.728479       -1.747250       -0.334810
H          -2.014745       -0.733439        1.073526
H          -4.014489       -2.180794        0.591426
H          -4.140515       -1.470979       -1.016998
H          -4.421937       -0.475379        0.410614
S           2.792096       -1.119225       -0.320855
C           1.680507       -1.472744        1.052289
H           0.941210       -2.230662        0.795068
H           1.145869       -0.548153        1.309497
H           2.224589       -1.802904        1.936177
H           1.609735        2.078018        1.139440
```

---

n-propylacrylate_TS_6_UNCON_m062x

Datum	Value
M06-2X/def2tzvpp-IEFPCM(water) Energy	-823.295034
M06-2X/def2tzvpp-IEFPCM(water) Free Energy (Quasiharmonic)	-823.138743
Number of Imaginary Frequencies	1

Frequencies (Top 3 out of 63)

```
1.    -196.3583 cm-1
2.      38.0449 cm-1
3.      46.1265 cm-1
```

M06-2X/def2tzvpp-IEFPCM(water) Molecular Geometry in Cartesian Coordinates

```
C           0.395011        0.969357        0.461563
C          -0.705216        0.666809        1.324287
C          -1.301321       -0.574498        1.395135
O           0.975864        2.048407        0.389133
O           0.777373       -0.079815       -0.320170
C           1.940274        0.099173       -1.127128
C           3.216241       -0.120813       -0.333691
```

```
C          3.291348      -1.513316       0.279546
H         -1.982973      -0.779295       2.206955
H         -0.772397      -1.431478       1.008842
H          1.851134      -0.647861      -1.916239
H          1.924848       1.091880      -1.573757
H          4.059652       0.039373      -1.009150
H          3.280917       0.641236       0.444366
H          3.242952      -2.281266      -0.494594
H          2.461690      -1.678621       0.967100
H          4.219899      -1.650674       0.832586
S         -3.045736      -0.867213      -0.212977
C         -2.643664       0.578340      -1.210740
H         -1.908234       1.182074      -0.662671
H         -2.209663       0.304976      -2.172128
H         -3.520964       1.198542      -1.389387
H         -1.097030       1.507477       1.880177
```

---

n-propylacrylate_TS_7_UNCON_m062x_reopt

Datum	Value
M06-2X/def2tzvpp-IEFPCM(water) Energy	-823.295147
M06-2X/def2tzvpp-IEFPCM(water) Free Energy (Quasiharmonic)	-823.138847
Number of Imaginary Frequencies	1

Frequencies (Top 3 out of 63)

```
1.    -200.9284 cm-1
2.      27.8511 cm-1
3.      50.9087 cm-1
```

M06-2X/def2tzvpp-IEFPCM(water) Molecular Geometry in Cartesian Coordinates

```
C          0.500500      -1.300919       0.353451
C         -0.890999      -1.621721       0.445652
C         -1.811325      -1.335925      -0.541629
O          1.360245      -1.601430       1.175795
O          0.819994      -0.615225      -0.781046
C          2.184416      -0.268636      -1.015528
C          2.432749       1.195005      -0.699710
C          2.320981       1.515419       0.784871
H         -2.785094      -1.800033      -0.493729
H         -1.458059      -1.109494      -1.535008
H          2.831270      -0.910844      -0.420052
```

```
H          2.362227      -0.464957      -2.072618
H          3.429917       1.451119      -1.065254
H          1.722022       1.797753      -1.270341
H          2.473821       2.579047       0.967474
H          3.064088       0.959221       1.357168
H          1.336664       1.244860       1.167212
S         -2.774038       0.833654      -0.336104
C         -1.561368       1.474198       0.830651
H         -0.871613       2.177201       0.363398
H         -2.039340       1.965003       1.677448
H         -0.975473       0.628340       1.215287
H         -1.197465      -2.066967       1.382130
```

---

n-propylacrylate_TS_8_UNCON_m062x

Datum	Value
M06-2X/def2tzvpp-IEFPCM(water) Energy	-823.295477
M06-2X/def2tzvpp-IEFPCM(water) Free Energy (Quasiharmonic)	-823.139998
Number of Imaginary Frequencies	1

Frequencies (Top 3 out of 63)

```
1.    -197.4896 cm-1
2.      36.2689 cm-1
3.      50.5428 cm-1
```

M06-2X/def2tzvpp-IEFPCM(water) Molecular Geometry in Cartesian Coordinates

```
C         -0.048062       1.454952       0.091117
C          1.281887       1.517457      -0.431079
C          1.807057       0.582081      -1.297741
O         -0.565258       2.271097       0.848936
O         -0.754444       0.371613      -0.334624
C         -2.090541       0.255073       0.143359
C         -2.690671      -1.004776      -0.441093
C         -4.127895      -1.196610       0.025571
H          2.735682       0.803064      -1.802283
H          1.135607      -0.069318      -1.834259
H         -2.086903       0.215248       1.235014
H         -2.665595       1.135207      -0.152903
H         -2.652029      -0.944083      -1.530370
H         -2.078946      -1.859433      -0.145472
H         -4.560015      -2.102850      -0.395868
```

```
H          -4.751761       -0.354728       -0.277996
H          -4.178139       -1.274650        1.112511
S           2.794736       -1.279326       -0.176872
C           2.081154       -0.940708        1.442617
H           1.297102       -1.651664        1.702288
H           2.838644       -0.957773        2.225019
H           1.639070        0.064156        1.421818
H           1.891548        2.319544       -0.038539
```

---

n-propylacrylate_TS_9_UNCON_m062x

Datum	Value
M06-2X/def2tzvpp-IEFPCM(water) Energy	-823.294998
M06-2X/def2tzvpp-IEFPCM(water) Free Energy (Quasiharmonic)	-823.139103
Number of Imaginary Frequencies	1

Frequencies (Top 3 out of 63)

```
1.    -197.5854 cm-1
2.      38.8321 cm-1
3.      43.0094 cm-1
```

M06-2X/def2tzvpp-IEFPCM(water) Molecular Geometry in Cartesian Coordinates

```
C          -0.266920        0.982587        0.304347
C           0.958525        1.486860       -0.234834
C           1.674979        0.853565       -1.227722
O          -0.946884        1.523244        1.171556
O          -0.652187       -0.207173       -0.239437
C          -1.899765       -0.744597        0.190817
C          -3.068817       -0.144552       -0.567778
C          -4.386775       -0.766225       -0.123942
H           2.474569        1.389891       -1.716344
H           1.186133        0.104773       -1.830293
H          -1.833140       -1.815719       -0.000593
H          -2.018053       -0.586399        1.262784
H          -3.080993        0.933044       -0.398857
H          -2.914666       -0.306088       -1.636544
H          -4.384051       -1.845107       -0.287454
H          -5.226762       -0.346354       -0.675311
H          -4.561386       -0.590597        0.938570
S           3.213322       -0.770619       -0.378778
C           2.533041       -0.837045        1.288541
```

```
H          1.827201       -0.004392        1.407101
H          1.995898       -1.766452        1.475876
H          3.311036       -0.727195        2.042830
H          1.338017        2.378382        0.245161
```

Created using ESIgen v0.0.5

ESIgen is scientific software, funded by public research grants and published as:

```
J Rodriguez-Guerra, P Gomez-Orellana, JD Marechal.
J. Chem. Inf. Model., 2018, 58 (3), pp 561564.
DOI: 10.1021/acs.jcim.7b00714.
```

If you make use of ESIgen in scientific publications, please cite us in the main text! References only mentioned in SI documents are not indexed by citation engines.

1_pentene-3-one_truncated_1

Datum	Value
M06-2X/def2tzvpp-IEFPCM(water) Energy	-231.227508
M06-2X/def2tzvpp-IEFPCM(water) Free Energy (Quasiharmonic)	-231.162782
Number of Imaginary Frequencies	0

Frequencies (Top 3 out of 27)

```
1.     123.2502 cm-1
2.     159.7787 cm-1
3.     300.6150 cm-1
```

M06-2X/def2tzvpp-IEFPCM(water) Molecular Geometry in Cartesian Coordinates

```
C        -0.826853        1.293812        0.000000
H        -0.384491        1.763114        0.878963
H        -1.900669        1.453387       -0.000001
C        -0.542713       -0.184126       -0.000002
H        -0.384489        1.763116       -0.878961
C         0.866606       -0.648954        0.000001
O        -1.440894       -1.001993       -0.000006
C         1.916006        0.163733        0.000006
H         2.925190       -0.225188        0.000008
H         1.806529        1.240522        0.000008
H         0.986797       -1.725800       -0.000001
```

1_pentene-3-one_truncated_2

Datum	Value
M06-2X/def2tzvpp-IEFPCM(water) Energy	-231.226386
M06-2X/def2tzvpp-IEFPCM(water) Free Energy (Quasiharmonic)	-231.162235
Number of Imaginary Frequencies	0

Frequencies (Top 3 out of 27)

```
1.      69.2637 cm-1
2.     141.8725 cm-1
3.     276.6380 cm-1
```

M06-2X/def2tzvpp-IEFPCM(water) Molecular Geometry in Cartesian Coordinates

```
C          1.163030        1.423480        0.000000
H          1.785624        1.234348        0.876313
H          0.818211        2.452875        0.000000
C          0.000000        0.473637        0.000000
H          1.785624        1.234348       -0.876313
C          0.342042       -0.979598        0.000000
O         -1.149435        0.859046        0.000000
C         -0.601670       -1.910979        0.000000
H         -0.360685       -2.964942        0.000000
H         -1.647057       -1.628325        0.000000
H          1.393353       -1.239908        0.000000
```

1_pentene-3-one_trunc_HEI_1

Datum	Value
M06-2X/def2tzvpp-IEFPCM(water) Energy	-669.451211
M06-2X/def2tzvpp-IEFPCM(water) Free Energy (Quasiharmonic)	-669.351765
Number of Imaginary Frequencies	0

Frequencies (Top 3 out of 42)

```
1.      78.4530 cm-1
2.      97.4899 cm-1
```

```
   3.     109.7831 cm-1
```

M06-2X/def2tzvpp-IEFPCM(water) Molecular Geometry in Cartesian Coordinates

```
C         -1.594231       -0.023062        0.130894
C         -0.610517       -0.929647       -0.191544
C          0.642447       -1.045653        0.587993
O         -1.574597        0.800674        1.109540
H         -0.734764       -1.562413       -1.061618
H          0.526936       -0.580426        1.566424
H          0.967820       -2.077190        0.733976
C          1.548936        1.430218       -0.314502
H          1.527528        1.909009        0.662677
H          0.545070        1.422696       -0.737351
H          2.222877        1.976256       -0.970762
S          2.135787       -0.267989       -0.190811
C         -2.842004        0.013255       -0.741619
H         -2.814542       -0.710232       -1.556016
H         -3.723149       -0.180233       -0.125727
H         -2.961375        1.014301       -1.162277
```

1_pentene-3-one_trunc_HEI_2

Datum	Value
M06-2X/def2tzvpp-IEFPCM(water) Energy	-669.447746
M06-2X/def2tzvpp-IEFPCM(water) Free Energy (Quasiharmonic)	-669.34827
Number of Imaginary Frequencies	0

Frequencies (Top 3 out of 42)

```
   1.      74.9024 cm-1
   2.     100.1561 cm-1
   3.     118.2623 cm-1
```

M06-2X/def2tzvpp-IEFPCM(water) Molecular Geometry in Cartesian Coordinates

```
C         -1.686191       -0.160400       -0.099270
C         -0.629063       -1.042791       -0.089567
C          0.599687       -0.969265        0.732702
```

```
O          -2.714486       -0.265428       -0.855720
H          -0.675994       -1.857262       -0.807411
H           0.479182       -0.381133        1.640473
H           0.952098       -1.956492        1.035605
C           1.495479        1.381963       -0.472282
H           1.411445        1.969087        0.441097
H           0.518933        1.311914       -0.951017
H           2.196621        1.866745       -1.147663
S           2.103006       -0.276121       -0.124690
C          -1.709348        1.042184        0.837142
H          -2.608299        0.990265        1.454376
H          -0.843190        1.137729        1.487543
H          -1.786393        1.950357        0.235451
```

1_pentene-3-one_trunc_HEI_3

Datum	Value
M06-2X/def2tzvpp-IEFPCM(water) Energy	-669.450513
M06-2X/def2tzvpp-IEFPCM(water) Free Energy (Quasiharmonic)	-669.351422
Number of Imaginary Frequencies	0

Frequencies (Top 3 out of 42)

```
 1.      60.3905 cm-1
 2.      87.2277 cm-1
 3.     101.0128 cm-1
```

M06-2X/def2tzvpp-IEFPCM(water) Molecular Geometry in Cartesian Coordinates

```
C          -1.722719       -0.060916        0.002746
C          -0.601697        0.389393        0.662119
C           0.536654       -0.512517        0.946590
O          -1.907187       -1.251517       -0.424716
H          -0.539071        1.425773        0.966591
H           1.045705       -0.272038        1.881248
H           0.202268       -1.549367        0.989129
C           2.391805        1.162451       -0.259742
H           2.792193        1.401440        0.724954
H           1.545383        1.812468       -0.475406
H           3.165688        1.320526       -1.007327
S           1.877581       -0.561970       -0.336279
C          -2.858009        0.922360       -0.243363
H          -2.649615        1.918308        0.146582
```

```
H          -3.049244        0.996043       -1.316361
H          -3.773308        0.545881        0.218680
```

1_pentene-3-one_trunc_HEI_4

Datum	Value
M06-2X/def2tzvpp-IEFPCM(water) Energy	-669.446335
M06-2X/def2tzvpp-IEFPCM(water) Free Energy (Quasiharmonic)	-669.347804
Number of Imaginary Frequencies	0

Frequencies (Top 3 out of 42)

```
1.      42.4781 cm-1
2.      76.7734 cm-1
3.      95.7518 cm-1
```

M06-2X/def2tzvpp-IEFPCM(water) Molecular Geometry in Cartesian Coordinates

```
C           1.757821        0.242688        0.013807
C           0.606662        0.570038        0.695576
C          -0.496293       -0.350136        1.064826
O           2.688490        1.065590       -0.291624
H           0.475570        1.616171        0.954720
H          -0.166498       -1.361263        1.300081
H          -1.040091        0.018571        1.935444
C          -2.327146        1.059017       -0.485315
H          -1.470202        1.664228       -0.776688
H          -2.771230        1.469740        0.420718
H          -3.066116        1.071686       -1.282975
S          -1.808446       -0.645424       -0.226252
C           1.995109       -1.193783       -0.437272
H           2.926222       -1.557450        0.002304
H           1.193527       -1.886415       -0.189554
H           2.129116       -1.200156       -1.520750
```

1_pentene-3-one_trunc_HEI_5

Datum	Value

Datum	Value
M06-2X/def2tzvpp-IEFPCM(water) Energy	-669.450723
M06-2X/def2tzvpp-IEFPCM(water) Free Energy (Quasiharmonic)	-669.351983
Number of Imaginary Frequencies	0

Frequencies (Top 3 out of 42)

```
1.      60.0177 cm-1
2.      82.6555 cm-1
3.      88.1153 cm-1
```

M06-2X/def2tzvpp-IEFPCM(water) Molecular Geometry in Cartesian Coordinates

```
C          1.839392        0.161371        0.057582
C          0.837178       -0.691971        0.458993
C         -0.514698       -0.194370        0.814903
O          1.744500        1.431484       -0.055193
H          1.024194       -1.757160        0.499193
H         -0.982326       -0.766771        1.617966
H         -0.471022        0.855197        1.107174
C         -3.177773        0.349517        0.154733
H         -3.023931        1.367964        0.508321
H         -3.478431       -0.281438        0.989999
H         -3.969114        0.349343       -0.591586
S         -1.674267       -0.289029       -0.614624
C          3.197099       -0.439294       -0.278061
H          3.223710       -1.522382       -0.161586
H          3.461403       -0.188040       -1.307668
H          3.960590        0.004364        0.364805
```

1_pentene-3-one_trunc_HEI_6

Datum	Value
M06-2X/def2tzvpp-IEFPCM(water) Energy	-669.447007
M06-2X/def2tzvpp-IEFPCM(water) Free Energy (Quasiharmonic)	-669.348535
Number of Imaginary Frequencies	0

Frequencies (Top 3 out of 42)

```
1.      54.9921 cm-1
2.      70.9232 cm-1
3.      93.9559 cm-1
```

M06-2X/def2tzvpp-IEFPCM(water) Molecular Geometry in Cartesian Coordinates

```
C         -1.940682       -0.094426        0.015405
C         -0.868079       -0.903273        0.314278
C          0.475207       -0.453312        0.766411
O         -3.088507       -0.508935       -0.369362
H         -1.001294       -1.971317        0.172064
H          0.449971        0.450801        1.374671
H          0.977788       -1.225798        1.350268
C          3.120241        0.307493        0.231727
H          3.455235       -0.545933        0.819449
H          2.969622        1.162686        0.888985
H          3.885164        0.554928       -0.500957
S          1.596600       -0.089227       -0.653596
C         -1.810860        1.419251        0.142248
H         -2.501450        1.777038        0.909135
H         -0.808390        1.769814        0.379773
H         -2.119151        1.872501       -0.801362
```

1_pentene-3-one_trunc_TS_1

Datum	Value
M06-2X/def2tzvpp-IEFPCM(water) Energy	-669.435969
M06-2X/def2tzvpp-IEFPCM(water) Free Energy (Quasiharmonic)	-669.339302
Number of Imaginary Frequencies	1

Frequencies (Top 3 out of 42)

```
1.    -164.8676 cm-1
2.      37.8484 cm-1
3.      42.9396 cm-1
```

M06-2X/def2tzvpp-IEFPCM(water) Molecular Geometry in Cartesian Coordinates

```
C          -1.637710       -0.081614        0.166756
C          -0.748198       -0.761492       -0.744861
C           0.275907       -1.536693       -0.289553
O          -1.531973       -0.140338        1.394136
H          -0.879383       -0.583505       -1.804384
H           0.305754       -1.815231        0.752627
H           0.861027       -2.137093       -0.968467
C           1.505753        1.478306       -0.188754
H           1.444453        2.108178        0.698768
H           0.479639        1.267805       -0.521835
H           2.000914        2.044039       -0.977623
S           2.346864       -0.082196        0.148695
C          -2.776495        0.712847       -0.438302
H          -2.531715        1.089273       -1.429800
H          -3.646739        0.059292       -0.532962
H          -3.043544        1.536956        0.219748
```

1_pentene-3-one_trunc_TS_2

Datum	Value
M06-2X/def2tzvpp-IEFPCM(water) Energy	-669.437096
M06-2X/def2tzvpp-IEFPCM(water) Free Energy (Quasiharmonic)	-669.339121
Number of Imaginary Frequencies	1

Frequencies (Top 3 out of 42)

```
  1.    -191.8724 cm-1
  2.      68.2150 cm-1
  3.      92.3069 cm-1
```

M06-2X/def2tzvpp-IEFPCM(water) Molecular Geometry in Cartesian Coordinates

```
C          -1.705684        0.002919       -0.092905
C          -0.777795       -0.907357       -0.691104
C           0.244610       -1.511648        0.006323
O          -2.645096        0.517540       -0.717424
H          -0.868631       -1.050119       -1.761779
H           0.210335       -1.558282        1.083584
H           0.810213       -2.302405       -0.463600
C           1.439205        1.394867       -0.461241
H           1.342290        2.160503        0.308369
H           0.431811        1.140302       -0.817378
```

```
H        1.992293     1.814391    -1.300582
S        2.218989    -0.102256     0.168603
C       -1.512467     0.353335     1.370608
H       -1.710948    -0.515085     2.001288
H       -0.481419     0.661001     1.558003
H       -2.196204     1.152780     1.643745
```

1_pentene-3-one_trunc_TS_3_reopt

Datum	Value
M06-2X/def2tzvpp-IEFPCM(water) Energy	-669.435981
M06-2X/def2tzvpp-IEFPCM(water) Free Energy (Quasiharmonic)	-669.339242
Number of Imaginary Frequencies	1

Frequencies (Top 3 out of 42)

```
1.   -162.5073 cm-1
2.     40.8243 cm-1
3.     51.5580 cm-1
```

M06-2X/def2tzvpp-IEFPCM(water) Molecular Geometry in Cartesian Coordinates

```
C        1.644765    -0.108756     0.163923
C        0.751163    -0.773832    -0.754900
C       -0.281756    -1.541789    -0.307523
O        1.541082    -0.183479     1.390375
H        0.889453    -0.591637    -1.812927
H       -0.872728    -2.129833    -0.992139
H       -0.315100    -1.829012     0.732158
C       -1.482476     1.482427    -0.171534
H       -1.979955     2.068135    -0.944208
H       -0.464565     1.262932    -0.521481
H       -1.400970     2.096584     0.725422
S       -2.341940    -0.071946     0.151212
C        2.745190     0.744554    -0.432246
H        3.032788     0.407802    -1.426362
H        2.382027     1.771445    -0.516996
H        3.610129     0.746917     0.227813
```

1_pentene-3-one_trunc_TS_4

Datum	Value
M06-2X/def2tzvpp-IEFPCM(water) Energy	-669.437096
M06-2X/def2tzvpp-IEFPCM(water) Free Energy (Quasiharmonic)	-669.339121
Number of Imaginary Frequencies	1

Frequencies (Top 3 out of 42)

```
1.    -191.6744 cm-1
2.      68.2909 cm-1
3.      92.3051 cm-1
```

M06-2X/def2tzvpp-IEFPCM(water) Molecular Geometry in Cartesian Coordinates

```
C          1.705667        0.002977        0.092882
C          0.777803       -0.907373        0.691114
C         -0.244448       -1.511890       -0.006257
O          2.644996        0.517635        0.717445
H          0.868700       -1.050115        1.761787
H         -0.210360       -1.558488       -1.083521
H         -0.810060       -2.302577        0.463782
C         -1.439021        1.394779        0.461295
H         -0.431847        1.139885        0.817835
H         -1.992225        1.814789        1.300314
H         -1.341471        2.160177       -0.308480
S         -2.219194       -0.102122       -0.168648
C          1.512570        0.353303       -1.370656
H          1.711605       -0.515050       -2.001253
H          0.481388        0.660414       -1.558226
H          2.195974        1.153061       -1.643706
```

1_pentene-3-one_trunc_TS_5_reopt2

Datum	Value
M06-2X/def2tzvpp-IEFPCM(water) Energy	-669.431086
M06-2X/def2tzvpp-IEFPCM(water) Free Energy (Quasiharmonic)	-669.335612
Number of Imaginary Frequencies	1

Frequencies (Top 3 out of 42)

```
1.    -195.1898 cm-1
2.      17.8017 cm-1
3.      43.6376 cm-1
```

M06-2X/def2tzvpp-IEFPCM(water) Molecular Geometry in Cartesian Coordinates

```
C          1.966469        0.186865        0.060046
C          1.037753       -0.842938        0.442568
C         -0.218811       -0.531550        0.879482
O          1.690195        1.391013        0.059939
H          1.339271       -1.874280        0.317517
H         -0.864703       -1.290307        1.295789
H         -0.441714        0.490551        1.151009
C         -3.104427        0.446796        0.503037
H         -2.947093        1.516158        0.646199
H         -2.875488       -0.053122        1.448009
H         -4.160814        0.285758        0.291412
S         -2.056571       -0.211103       -0.827613
C          3.355258       -0.237109       -0.372009
H          3.523541       -1.305616       -0.260331
H          3.498066        0.037779       -1.418280
H          4.095043        0.310228        0.212234
```

1_pentene-3-one_trunc_TS_6_reopt

Datum	Value
M06-2X/def2tzvpp-IEFPCM(water) Energy	-669.431731
M06-2X/def2tzvpp-IEFPCM(water) Free Energy (Quasiharmonic)	-669.335181
Number of Imaginary Frequencies	1

Frequencies (Top 3 out of 42)

```
1.    -224.1020 cm-1
2.      41.4689 cm-1
3.      63.7765 cm-1
```

M06-2X/def2tzvpp-IEFPCM(water) Molecular Geometry in Cartesian Coordinates

```
C        -2.086146      -0.055435      -0.004248
C        -1.045632      -0.986959       0.295902
C         0.188533      -0.618016       0.782486
O        -3.189715      -0.402559      -0.450947
H        -1.241795      -2.022057       0.041391
H         0.329603       0.359155       1.222598
H         0.862545      -1.378304       1.151112
C         3.140840       0.228713       0.499499
H         2.647370       0.261262       1.475617
H         3.689822       1.161246       0.376397
H         3.858687      -0.590566       0.516788
S         1.899082       0.008891      -0.806509
C        -1.822616       1.420383       0.232738
H        -1.692508       1.621292       1.297348
H        -0.906668       1.734454      -0.270582
H        -2.664516       1.999628      -0.137210
```

2_2-cyclopentene-1-one_1

Datum	Value
M06-2X/def2tzvpp-IEFPCM(water) Energy	-269.343244
M06-2X/def2tzvpp-IEFPCM(water) Free Energy (Quasiharmonic)	-269.270219
Number of Imaginary Frequencies	0

Frequencies (Top 3 out of 30)

```
1.      95.3968 cm-1
2.     295.9541 cm-1
3.     471.7202 cm-1
```

M06-2X/def2tzvpp-IEFPCM(water) Molecular Geometry in Cartesian Coordinates

```
C        -0.049804      -1.192797       0.000000
C        -1.468512      -0.616104      -0.000000
C        -1.257968       0.871505       0.000000
C         0.028018       1.224371       0.000000
C         0.872978       0.016502      -0.000000
H         0.164467      -1.801338       0.877858
H         0.164467      -1.801339      -0.877857
H        -2.046271      -0.918386       0.874408
H        -2.046270      -0.918385      -0.874409
O         2.084282      -0.021394      -0.000001
```

```
H          0.430654        2.226134        0.000000
H         -2.089572        1.563599        0.000000
```

2_2cyclopentene1one_HEI_1

Datum	Value
M06-2X/def2tzvpp-IEFPCM(water) Energy	-707.560008
M06-2X/def2tzvpp-IEFPCM(water) Free Energy (Quasiharmonic)	-707.451882
Number of Imaginary Frequencies	0

Frequencies (Top 3 out of 45)

```
  1.      90.4697 cm-1
  2.     116.9776 cm-1
  3.     163.3776 cm-1
```

M06-2X/def2tzvpp-IEFPCM(water) Molecular Geometry in Cartesian Coordinates

```
C          1.337131        0.627083        0.980639
C          0.180697        1.500371        0.486253
C         -0.401709        0.757764       -0.739318
C          0.685877       -0.159908       -1.152951
C          1.669749       -0.293778       -0.199973
H          2.214570        1.208385        1.269424
H          1.061895        0.012797        1.843000
H          0.563229        2.465567        0.150488
H         -0.575477        1.697263        1.245981
O          2.709759       -1.025590       -0.179143
H          0.644392       -0.743357       -2.064572
H         -0.733623        1.459233       -1.507666
C         -1.509903       -1.150970        1.009142
H         -1.348491       -0.574797        1.918868
H         -0.589782       -1.666353        0.733726
H         -2.295077       -1.882478        1.185034
S         -2.024422       -0.090932       -0.352743
```

2_2cyclopentene1one_HEI_2

Datum	Value

Datum	Value
M06-2X/def2tzvpp-IEFPCM(water) Energy	-707.55707
M06-2X/def2tzvpp-IEFPCM(water) Free Energy (Quasiharmonic)	-707.449835
Number of Imaginary Frequencies	0

Frequencies (Top 3 out of 45)

```
1.      61.8777 cm-1
2.      89.0043 cm-1
3.     144.2390 cm-1
```

M06-2X/def2tzvpp-IEFPCM(water) Molecular Geometry in Cartesian Coordinates

```
C         -1.996467        0.971164        0.107697
C         -0.562570        1.505519        0.109368
C          0.319157        0.293679        0.483511
C         -0.531719       -0.884896        0.156037
C         -1.847411       -0.547099       -0.054807
H         -2.511526        1.172872        1.051782
H         -2.611606        1.391909       -0.689499
H         -0.402164        2.349493        0.779386
H         -0.298832        1.834446       -0.898453
O         -2.864052       -1.273261       -0.298194
H         -0.171597       -1.905276        0.199607
H          0.627179        0.341834        1.534760
C          2.767331       -1.002656        0.242993
H          2.838421       -0.923485        1.327003
H          3.770496       -1.023243       -0.176821
H          2.251983       -1.923648       -0.022714
S          1.908133        0.428558       -0.436768
```

2_2cyclopentene1one_HEI_3

Datum	Value
M06-2X/def2tzvpp-IEFPCM(water) Energy	-707.557663
M06-2X/def2tzvpp-IEFPCM(water) Free Energy (Quasiharmonic)	-707.450409
Number of Imaginary Frequencies	0

Frequencies (Top 3 out of 45)

```
1.      70.5385 cm-1
2.      78.4695 cm-1
3.     138.2238 cm-1
```

M06-2X/def2tzvpp-IEFPCM(water) Molecular Geometry in Cartesian Coordinates

```
C          -1.679958        1.234260       -0.090144
C          -0.149286        1.282289       -0.104711
C           0.302710       -0.066390        0.499907
C          -0.885375       -0.953372        0.327055
C          -2.026144       -0.257017        0.005318
H          -2.099899        1.744433        0.782120
H          -2.134212        1.686269       -0.973778
H           0.266977        2.133818        0.433532
H           0.209026        1.337075       -1.134028
O          -3.223446       -0.655187       -0.158644
H          -0.864290       -2.012045        0.554849
H           0.617515        0.061545        1.543437
C           3.010265        0.479215        0.134645
H           2.768133        1.445621       -0.304128
H           3.978411        0.153106       -0.238333
H           3.061722        0.574774        1.218560
S           1.784432       -0.762313       -0.327844
```

2_2cyclopentene1one_TS_1_reopt

Datum	Value
M06-2X/def2tzvpp-IEFPCM(water) Energy	-707.549412
M06-2X/def2tzvpp-IEFPCM(water) Free Energy (Quasiharmonic)	-707.443019
Number of Imaginary Frequencies	1

Frequencies (Top 3 out of 45)

```
1.    -205.8851 cm-1
2.      57.7381 cm-1
3.      86.6377 cm-1
```

M06-2X/def2tzvpp-IEFPCM(water) Molecular Geometry in Cartesian Coordinates

```
C          -1.336190        0.561284       -1.033043
C          -0.504613        1.663060       -0.376610
C           0.055911        0.972853        0.850506
C          -0.756969       -0.098281        1.182301
C          -1.640107       -0.408173        0.107413
H          -2.249207        0.901972       -1.518589
H          -0.739050        0.021102       -1.774376
H          -1.152972        2.479831       -0.045500
H           0.264445        2.081378       -1.019068
O          -2.506917       -1.288695        0.040741
H          -0.703302       -0.676736        2.093599
H           0.610284        1.539837        1.582369
C           1.625754       -1.510764       -0.418550
H           1.536918       -1.653168       -1.495531
H           0.636858       -1.671785        0.028144
H           2.300271       -2.269668       -0.023804
S           2.180523        0.154808       -0.001580
```

2_2cyclopentene1one_TS_2

Datum	Value
M06-2X/def2tzvpp-IEFPCM(water) Energy	-707.549412
M06-2X/def2tzvpp-IEFPCM(water) Free Energy (Quasiharmonic)	-707.443018
Number of Imaginary Frequencies	1

Frequencies (Top 3 out of 45)

```
1.    -205.9218 cm-1
2.      57.8145 cm-1
3.      86.6484 cm-1
```

M06-2X/def2tzvpp-IEFPCM(water) Molecular Geometry in Cartesian Coordinates

```
C          -1.336086        0.561259       -1.033107
C          -0.504475        1.663028       -0.376708
C           0.055954        0.972842        0.850468
C          -0.757034       -0.098218        1.182314
C          -1.640135       -0.408116        0.107386
H          -2.249048        0.901980       -1.518732
H          -0.738944        0.020983       -1.774373
H          -1.152774        2.479875       -0.045667
H           0.264632        2.081250       -1.019171
```

```
O         -2.507015      -1.288575       0.040709
H         -0.703457      -0.676610       2.093658
H          0.610279       1.539827       1.582369
C          1.625631      -1.510684      -0.418855
H          2.300275      -2.269764      -0.024670
H          1.536453      -1.652585      -1.495878
H          0.636875      -1.671899       0.028075
S          2.180544       0.154680      -0.001267
```

2_2cyclopentene1one_TS_3

Datum	Value
M06-2X/def2tzvpp-IEFPCM(water) Energy	-707.546155
M06-2X/def2tzvpp-IEFPCM(water) Free Energy (Quasiharmonic)	-707.440464
Number of Imaginary Frequencies	1

Frequencies (Top 3 out of 45)

```
1.    -240.3974 cm-1
2.      65.5299 cm-1
3.      86.6001 cm-1
```

M06-2X/def2tzvpp-IEFPCM(water) Molecular Geometry in Cartesian Coordinates

```
C         -1.424525       1.080435      -0.601664
C         -0.221742       1.337753       0.305501
C          0.042175      -0.019608       0.921248
C         -1.102650      -0.792741       0.839582
C         -2.041330      -0.205741      -0.055255
H         -2.154220       1.888316      -0.626734
H         -1.104616       0.886496      -1.629272
H         -0.494306       2.027231       1.109900
H          0.643690       1.750960      -0.206210
O         -3.161180      -0.616024      -0.384733
H         -1.253767      -1.749340       1.318688
H          0.768502      -0.117189       1.715675
C          3.019008       0.444515       0.070299
H          3.110883       1.269582      -0.635806
H          4.009162       0.018810       0.227894
H          2.681055       0.858968       1.025102
S          1.841090      -0.810832      -0.506228
```

3_3methyl3pentene2one_HEI_1

Datum	Value
M06-2X/def2tzvpp-IEFPCM(water) Energy	-748.063725
M06-2X/def2tzvpp-IEFPCM(water) Free Energy (Quasiharmonic)	-747.911649
Number of Imaginary Frequencies	0

Frequencies (Top 3 out of 60)

```
1.      66.3362 cm-1
2.      72.0385 cm-1
3.     107.1973 cm-1
```

M06-2X/def2tzvpp-IEFPCM(water) Molecular Geometry in Cartesian Coordinates

```
C         -1.630516       -0.247313       -0.325560
C         -0.600481        0.555849        0.112707
C          0.692358        0.502380       -0.626557
O         -1.562519       -1.070521       -1.313654
H          0.537805       -0.049345       -1.551347
C          1.201485       -2.023450        0.517199
H          1.192187       -2.605977       -0.401827
H          1.735803       -2.574126        1.288312
H          0.177527       -1.835139        0.838302
S          2.026841       -0.440157        0.282680
C         -2.994937       -0.211907        0.353744
H         -3.109171        0.536598        1.133156
H         -3.753613       -0.030717       -0.410181
H         -3.207142       -1.195541        0.780362
C          1.311846        1.864193       -0.927610
H          2.228994        1.765451       -1.510058
H          0.604859        2.471101       -1.495529
H          1.551604        2.401785       -0.008695
C         -0.643154        1.481489        1.301207
H          0.275323        1.383617        1.891401
H         -0.713468        2.541205        1.025846
H         -1.469619        1.270330        1.975818
```

3_3methyl3pentene2one_HEI_2_reopt

Datum	Value
M06-2X/def2tzvpp-IEFPCM(water) Energy	-748.063096
M06-2X/def2tzvpp-IEFPCM(water) Free Energy (Quasiharmonic)	-747.912213
Number of Imaginary Frequencies	0

Frequencies (Top 3 out of 60)

```
1.      33.6500 cm-1
2.      55.3677 cm-1
3.      83.8813 cm-1
```

M06-2X/def2tzvpp-IEFPCM(water) Molecular Geometry in Cartesian Coordinates

```
C         -1.741808        0.024117        0.117984
C         -0.557887       -0.624941       -0.158461
C          0.679406       -0.503771        0.656247
O         -2.757568        0.012873       -0.670201
H          0.476622        0.013280        1.589696
C          0.990950        2.012458       -0.543407
H          1.617310        2.718969       -1.083861
H          0.136007        1.730467       -1.155845
H          0.634580        2.477662        0.374788
S          1.984409        0.557072       -0.177710
C         -1.961648        0.830059        1.393377
H         -2.901236        0.506991        1.845636
H         -1.179134        0.761120        2.144289
H         -2.085056        1.882685        1.125424
C          1.394963       -1.811979        0.990629
H          0.726390       -2.454355        1.564807
H          1.692487       -2.350144        0.090990
H          2.292669       -1.630373        1.584357
C         -0.444402       -1.300294       -1.501473
H         -1.343797       -1.873283       -1.731549
H         -0.324071       -0.576504       -2.319631
H          0.409787       -1.976538       -1.553507
```

3_3methyl3pentene2one_HEI_3

Datum	Value
M06-2X/def2tzvpp-IEFPCM(water) Energy	-748.06253
M06-2X/def2tzvpp-IEFPCM(water) Free Energy (Quasiharmonic)	-747.910748

Datum	Value
Number of Imaginary Frequencies	0

Frequencies (Top 3 out of 60)

```
1.      56.9238 cm-1
2.      65.2228 cm-1
3.      90.0391 cm-1
```

M06-2X/def2tzvpp-IEFPCM(water) Molecular Geometry in Cartesian Coordinates

```
C          1.813051      -0.534569       0.188810
C          0.862337       0.445426       0.012412
C         -0.513470       0.190679       0.547015
O          1.602504      -1.673118       0.752550
H         -0.460061      -0.634872       1.255730
C         -3.148212      -0.748314      -0.026888
H         -3.758208      -1.342185      -0.704549
H         -2.992361      -1.314341       0.891126
H         -3.674177       0.175985       0.201553
S         -1.563950      -0.441179      -0.842551
C          3.249087      -0.333456      -0.280714
H          3.914308      -0.564407       0.553468
H          3.474290      -1.053202      -1.071599
H          3.486660       0.663288      -0.642646
C         -1.169431       1.399980       1.202122
H         -0.525863       1.775323       2.001148
H         -1.322640       2.205705       0.483181
H         -2.135275       1.148536       1.640380
C          1.057957       1.754326      -0.709894
H          1.062491       2.623213      -0.040120
H          1.982236       1.785156      -1.281631
H          0.243858       1.931166      -1.422807
```

3_3methyl3pentene2one_HEI_4

Datum	Value
M06-2X/def2tzvpp-IEFPCM(water) Energy	-748.057756
M06-2X/def2tzvpp-IEFPCM(water) Free Energy (Quasiharmonic)	-747.905898
Number of Imaginary Frequencies	0

Frequencies (Top 3 out of 60)

```
1.      53.1761 cm-1
2.      81.0240 cm-1
3.     108.0376 cm-1
```

M06-2X/def2tzvpp-IEFPCM(water) Molecular Geometry in Cartesian Coordinates

```
C          1.653974        0.079486       -0.357499
C          0.680942       -0.402228        0.488404
C         -0.606538        0.281269        0.849133
O          2.790261       -0.486444       -0.573822
H         -1.075759       -0.328801        1.624586
C         -1.938690       -1.417532       -0.926603
H         -2.596511       -1.538487       -1.784255
H         -0.931049       -1.735357       -1.191039
H         -2.308590       -2.026644       -0.102549
S         -1.916913        0.325600       -0.480031
C          1.437977        1.369515       -1.141456
H          1.812757        1.212819       -2.154246
H          0.399494        1.684264       -1.202140
H          2.027660        2.178933       -0.702331
C         -0.520456        1.701025        1.410711
H          0.144685        1.702043        2.275132
H         -0.124411        2.408079        0.684946
H         -1.501761        2.056573        1.731850
C          0.898754       -1.726070        1.178587
H          0.033098       -2.388715        1.064408
H          1.768699       -2.228905        0.759523
H          1.064434       -1.626632        2.259523
```

3_3methyl3pentene2one_HEI_5

Datum	Value
M06-2X/def2tzvpp-IEFPCM(water) Energy	-748.057952
M06-2X/def2tzvpp-IEFPCM(water) Free Energy (Quasiharmonic)	-747.905219
Number of Imaginary Frequencies	0

Frequencies (Top 3 out of 60)

```
1.      47.1253 cm-1
2.      87.5532 cm-1
3.     106.4842 cm-1
```

M06-2X/def2tzvpp-IEFPCM(water) Molecular Geometry in Cartesian Coordinates

```
C          2.031464        0.069725       -0.119648
C          0.718367        0.356285        0.180638
C         -0.364129       -0.665004        0.325571
O          2.953152        0.955040       -0.286942
H          0.013767       -1.654678        0.085411
C         -2.906051        0.615983       -0.203032
H         -3.664972        0.813244       -0.957679
H         -3.383561        0.162672        0.662896
H         -2.442409        1.556465        0.085135
S         -1.711346       -0.505919       -0.962051
C          2.537874       -1.361360       -0.274549
H          3.401794       -1.487154        0.381121
H          1.827508       -2.153216       -0.052391
H          2.897626       -1.499297       -1.297038
C         -0.995439       -0.739934        1.716031
H         -0.228969       -1.035273        2.433845
H         -1.391716        0.224623        2.032112
H         -1.807337       -1.468229        1.753549
C          0.302538        1.798026        0.303191
H         -0.314686        1.987618        1.187772
H          1.186516        2.429418        0.365056
H         -0.284971        2.135861       -0.560668
```

3_3methyl3pentene2one_HEI_6

Datum	Value
M06-2X/def2tzvpp-IEFPCM(water) Energy	-748.060302
M06-2X/def2tzvpp-IEFPCM(water) Free Energy (Quasiharmonic)	-747.907513
Number of Imaginary Frequencies	0

Frequencies (Top 3 out of 60)

```
1.      68.1181 cm-1
2.      84.1947 cm-1
3.     119.3058 cm-1
```

M06-2X/def2tzvpp-IEFPCM(water) Molecular Geometry in Cartesian Coordinates

```
C          1.805886      -0.197183      -0.023541
C          0.652561       0.547838       0.092503
C         -0.537675       0.109234       0.887138
O          2.866050       0.172426      -0.651108
H         -0.241276      -0.527351       1.719000
C         -1.842270      -0.247572      -1.618405
H         -2.424841      -0.900885      -2.264298
H         -2.345755       0.715016      -1.542672
H         -0.849032      -0.110775      -2.043082
S         -1.707267      -1.055901      -0.014915
C          1.910631      -1.579739       0.611963
H          2.693572      -1.567497       1.374437
H          0.993702      -1.960340       1.054950
H          2.230827      -2.283954      -0.158608
C         -1.354734       1.266797       1.456786
H         -0.708990       1.931911       2.034402
H         -1.813882       1.858636       0.664186
H         -2.147224       0.899097       2.107044
C          0.580742       1.887895      -0.596594
H          0.629193       2.736568       0.097163
H          1.421203       1.979900      -1.282465
H         -0.340474       2.021077      -1.171645
```

3_3methyl3pentene2one_HEI_7_reopt

Datum	Value
M06-2X/def2tzvpp-IEFPCM(water) Energy	-748.057172
M06-2X/def2tzvpp-IEFPCM(water) Free Energy (Quasiharmonic)	-747.906012
Number of Imaginary Frequencies	0

Frequencies (Top 3 out of 60)

```
1.      37.9088 cm-1
2.      59.3419 cm-1
3.      62.6720 cm-1
```

M06-2X/def2tzvpp-IEFPCM(water) Molecular Geometry in Cartesian Coordinates

```
C          -1.516735       0.373677       0.123814
C          -0.603509      -0.347660      -0.616246
C           0.754217       0.175441      -0.958792
O          -1.342021       1.545983       0.617455
H           1.078353      -0.306568      -1.886575
C           1.195173      -0.267740       1.783092
H           1.917889      -0.374316       2.589526
H           0.718585       0.709705       1.824528
H           0.426520      -1.033227       1.874209
S           2.078592      -0.447876       0.221012
C          -2.884868      -0.239370       0.439100
H          -3.600232       0.575900       0.543249
H          -3.257864      -0.934055      -0.310475
H          -2.850217      -0.767581       1.396711
C           0.919308       1.680757      -1.130381
H           0.126044       2.069336      -1.772207
H           0.845286       2.193675      -0.176860
H           1.884591       1.900131      -1.589301
C          -0.768079      -1.808606      -0.948898
H          -1.776842      -2.178857      -0.784048
H          -0.508254      -2.020838      -1.993034
H          -0.098204      -2.434151      -0.341693
```

3_3methyl3pentene2one_TS_1

Datum	Value
M06-2X/def2tzvpp-IEFPCM(water) Energy	-748.054371
M06-2X/def2tzvpp-IEFPCM(water) Free Energy (Quasiharmonic)	-747.903537
Number of Imaginary Frequencies	1

Frequencies (Top 3 out of 60)

```
1.    -206.0842 cm-1
2.      63.5762 cm-1
3.      65.7839 cm-1
```

M06-2X/def2tzvpp-IEFPCM(water) Molecular Geometry in Cartesian Coordinates

```
C          -1.624443      -0.201746      -0.476678
C          -0.638576       0.690179       0.060681
C           0.510208       0.909529      -0.681484
O          -1.501191      -0.792577      -1.565257
```

```
H           0.480910       0.550246      -1.700350
C           1.025183      -1.876369       0.842194
H           0.913507      -2.848177       0.361702
H           1.368823      -2.031090       1.864731
H           0.035402      -1.403768       0.886909
S           2.133359      -0.788419      -0.072208
C          -2.902444      -0.442464       0.313104
H          -3.400883       0.494604       0.563073
H          -3.572096      -1.060768      -0.278826
H          -2.686694      -0.953540       1.253320
C           1.403014       2.091671      -0.434720
H           2.343733       1.997351      -0.974326
H           0.902639       3.002364      -0.781122
H           1.626707       2.215314       0.623311
C          -0.747594       1.277792       1.444089
H           0.097388       0.955311       2.064300
H          -0.728355       2.370983       1.434876
H          -1.657381       0.974934       1.956668
```

3_3methyl3pentene2one_TS_2_reopt

Datum	Value
M06-2X/def2tzvpp-IEFPCM(water) Energy	-748.057292
M06-2X/def2tzvpp-IEFPCM(water) Free Energy (Quasiharmonic)	-747.906178
Number of Imaginary Frequencies	1

Frequencies (Top 3 out of 60)

```
  1.    -217.7724 cm-1
  2.      69.2213 cm-1
  3.      88.4286 cm-1
```

M06-2X/def2tzvpp-IEFPCM(water) Molecular Geometry in Cartesian Coordinates

```
C          -1.724072      -0.204748      -0.199913
C          -0.681056       0.724464       0.078062
C           0.452074       0.790267      -0.734591
O          -2.776806      -0.288251       0.467169
H           0.388198       0.309269      -1.699739
C           1.018288      -1.779872       1.019362
H           1.417384      -1.888163       2.026973
H           0.034490      -1.299881       1.095223
H           0.883325      -2.771345       0.587775
```

```
S         2.074486      -0.731809       0.004638
C        -1.554767      -1.163540      -1.371157
H        -1.607320      -0.634110      -2.323913
H        -0.588718      -1.670844      -1.331360
H        -2.354402      -1.899310      -1.337867
C         1.325627       2.016447      -0.732154
H         0.788356       2.845575      -1.203563
H         1.588143       2.320055       0.279496
H         2.245843       1.847740      -1.289410
C        -0.749638       1.547336       1.335594
H        -1.655566       1.308684       1.888584
H         0.110393       1.341819       1.983910
H        -0.746201       2.623347       1.141113
```

3_3methyl3pentene2one_TS_3_reopt

Datum	Value
M06-2X/def2tzvpp-IEFPCM(water) Energy	-748.048351
M06-2X/def2tzvpp-IEFPCM(water) Free Energy (Quasiharmonic)	-747.89856
Number of Imaginary Frequencies	1

Frequencies (Top 3 out of 60)

```
1.    -250.5005 cm-1
2.      48.9800 cm-1
3.      55.4228 cm-1
```

M06-2X/def2tzvpp-IEFPCM(water) Molecular Geometry in Cartesian Coordinates

```
C        -1.802765      -0.660850      -0.252804
C        -0.992333       0.508201      -0.109532
C         0.300306       0.472080      -0.616970
O        -1.418300      -1.707696      -0.808540
H         0.520146      -0.351122      -1.285172
C         3.186909      -0.735499      -0.266237
H         3.743147      -1.652948      -0.077324
H         2.774035      -0.799658      -1.276886
H         3.888010       0.098798      -0.243183
S         1.843881      -0.527206       0.937901
C        -3.221535      -0.641854       0.296594
H        -3.798931       0.188325      -0.111692
H        -3.710304      -1.578737       0.042441
H        -3.216081      -0.526287       1.382068
```

```
C          1.068237       1.739636      -0.862183
H          0.589540       2.294591      -1.676831
H          1.072755       2.382817       0.016129
H          2.097859       1.539261      -1.149628
C         -1.445606       1.713995       0.673715
H         -1.370015       2.635891       0.090757
H         -2.476666       1.629978       1.007654
H         -0.828461       1.861703       1.568060
```

3_3methyl3pentene2one_TS_4_reopt

Datum	Value
M06-2X/def2tzvpp-IEFPCM(water) Energy	-748.049978
M06-2X/def2tzvpp-IEFPCM(water) Free Energy (Quasiharmonic)	-747.898667
Number of Imaginary Frequencies	1

Frequencies (Top 3 out of 60)

```
1.    -223.8345 cm-1
2.      27.0791 cm-1
3.      67.7120 cm-1
```

M06-2X/def2tzvpp-IEFPCM(water) Molecular Geometry in Cartesian Coordinates

```
C          1.645050       0.069623      -0.322836
C          0.727112      -0.108408       0.749467
C         -0.422347       0.654934       0.990326
O          2.663068      -0.646177      -0.444978
H         -0.888193       0.407786       1.936895
C         -1.347346      -1.373862      -1.236431
H         -1.442418      -1.094559      -2.285228
H         -0.285319      -1.325890      -0.966212
H         -1.683725      -2.402924      -1.116143
S         -2.251259      -0.245768      -0.160985
C          1.400447       1.118040      -1.394317
H          2.099716       0.945771      -2.209344
H          0.378343       1.059961      -1.772620
H          1.554302       2.125711      -1.007535
C         -0.630391       2.114392       0.656163
H          0.143778       2.716332       1.141412
H         -0.610410       2.328641      -0.406088
H         -1.597763       2.438368       1.037596
C          0.958161      -1.329641       1.605941
```

```
H          0.194337      -1.410404       2.379799
H          0.935180      -2.251521       1.015552
H          1.933651      -1.306026       2.097626
```

3_3methyl3pentene2one_TS_5_reopt3

Datum	Value
M06-2X/def2tzvpp-IEFPCM(water) Energy	-748.057292
M06-2X/def2tzvpp-IEFPCM(water) Free Energy (Quasiharmonic)	-747.906178
Number of Imaginary Frequencies	1

Frequencies (Top 3 out of 60)

```
  1.    -217.7642 cm-1
  2.      69.1951 cm-1
  3.      88.4098 cm-1
```

M06-2X/def2tzvpp-IEFPCM(water) Molecular Geometry in Cartesian Coordinates

```
C         -1.724082      -0.204820      -0.199900
C         -0.681094       0.724425       0.078084
C          0.452022       0.790287      -0.734572
O         -2.776805      -0.288362       0.467191
H          0.388170       0.309275      -1.699714
C          1.018472      -1.779886       1.019351
H          0.883617      -2.771374       0.587768
H          1.417568      -1.888123       2.026968
H          0.034623      -1.299985       1.095184
S          2.074554      -0.731708       0.004613
C         -1.554770      -1.163587      -1.371164
H         -2.354397      -1.899366      -1.337885
H         -1.607333      -0.634135      -2.323907
H         -0.588717      -1.670882      -1.331389
C          1.325506       2.016515      -0.732156
H          0.788189       2.845600      -1.203588
H          1.587998       2.320166       0.279487
H          2.245736       1.847846      -1.289400
C         -0.749741       1.547304       1.335608
H          0.110343       1.341948       1.983896
H         -0.746494       2.623313       1.141105
H         -1.655606       1.308511       1.888640
```

3_3methyl3pentene2one_TS_6_reopt

Datum	Value
M06-2X/def2tzvpp-IEFPCM(water) Energy	-748.057292
M06-2X/def2tzvpp-IEFPCM(water) Free Energy (Quasiharmonic)	-747.906178
Number of Imaginary Frequencies	1

Frequencies (Top 3 out of 60)

```
1.    -217.7722 cm-1
2.      69.2253 cm-1
3.      88.4140 cm-1
```

M06-2X/def2tzvpp-IEFPCM(water) Molecular Geometry in Cartesian Coordinates

```
C          -1.724106       -0.204906       -0.199917
C          -0.681133        0.724391        0.078055
C           0.452003        0.790249       -0.734619
O          -2.776833       -0.288484        0.467160
H           0.388133        0.309259       -1.699770
C           1.018634       -1.779438        1.019848
H           0.882847       -2.770806        0.588272
H           1.418361       -1.888008        2.027178
H           0.035198       -1.298869        1.096360
S           2.074531       -0.731836        0.004378
C          -1.554749       -1.163674       -1.371164
H          -1.607376       -0.634234       -2.323912
H          -0.588656       -1.670889       -1.331391
H          -2.354333       -1.899501       -1.337854
C           1.325487        2.016480       -0.732116
H           0.788119        2.845668       -1.203307
H           1.588102        2.319927        0.279558
H           2.245650        1.847915       -1.289499
C          -0.749797        1.547358        1.335526
H          -0.746665        2.623355        1.140950
H          -1.655606        1.308529        1.888637
H           0.110357        1.342140        1.983766
```

3_3methyl3pentene2one_TS_7

Datum	Value
M06-2X/def2tzvpp-IEFPCM(water) Energy	-748.049803
M06-2X/def2tzvpp-IEFPCM(water) Free Energy (Quasiharmonic)	-747.898941
Number of Imaginary Frequencies	1

Frequencies (Top 3 out of 60)

```
1.    -224.7761 cm-1
2.      57.7775 cm-1
3.      72.7277 cm-1
```

M06-2X/def2tzvpp-IEFPCM(water) Molecular Geometry in Cartesian Coordinates

```
C         -1.584744        0.244807        0.339204
C         -0.649780        0.072012       -0.724507
C          0.505471        0.847419       -0.853699
O         -1.524097        1.126292        1.220327
H          0.997523        0.751481       -1.814508
C          1.316989       -1.441245        1.174113
H          1.539566       -2.491855        0.989555
H          1.477575       -1.230259        2.230812
H          0.255854       -1.273634        0.951211
S          2.296686       -0.359686        0.116092
C         -2.763235       -0.719890        0.392248
H         -3.405171       -0.596895       -0.482217
H         -2.427280       -1.758253        0.396164
H         -3.345274       -0.523919        1.289184
C          0.713847        2.194842       -0.215684
H         -0.044237        2.893032       -0.583866
H          0.618134        2.152005        0.863397
H          1.696402        2.584745       -0.477556
C         -0.786065       -1.111039       -1.654646
H         -1.809536       -1.268686       -1.997471
H         -0.165961       -0.966908       -2.540118
H         -0.456700       -2.047645       -1.186839
```

3_methyl-3-pentene-2-one_1

Datum	Value
M06-2X/def2tzvpp-IEFPCM(water) Energy	-309.853172
M06-2X/def2tzvpp-IEFPCM(water) Free Energy (Quasiharmonic)	-309.736065

Datum	Value
Number of Imaginary Frequencies	0

Frequencies (Top 3 out of 45)

```
  1.      69.5428 cm-1
  2.     104.7879 cm-1
  3.     138.1506 cm-1
```

M06-2X/def2tzvpp-IEFPCM(water) Molecular Geometry in Cartesian Coordinates

```
C          2.628339      -0.570494      -0.000002
H          2.978382       0.456989      -0.000039
H          3.040586      -1.078133      -0.874262
C          1.142087      -0.692102      -0.000001
H          3.040581      -1.078066       0.874299
C          0.237318       0.292936      -0.000011
C         -1.215551      -0.040346       0.000007
C         -1.670291      -1.480184       0.000005
O         -2.037247       0.856923       0.000026
H         -2.755890      -1.501151      -0.000019
H         -1.296468      -2.003460      -0.879627
H         -1.296509      -2.003441       0.879666
H          0.779000      -1.713457       0.000014
C          0.543945       1.761406      -0.000024
H          0.101022       2.239362      -0.874245
H          0.101171       2.239351       0.874279
H          1.611024       1.959329      -0.000112
```

3_methyl-3-pentene-2-one_2

Datum	Value
M06-2X/def2tzvpp-IEFPCM(water) Energy	-309.85038
M06-2X/def2tzvpp-IEFPCM(water) Free Energy (Quasiharmonic)	-309.732627
Number of Imaginary Frequencies	1

Frequencies (Top 3 out of 45)

```
  1.      -8.6017 cm-1
  2.      85.7224 cm-1
```

```
3.     121.8236 cm-1
```

M06-2X/def2tzvpp-IEFPCM(water) Molecular Geometry in Cartesian Coordinates

```
C          2.732992      -0.267869       0.000212
H          2.926606       0.800678      -0.000001
H          3.217942      -0.707951      -0.873567
C          1.283784      -0.613384       0.000135
H          3.217764      -0.707575       0.874280
C          0.234211       0.213403      -0.000072
C         -1.128565      -0.419639      -0.000060
C         -2.322726       0.498515       0.000241
O         -1.281246      -1.624838      -0.000250
H         -3.235006      -0.089983       0.000067
H         -2.299943       1.147303       0.876743
H         -2.299982       1.147914      -0.875804
H          1.054803      -1.674108       0.000275
C          0.314142       1.714049      -0.000283
H         -0.178775       2.135199      -0.877680
H         -0.179224       2.135481       0.876722
H          1.342754       2.061298      -0.000074
```

4_4-methyl-3-pentene-2-one_1

Datum	Value
M06-2X/def2tzvpp-IEFPCM(water) Energy	-309.85227
M06-2X/def2tzvpp-IEFPCM(water) Free Energy (Quasiharmonic)	-309.735792
Number of Imaginary Frequencies	0

Frequencies (Top 3 out of 45)

```
1.      46.8412 cm-1
2.     104.1799 cm-1
3.     130.2159 cm-1
```

M06-2X/def2tzvpp-IEFPCM(water) Molecular Geometry in Cartesian Coordinates

```
C         -2.402099      -1.005957      -0.038832
H         -2.151541      -2.053496      -0.188385
```

```
H          -3.063745       -0.677206       -0.843643
C          -1.188349       -0.129775        0.005293
H          -2.967934       -0.902120        0.889635
C           0.037636       -0.669070       -0.068675
C           1.311590        0.071773       -0.043905
C           2.547814       -0.776085        0.120423
O           1.391117        1.282162       -0.157418
H           3.434808       -0.150328        0.151582
H           2.473547       -1.368420        1.033487
H           2.618878       -1.479901       -0.710699
C          -1.468421        1.338288        0.130931
H           0.126393       -1.747547       -0.132387
H          -0.730504        1.843387        0.747282
H          -1.431241        1.806383       -0.855564
H          -2.466624        1.496914        0.536636
```

4_4-methyl-3-pentene-2-one_2

Datum	Value
M06-2X/def2tzvpp-IEFPCM(water) Energy	-309.849963
M06-2X/def2tzvpp-IEFPCM(water) Free Energy (Quasiharmonic)	-309.732399
Number of Imaginary Frequencies	0

Frequencies (Top 3 out of 45)

```
1.      40.9809 cm-1
2.     112.0935 cm-1
3.     159.2112 cm-1
```

M06-2X/def2tzvpp-IEFPCM(water) Molecular Geometry in Cartesian Coordinates

```
C          -2.458273       -0.836351        0.059437
H          -2.308520       -1.904489        0.195487
H          -3.036795       -0.669299       -0.851739
C          -1.163192       -0.082859       -0.014205
H          -3.061061       -0.451199        0.885234
C          -0.007626       -0.763178        0.011171
C           1.386858       -0.282842       -0.004378
C           1.733347        1.179014        0.107209
O           2.282129       -1.107822       -0.086578
H           2.793752        1.267476        0.326454
H           1.528635        1.679563       -0.840310
H           1.146939        1.674052        0.878466
```

```
C          -1.335302       1.407977      -0.099059
H          -0.058382      -1.845601       0.057038
H          -0.584735       1.896169      -0.710734
H          -2.316736       1.639892      -0.509837
H          -1.295005       1.845447       0.901515
```

4_4methyl3pentene2one_HEI_1_reopt

Datum	Value
M06-2X/def2tzvpp-IEFPCM(water) Energy	-748.066189
M06-2X/def2tzvpp-IEFPCM(water) Free Energy (Quasiharmonic)	-747.913331
Number of Imaginary Frequencies	0

Frequencies (Top 3 out of 60)

```
  1.      74.6308 cm-1
  2.      86.3018 cm-1
  3.     105.8432 cm-1
```

M06-2X/def2tzvpp-IEFPCM(water) Molecular Geometry in Cartesian Coordinates

```
C          -1.801872      -0.052346       0.026782
C          -0.670628      -0.611036      -0.527478
C           0.693892      -0.710207       0.069675
O          -1.931745       0.497771       1.172916
C           0.824352       2.129648      -0.036523
H           1.244607       3.000094      -0.536112
H          -0.202802       1.973712      -0.363371
H           0.841709       2.290981       1.039492
S           1.805936       0.698261      -0.510200
C          -3.072545      -0.090902      -0.819834
H          -2.936441      -0.574370      -1.786632
H          -3.856410      -0.616059      -0.269333
H          -3.427349       0.929606      -0.981909
C           0.726889      -0.734087       1.595915
H           0.099769      -1.552571       1.957954
H           1.748790      -0.895676       1.944089
H           0.334543       0.185813       2.017926
H          -0.750299      -0.986794      -1.541834
C           1.416021      -1.949732      -0.462756
H           2.454339      -1.981416      -0.125233
H           0.908194      -2.844028      -0.096771
H           1.403672      -1.971671      -1.553092
```

4_4methyl3pentene2one_HEI_2_reopt

Datum	Value
M06-2X/def2tzvpp-IEFPCM(water) Energy	-748.066213
M06-2X/def2tzvpp-IEFPCM(water) Free Energy (Quasiharmonic)	-747.913977
Number of Imaginary Frequencies	0

Frequencies (Top 3 out of 60)

```
1.      53.6281 cm-1
2.      62.3332 cm-1
3.     102.8842 cm-1
```

M06-2X/def2tzvpp-IEFPCM(water) Molecular Geometry in Cartesian Coordinates

```
C          1.849163       -0.085987       -0.056809
C          0.719226        0.091795        0.711684
C         -0.577548        0.680612        0.266486
O          2.005290        0.212333       -1.288785
C         -1.644857       -1.962213        0.176209
H         -1.988032       -2.780757       -0.452841
H         -0.647976       -2.190484        0.552387
H         -2.335225       -1.844666        1.009456
S         -1.564227       -0.482980       -0.845303
C          3.062848       -0.711281        0.625231
H          2.890891       -0.952856        1.673618
H          3.346087       -1.623085        0.094403
H          3.910742       -0.026116        0.555777
C         -1.449410        1.000564        1.479394
H         -0.937348        1.731983        2.109393
H         -2.411476        1.413888        1.174785
H         -1.627966        0.110580        2.084473
H          0.747121       -0.271599        1.732209
C         -0.427476        1.937412       -0.591095
H         -1.403015        2.304915       -0.919108
H          0.048410        2.717415        0.007507
H          0.201418        1.734383       -1.453537
```

4_4methyl3pentene2one_HEI_3

Datum	Value
M06-2X/def2tzvpp-IEFPCM(water) Energy	-748.060922
M06-2X/def2tzvpp-IEFPCM(water) Free Energy (Quasiharmonic)	-747.908774
Number of Imaginary Frequencies	0

Frequencies (Top 3 out of 60)

```
1.      32.5018 cm-1
2.      80.2347 cm-1
3.      99.4824 cm-1
```

M06-2X/def2tzvpp-IEFPCM(water) Molecular Geometry in Cartesian Coordinates

```
C         -1.903398       -0.143769       -0.264632
C         -0.709006        0.265519       -0.817005
C          0.556554        0.725077       -0.161199
O         -2.941939       -0.453511       -0.947280
C          1.565407       -1.923897       -0.445445
H          1.837840       -2.848255        0.059132
H          0.585277       -2.037626       -0.908109
H          2.310206       -1.704458       -1.208144
S          1.471813       -0.624824        0.795550
C         -2.070596       -0.261198        1.246239
H         -2.685225       -1.138232        1.451753
H         -1.129472       -0.356221        1.782136
H         -2.607942        0.609713        1.630391
C          1.515597        1.225860       -1.243741
H          1.053589        2.058930       -1.778912
H          2.455184        1.567059       -0.808979
H          1.730312        0.442534       -1.971301
H         -0.681055        0.257968       -1.903742
C          0.390311        1.841563        0.874979
H          1.355456        2.119333        1.306448
H         -0.028245        2.719439        0.379236
H         -0.278635        1.560154        1.684366
```

4_4methyl3pentene2one_HEI_4

Datum	Value
M06-2X/def2tzvpp-IEFPCM(water) Energy	-748.055956
M06-2X/def2tzvpp-IEFPCM(water) Free Energy (Quasiharmonic)	-747.903669

Datum	Value
Number of Imaginary Frequencies	0

Frequencies (Top 3 out of 60)

```
1.      46.2976 cm-1
2.      74.1079 cm-1
3.     117.9714 cm-1
```

M06-2X/def2tzvpp-IEFPCM(water) Molecular Geometry in Cartesian Coordinates

```
C         -1.972311       -0.555236       -0.144727
C         -0.618582       -0.529740       -0.374827
C          0.399616        0.550084       -0.053096
O         -2.735790       -1.545446       -0.449065
C          2.287636       -1.481173        0.604768
H          3.157727       -2.035459        0.259364
H          2.458617       -1.159585        1.629589
H          1.411940       -2.126305        0.562075
S          2.080017       -0.079373       -0.507817
C         -2.706175        0.612412        0.507416
H         -3.628878        0.783969       -0.048420
H         -2.990645        0.340851        1.527212
H         -2.148936        1.543936        0.546420
C          0.264670        1.802699       -0.932552
H         -0.705076        2.270698       -0.766634
H          1.041575        2.536985       -0.705901
H          0.331122        1.535759       -1.987757
H         -0.226170       -1.411495       -0.871112
C          0.440061        0.961290        1.423428
H         -0.505946        1.408842        1.724613
H          0.607269        0.094576        2.062051
H          1.233964        1.688745        1.603637
```

4_4methyl3pentene2one_HEI_5_reopt

Datum	Value
M06-2X/def2tzvpp-IEFPCM(water) Energy	-748.0638
M06-2X/def2tzvpp-IEFPCM(water) Free Energy (Quasiharmonic)	-747.912165
Number of Imaginary Frequencies	0

Frequencies (Top 3 out of 60)

```
1.      32.1574 cm-1
2.      51.7066 cm-1
3.     118.9265 cm-1
```

M06-2X/def2tzvpp-IEFPCM(water) Molecular Geometry in Cartesian Coordinates

```
C          2.137445       -0.080007        0.000017
C          0.874037       -0.615888        0.000003
C         -0.386387        0.222661        0.000028
O          2.443433        1.165302        0.000055
C         -3.239578       -0.009553       -0.000185
H         -4.080030       -0.700783       -0.000057
H         -3.301804        0.612689        0.889890
H         -3.301787        0.612343       -0.890502
S         -1.747234       -1.020672        0.000030
C          3.315231       -1.048186       -0.000020
H          3.011087       -2.094620       -0.000060
H          3.938248       -0.861857       -0.877841
H          3.938247       -0.861925        0.877818
C         -0.484162        1.103152        1.248782
H          0.394531        1.748183        1.274260
H         -1.376407        1.733053        1.236241
H         -0.498089        0.490928        2.150396
H          0.769984       -1.694233       -0.000045
C         -0.484191        1.103194       -1.248694
H         -1.376404        1.733139       -1.236080
H          0.394532        1.748183       -1.274198
H         -0.498201        0.491001       -2.150329
```

4_4methyl3pentene2one_HEI_6

Datum	Value
M06-2X/def2tzvpp-IEFPCM(water) Energy	-748.059573
M06-2X/def2tzvpp-IEFPCM(water) Free Energy (Quasiharmonic)	-747.906263
Number of Imaginary Frequencies	0

Frequencies (Top 3 out of 60)

```
1.      69.2260 cm-1
2.      95.5568 cm-1
3.     126.6959 cm-1
```

M06-2X/def2tzvpp-IEFPCM(water) Molecular Geometry in Cartesian Coordinates

```
C         -1.887292        0.126238       -0.289003
C         -0.652422        0.638370       -0.630927
C          0.647584        0.706106        0.095247
O         -2.903447        0.171893       -1.068992
C          0.839183       -2.135067       -0.113088
H          1.323465       -2.983363       -0.592011
H          0.753811       -2.334654        0.954258
H         -0.152678       -1.992956       -0.542710
S          1.832822       -0.674631       -0.442787
C         -2.154844       -0.522118        1.071607
H         -3.136173       -0.990447        1.028914
H         -1.419861       -1.281075        1.334372
H         -2.162999        0.217193        1.873965
C          1.407691        1.964481       -0.337413
H          0.877112        2.845418        0.028654
H          2.420737        1.977793        0.070650
H          1.468131        2.028395       -1.423933
H         -0.604533        1.037083       -1.641854
C          0.595470        0.689002        1.623119
H          1.584884        0.911583        2.024581
H         -0.102085        1.451258        1.977344
H          0.280396       -0.269343        2.027028
```

4_4methyl3pentene2one_TS_1_reopt2

Datum	Value
M06-2X/def2tzvpp-IEFPCM(water) Energy	-748.05414
M06-2X/def2tzvpp-IEFPCM(water) Free Energy (Quasiharmonic)	-747.9037
Number of Imaginary Frequencies	1

Frequencies (Top 3 out of 60)

```
1.    -192.0031 cm-1
2.      39.3684 cm-1
3.      72.4582 cm-1
```

M06-2X/def2tzvpp-IEFPCM(water) Molecular Geometry in Cartesian Coordinates

```
C         -1.808205      -0.088340       0.093059
C         -0.694988      -0.555196      -0.666864
C          0.516191      -1.024574      -0.176483
O         -1.874694      -0.029864       1.332286
C          0.705338       2.264416      -0.204278
H          1.077688       2.923439      -0.988181
H         -0.196578       1.765234      -0.582085
H          0.416981       2.874825       0.651570
S          1.912246       1.000192       0.241844
C         -3.018922       0.393853      -0.691075
H         -2.895173       0.307146      -1.768141
H         -3.892396      -0.182245      -0.382928
H         -3.212204       1.436808      -0.435561
C          0.652818      -1.594864       1.211754
H          0.212813      -2.599254       1.210865
H          1.703502      -1.690513       1.482055
H          0.132843      -0.998273       1.950476
H         -0.777047      -0.439051      -1.741147
C          1.448089      -1.666373      -1.174634
H          2.473909      -1.674219      -0.810903
H          1.136119      -2.705392      -1.326972
H          1.419238      -1.156195      -2.135720
```

4_4methyl3pentene2one_TS_2_reopt

Datum	Value
M06-2X/def2tzvpp-IEFPCM(water) Energy	-748.05414
M06-2X/def2tzvpp-IEFPCM(water) Free Energy (Quasiharmonic)	-747.903702
Number of Imaginary Frequencies	1

Frequencies (Top 3 out of 60)

```
1.    -191.9785 cm-1
2.      39.1700 cm-1
3.      72.4445 cm-1
```

M06-2X/def2tzvpp-IEFPCM(water) Molecular Geometry in Cartesian Coordinates

```
C         -1.808180      -0.088344      -0.093213
C         -0.695041      -0.555108       0.666889
C          0.516166      -1.024580       0.176691
O         -1.874536      -0.030009      -1.332454
C          0.705296       2.264479       0.203968
H          0.417356       2.874988      -0.651950
H         -0.196835       1.765351       0.581339
H          1.077376       2.923403       0.988083
S          1.912260       1.000174      -0.241803
C         -3.018984       0.393921       0.690743
H         -2.895294       0.307452       1.767834
H         -3.212347       1.436802       0.434991
H         -3.892389      -0.182325       0.382670
C          1.448015      -1.666189       1.175010
H          1.136025      -2.705172       1.327551
H          2.473849      -1.674125       0.811320
H          1.419136      -1.155810       2.135987
H         -0.777173      -0.438781       1.741138
C          0.652891      -1.595103      -1.211441
H          1.703589      -1.690692      -1.481704
H          0.212996      -2.599542      -1.210371
H          0.132859      -0.998707      -1.950281
```

4_4methyl3pentene2one_TS_3_reopt

Datum	Value
M06-2X/def2tzvpp-IEFPCM(water) Energy	-748.051624
M06-2X/def2tzvpp-IEFPCM(water) Free Energy (Quasiharmonic)	-747.900102
Number of Imaginary Frequencies	1

Frequencies (Top 3 out of 60)

```
1.    -219.6187 cm-1
2.      56.8082 cm-1
3.      67.6907 cm-1
```

M06-2X/def2tzvpp-IEFPCM(water) Molecular Geometry in Cartesian Coordinates

```
C          1.875580      -0.102654      -0.174920
C          0.694841      -0.685784      -0.716015
C         -0.514292      -1.003815      -0.093586
O          2.917053       0.018707      -0.848685
```

```
C          -0.650821       2.195497       -0.528460
H          -0.271771       2.925685        0.186561
H           0.204093       1.633887       -0.926147
H          -1.119721       2.728344       -1.354859
S          -1.779083       1.022391        0.243690
C           1.885401       0.456747        1.235250
H           2.681547       1.195191        1.304352
H           0.927401       0.913391        1.486749
H           2.088151      -0.333257        1.960231
C          -1.505595      -1.733463       -0.975643
H          -1.204240      -2.783335       -1.054063
H          -2.508654      -1.701767       -0.554484
H          -1.532562      -1.310311       -1.978167
H           0.737942      -0.821335       -1.792766
C          -0.609647      -1.475157        1.340224
H          -1.651112      -1.497414        1.659820
H          -0.221089      -2.498241        1.389432
H          -0.053878      -0.866987        2.042684
```

4_4methyl3pentene2one_TS_4_reopt

Datum	Value
M06-2X/def2tzvpp-IEFPCM(water) Energy	-748.051086
M06-2X/def2tzvpp-IEFPCM(water) Free Energy (Quasiharmonic)	-747.899231
Number of Imaginary Frequencies	1

Frequencies (Top 3 out of 60)

```
1.    -230.0830 cm-1
2.      39.1090 cm-1
3.      81.8258 cm-1
```

M06-2X/def2tzvpp-IEFPCM(water) Molecular Geometry in Cartesian Coordinates

```
C           1.917928      -0.016967       -0.293467
C           0.697585      -0.625775       -0.704419
C          -0.450235      -0.970064        0.010262
O           2.831555       0.206031       -1.113236
C          -0.816229       2.165985       -0.045336
H          -1.112052       3.034148       -0.633078
H          -0.666584       2.485168        0.986991
H           0.140002       1.803412       -0.438791
S          -2.030484       0.840260       -0.181777
```

```
C          2.184143       0.368472        1.152179
H          3.180761       0.798970        1.211373
H          1.458335       1.100481        1.506110
H          2.128070      -0.496051        1.812284
C         -1.349744      -1.986686       -0.655850
H         -0.949797      -2.988124       -0.464481
H         -2.361620      -1.946967       -0.254272
H         -1.389341      -1.836104       -1.732980
H          0.661795      -0.807567       -1.774741
C         -0.480152      -1.079198        1.518447
H          0.201435      -1.874296        1.838304
H         -0.191419      -0.160937        2.018790
H         -1.484060      -1.339142        1.847912
```

4_4methyl3pentene2one_TS_5_reopt

Datum	Value
M06-2X/def2tzvpp-IEFPCM(water) Energy	-748.050048
M06-2X/def2tzvpp-IEFPCM(water) Free Energy (Quasiharmonic)	-747.900209
Number of Imaginary Frequencies	1

Frequencies (Top 3 out of 60)

```
1.    -238.0725 cm-1
2.      63.3513 cm-1
3.      68.2148 cm-1
```

M06-2X/def2tzvpp-IEFPCM(water) Molecular Geometry in Cartesian Coordinates

```
C         -2.124701      -0.257949        0.090796
C         -1.120324       0.581851       -0.470165
C          0.124709       0.875378        0.073333
O         -2.040992      -0.886821        1.159565
C          3.172506      -0.743060        0.217032
H          3.982025      -1.316005       -0.236211
H          3.464472       0.309600        0.198872
H          3.086192      -1.048431        1.260319
S          1.611449      -0.993401       -0.680958
C         -3.413861      -0.405278       -0.702176
H         -3.447484       0.225202       -1.587655
H         -3.524318      -1.448151       -1.004218
H         -4.257794      -0.164302       -0.055071
C          0.909470       1.980254       -0.584529
```

```
H          0.563657       2.937495      -0.178761
H          1.974042       1.896306      -0.373716
H          0.758316       1.992717      -1.662063
H         -1.324668       0.970704      -1.460523
C          0.420962       0.719820       1.539864
H          1.491020       0.802566       1.725645
H         -0.075235       1.539742       2.072730
H          0.041955      -0.214556       1.934537
```

4_4methyl3pentene2one_TS_6

Datum	Value
M06-2X/def2tzvpp-IEFPCM(water) Energy	-748.051086
M06-2X/def2tzvpp-IEFPCM(water) Free Energy (Quasiharmonic)	-747.89923
Number of Imaginary Frequencies	1

Frequencies (Top 3 out of 60)

```
1.    -230.1214 cm-1
2.      39.1601 cm-1
3.      81.7792 cm-1
```

M06-2X/def2tzvpp-IEFPCM(water) Molecular Geometry in Cartesian Coordinates

```
C         -1.917914       0.016957      -0.293519
C         -0.697561       0.625770      -0.704398
C          0.450239       0.970023       0.010323
O         -2.831458      -0.206094      -1.113370
C          0.816283      -2.165912      -0.045123
H          1.112074      -3.034232      -0.632648
H          0.666741      -2.484795       0.987312
H         -0.140008      -1.803494      -0.438577
S          2.030521      -0.840196      -0.181999
C         -2.184323      -0.368369       1.152123
H         -3.181038      -0.798656       1.211227
H         -1.458701      -1.100522       1.506127
H         -2.128118       0.496147       1.812227
C          1.349688       1.986798      -0.655633
H          0.949663       2.988181      -0.464139
H          2.361562       1.947097      -0.254049
H          1.389306       1.836366      -1.732783
H         -0.661745       0.807622      -1.774709
C          0.480172       1.078890       1.518518
```

```
H          1.484157        1.338437        1.848050
H         -0.201161        1.874162        1.838489
H          0.191095        0.160637        2.018680
```

4-hexene-3-one_1

Datum	Value
M06-2X/def2tzvpp-IEFPCM(water) Energy	-309.849904
M06-2X/def2tzvpp-IEFPCM(water) Free Energy (Quasiharmonic)	-309.732746
Number of Imaginary Frequencies	0

Frequencies (Top 3 out of 45)

```
1.      44.1323 cm-1
2.      96.9275 cm-1
3.     146.8401 cm-1
```

M06-2X/def2tzvpp-IEFPCM(water) Molecular Geometry in Cartesian Coordinates

```
C          3.043151       -0.116681        0.000001
C          1.685162       -0.791352       -0.000001
C          0.522605        0.170935        0.000000
C         -0.825990       -0.450014        0.000001
C         -1.935729        0.283922       -0.000001
C         -3.322371       -0.257597        0.000000
O          0.684996        1.374738        0.000000
H          3.839136       -0.859204        0.000000
H          3.161851        0.514548       -0.879496
H          3.161850        0.514546        0.879498
H          1.560052       -1.445398        0.868430
H          1.560053       -1.445396       -0.868434
H         -0.873631       -1.533387        0.000002
H         -1.824251        1.364411       -0.000002
H         -3.867913        0.101191       -0.874806
H         -3.330175       -1.345693        0.000001
H         -3.867913        0.101193        0.874806
```

4-hexene-3-one_2

Datum	Value
M06-2X/def2tzvpp-IEFPCM(water) Energy	-309.85036
M06-2X/def2tzvpp-IEFPCM(water) Free Energy (Quasiharmonic)	-309.73259
Number of Imaginary Frequencies	0

Frequencies (Top 3 out of 45)

```
1.      76.2755 cm-1
2.     112.6474 cm-1
3.     165.6365 cm-1
```

M06-2X/def2tzvpp-IEFPCM(water) Molecular Geometry in Cartesian Coordinates

```
C         -2.750722       -0.747446        0.000001
C         -1.234314       -0.820958       -0.000001
C         -0.574456        0.540844       -0.000000
C          0.901016        0.630537        0.000000
C          1.723149       -0.417074       -0.000000
C          3.210170       -0.324930        0.000000
O         -1.236558        1.561569       -0.000000
H         -3.177887       -1.748740        0.000002
H         -3.114124       -0.218098        0.879388
H         -3.114126       -0.218098       -0.879386
H         -0.869182       -1.370457       -0.871019
H         -0.869179       -1.370460        0.871014
H          1.293322        1.642137        0.000001
H          1.306810       -1.419428       -0.000001
H          3.619991       -0.832313       -0.875176
H          3.547794        0.709382        0.000001
H          3.619990       -0.832313        0.875177
```

4-hexene-3-one_3

Datum	Value
M06-2X/def2tzvpp-IEFPCM(water) Energy	-309.848044
M06-2X/def2tzvpp-IEFPCM(water) Free Energy (Quasiharmonic)	-309.730454
Number of Imaginary Frequencies	0

Frequencies (Top 3 out of 45)

```
1.      59.1809 cm-1
2.      85.4025 cm-1
3.     165.1824 cm-1
```

M06-2X/def2tzvpp-IEFPCM(water) Molecular Geometry in Cartesian Coordinates

```
C          2.328968       -1.110880       -0.485527
C          1.908155       -0.072420        0.559502
C          0.611765        0.583056        0.154236
C         -0.607850       -0.256251        0.264541
C         -1.802319        0.190800       -0.115894
C         -3.073381       -0.578728       -0.031288
O          0.580240        1.723658       -0.265340
H          3.262478       -1.586050       -0.190190
H          1.574262       -1.889214       -0.596529
H          2.477073       -0.639285       -1.456661
H          2.667615        0.700269        0.661450
H          1.770743       -0.561200        1.526196
H         -0.492372       -1.253302        0.674630
H         -1.856888        1.197578       -0.519953
H         -3.523058       -0.674282       -1.021387
H         -2.916856       -1.572239        0.383911
H         -3.796941       -0.045006        0.587840
```

4-hexene-3-one_4

Datum	Value
M06-2X/def2tzvpp-IEFPCM(water) Energy	-309.848683
M06-2X/def2tzvpp-IEFPCM(water) Free Energy (Quasiharmonic)	-309.73063
Number of Imaginary Frequencies	0

Frequencies (Top 3 out of 45)

```
1.      69.8634 cm-1
2.     107.8091 cm-1
3.     164.5924 cm-1
```

M06-2X/def2tzvpp-IEFPCM(water) Molecular Geometry in Cartesian Coordinates

```
C          1.701100      -1.510056      -0.487142
C          1.453326      -0.503655       0.641575
C          0.763211       0.730416       0.108645
C         -0.696005       0.724242      -0.118304
C         -1.507198      -0.292128       0.171769
C         -2.978133      -0.292461      -0.062982
O          1.409714       1.722679      -0.176794
H          2.239540      -2.376576      -0.107787
H          0.764197      -1.854146      -0.923480
H          2.298394      -1.056714      -1.277893
H          2.401288      -0.180854       1.068150
H          0.863199      -0.963631       1.433293
H         -1.089175       1.635262      -0.557138
H         -1.094746      -1.195639       0.609401
H         -3.509835      -0.456492       0.876004
H         -3.314602       0.644281      -0.502232
H         -3.253773      -1.115075      -0.725334
```

4-hexene-3-one_5

Datum	Value
M06-2X/def2tzvpp-IEFPCM(water) Energy	-309.849904
M06-2X/def2tzvpp-IEFPCM(water) Free Energy (Quasiharmonic)	-309.732747
Number of Imaginary Frequencies	0

Frequencies (Top 3 out of 45)

```
  1.      44.0452 cm-1
  2.      96.9466 cm-1
  3.     146.8115 cm-1
```

M06-2X/def2tzvpp-IEFPCM(water) Molecular Geometry in Cartesian Coordinates

```
C          3.043119      -0.116667       0.000365
C          1.685184      -0.791358      -0.000285
C          0.522654       0.170924      -0.000148
C         -0.826036      -0.450051      -0.000039
C         -1.935736       0.283927       0.000061
C         -3.322393      -0.257562       0.000141
O          0.685029       1.374711      -0.000161
H          3.839145      -0.859150       0.000079
H          3.162008       0.515092      -0.878730
```

```
H          3.161666       0.514044       0.880262
H          1.559904      -1.445817       0.867803
H          1.560315      -1.444933      -0.869104
H         -0.873682      -1.533419      -0.000042
H         -1.824254       1.364414       0.000016
H         -3.867899       0.101219      -0.874689
H         -3.330307      -1.345664       0.000161
H         -3.867892       0.101245       0.874962
```

4-hexene-3-one_HEI_10

Datum	Value
M06-2X/def2tzvpp-IEFPCM(water) Energy	-748.06377
M06-2X/def2tzvpp-IEFPCM(water) Free Energy (Quasiharmonic)	-747.910594
Number of Imaginary Frequencies	0

Frequencies (Top 3 out of 60)

```
1.      58.5033 cm-1
2.      76.7668 cm-1
3.      92.9933 cm-1
```

M06-2X/def2tzvpp-IEFPCM(water) Molecular Geometry in Cartesian Coordinates

```
C          2.481505       1.745818       0.060023
C          1.905487       0.573172       0.851079
C          1.725118      -0.657355      -0.035869
C          0.468909      -1.184746      -0.236611
C         -0.818486      -0.704718       0.343418
C         -1.859518      -1.814381       0.412313
O          2.800498      -1.120526      -0.553062
H          2.659960       2.611302       0.699337
H          3.424847       1.461909      -0.404514
H          1.791522       2.047352      -0.730653
H          0.971191       0.878693       1.320803
H          2.603566       0.303724       1.649188
H         -0.689152      -0.283809       1.342069
H         -1.498914      -2.612748       1.063752
H         -2.813594      -1.462481       0.804073
H         -2.028601      -2.234450      -0.580513
C         -2.941460       1.204836       0.228272
H         -3.243147       2.183143      -0.140165
H         -3.760900       0.504981       0.080568
```

```
H          -2.719357        1.285620        1.292224
S          -1.454067        0.708834       -0.674907
H           0.394340       -2.036125       -0.908900
```

4-hexene-3-one_HEI_11_reopt

Datum	Value
M06-2X/def2tzvpp-IEFPCM(water) Energy	-748.062182
M06-2X/def2tzvpp-IEFPCM(water) Free Energy (Quasiharmonic)	-747.909141
Number of Imaginary Frequencies	0

Frequencies (Top 3 out of 60)

```
1.      34.1109 cm-1
2.      67.8926 cm-1
3.      95.9302 cm-1
```

M06-2X/def2tzvpp-IEFPCM(water) Molecular Geometry in Cartesian Coordinates

```
C           2.476783       -1.485814       -0.290250
C           1.989196       -0.159111       -0.870207
C           1.497935        0.765684        0.240112
C           0.163079        1.094394        0.318698
C          -0.925720        0.686874       -0.609049
C          -2.116573        1.637370       -0.531236
O           2.399184        1.161743        1.058868
H           2.900199       -2.131818       -1.060382
H           3.239555       -1.306518        0.466625
H           1.648614       -2.018849        0.180847
H           1.218267       -0.351216       -1.615750
H           2.821125        0.341165       -1.373787
H          -0.586532        0.639122       -1.644691
H          -2.533873        1.645072        0.477489
H          -1.794020        2.654495       -0.761405
H          -2.905364        1.352701       -1.226159
C          -1.853323       -1.090083        1.386417
H          -0.958994       -0.753908        1.909602
H          -2.695655       -0.454958        1.654864
H          -2.078293       -2.114635        1.673906
S          -1.544703       -1.074524       -0.386664
H          -0.141516        1.701911        1.167607
```

4-hexene-3-one_HEI_12_reopt

Datum	Value
M06-2X/def2tzvpp-IEFPCM(water) Energy	-748.062182
M06-2X/def2tzvpp-IEFPCM(water) Free Energy (Quasiharmonic)	-747.909142
Number of Imaginary Frequencies	0

Frequencies (Top 3 out of 60)

```
1.      34.0570 cm-1
2.      67.8849 cm-1
3.      95.9239 cm-1
```

M06-2X/def2tzvpp-IEFPCM(water) Molecular Geometry in Cartesian Coordinates

```
C        -2.476803      -1.485836      -0.290213
C        -1.989217      -0.159140      -0.870189
C        -1.497954       0.765671       0.240116
C        -0.163097       1.094379       0.318698
C         0.925697       0.686860      -0.609057
C         2.116534       1.637378      -0.531274
O        -2.399204       1.161747       1.058863
H        -2.900228      -2.131846      -1.060335
H        -1.648631      -2.018868       0.180881
H        -3.239567      -1.306532       0.466668
H        -2.821147       0.341129      -1.373775
H        -1.218290      -0.351256      -1.615731
H         0.586495       0.639089      -1.644693
H         2.905326       1.352706      -1.226195
H         1.793963       2.654492      -0.761466
H         2.533840       1.645113       0.477448
C         1.853450      -1.090021       1.386402
H         0.959171      -0.753778       1.909630
H         2.078386      -2.114572       1.673923
H         2.695832      -0.454927       1.654766
S         1.544708      -1.074524      -0.386658
H         0.141498       1.701914       1.167594
```

4-hexene-3-one_HEI_13

Datum	Value
M06-2X/def2tzvpp-IEFPCM(water) Energy	-748.068222
M06-2X/def2tzvpp-IEFPCM(water) Free Energy (Quasiharmonic)	-747.914827
Number of Imaginary Frequencies	0

Frequencies (Top 3 out of 60)

```
1.      58.8206 cm-1
2.      80.1931 cm-1
3.      92.2385 cm-1
```

M06-2X/def2tzvpp-IEFPCM(water) Molecular Geometry in Cartesian Coordinates

```
C         -3.310876        0.670196       -0.560158
C         -2.598703       -0.672160       -0.410955
C         -1.270187       -0.520108        0.325472
C         -0.120789       -0.848323       -0.357231
C          1.228524       -0.725919        0.237867
C          2.158821       -1.884405       -0.104600
O         -1.337333       -0.086369        1.527373
H         -4.281594        0.561617       -1.045487
H         -3.465453        1.124849        0.417665
H         -2.709525        1.357532       -1.159270
H         -2.442748       -1.124048       -1.392200
H         -3.233405       -1.350685        0.167204
H          1.132952       -0.626999        1.319168
H          2.251126       -1.988968       -1.187552
H          3.158091       -1.744239        0.311236
H          1.747172       -2.814445        0.288319
C          0.952907        2.075316        0.147377
H          1.257312        3.007883       -0.322858
H          0.913258        2.210424        1.226179
H         -0.029871        1.778741       -0.217266
S          2.146864        0.807479       -0.306584
H         -0.186658       -1.187941       -1.385399
```

4-hexene-3-one_HEI_14

Datum	Value
M06-2X/def2tzvpp-IEFPCM(water) Energy	-748.068222
M06-2X/def2tzvpp-IEFPCM(water) Free Energy (Quasiharmonic)	-747.914829

Datum	Value
Number of Imaginary Frequencies	0

Frequencies (Top 3 out of 60)

```
1.      58.8685 cm-1
2.      80.1387 cm-1
3.      92.2795 cm-1
```

M06-2X/def2tzvpp-IEFPCM(water) Molecular Geometry in Cartesian Coordinates

```
C          3.310837        0.670106       -0.559943
C          2.598580       -0.672258       -0.411222
C          1.270119       -0.520287        0.325405
C          0.120668       -0.848596       -0.357176
C         -1.228585       -0.725896        0.237984
C         -2.159205       -1.884167       -0.104404
O          1.337420       -0.086429        1.527212
H          4.281728        0.561629       -1.044945
H          2.709705        1.357574       -1.159131
H          3.465014        1.124500        0.418069
H          3.233276       -1.351043        0.166649
H          2.442504       -1.123772       -1.392619
H         -1.133059       -0.626885        1.319282
H         -2.251799       -1.988600       -1.187338
H         -1.747803       -2.814389        0.288356
H         -3.158342       -1.743743        0.311669
C         -0.952612        2.075286        0.147543
H         -1.256784        3.007981       -0.322601
H         -0.913305        2.210198        1.226381
H          0.030238        1.778625       -0.216823
S         -2.146624        0.807656       -0.306777
H          0.186421       -1.188261       -1.385330
```

4-hexene-3-one_HEI_15_reopt

Datum	Value
M06-2X/def2tzvpp-IEFPCM(water) Energy	-748.06646
M06-2X/def2tzvpp-IEFPCM(water) Free Energy (Quasiharmonic)	-747.913786
Number of Imaginary Frequencies	0

Frequencies (Top 3 out of 60)

```
1.      44.1539 cm-1
2.      68.8193 cm-1
3.      74.1413 cm-1
```

M06-2X/def2tzvpp-IEFPCM(water) Molecular Geometry in Cartesian Coordinates

```
C          -3.371784        1.040947       -0.261113
C          -2.697094       -0.279341       -0.624518
C          -1.419544       -0.502700        0.179470
C          -0.234568       -0.612669       -0.511308
C           1.061661       -0.850407        0.168111
C           2.040602       -1.655876       -0.678178
O          -1.550698       -0.567783        1.449896
H          -4.306296        1.179634       -0.806116
H          -3.590192        1.072490        0.805698
H          -2.716888        1.882934       -0.495465
H          -2.482979       -0.308448       -1.694480
H          -3.383310       -1.103144       -0.404652
H           0.882806       -1.358608        1.117356
H           2.247411       -1.142508       -1.619272
H           2.986680       -1.816350       -0.161647
H           1.605453       -2.626235       -0.924015
C           2.064115        1.617087       -0.770972
H           2.801925        1.166194       -1.431833
H           2.382126        2.628141       -0.526704
H           1.097147        1.657455       -1.271255
S           1.903447        0.705991        0.774743
H          -0.233771       -0.527408       -1.591621
```

4-hexene-3-one_HEI_16

Datum	Value
M06-2X/def2tzvpp-IEFPCM(water) Energy	-748.066459
M06-2X/def2tzvpp-IEFPCM(water) Free Energy (Quasiharmonic)	-747.913786
Number of Imaginary Frequencies	0

Frequencies (Top 3 out of 60)

```
1.      44.0342 cm-1
2.      68.8199 cm-1
3.      74.1534 cm-1
```

M06-2X/def2tzvpp-IEFPCM(water) Molecular Geometry in Cartesian Coordinates

```
C          -3.371697        1.040895        0.261186
C          -2.697061       -0.279471        0.624428
C          -1.419479       -0.502769       -0.179531
C          -0.234540       -0.612825        0.511279
C           1.061723       -0.850459       -0.168069
C           2.040773       -1.655728        0.678297
O          -1.550590       -0.567724       -1.449964
H          -3.590073        1.072639       -0.805623
H          -4.306216        1.179530        0.806191
H          -2.716776        1.882825        0.495675
H          -2.482996       -0.308718        1.694395
H          -3.383284       -1.103229        0.404425
H           0.883022       -1.358750       -1.117305
H           2.247525       -1.142247        1.619339
H           1.605783       -2.626139        0.924215
H           2.986868       -1.816094        0.161755
C           2.063682        1.617285        0.770911
H           2.381819        2.628287        0.526584
H           2.801311        1.166430        1.432003
H           1.096613        1.657753        1.270978
S           1.903407        0.705974       -0.774721
H          -0.233790       -0.527650        1.591598
```

4-hexene-3-one_HEI_17_reopt

Datum	Value
M06-2X/def2tzvpp-IEFPCM(water) Energy	-748.06646
M06-2X/def2tzvpp-IEFPCM(water) Free Energy (Quasiharmonic)	-747.913787
Number of Imaginary Frequencies	0

Frequencies (Top 3 out of 60)

```
1.      44.0971 cm-1
2.      68.7955 cm-1
3.      74.1370 cm-1
```

M06-2X/def2tzvpp-IEFPCM(water) Molecular Geometry in Cartesian Coordinates

```
C          3.371792       1.040931       -0.261000
C          2.697066      -0.279299       -0.624551
C          1.419525      -0.502724        0.179435
C          0.234541      -0.612635       -0.511338
C         -1.061682      -0.850412        0.168074
C         -2.040620      -1.655864       -0.678237
O          1.550695      -0.567914        1.449853
H          4.306280       1.179675       -0.806030
H          2.716897       1.882958       -0.495208
H          3.590254       1.072330        0.805805
H          3.383269      -1.103143       -0.404797
H          2.482930      -0.308273       -1.694511
H         -0.882818      -1.358639        1.117303
H         -2.986686      -1.816379       -0.161697
H         -2.247456      -1.142458       -1.619304
H         -1.605456      -2.626204       -0.924124
C         -2.063964       1.617198       -0.770899
H         -1.096925       1.657659       -1.271038
H         -2.382061       2.628212       -0.526578
H         -2.801656       1.166334       -1.431911
S         -1.903489       0.705959        0.774750
H          0.233732      -0.527278       -1.591645
```

4-hexene-3-one_HEI_18

Datum	Value
M06-2X/def2tzvpp-IEFPCM(water) Energy	-748.067611
M06-2X/def2tzvpp-IEFPCM(water) Free Energy (Quasiharmonic)	-747.914992
Number of Imaginary Frequencies	0

Frequencies (Top 3 out of 60)

```
1.      37.1767 cm-1
2.      59.0313 cm-1
3.      73.9908 cm-1
```

M06-2X/def2tzvpp-IEFPCM(water) Molecular Geometry in Cartesian Coordinates

```
C         3.583841      -0.857037      -0.557744
C         2.916632       0.467799      -0.197542
C         1.525222       0.255361       0.391628
C         0.454477       0.803361      -0.276808
C        -0.945100       0.663462       0.206066
C        -1.812001       1.873859      -0.110333
O         1.464788      -0.423803       1.473087
H         3.636139      -1.502433       0.318230
H         3.009110      -1.378498      -1.326193
H         4.596066      -0.709428      -0.935994
H         2.864745       1.112983      -1.076354
H         3.525140       0.981891       0.552888
H        -0.939174       0.471479       1.280826
H        -1.813362       2.065381      -1.184966
H        -2.844207       1.742260       0.215039
H        -1.408647       2.755321       0.391880
C        -3.280443      -0.974673       0.285536
H        -3.685053      -1.966295       0.093186
H        -3.154663      -0.850594       1.360777
H        -3.981718      -0.230992      -0.086400
S        -1.674699      -0.865581      -0.539834
H         0.616727       1.355856      -1.195097
```

4-hexene-3-one_HEI_19

Datum	Value
M06-2X/def2tzvpp-IEFPCM(water) Energy	-748.067612
M06-2X/def2tzvpp-IEFPCM(water) Free Energy (Quasiharmonic)	-747.914992
Number of Imaginary Frequencies	0

Frequencies (Top 3 out of 60)

```
1.     37.2731 cm-1
2.     59.0405 cm-1
3.     74.0082 cm-1
```

M06-2X/def2tzvpp-IEFPCM(water) Molecular Geometry in Cartesian Coordinates

```
C        -3.583711      -0.857010      -0.558153
C        -2.916733       0.467799      -0.197307
C        -1.525338       0.255274       0.391817
C        -0.454563       0.803278      -0.276638
```

```
C          0.944999       0.663425       0.206044
C          1.811832       1.873878      -0.110499
O         -1.464865      -0.423984       1.473208
H         -3.635657      -1.502954       0.317452
H         -4.596082      -0.709393      -0.936010
H         -3.009117      -1.377864      -1.327099
H         -2.864994       1.113408      -1.075815
H         -3.525393       0.981354       0.553358
H          0.939237       0.471349       1.280809
H          1.813062       2.065362      -1.185133
H          1.408544       2.755357       0.391743
H          2.844073       1.742303       0.214760
C          3.280563      -0.974478       0.285628
H          3.154785      -0.850047       1.360827
H          3.981945      -0.231005      -0.086513
H          3.685066      -1.966207       0.093609
S          1.674884      -0.865549      -0.539890
H         -0.616979       1.355992      -1.194770
```

4-hexene-3-one_HEI_1

Datum	Value
M06-2X/def2tzvpp-IEFPCM(water) Energy	-748.064145
M06-2X/def2tzvpp-IEFPCM(water) Free Energy (Quasiharmonic)	-747.910893
Number of Imaginary Frequencies	0

Frequencies (Top 3 out of 60)

```
1.      70.8408 cm-1
2.      75.8046 cm-1
3.      97.6981 cm-1
```

M06-2X/def2tzvpp-IEFPCM(water) Molecular Geometry in Cartesian Coordinates

```
C          2.830026       1.086876      -0.475480
C          1.860308      -0.010936      -0.902787
C          1.344622      -0.821546       0.288936
C         -0.002958      -1.067472       0.443426
C         -1.126612      -0.627656      -0.414173
C         -2.200724      -1.699854      -0.575852
O          2.251530      -1.260303       1.079311
H          3.243564       1.608894      -1.339152
H          2.330411       1.825643       0.154349
```

```
H          3.650777        0.659456        0.097300
H          2.387154       -0.706535       -1.564197
H          1.048536        0.424921       -1.482936
H         -0.789434       -0.316897       -1.401370
H         -2.568935       -2.015487        0.402046
H         -3.048402       -1.342870       -1.162785
H         -1.775369       -2.572349       -1.072296
C         -0.705597        2.048079        0.352949
H         -0.371639        2.358810       -0.636388
H          0.125298        1.589362        0.888285
H         -1.057459        2.918648        0.901980
S         -2.055769        0.865882        0.247473
H         -0.288826       -1.648223        1.319004
```

4-hexene-3-one_HEI_2

Datum	Value
M06-2X/def2tzvpp-IEFPCM(water) Energy	-748.064145
M06-2X/def2tzvpp-IEFPCM(water) Free Energy (Quasiharmonic)	-747.910889
Number of Imaginary Frequencies	0

Frequencies (Top 3 out of 60)

```
1.      70.8500 cm-1
2.      75.9632 cm-1
3.      98.1002 cm-1
```

M06-2X/def2tzvpp-IEFPCM(water) Molecular Geometry in Cartesian Coordinates

```
C         -2.830361        1.086484       -0.475485
C         -1.860159       -0.010914       -0.902741
C         -1.344450       -0.821563        0.288954
C          0.003124       -1.067503        0.443387
C          1.126773       -0.627517       -0.414206
C          2.200943       -1.699636       -0.575950
O         -2.251351       -1.260349        1.079330
H         -3.244013        1.608386       -1.339173
H         -3.651007        0.658693        0.097176
H         -2.331136        1.825420        0.154454
H         -1.048382        0.425310       -1.482613
H         -2.386602       -0.706579       -1.564400
H          0.789528       -0.316783       -1.401393
H          2.569097       -2.015374        0.401938
```

```
H          1.775654       -2.572088       -1.072528
H          3.048651       -1.342554       -1.162779
C          0.705267        2.047994        0.352780
H          0.371616        2.358951       -0.636588
H          1.056785        2.918456        0.902198
H         -0.125725        1.588970        0.887709
S          2.055654        0.866012        0.247625
H          0.289059       -1.648276        1.318924
```

4-hexene-3-one_HEI_3

Datum	Value
M06-2X/def2tzvpp-IEFPCM(water) Energy	-748.06377
M06-2X/def2tzvpp-IEFPCM(water) Free Energy (Quasiharmonic)	-747.910589
Number of Imaginary Frequencies	0

Frequencies (Top 3 out of 60)

```
1.      58.5561 cm-1
2.      76.7332 cm-1
3.      93.0653 cm-1
```

M06-2X/def2tzvpp-IEFPCM(water) Molecular Geometry in Cartesian Coordinates

```
C          2.481081        1.746048        0.060115
C          1.905989        0.573006        0.851282
C          1.725300       -0.657248       -0.035956
C          0.469029       -1.184480       -0.236758
C         -0.818379       -0.704642        0.343504
C         -1.859227       -1.814484        0.412517
O          2.800552       -1.120391       -0.553454
H          2.659487        2.611497        0.699492
H          3.424322        1.462666       -0.404969
H          1.790533        2.047380       -0.730147
H          0.972016        0.878110        1.321922
H          2.604818        0.303492        1.648710
H         -0.688890       -0.283773        1.342142
H         -1.498411       -2.612827        1.063866
H         -2.813307       -1.462737        0.804401
H         -2.028374       -2.234536       -0.580308
C         -2.941896        1.204522        0.228085
H         -2.720036        1.285445        1.292076
H         -3.761145        0.504468        0.080269
```

```
H          -3.243722        2.182727       -0.140498
S          -1.454216        0.708784       -0.674799
H           0.394370       -2.035674       -0.909275
```

4-hexene-3-one_HEI_4

Datum	Value
M06-2X/def2tzvpp-IEFPCM(water) Energy	-748.06377
M06-2X/def2tzvpp-IEFPCM(water) Free Energy (Quasiharmonic)	-747.910593
Number of Imaginary Frequencies	0

Frequencies (Top 3 out of 60)

```
1.      58.4939 cm-1
2.      76.7020 cm-1
3.      93.0273 cm-1
```

M06-2X/def2tzvpp-IEFPCM(water) Molecular Geometry in Cartesian Coordinates

```
C          -2.481115        1.745927        0.060036
C          -1.905640        0.573078        0.851199
C          -1.725181       -0.657333       -0.035862
C          -0.468970       -1.184733       -0.236612
C           0.818455       -0.704776        0.343488
C           1.859483       -1.814461        0.412293
O          -2.800522       -1.120387       -0.553244
H          -2.659460        2.611444        0.699337
H          -1.790825        2.047251       -0.730458
H          -3.424431        1.462343       -0.404758
H          -2.604081        0.303674        1.649005
H          -0.971466        0.878367        1.321316
H           0.689089       -0.283958        1.342162
H           1.498883       -2.612908        1.063636
H           2.028580       -2.234415       -0.580580
H           2.813553       -1.462590        0.804096
C           2.941543        1.204797        0.228122
H           2.719620        1.285722        1.292101
H           3.243258        2.183031       -0.140478
H           3.760900        0.504855        0.080367
S           1.453968        0.708831       -0.674797
H          -0.394383       -2.036015       -0.909022
```

4-hexene-3-one_HEI_5

Datum	Value
M06-2X/def2tzvpp-IEFPCM(water) Energy	-748.064202
M06-2X/def2tzvpp-IEFPCM(water) Free Energy (Quasiharmonic)	-747.911126
Number of Imaginary Frequencies	0

Frequencies (Top 3 out of 60)

```
1.      36.3523 cm-1
2.      67.6671 cm-1
3.      82.6115 cm-1
```

M06-2X/def2tzvpp-IEFPCM(water) Molecular Geometry in Cartesian Coordinates

```
C         -2.889371        0.153940        1.314555
C         -1.826083       -0.667218        0.586739
C         -1.396164        0.015723       -0.710926
C         -0.179675        0.656751       -0.792849
C          0.859190        0.768560        0.255603
C          1.526050        2.141009        0.287415
O         -2.259968       -0.029638       -1.655910
H         -3.278424       -0.370619        2.188087
H         -2.473657        1.105996        1.650501
H         -3.722357        0.367789        0.645115
H         -2.243899       -1.638401        0.310307
H         -0.988054       -0.859324        1.255190
H          0.461893        0.548349        1.245314
H          2.328657        2.188068        1.024965
H          1.945237        2.379597       -0.691810
H          0.784496        2.902476        0.530171
C          1.397698       -1.999353        0.063354
H          1.082228       -2.262919        1.071505
H          0.524184       -1.919702       -0.584425
H          2.061843       -2.772458       -0.316540
S          2.286267       -0.435493        0.040129
H          0.067458        1.119660       -1.746502
```

4-hexene-3-one_HEI_6

Datum	Value
M06-2X/def2tzvpp-IEFPCM(water) Energy	-748.064202
M06-2X/def2tzvpp-IEFPCM(water) Free Energy (Quasiharmonic)	-747.911126
Number of Imaginary Frequencies	0

Frequencies (Top 3 out of 60)

```
1.      36.3495 cm-1
2.      67.6657 cm-1
3.      82.6113 cm-1
```

M06-2X/def2tzvpp-IEFPCM(water) Molecular Geometry in Cartesian Coordinates

```
C          2.889364        0.153958        1.314560
C          1.826087       -0.667217        0.586746
C          1.396167        0.015706       -0.710929
C          0.179679        0.656733       -0.792860
C         -0.859183        0.768561        0.255592
C         -1.526028        2.141017        0.287396
O          2.259970       -0.029672       -1.655913
H          3.278416       -0.370589        2.188099
H          3.722351        0.367808        0.645122
H          2.473639        1.106014        1.650493
H          0.988055       -0.859324        1.255195
H          2.243912       -1.638399        0.310327
H         -0.461885        0.548353        1.245305
H         -1.945216        2.379601       -0.691829
H         -2.328632        2.188090        1.024949
H         -0.784465        2.902478        0.530143
C         -1.397720       -1.999349        0.063371
H         -1.082246       -2.262906        1.071524
H         -0.524208       -1.919715       -0.584413
H         -2.061876       -2.772449       -0.316509
S         -2.286271       -0.435479        0.040135
H         -0.067457        1.119627       -1.746519
```

4-hexene-3-one_HEI_7

Datum	Value
M06-2X/def2tzvpp-IEFPCM(water) Energy	-748.063817
M06-2X/def2tzvpp-IEFPCM(water) Free Energy (Quasiharmonic)	-747.911014

Datum	Value
Number of Imaginary Frequencies	0

Frequencies (Top 3 out of 60)

```
1.      40.5235 cm-1
2.      52.8324 cm-1
3.      83.9985 cm-1
```

M06-2X/def2tzvpp-IEFPCM(water) Molecular Geometry in Cartesian Coordinates

```
C         -2.583088      -1.321078       1.104950
C         -1.863115      -1.046094      -0.215009
C         -1.718065       0.456100      -0.458439
C         -0.535807       1.078527      -0.127089
C          0.665322       0.424758       0.466247
C          1.454404       1.370922       1.361867
O         -2.753696       1.042016      -0.929948
H         -2.773031      -2.385325       1.250005
H         -1.987618      -0.966425       1.948423
H         -3.540518      -0.799522       1.127603
H         -2.452915      -1.448398      -1.041055
H         -0.898130      -1.554182      -0.227629
H          0.398314      -0.464288       1.040898
H          1.744440       2.262517       0.803525
H          0.831457       1.682556       2.202488
H          2.356184       0.907336       1.761718
C          3.062702      -1.062247      -0.000401
H          2.631314      -1.703547       0.767964
H          3.759951      -0.364798       0.458434
H          3.603294      -1.684327      -0.710775
S          1.745203      -0.210725      -0.901955
H         -0.460542       2.148553      -0.303479
```

4-hexene-3-one_HEI_8

Datum	Value
M06-2X/def2tzvpp-IEFPCM(water) Energy	-748.063817
M06-2X/def2tzvpp-IEFPCM(water) Free Energy (Quasiharmonic)	-747.911014
Number of Imaginary Frequencies	0

Frequencies (Top 3 out of 60)

```
1.      40.5269 cm-1
2.      52.8405 cm-1
3.      83.9930 cm-1
```

M06-2X/def2tzvpp-IEFPCM(water) Molecular Geometry in Cartesian Coordinates

```
C          2.583049      -1.321038       1.105014
C          1.863054      -1.046136      -0.214951
C          1.718031       0.456045      -0.458471
C          0.535797       1.078543      -0.127135
C         -0.665375       0.424879       0.466256
C         -1.454382       1.371152       1.361825
O          2.753667       1.041900      -0.930043
H          2.772995      -2.385276       1.250129
H          3.540478      -0.799479       1.127616
H          1.987596      -0.966334       1.948478
H          0.898064      -1.554216      -0.227520
H          2.452851      -1.448493      -1.040974
H         -0.398398      -0.464140       1.040956
H         -0.831472       1.682674       2.202514
H         -1.744208       2.262804       0.803472
H         -2.356288       0.907728       1.761582
C         -3.062453      -1.062598      -0.000361
H         -3.759655      -0.365351       0.458857
H         -3.603181      -1.684494      -0.710793
H         -2.630824      -1.704096       0.767698
S         -1.745262      -0.210636      -0.901947
H          0.460581       2.148564      -0.303580
```

4-hexene-3-one_HEI_9

Datum	Value
M06-2X/def2tzvpp-IEFPCM(water) Energy	-748.06377
M06-2X/def2tzvpp-IEFPCM(water) Free Energy (Quasiharmonic)	-747.910593
Number of Imaginary Frequencies	0

Frequencies (Top 3 out of 60)

```
1.      58.4774 cm-1
2.      76.6780 cm-1
3.      93.0373 cm-1
```

M06-2X/def2tzvpp-IEFPCM(water) Molecular Geometry in Cartesian Coordinates

```
C         -2.480930       1.746002       0.059972
C         -1.905558       0.573133       0.851176
C         -1.725196      -0.657330      -0.035844
C         -0.469002      -1.184752      -0.236613
C          0.818419      -0.704794       0.343469
C          1.859438      -1.814495       0.412210
O         -2.800577      -1.120386      -0.553142
H         -2.659237       2.611549       0.699243
H         -1.790592       2.047249      -0.730508
H         -3.424251       1.462471      -0.404838
H         -2.604024       0.303804       1.648984
H         -0.971363       0.878366       1.321288
H          0.689066      -0.284047       1.342182
H          1.498867      -2.612939       1.063572
H          2.028461      -2.234448      -0.580677
H          2.813540      -1.462648       0.803951
C          2.941530       1.204758       0.228119
H          3.243122       2.183062      -0.140395
H          3.760941       0.504910       0.080227
H          2.719680       1.285550       1.292122
S          1.453915       0.708823      -0.674750
H         -0.394433      -2.036091      -0.908951
```

4-hexene-3-one_TS_10_reopt

Datum	Value
M06-2X/def2tzvpp-IEFPCM(water) Energy	-748.05415
M06-2X/def2tzvpp-IEFPCM(water) Free Energy (Quasiharmonic)	-747.901434
Number of Imaginary Frequencies	1

Frequencies (Top 3 out of 60)

```
1.    -205.8737 cm-1
2.      63.7505 cm-1
3.      85.5028 cm-1
```

M06-2X/def2tzvpp-IEFPCM(water) Molecular Geometry in Cartesian Coordinates

```
C         -1.576732      -1.495907      -1.079373
C         -2.074502      -0.055966      -0.946007
C         -1.653982       0.566280       0.378691
C         -0.347314       1.118296       0.524394
C          0.651160       1.095640      -0.433582
C          1.756396       2.115034      -0.399129
O         -2.488462       0.577344       1.301971
H         -1.907809      -2.095364      -0.229999
H         -1.963514      -1.954772      -1.989282
H         -0.487191      -1.527487      -1.111471
H         -3.163703      -0.034012      -0.969221
H         -1.711480       0.537315      -1.785581
H          0.381292       0.756318      -1.425565
H          1.374633       3.070090      -0.772477
H          2.109995       2.268170       0.619928
H          2.601247       1.820887      -1.018705
C          1.069321      -1.710776       1.075029
H          0.721424      -2.668970       0.690120
H          0.188906      -1.094886       1.300341
H          1.608466      -1.883737       2.005529
S          2.083702      -0.822835      -0.121474
H         -0.129893       1.557457       1.494073
```

4-hexene-3-one_TS_11_reopt

Datum	Value
M06-2X/def2tzvpp-IEFPCM(water) Energy	-748.049878
M06-2X/def2tzvpp-IEFPCM(water) Free Energy (Quasiharmonic)	-747.899097
Number of Imaginary Frequencies	1

Frequencies (Top 3 out of 60)

```
1.    -172.3877 cm-1
2.      33.7467 cm-1
3.      34.0205 cm-1
```

M06-2X/def2tzvpp-IEFPCM(water) Molecular Geometry in Cartesian Coordinates

```
C          -3.752244       -0.173325       -0.155052
C          -2.427112       -0.934806       -0.166586
C          -1.267597       -0.002468        0.157246
C          -0.318784        0.248329       -0.888710
C           0.784483        1.066723       -0.799381
C           0.958204        2.187672        0.184175
O          -1.222166        0.489797        1.292503
H          -4.590917       -0.843021       -0.341809
H          -3.904432        0.306302        0.810956
H          -3.759509        0.600792       -0.923778
H          -2.276718       -1.418396       -1.131297
H          -2.447233       -1.712542        0.600031
H           1.348159        1.186307       -1.715406
H           1.971159        2.582805        0.136768
H           0.264753        2.995412       -0.073775
H           0.738048        1.873490        1.197976
C           1.810420       -1.901266        0.173865
H           0.760780       -1.722685       -0.095677
H           1.828766       -2.315514        1.181753
H           2.211135       -2.648626       -0.510906
S           2.709344       -0.338282        0.080258
H          -0.440376       -0.335347       -1.792330
```

4-hexene-3-one_TS_12_reopt

Datum	Value
M06-2X/def2tzvpp-IEFPCM(water) Energy	-748.047733
M06-2X/def2tzvpp-IEFPCM(water) Free Energy (Quasiharmonic)	-747.895048
Number of Imaginary Frequencies	1

Frequencies (Top 3 out of 60)

```
1.    -194.6219 cm-1
2.      57.2530 cm-1
3.      71.2321 cm-1
```

M06-2X/def2tzvpp-IEFPCM(water) Molecular Geometry in Cartesian Coordinates

```
C           2.580510        0.839417       -1.124753
C           1.486436       -0.218028       -0.972546
C           1.357842       -0.675384        0.469226
C           0.304836       -0.179180        1.304876
```

```
C          -0.583263       0.855731       1.079146
C          -0.330795       2.121570       0.303746
O           2.172831      -1.517215       0.878992
H           2.361261       1.724992      -0.527841
H           3.537464       0.438110      -0.791340
H           2.684510       1.148719      -2.164396
H           1.759211      -1.102107      -1.551100
H           0.527652       0.140047      -1.346932
H          -1.273178       1.015156       1.898861
H           0.074376       1.956678      -0.688870
H          -1.254663       2.687202       0.199823
H           0.382633       2.737496       0.860833
C          -1.828405      -1.598293      -0.628533
H          -2.542216      -2.358980      -0.313646
H          -0.911266      -1.728872      -0.039317
H          -1.579452      -1.770349      -1.675698
S          -2.451897       0.071258      -0.355341
H           0.158405      -0.765492       2.206184
```

4-hexene-3-one_TS_13_reopt

Datum	Value
M06-2X/def2tzvpp-IEFPCM(water) Energy	-748.049819
M06-2X/def2tzvpp-IEFPCM(water) Free Energy (Quasiharmonic)	-747.899328
Number of Imaginary Frequencies	1

Frequencies (Top 3 out of 60)

```
1.    -223.6411 cm-1
2.      39.0879 cm-1
3.      42.4166 cm-1
```

M06-2X/def2tzvpp-IEFPCM(water) Molecular Geometry in Cartesian Coordinates

```
C          -4.086403      -0.092440      -0.305325
C          -2.930839      -0.118474       0.690983
C          -1.583763      -0.201670      -0.012198
C          -0.605021       0.789494       0.310890
C           0.632481       0.819307      -0.290030
C           1.506081       2.033378      -0.185496
O          -1.403162      -1.117434      -0.827949
H          -5.046935      -0.083609       0.208542
H          -4.032078       0.796082      -0.936110
```

```
H         -4.048889      -0.968117      -0.951004
H         -3.018428      -1.003398       1.327559
H         -2.964822       0.754990       1.341653
H          0.763030       0.200252      -1.169535
H          2.536595       1.825528      -0.461968
H          1.123431       2.803660      -0.862486
H          1.486405       2.439533       0.825341
C          3.439497      -0.665335      -0.638326
H          4.253749       0.019466      -0.400514
H          2.939150      -0.289102      -1.535834
H          3.872595      -1.633854      -0.886204
S          2.248149      -0.801067       0.726004
H         -0.841090       1.509559       1.085098
```

4-hexene-3-one_TS_14_reopt

Datum	Value
M06-2X/def2tzvpp-IEFPCM(water) Energy	-748.04985
M06-2X/def2tzvpp-IEFPCM(water) Free Energy (Quasiharmonic)	-747.899076
Number of Imaginary Frequencies	1

Frequencies (Top 3 out of 60)

```
1.    -223.4941 cm-1
2.      44.8797 cm-1
3.      47.5140 cm-1
```

M06-2X/def2tzvpp-IEFPCM(water) Molecular Geometry in Cartesian Coordinates

```
C          3.378952      -0.999362      -0.768793
C          3.060044       0.302994      -0.032045
C          1.643234       0.258541       0.514315
C          0.637546       0.977779      -0.202473
C         -0.683846       0.971208       0.184237
C         -1.649059       1.974647      -0.372654
O          1.426350      -0.425248       1.526165
H          4.402136      -0.998915      -1.142893
H          3.258121      -1.851270      -0.100196
H          2.707670      -1.136340      -1.617829
H          3.186159       1.152835      -0.703001
H          3.744087       0.425922       0.809073
H         -0.898877       0.586301       1.174177
H         -2.684316       1.694279      -0.195728
```

```
H          -1.473326        2.940975        0.110465
H          -1.496704        2.104854       -1.443644
C          -3.341329       -0.780926        0.498264
H          -4.157606       -0.299388       -0.040610
H          -3.062872       -0.130457        1.332823
H          -3.716348       -1.713101        0.919467
S          -1.901314       -1.068977       -0.570662
H           0.928841        1.500652       -1.105927
```

4-hexene-3-one_TS_15_reopt

Datum	Value
M06-2X/def2tzvpp-IEFPCM(water) Energy	-748.050298
M06-2X/def2tzvpp-IEFPCM(water) Free Energy (Quasiharmonic)	-747.899053
Number of Imaginary Frequencies	1

Frequencies (Top 3 out of 60)

```
1.    -168.7824 cm-1
2.      38.3213 cm-1
3.      60.1603 cm-1
```

M06-2X/def2tzvpp-IEFPCM(water) Molecular Geometry in Cartesian Coordinates

```
C           3.127949       -1.078806       -0.317210
C           2.608872        0.051225        0.567057
C           1.284257        0.603062        0.052520
C           0.197723        0.666809        0.985246
C          -1.075613        1.117523        0.713563
C          -1.444165        2.062032       -0.393998
O           1.224575        0.954863       -1.133538
H           4.100652       -1.429898        0.025396
H           2.438740       -1.924817       -0.305889
H           3.226750       -0.738070       -1.346387
H           3.325622        0.877221        0.560274
H           2.508287       -0.282941        1.599192
H          -1.746210        1.147041        1.562579
H          -2.525642        2.132002       -0.493166
H          -1.060230        3.058362       -0.149154
H          -1.009321        1.762769       -1.340622
C          -1.103770       -2.081073       -0.023043
H          -1.361054       -2.877787        0.674696
H          -0.191243       -1.590880        0.342992
```

```
H          -0.872982       -2.531444       -0.988389
S          -2.411197       -0.844370       -0.158437
H           0.377670        0.224822        1.956961
```

4-hexene-3-one_TS_16_reopt

Datum	Value
M06-2X/def2tzvpp-IEFPCM(water) Energy	-748.049086
M06-2X/def2tzvpp-IEFPCM(water) Free Energy (Quasiharmonic)	-747.898024
Number of Imaginary Frequencies	1

Frequencies (Top 3 out of 60)

```
1.    -168.4572 cm-1
2.      15.5218 cm-1
3.      43.1267 cm-1
```

M06-2X/def2tzvpp-IEFPCM(water) Molecular Geometry in Cartesian Coordinates

```
C          -3.016920       -1.062788       -0.778567
C          -2.650692       -0.151197        0.383364
C          -1.290967        0.544686        0.364068
C          -0.446597        0.387613       -0.781520
C           0.811291        0.930578       -0.929956
C           1.341503        2.118788       -0.180910
O          -1.001734        1.216185        1.365419
H          -4.002943       -1.498292       -0.618587
H          -3.043264       -0.518612       -1.722532
H          -2.303933       -1.881365       -0.881946
H          -2.687926       -0.705915        1.323963
H          -3.393065        0.645138        0.483668
H           1.271120        0.772860       -1.896686
H           2.397303        2.266679       -0.399206
H           0.796740        3.012862       -0.503076
H           1.200997        2.016791        0.889005
C           1.260292       -1.946495        0.579177
H           0.253315       -1.583704        0.331468
H           1.309664       -2.077899        1.659995
H           1.390383       -2.921141        0.109111
S           2.472603       -0.743292       -0.005047
H          -0.783624       -0.291321       -1.551713
```

4-hexene-3-one_TS_17_reopt

Datum	Value
M06-2X/def2tzvpp-IEFPCM(water) Energy	-748.04856
M06-2X/def2tzvpp-IEFPCM(water) Free Energy (Quasiharmonic)	-747.897033
Number of Imaginary Frequencies	1

Frequencies (Top 3 out of 60)

```
1.    -206.4009 cm-1
2.      21.8536 cm-1
3.      43.9455 cm-1
```

M06-2X/def2tzvpp-IEFPCM(water) Molecular Geometry in Cartesian Coordinates

```
C          2.705552       -0.833071       -1.198140
C          1.535047        0.072635       -0.852174
C          1.378583        0.307495        0.645135
C          0.140711        0.812209        1.151320
C         -0.976236        1.253689        0.453494
C         -0.983996        1.976289       -0.871036
O          2.321304        0.010251        1.397256
H          2.768806       -0.981873       -2.275851
H          3.646374       -0.407459       -0.854049
H          2.593459       -1.808957       -0.725189
H          0.596165       -0.343628       -1.228123
H          1.653506        1.043751       -1.340069
H         -1.768242        1.604220        1.104084
H         -0.477319        2.939601       -0.756253
H         -0.493529        1.428078       -1.667475
H         -2.009443        2.169368       -1.182379
C         -1.275576       -1.930682        0.377156
H         -1.807813       -2.569157        1.081293
H         -0.430203       -1.470173        0.905081
H         -0.873573       -2.553660       -0.421768
S         -2.322670       -0.613633       -0.267602
H          0.049582        0.734619        2.229749
```

4-hexene-3-one_TS_18_reopt

Datum	Value
M06-2X/def2tzvpp-IEFPCM(water) Energy	-748.048868
M06-2X/def2tzvpp-IEFPCM(water) Free Energy (Quasiharmonic)	-747.898071
Number of Imaginary Frequencies	1

Frequencies (Top 3 out of 60)

```
1.    -225.5036 cm-1
2.      35.3240 cm-1
3.      62.2193 cm-1
```

M06-2X/def2tzvpp-IEFPCM(water) Molecular Geometry in Cartesian Coordinates

```
C         -3.578330        0.643323        0.779377
C         -3.032389       -0.573344        0.046423
C         -1.578631       -0.540144       -0.412719
C         -0.771422        0.602139       -0.117049
C          0.536281        0.687947       -0.536183
C          1.268281        1.995793       -0.503990
O         -1.165718       -1.530276       -1.035787
H         -3.506297        1.542430        0.167185
H         -4.628184        0.493386        1.029650
H         -3.038242        0.825937        1.708363
H         -3.627106       -0.773235       -0.848279
H         -3.132437       -1.465460        0.670100
H          0.862474       -0.034714       -1.274233
H          1.052589        2.539206        0.415410
H          2.344044        1.868180       -0.594308
H          0.929278        2.611398       -1.343272
C          3.492892       -0.506917       -0.233386
H          4.040394       -1.448575       -0.258804
H          4.198606        0.289375        0.004347
H          3.109588       -0.321795       -1.241488
S          2.117801       -0.570927        0.951809
H         -1.183875        1.398117        0.487837
```

4-hexene-3-one_TS_19

Datum	Value
M06-2X/def2tzvpp-IEFPCM(water) Energy	-748.049738
M06-2X/def2tzvpp-IEFPCM(water) Free Energy (Quasiharmonic)	-747.898521

Datum	Value
Number of Imaginary Frequencies	1

Frequencies (Top 3 out of 60)

```
1.    -247.5574 cm-1
2.      46.6275 cm-1
3.      62.6886 cm-1
```

M06-2X/def2tzvpp-IEFPCM(water) Molecular Geometry in Cartesian Coordinates

```
C         -2.359596       1.415076       1.204432
C         -1.822719       1.117120      -0.197389
C         -1.847069      -0.376789      -0.479252
C         -0.735941      -1.175207      -0.087286
C          0.404369      -0.692160       0.530391
C          1.341440      -1.653176       1.205885
O         -2.863567      -0.853738      -1.015215
H         -2.401276       2.488250       1.388729
H         -3.366563       1.012317       1.319143
H         -1.727299       0.963103       1.969151
H         -0.812860       1.513321      -0.310984
H         -2.459764       1.593982      -0.942291
H          0.370099       0.297252       0.970093
H          2.303476      -1.200226       1.432723
H          0.891954      -1.986193       2.146358
H          1.504075      -2.532908       0.583989
C          3.012130       1.088110       0.254586
H          2.428493       1.223488       1.170369
H          3.887150       0.487292       0.502657
H          3.353699       2.071648      -0.065771
S          1.985619       0.299383      -1.018087
H         -0.788230      -2.229406      -0.341262
```

4-hexene-3-one_TS_1_reopt

Datum	Value
M06-2X/def2tzvpp-IEFPCM(water) Energy	-748.05505
M06-2X/def2tzvpp-IEFPCM(water) Free Energy (Quasiharmonic)	-747.903738
Number of Imaginary Frequencies	1

Frequencies (Top 3 out of 60)

```
1.    -181.1460 cm-1
2.      36.6120 cm-1
3.      65.1398 cm-1
```

M06-2X/def2tzvpp-IEFPCM(water) Molecular Geometry in Cartesian Coordinates

```
C         -3.274951        0.762652       -0.285471
C         -2.565577       -0.572269       -0.490744
C         -1.260962       -0.645793        0.290002
C         -0.086035       -1.072747       -0.411486
C          1.135922       -1.146352        0.209769
C          2.296707       -1.865104       -0.408955
O         -1.275536       -0.342947        1.490771
H         -4.231648        0.782718       -0.806118
H         -3.454262        0.936431        0.774077
H         -2.665108        1.584807       -0.663584
H         -2.381603       -0.753763       -1.549529
H         -3.203965       -1.383587       -0.129006
H          1.131954       -1.077280        1.288852
H          3.244089       -1.549321        0.022191
H          2.187991       -2.940239       -0.236020
H          2.331366       -1.700312       -1.485498
C          0.710114        2.038004       -0.420794
H          0.262322        2.679446        0.338646
H         -0.045349        1.301968       -0.728329
H          0.950938        2.648823       -1.290456
S          2.157003        1.155581        0.194972
H         -0.165793       -1.265765       -1.474873
```

4-hexene-3-one_TS_2_reopt

Datum	Value
M06-2X/def2tzvpp-IEFPCM(water) Energy	-748.054767
M06-2X/def2tzvpp-IEFPCM(water) Free Energy (Quasiharmonic)	-747.903695
Number of Imaginary Frequencies	1

Frequencies (Top 3 out of 60)

```
1.    -183.4377 cm-1
2.      27.4895 cm-1
3.      63.8170 cm-1
```

M06-2X/def2tzvpp-IEFPCM(water) Molecular Geometry in Cartesian Coordinates

```
C          3.800631       -0.209970        0.033779
C          2.505057       -0.005878       -0.734155
C          1.295714        0.236961        0.162837
C          0.172315        0.892505       -0.436409
C         -0.983654        1.142813        0.267203
C         -2.006023        2.122670       -0.229039
O          1.327557       -0.148116        1.339384
H          4.628452       -0.404238       -0.647425
H          4.046185        0.673815        0.623454
H          3.714471       -1.050332        0.719946
H          2.281889       -0.892978       -1.336000
H          2.593759        0.820379       -1.441520
H         -0.922489        1.020155        1.340001
H         -2.969045        1.984498        0.258064
H         -1.662941        3.140281       -0.019203
H         -2.144960        2.030219       -1.305892
C         -1.226549       -1.993387       -0.512218
H         -0.891759       -2.745806        0.201945
H         -0.351459       -1.412874       -0.831468
H         -1.627667       -2.502355       -1.388088
S         -2.430774       -0.858506        0.205519
H          0.232553        1.145974       -1.489175
```

4-hexene-3-one_TS_3_reopt

Datum	Value
M06-2X/def2tzvpp-IEFPCM(water) Energy	-748.05397
M06-2X/def2tzvpp-IEFPCM(water) Free Energy (Quasiharmonic)	-747.902637
Number of Imaginary Frequencies	1

Frequencies (Top 3 out of 60)

```
1.    -171.2137 cm-1
2.      41.2803 cm-1
3.      58.2918 cm-1
```

M06-2X/def2tzvpp-IEFPCM(water) Molecular Geometry in Cartesian Coordinates

```
C          2.997330        0.030284       -1.278872
C          2.673984       -0.322935        0.165365
C          1.308498        0.079823        0.710727
C          0.414870        0.844529       -0.110307
C         -0.832794        1.197721        0.335316
C         -1.667520        2.220924       -0.372539
O          1.036617       -0.279767        1.864659
H          2.284911       -0.424627       -1.967771
H          2.980055        1.107985       -1.440858
H          3.991683       -0.329453       -1.541575
H          3.412475        0.122671        0.837023
H          2.752032       -1.402109        0.317766
H         -1.030986        1.057066        1.388724
H         -1.560388        2.134269       -1.453398
H         -2.720968        2.128457       -0.118146
H         -1.335492        3.221864       -0.079911
C         -1.101683       -1.955784       -0.553470
H         -0.952564       -2.733410        0.196004
H         -0.152055       -1.417196       -0.674448
H         -1.334256       -2.434329       -1.504517
S         -2.380472       -0.777552       -0.070487
H          0.704053        1.080407       -1.125699
```

4-hexene-3-one_TS_4

Datum	Value
M06-2X/def2tzvpp-IEFPCM(water) Energy	-748.055054
M06-2X/def2tzvpp-IEFPCM(water) Free Energy (Quasiharmonic)	-747.903184
Number of Imaginary Frequencies	1

Frequencies (Top 3 out of 60)

```
1.    -202.5888 cm-1
2.      51.5162 cm-1
3.      62.7588 cm-1
```

M06-2X/def2tzvpp-IEFPCM(water) Molecular Geometry in Cartesian Coordinates

```
C          -2.590128        0.271863       -1.516519
C          -1.708351       -0.668104       -0.691979
C          -1.452642       -0.094118        0.693406
C          -0.313709        0.738313        0.898550
C           0.571059        1.117519       -0.094086
C           1.507976        2.271139        0.127701
O          -2.272010       -0.351621        1.593071
H          -2.828283       -0.163528       -2.486550
H          -3.526602        0.469217       -0.994061
H          -2.091331        1.226645       -1.685893
H          -0.768848       -0.862750       -1.211230
H          -2.218336       -1.621692       -0.552570
H           0.268256        0.974986       -1.121409
H           0.956715        3.210576        0.027933
H           1.935787        2.236350        1.129074
H           2.319891        2.276489       -0.596931
C           1.392414       -1.959072        0.326310
H           1.001611       -2.665608       -0.405865
H           0.547699       -1.555803        0.898862
H           2.035541       -2.499015        1.020104
S           2.264827       -0.583723       -0.446615
H          -0.132970        1.061423        1.919506
```

4-hexene-3-one_TS_5_reopt

Datum	Value
M06-2X/def2tzvpp-IEFPCM(water) Energy	-748.055637
M06-2X/def2tzvpp-IEFPCM(water) Free Energy (Quasiharmonic)	-747.90355
Number of Imaginary Frequencies	1

Frequencies (Top 3 out of 60)

```
1.    -212.9409 cm-1
2.      49.6738 cm-1
3.      70.8098 cm-1
```

M06-2X/def2tzvpp-IEFPCM(water) Molecular Geometry in Cartesian Coordinates

```
C           3.017708        0.809079       -0.783633
C           1.677735        0.099030       -0.877300
C           1.336166       -0.716021        0.368985
C           0.001666       -1.188957        0.511050
```

```
C        -1.003840      -0.990585      -0.426341
C        -2.253143      -1.826605      -0.367316
O         2.227410      -0.935610       1.208932
H         3.216911       1.376309      -1.692662
H         3.030740       1.500962       0.059022
H         3.829176       0.098489      -0.636893
H         1.666516      -0.577154      -1.737564
H         0.873194       0.817560      -1.054090
H        -0.700763      -0.715959      -1.427084
H        -2.037102      -2.830207      -0.744331
H        -2.606967      -1.920823       0.659059
H        -3.053177      -1.402907      -0.971816
C        -0.774513       1.906544       0.877869
H        -0.196887       2.661689       0.344755
H        -0.085890       1.125846       1.225666
H        -1.225838       2.367954       1.755043
S        -2.018810       1.132000      -0.169855
H        -0.238917      -1.683784       1.447237
```

4-hexene-3-one_TS_6_reopt

Datum	Value
M06-2X/def2tzvpp-IEFPCM(water) Energy	-748.05469
M06-2X/def2tzvpp-IEFPCM(water) Free Energy (Quasiharmonic)	-747.903291
Number of Imaginary Frequencies	1

Frequencies (Top 3 out of 60)

```
1.    -173.0754 cm-1
2.      40.2993 cm-1
3.      64.4200 cm-1
```

M06-2X/def2tzvpp-IEFPCM(water) Molecular Geometry in Cartesian Coordinates

```
C         3.796272       0.091806      -0.054945
C         2.489571      -0.429577      -0.649244
C         1.295282      -0.016951       0.197600
C         0.280671       0.784933      -0.423737
C        -0.832163       1.198703       0.262157
C        -1.738192       2.268710      -0.266700
O         1.260686      -0.380723       1.380526
H         3.804492       1.182602      -0.037288
H         3.915105      -0.264612       0.967009
```

```
H          4.652989       -0.243238       -0.638362
H          2.510522       -1.522107       -0.673007
H          2.368419       -0.079312       -1.674024
H         -0.815587        1.066795        1.335069
H         -1.863784        2.178085       -1.345214
H         -2.718968        2.235408        0.202257
H         -1.297216        3.248663       -0.059761
C         -1.447699       -1.931574       -0.531001
H         -0.513031       -1.446281       -0.842459
H         -1.896470       -2.384823       -1.414523
H         -1.195170       -2.724934        0.172818
S         -2.529925       -0.687739        0.202393
H          0.379562        1.007055       -1.479794
```

4-hexene-3-one_TS_7

Datum	Value
M06-2X/def2tzvpp-IEFPCM(water) Energy	-748.055054
M06-2X/def2tzvpp-IEFPCM(water) Free Energy (Quasiharmonic)	-747.903186
Number of Imaginary Frequencies	1

Frequencies (Top 3 out of 60)

```
1.    -202.6039 cm-1
2.      51.4947 cm-1
3.      62.6939 cm-1
```

M06-2X/def2tzvpp-IEFPCM(water) Molecular Geometry in Cartesian Coordinates

```
C         -2.590726        0.271879       -1.516124
C         -1.708381       -0.667972       -0.692069
C         -1.452559       -0.094234        0.693405
C         -0.313603        0.738124        0.898629
C          0.571050        1.117525       -0.094047
C          1.507870        2.271244        0.127613
O         -2.271913       -0.351863        1.593042
H         -3.527206        0.468600       -0.993445
H         -2.092390        1.226955       -1.685204
H         -2.828819       -0.163251       -2.486288
H         -0.768972       -0.862153       -1.211653
H         -2.217961       -1.621793       -0.552771
H          0.268074        0.974884       -1.121307
H          0.956486        3.210621        0.027994
```

```
H          1.935890      2.236459      1.128897
H          2.319641      2.276714     -0.597183
C          1.392693     -1.959220      0.325882
H          0.547879     -1.556255      0.898499
H          2.035851     -2.499246      1.019587
H          1.002077     -2.665610     -0.406531
S          2.264968     -0.583510     -0.446518
H         -0.132801      1.061073      1.919624
```

4-hexene-3-one_TS_8_reopt

Datum	Value
M06-2X/def2tzvpp-IEFPCM(water) Energy	-748.05505
M06-2X/def2tzvpp-IEFPCM(water) Free Energy (Quasiharmonic)	-747.903736
Number of Imaginary Frequencies	1

Frequencies (Top 3 out of 60)

```
1.    -181.1412 cm-1
2.      36.6343 cm-1
3.      65.1604 cm-1
```

M06-2X/def2tzvpp-IEFPCM(water) Molecular Geometry in Cartesian Coordinates

```
C         -3.274826      0.762670     -0.285473
C         -2.565533     -0.572288     -0.490787
C         -1.260939     -0.645868      0.289983
C         -0.085987     -1.072764     -0.411482
C          1.135966     -1.146283      0.209780
C          2.296758     -1.865019     -0.408953
O         -1.275557     -0.343074      1.490770
H         -4.231590      0.782773     -0.805996
H         -3.453989      0.936475      0.774098
H         -2.664983      1.584782     -0.663681
H         -2.381562     -0.753771     -1.549575
H         -3.203968     -1.383574     -0.129061
H          1.132008     -1.077209      1.288863
H          3.244147     -1.549139      0.022106
H          2.188114     -2.940145     -0.235915
H          2.331340     -1.700326     -1.485515
C          0.709940      2.037963     -0.420709
H         -0.045327      1.301784     -0.728411
H          0.950730      2.649000     -1.290228
```

```
H          0.261934       2.679157       0.338813
S          2.156940       1.155634       0.194941
H         -0.165713      -1.265827      -1.474867
```

4-hexene-3-one_TS_9_reopt

Datum	Value
M06-2X/def2tzvpp-IEFPCM(water) Energy	-748.05397
M06-2X/def2tzvpp-IEFPCM(water) Free Energy (Quasiharmonic)	-747.902638
Number of Imaginary Frequencies	1

Frequencies (Top 3 out of 60)

```
1.    -171.1934 cm-1
2.      41.2341 cm-1
3.      58.3964 cm-1
```

M06-2X/def2tzvpp-IEFPCM(water) Molecular Geometry in Cartesian Coordinates

```
C          2.997164       0.029733      -1.279009
C          2.674039      -0.322717       0.165468
C          1.308544       0.080114       0.710763
C          0.414870       0.844602      -0.110426
C         -0.832809       1.197844       0.335133
C         -1.667637       2.220749      -0.373032
O          1.036702      -0.279185       1.864795
H          3.991477      -0.330142      -1.541669
H          2.284639      -0.425550      -1.967554
H          2.979867       1.107345      -1.441578
H          3.412525       0.123419       0.836781
H          2.752312      -1.401785       0.318486
H         -1.031024       1.057449       1.388577
H         -2.721063       2.128307      -0.118536
H         -1.335651       3.221817      -0.080794
H         -1.560577       2.133699      -1.453868
C         -1.101669      -1.955890      -0.553360
H         -0.151983      -1.417326      -0.673916
H         -1.334040      -2.434106      -1.504621
H         -0.952819      -2.733782       0.195890
S         -2.380392      -0.777667      -0.070178
H          0.703992       1.080198      -1.125914
```

5_3-methyl-2-cyclopentene-1-one_1

Datum	Value
M06-2X/def2tzvpp-IEFPCM(water) Energy	-308.660411
M06-2X/def2tzvpp-IEFPCM(water) Free Energy (Quasiharmonic)	-308.561666
Number of Imaginary Frequencies	0

Frequencies (Top 3 out of 39)

```
1.      99.8504 cm-1
2.     140.7928 cm-1
3.     186.3999 cm-1
```

M06-2X/def2tzvpp-IEFPCM(water) Molecular Geometry in Cartesian Coordinates

```
C         -0.581171        1.273116        0.000001
C          0.946705        1.200376       -0.000001
C          1.268077       -0.287146       -0.000001
C         -0.001629       -1.019500       -0.000000
C         -1.032714       -0.164830        0.000001
H         -0.980918        1.788566        0.875083
H         -0.980920        1.788567       -0.875081
H          1.398262        1.661104        0.877949
H          1.398260        1.661104       -0.877951
O          2.385814       -0.761401       -0.000002
H         -0.063578       -2.098230       -0.000000
C         -2.479877       -0.502501        0.000002
H         -2.647401       -1.576779        0.000001
H         -2.963283       -0.065107        0.875917
H         -2.963284       -0.065107       -0.875913
```

5_3methyl2cyclopentene1one_HEI_1

Datum	Value
M06-2X/def2tzvpp-IEFPCM(water) Energy	-746.870798
M06-2X/def2tzvpp-IEFPCM(water) Free Energy (Quasiharmonic)	-746.736194
Number of Imaginary Frequencies	0

Frequencies (Top 3 out of 54)

```
1.      92.5471 cm-1
2.     116.2500 cm-1
3.     168.9810 cm-1
```

M06-2X/def2tzvpp-IEFPCM(water) Molecular Geometry in Cartesian Coordinates

```
C           1.487548       -0.151060        1.164888
C           0.165890        0.593616        1.368476
C          -0.466528        0.735466       -0.038671
C           0.673414        0.500787       -0.956404
C           1.782818       -0.024003       -0.334600
H           2.306510        0.271972        1.749460
H           1.421986       -1.210501        1.427592
H           0.363723        1.598896        1.747269
H          -0.509345        0.109730        2.074606
O           2.906045       -0.387664       -0.807153
H           0.587032        0.644226       -2.027605
C          -0.984845       -2.059217        0.067714
H          -0.783289       -2.174011        1.131681
H          -0.046303       -2.069144       -0.486750
H          -1.610528       -2.883771       -0.266513
S          -1.857491       -0.524060       -0.273120
C          -1.187917        2.067535       -0.223108
H          -0.473273        2.881385       -0.091615
H          -1.613719        2.147263       -1.224851
H          -1.993568        2.191476        0.504090
```

5_3methyl2cyclopentene1one_HEI_2

Datum	Value
M06-2X/def2tzvpp-IEFPCM(water) Energy	-746.869627
M06-2X/def2tzvpp-IEFPCM(water) Free Energy (Quasiharmonic)	-746.735811
Number of Imaginary Frequencies	0

Frequencies (Top 3 out of 54)

```
1.      65.3372 cm-1
2.     104.1520 cm-1
3.     141.3933 cm-1
```

M06-2X/def2tzvpp-IEFPCM(water) Molecular Geometry in Cartesian Coordinates

```
C          1.787965      -0.520758      -0.934288
C          0.630526      -1.444478      -0.561363
C         -0.295811      -0.583850       0.320339
C          0.627500       0.432041       0.886938
C          1.816473       0.517035       0.196193
H          2.745909      -1.035678      -1.019572
H          1.608335      -0.005448      -1.882883
H          0.998993      -2.267982       0.055143
H          0.110036      -1.881562      -1.416387
O          2.816551       1.284040       0.353578
H          0.349676       1.083195       1.706795
C         -2.233427       1.492734       0.110775
H         -2.770196       1.116605       0.980255
H         -1.430532       2.153353       0.433932
H         -2.926876       2.052440      -0.513277
S         -1.562190       0.151031      -0.884492
C         -1.072793      -1.404190       1.341796
H         -0.373234      -1.877187       2.034706
H         -1.747487      -0.775458       1.925258
H         -1.664587      -2.182296       0.856939
```

5_3methyl2cyclopentene1one_HEI_3

Datum	Value
M06-2X/def2tzvpp-IEFPCM(water) Energy	-746.867217
M06-2X/def2tzvpp-IEFPCM(water) Free Energy (Quasiharmonic)	-746.733283
Number of Imaginary Frequencies	0

Frequencies (Top 3 out of 54)

```
1.      59.7448 cm-1
2.      75.4790 cm-1
3.     134.2102 cm-1
```

M06-2X/def2tzvpp-IEFPCM(water) Molecular Geometry in Cartesian Coordinates

```
C          -1.753562       1.165645       -0.455093
C          -0.223821       1.207305       -0.503598
C           0.239299      -0.003935        0.344756
C          -0.946869      -0.919254        0.303443
C          -2.094335      -0.290694       -0.114514
H          -2.160843       1.810685        0.329641
H          -2.220405       1.470400       -1.393348
H           0.198694       2.142927       -0.133589
H           0.113342       1.074893       -1.531877
O          -3.292390      -0.712825       -0.197562
H          -0.913738      -1.922798        0.712029
C           2.965763       0.373329       -0.414511
H           3.342677       0.549145        0.590435
H           2.599429       1.307749       -0.837885
H           3.780098       0.005068       -1.035100
S           1.663700      -0.879292       -0.439391
C           0.624296       0.423229        1.763101
H          -0.209414       0.976484        2.202677
H           0.822741      -0.448920        2.386935
H           1.502714       1.071891        1.779323
```

5_3methyl2cyclopentene1one_TS_1

Datum	Value
M06-2X/def2tzvpp-IEFPCM(water) Energy	-746.862263
M06-2X/def2tzvpp-IEFPCM(water) Free Energy (Quasiharmonic)	-746.729397
Number of Imaginary Frequencies	1

Frequencies (Top 3 out of 54)

```
1.    -219.4180 cm-1
2.      61.9380 cm-1
3.      96.3570 cm-1
```

M06-2X/def2tzvpp-IEFPCM(water) Molecular Geometry in Cartesian Coordinates

```
C           1.433113       0.003805        1.219717
C           0.362060       1.090795        1.203330
C          -0.219776       0.992720       -0.197472
C           0.739393       0.403938       -1.020050
C           1.771217      -0.200596       -0.256705
H           2.322401       0.244971        1.800308
```

```
H          1.023919      -0.936784       1.601244
H          0.824719       2.078964       1.300206
H         -0.391968       0.993576       1.979800
O          2.782469      -0.807190      -0.642964
H          0.680764       0.349447      -2.099029
C         -1.051060      -2.117792      -0.163612
H         -0.791227      -2.659177       0.746085
H         -0.122427      -1.846775      -0.679309
H         -1.613724      -2.785423      -0.815085
S         -1.970236      -0.604597       0.181102
C         -1.135010       2.087189      -0.661378
H         -0.564593       3.019735      -0.719106
H         -1.543936       1.873596      -1.647079
H         -1.959537       2.238587       0.035059
```

5_3methyl2cyclopentene1one_TS_2

Datum	Value
M06-2X/def2tzvpp-IEFPCM(water) Energy	-746.862263
M06-2X/def2tzvpp-IEFPCM(water) Free Energy (Quasiharmonic)	-746.729394
Number of Imaginary Frequencies	1

Frequencies (Top 3 out of 54)

```
1.    -219.3785 cm-1
2.      62.1772 cm-1
3.      96.3748 cm-1
```

M06-2X/def2tzvpp-IEFPCM(water) Molecular Geometry in Cartesian Coordinates

```
C          1.432899      -0.002864      -1.219741
C          0.362256      -1.090238      -1.203652
C         -0.219424      -0.992907       0.197240
C          0.739655      -0.404197       1.019959
C          1.771360       0.200694       0.256708
H          2.322060      -0.243235      -1.800847
H          1.023145       0.937834      -1.600402
H          0.825300      -2.078186      -1.300948
H         -0.391946      -0.993023      -1.979948
O          2.782712       0.807014       0.643095
H          0.681142      -0.350085       2.098961
C         -1.051298       2.117513       0.163680
H         -1.613913       2.785410       0.814929
```

```
H          -0.122688       1.846629       0.679469
H          -0.791433       2.658599      -0.746205
S          -1.970762       0.604391      -0.180584
C          -1.134547      -2.087537       0.660857
H          -0.563942      -3.019957       0.718774
H          -1.543860      -1.874031       1.646401
H          -1.958775      -2.239108      -0.035909
```

5_3methyl2cyclopentene1one_TS_3

Datum	Value
M06-2X/def2tzvpp-IEFPCM(water) Energy	-746.85844
M06-2X/def2tzvpp-IEFPCM(water) Free Energy (Quasiharmonic)	-746.726421
Number of Imaginary Frequencies	1

Frequencies (Top 3 out of 54)

```
1.    -256.6556 cm-1
2.      75.8278 cm-1
3.      81.5561 cm-1
```

M06-2X/def2tzvpp-IEFPCM(water) Molecular Geometry in Cartesian Coordinates

```
C          -1.532121      -0.641998      -1.147291
C          -0.356170       0.304382      -1.373845
C          -0.037370       0.825313       0.013411
C          -1.158344       0.647349       0.820429
C          -2.097380      -0.220193       0.208749
H          -2.298910      -0.603907      -1.920161
H          -1.191398      -1.677067      -1.062283
H          -0.664980       1.160316      -1.983443
H           0.503433      -0.153504      -1.859115
O          -3.191393      -0.617963       0.637637
H          -1.270827       1.050511       1.817761
C           3.014358      -0.594406      -0.341555
H           3.633605       0.214311       0.047473
H           2.715955      -0.330863      -1.358939
H           3.628395      -1.493200      -0.400546
S           1.548037      -0.874923       0.695150
C           0.893372       1.991652       0.148756
H           0.399996       2.875866      -0.268830
H           1.128287       2.192470       1.192384
H           1.820926       1.834955      -0.399724
```

6_3pentene2one_HEI_1

Datum	Value
M06-2X/def2tzvpp-IEFPCM(water) Energy	-708.760776
M06-2X/def2tzvpp-IEFPCM(water) Free Energy (Quasiharmonic)	-708.635078
Number of Imaginary Frequencies	0

Frequencies (Top 3 out of 51)

```
 1.     65.5606 cm-1
 2.     86.5236 cm-1
 3.     97.8224 cm-1
```

M06-2X/def2tzvpp-IEFPCM(water) Molecular Geometry in Cartesian Coordinates

```
C          2.993217       0.019702      -0.731518
C          1.729053       0.050976       0.117794
C          0.613341       0.653837      -0.415766
C         -0.671622       0.757231       0.311136
O          1.820706      -0.488611       1.274441
C         -1.347512       2.117383       0.173047
H          3.807191       0.508958      -0.192054
H          3.296134      -1.017399      -0.893241
H          2.868934       0.504788      -1.699342
H          0.654447       1.063161      -1.419433
H         -0.505833       0.526610       1.363408
H         -2.312809       2.148782       0.681358
H         -0.706795       2.890271       0.598289
H         -1.507983       2.355929      -0.880260
S         -1.953357      -0.476734      -0.259539
C         -1.038958      -2.012308      -0.047305
H         -0.042987      -1.886025      -0.470312
H         -0.957051      -2.281212       1.004061
H         -1.570292      -2.798166      -0.579703
```

6_3pentene2one_HEI_2_reopt2

Datum	Value

Datum	Value
M06-2X/def2tzvpp-IEFPCM(water) Energy	-708.759249
M06-2X/def2tzvpp-IEFPCM(water) Free Energy (Quasiharmonic)	-708.633682
Number of Imaginary Frequencies	0

Frequencies (Top 3 out of 51)

```
1.      45.5116 cm-1
2.      77.8442 cm-1
3.     106.5720 cm-1
```

M06-2X/def2tzvpp-IEFPCM(water) Molecular Geometry in Cartesian Coordinates

```
C         -3.033129      -0.331326       0.834564
C         -1.854014      -0.047554      -0.085620
C         -0.694482       0.433602       0.476358
C          0.497005       0.776590      -0.337065
O         -2.042655      -0.275954      -1.329714
C          1.304896       1.930747       0.244763
H         -3.894005       0.261327       0.516975
H         -3.320023      -1.381842       0.748673
H         -2.818830      -0.109573       1.879733
H         -0.637157       0.577949       1.548718
H          0.180154       1.016723      -1.353569
H          2.177088       2.163841      -0.365628
H          0.678347       2.821529       0.316966
H          1.647433       1.689667       1.252976
S          1.637678      -0.667835      -0.674496
C          2.060728      -1.152521       1.008287
H          2.716470      -0.427045       1.485782
H          1.149180      -1.266534       1.593893
H          2.573703      -2.110271       0.957400
```

6_3pentene2one_HEI_3

Datum	Value
M06-2X/def2tzvpp-IEFPCM(water) Energy	-708.757306
M06-2X/def2tzvpp-IEFPCM(water) Free Energy (Quasiharmonic)	-708.631745
Number of Imaginary Frequencies	0

Frequencies (Top 3 out of 51)

```
1.      62.9190 cm-1
2.      90.5919 cm-1
3.      95.5811 cm-1
```

M06-2X/def2tzvpp-IEFPCM(water) Molecular Geometry in Cartesian Coordinates

```
C         1.990940      -0.612095       1.123559
C         1.796405       0.195803      -0.154623
C         0.587877       0.791920      -0.437478
C        -0.663481       0.744959       0.351633
O         2.834123       0.266828      -0.902281
C        -1.436782       2.060031       0.316621
H         1.124478      -0.654628       1.779172
H         2.262510      -1.634603       0.851855
H         2.832412      -0.194049       1.679498
H         0.522225       1.323990      -1.385120
H        -0.479876       0.471227       1.389174
H        -0.835327       2.850903       0.765324
H        -2.382460       1.991777       0.856356
H        -1.650590       2.344149      -0.715400
S        -1.895905      -0.537925      -0.252274
C        -0.886929      -2.022274      -0.134063
H         0.062527      -1.851808      -0.640812
H        -0.701202      -2.291614       0.904576
H        -1.421378      -2.833227      -0.623894
```

6_3pentene2one_HEI_4

Datum	Value
M06-2X/def2tzvpp-IEFPCM(water) Energy	-708.760358
M06-2X/def2tzvpp-IEFPCM(water) Free Energy (Quasiharmonic)	-708.635046
Number of Imaginary Frequencies	0

Frequencies (Top 3 out of 51)

```
1.      56.9514 cm-1
2.      77.4122 cm-1
3.      88.5218 cm-1
```

M06-2X/def2tzvpp-IEFPCM(water) Molecular Geometry in Cartesian Coordinates

```
C          3.320579      -0.016012      -0.515824
C          1.960482      -0.172719       0.149887
C          0.927654       0.616324      -0.300121
C         -0.436183       0.560473       0.290184
O          1.893528      -1.032009       1.094126
C         -1.135698       1.912266       0.310984
H          3.322593       0.731570      -1.308567
H          4.063060       0.262683       0.235200
H          3.634247      -0.975086      -0.934117
H          1.092590       1.305896      -1.119788
H         -0.376889       0.148815       1.299627
H         -0.570399       2.607718       0.934354
H         -2.149194       1.850253       0.708003
H         -1.187446       2.324882      -0.698245
S         -1.438857      -0.670426      -0.661816
C         -2.980755      -0.731336       0.281889
H         -3.542609      -1.599374      -0.056951
H         -2.762601      -0.848087       1.343082
H         -3.586338       0.159630       0.131456
```

6_3pentene2one_HEI_5_reopt

Datum	Value
M06-2X/def2tzvpp-IEFPCM(water) Energy	-708.75541
M06-2X/def2tzvpp-IEFPCM(water) Free Energy (Quasiharmonic)	-708.629217
Number of Imaginary Frequencies	0

Frequencies (Top 3 out of 51)

```
1.      77.9228 cm-1
2.     106.8922 cm-1
3.     107.7473 cm-1
```

M06-2X/def2tzvpp-IEFPCM(water) Molecular Geometry in Cartesian Coordinates

```
C         -2.970519      -0.562500      -0.535610
C         -1.661830      -0.012390       0.026880
C         -0.682170       0.316681      -0.882910
```

```
C          0.666450        0.878671        -0.584520
O         -1.616410        0.091850         1.300910
C          0.765020        1.795241         0.630800
H         -3.164339       -1.546200        -0.101770
H         -3.795350        0.086279        -0.231910
H         -2.973189       -0.648960        -1.621670
H         -0.878280        0.105051        -1.927250
H          1.014270        1.436461        -1.457340
H          1.754339        2.250412         0.685500
H          0.017679        2.587721         0.554590
H          0.564800        1.248851         1.547900
S          2.002981       -0.425798        -0.491290
C          1.337881       -1.475368         0.812820
H          1.447291       -1.014428         1.792340
H          0.282571       -1.653549         0.612180
H          1.884801       -2.415678         0.796050
```

6_3pentene2one_HEI_6

Datum	Value
M06-2X/def2tzvpp-IEFPCM(water) Energy	-708.754933
M06-2X/def2tzvpp-IEFPCM(water) Free Energy (Quasiharmonic)	-708.629884
Number of Imaginary Frequencies	0

Frequencies (Top 3 out of 51)

```
1.      43.3364 cm-1
2.      55.2989 cm-1
3.      94.6534 cm-1
```

M06-2X/def2tzvpp-IEFPCM(water) Molecular Geometry in Cartesian Coordinates

```
C         -2.154089       -0.641382        -1.115518
C         -1.888782        0.040607         0.221217
C         -0.676099        0.647847         0.454063
C          0.478715        0.736584        -0.478046
O         -2.858538       -0.010066         1.055341
C          1.373747        1.930640        -0.161986
H         -2.405194       -1.687275        -0.928017
H         -3.027481       -0.182113        -1.583193
H         -1.321081       -0.611261        -1.814348
H         -0.529631        1.088064         1.437141
H          0.160122        0.807555        -1.518425
```

```
H          0.794854       2.853568      -0.228310
H          2.218253       1.994532      -0.846959
H          1.761350       1.859043       0.855874
S          1.540017      -0.812469      -0.562648
C          1.948395      -1.017288       1.179087
H          2.392557      -2.002225       1.302975
H          2.657530      -0.265080       1.519226
H          1.035426      -0.956832       1.770768
```

6_3pentene2one_HEI_7

Datum	Value
M06-2X/def2tzvpp-IEFPCM(water) Energy	-708.751492
M06-2X/def2tzvpp-IEFPCM(water) Free Energy (Quasiharmonic)	-708.626048
Number of Imaginary Frequencies	0

Frequencies (Top 3 out of 51)

```
1.      53.6420 cm-1
2.      83.6133 cm-1
3.     106.6484 cm-1
```

M06-2X/def2tzvpp-IEFPCM(water) Molecular Geometry in Cartesian Coordinates

```
C         -1.773787       0.295894       1.294528
C         -1.760805      -0.340239      -0.091297
C         -0.678278      -0.171609      -0.926406
C          0.551728       0.649999      -0.720142
O         -2.806585      -1.017602      -0.386016
C          0.355246       2.128606      -0.380827
H         -2.392762       1.196743       1.286054
H         -2.234671      -0.408621       1.987739
H         -0.783538       0.555607       1.661973
H         -0.727470      -0.705119      -1.871081
H          1.123415       0.609483      -1.648880
H         -0.251360       2.596201      -1.156966
H          1.312044       2.651794      -0.327601
H         -0.158093       2.263545       0.569453
S          1.770689       0.001581       0.544387
C          1.991661      -1.666769      -0.090735
H          2.644424      -2.204700       0.593073
H          1.026774      -2.167739      -0.146828
H          2.448317      -1.646967      -1.079734
```

6_3pentene2one_TS_1_reopt

Datum	Value
M06-2X/def2tzvpp-IEFPCM(water) Energy	-708.747583
M06-2X/def2tzvpp-IEFPCM(water) Free Energy (Quasiharmonic)	-708.623927
Number of Imaginary Frequencies	1

Frequencies (Top 3 out of 51)

```
1.    -175.8704 cm-1
2.      49.9216 cm-1
3.      64.3090 cm-1
```

M06-2X/def2tzvpp-IEFPCM(water) Molecular Geometry in Cartesian Coordinates

```
C          2.940720       -0.277989       -0.647222
C          1.739465        0.093813        0.200620
C          0.685062        0.838087       -0.425292
C         -0.448051        1.195633        0.259273
O          1.726784       -0.262471        1.386248
C         -1.407687        2.217079       -0.271312
H          3.845735        0.100220       -0.170766
H          3.023288       -1.365210       -0.685250
H          2.878086        0.109863       -1.661448
H          0.772833        1.060176       -1.482238
H         -0.423058        1.068867        1.332778
H         -2.384485        2.136113        0.200132
H         -1.015820        3.218481       -0.068159
H         -1.530846        2.117060       -1.349233
S         -2.046209       -0.771776        0.201685
C         -0.897393       -1.963524       -0.516393
H          0.012066       -1.432605       -0.827800
H         -0.607400       -2.737036        0.195089
H         -1.318019       -2.446342       -1.398089
```

6_3pentene2one_TS_2_reopt

Datum	Value

Datum	Value
M06-2X/def2tzvpp-IEFPCM(water) Energy	-708.747583
M06-2X/def2tzvpp-IEFPCM(water) Free Energy (Quasiharmonic)	-708.623933
Number of Imaginary Frequencies	1

Frequencies (Top 3 out of 51)

```
1.    -175.8163 cm-1
2.      49.1787 cm-1
3.      64.3348 cm-1
```

M06-2X/def2tzvpp-IEFPCM(water) Molecular Geometry in Cartesian Coordinates

```
C          2.940469      -0.277891      -0.647278
C          1.739439       0.094285       0.200719
C          0.684971       0.838457      -0.425222
C         -0.448295       1.195686       0.259248
O          1.726711      -0.262087       1.386314
C         -1.408144       2.216906      -0.271389
H          3.846380       0.094024      -0.167660
H          3.018496      -1.365273      -0.690837
H          2.880694       0.115155      -1.659679
H          0.772841       1.060695      -1.482131
H         -0.423382       1.068866       1.332748
H         -2.384969       2.135654       0.199949
H         -1.016565       3.218403      -0.068142
H         -1.531162       2.116918      -1.349329
S         -2.045962      -0.772184       0.201648
C         -0.896705      -1.963588      -0.516303
H         -1.317249      -2.446825      -1.397808
H          0.012453      -1.432328      -0.827977
H         -0.606247      -2.736784       0.195335
```

6_3pentene2one_TS_3

Datum	Value
M06-2X/def2tzvpp-IEFPCM(water) Energy	-708.748244
M06-2X/def2tzvpp-IEFPCM(water) Free Energy (Quasiharmonic)	-708.623547
Number of Imaginary Frequencies	1

Frequencies (Top 3 out of 51)

```
1.    -207.7977 cm-1
2.      72.8449 cm-1
3.      95.2196 cm-1
```

M06-2X/def2tzvpp-IEFPCM(water) Molecular Geometry in Cartesian Coordinates

```
C          1.794647       -0.523181        1.287317
C          1.822495        0.109832       -0.093716
C          0.692278        0.869538       -0.507162
C         -0.419105        1.101241        0.287991
O          2.828854       -0.057837       -0.805724
C         -1.380763        2.201758       -0.064818
H          0.858599       -1.059769        1.453277
H          2.635177       -1.205645        1.385457
H          1.871534        0.244256        2.059830
H          0.701957        1.236573       -1.528923
H         -0.316565        0.917909        1.348505
H         -0.942103        3.166850        0.204295
H         -2.325570        2.099341        0.465648
H         -1.582872        2.210359       -1.135533
S         -1.966300       -0.719430        0.160121
C         -0.873829       -1.939710       -0.591116
H          0.039206       -1.427015       -0.919508
H         -0.592307       -2.728227        0.106683
H         -1.331418       -2.397924       -1.466852
```

6_3pentene2one_TS_4

Datum	Value
M06-2X/def2tzvpp-IEFPCM(water) Energy	-708.742738
M06-2X/def2tzvpp-IEFPCM(water) Free Energy (Quasiharmonic)	-708.619556
Number of Imaginary Frequencies	1

Frequencies (Top 3 out of 51)

```
1.    -225.0791 cm-1
2.      57.7762 cm-1
3.      74.1111 cm-1
```

M06-2X/def2tzvpp-IEFPCM(water) Molecular Geometry in Cartesian Coordinates

```
C          3.427344      -0.182356      -0.457626
C          2.049146      -0.233771       0.173533
C          1.104815       0.768282      -0.210219
C         -0.163605       0.818043       0.321146
O          1.811209      -1.141502       0.983510
C         -1.016196       2.040158       0.154358
H          3.575504       0.699143      -1.077343
H          4.182974      -0.205479       0.327801
H          3.568018      -1.074076      -1.070932
H          1.392527       1.477881      -0.976491
H         -0.349107       0.211723       1.199784
H         -0.663900       2.814217       0.843389
H         -2.062950       1.846206       0.374186
H         -0.935079       2.434196      -0.858219
S         -1.737220      -0.797116      -0.760873
C         -3.001739      -0.632365       0.532636
H         -3.457288      -1.593674       0.768034
H         -2.548719      -0.248761       1.451883
H         -3.794720       0.056548       0.240831
```

6_3pentene2one_TS_5_reopt

Datum	Value
M06-2X/def2tzvpp-IEFPCM(water) Energy	-708.742808
M06-2X/def2tzvpp-IEFPCM(water) Free Energy (Quasiharmonic)	-708.619483
Number of Imaginary Frequencies	1

Frequencies (Top 3 out of 51)

```
1.   -175.7306 cm-1
2.     42.6136 cm-1
3.     52.5006 cm-1
```

M06-2X/def2tzvpp-IEFPCM(water) Molecular Geometry in Cartesian Coordinates

```
C          2.815094      -1.032800       0.257843
C          1.714380      -0.043977      -0.085632
C          0.741131       0.229355       0.931018
```

```
C        -0.342852     1.069136     0.805541
O         1.716115     0.449465    -1.221011
C        -0.460879     2.193664    -0.182427
H         2.709256    -1.910535    -0.382034
H         3.782120    -0.582068     0.033993
H         2.794937    -1.349213     1.298057
H         0.824772    -0.352823     1.839797
H        -0.931382     1.202363     1.703899
H        -1.466917     2.608648    -0.169558
H         0.238921     2.987615     0.099910
H        -0.211757     1.875420    -1.188100
S        -2.265370    -0.291892    -0.134506
C        -1.403422    -1.877599    -0.183415
H        -1.400087    -2.302216    -1.187131
H        -0.359062    -1.721963     0.118244
H        -1.844524    -2.607344     0.495551
```

6_3pentene2one_TS_6

Datum	Value
M06-2X/def2tzvpp-IEFPCM(water) Energy	-708.748244
M06-2X/def2tzvpp-IEFPCM(water) Free Energy (Quasiharmonic)	-708.623547
Number of Imaginary Frequencies	1

Frequencies (Top 3 out of 51)

```
 1.   -207.7085 cm-1
 2.     72.8461 cm-1
 3.     95.2144 cm-1
```

M06-2X/def2tzvpp-IEFPCM(water) Molecular Geometry in Cartesian Coordinates

```
C        -1.794615    -0.523349    -1.287267
C        -1.822507     0.109812     0.093680
C        -0.692330     0.869641     0.507074
C         0.419013     1.101373    -0.288027
O        -2.828846    -0.057840     0.805704
C         1.380711     2.201846     0.064788
H        -2.635109    -1.205867    -1.385317
H        -1.871528     0.243986    -2.059880
H        -0.858535    -1.059907    -1.453135
H        -0.702084     1.236775     1.528800
H         0.316632     0.917898    -1.348530
```

```
H          0.942130     3.166949    -0.204413
H          2.325542     2.099330    -0.465608
H          1.582747     2.210503     1.135516
S          1.966398    -0.719501    -0.160087
C          0.873782    -1.939655     0.591165
H          0.592376    -2.728298    -0.106535
H          1.331217    -2.397724     1.467060
H         -0.039308    -1.426912     0.919327
```

6_3pentene2one_TS_7

Datum	Value
M06-2X/def2tzvpp-IEFPCM(water) Energy	-708.74125
M06-2X/def2tzvpp-IEFPCM(water) Free Energy (Quasiharmonic)	-708.616396
Number of Imaginary Frequencies	1

Frequencies (Top 3 out of 51)

```
1.    -198.4299 cm-1
2.      54.1396 cm-1
3.      62.6093 cm-1
```

M06-2X/def2tzvpp-IEFPCM(water) Molecular Geometry in Cartesian Coordinates

```
C         -1.609273     0.017212     1.374915
C         -1.771389    -0.223234    -0.112933
C         -0.781805     0.259886    -1.025131
C          0.308655     1.075839    -0.770860
O         -2.753433    -0.879712    -0.496280
C          0.397970     2.165014     0.267666
H         -1.879039     1.043024     1.629653
H         -2.268487    -0.660083     1.912898
H         -0.573992    -0.142876     1.681361
H         -0.871224    -0.152643    -2.024398
H          0.880633     1.314491    -1.658947
H         -0.315044     2.956753     0.017234
H          1.396859     2.597967     0.266871
H          0.184601     1.824200     1.274476
S          2.170981    -0.244587     0.100783
C          1.340049    -1.844176     0.097398
H          1.251265    -2.254253     1.103220
H          0.326779    -1.706358    -0.301874
H          1.854164    -2.572389    -0.529109
```

6_cis_3pentene2one_1

Datum	Value
M06-2X/def2tzvpp-IEFPCM(water) Energy	-270.537274
M06-2X/def2tzvpp-IEFPCM(water) Free Energy (Quasiharmonic)	-270.447003
Number of Imaginary Frequencies	0

Frequencies (Top 3 out of 36)

```
1.      47.6694 cm-1
2.      68.6799 cm-1
3.     137.3835 cm-1
```

M06-2X/def2tzvpp-IEFPCM(water) Molecular Geometry in Cartesian Coordinates

```
C          -2.297330       -0.270427        0.190285
C          -0.877956        0.163455       -0.064601
C           0.115789       -0.931828       -0.154908
C           1.439537       -0.791365       -0.051023
O          -0.587188        1.336696       -0.196230
C           2.218169        0.460970        0.177659
H          -2.950021        0.593647        0.270490
H          -2.630285       -0.913982       -0.626019
H          -2.345033       -0.865500        1.103410
H          -0.294953       -1.926410       -0.281055
H           2.030225       -1.698747       -0.131480
H           3.083167        0.251393        0.805612
H           2.600410        0.834666       -0.776161
H           1.614732        1.246532        0.620576
```

6_cis_3pentene2one_2

Datum	Value
M06-2X/def2tzvpp-IEFPCM(water) Energy	-270.535588
M06-2X/def2tzvpp-IEFPCM(water) Free Energy (Quasiharmonic)	-270.444392
Number of Imaginary Frequencies	0

Frequencies (Top 3 out of 36)

```
1.      46.4002 cm-1
2.      75.9100 cm-1
3.     257.0370 cm-1
```

M06-2X/def2tzvpp-IEFPCM(water) Molecular Geometry in Cartesian Coordinates

```
C          -0.828335        1.409395        0.068758
C          -1.042312       -0.078658       -0.002052
C           0.094371       -1.025869        0.050808
C           1.408043       -0.785000        0.034622
O          -2.170030       -0.530659       -0.084383
C           2.161182        0.503480       -0.072233
H          -1.783606        1.892983        0.252528
H          -0.115452        1.672463        0.848043
H          -0.429163        1.766307       -0.881986
H          -0.235514       -2.056093        0.118462
H           2.039661       -1.664170        0.115096
H           2.354033        0.908086        0.924809
H           3.129838        0.322547       -0.534010
H           1.642743        1.263062       -0.647298
```

Created using ESIgen v0.0.5

ESIgen is scientific software, funded by public research grants and published as:

```
J Rodriguez-Guerra, P Gomez-Orellana, JD Marechal.
J. Chem. Inf. Model., 2018, 58 (3), pp 561564.
DOI: 10.1021/acs.jcim.7b00714.
```

If you make use of ESIgen in scientific publications, please cite us in the main text! References only mentioned in SI documents are not indexed by citation engines.

1_methylacrolein_1_am1_HEI

Datum	Value
AM1 Energy	-0.111465
AM1 Free Energy (Quasiharmonic)	-0.012505
Number of Imaginary Frequencies	0

Frequencies (Top 3 out of 42)

```
1.      33.8223 cm-1
2.      67.6256 cm-1
3.      94.2264 cm-1
```

AM1 Molecular Geometry in Cartesian Coordinates

```
C         -1.793440      -0.611816      -0.145821
C         -0.983371       0.504765       0.093542
C          0.121048       0.454914       1.013883
O         -1.694333      -1.761703       0.364529
H          0.287096       1.413363       1.563507
H          0.033409      -0.400702       1.723716
C          1.595488      -0.986981      -0.952579
H          2.128265      -1.915301      -0.651493
H          0.506357      -1.208773      -1.070584
H          2.008764      -0.631460      -1.921429
S          1.812877       0.255989       0.267323
H         -2.632465      -0.444109      -0.874513
C         -1.245816       1.772468      -0.605024
H         -1.496364       2.592931       0.118909
```

```
  H          -2.101879       1.680876       -1.320355
  H          -0.348015       2.110883       -1.185167
```

1_methylacrolein_2_am1_HEI

Datum	Value
AM1 Energy	-0.112426
AM1 Free Energy (Quasiharmonic)	-0.013337
Number of Imaginary Frequencies	0

Frequencies (Top 3 out of 42)

```
    1.      38.7715 cm-1
    2.      75.5113 cm-1
    3.      95.8763 cm-1
```

AM1 Molecular Geometry in Cartesian Coordinates

```
  C           1.633504      -0.544295        0.623332
  C           0.811971       0.491707        0.151696
  C          -0.386869       0.858647        0.849006
  O           2.709521      -0.959607        0.115737
  H          -0.378983       0.574227        1.927608
  H          -0.648711       1.937788        0.737000
  C          -1.509996      -1.183099       -0.860055
  H          -0.394638      -1.208210       -0.950576
  H          -1.874607      -2.168460       -0.496458
  H          -1.954026      -0.979597       -1.858814
  S          -1.984894       0.074587        0.265083
  H           1.279892      -1.043979        1.565088
  C           1.164222       1.225276       -1.074073
  H           2.147352       0.871695       -1.473594
  H           0.389698       1.082346       -1.873004
  H           1.239158       2.328248       -0.883913
```

1_methylacrolein_3_am1_HEI

Datum	Value
AM1 Energy	-0.111465

Datum	Value
AM1 Free Energy (Quasiharmonic)	-0.012504
Number of Imaginary Frequencies	0

Frequencies (Top 3 out of 42)

```
1.      33.8382 cm-1
2.      67.7514 cm-1
3.      94.2407 cm-1
```

AM1 Molecular Geometry in Cartesian Coordinates

```
C        1.793396     -0.611883     -0.145788
C        0.983385      0.504748      0.093531
C       -0.121053      0.454974      1.013856
O        1.694173     -1.761769      0.364548
H       -0.033462     -0.400621      1.723720
H       -0.287055      1.413446      1.563456
C       -1.595476     -0.987031     -0.952499
H       -2.008619     -0.631575     -1.921432
H       -0.506358     -1.208944     -1.070378
H       -2.128395     -1.915253     -0.651361
S       -1.812858      0.256083      0.267264
H        2.632481     -0.444237     -0.874425
C        1.245927      1.772431     -0.605026
H        2.102207      1.680845     -1.320097
H        1.496186      2.592965      0.118930
H        0.348280      2.110757     -1.185469
```

1_methylacrolein_4_reopt_am1_HEI

Datum	Value
AM1 Energy	-0.112426
AM1 Free Energy (Quasiharmonic)	-0.013337
Number of Imaginary Frequencies	0

Frequencies (Top 3 out of 42)

```
1.      38.7637 cm-1
2.      75.5166 cm-1
```

```
3.      95.8886 cm-1
```

AM1 Molecular Geometry in Cartesian Coordinates

```
C        -1.633539      -0.544290       0.623309
C        -0.811977       0.491699       0.151698
C         0.386857       0.858617       0.849037
O        -2.709552      -0.959581       0.115691
H         0.648679       1.937771       0.737102
H         0.378975       0.574131       1.927620
C         1.510014      -1.183211      -0.859929
H         0.394662      -1.208263      -0.950550
H         1.954151      -0.979923      -1.858685
H         1.874526      -2.168529      -0.496116
S         1.984896       0.074650       0.265014
H        -1.279955      -1.043987       1.565069
C        -1.164177       1.225296      -1.074067
H        -2.147296       0.871737      -1.473634
H        -1.239106       2.328265      -0.883885
H        -0.389621       1.082375      -1.872968
```

1_methylacrolein_5_am1_HEI

Datum	Value
AM1 Energy	-0.106715
AM1 Free Energy (Quasiharmonic)	-0.008644
Number of Imaginary Frequencies	0

Frequencies (Top 3 out of 42)

```
1.      36.1511 cm-1
2.      57.3415 cm-1
3.      60.3798 cm-1
```

AM1 Molecular Geometry in Cartesian Coordinates

```
C        -2.108547      -0.522424      -0.182084
C        -1.122298       0.401441       0.191233
C         0.138257      -0.026896       0.732704
O        -2.077187      -1.776816      -0.063038
H         0.509190       0.611176       1.570723
```

```
H          0.130668       -1.098963        1.039685
C          2.886076       -0.429610        0.255454
H          3.131561        0.209078        1.133732
H          2.768424       -1.477160        0.613358
H          3.730179       -0.387833       -0.467888
S          1.444234        0.130311       -0.563550
H         -3.042281       -0.070098       -0.614742
C         -1.364978        1.844390        0.041443
H         -1.446822        2.350979        1.040633
H         -2.314410        2.046937       -0.515879
H         -0.527813        2.344029       -0.511022
```

1_methylacrolein_6_reopt_am1_HEI

Datum	Value
AM1 Energy	-0.112426
AM1 Free Energy (Quasiharmonic)	-0.013337
Number of Imaginary Frequencies	0

Frequencies (Top 3 out of 42)

```
1.      38.7617 cm-1
2.      75.5160 cm-1
3.      95.8874 cm-1
```

AM1 Molecular Geometry in Cartesian Coordinates

```
C         -1.633525       -0.544301        0.623307
C         -0.811980        0.491703        0.151701
C          0.386860        0.858623        0.849032
O         -2.709538       -0.959598        0.115692
H          0.648678        1.937778        0.737095
H          0.378984        0.574139        1.927616
C          1.510014       -1.183215       -0.859927
H          0.394662       -1.208274       -0.950545
H          1.954146       -0.979933       -1.858686
H          1.874533       -2.168529       -0.496107
S          1.984894        0.074655        0.265007
H         -1.279926       -1.044007        1.565057
C         -1.164199        1.225309       -1.074054
H         -2.147334        0.871766       -1.473596
H         -1.239104        2.328279       -0.883869
H         -0.389665        1.082374       -1.872974
```

1_methylacrolein_7_am1_HEI

Datum	Value
AM1 Energy	-0.10788
AM1 Free Energy (Quasiharmonic)	-0.009611
Number of Imaginary Frequencies	0

Frequencies (Top 3 out of 42)

```
1.      45.0407 cm-1
2.      59.6497 cm-1
3.      74.3969 cm-1
```

AM1 Molecular Geometry in Cartesian Coordinates

```
C          1.829284       -0.856094        0.163841
C          0.998038        0.270104        0.280274
C         -0.331044        0.151427        0.805721
O          3.019094       -0.889394       -0.248999
H         -0.472311       -0.714985        1.494552
H         -0.700206        1.082648        1.297176
C         -3.078409       -0.198856        0.193459
H         -3.138525       -1.019845        0.942966
H         -3.303503        0.759214        0.713856
H         -3.847382       -0.371611       -0.591669
S         -1.511245       -0.153322       -0.583569
H          1.370178       -1.825563        0.496312
C          1.469441        1.596042       -0.148739
H          2.515453        1.530696       -0.539746
H          0.818890        2.024439       -0.954499
H          1.460713        2.327571        0.702812
```

1_methylacrolein_8_am1_HEI_reopt

Datum	Value
AM1 Energy	-0.10788
AM1 Free Energy (Quasiharmonic)	-0.009612
Number of Imaginary Frequencies	0

Frequencies (Top 3 out of 42)

```
1.      45.0342 cm-1
2.      59.6120 cm-1
3.      74.3758 cm-1
```

AM1 Molecular Geometry in Cartesian Coordinates

```
C          -1.829292       -0.856074        0.163875
C          -0.998062        0.270138        0.280284
C           0.330986        0.151494        0.805778
O          -3.019073       -0.889432       -0.249056
H           0.700140        1.082745        1.297178
H           0.472238       -0.714884        1.494651
C           3.078450       -0.198862        0.193436
H           3.138329       -1.019301        0.943561
H           3.847273       -0.372493       -0.591645
H           3.303979        0.759517        0.713075
S           1.511228       -0.153361       -0.583492
H          -1.370214       -1.825507        0.496495
C          -1.469402        1.596049       -0.148878
H          -0.819130        2.024136       -0.955023
H          -2.515587        1.530763       -0.539428
H          -1.460175        2.327792        0.702479
```

1_methylacrolein_conf2_min_am1

Datum	Value
AM1 Energy	-0.038056
AM1 Free Energy (Quasiharmonic)	0.026225
Number of Imaginary Frequencies	0

Frequencies (Top 3 out of 27)

```
1.      19.0539 cm-1
2.      82.7581 cm-1
3.     269.6063 cm-1
```

AM1 Molecular Geometry in Cartesian Coordinates

```
C         -1.554806       -0.880095        0.000009
H         -1.475342       -1.975947        0.000009
H         -2.579759       -0.485298        0.000020
C         -0.481780       -0.080733       -0.000000
C          0.879445       -0.656113       -0.000015
H          0.923291       -1.770162       -0.000057
O          1.907618        0.022832        0.000016
C         -0.572827        1.397752       -0.000005
H         -0.057346        1.809858        0.903823
H         -0.057607        1.809833       -0.903994
H         -1.634377        1.744202        0.000139
```

1_methylacrolein_min_am1

Datum	Value
AM1 Energy	-0.038885
AM1 Free Energy (Quasiharmonic)	0.025987
Number of Imaginary Frequencies	0

Frequencies (Top 3 out of 27)

```
1.      68.2365 cm-1
2.      81.5885 cm-1
3.     287.2702 cm-1
```

AM1 Molecular Geometry in Cartesian Coordinates

```
C          0.441184        1.450224       -0.000006
H         -0.483749        2.045955       -0.000007
H          1.378878        2.021578       -0.000009
C          0.412792        0.111807       -0.000001
C         -0.862632       -0.630288        0.000004
H         -0.760873       -1.739832        0.000009
O         -1.971137       -0.090016        0.000001
C          1.635846       -0.724023        0.000002
H          1.656485       -1.379658        0.906251
H          1.656484       -1.379667       -0.906241
H          2.558732       -0.094567       -0.000002
```

2_crotonaldehyde-1-0_am1_HEI

Datum	Value
AM1 Energy	-0.104412
AM1 Free Energy (Quasiharmonic)	-0.005406
Number of Imaginary Frequencies	0

Frequencies (Top 3 out of 42)

```
1.      41.5973 cm-1
2.      77.3142 cm-1
3.     118.8495 cm-1
```

AM1 Molecular Geometry in Cartesian Coordinates

```
C         -2.110854       -0.034781        0.362736
C         -0.899730        0.570354        0.693749
C          0.155098        0.807865       -0.246418
O         -2.499732       -0.447170       -0.765553
H         -0.729175        0.863941        1.734502
H         -0.189063        0.683859       -1.303101
C          0.797283       -1.963731        0.222020
H         -0.279171       -1.777531        0.463048
H          0.863331       -2.650180       -0.650215
H          1.295081       -2.439986        1.094660
S          1.587399       -0.442515       -0.143122
H         -2.825241       -0.148501        1.223807
C          0.915421        2.092620       -0.054037
H          1.226703        2.214569        1.011291
H          1.829633        2.119124       -0.697075
H          0.264054        2.958343       -0.330841
```

2_crotonaldehyde-2-0_am1_HEI

Datum	Value
AM1 Energy	-0.104518
AM1 Free Energy (Quasiharmonic)	-0.005567
Number of Imaginary Frequencies	0

Frequencies (Top 3 out of 42)

```
1.      41.1680 cm-1
2.      82.6451 cm-1
3.     136.7621 cm-1
```

AM1 Molecular Geometry in Cartesian Coordinates

```
C          1.926143       -0.072589       -0.234608
C          0.928001        0.110254        0.726353
C         -0.260781        0.878050        0.546318
O          2.981658       -0.749702       -0.096753
H          1.048676       -0.397996        1.690541
H         -0.657453        1.277017        1.515253
C         -1.201325       -1.751831       -0.323272
H         -0.089405       -1.773438       -0.191217
H         -1.665384       -2.504845        0.350374
H         -1.452858       -2.006828       -1.375894
S         -1.812280       -0.156467        0.063582
H          1.772270        0.444521       -1.219129
C         -0.242674        1.958335       -0.498991
H         -1.217154        2.505357       -0.517093
H         -0.063606        1.532539       -1.515268
H          0.571944        2.691438       -0.275656
```

2_crotonaldehyde-3-0_am1_HEI

Datum	Value
AM1 Energy	-0.106264
AM1 Free Energy (Quasiharmonic)	-0.007377
Number of Imaginary Frequencies	0

Frequencies (Top 3 out of 42)

```
1.      43.4230 cm-1
2.      91.2258 cm-1
3.     140.2700 cm-1
```

AM1 Molecular Geometry in Cartesian Coordinates

```
C         -2.006287       -0.014112       -0.366520
C         -0.923646        0.549786        0.314870
```

```
C          0.314903       0.864181       -0.321204
O         -3.127496      -0.320399        0.123108
H         -1.021034       0.744014        1.387716
H          0.236134       0.947553       -1.433656
C          0.803451      -1.943132        0.321562
H          1.256830      -2.326601        1.261515
H         -0.273100      -1.694605        0.505345
H          0.871405      -2.732189       -0.458924
S          1.659339      -0.508704       -0.204424
H         -1.852865      -0.198926       -1.463973
C          1.061763       2.033192        0.260606
H          2.090013       2.107525       -0.171272
H          1.148612       1.934678        1.369215
H          0.513446       2.981505        0.034066
```

2_crotonaldehyde-4-0_am1_HEI

Datum	Value
AM1 Energy	-0.104518
AM1 Free Energy (Quasiharmonic)	-0.005566
Number of Imaginary Frequencies	0

Frequencies (Top 3 out of 42)

```
1.      41.2155 cm-1
2.      82.6342 cm-1
3.     136.6572 cm-1
```

AM1 Molecular Geometry in Cartesian Coordinates

```
C         -1.926156      -0.072531        0.234593
C         -0.927984       0.110270       -0.726338
C          0.260811       0.878036       -0.546310
O         -2.981702      -0.749595        0.096707
H         -1.048676      -0.397963       -1.690534
H          0.657452       1.277046       -1.515243
C          1.201199      -1.751810        0.323374
H          1.452509      -2.006629        1.376091
H          0.089311      -1.773366        0.191127
H          1.665356      -2.504959       -0.350049
S          1.812292      -0.156527       -0.063671
H         -1.772302       0.444576        1.219118
C          0.242803       1.958269        0.499064
H          1.217409       2.505060        0.517355
```

```
H          0.063458        1.532453        1.515282
H         -0.571599        2.691581        0.275630
```

2_crotonaldehyde-5-0_am1_HEI

Datum	Value
AM1 Energy	-0.104412
AM1 Free Energy (Quasiharmonic)	-0.005408
Number of Imaginary Frequencies	0

Frequencies (Top 3 out of 42)

```
1.      41.5496 cm-1
2.      77.2744 cm-1
3.     118.7466 cm-1
```

AM1 Molecular Geometry in Cartesian Coordinates

```
C         -2.110888       -0.035291        0.362813
C         -0.899771        0.569806        0.693862
C          0.154952        0.807716       -0.246356
O         -2.499890       -0.447378       -0.765547
H         -0.729146        0.863181        1.734660
H         -0.189276        0.683609       -1.303005
C          0.797973       -1.963604        0.221736
H          1.295994       -2.439970        1.094184
H         -0.278509       -1.777823        0.462965
H          0.864166       -2.649752       -0.650719
S          1.587615       -0.442059       -0.143042
H         -2.825158       -0.149309        1.223941
C          0.914662        2.092864       -0.054080
H          1.226004        2.214999        1.011203
H          1.828789        2.119831       -0.697213
H          0.262842        2.958259       -0.330827
```

2_crotonaldehyde-6-1_am1_HEI

Datum	Value
AM1 Energy	-0.099891

Datum	Value
AM1 Free Energy (Quasiharmonic)	-0.001275
Number of Imaginary Frequencies	0

Frequencies (Top 3 out of 42)

```
1.     46.2292 cm-1
2.     69.6864 cm-1
3.     78.8464 cm-1
```

AM1 Molecular Geometry in Cartesian Coordinates

```
C     2.384314    -0.256252    -0.263008
C     1.228589     0.434765    -0.626075
C     0.070064     0.532338     0.216999
O     2.648944    -0.819781     0.834700
H     1.180093     0.900757    -1.614771
H     0.260242     0.147084     1.249963
C    -2.552347    -0.680974     0.494459
H    -2.259029    -0.754648     1.565483
H    -3.181465     0.229342     0.370262
H    -3.159738    -1.572871     0.223077
S    -1.145518    -0.649701    -0.546676
H     3.183000    -0.274959    -1.054593
C    -0.576123     1.892194     0.261447
H    -0.791989     2.263328    -0.769042
H    -1.533975     1.864000     0.837038
H     0.112615     2.619002     0.758869
```

2_crotonaldehyde-7-0_am1_HEI

Datum	Value
AM1 Energy	-0.10172
AM1 Free Energy (Quasiharmonic)	-0.00331
Number of Imaginary Frequencies	0

Frequencies (Top 3 out of 42)

```
1.     54.3570 cm-1
2.     69.9752 cm-1
```

```
   3.      78.4510 cm-1
```

AM1 Molecular Geometry in Cartesian Coordinates

```
C          2.213577       -0.340538        0.363146
C          1.217529        0.417991       -0.259696
C         -0.080504        0.610090        0.311347
O          3.382713       -0.543257       -0.063661
H          1.421165        0.853910       -1.242387
H         -0.118724        0.443283        1.417537
C         -2.706475       -0.674248        0.323236
H         -2.649078       -0.521112        1.424163
H         -3.298146        0.163226       -0.110673
H         -3.239019       -1.629479        0.118418
S         -1.110794       -0.781080       -0.387691
H          1.938771       -0.791794        1.354348
C         -0.745086        1.913348       -0.045056
H         -1.795234        1.945280        0.336494
H         -0.764575        2.056455       -1.152038
H         -0.178404        2.763701        0.408627
```

2_crotonaldehyde-8-1_am1_HEI

Datum	Value
AM1 Energy	-0.102333
AM1 Free Energy (Quasiharmonic)	-0.003211
Number of Imaginary Frequencies	0

Frequencies (Top 3 out of 42)

```
   1.      38.5170 cm-1
   2.      56.0144 cm-1
   3.     121.8176 cm-1
```

AM1 Molecular Geometry in Cartesian Coordinates

```
C         -1.902353       -0.691288       -0.377288
C         -0.837719       -0.105813       -1.059677
C          0.077690        0.876446       -0.561981
O         -2.316040       -0.488085        0.797599
H         -0.647160       -0.459437       -2.081221
```

```
H         0.464946      1.546767     -1.374582
C         1.421674     -1.478453      0.481686
H         1.608520     -1.562954      1.574502
H         0.350272     -1.722668      0.271040
H         2.077639     -2.199342     -0.053314
S         1.771751      0.147668     -0.069685
H        -2.469134     -1.449024     -0.988159
C        -0.375157      1.684296      0.620442
H        -0.694337      1.009890      1.453149
H         0.440362      2.356568      0.981509
H        -1.255607      2.311055      0.332147
```

2_crotonaldehyde_min_am1

Datum	Value
AM1 Energy	-0.043079
AM1 Free Energy (Quasiharmonic)	0.021628
Number of Imaginary Frequencies	0

Frequencies (Top 3 out of 27)

```
  1.      77.5650 cm-1
  2.     119.5921 cm-1
  3.     208.0285 cm-1
```

AM1 Molecular Geometry in Cartesian Coordinates

```
C         0.841994     -0.320672      0.000012
H         0.506684     -1.374814      0.000113
C        -0.059071      0.671425     -0.000061
H         0.245643      1.729967     -0.000162
C        -1.504586      0.430808     -0.000007
H        -2.133685      1.350639     -0.000057
O        -2.021177     -0.690765      0.000087
C         2.300687     -0.111120     -0.000025
H         2.753115     -0.587832      0.906316
H         2.753065     -0.587797     -0.906410
H         2.570449      0.973312     -0.000011
```

2_crotonaldehyde_min_conf2_am1

Datum	Value
AM1 Energy	-0.04312
AM1 Free Energy (Quasiharmonic)	0.021706
Number of Imaginary Frequencies	0

Frequencies (Top 3 out of 27)

```
1.      71.1397 cm-1
2.     109.8476 cm-1
3.     227.3218 cm-1
```

AM1 Molecular Geometry in Cartesian Coordinates

```
C         1.055342       0.415507       0.000001
H         1.039740       1.520263      -0.000004
C        -0.082293      -0.289596       0.000005
H        -0.087453      -1.392392       0.000011
C        -1.401413       0.353030       0.000002
H        -1.400537       1.467741       0.000006
O        -2.457200      -0.284499      -0.000004
C         2.398485      -0.192880      -0.000002
H         2.966292       0.136681      -0.906666
H         2.966366       0.136827       0.906563
H         2.352462      -1.309496       0.000089
```

3_4methyl2pentenal_10_reopt_am1_HEI

Datum	Value
AM1 Energy	-0.114671
AM1 Free Energy (Quasiharmonic)	0.038561
Number of Imaginary Frequencies	0

Frequencies (Top 3 out of 60)

```
1.      39.4706 cm-1
2.      60.0635 cm-1
3.      92.5093 cm-1
```

AM1 Molecular Geometry in Cartesian Coordinates

```
C       -2.101168      -1.024095      -0.369305
C       -0.882755      -0.877412      -1.024235
C        0.118707       0.137218      -0.857796
O       -2.643879      -0.301555       0.512622
H        0.529392       0.471594      -1.851276
C        1.287427      -1.906146       0.831560
H        1.457614      -1.616654       1.891980
H        1.893994      -2.808112       0.599081
H        0.202410      -2.135209       0.684551
S        1.761001      -0.603969      -0.242288
H       -2.686107      -1.925099      -0.709412
H       -0.638546      -1.639872      -1.777570
C       -0.257316       1.359739      -0.035637
H       -1.343107       1.577578      -0.264420
C       -0.149697       1.138623       1.458107
H        0.895304       0.864024       1.742172
H       -0.440937       2.067335       2.006390
H       -0.841540       0.312017       1.757267
C        0.578909       2.557572      -0.445104
H        1.664080       2.341656      -0.291276
H        0.414870       2.802294      -1.522169
H        0.302933       3.451401       0.164771
```

3_4methyl2pentenal_11_am1_HEI

Datum	Value
AM1 Energy	-0.115303
AM1 Free Energy (Quasiharmonic)	0.037662
Number of Imaginary Frequencies	0

Frequencies (Top 3 out of 60)

```
1.     25.8360 cm-1
2.     49.5258 cm-1
3.     94.9828 cm-1
```

AM1 Molecular Geometry in Cartesian Coordinates

```
C       -0.342243       1.908955       0.255753
C       -0.218590       0.992066      -0.791304
```

```
C        0.215297    -0.365345    -0.722656
O       -0.708075     3.112164     0.143987
H        0.300438    -0.788735    -1.760426
C       -2.564056    -0.694198     0.109031
H       -2.993584    -0.825135     1.126167
H       -2.363952     0.392563    -0.072461
H       -3.296712    -1.062519    -0.641913
S       -1.070527    -1.601557    -0.008853
H       -0.114613     1.538107     1.288368
H       -0.544638     1.337102    -1.782769
C        1.478962    -0.765323     0.026096
H        1.537319    -1.894360    -0.011209
C        2.689134    -0.208936    -0.702606
H        2.603388     0.901558    -0.787118
H        3.626441    -0.460113    -0.150160
H        2.757010    -0.637226    -1.731260
C        1.521212    -0.363557     1.482876
H        1.746635     0.724764     1.589752
H        0.537113    -0.582956     1.965004
H        2.319879    -0.937414     2.014645
```

3_4methyl2pentenal_12_reopt_am1_HEI

Datum	Value
AM1 Energy	-0.118533
AM1 Free Energy (Quasiharmonic)	0.034329
Number of Imaginary Frequencies	0

Frequencies (Top 3 out of 60)

```
1.      34.1181 cm-1
2.      55.0023 cm-1
3.      66.4440 cm-1
```

AM1 Molecular Geometry in Cartesian Coordinates

```
C       -2.208969    -1.219727    -0.310557
C       -0.908109    -0.882192    -0.680486
C        0.066885    -0.365310     0.235645
O       -2.740749    -1.157063     0.833091
H       -0.275737    -0.467345     1.297135
C       -1.298155     2.125583    -0.216629
H       -1.272628     2.804109    -1.097051
H       -1.988824     1.270915    -0.425485
```

```
H        -1.672600     2.690975     0.664599
S         0.317119     1.521461     0.098047
H        -2.846300    -1.607707    -1.151732
H        -0.618751    -0.998046    -1.729447
C         1.472373    -0.931654     0.084194
H         1.369388    -2.053102     0.169523
C         2.097916    -0.620883    -1.258656
H         2.118870     0.484667    -1.425372
H         3.142281    -1.015820    -1.303380
H         1.504474    -1.090482    -2.079156
C         2.374630    -0.456584     1.206650
H         2.464358     0.657811     1.185229
H         1.953802    -0.757282     2.196086
H         3.394320    -0.900970     1.104614
```

3_4methyl2pentenal_13_am1_HEI

Datum	Value
AM1 Energy	-0.117561
AM1 Free Energy (Quasiharmonic)	0.035664
Number of Imaginary Frequencies	0

Frequencies (Top 3 out of 60)

```
1.      36.2756 cm-1
2.      69.9440 cm-1
3.      79.1900 cm-1
```

AM1 Molecular Geometry in Cartesian Coordinates

```
C        -0.322323     2.199386     0.155432
C         0.033308     1.147951     0.998521
C        -0.055549    -0.257954     0.737341
O        -0.827008     2.168930    -1.001000
H        -0.144020    -0.860310     1.679492
C         2.508646     0.344145    -0.539939
H         2.674513     0.154509    -1.623021
H         1.932797     1.295316    -0.417475
H         3.494769     0.442928    -0.035563
S         1.622881    -0.989037     0.171503
H        -0.127894     3.217462     0.595530
H         0.486410     1.419118     1.961458
C        -1.108766    -0.708184    -0.261728
H        -0.906486    -0.178270    -1.236937
```

```
C          -1.074639       -2.206698       -0.483497
H          -1.338951       -2.750765        0.455616
H          -1.808204       -2.496387       -1.274371
H          -0.051424       -2.525646       -0.803987
C          -2.488791       -0.297824        0.218799
H          -2.704195       -0.730989        1.224958
H          -2.539114        0.816919        0.283710
H          -3.269531       -0.655664       -0.495026
```

3_4methyl2pentenal_14_am1_HEI

Datum	Value
AM1 Energy	-0.115214
AM1 Free Energy (Quasiharmonic)	0.037266
Number of Imaginary Frequencies	0

Frequencies (Top 3 out of 60)

```
1.      46.7949 cm-1
2.      50.0865 cm-1
3.      65.0255 cm-1
```

AM1 Molecular Geometry in Cartesian Coordinates

```
C           2.491346       -0.521970       -0.403823
C           1.235155       -0.010217       -0.732031
C           0.130862        0.005753        0.185005
O           2.895190       -0.953679        0.710826
H           0.440291       -0.291182        1.219860
C          -2.267612       -1.555089        0.722779
H          -3.073773       -0.806001        0.553027
H          -2.697551       -2.573426        0.594847
H          -1.905341       -1.443475        1.769048
S          -0.955716       -1.375772       -0.422739
H           3.233076       -0.510172       -1.248926
H           1.066406        0.354904       -1.749475
C          -0.693726        1.286044        0.254294
H          -1.652036        1.052690        0.803759
C           0.060702        2.335070        1.047990
H           1.063016        2.510469        0.587493
H          -0.505608        3.297510        1.063220
H           0.212627        1.993262        2.099755
C          -1.057400        1.824093       -1.112480
H          -0.153174        2.231523       -1.625147
```

```
H          -1.481221        1.001332       -1.739216
H          -1.812743        2.642245       -1.021441
```

3_4methyl2pentenal_1_am1_HEI

Datum	Value
AM1 Energy	-0.118533
AM1 Free Energy (Quasiharmonic)	0.034329
Number of Imaginary Frequencies	0

Frequencies (Top 3 out of 60)

```
1.      34.1720 cm-1
2.      54.8981 cm-1
3.      66.4173 cm-1
```

AM1 Molecular Geometry in Cartesian Coordinates

```
C          -2.209214       -1.219307       -0.310678
C          -0.908376       -0.881686       -0.680608
C           0.066770       -0.365320        0.235647
O          -2.740863       -1.157143        0.833056
H          -0.275860       -0.467600        1.297106
C          -1.297615        2.125835       -0.216578
H          -1.672521        2.690423        0.664962
H          -1.271631        2.805152       -1.096366
H          -1.988135        1.271323       -0.426528
S           0.317500        1.521382        0.098416
H          -2.846664       -1.606850       -1.151963
H          -0.619153       -0.997096       -1.729652
C           1.472126       -0.931934        0.084004
H           1.368961       -2.053371        0.169062
C           2.097632       -0.620855       -1.258798
H           2.118490        0.484727       -1.425250
H           3.142022       -1.015701       -1.303624
H           1.504222       -1.090308       -2.079393
C           2.374508       -0.457235        1.206512
H           2.464232        0.657160        1.185397
H           1.953775       -0.758214        2.195897
H           3.394173       -0.901606        1.104239
```

3_4methyl2pentenal_1_am1

Datum	Value
AM1 Energy	-0.0595
AM1 Free Energy (Quasiharmonic)	0.058979
Number of Imaginary Frequencies	0

Frequencies (Top 3 out of 45)

```
1.      40.5471 cm-1
2.      94.5033 cm-1
3.     150.0735 cm-1
```

AM1 Molecular Geometry in Cartesian Coordinates

```
C          2.308364       -0.406565       -0.018314
C          0.964685        0.183649       -0.054599
C         -0.123368       -0.547587       -0.320681
C         -1.517809       -0.032936       -0.398157
C         -2.445553       -0.925634        0.407001
O          3.322862        0.251712        0.224257
C         -1.660465        1.407533        0.045966
H          2.366977       -1.500475       -0.224771
H          0.915765        1.265646        0.152765
H         -0.035048       -1.632273       -0.518398
H         -1.825424       -0.096282       -1.485386
H         -3.501459       -0.582482        0.289033
H         -2.373883       -1.983500        0.057633
H         -2.180627       -0.889476        1.490807
H         -2.731108        1.720050       -0.014524
H         -1.314876        1.531919        1.100413
H         -1.058337        2.082406       -0.608919
```

3_4methyl2pentenal_2_am1

Datum	Value
AM1 Energy	-0.060516
AM1 Free Energy (Quasiharmonic)	0.057565
Number of Imaginary Frequencies	0

Frequencies (Top 3 out of 45)

```
1.      36.4524 cm-1
2.      96.3614 cm-1
3.     139.2734 cm-1
```

AM1 Molecular Geometry in Cartesian Coordinates

```
C         -1.378596       -1.949080       -0.000000
C         -0.182447       -1.098397       -0.000000
C         -0.257362        0.237222        0.000000
C          0.934177        1.132730        0.000000
C          0.934177        1.997666        1.246657
O         -1.324645       -3.181269       -0.000000
C          0.934177        1.997666       -1.246657
H         -2.356831       -1.414670       -0.000000
H          0.783122       -1.631343       -0.000000
H         -1.230308        0.759299        0.000000
H          1.874682        0.509738        0.000000
H          1.833848        2.658914        1.250210
H          0.021741        2.640273        1.280201
H          0.955287        1.360962        2.163196
H          1.833848        2.658914       -1.250210
H          0.955287        1.360962       -2.163196
H          0.021741        2.640273       -1.280201
```

3_4methyl2pentenal_2_reopt_am1_HEI

Datum	Value
AM1 Energy	-0.120311
AM1 Free Energy (Quasiharmonic)	0.032632
Number of Imaginary Frequencies	0

Frequencies (Top 3 out of 60)

```
1.      42.0022 cm-1
2.      49.7860 cm-1
3.      65.7084 cm-1
```

AM1 Molecular Geometry in Cartesian Coordinates

```
C          -1.764990       -1.657431       -0.192182
C          -0.689330       -0.930291       -0.700083
C           0.190277       -0.137730        0.108883
O          -2.137630       -1.773036        1.008224
H           0.065156       -0.345391        1.199739
C          -1.916910        1.811962       -0.232555
H          -2.140174        2.449962       -1.115411
H          -2.326036        0.784937       -0.403451
H          -2.402619        2.254130        0.664684
S          -0.182490        1.730504        0.003215
H          -2.356535       -2.215632       -0.968859
H          -0.511494       -0.944950       -1.780221
C           1.657259       -0.180203       -0.298982
H           1.745140        0.240855       -1.340220
C           2.521389        0.646833        0.632044
H           2.452172        0.259231        1.677032
H           3.590341        0.603881        0.309948
H           2.188877        1.714665        0.628417
C           2.151375       -1.613876       -0.319892
H           2.102297       -2.058136        0.703128
H           1.510121       -2.225821       -0.999300
H           3.209200       -1.657097       -0.676110
```

3_4methyl2pentenal_3_am1_HEI

Datum	Value
AM1 Energy	-0.119508
AM1 Free Energy (Quasiharmonic)	0.033398
Number of Imaginary Frequencies	0

Frequencies (Top 3 out of 60)

```
1.      39.3768 cm-1
2.      42.0036 cm-1
3.      66.0175 cm-1
```

AM1 Molecular Geometry in Cartesian Coordinates

```
C          -1.368152        1.829605        0.563201
C          -0.365324        0.879601        0.756224
C           0.187783        0.089491       -0.301761
O          -1.925818        2.173437       -0.515955
H          -0.121864        0.474150       -1.306746
```

```
C         -2.161913      -1.533580       0.087246
H         -2.808572      -1.743151      -0.792682
H         -2.408291      -2.255098       0.896443
H         -2.355012      -0.491625       0.445793
S         -0.472790      -1.700887      -0.349482
H         -1.698375       2.354666       1.501041
H         -0.005359       0.705154       1.775001
C          1.687639      -0.170633      -0.278649
H          1.900395      -1.039398      -0.969310
C          2.422686       1.044461      -0.811872
H          2.161519       1.944790      -0.205030
H          3.527009       0.884604      -0.765044
H          2.134557       1.239276      -1.872586
C          2.204217      -0.533209       1.097077
H          2.173405       0.352904       1.775466
H          1.569736      -1.341742       1.535402
H          3.260423      -0.892252       1.032810
```

3_4methyl2pentenal_3_reopt2_am1

Datum	Value
AM1 Energy	-0.059426
AM1 Free Energy (Quasiharmonic)	0.059013
Number of Imaginary Frequencies	0

Frequencies (Top 3 out of 45)

```
  1.      38.1875 cm-1
  2.      95.7473 cm-1
  3.     151.4256 cm-1
```

AM1 Molecular Geometry in Cartesian Coordinates

```
C          2.380350       0.383617       0.165876
C          0.942203       0.649172       0.063601
C          0.056622      -0.300495      -0.264764
C         -1.411666      -0.111448      -0.409947
C         -2.151356      -1.206851       0.337802
O          2.901267      -0.715643      -0.046871
C         -1.899367       1.250307       0.037356
H          2.999671       1.263090       0.457296
H          0.638394       1.686060       0.277329
H          0.409192      -1.330989      -0.465144
H         -1.642339      -0.221632      -1.512546
```

```
H          -3.250142       -1.111359        0.164522
H          -1.820792       -2.213561       -0.013869
H          -1.957682       -1.131620        1.434585
H          -3.008609        1.314093       -0.077262
H          -1.644322        1.428523        1.109683
H          -1.434234        2.056725       -0.579172
```

3_4methyl2pentenal_4_am1_HEI

Datum	Value
AM1 Energy	-0.116439
AM1 Free Energy (Quasiharmonic)	0.036341
Number of Imaginary Frequencies	0

Frequencies (Top 3 out of 60)

```
1.      32.8887 cm-1
2.      57.6985 cm-1
3.      90.8456 cm-1
```

AM1 Molecular Geometry in Cartesian Coordinates

```
C          -2.051115        0.861722        0.079141
C          -1.207611        0.201163       -0.815431
C           0.220889        0.164155       -0.780890
O          -3.313018        0.867562        0.046910
H           0.653400        0.144383       -1.818124
C          -0.409475       -2.341501        0.574446
H          -0.149948       -2.475072        1.647497
H          -1.337222       -1.719884        0.492361
H          -0.593149       -3.339248        0.119981
S           0.908107       -1.554531       -0.271501
H          -1.559686        1.459159        0.892628
H          -1.682308       -0.364827       -1.627783
C           0.937461        1.229338        0.038297
H           0.348060        2.185091       -0.079807
C           1.024385        0.910016        1.516073
H           1.678366        0.018231        1.679621
H           1.450161        1.779926        2.073393
H           0.012776        0.680025        1.927949
C           2.332787        1.470409       -0.509817
H           2.281660        1.835770       -1.563520
H           2.866464        2.235286        0.104265
H           2.921918        0.521344       -0.490639
```

3_4methyl2pentenal_4_am1

Datum	Value
AM1 Energy	-0.060367
AM1 Free Energy (Quasiharmonic)	0.057704
Number of Imaginary Frequencies	0

Frequencies (Top 3 out of 45)

```
1.      39.1490 cm-1
2.      81.0092 cm-1
3.     139.5450 cm-1
```

AM1 Molecular Geometry in Cartesian Coordinates

```
C          2.465483        0.000005        0.331562
C          1.065671        0.000007        0.766433
C          0.039146       -0.000002       -0.093908
C         -1.392150        0.000002        0.319764
C         -2.082458       -1.246674       -0.201738
O          2.826053       -0.000010       -0.849404
C         -2.082457        1.246672       -0.201754
H          3.213138        0.000018        1.158001
H          0.908406        0.000017        1.856763
H          0.219733       -0.000012       -1.184494
H         -1.461828        0.000009        1.445654
H         -3.152845       -1.249294        0.115786
H         -2.042702       -1.280689       -1.317078
H         -1.588093       -2.163256        0.199900
H         -3.152844        1.249297        0.115770
H         -1.588091        2.163258        0.199872
H         -2.042700        1.280672       -1.317095
```

3_4methyl2pentenal_5_am1

Datum	Value
AM1 Energy	-0.0595
AM1 Free Energy (Quasiharmonic)	0.058979

Datum	Value
Number of Imaginary Frequencies	0

Frequencies (Top 3 out of 45)

```
1.      40.5472 cm-1
2.      94.5033 cm-1
3.     150.0735 cm-1
```

AM1 Molecular Geometry in Cartesian Coordinates

```
C         -2.308364       -0.406565       -0.018314
C         -0.964685        0.183649       -0.054599
C          0.123368       -0.547587       -0.320681
C          1.517808       -0.032935       -0.398157
C          1.660465        1.407533        0.045966
O         -3.322862        0.251712        0.224256
C          2.445554       -0.925634        0.407001
H         -2.366977       -1.500476       -0.224770
H         -0.915765        1.265646        0.152764
H          0.035048       -1.632273       -0.518398
H          1.825424       -0.096282       -1.485386
H          2.731108        1.720050       -0.014524
H          1.058337        2.082406       -0.608919
H          1.314876        1.531919        1.100413
H          3.501459       -0.582482        0.289032
H          2.180627       -0.889476        1.490807
H          2.373883       -1.983500        0.057633
```

3_4methyl2pentenal_5_reopt_am1_HEI

Datum	Value
AM1 Energy	-0.120342
AM1 Free Energy (Quasiharmonic)	0.032401
Number of Imaginary Frequencies	0

Frequencies (Top 3 out of 60)

```
1.      37.6703 cm-1
2.      63.4984 cm-1
3.      85.7800 cm-1
```

AM1 Molecular Geometry in Cartesian Coordinates

```
C          2.308400      -0.773182      -0.443171
C          1.065563      -0.762059       0.198250
C         -0.137412      -0.357023      -0.455729
O          3.412027      -1.114526       0.062538
H         -0.054696      -0.375400      -1.572577
C          0.865896       2.236493       0.450057
H          1.262723       3.013587      -0.239178
H          0.615751       2.712052       1.423454
H          1.647682       1.451375       0.613317
S         -0.571912       1.514445      -0.242170
H          2.308805      -0.453278      -1.519929
H          1.018751      -1.063257       1.249238
C         -1.397485      -1.094650      -0.025642
H         -1.189950      -2.193660      -0.183580
C         -1.738528      -0.888949       1.434424
H         -1.847337       0.203159       1.648597
H         -2.695122      -1.407767       1.687720
H         -0.928365      -1.299280       2.083388
C         -2.575435      -0.707665      -0.899133
H         -2.773335       0.389940      -0.814455
H         -2.363299      -0.947802      -1.968666
H         -3.493218      -1.262365      -0.587246
```

3_4methyl2pentenal_6_reopt2_am1

Datum	Value
AM1 Energy	-0.059426
AM1 Free Energy (Quasiharmonic)	0.059013
Number of Imaginary Frequencies	0

Frequencies (Top 3 out of 45)

```
1.      38.1875 cm-1
2.      95.7473 cm-1
3.     151.4256 cm-1
```

AM1 Molecular Geometry in Cartesian Coordinates

```
C          2.380350       0.383617       0.165876
C          0.942203       0.649172       0.063601
C          0.056622      -0.300495      -0.264764
C         -1.411666      -0.111448      -0.409947
C         -2.151356      -1.206851       0.337802
O          2.901267      -0.715643      -0.046871
C         -1.899367       1.250307       0.037356
H          2.999671       1.263090       0.457296
H          0.638394       1.686060       0.277329
H          0.409192      -1.330989      -0.465144
H         -1.642339      -0.221632      -1.512546
H         -3.250142      -1.111359       0.164522
H         -1.820792      -2.213561      -0.013869
H         -1.957682      -1.131620       1.434585
H         -3.008609       1.314093      -0.077262
H         -1.644322       1.428523       1.109683
H         -1.434234       2.056725      -0.579172
```

3_4methyl2pentenal_6_reopt_am1_HEI

Datum	Value
AM1 Energy	-0.120129
AM1 Free Energy (Quasiharmonic)	0.032937
Number of Imaginary Frequencies	0

Frequencies (Top 3 out of 60)

```
1.      41.3156 cm-1
2.      73.9241 cm-1
3.      92.9126 cm-1
```

AM1 Molecular Geometry in Cartesian Coordinates

```
C         -1.747792      -1.117022      -0.298630
C         -1.029035      -0.489182       0.723288
C          0.311820      -0.010831       0.625480
O         -2.933720      -1.540583      -0.225562
H          0.788002       0.119787       1.630071
C         -1.223099       2.332769      -0.256426
H         -1.307316       2.735522      -1.289425
H         -1.917001       1.461835      -0.140185
H         -1.510837       3.128323       0.465151
S          0.428279       1.829322       0.038211
```

```
H          -1.208690       -1.259636       -1.272901
H          -1.548609       -0.308336        1.672323
C           1.264029       -0.746129       -0.305226
H           0.875348       -0.654514       -1.358179
C           2.658229       -0.151197       -0.255922
H           3.087200       -0.239218        0.771387
H           3.332999       -0.688367       -0.965744
H           2.628264        0.930965       -0.537992
C           1.327738       -2.217562        0.059810
H           0.302129       -2.660348        0.031989
H           1.984224       -2.767870       -0.656688
H           1.740238       -2.347715        1.089066
```

3_4methyl2pentenal_7_reopt_am1_HEI

Datum	Value
AM1 Energy	-0.122345
AM1 Free Energy (Quasiharmonic)	0.030568
Number of Imaginary Frequencies	0

Frequencies (Top 3 out of 60)

```
1.      42.1697 cm-1
2.      57.1149 cm-1
3.      83.0870 cm-1
```

AM1 Molecular Geometry in Cartesian Coordinates

```
C          -2.077533        1.016237       -0.453758
C          -0.926422        0.736847        0.288762
C           0.245317        0.154534       -0.285332
O          -3.147617        1.523561       -0.019923
H           0.289990        0.241751       -1.398779
C          -1.286959       -2.245020        0.380262
H          -1.729199       -2.910893       -0.392712
H          -1.235445       -2.796671        1.344228
H          -1.936480       -1.341634        0.506847
S           0.324366       -1.762191       -0.107539
H          -2.028043        0.768337       -1.548186
H          -0.926108        0.955700        1.361407
C           1.560235        0.594454        0.342981
H           1.558000        0.270586        1.421680
C           2.750551       -0.038137       -0.350269
H           2.786079        0.265136       -1.424457
```

```
H          3.701064       0.286763       0.138494
H          2.684247      -1.153575      -0.298056
C          1.683215       2.105685       0.297083
H          1.725081       2.462728      -0.759917
H          0.798034       2.574135       0.791847
H          2.613441       2.436608       0.819238
```

3_4methyl2pentenal_8_reopt_am1_HEI

Datum	Value
AM1 Energy	-0.12123
AM1 Free Energy (Quasiharmonic)	0.031473
Number of Imaginary Frequencies	0

Frequencies (Top 3 out of 60)

```
1.      34.7543 cm-1
2.      50.5304 cm-1
3.      80.3257 cm-1
```

AM1 Molecular Geometry in Cartesian Coordinates

```
C         -1.785349       1.418348      -0.286990
C         -0.632958       0.852384       0.268959
C          0.276125       0.056942      -0.488953
O         -2.641079       2.123672       0.313179
H          0.153228       0.187133      -1.594384
C         -1.651573      -2.002683       0.275269
H         -2.310799      -2.494907      -0.472746
H         -1.628113      -2.622770       1.197801
H         -2.061469      -0.989875       0.521552
S         -0.031104      -1.844687      -0.369710
H         -1.948079       1.227044      -1.381979
H         -0.440211       1.006258       1.335158
C          1.752261       0.134680      -0.128848
H          2.271140      -0.749038      -0.604379
C          2.351380       1.402237      -0.709347
H          1.798012       2.294330      -0.328137
H          3.426255       1.493179      -0.420712
H          2.281969       1.392431      -1.823461
C          2.003762       0.073754       1.362315
H          1.665195       1.014404       1.859232
H          1.442888      -0.786372       1.802446
H          3.094398      -0.060172       1.565108
```

3_4methyl2pentenal_9_am1_HEI

Datum	Value
AM1 Energy	-0.117561
AM1 Free Energy (Quasiharmonic)	0.035662
Number of Imaginary Frequencies	0

Frequencies (Top 3 out of 60)

```
1.      36.2424 cm-1
2.      69.9317 cm-1
3.      79.2250 cm-1
```

AM1 Molecular Geometry in Cartesian Coordinates

```
C          -0.323851       -2.199222       -0.155472
C           0.032654       -1.148027       -0.998519
C          -0.055443        0.257902       -0.737399
O          -0.828796       -2.168373        1.000833
H          -0.143426        0.860323       -1.679530
C           2.508473       -0.345584        0.540190
H           2.674269       -0.156032        1.623293
H           3.494620       -0.445011        0.035998
H           1.932005       -1.296364        0.417623
S           1.623652        0.988062       -0.171543
H          -0.129975       -3.217435       -0.595480
H           0.485868       -1.419521       -1.961308
C          -1.108276        0.708844        0.261729
H          -0.906228        0.178892        1.236953
C          -1.073160        2.207361        0.483319
H          -1.337588        2.751469       -0.455734
H          -1.806189        2.497558        1.274496
H          -0.049583        2.525685        0.803273
C          -2.488614        0.299319       -0.218620
H          -2.703864        0.732545       -1.224782
H          -2.539642       -0.815394       -0.283422
H          -3.269033        0.657724        0.495268
```

4_3-methyl-2-butenal_1_am1

Datum	Value
AM1 Energy	-0.054069
AM1 Free Energy (Quasiharmonic)	0.036886
Number of Imaginary Frequencies	0

Frequencies (Top 3 out of 36)

```
1.      60.0704 cm-1
2.      77.3123 cm-1
3.     112.7599 cm-1
```

AM1 Molecular Geometry in Cartesian Coordinates

```
C         1.588740        0.240165        0.000015
C         0.367788       -0.568816        0.000032
O         2.718980       -0.255595       -0.000028
C        -0.875395       -0.055948        0.000017
C        -2.068956       -0.937396       -0.000015
H        -2.692692       -0.733000       -0.906467
H         1.447523        1.345933        0.000032
H        -2.692611       -0.733189        0.906534
H        -1.787206       -2.018660       -0.000135
C        -1.156956        1.400896       -0.000004
H         0.528534       -1.660732        0.000045
H        -2.257023        1.599715        0.000244
H        -0.714633        1.885717        0.906285
H        -0.715064        1.885566       -0.906585
```

4_3methyl2butenal_1_reopt_am1_HEI

Datum	Value
AM1 Energy	-0.107762
AM1 Free Energy (Quasiharmonic)	0.017065
Number of Imaginary Frequencies	0

Frequencies (Top 3 out of 51)

```
1.      39.9937 cm-1
2.      86.1156 cm-1
3.     139.0825 cm-1
```

```
```

AM1 Molecular Geometry in Cartesian Coordinates

```
C         -2.060942      -0.016149      -0.234404
C         -0.963021       0.338423       0.558441
C          0.299940       0.782940       0.068253
O         -3.180766      -0.416344       0.184236
C          0.682602      -2.167454       0.067595
H          1.077921      -2.759016       0.922120
H         -0.389258      -1.908231       0.263968
H          0.748597      -2.782687      -0.856404
S          1.619125      -0.701662      -0.123062
H         -1.930400       0.077427      -1.344895
H         -1.069740       0.235661       1.645420
C          0.337537       1.355403      -1.325432
H         -0.008448       0.603608      -2.074112
H         -0.329331       2.251304      -1.386701
H          1.376163       1.666442      -1.596636
C          1.077818       1.637738       1.040470
H          2.136315       1.763264       0.704820
H          0.606112       2.649573       1.112217
H          1.078600       1.174592       2.055771
```

4_3-methyl-2-butenal_2_am1

Datum	Value
AM1 Energy	-0.054229
AM1 Free Energy (Quasiharmonic)	0.036991
Number of Imaginary Frequencies	0

Frequencies (Top 3 out of 36)

```
  1.      70.6709 cm-1
  2.      99.9176 cm-1
  3.     123.2436 cm-1
```

AM1 Molecular Geometry in Cartesian Coordinates

```
C          1.632233      -0.651985       0.000001
C          0.204438      -0.958564      -0.000000
O          2.112752       0.486943       0.000001
```

```
C        -0.783228      -0.041171       0.000018
C        -2.209913      -0.446770      -0.000000
H        -2.721260      -0.034688      -0.906308
H         2.297988      -1.546915      -0.000012
H        -2.721305      -0.034635       0.906257
H        -2.328836      -1.557666       0.000028
C        -0.523595       1.416771      -0.000003
H        -0.038166      -2.034147      -0.000009
H        -1.473192       2.003951      -0.000492
H         0.081173       1.699564       0.900039
H         0.081977       1.699309      -0.899601
```

4_3methyl2butenal_2_reopt_am1_HEI

Datum	Value
AM1 Energy	-0.107762
AM1 Free Energy (Quasiharmonic)	0.017064
Number of Imaginary Frequencies	0

Frequencies (Top 3 out of 51)

```
1.      39.9537 cm-1
2.      86.1197 cm-1
3.     139.0786 cm-1
```

AM1 Molecular Geometry in Cartesian Coordinates

```
C         2.061000      -0.016250       0.234489
C         0.963082       0.338096      -0.558487
C        -0.299767       0.783043      -0.068420
O         3.180734      -0.416816      -0.184017
C        -0.683045      -2.167374      -0.067926
H         0.388876      -1.908201      -0.264038
H        -0.749247      -2.783171       0.855684
H        -1.078355      -2.758356      -0.922861
S        -1.619265      -0.701542       0.123648
H         1.930525       0.077932       1.344935
H         1.069717       0.234764      -1.645419
C        -0.337282       1.355882       1.325097
H         0.008535       0.604209       2.073983
H         0.329763       2.251667       1.386122
H        -1.375856       1.667199       1.596190
C        -1.077553       1.637631      -1.040863
H        -2.136142       1.763042      -0.705445
```

```
H          -0.605972        2.649532       -1.112594
H          -1.078077        1.174415       -2.056135
```

4_3methyl2butenal_3_am1_HEI

Datum	Value
AM1 Energy	-0.107762
AM1 Free Energy (Quasiharmonic)	0.017065
Number of Imaginary Frequencies	0

Frequencies (Top 3 out of 51)

```
 1.      40.0050 cm-1
 2.      86.1110 cm-1
 3.     139.0805 cm-1
```

AM1 Molecular Geometry in Cartesian Coordinates

```
C          -2.060954       -0.016182       -0.234406
C          -0.963031        0.338376        0.558444
C           0.299906        0.782949        0.068264
O          -3.180765       -0.416420        0.184228
C           0.682692       -2.167451        0.067607
H           1.078037       -2.758989        0.922136
H          -0.389174       -1.908258        0.263986
H           0.748699       -2.782696       -0.856383
S           1.619159       -0.701626       -0.123086
H          -1.930428        0.077450       -1.344893
H          -1.069739        0.235560        1.645419
C           0.337482        1.355427       -1.325415
H          -0.008499        0.603635       -2.074099
H          -0.329398        2.251320       -1.386663
H           1.376101        1.666482       -1.596623
C           1.077761        1.637768        1.040480
H           2.136271        1.763273        0.704859
H           0.606064        2.649610        1.112180
H           1.078512        1.174655        2.055796
```

4_3methyl2butenal_4_am1_HEI

Datum	Value
AM1 Energy	-0.103311
AM1 Free Energy (Quasiharmonic)	0.021565
Number of Imaginary Frequencies	0

Frequencies (Top 3 out of 51)

```
1.      51.7373 cm-1
2.      70.0890 cm-1
3.      88.9743 cm-1
```

AM1 Molecular Geometry in Cartesian Coordinates

```
C        -2.251965      -0.322875       0.272186
C        -1.225323       0.200006      -0.520385
C         0.065116       0.605660      -0.039347
O        -3.395833      -0.673226      -0.127445
C         2.724038      -0.736458       0.206529
H         3.243331      -0.014363      -0.463066
H         2.799115      -0.354075       1.249294
H         3.243628      -1.718500       0.144801
S         1.058100      -0.983280      -0.270698
H        -2.040303      -0.418951       1.370123
H        -1.395199       0.264190      -1.601387
C         0.157992       0.980385       1.420769
H        -0.111137       0.113020       2.069676
H         1.195447       1.306113       1.681243
H        -0.542519       1.822437       1.642423
C         0.725593       1.659439      -0.901946
H         0.700745       1.355812      -1.975559
H         0.181466       2.629993      -0.794954
H         1.789776       1.815680      -0.598700
```

4_3methyl2butenal_5_am1_HEI

Datum	Value
AM1 Energy	-0.105601
AM1 Free Energy (Quasiharmonic)	0.019419
Number of Imaginary Frequencies	0

Frequencies (Top 3 out of 51)

```
1.      38.6334 cm-1
2.      54.5255 cm-1
3.     118.5943 cm-1
```

AM1 Molecular Geometry in Cartesian Coordinates

```
C          2.066963        0.273444       -0.613084
C          0.845656       -0.310492       -0.948040
C         -0.171142       -0.771070       -0.048794
O          2.568011        0.500729        0.522171
C         -0.868244        2.084495       -0.067241
H         -0.902228        2.645646        0.892221
H          0.198792        1.944992       -0.373812
H         -1.402206        2.670839       -0.846677
S         -1.635479        0.521202        0.116789
H          2.685639        0.567389       -1.506892
H          0.612661       -0.393439       -2.017665
C          0.258321       -0.998744        1.376750
H          0.729367       -0.072104        1.787831
H         -0.611230       -1.287910        2.014963
H          1.021184       -1.815734        1.413267
C         -0.987059       -1.929270       -0.577972
H         -1.307308       -1.738672       -1.629912
H         -0.370360       -2.861784       -0.557977
H         -1.897709       -2.094461        0.048929
```

4_3methyl2butenal_6_am1_HEI_reopt

Datum	Value
AM1 Energy	-0.105601
AM1 Free Energy (Quasiharmonic)	0.019417
Number of Imaginary Frequencies	0

Frequencies (Top 3 out of 51)

```
1.      38.6253 cm-1
2.      54.5084 cm-1
3.     118.4650 cm-1
```

AM1 Molecular Geometry in Cartesian Coordinates

```
C          2.066923       0.273430       -0.613266
C          0.845539      -0.310434       -0.948040
C         -0.171138      -0.770995       -0.048645
O          2.568137       0.500688        0.521928
C         -0.868315       2.084389       -0.066825
H          0.198855       1.944854       -0.372891
H         -1.401822       2.671025       -0.846353
H         -0.902774       2.645232        0.892794
S         -1.635687       0.521031        0.116214
H          2.685492       0.567341       -1.507156
H          0.612378      -0.393396       -2.017626
C          0.258438      -0.998065        1.376964
H          0.728827      -0.071020        1.787856
H         -0.610919      -1.287716        2.015211
H          1.021902      -1.814478        1.413663
C         -0.986753      -1.929596       -0.577418
H         -1.306872      -1.739545       -1.629494
H         -0.369867      -2.861972       -0.556890
H         -1.897470      -2.094709        0.049414
```

4_3methyl2butenal_7_reopt_am1_HEI

Datum	Value
AM1 Energy	-0.103311
AM1 Free Energy (Quasiharmonic)	0.021565
Number of Imaginary Frequencies	0

Frequencies (Top 3 out of 51)

```
1.     51.7376 cm-1
2.     70.0892 cm-1
3.     88.9747 cm-1
```

AM1 Molecular Geometry in Cartesian Coordinates

```
C          2.251965      -0.322874        0.272186
C          1.225322       0.200006       -0.520385
C         -0.065116       0.605660       -0.039347
O          3.395833      -0.673226       -0.127445
C         -2.724039      -0.736458        0.206529
H         -2.799116      -0.354069        1.249292
H         -3.243332      -0.014366       -0.463069
H         -3.243627      -1.718500        0.144806
```

```
S          -1.058100       -0.983281       -0.270698
H           2.040304       -0.418948        1.370123
H           1.395198        0.264188       -1.601387
C          -0.725593        1.659438       -0.901946
H          -0.700746        1.355811       -1.975559
H          -1.789776        1.815679       -0.598700
H          -0.181467        2.629992       -0.794954
C          -0.157991        0.980385        1.420769
H          -1.195445        1.306115        1.681243
H           0.542522        1.822434        1.642423
H           0.111135        0.113020        2.069676
```

4_3methyl2butenal_8_reopt_am1_HEI

Datum	Value
AM1 Energy	-0.101531
AM1 Free Energy (Quasiharmonic)	0.023557
Number of Imaginary Frequencies	0

Frequencies (Top 3 out of 51)

```
1.      45.2673 cm-1
2.      58.7695 cm-1
3.      85.7882 cm-1
```

AM1 Molecular Geometry in Cartesian Coordinates

```
C           2.342417       -0.373082       -0.513739
C           1.141117        0.228752       -0.887244
C           0.034357        0.551416       -0.024626
O           2.765902       -0.682146        0.633568
C          -2.675269       -0.612634        0.383746
H          -2.505075       -0.493745        1.477293
H          -3.234435        0.279959        0.022760
H          -3.298676       -1.518227        0.211944
S          -1.169450       -0.830897       -0.481800
H           3.032388       -0.577271       -1.379867
H           0.994243        0.441315       -1.953461
C          -0.660832        1.847357       -0.382982
H          -0.901358        1.876427       -1.472272
H          -1.608954        1.966325        0.196544
H           0.008707        2.710193       -0.144743
C           0.306659        0.474403        1.455450
H           0.707946       -0.535242        1.718660
```

```
H          1.080430        1.231071        1.735057
H         -0.621929        0.673434        2.044703
```

5_trans-2-methyl-2-butenal_1_am1

Datum	Value
AM1 Energy	-0.053372
AM1 Free Energy (Quasiharmonic)	0.037279
Number of Imaginary Frequencies	0

Frequencies (Top 3 out of 36)

```
1.      23.0622 cm-1
2.      68.1120 cm-1
3.     101.4815 cm-1
```

AM1 Molecular Geometry in Cartesian Coordinates

```
C          1.362737       -0.712663        0.000036
C          0.066348       -0.005233        0.000006
O          2.456845       -0.144760       -0.000047
C         -1.071915       -0.722142       -0.000006
C         -2.433065       -0.158334       -0.000018
H          1.295333       -1.825981        0.000130
H         -1.030131       -1.826794       -0.000004
C          0.102242        1.475521        0.000020
H         -2.989381       -0.509152        0.906327
H         -2.428993        0.959363       -0.000106
H         -2.989424       -0.509299       -0.906278
H         -0.414130        1.879807       -0.906370
H         -0.413960        1.879767        0.906526
H          1.157835        1.847473       -0.000080
```

5_trans2methyl2butenal_1_reopt_am1_HEI

Datum	Value
AM1 Energy	-0.116606
AM1 Free Energy (Quasiharmonic)	0.008834

Datum	Value
Number of Imaginary Frequencies	0

Frequencies (Top 3 out of 51)

```
1.      36.3045 cm-1
2.      73.2536 cm-1
3.     104.6237 cm-1
```

AM1 Molecular Geometry in Cartesian Coordinates

```
C          -1.951259       -0.573187       -0.058607
C          -0.971456        0.388925        0.222040
C           0.175435        0.554687       -0.636580
O          -1.990623       -1.374517       -1.032346
C           1.229403       -1.803552        0.626503
H           0.112150       -1.838719        0.635885
H           1.616313       -2.629721       -0.009321
H           1.612202       -1.935392        1.661997
S           1.765737       -0.257313       -0.003245
H          -2.800691       -0.614621        0.675771
C           0.656660        1.967083       -0.834597
H           0.898394        2.447240        0.144529
H           1.570843        1.989680       -1.477442
H          -0.143369        2.568988       -1.332170
H           0.018501        0.036196       -1.616570
C          -1.104511        1.240477        1.414191
H          -0.160645        1.241645        2.018923
H          -1.318313        2.306870        1.134521
H          -1.937823        0.894373        2.076867
```

5_trans2methyl2butenal_2_am1_HEI

Datum	Value
AM1 Energy	-0.117426
AM1 Free Energy (Quasiharmonic)	0.008212
Number of Imaginary Frequencies	0

Frequencies (Top 3 out of 51)

```
1.      37.4126 cm-1
2.      63.7547 cm-1
3.     116.6724 cm-1
```

AM1 Molecular Geometry in Cartesian Coordinates

```
C          -1.778300       -0.393026       -0.731005
C          -0.850535        0.390527       -0.022498
C           0.423062        0.709899       -0.606966
O          -2.923756       -0.751777       -0.348652
C           1.082158       -1.875572        0.581990
H          -0.021951       -1.705495        0.654572
H           1.276057       -2.790397       -0.019556
H           1.498168       -2.020273        1.602887
S           1.847132       -0.494028       -0.176373
H          -1.448449       -0.721692       -1.752999
C           1.028368        2.027006       -0.205032
H           1.140267        2.090492        0.904207
H           2.035270        2.159924       -0.671712
H           0.366845        2.864484       -0.539754
H           0.421385        0.600257       -1.721334
C          -1.177039        0.876082        1.327487
H          -2.170145        0.479003        1.655723
H          -1.223100        1.996924        1.360362
H          -0.404682        0.555941        2.074941
```

5_trans-2-methyl-2-butenal_2_am1

Datum	Value
AM1 Energy	-0.054036
AM1 Free Energy (Quasiharmonic)	0.036459
Number of Imaginary Frequencies	0

Frequencies (Top 3 out of 36)

```
1.      28.2389 cm-1
2.      74.0283 cm-1
3.      79.5065 cm-1
```

AM1 Molecular Geometry in Cartesian Coordinates

```
C          1.566282       0.160257      -0.000034
C          0.100707       0.321959      -0.000004
O          2.137102      -0.934168       0.000033
C         -0.696768      -0.762592      -0.000004
C         -2.169089      -0.742283       0.000000
H          2.149247       1.110212      -0.000126
H         -0.235987      -1.769221      -0.000030
C         -0.410919       1.711971       0.000017
H         -2.554823      -1.276124       0.905859
H         -2.581314       0.296515       0.000589
H         -2.554790      -1.275077      -0.906491
H         -1.041394       1.897005      -0.905938
H         -1.041542       1.896913       0.905888
H          0.422510       2.457244       0.000133
```

5_trans2methyl2butenal_3_am1_HEI

Datum	Value
AM1 Energy	-0.111744
AM1 Free Energy (Quasiharmonic)	0.01323
Number of Imaginary Frequencies	0

Frequencies (Top 3 out of 51)

```
1.      36.8456 cm-1
2.      58.3821 cm-1
3.      81.7791 cm-1
```

AM1 Molecular Geometry in Cartesian Coordinates

```
C          2.156158      -0.708557      -0.086203
C          1.190888       0.309202      -0.095520
C         -0.083811       0.121993       0.556095
O          2.099616      -1.821751       0.502538
C         -2.783068      -0.712463       0.035846
H         -2.561112      -1.423105       0.863298
H         -3.323791       0.161136       0.465214
H         -3.446011      -1.213106      -0.704414
S         -1.319086      -0.230693      -0.794475
H          3.096177      -0.474698      -0.656013
C         -0.594621       1.304648       1.336885
H         -0.682852       2.205418       0.682867
H         -1.597107       1.087453       1.781259
```

```
H          0.116418       1.542391       2.166270
H         -0.084699      -0.800257       1.192332
C          1.471916       1.580131      -0.780225
H          0.604155       1.905293      -1.410013
H          1.673422       2.406487      -0.045846
H          2.369078       1.498354      -1.444927
```

5_trans2methyl2butenal_4_am1_HEI_reopt

Datum	Value
AM1 Energy	-0.116627
AM1 Free Energy (Quasiharmonic)	0.008795
Number of Imaginary Frequencies	0

Frequencies (Top 3 out of 51)

```
1.      38.8314 cm-1
2.      58.1472 cm-1
3.      98.4130 cm-1
```

AM1 Molecular Geometry in Cartesian Coordinates

```
C          1.673431       0.240940      -0.711696
C          0.855181       0.129313       0.423889
C         -0.404457       0.809608       0.553092
O          2.788209      -0.315794      -0.901685
C         -1.299604      -1.675240      -0.697266
H         -1.680264      -1.799770      -1.734462
H         -0.180861      -1.642543      -0.711558
H         -1.630909      -2.541548      -0.083930
S         -1.930637      -0.188424      -0.017783
H          1.286005       0.903481      -1.530104
C         -0.569766       2.107141      -0.189691
H         -0.529782       1.951102      -1.294298
H         -1.553019       2.577647       0.058620
H          0.245696       2.816647       0.096693
H         -0.691181       0.941176       1.629070
C          1.272630      -0.736330       1.542021
H          2.271049      -1.194180       1.331258
H          0.535488      -1.564320       1.713799
H          1.347818      -0.159150       2.500829
```

5_trans2methyl2butenal_5_am1_HEI

Datum	Value
AM1 Energy	-0.116627
AM1 Free Energy (Quasiharmonic)	0.008793
Number of Imaginary Frequencies	0

Frequencies (Top 3 out of 51)

```
1.      38.8066 cm-1
2.      58.1445 cm-1
3.      98.4129 cm-1
```

AM1 Molecular Geometry in Cartesian Coordinates

```
C          1.673534        0.240911       -0.711624
C          0.855278        0.129393        0.423978
C         -0.404311        0.809755        0.553117
O          2.788211       -0.316001       -0.901656
C         -1.299765       -1.675030       -0.697636
H         -0.181018       -1.642521       -0.711836
H         -1.631194       -2.541604       -0.084739
H         -1.680377       -1.799027       -1.734912
S         -1.930680       -0.188474       -0.017495
H          1.286219        0.903583       -1.529981
C         -0.569691        2.107076       -0.189979
H         -0.529542        1.950801       -1.294548
H         -1.553022        2.577537        0.058104
H          0.245665        2.816731        0.096345
H         -0.691074        0.941471        1.629055
C          1.272554       -0.736362        1.542086
H          2.271010       -1.194184        1.331451
H          0.535366       -1.564369        1.713601
H          1.347560       -0.159296        2.500972
```

5_trans2methyl2butenal_6_reopt_am1_HEI

Datum	Value
AM1 Energy	-0.117426
AM1 Free Energy (Quasiharmonic)	0.008211
Number of Imaginary Frequencies	0

Frequencies (Top 3 out of 51)

```
1.      37.4269 cm-1
2.      63.7176 cm-1
3.     116.5954 cm-1
```

AM1 Molecular Geometry in Cartesian Coordinates

```
C         -1.778368       -0.392262       -0.731306
C         -0.850355        0.390680       -0.022464
C          0.423249        0.710104       -0.606860
O         -2.923820       -0.751047       -0.348976
C          1.081578       -1.875319        0.582346
H          1.497161       -2.019159        1.603541
H         -0.022527       -1.705133        0.654394
H          1.275731       -2.790629       -0.018375
S          1.846836       -0.494501       -0.177053
H         -1.448764       -0.720287       -1.753585
C          1.028818        2.026938       -0.204389
H          1.140482        2.090077        0.904887
H          2.035869        2.159710       -0.670793
H          0.367615        2.864699       -0.539026
H          0.421433        0.601087       -1.721298
C         -1.176533        0.875171        1.327998
H         -2.169693        0.478067        1.656031
H         -1.222299        1.995986        1.361904
H         -0.404150        0.554102        2.075021
```

5_trans2methyl2butenal_7_reopt_am1_HEI

Datum	Value
AM1 Energy	-0.11277
AM1 Free Energy (Quasiharmonic)	0.01257
Number of Imaginary Frequencies	0

Frequencies (Top 3 out of 51)

```
1.      49.3630 cm-1
2.      62.4772 cm-1
3.      75.2676 cm-1
```

AM1 Molecular Geometry in Cartesian Coordinates

```
C          -1.903124       -0.830654       -0.502674
C          -1.079513        0.225677       -0.073682
C           0.264302        0.342926       -0.576634
O          -3.098391       -1.048413       -0.170439
C           2.949565       -0.643483       -0.096749
H           2.941249       -0.820319       -1.195688
H           3.451180        0.332539        0.091554
H           3.539944       -1.448221        0.395113
S           1.329998       -0.681272        0.565789
H          -1.432365       -1.539955       -1.234651
C           0.826764        1.738401       -0.621702
H           0.832195        2.196380        0.396776
H           1.873184        1.732572       -1.015213
H           0.199112        2.378445       -1.289922
H           0.402846       -0.156521       -1.570357
C          -1.581327        1.200714        0.906854
H          -2.603988        0.910099        1.255418
H          -1.645857        2.230659        0.463587
H          -0.910341        1.270490        1.801799
```

6_2ethylacrolein_10_reopt_am1_HEI

Datum	Value
AM1 Energy	-0.121377
AM1 Free Energy (Quasiharmonic)	0.005255
Number of Imaginary Frequencies	0

Frequencies (Top 3 out of 51)

```
  1.      32.5404 cm-1
  2.      55.7350 cm-1
  3.      76.5164 cm-1
```

AM1 Molecular Geometry in Cartesian Coordinates

```
C           1.414404       -1.261217       -0.050301
O           2.524349       -1.220538       -0.644763
C           0.738565       -0.198618        0.572268
C          -0.509369       -0.419532        1.244794
C           1.334363        1.154066        0.581519
```

```
C          0.739523       2.082998       -0.454145
H          0.894491      -2.255403        0.005781
H         -0.698527       0.301890        2.075245
H         -0.633550      -1.467004        1.609051
H          1.202415       1.625444        1.595660
H          2.440304       1.068994        0.395412
H          1.145643       3.117775       -0.340579
H          0.980924       1.719568       -1.481879
H         -0.372561       2.117702       -0.349660
C         -1.598513      -0.189746       -1.422773
H         -1.963693       0.736350       -1.918055
H         -0.481322      -0.226595       -1.490019
H         -2.031118      -1.072894       -1.941154
S         -2.086977      -0.181826        0.260633
```

6_2ethylacrolein_11_reopt_am1_HEI

Datum	Value
AM1 Energy	-0.120847
AM1 Free Energy (Quasiharmonic)	0.005594
Number of Imaginary Frequencies	0

Frequencies (Top 3 out of 51)

```
1.      34.3893 cm-1
2.      51.0513 cm-1
3.      85.6959 cm-1
```

AM1 Molecular Geometry in Cartesian Coordinates

```
C         -1.505974      -1.145253       -0.240060
O         -1.154786      -2.341667       -0.425362
C         -0.844006      -0.160319        0.506980
C          0.390716      -0.436154        1.189070
C         -1.440225       1.188688        0.606646
C         -1.274093       2.005227       -0.657278
H         -2.468252      -0.809944       -0.713750
H          0.563638      -1.532044        1.305056
H          0.492314       0.086753        2.171144
H         -2.541032       1.110011        0.831821
H         -0.976179       1.753271        1.460637
H         -1.704905       3.028622       -0.533125
H         -1.790644       1.504388       -1.511270
H         -0.190455       2.099142       -0.912350
```

```
C          1.652768        0.000448       -1.341243
H          0.575310       -0.249643       -1.507751
H          1.894712        0.954818       -1.858039
H          2.287288       -0.810444       -1.760370
S          1.951336        0.180162        0.377887
```

6_2ethylacrolein_1_am1

Datum	Value
AM1 Energy	-0.047014
AM1 Free Energy (Quasiharmonic)	0.044819
Number of Imaginary Frequencies	0

Frequencies (Top 3 out of 36)

```
1.      36.5757 cm-1
2.      67.7121 cm-1
3.     160.3459 cm-1
```

AM1 Molecular Geometry in Cartesian Coordinates

```
C          1.348875       -0.330276       -0.156736
O          1.309480       -1.530756        0.118017
C          0.239838        0.611902        0.100649
C          0.404546        1.921446       -0.119824
C         -1.033120        0.036342        0.618212
C         -1.820540       -0.656036       -0.468639
H          2.253443        0.123369       -0.625584
H         -0.393794        2.652404        0.066988
H          1.346314        2.343528       -0.497123
H         -0.787799       -0.706828        1.425938
H         -1.659817        0.845205        1.079935
H         -2.757555       -1.088877       -0.042658
H         -2.097077        0.060785       -1.278403
H         -1.217150       -1.483806       -0.915200
```

6_2ethylacrolein_1_reopt_am1_HEI

Datum	Value
AM1 Energy	-0.120847

Datum	Value
AM1 Free Energy (Quasiharmonic)	0.005594
Number of Imaginary Frequencies	0

Frequencies (Top 3 out of 51)

```
1.      34.2873 cm-1
2.      51.0915 cm-1
3.      85.7007 cm-1
```

AM1 Molecular Geometry in Cartesian Coordinates

```
C         -1.506147        1.145129        0.240155
O         -1.155057        2.341603        0.425243
C         -0.844199        0.160174       -0.506881
C          0.390415        0.436064       -1.189133
C         -1.440320       -1.188894       -0.606321
C         -1.273064       -2.005664        0.657311
H         -2.468306        0.809776        0.714056
H          0.563205        1.531954       -1.305301
H          0.491986       -0.086980       -2.171136
H         -2.541317       -1.110338       -0.830616
H         -0.976857       -1.753240       -1.460777
H         -1.703480       -3.029226        0.533171
H         -0.189231       -2.099156        0.911690
H         -1.789326       -1.505283        1.511743
C          1.652804        0.000104        1.341122
H          2.286884        0.811497        1.759944
H          0.575210        0.249594        1.507727
H          1.895411       -0.953978        1.858132
S          1.951209       -0.179932       -0.378006
```

6_2ethylacrolein_2_am1_HEI_reopt

Datum	Value
AM1 Energy	-0.12141
AM1 Free Energy (Quasiharmonic)	0.004745
Number of Imaginary Frequencies	0

Frequencies (Top 3 out of 51)

```
1.      33.1763 cm-1
2.      46.2138 cm-1
3.      92.4143 cm-1
```

AM1 Molecular Geometry in Cartesian Coordinates

```
C          -0.817237        1.621743        0.155635
O          -0.144772        2.525887       -0.412173
C          -0.729547        0.237910       -0.041018
C           0.201118       -0.335167       -0.976376
C          -1.595589       -0.671720        0.737349
C          -2.814370       -1.132320       -0.034286
H          -1.588339        1.947915        0.905045
H           0.553611        0.413649       -1.723842
H          -0.183627       -1.253599       -1.483390
H          -1.008606       -1.579303        1.050112
H          -1.944511       -0.168207        1.680786
H          -2.504388       -1.653585       -0.971639
H          -3.430488       -1.837544        0.575637
H          -3.450430       -0.259492       -0.315806
C           2.296032        0.187020        0.881342
H           2.500799       -0.287227        1.865712
H           1.497349        0.960045        0.998490
H           3.226058        0.673473        0.514335
S           1.765519       -1.038001       -0.258371
```

6_2ethylacrolein_2_am1

Datum	Value
AM1 Energy	-0.047014
AM1 Free Energy (Quasiharmonic)	0.044819
Number of Imaginary Frequencies	0

Frequencies (Top 3 out of 36)

```
1.      36.5535 cm-1
2.      67.7163 cm-1
3.     160.3848 cm-1
```

AM1 Molecular Geometry in Cartesian Coordinates

```
C          -1.348967       -0.330004       -0.156822
O          -1.309926       -1.530475        0.118017
C          -0.239717        0.611909        0.100639
C          -0.404128        1.921500       -0.119758
C           1.033080        0.036010        0.618238
C           1.820554       -0.656257       -0.468644
H          -2.253362        0.123856       -0.625802
H           0.394360        2.652278        0.067132
H          -1.345790        2.343811       -0.497069
H           1.659853        0.844670        1.080220
H           0.787512       -0.707287        1.425775
H           2.757486       -1.089249       -0.042624
H           1.217155       -1.483903       -0.915429
H           2.097254        0.060677       -1.278254
```

6_2ethylacrolein_3_am1

Datum	Value
AM1 Energy	-0.04719
AM1 Free Energy (Quasiharmonic)	0.044666
Number of Imaginary Frequencies	0

Frequencies (Top 3 out of 36)

```
  1.      28.6850 cm-1
  2.      72.7218 cm-1
  3.     207.0465 cm-1
```

AM1 Molecular Geometry in Cartesian Coordinates

```
C           1.586100        0.213522        0.000001
O           2.106915       -0.903217       -0.000001
C           0.123348        0.436836        0.000000
C          -0.358699        1.684647       -0.000001
C          -0.709031       -0.796025       -0.000000
C          -2.195215       -0.552809        0.000001
H           2.206905        1.139631        0.000004
H          -1.434463        1.905767       -0.000001
H           0.297176        2.566744       -0.000000
H          -0.426545       -1.408053       -0.901401
H          -0.426543       -1.408055        0.901399
H          -2.506631        0.021160        0.906171
```

```
H          -2.506630      0.021172      -0.906162
H          -2.737602     -1.529660      -0.000006
```

6_2ethylacrolein_3_reopt_am1_HEI

Datum	Value
AM1 Energy	-0.120847
AM1 Free Energy (Quasiharmonic)	0.005594
Number of Imaginary Frequencies	0

Frequencies (Top 3 out of 51)

```
1.      34.2914 cm-1
2.      51.0985 cm-1
3.      85.6876 cm-1
```

AM1 Molecular Geometry in Cartesian Coordinates

```
C          -1.505999     -1.145243       0.240149
O          -1.154783     -2.341661       0.425355
C          -0.844127     -0.160271      -0.506926
C           0.390533     -0.436073      -1.189138
C          -1.440343      1.188749      -0.606478
C          -1.273468      2.005491       0.657219
H          -2.468216     -0.809960       0.713978
H           0.492086      0.086965      -2.171141
H           0.563399     -1.531953      -1.305281
H          -0.976734      1.753162      -1.460821
H          -2.541270      1.110095      -0.831038
H          -1.789842      1.504993       1.511511
H          -1.703999      3.028996       0.533035
H          -0.189696      2.099093       0.911852
C           1.652627      0.000328       1.341181
H           0.575019     -0.249146       1.507660
H           2.286666     -0.810957       1.760265
H           1.895120      0.954512       1.858057
S           1.951275      0.179976      -0.377935
```

6_2ethylacrolein_4_am1

Datum	Value
AM1 Energy	-0.048143
AM1 Free Energy (Quasiharmonic)	0.043885
Number of Imaginary Frequencies	0

Frequencies (Top 3 out of 36)

```
1.      51.4616 cm-1
2.      89.0565 cm-1
3.     150.0486 cm-1
```

AM1 Molecular Geometry in Cartesian Coordinates

```
C          1.007171       -0.765542        0.175666
O          2.198485       -0.735738       -0.142938
C          0.122178        0.415169        0.153357
C          0.581853        1.621352       -0.201901
C         -1.290965        0.177674        0.560125
C         -2.055089       -0.616840       -0.471758
H          0.522094       -1.712059        0.508097
H         -0.056296        2.514759       -0.222336
H          1.632141        1.774164       -0.492134
H         -1.301942       -0.373945        1.539733
H         -1.809102        1.159704        0.729932
H         -3.100787       -0.792506       -0.121192
H         -2.094948       -0.067773       -1.443184
H         -1.569930       -1.607317       -0.648351
```

6_2ethylacrolein_4_reopt_am1_HEI

Datum	Value
AM1 Energy	-0.12141
AM1 Free Energy (Quasiharmonic)	0.004744
Number of Imaginary Frequencies	0

Frequencies (Top 3 out of 51)

```
1.      33.1236 cm-1
2.      46.2683 cm-1
3.      92.4119 cm-1
```

AM1 Molecular Geometry in Cartesian Coordinates

```
C         -0.816912      -1.621969      -0.155637
O         -0.144279      -2.525904       0.412314
C         -0.729613      -0.238108       0.040930
C          0.200932       0.335295       0.976211
C         -1.595874       0.671253      -0.737502
C         -2.814302       1.132305       0.034423
H         -1.587833      -1.948405      -0.905120
H          0.553577      -0.413362       1.723766
H         -0.184019       1.253708       1.483107
H         -1.945221       0.167363      -1.680574
H         -1.008934       1.578653      -1.050893
H         -3.430571       1.837348      -0.575552
H         -3.450363       0.259682       0.316563
H         -2.503895       1.653922       0.971441
C          2.296221      -0.186900      -0.881037
H          1.498043      -0.960531      -0.997549
H          2.500367       0.286906      -1.865748
H          3.226703      -0.672450      -0.514000
S          1.765229       1.038321       0.258232
```

6_2ethylacrolein_5_am1

Datum	Value
AM1 Energy	-0.048143
AM1 Free Energy (Quasiharmonic)	0.043885
Number of Imaginary Frequencies	0

Frequencies (Top 3 out of 36)

```
  1.      51.4614 cm-1
  2.      89.0563 cm-1
  3.     150.0484 cm-1
```

AM1 Molecular Geometry in Cartesian Coordinates

```
C         -1.007171      -0.765542       0.175666
O         -2.198484      -0.735739      -0.142938
C         -0.122178       0.415169       0.153357
```

```
C          -0.581854       1.621352       -0.201901
C           1.290965       0.177674        0.560125
C           2.055089      -0.616840       -0.471757
H          -0.522094      -1.712059        0.508097
H           0.056295       2.514759       -0.222336
H          -1.632142       1.774163       -0.492134
H           1.809102       1.159705        0.729931
H           1.301942      -0.373943        1.539734
H           3.100787      -0.792506       -0.121192
H           1.569930      -1.607317       -0.648350
H           2.094947      -0.067774       -1.443184
```

6_2ethylacrolein_5_reopt_am1_HEI

Datum	Value
AM1 Energy	-0.12141
AM1 Free Energy (Quasiharmonic)	0.004745
Number of Imaginary Frequencies	0

Frequencies (Top 3 out of 51)

```
1.      33.1417 cm-1
2.      46.1704 cm-1
3.      92.4727 cm-1
```

AM1 Molecular Geometry in Cartesian Coordinates

```
C          -0.816594      -1.621832       -0.155511
O          -0.143651      -2.525616        0.412309
C          -0.729550      -0.237940        0.041035
C           0.200978       0.335643        0.976223
C          -1.596051       0.671227       -0.737351
C          -2.814963       1.131403        0.034325
H          -1.587592      -1.948426       -0.904847
H           0.553609      -0.412857        1.723940
H          -0.183953       1.254167        1.482922
H          -1.944834       0.167459       -1.680702
H          -1.009497       1.579029       -1.050280
H          -2.505133       1.652905        0.971595
H          -3.450635       0.258340        0.315990
H          -3.431417       1.836301       -0.575633
C           2.296185      -0.187109       -0.880846
H           3.226210      -0.673191       -0.513345
H           1.497626      -0.960314       -0.997625
```

```
H          2.501071        0.286542        -1.865476
S          1.765233        1.038538         0.257984
```

6_2ethylacrolein_6_am1_HEI

Datum	Value
AM1 Energy	-0.122157
AM1 Free Energy (Quasiharmonic)	0.004061
Number of Imaginary Frequencies	0

Frequencies (Top 3 out of 51)

```
1.      31.0704 cm-1
2.      52.2710 cm-1
3.      74.3407 cm-1
```

AM1 Molecular Geometry in Cartesian Coordinates

```
C          1.016382        1.406437         0.532189
O          1.933849        2.048892        -0.044824
C          0.620162        0.080850         0.290910
C         -0.459219       -0.509271         1.029375
C          1.295205       -0.719039        -0.752313
C          2.194486       -1.798344        -0.189804
H          0.440502        1.937823         1.336847
H         -0.635141       -0.027499         2.020005
H         -0.360066       -1.614255         1.150818
H          0.529284       -1.206467        -1.418133
H          1.913588       -0.036078        -1.397048
H          2.677664       -2.379843        -1.012952
H          2.996004       -1.344852         0.440570
H          1.610385       -2.507740         0.444530
C         -2.012084        0.671296        -1.104188
H         -2.681121        1.547666        -0.961152
H         -0.952567        1.021857        -1.191261
H         -2.302382        0.144077        -2.038979
S         -2.164783       -0.419837         0.259271
```

6_2ethylacrolein_6_am1

Datum	Value
AM1 Energy	-0.0478
AM1 Free Energy (Quasiharmonic)	0.044415
Number of Imaginary Frequencies	0

Frequencies (Top 3 out of 36)

```
1.      40.8091 cm-1
2.      80.1476 cm-1
3.     209.0824 cm-1
```

AM1 Molecular Geometry in Cartesian Coordinates

```
C          -1.402758       -0.627386       -0.000000
O          -2.505664       -0.075343        0.000001
C          -0.117929        0.102718       -0.000000
C          -0.091714        1.440871       -0.000000
C           1.086533       -0.771344        0.000000
C           2.397994       -0.029291        0.000000
H          -1.314319       -1.738210       -0.000002
H           0.840117        2.021436        0.000000
H          -1.021542        2.029980       -0.000001
H           1.041220       -1.440262        0.903904
H           1.041220       -1.440262       -0.903904
H           3.245373       -0.757435        0.000001
H           2.490240        0.617045       -0.906220
H           2.490240        0.617046        0.906220
```

6_2ethylacrolein_7_am1_HEI

Datum	Value
AM1 Energy	-0.116826
AM1 Free Energy (Quasiharmonic)	0.008927
Number of Imaginary Frequencies	0

Frequencies (Top 3 out of 51)

```
1.      35.6026 cm-1
2.      56.6112 cm-1
3.      63.4974 cm-1
```

AM1 Molecular Geometry in Cartesian Coordinates

```
C        -1.803685     1.164397     -0.200998
O        -1.498572     2.367340     -0.419470
C        -1.011970     0.172608      0.397504
C         0.342475     0.441247      0.794551
C        -1.574871    -1.174415      0.624380
C        -1.277526    -2.140376     -0.502694
H        -2.839229     0.834833     -0.486980
H         0.626279    -0.014735      1.773585
H         0.576841     1.531865      0.795645
H        -2.690689    -1.111784      0.756121
H        -1.161786    -1.605183      1.578794
H        -1.788695    -1.812873     -1.439328
H        -0.175348    -2.167349     -0.697817
H        -1.627524    -3.171386     -0.250885
C         3.073125     0.304016      0.091344
H         3.352514    -0.044981      1.110876
H         3.069277     1.417197      0.096030
H         3.838241    -0.052389     -0.633199
S         1.520213    -0.334175     -0.404476
```

6_2ethylacrolein_8_am1_HEI_reopt

Datum	Value
AM1 Energy	-0.122157
AM1 Free Energy (Quasiharmonic)	0.004061
Number of Imaginary Frequencies	0

Frequencies (Top 3 out of 51)

```
1.     31.0667 cm-1
2.     52.2730 cm-1
3.     74.3440 cm-1
```

AM1 Molecular Geometry in Cartesian Coordinates

```
C         1.016403     1.406437     -0.532190
O         1.933871     2.048889      0.044824
C         0.620172     0.080854     -0.290909
```

```
C          -0.459211      -0.509260      -1.029380
C           1.295200      -0.719035       0.752323
C           2.194450      -1.798372       0.189825
H           0.440532       1.937824      -1.336855
H          -0.360048      -1.614239      -1.150859
H          -0.635151      -0.027456      -2.019991
H           1.913604      -0.036080       1.397044
H           0.529269      -1.206433       1.418154
H           2.677623      -2.379867       1.012981
H           1.610326      -2.507769      -0.444487
H           2.995973      -1.344912      -0.440566
C          -2.012133       0.671401       1.104100
H          -2.302578       0.144316       2.038923
H          -0.952598       1.021891       1.191250
H          -2.681086       1.547807       0.960888
S          -2.164758      -0.419896      -0.259231
```

6_2ethylacrolein_9_am1_HEI

Datum	Value
AM1 Energy	-0.116771
AM1 Free Energy (Quasiharmonic)	0.0085
Number of Imaginary Frequencies	0

Frequencies (Top 3 out of 51)

```
1.      33.7046 cm-1
2.      44.4282 cm-1
3.      59.3733 cm-1
```

AM1 Molecular Geometry in Cartesian Coordinates

```
C           1.548439       1.385731      -0.214039
O           1.196497       2.516058       0.217737
C           0.896294       0.156835      -0.036292
C          -0.361634       0.069820       0.653425
C           1.499212      -1.080408      -0.573426
C           2.303080      -1.841612       0.460394
H           2.510393       1.331606      -0.792761
H          -0.607630       1.001924       1.213962
H          -0.448168      -0.818021       1.325391
H           2.175162      -0.841117      -1.440106
H           0.692169      -1.758536      -0.966133
H           3.148548      -1.216356       0.834431
```

```
H          1.657331      -2.109947       1.330669
H          2.722741      -2.782594       0.027525
C         -3.151641      -0.260935       0.408831
H         -3.107366      -1.122450       1.112635
H         -3.293235       0.666600       1.007881
H         -4.025900      -0.391504      -0.266521
S         -1.712408      -0.166541      -0.582639
```

7_transtrans24hexadienal_10_am1_HEI

Datum	Value
AM1 Energy	-0.081815
AM1 Free Energy (Quasiharmonic)	0.047273
Number of Imaginary Frequencies	0

Frequencies (Top 3 out of 54)

```
1.      56.9784 cm-1
2.      59.3386 cm-1
3.      79.6730 cm-1
```

AM1 Molecular Geometry in Cartesian Coordinates

```
C          2.704025      -0.589498       0.400566
C          1.504698      -0.634595      -0.321890
C          0.268508      -0.162043       0.215212
C         -0.945163      -0.820199      -0.312901
C         -2.029530      -1.096795       0.423465
C         -3.241320      -1.746982      -0.110183
O          3.830595      -0.992369       0.007058
H          2.635845      -0.168301       1.439109
H          1.522034      -1.007398      -1.350420
H          0.253648      -0.077432       1.329837
H         -0.904544      -1.079158      -1.384957
H         -2.065584      -0.847945       1.495707
H         -4.132321      -1.083614       0.027527
H         -3.142889      -1.976520      -1.199785
H         -3.439397      -2.705920       0.432055
C         -1.320919       2.306219       0.340049
H         -1.091192       2.791825       1.314850
H         -1.737516       3.074986      -0.347756
H         -2.088832       1.516469       0.512563
S          0.119012       1.622832      -0.383194
```

7_transtrans24hexadienal_11_am1_HEI_reopt

Datum	Value
AM1 Energy	-0.075392
AM1 Free Energy (Quasiharmonic)	0.053366
Number of Imaginary Frequencies	0

Frequencies (Top 3 out of 54)

```
1.      38.3333 cm-1
2.      61.0211 cm-1
3.      66.5779 cm-1
```

AM1 Molecular Geometry in Cartesian Coordinates

```
C         -2.354901       0.343284      -0.213504
C         -1.589019      -0.543017       0.550504
C         -0.181472      -0.397985       0.785587
C          0.343186       0.986786       0.705680
C          1.200649       1.463339      -0.200717
C          1.685905       2.856787      -0.221064
O         -3.592834       0.261158      -0.438032
H         -1.805335       1.221564      -0.645967
H         -2.072226      -1.436383       0.959241
H          0.137341      -0.835173       1.771255
H         -0.059796       1.645376       1.496709
H          1.574740       0.812884      -1.010909
H          2.803358       2.880374      -0.163205
H          1.279188       3.454147       0.631852
H          1.379101       3.356898      -1.174193
C          2.262829      -1.804062       0.003936
H          2.871334      -0.884620      -0.150692
H          2.671940      -2.617160      -0.635998
H          2.356898      -2.103918       1.071749
S          0.587691      -1.563378      -0.444257
```

7_transtrans24hexadienal_1_am1_HEI

Datum	Value
AM1 Energy	-0.085897

Datum	Value
AM1 Free Energy (Quasiharmonic)	0.043568
Number of Imaginary Frequencies	0

Frequencies (Top 3 out of 54)

```
1.      42.4177 cm-1
2.      51.4748 cm-1
3.      82.0654 cm-1
```

AM1 Molecular Geometry in Cartesian Coordinates

```
C          -2.146958       -1.342730       -0.351770
C          -1.010369       -0.891744        0.331276
C           0.062611       -0.217559       -0.322363
C           1.388378       -0.366303        0.312019
C           2.519573       -0.597696       -0.366608
C           3.842939       -0.743460        0.268274
O          -3.135623       -1.938195        0.152858
H          -2.161693       -1.154691       -1.458486
H          -0.949021       -1.053068        1.412205
H           0.089088       -0.368390       -1.429308
H           1.391521       -0.281853        1.411983
H           2.514076       -0.685162       -1.464293
H           3.781530       -0.653812        1.380840
H           4.285501       -1.741687        0.022202
H           4.542863        0.045456       -0.106843
C          -1.617235        2.065495        0.419292
H          -1.496314        2.627968        1.370690
H          -2.213061        2.680608       -0.289978
H          -2.159210        1.106709        0.624010
S          -0.048370        1.721716       -0.277287
```

7_transtrans24hexadienal_1_am1

Datum	Value
AM1 Energy	-0.022103
AM1 Free Energy (Quasiharmonic)	0.073558
Number of Imaginary Frequencies	0

Frequencies (Top 3 out of 39)

```
1.      50.6400 cm-1
2.      96.1091 cm-1
3.     116.1256 cm-1
```

AM1 Molecular Geometry in Cartesian Coordinates

```
C         -2.610090        0.339895       -0.000001
C         -1.304913       -0.328406       -0.000000
C         -0.150916        0.358426       -0.000001
C          1.148288       -0.278994       -0.000001
C          2.298098        0.412251        0.000001
C          3.631492       -0.217373        0.000000
O         -3.678919       -0.276279        0.000001
H         -2.587603        1.454418       -0.000003
H         -1.328159       -1.430737       -0.000000
H         -0.153602        1.464254       -0.000001
H          1.151295       -1.383055       -0.000003
H          2.297934        1.515999        0.000002
H          4.205480        0.103924        0.905999
H          3.568761       -1.333302       -0.000007
H          4.205486        0.103936       -0.905989
```

7_transtrans24hexadienal_2_am1_HEI

Datum	Value
AM1 Energy	-0.085897
AM1 Free Energy (Quasiharmonic)	0.043559
Number of Imaginary Frequencies	0

Frequencies (Top 3 out of 54)

```
1.      42.2303 cm-1
2.      51.6123 cm-1
3.      82.1985 cm-1
```

AM1 Molecular Geometry in Cartesian Coordinates

```
C         -2.148135       -1.342076       -0.351696
C         -1.011083       -0.891874        0.331245
C          0.062334       -0.218835       -0.322574
```

```
C          1.387906      -0.367400       0.311922
C          2.519217      -0.598695      -0.366586
C          3.842518      -0.744071       0.268457
O         -3.137061      -1.936918       0.153057
H         -2.162903      -1.153984      -1.458403
H         -0.949861      -1.052862       1.412233
H          0.088592      -0.369229      -1.429533
H          1.390934      -0.282743       1.411880
H          2.513833      -0.686266      -1.464269
H          4.542304       0.044983      -0.106654
H          3.780954      -0.654323       1.381015
H          4.285363      -1.742221       0.022540
C         -1.615439       2.066864       0.419387
H         -1.493775       2.629463       1.370625
H         -2.211060       2.682255      -0.289833
H         -2.158141       1.108576       0.624541
S         -0.047104       1.721764      -0.277470
```

7_transtrans24hexadienal_2_am1

Datum	Value
AM1 Energy	-0.022074
AM1 Free Energy (Quasiharmonic)	0.07348
Number of Imaginary Frequencies	0

Frequencies (Top 3 out of 39)

```
  1.      66.1883 cm-1
  2.      87.6745 cm-1
  3.     107.6312 cm-1
```

AM1 Molecular Geometry in Cartesian Coordinates

```
C          2.720489       0.299356      -0.000000
C          1.333915       0.769801      -0.000000
C          0.283354      -0.069352       0.000000
C         -1.091966       0.378647       0.000000
C         -2.131219      -0.470205      -0.000001
C         -3.541099      -0.038599      -0.000000
O          3.050650      -0.890959       0.000000
H          3.489570       1.105899      -0.000001
H          1.200326       1.862903      -0.000001
H          0.454358      -1.163198       0.000001
H         -1.253214       1.470685       0.000001
```

```
H          -1.971654       -1.562613       -0.000002
H          -4.062873       -0.439297        0.905965
H          -3.639687        1.074702        0.000001
H          -4.062874       -0.439294       -0.905965
```

7_transtrans24hexadienal_3_am1_HEI

Datum	Value
AM1 Energy	-0.084016
AM1 Free Energy (Quasiharmonic)	0.045272
Number of Imaginary Frequencies	0

Frequencies (Top 3 out of 54)

```
1.      31.4341 cm-1
2.      56.9131 cm-1
3.      77.1270 cm-1
```

AM1 Molecular Geometry in Cartesian Coordinates

```
C          -1.839051       -1.550589       -0.371570
C          -0.667043       -0.943108        0.098379
C           0.051864        0.023003       -0.658569
C           1.512909        0.132327       -0.473138
C           2.269513       -0.740344        0.201809
C           3.728394       -0.599658        0.373550
O          -2.537331       -2.402686        0.239001
H          -2.171395       -1.246688       -1.400283
H          -0.310803       -1.201765        1.100793
H          -0.210346        0.031620       -1.746734
H           1.970749        1.019568       -0.947907
H           1.817527       -1.627609        0.675316
H           4.124745        0.308978       -0.143226
H           3.983058       -0.520822        1.460675
H           4.256707       -1.495682       -0.039684
C          -1.793659        1.774343        0.814186
H          -1.558314        2.235614        1.798045
H          -2.653530        2.313142        0.359394
H          -2.068589        0.699275        0.967290
S          -0.403670        1.883126       -0.243974
```

7_transtrans24hexadienal_3_am1

Datum	Value
AM1 Energy	-0.022103
AM1 Free Energy (Quasiharmonic)	0.073542
Number of Imaginary Frequencies	0

Frequencies (Top 3 out of 39)

```
1.      49.8125 cm-1
2.      94.9446 cm-1
3.     115.7269 cm-1
```

AM1 Molecular Geometry in Cartesian Coordinates

```
C         -2.610022        0.339903       -0.000019
C         -1.304864       -0.328403        0.000029
C         -0.150869        0.358447        0.000070
C          1.148322       -0.278994        0.000109
C          2.298183        0.412192        0.000040
C          3.631168       -0.217319       -0.000084
O         -3.678827       -0.276279       -0.000077
H         -2.587561        1.454402        0.000001
H         -1.328120       -1.430696        0.000026
H         -0.153623        1.464239        0.000071
H          1.151426       -1.383015        0.000246
H          2.297869        1.515913       -0.000047
H          4.204904        0.103426       -0.906438
H          4.205684        0.104262        0.905469
H          3.568531       -1.333249        0.000423
```

7_transtrans24hexadienal_4_am1_HEI

Datum	Value
AM1 Energy	-0.079759
AM1 Free Energy (Quasiharmonic)	0.049279
Number of Imaginary Frequencies	0

Frequencies (Top 3 out of 54)

```
    1.      15.7915 cm-1
    2.      41.7560 cm-1
    3.      66.4766 cm-1
```

AM1 Molecular Geometry in Cartesian Coordinates

```
C          -2.135080       -1.467010        0.450345
C          -0.839986       -0.993331        0.647998
C          -0.080858       -0.319685       -0.371238
C           1.344824       -0.719633       -0.438925
C           2.363323       -0.118838        0.183738
C           3.763555       -0.577197        0.098418
O          -2.835413       -1.414422       -0.599857
H          -2.591895       -1.965251        1.348194
H          -0.375612       -1.117192        1.630528
H          -0.566913       -0.430896       -1.376456
H           1.516298       -1.621381       -1.054272
H           2.190662        0.780046        0.801628
H           4.410363        0.229074       -0.330809
H           4.152396       -0.824072        1.118490
H           3.867920       -1.486681       -0.543335
C          -1.674675        1.968223        0.237022
H          -2.246237        1.037332        0.477015
H          -1.682353        2.644965        1.119068
H          -2.161653        2.484738       -0.618849
S          -0.017268        1.559345       -0.163531
```

7_transtrans24hexadienal_4_am1

Datum	Value
AM1 Energy	-0.020978
AM1 Free Energy (Quasiharmonic)	0.073896
Number of Imaginary Frequencies	0

Frequencies (Top 3 out of 39)

```
    1.      18.3256 cm-1
    2.      87.9607 cm-1
    3.     103.5453 cm-1
```

AM1 Molecular Geometry in Cartesian Coordinates

```
C          2.523946       0.158452       0.012217
C          1.078045      -0.084206      -0.018522
C          0.187039       0.920653       0.009006
C         -1.250248       0.773324      -0.018008
C         -1.909696      -0.394739       0.024879
C         -3.379992      -0.502410      -0.002831
O          3.356441      -0.751932      -0.008061
H          2.839307       1.226733       0.055659
H          0.773413      -1.142325      -0.067154
H          0.541942       1.968314       0.053240
H         -1.812917       1.722693      -0.075433
H         -1.370184      -1.355611       0.086862
H         -3.742298      -1.017470       0.922951
H         -3.874490       0.497890      -0.066026
H         -3.700863      -1.111220      -0.886050
```

7_transtrans24hexadienal_5_am1_HEI

Datum	Value
AM1 Energy	-0.085897
AM1 Free Energy (Quasiharmonic)	0.043559
Number of Imaginary Frequencies	0

Frequencies (Top 3 out of 54)

```
1.      42.2768 cm-1
2.      51.4706 cm-1
3.      81.9709 cm-1
```

AM1 Molecular Geometry in Cartesian Coordinates

```
C          2.147504      -1.342576       0.351740
C          1.010846      -0.891652      -0.331301
C         -0.062433      -0.218259       0.322407
C         -1.388086      -0.366968      -0.312009
C         -2.519324      -0.598328       0.366597
C         -3.842671      -0.743833      -0.268330
O          3.136432      -1.937456      -0.153006
H          2.161957      -1.155099       1.458559
H          0.949875      -1.052238      -1.412367
H         -0.088816      -0.368940       1.429343
H         -1.391222      -0.282363      -1.411966
H         -2.513825      -0.685883       1.464280
```

```
H          -3.781199       -0.654190       -1.380890
H          -4.285440       -1.741972       -0.022276
H          -4.542462        0.045209        0.106765
C           1.616318        2.066286       -0.419076
H           2.158812        1.107789       -0.623597
H           1.495134        2.628559       -1.370556
H           2.211803        2.681779        0.290154
S           0.047563        1.721811        0.277276
```

7_transtrans24hexadienal_5_am1

Datum	Value
AM1 Energy	-0.020978
AM1 Free Energy (Quasiharmonic)	0.073896
Number of Imaginary Frequencies	0

Frequencies (Top 3 out of 39)

```
1.      18.3259 cm-1
2.      87.9607 cm-1
3.     103.5453 cm-1
```

AM1 Molecular Geometry in Cartesian Coordinates

```
C          -2.523946        0.158452        0.012217
C          -1.078045       -0.084206       -0.018522
C          -0.187039        0.920653        0.009006
C           1.250248        0.773324       -0.018008
C           1.909696       -0.394739        0.024879
C           3.379992       -0.502410       -0.002831
O          -3.356441       -0.751932       -0.008061
H          -2.839307        1.226733        0.055660
H          -0.773413       -1.142325       -0.067155
H          -0.541942        1.968314        0.053241
H           1.812917        1.722693       -0.075434
H           1.370184       -1.355611        0.086863
H           3.700862       -1.111220       -0.886050
H           3.874490        0.497890       -0.066026
H           3.742298       -1.017469        0.922951
```

7_transtrans24hexadienal_6_am1_HEI

Datum	Value
AM1 Energy	-0.083757
AM1 Free Energy (Quasiharmonic)	0.045837
Number of Imaginary Frequencies	0

Frequencies (Top 3 out of 54)

```
1.      37.6172 cm-1
2.      46.0539 cm-1
3.      64.9795 cm-1
```

AM1 Molecular Geometry in Cartesian Coordinates

```
C        -1.992730      -1.660480       0.344489
C        -0.909771      -0.902060       0.793626
C         0.005732      -0.235292      -0.082857
C         1.409554      -0.199397       0.381324
C         2.463722      -0.426932      -0.412080
C         3.863717      -0.396626       0.051921
O        -2.339286      -1.902748      -0.843113
H        -2.615008      -2.111458       1.164988
H        -0.753662      -0.802102       1.872388
H        -0.102301      -0.565169      -1.145443
H         1.541966       0.020737       1.454245
H         2.328096      -0.650592      -1.481999
H         4.356556      -1.384599      -0.132172
H         4.440579       0.384782      -0.504462
H         3.934535      -0.169772       1.144242
C        -1.983708       1.863272       0.136061
H        -2.423949       0.883629       0.450886
H        -2.106325       2.603491       0.956658
H        -2.519353       2.231616      -0.766094
S        -0.281620       1.657907      -0.221452
```

7_transtrans24hexadienal_6_am1

Datum	Value
AM1 Energy	-0.020743
AM1 Free Energy (Quasiharmonic)	0.074417
Number of Imaginary Frequencies	0

Frequencies (Top 3 out of 39)

```
1.      26.1641 cm-1
2.      85.6211 cm-1
3.     106.4550 cm-1
```

AM1 Molecular Geometry in Cartesian Coordinates

```
C          2.505440       -0.574456       -0.016743
C          1.041640       -0.547403       -0.059399
C          0.338380        0.597725       -0.009183
C         -1.101875        0.706690       -0.047614
C         -1.958328       -0.321472        0.052911
C         -3.424465       -0.169241        0.014783
O          3.217441        0.431664        0.062224
H          2.956989       -1.592322       -0.061429
H          0.552319       -1.530380       -0.139059
H          0.884071        1.559783        0.062336
H         -1.485831        1.736645       -0.162943
H         -1.597451       -1.356891        0.177348
H         -3.873257       -0.553392        0.965859
H         -3.733379        0.897041       -0.114899
H         -3.847741       -0.764859       -0.833535
```

7_transtrans24hexadienal_7_am1_HEI

Datum	Value
AM1 Energy	-0.075597
AM1 Free Energy (Quasiharmonic)	0.053444
Number of Imaginary Frequencies	0

Frequencies (Top 3 out of 54)

```
1.      40.7924 cm-1
2.      57.8590 cm-1
3.      71.7327 cm-1
```

AM1 Molecular Geometry in Cartesian Coordinates

```
C          2.688875      -0.943244      -0.303932
C          1.339658      -0.804787      -0.626717
C          0.385466      -0.171536       0.251033
C         -0.864430      -0.949526       0.435643
C         -2.038151      -0.733817      -0.164988
C         -3.235260      -1.571344       0.041030
O          3.269806      -0.639363       0.774674
H          3.314698      -1.412971      -1.110883
H          0.982701      -1.161571      -1.596334
H          0.834810       0.051937       1.255616
H         -0.738859      -1.801924       1.128305
H         -2.155936       0.106263      -0.871389
H         -3.047409      -2.396788       0.771245
H         -4.083369      -0.950840       0.426387
H         -3.557497      -2.030140      -0.927708
C         -0.917348       2.399504       0.542643
H         -0.440467       2.523473       1.540489
H         -1.065248       3.403580       0.086852
H         -1.910532       1.918069       0.690353
S          0.097237       1.469645      -0.539912
```

7_transtrans24hexadienal_7_am1

Datum	Value
AM1 Energy	-0.020743
AM1 Free Energy (Quasiharmonic)	0.074417
Number of Imaginary Frequencies	0

Frequencies (Top 3 out of 39)

```
1.      26.1642 cm-1
2.      85.6212 cm-1
3.     106.4552 cm-1
```

AM1 Molecular Geometry in Cartesian Coordinates

```
C         -2.505440      -0.574456      -0.016743
C         -1.041640      -0.547403      -0.059399
C         -0.338380       0.597725      -0.009183
C          1.101875       0.706690      -0.047614
C          1.958328      -0.321472       0.052911
C          3.424465      -0.169241       0.014783
O         -3.217441       0.431664       0.062224
```

```
H          -2.956989      -1.592322      -0.061429
H          -0.552319      -1.530380      -0.139059
H          -0.884071       1.559783       0.062336
H           1.485831       1.736645      -0.162943
H           1.597451      -1.356891       0.177348
H           3.733379       0.897041      -0.114899
H           3.873257      -0.553392       0.965859
H           3.847741      -0.764859      -0.833535
```

7_transtrans24hexadienal_8_am1_HEI

Datum	Value
AM1 Energy	-0.082232
AM1 Free Energy (Quasiharmonic)	0.047285
Number of Imaginary Frequencies	0

Frequencies (Top 3 out of 54)

```
1.      32.5298 cm-1
2.      51.8990 cm-1
3.      71.5754 cm-1
```

AM1 Molecular Geometry in Cartesian Coordinates

```
C           1.517769       1.894880       0.339757
C           0.483131       0.986600       0.572516
C          -0.001566       0.075094      -0.416405
C          -1.449836      -0.221597      -0.451633
C          -2.387944       0.482008       0.191543
C          -3.829912       0.173609       0.136415
O           2.161532       2.089467      -0.727286
H           1.785772       2.532005       1.226139
H           0.036687       0.948414       1.571218
H           0.383659       0.318985      -1.439739
H          -1.729187      -1.091439      -1.074402
H          -2.108310       1.351003       0.809698
H          -4.043332      -0.711723      -0.511963
H          -4.218216      -0.045082       1.163176
H          -4.399567       1.048182      -0.267793
C           2.216609      -1.537084       0.520421
H           2.377793      -0.463403       0.791709
H           2.293674      -2.164657       1.434965
H           3.000299      -1.853046      -0.202386
S           0.627436      -1.731627      -0.189876
```

7_transtrans24hexadienal_9_am1_HEI

Datum	Value
AM1 Energy	-0.082638
AM1 Free Energy (Quasiharmonic)	0.047454
Number of Imaginary Frequencies	0

Frequencies (Top 3 out of 54)

```
1.      39.3820 cm-1
2.      65.8771 cm-1
3.      67.5087 cm-1
```

AM1 Molecular Geometry in Cartesian Coordinates

```
C          1.199160        2.028680       -0.226344
C          1.003522        0.929072       -1.061860
C          0.087429       -0.156321       -0.879605
C         -1.095432        0.100088       -0.032953
C         -2.331773       -0.311950       -0.342600
C         -3.514057       -0.053381        0.500649
O          0.629976        2.323835        0.860235
H          1.983421        2.745131       -0.598264
H          1.645949        0.859256       -1.949124
H         -0.189386       -0.644547       -1.851093
H         -0.888965        0.691312        0.879686
H         -2.529304       -0.885372       -1.261553
H         -3.251360        0.547732        1.405809
H         -4.291114        0.507508       -0.077786
H         -3.969028       -1.017988        0.840926
C          2.190318       -1.092172        0.913147
H          1.984266       -1.257041        1.993307
H          3.123398       -1.630271        0.636816
H          2.324908        0.003018        0.728111
S          0.861400       -1.698344       -0.053208
```

Created using ESIgen v0.0.5

Ester Structures (AM1)

ESIgen is scientific software, funded by public research grants and published as:

```
J Rodriguez-Guerra, P Gomez-Orellana, JD Marechal.
J. Chem. Inf. Model., 2018, 58 (3), pp 561564.
DOI: 10.1021/acs.jcim.7b00714.
```

If you make use of ESIgen in scientific publications, please cite us in the main text! References only mentioned in SI documents are not indexed by citation engines.

1_methylacrylate_1_am1

Datum	Value
AM1 Energy	-0.111754
AM1 Free Energy (Quasiharmonic)	-0.042739
Number of Imaginary Frequencies	0

Frequencies (Top 3 out of 30)

```
  1.      64.7273 cm-1
  2.     105.8498 cm-1
  3.     156.2156 cm-1
```

AM1 Molecular Geometry in Cartesian Coordinates

```
C          2.514027        0.004176       -0.000243
C          1.341801       -0.630855       -0.000000
C          0.070446        0.101925        0.000162
O         -1.022280       -0.726364        0.000452
O         -0.128107        1.320526        0.000217
C         -2.302045       -0.093503       -0.000400
H         -3.019472       -0.950469       -0.000758
H         -2.416005        0.536895        0.913941
H         -2.415065        0.536712       -0.915028
H          2.589027        1.102064       -0.000315
H          3.468471       -0.540034       -0.000387
H          1.250761       -1.728916        0.000086
```

1_methylacrylate_1_reopt_am1_HEI

Datum	Value
AM1 Energy	-0.18556
AM1 Free Energy (Quasiharmonic)	-0.082671
Number of Imaginary Frequencies	0

Frequencies (Top 3 out of 45)

```
1.      32.4607 cm-1
2.      66.1314 cm-1
3.      87.1139 cm-1
```

AM1 Molecular Geometry in Cartesian Coordinates

```
C        0.966286      -0.850938      -0.878882
C        0.006820      -1.186344       0.123217
C       -1.236957      -0.573891       0.282595
O       -1.548541       0.431015      -0.653645
O       -2.150404      -0.777891       1.121036
C       -2.813348       1.042893      -0.512121
H       -2.905309       1.535872       0.486622
H       -2.854778       1.800851      -1.334037
H       -3.631742       0.290235      -0.625361
H        0.543991      -0.242816      -1.711556
H        1.515476      -1.736739      -1.282021
H        0.250755      -1.969749       0.847202
S        2.435938       0.145771      -0.296063
C        1.838430       1.065760       1.071433
H        0.818334       0.700884       1.351354
H        2.527947       0.932999       1.933282
H        1.784493       2.146243       0.814938
```

1_methylacrylate_2_am1_HEI

Datum	Value
AM1 Energy	-0.185964
AM1 Free Energy (Quasiharmonic)	-0.083141

Datum	Value
Number of Imaginary Frequencies	0

Frequencies (Top 3 out of 45)

```
1.      37.1627 cm-1
2.      62.1105 cm-1
3.      83.6437 cm-1
```

AM1 Molecular Geometry in Cartesian Coordinates

```
C          1.158592       -1.218346        0.017424
C         -0.029832       -0.764707       -0.633377
C         -1.148472       -0.332589        0.080666
O         -2.210579        0.096011       -0.744486
O         -1.407421       -0.263520        1.307020
C         -3.383962        0.512003       -0.079213
H         -4.088562        0.800986       -0.898968
H         -3.809131       -0.321047        0.532668
H         -3.173283        1.384192        0.587206
H          1.646025       -2.081437       -0.498096
H          1.005775       -1.441893        1.098796
H         -0.061419       -0.733409       -1.724950
S          2.598530       -0.031182       -0.006115
C          1.860868        1.559196        0.020158
H          2.029585        2.047141        1.004824
H          2.314401        2.186974       -0.777344
H          0.760970        1.464133       -0.160516
```

1_methylacrylate_2_am1

Datum	Value
AM1 Energy	-0.111084
AM1 Free Energy (Quasiharmonic)	-0.042211
Number of Imaginary Frequencies	0

Frequencies (Top 3 out of 30)

```
1.      53.5318 cm-1
2.     109.5252 cm-1
3.     150.1631 cm-1
```

AM1 Molecular Geometry in Cartesian Coordinates

```
C          -2.184665       -0.745815       -0.000034
C          -1.506473        0.400865       -0.000003
C          -0.042373        0.489976        0.000016
O           0.606468       -0.718736        0.000106
O           0.648891        1.512387       -0.000009
C           2.033678       -0.676146       -0.000057
H           2.399004       -0.150323       -0.914749
H           2.399145       -0.150130        0.914478
H           2.329894       -1.753868        0.000039
H          -1.688730       -1.727648       -0.000053
H          -3.283534       -0.768488       -0.000039
H          -1.999652        1.387969        0.000019
```

1_methylacrylate_3_reopt_am1_HEI

Datum	Value
AM1 Energy	-0.18556
AM1 Free Energy (Quasiharmonic)	-0.082672
Number of Imaginary Frequencies	0

Frequencies (Top 3 out of 45)

```
  1.      32.4206 cm-1
  2.      66.0983 cm-1
  3.      87.0716 cm-1
```

AM1 Molecular Geometry in Cartesian Coordinates

```
C           0.966359        0.851109        0.878887
C           0.006827        1.186533       -0.123115
C          -1.236894        0.573964       -0.282495
O          -1.548368       -0.431034        0.653679
O          -2.150397        0.777926       -1.120885
C          -2.813064       -1.043116        0.512032
H          -2.904625       -1.536560       -0.486516
H          -2.854669       -1.800707        1.334273
H          -3.631590       -0.290510        0.624655
H           0.544052        0.243219        1.711732
H           1.515720        1.736891        1.281816
```

```
H         0.250666       1.970018       -0.847049
S         2.435696      -0.146162        0.296221
C         1.838276      -1.065274       -1.071895
H         2.527809      -0.931980       -1.933651
H         1.784403      -2.145894       -0.815963
H         0.818189      -0.700327       -1.351676
```

1_methylacrylate_4_am1_HEI

Datum	Value
AM1 Energy	-0.185964
AM1 Free Energy (Quasiharmonic)	-0.083142
Number of Imaginary Frequencies	0

Frequencies (Top 3 out of 45)

```
 1.      37.1569 cm-1
 2.      62.0973 cm-1
 3.      83.6094 cm-1
```

AM1 Molecular Geometry in Cartesian Coordinates

```
C        -1.158598      -1.218340       -0.016949
C         0.029803      -0.764359        0.633659
C         1.148487      -0.332626       -0.080550
O         2.210529       0.096447        0.744440
O         1.407525      -0.264249       -1.306921
C         3.383971       0.512036        0.079019
H         4.088539       0.801409        0.898657
H         3.809136      -0.321345       -0.532407
H         3.173374       1.383889       -0.587859
H        -1.645997      -2.081200        0.498980
H        -1.005760      -1.442422       -1.098202
H         0.061347      -0.732514        1.725217
S        -2.598549      -0.031190        0.005945
C        -1.860877       1.559192       -0.020463
H        -0.761051       1.464193        0.160667
H        -2.029195       2.046878       -1.005321
H        -2.314753       2.187145        0.776699
```

1_methylacrylate_5_reopt3_am1_HEI

Datum	Value
AM1 Energy	-0.181937
AM1 Free Energy (Quasiharmonic)	-0.078611
Number of Imaginary Frequencies	0

Frequencies (Top 3 out of 45)

```
1.      25.6951 cm-1
2.      60.5611 cm-1
3.      81.9644 cm-1
```

AM1 Molecular Geometry in Cartesian Coordinates

```
C         -0.973005        0.501776       -1.118016
C          0.296965       -0.066918       -0.784496
C          1.210722        0.602727        0.032707
O          2.459395        0.024083        0.354581
O          1.162433        1.726668        0.586388
C          2.739852       -1.251413       -0.167008
H          2.000764       -2.008845        0.195561
H          3.762323       -1.503391        0.216186
H          2.745948       -1.238962       -1.285575
H         -1.030508        1.593180       -0.898060
H         -1.292200        0.294957       -2.168741
H          0.522781       -1.061846       -1.172603
S         -2.439138       -0.232708       -0.234213
C         -1.854035       -0.541056        1.390385
H         -2.311945        0.174128        2.107992
H         -2.126498       -1.576820        1.688218
H         -0.742077       -0.425780        1.415237
```

1_methylacrylate_6_am1_HEI

Datum	Value
AM1 Energy	-0.181676
AM1 Free Energy (Quasiharmonic)	-0.079574
Number of Imaginary Frequencies	0

Frequencies (Top 3 out of 45)

```
   1.      39.5511 cm-1
   2.      59.4888 cm-1
   3.      61.0891 cm-1
```

AM1 Molecular Geometry in Cartesian Coordinates

```
C          -1.011505        0.096091        0.864191
C           0.241428       -0.559519        0.659366
C           1.364904        0.129065        0.196043
O           2.493500       -0.697879        0.011127
O           1.584937        1.339954       -0.049205
C           3.669746       -0.052880       -0.427568
H           3.501824        0.453741       -1.409492
H           4.423747       -0.873446       -0.528551
H           4.008542        0.704596        0.321371
H          -1.547349       -0.234031        1.786760
H          -0.943758        1.208498        0.841947
H           0.315614       -1.636255        0.826341
S          -2.131986       -0.402491       -0.512290
C          -3.649183        0.388174       -0.144889
H          -3.539070        1.495815       -0.135188
H          -4.383373        0.106342       -0.931707
H          -4.044247        0.072405        0.846915
```

1_methylacrylate_7_am1_HEI

Datum	Value
AM1 Energy	-0.181188
AM1 Free Energy (Quasiharmonic)	-0.079046
Number of Imaginary Frequencies	0

Frequencies (Top 3 out of 45)

```
   1.      40.1008 cm-1
   2.      57.4743 cm-1
   3.      67.7840 cm-1
```

AM1 Molecular Geometry in Cartesian Coordinates

```
C          -0.860618      -0.293920       0.831783
C           0.242580      -1.085533       0.391821
C           1.498081      -0.583179       0.043821
O           1.671764       0.803641       0.219021
O           2.530788      -1.168740      -0.366661
C           2.936602       1.321398      -0.135409
H           2.851483       2.423158       0.040117
H           3.738035       0.879679       0.506521
H           3.166972       1.107365      -1.207725
H          -0.588106       0.747704       1.118188
H          -1.455230      -0.766714       1.650873
H           0.105166      -2.164032       0.270564
S          -2.050943      -0.165574      -0.572055
C          -3.401314       0.733052       0.084289
H          -3.097248       1.758351       0.393215
H          -4.179513       0.811812      -0.706772
H          -3.838865       0.221739       0.971192
```

1_methylacrylate_8_am1_HEI

Datum	Value
AM1 Energy	-0.181937
AM1 Free Energy (Quasiharmonic)	-0.078613
Number of Imaginary Frequencies	0

Frequencies (Top 3 out of 45)

```
  1.      25.6653 cm-1
  2.      60.5208 cm-1
  3.      81.9234 cm-1
```

AM1 Molecular Geometry in Cartesian Coordinates

```
C          -0.973019      -0.504592       1.116756
C           0.296886       0.065036       0.784506
C           1.210834      -0.602734      -0.034025
O           2.459338      -0.023074      -0.354714
O           1.162852      -1.725559      -0.589961
C           2.739630       1.251239       0.169850
H           2.000507       2.009425      -0.191057
H           3.762109       1.504201      -0.212650
H           2.745605       1.236223       1.288379
H          -1.030361      -1.595501       0.894372
```

```
H          -1.292279       -0.300165        2.167923
H           0.522496        1.059196        1.174690
S          -2.439237        0.231789        0.234610
C          -1.853950        0.544995       -1.388994
H          -0.741899        0.430575       -1.413826
H          -2.311108       -0.168550       -2.108700
H          -2.127092        1.581370       -1.684043
```

2_tert-butylacrylate_1_am1

Datum	Value
AM1 Energy	-0.128603
AM1 Free Energy (Quasiharmonic)	0.019497
Number of Imaginary Frequencies	0

Frequencies (Top 3 out of 57)

```
1.      50.0712 cm-1
2.      64.2355 cm-1
3.      95.0063 cm-1
```

AM1 Molecular Geometry in Cartesian Coordinates

```
C           3.499667        0.174956       -0.000001
C           2.289815        0.734049        0.000000
C           1.064378       -0.082790       -0.000000
O          -0.059596        0.690086        0.000002
O           0.976581       -1.314219       -0.000002
C          -1.376447        0.083455       -0.000000
C          -2.312205        1.291543        0.000111
C          -1.586020       -0.745610       -1.256449
C          -1.585952       -0.745792        1.256339
H           3.643380       -0.916060       -0.000002
H           4.417989        0.777979       -0.000001
H           2.128587        1.823763        0.000001
H          -2.656021       -1.054840       -1.322444
H          -1.324800       -0.144627       -2.159757
H          -0.944778       -1.659697       -1.233612
H          -3.368647        0.934785        0.000121
H          -2.132804        1.916309        0.906609
H          -2.132861        1.916431       -0.906314
H          -2.655936       -1.055083        1.322317
H          -0.944664       -1.659844        1.233359
H          -1.324738       -0.144921        2.159724
```

2_tertbutylacrylate_1_reopt_am1_HEI_reopt

Datum	Value
AM1 Energy	-0.200538
AM1 Free Energy (Quasiharmonic)	-0.018109
Number of Imaginary Frequencies	0

Frequencies (Top 3 out of 72)

```
1.      24.8114 cm-1
2.      28.7396 cm-1
3.      59.8356 cm-1
```

AM1 Molecular Geometry in Cartesian Coordinates

```
C          1.896407        0.486424       -1.143991
C          1.124029        1.344700       -0.304913
C         -0.191688        1.108759        0.104339
O         -0.759453       -0.071605       -0.400842
O         -0.938694        1.800948        0.839512
C         -2.105984       -0.425628       -0.087549
C         -2.321025       -1.751289       -0.826689
C         -2.296970       -0.647169        1.407616
C         -3.096264        0.607917       -0.609264
H          1.285708       -0.270734       -1.687277
H          2.555982        1.039570       -1.856742
H          1.583160        2.261082        0.078638
H         -3.309750       -1.072219        1.603046
H         -1.523495       -1.356656        1.785532
H         -2.188676        0.322501        1.951228
H         -3.355832       -2.121032       -0.639222
H         -1.585830       -2.509191       -0.468303
H         -2.173559       -1.604075       -1.922053
H         -4.139356        0.232896       -0.483257
H         -2.974203        1.566015       -0.048238
H         -2.905648        0.801146       -1.691260
C          2.593788       -0.763850        1.343521
H          2.318122       -1.829502        1.500250
H          3.379703       -0.482284        2.077646
H          1.691098       -0.121504        1.500161
S          3.207125       -0.528121       -0.281745
```

2_tert-butylacrylate_2_am1

Datum	Value
AM1 Energy	-0.127898
AM1 Free Energy (Quasiharmonic)	0.020043
Number of Imaginary Frequencies	0

Frequencies (Top 3 out of 57)

```
1.      36.1412 cm-1
2.      70.9276 cm-1
3.      83.1618 cm-1
```

AM1 Molecular Geometry in Cartesian Coordinates

```
C         -2.873458        1.138045       -0.000000
C         -2.485984       -0.136136        0.000001
C         -1.081137       -0.575533       -0.000000
O         -0.184328        0.452685       -0.000005
O         -0.686293       -1.744841        0.000001
C          1.242180        0.192142        0.000000
C          1.848884        1.594670       -0.000093
C          1.651115       -0.558700        1.256537
C          1.651102       -0.558863       -1.256443
H         -2.159244        1.974451       -0.000002
H         -3.935599        1.420472        0.000001
H         -3.198913       -0.978228        0.000003
H          2.764400       -0.591233        1.323190
H          1.247649       -0.042208        2.159757
H          1.258224       -1.603903        1.233259
H          2.960841        1.511113       -0.000100
H          1.520576        2.155439       -0.906714
H          1.520597        2.155549        0.906468
H          2.764387       -0.591452       -1.323076
H          1.258164       -1.604046       -1.233047
H          1.247671       -0.042460       -2.159729
```

2_tertbutylacrylate_2_reopt_am1_HEI_reopt

Datum	Value
AM1 Energy	-0.201033

Datum	Value
AM1 Free Energy (Quasiharmonic)	-0.018611
Number of Imaginary Frequencies	0

Frequencies (Top 3 out of 72)

```
1.      25.8607 cm-1
2.      33.2063 cm-1
3.      61.1148 cm-1
```

AM1 Molecular Geometry in Cartesian Coordinates

```
C         -2.262422       -1.210975        0.147332
C         -1.025059       -0.784787        0.721445
C          0.104376       -0.525978       -0.061000
O          1.198480       -0.087199        0.707900
O          0.310829       -0.626865       -1.294496
C          2.462789        0.163242        0.097178
C          3.034491       -1.090575       -0.552693
C          2.385081        1.303238       -0.910746
C          3.362931        0.582269        1.265349
H         -2.156666       -1.562165       -0.905436
H         -2.799082       -1.972371        0.764297
H         -0.961584       -0.634272        1.801520
H          3.409641        1.573570       -1.259655
H          1.762965        0.993197       -1.785190
H          1.913495        2.195731       -0.435954
H          2.409221       -1.379416       -1.432108
H          4.082178       -0.902509       -0.886611
H          3.029755       -1.931562        0.180164
H          4.387381        0.806408        0.887253
H          2.944873        1.489483        1.760757
H          3.417649       -0.239564        2.016883
C         -2.750025        1.598083       -0.135203
H         -2.903116        1.993630       -1.162938
H         -3.136846        2.338034        0.598734
H         -1.656765        1.436965        0.038814
S         -3.605659        0.081265        0.067642
```

2_tertbutylacrylate_3_am1_HEI

Datum	Value
AM1 Energy	-0.200538

Datum	Value
AM1 Free Energy (Quasiharmonic)	-0.018108
Number of Imaginary Frequencies	0

Frequencies (Top 3 out of 72)

```
1.      24.8366 cm-1
2.      28.7355 cm-1
3.      59.8376 cm-1
```

AM1 Molecular Geometry in Cartesian Coordinates

```
C        -1.896364       0.486231      -1.144049
C        -1.124005       1.344587      -0.305030
C         0.191705       1.108689       0.104264
O         0.759499      -0.071711      -0.400803
O         0.938691       1.800935       0.839404
C         2.106059      -0.425623      -0.087503
C         2.321159      -1.751359      -0.826487
C         3.096253       0.607928      -0.609373
C         2.297108      -0.646966       1.407682
H        -2.555860       1.039304      -1.856932
H        -1.285655      -0.271027      -1.687185
H        -1.583147       2.261003       0.078427
H         4.139379       0.233050      -0.483234
H         2.905674       0.800923      -1.691417
H         2.974049       1.566120      -0.048540
H         1.586064      -2.509281      -0.467941
H         2.173588      -1.604303      -1.921858
H         3.356016      -2.120982      -0.639059
H         3.309897      -1.071987       1.603128
H         2.188833       0.322776       1.951170
H         1.523650      -1.356401       1.785726
C        -2.594058      -0.763621       1.343614
H        -1.691510      -0.121096       1.500336
H        -3.380148      -0.482145       2.077585
H        -2.318214      -1.829207       1.500474
S        -3.207204      -0.528108      -0.281762
```

2_tert-butylacrylate_3_am1

Datum	Value
AM1 Energy	-0.117824

Datum	Value
AM1 Free Energy (Quasiharmonic)	0.030767
Number of Imaginary Frequencies	0

Frequencies (Top 3 out of 57)

```
1.      39.2989 cm-1
2.      48.4828 cm-1
3.     109.8983 cm-1
```

AM1 Molecular Geometry in Cartesian Coordinates

```
C         -2.881558       -0.931187       -0.188380
C         -1.650562       -0.663990        0.250243
C         -1.068126        0.679260        0.129335
O          0.278750        0.926468        0.138748
O         -1.710063        1.733089        0.048771
C          1.269023       -0.108975       -0.026874
C          1.349536       -0.995600        1.209627
C          1.024003       -0.912899       -1.293756
C          2.564692        0.695379       -0.177793
H         -3.509987       -0.169806       -0.674667
H         -3.333475       -1.927188       -0.085347
H         -1.032091       -1.423270        0.752467
H          0.091442       -1.520411       -1.216205
H          0.928979       -0.224750       -2.168138
H          1.884468       -1.602296       -1.467397
H          2.413596       -1.122282        1.525096
H          0.787982       -0.529455        2.055199
H          0.931299       -2.011307        1.009672
H          3.413821       -0.004447       -0.356411
H          2.476817        1.400707       -1.037764
H          2.755606        1.286116        0.748935
```

2_tertbutylacrylate_4_reopt_am1_HEI

Datum	Value
AM1 Energy	-0.201033
AM1 Free Energy (Quasiharmonic)	-0.018611
Number of Imaginary Frequencies	0

Frequencies (Top 3 out of 72)

```
1.      25.8647 cm-1
2.      33.2002 cm-1
3.      61.1074 cm-1
```

AM1 Molecular Geometry in Cartesian Coordinates

```
C          2.262438       -1.210985        0.147318
C          1.025091       -0.784786        0.721456
C         -0.104391       -0.526077       -0.060957
O         -1.198479       -0.087255        0.707953
O         -0.310928       -0.627137       -1.294423
C         -2.462776        0.163276        0.097251
C         -3.362830        0.582503        1.265421
C         -2.384982        1.303184       -0.910766
C         -3.034652       -1.090527       -0.552492
H          2.799154       -1.972300        0.764336
H          2.156637       -1.562289       -0.905411
H          0.961670       -0.634174        1.801521
H         -3.409533        1.573635       -1.259614
H         -1.913230        2.195646       -0.436077
H         -1.762981        0.992996       -1.785241
H         -4.387271        0.806724        0.887344
H         -3.417611       -0.239263        2.017026
H         -2.944652        1.489713        1.760737
H         -2.409486       -1.379495       -1.431940
H         -3.029938       -1.931465        0.180424
H         -4.082351       -0.902381       -0.886337
C          2.750061        1.598144       -0.135207
H          1.656832        1.437132        0.039107
H          3.137149        2.338049        0.598639
H          2.902897        1.993705       -1.162977
S          3.605638        0.081282        0.067379
```

2_tertbutylacrylate_5_am1_HEI

Datum	Value
AM1 Energy	-0.200538
AM1 Free Energy (Quasiharmonic)	-0.018108
Number of Imaginary Frequencies	0

Frequencies (Top 3 out of 72)

```
   1.      24.8237 cm-1
   2.      28.7549 cm-1
   3.      59.8525 cm-1
```

AM1 Molecular Geometry in Cartesian Coordinates

```
 C         -1.896386       0.486194       1.144075
 C         -1.124038       1.344597       0.305097
 C          0.191661       1.108715      -0.104249
 O          0.759452      -0.071715       0.400747
 O          0.938623       1.801002      -0.839374
 C          2.106025      -0.425616       0.087499
 C          2.321107      -1.751335       0.826524
 C          2.297103      -0.646999      -1.407677
 C          3.096221       0.607932       0.609371
 H         -1.285683      -0.271086       1.687186
 H         -2.555893       1.039243       1.856965
 H         -1.583187       2.261034      -0.078301
 H          3.309974      -1.071830      -1.603111
 H          1.523781      -1.356610      -1.785676
 H          2.188631       0.322696      -1.951214
 H          3.355923      -2.121035       0.639015
 H          1.585928      -2.509226       0.468082
 H          2.173649      -1.604216       1.921903
 H          4.139333       0.232904       0.483545
 H          2.974265       1.566029       0.048322
 H          2.905419       0.801170       1.691334
 C         -2.593861      -0.763557      -1.343632
 H         -1.691147      -0.121220      -1.500124
 H         -2.318247      -1.829186      -1.500607
 H         -3.379779      -0.481777      -2.077672
 S         -3.207160      -0.528174       0.281706
```

2_tertbutylacrylate_6_am1_HEI

Datum	Value
AM1 Energy	-0.201033
AM1 Free Energy (Quasiharmonic)	-0.018611
Number of Imaginary Frequencies	0

Frequencies (Top 3 out of 72)

```
1.      25.8432 cm-1
2.      33.1992 cm-1
3.      61.1080 cm-1
```

AM1 Molecular Geometry in Cartesian Coordinates

```
C          -2.262423       -1.210937        0.147190
C          -1.025104       -0.784705        0.721354
C           0.104364       -0.525881       -0.061031
O           1.198391       -0.086954        0.707905
O           0.310898       -0.626814       -1.294509
C           2.462798        0.163225        0.097280
C           2.385325        1.303070       -0.910833
C           3.362873        0.582327        1.265475
C           3.034419       -1.090762       -0.552334
H          -2.156616       -1.562130       -0.905573
H          -2.799060       -1.972372        0.764131
H          -0.961688       -0.634190        1.801431
H           4.387356        0.806399        0.887433
H           2.944811        1.489598        1.760773
H           3.417512       -0.239439        2.017085
H           3.409933        1.573095       -1.259831
H           1.763094        0.993027       -1.785194
H           1.913976        2.195758       -0.436174
H           2.409328       -1.379555       -1.431890
H           4.082232       -0.902935       -0.885990
H           3.029321       -1.931684        0.180594
C          -2.750019        1.598056       -0.135162
H          -1.656723        1.436838        0.038538
H          -2.903344        1.993905       -1.162744
H          -3.136618        2.337811        0.599087
S          -3.605701        0.081228        0.067470
```

2_tertbutylacrylate_7_am1_HEI

Datum	Value
AM1 Energy	-0.196262
AM1 Free Energy (Quasiharmonic)	-0.014577
Number of Imaginary Frequencies	0

Frequencies (Top 3 out of 72)

```
  1.      26.1312 cm-1
  2.      37.7033 cm-1
  3.      48.6430 cm-1
```

AM1 Molecular Geometry in Cartesian Coordinates

```
C          -1.828256        0.422325       -0.848580
C          -0.902934        1.405988       -0.386672
C           0.439718        1.166025       -0.076793
O           0.876188       -0.142676       -0.339237
O           1.316768        1.955139        0.352734
C           2.198946       -0.551417        0.005798
C           2.236272       -2.038119       -0.366443
C           3.247748        0.196876       -0.807754
C           2.465730       -0.400250        1.497928
H          -2.528209        0.797483       -1.634295
H          -1.353722       -0.525963       -1.190079
H          -1.257640        2.426573       -0.214626
H           4.256181       -0.244856       -0.627234
H           3.006360        0.123716       -1.894358
H           3.254453        1.274759       -0.515013
H           3.241011       -2.458737       -0.129172
H           1.457244       -2.592003        0.207760
H           2.031341       -2.163071       -1.455287
H           3.441937       -0.872271        1.760141
H           2.490267        0.682678        1.770482
H           1.651086       -0.894868        2.077837
C          -4.080168       -1.144288       -0.111807
H          -4.804276       -1.418918        0.686827
H          -4.637197       -0.696855       -0.965611
H          -3.577485       -2.068628       -0.475149
S          -2.929833       -0.013223        0.565984
```

2_tertbutylacrylate_8_reopt_am1_HEI

Datum	Value
AM1 Energy	-0.196801
AM1 Free Energy (Quasiharmonic)	-0.01516
Number of Imaginary Frequencies	0

Frequencies (Top 3 out of 72)

```
   1.      27.3546 cm-1
   2.      39.1447 cm-1
   3.      46.5256 cm-1
```

AM1 Molecular Geometry in Cartesian Coordinates

```
C          -2.140516       -0.398273        0.815280
C          -0.864114        0.235867        0.919050
C           0.268421       -0.285666        0.284043
O           1.406439        0.519526        0.475289
O           0.449949       -1.339045       -0.372323
C           2.662803        0.148236       -0.088085
C           3.607877        1.279448        0.333622
C           3.170594       -1.169304        0.484628
C           2.601845        0.085550       -1.609107
H          -2.095297       -1.429244        0.393728
H          -2.719807       -0.402666        1.770075
H          -0.775695        1.174593        1.469917
H           4.217198       -1.354054        0.145340
H           3.149482       -1.129922        1.599229
H           2.514893       -2.007002        0.144147
H           4.629339        1.083663       -0.067627
H           3.235024        2.252826       -0.062335
H           3.653404        1.342308        1.445897
H           2.178970        1.037146       -2.009191
H           3.625861       -0.064037       -2.025687
H           1.942543       -0.758474       -1.926687
C          -4.721370       -0.199816       -0.349590
H          -5.165842       -0.248565        0.669974
H          -5.403417        0.376578       -1.013020
H          -4.634773       -1.239438       -0.737877
S          -3.169764        0.609136       -0.334916
```

3_methylcrotonate_1_am1_HEI

Datum	Value
AM1 Energy	-0.192289
AM1 Free Energy (Quasiharmonic)	-0.06335
Number of Imaginary Frequencies	0

Frequencies (Top 3 out of 54)

```
1.      38.9290 cm-1
2.      60.5975 cm-1
3.      70.6560 cm-1
```

AM1 Molecular Geometry in Cartesian Coordinates

```
C          -1.096570       -0.810800        0.274709
C           0.123937       -0.660346       -0.459531
C           1.308804       -0.247632        0.152275
O           2.392673       -0.132830       -0.744206
O           1.608774        0.025654        1.340121
C           3.628335        0.247904       -0.177477
H           3.960871       -0.498114        0.585397
H           4.339988        0.276429       -1.040405
H           3.549868        1.253310        0.304355
H          -0.932006       -0.802672        1.380959
C          -1.953402       -1.974383       -0.149070
H           0.127237       -0.853856       -1.534645
H          -1.467242       -2.931446        0.163906
H          -2.964719       -1.919834        0.324339
H          -2.079779       -1.991009       -1.258204
S          -2.331007        0.616093        0.047414
C          -1.329317        2.025876       -0.234111
H          -1.694960        2.562792       -1.136406
H          -1.376660        2.712384        0.639291
H          -0.268779        1.708220       -0.395309
```

3_methylcrotonate_1_am1

Datum	Value
AM1 Energy	-0.128575
AM1 Free Energy (Quasiharmonic)	-0.033628
Number of Imaginary Frequencies	0

Frequencies (Top 3 out of 39)

```
1.      59.4071 cm-1
2.      99.5100 cm-1
3.     112.7452 cm-1
```

AM1 Molecular Geometry in Cartesian Coordinates

```
C           1.902525        0.303453       -0.000015
C           0.794302       -0.448878        0.000017
C          -0.537398        0.160697        0.000029
O          -1.549133       -0.765943        0.000076
O          -0.850799        1.355577        0.000030
C          -2.881605       -0.254078       -0.000077
H          -3.517521       -1.173306       -0.000230
H          -3.053281        0.363013        0.914482
H          -3.053091        0.363171       -0.914568
H           1.830572        1.407125       -0.000033
C           3.267256       -0.253866       -0.000028
H           0.813325       -1.549994        0.000037
H           3.821351        0.100260        0.906285
H           3.266291       -1.371544       -0.000009
H           3.821322        0.100229       -0.906370
```

3_methylcrotonate_2_am1_HEI

Datum	Value
AM1 Energy	-0.191898
AM1 Free Energy (Quasiharmonic)	-0.062883
Number of Imaginary Frequencies	0

Frequencies (Top 3 out of 54)

```
1.      34.0248 cm-1
2.      57.1702 cm-1
3.      82.5991 cm-1
```

AM1 Molecular Geometry in Cartesian Coordinates

```
C          -0.943833        0.788245       -0.319088
C           0.055516        0.768913        0.704313
C           1.376802        0.355245        0.527312
O           1.727383       -0.034524       -0.779478
O           2.329079        0.285033        1.343953
C           3.067847       -0.435990       -0.970816
H           3.313279       -1.315966       -0.327129
H           3.129990       -0.707151       -2.054472
H           3.769121        0.400356       -0.730397
H          -0.515055        0.682611       -1.346001
C          -1.908352        1.942600       -0.242912
H          -0.218787        1.080077        1.716882
```

```
H          -2.767289       1.793407      -0.942706
H          -2.308846       2.057307       0.792869
H          -1.383337       2.889116      -0.523731
S          -2.162992      -0.672887      -0.258580
C          -1.259215      -1.977158       0.484041
H          -0.284343      -1.587459       0.871405
H          -1.847534      -2.400727       1.327008
H          -1.063630      -2.780589      -0.259352
```

3_methylcrotonate_2_am1

Datum	Value
AM1 Energy	-0.127879
AM1 Free Energy (Quasiharmonic)	-0.033089
Number of Imaginary Frequencies	0

Frequencies (Top 3 out of 39)

```
  1.      53.0971 cm-1
  2.      99.4099 cm-1
  3.     114.0481 cm-1
```

AM1 Molecular Geometry in Cartesian Coordinates

```
C           1.681962      -0.405366      -0.000021
C           0.857360       0.648921       0.000030
C          -0.602627       0.541447      -0.000014
O          -1.086497      -0.743383      -0.000090
O          -1.425428       1.461883      -0.000023
C          -2.506492      -0.889427       0.000069
H          -2.658429      -1.996757       0.000410
H          -2.938326      -0.416072       0.914491
H          -2.938374      -0.416615      -0.914608
H           1.281420      -1.435687      -0.000100
C           3.152083      -0.289028       0.000021
H           1.219551       1.690737       0.000107
H           3.572554      -0.794451      -0.906316
H           3.490778       0.776134       0.000103
H           3.572512      -0.794576       0.906307
```

3_methylcrotonate_3_am1_HEI

Datum	Value
AM1 Energy	-0.188816
AM1 Free Energy (Quasiharmonic)	-0.05945
Number of Imaginary Frequencies	0

Frequencies (Top 3 out of 54)

```
1.      33.5335 cm-1
2.      47.1911 cm-1
3.      83.8195 cm-1
```

AM1 Molecular Geometry in Cartesian Coordinates

```
C         0.979197       0.741333       0.778790
C        -0.044867      -0.179327       1.156489
C        -1.305701      -0.376980       0.589724
O        -1.669842       0.459316      -0.479986
O        -2.203468      -1.199686       0.903266
C        -2.950129       0.238064      -1.036335
H        -3.747378       0.404729      -0.271234
H        -3.030813       0.986558      -1.863891
H        -3.034736      -0.803098      -1.433121
H         1.587005       1.064184       1.665770
C         0.584695       1.932098      -0.046635
H         0.184381      -0.857113       1.989031
H         1.453108       2.620456      -0.189614
H         0.213383       1.612282      -1.050601
H        -0.239638       2.490328       0.462734
S         2.417204      -0.061623      -0.190799
C         1.752630      -1.532012      -0.873516
H         2.395883      -2.393472      -0.590037
H         0.717728      -1.697411      -0.481212
H         1.717342      -1.457574      -1.982383
```

3_methylcrotonate_3_am1

Datum	Value
AM1 Energy	-0.120201
AM1 Free Energy (Quasiharmonic)	-0.025379
Number of Imaginary Frequencies	0

Frequencies (Top 3 out of 39)

```
1.      17.1486 cm-1
2.     104.7923 cm-1
3.     109.9726 cm-1
```

AM1 Molecular Geometry in Cartesian Coordinates

```
C          1.760311        0.237067       -0.000056
C          0.521428       -0.276064        0.000079
C         -0.653980        0.598720        0.000027
O         -1.932928        0.091934        0.000006
O         -0.657252        1.833757        0.000008
C         -2.107036       -1.317291       -0.000045
H         -3.219777       -1.446734       -0.000222
H         -1.662681       -1.770835       -0.919118
H         -1.662970       -1.770844        0.919164
H          1.909166        1.333724       -0.000216
C          2.986689       -0.580329       -0.000004
H          0.347682       -1.362314        0.000244
H          3.600221       -0.342576        0.906195
H          2.765174       -1.675765        0.000137
H          3.600154       -0.342796       -0.906306
```

3_methylcrotonate_4_am1_HEI

Datum	Value
AM1 Energy	-0.187792
AM1 Free Energy (Quasiharmonic)	-0.059195
Number of Imaginary Frequencies	0

Frequencies (Top 3 out of 54)

```
1.      42.8879 cm-1
2.      58.3373 cm-1
3.      65.3842 cm-1
```

AM1 Molecular Geometry in Cartesian Coordinates

```
C          0.889370      0.607174      0.280483
C         -0.375817      0.632187     -0.396591
C         -1.519058      0.061678      0.167331
O         -2.652535      0.132728     -0.670296
O         -1.751776     -0.462194      1.283663
C         -3.846528     -0.405797     -0.144296
H         -4.601873     -0.272687     -0.959014
H         -3.718155     -1.487831      0.104099
H         -4.157560      0.146369      0.776488
H          0.803350      0.292070      1.350696
C          1.688894      1.878703      0.159888
H         -0.438822      1.069468     -1.395051
H          2.706707      1.755047      0.604907
H          1.801840      2.175826     -0.910219
H          1.164799      2.705263      0.700220
S          1.843777     -0.747311     -0.560709
C          3.349958     -0.920280      0.314067
H          3.179447     -0.923618      1.413859
H          3.808637     -1.889184      0.015757
H          4.064768     -0.100015      0.077378
```

3_methylcrotonate_5_reopt3_am1_HEI

Datum	Value
AM1 Energy	-0.188816
AM1 Free Energy (Quasiharmonic)	-0.059455
Number of Imaginary Frequencies	0

Frequencies (Top 3 out of 54)

```
1.      33.4622 cm-1
2.      47.1639 cm-1
3.      83.6736 cm-1
```

AM1 Molecular Geometry in Cartesian Coordinates

```
C          0.979191     -0.741325     -0.778770
C         -0.044906      0.179246     -1.156589
C         -1.305725      0.376912     -0.589819
O         -1.669728     -0.459226      0.480071
O         -2.203565      1.199492     -0.903469
C         -2.950020     -0.238010      1.036416
H         -3.030516     -0.986173      1.864273
```

```
H          -3.034830       0.803284       1.432784
H          -3.747280      -0.405151       0.271444
H           1.586944      -1.064333      -1.665719
C           0.584688      -1.931963       0.046856
H           0.184281       0.856894      -1.989252
H           1.453049      -2.620373       0.189809
H           0.213543      -1.611973       1.050817
H          -0.239763      -2.490173      -0.462321
S           2.417276       0.061780       0.190536
C           1.752471       1.531787       0.873858
H           2.395308       2.393543       0.590373
H           0.717379       1.696942       0.481979
H           1.717629       1.457032       1.982708
```

3_methylcrotonate_6_am1_HEI

Datum	Value
AM1 Energy	-0.188281
AM1 Free Energy (Quasiharmonic)	-0.058835
Number of Imaginary Frequencies	0

Frequencies (Top 3 out of 54)

```
1.      26.8692 cm-1
2.      53.4528 cm-1
3.      78.1351 cm-1
```

AM1 Molecular Geometry in Cartesian Coordinates

```
C           0.952951       0.793279       0.378084
C          -0.364266       0.582180      -0.149321
C          -1.368450      -0.024169       0.609384
O          -2.657408      -0.237177       0.071240
O          -1.376553      -0.449401       1.788892
C          -2.881979       0.161340      -1.258746
H          -2.744943       1.265082      -1.378744
H          -2.203107      -0.379947      -1.964071
H          -3.947697      -0.114587      -1.468149
H           0.992827       0.648091       1.486516
C           1.602862       2.087943      -0.033819
H          -0.553008       0.893924      -1.178174
H           2.682258       2.104096       0.256864
H           1.529623       2.236066      -1.138027
H           1.087536       2.942475       0.470437
```

```
S           2.247953       -0.443616       -0.243426
C           1.375516       -1.959306       -0.362560
H           1.562563       -2.414346       -1.359585
H           1.718839       -2.663615        0.426424
H           0.279748       -1.774353       -0.237867
```

3_methylcrotonate_7_am1_HEI

Datum	Value
AM1 Energy	-0.187286
AM1 Free Energy (Quasiharmonic)	-0.058627
Number of Imaginary Frequencies	0

Frequencies (Top 3 out of 54)

```
1.      40.7085 cm-1
2.      55.3036 cm-1
3.      72.9681 cm-1
```

AM1 Molecular Geometry in Cartesian Coordinates

```
C           0.756276        0.521337        0.424525
C          -0.352130        0.997839       -0.349808
C          -1.634509        0.446175       -0.328265
O          -1.837011       -0.598607        0.594291
O          -2.669065        0.754316       -0.970567
C          -3.128277       -1.169637        0.612831
H          -3.397800       -1.574741       -0.393109
H          -3.063578       -1.992584        1.368135
H          -3.890608       -0.411493        0.918729
H           0.457776       -0.192108        1.232291
C           1.644672        1.606391        0.977061
H          -0.193136        1.827380       -1.044694
H           2.549936        1.171631        1.467688
H           1.976678        2.297784        0.165923
H           1.082191        2.202247        1.737580
S           1.764526       -0.485085       -0.771224
C           3.078016       -1.192048        0.144503
H           3.871825       -0.446330        0.376014
H           2.715499       -1.620157        1.105723
H           3.523126       -2.006274       -0.469573
```

3_methylcrotonate_8_reopt_am1_HEI_reopt

Datum	Value
AM1 Energy	-0.19044
AM1 Free Energy (Quasiharmonic)	-0.061359
Number of Imaginary Frequencies	0

Frequencies (Top 3 out of 54)

```
1.      37.5280 cm-1
2.      50.8567 cm-1
3.      64.8926 cm-1
```

AM1 Molecular Geometry in Cartesian Coordinates

```
C          1.129815        0.813030        0.645806
C         -0.129882        0.201615        0.939120
C         -1.247765        0.219298        0.103120
O         -2.342344       -0.495624        0.639980
O         -1.497022        0.738704       -1.011713
C         -3.522417       -0.499307       -0.134333
H         -3.340153       -0.969156       -1.131949
H         -4.251137       -1.104427        0.461318
H         -3.901478        0.541365       -0.284204
H          1.654276        1.147320        1.579969
C          1.134899        1.906805       -0.384120
H         -0.213173       -0.355605        1.878259
H          2.177721        2.240311       -0.604832
H          0.653783        1.556465       -1.330525
H          0.550789        2.782668       -0.006170
S          2.466113       -0.419149        0.071985
C          1.589446       -1.782628       -0.592945
H          1.948732       -2.719818       -0.114557
H          0.494974       -1.661013       -0.395141
H          1.758203       -1.849244       -1.689952
```

4_methylmethacrylate_1_am1

Datum	Value
AM1 Energy	-0.122128
AM1 Free Energy (Quasiharmonic)	-0.027479

Datum	Value
Number of Imaginary Frequencies	0

Frequencies (Top 3 out of 39)

```
1.      16.8333 cm-1
2.      65.9829 cm-1
3.     107.4095 cm-1
```

AM1 Molecular Geometry in Cartesian Coordinates

```
C          1.408626       1.551213       0.075878
C          1.160591       0.238666       0.006574
C         -0.212607      -0.299396       0.029831
O         -1.194990       0.653712      -0.067255
O         -0.568209      -1.477253       0.124284
C         -2.543161       0.183740      -0.046825
H         -3.149439       1.118277      -0.138292
H         -2.719617      -0.505448      -0.907353
H         -2.747726      -0.345619       0.914705
H          0.605200       2.298385       0.144653
H          2.432720       1.948981       0.067210
C          2.220988      -0.793219      -0.093482
H          2.183041      -1.283133      -1.098918
H          3.233342      -0.343620       0.049196
H          2.061445      -1.585511       0.680708
```

4_methylmethacrylate_1_reopt_am1_HEI_reopt

Datum	Value
AM1 Energy	-0.19663
AM1 Free Energy (Quasiharmonic)	-0.068012
Number of Imaginary Frequencies	0

Frequencies (Top 3 out of 54)

```
1.      20.2413 cm-1
2.      45.2970 cm-1
3.      59.1946 cm-1
```

AM1 Molecular Geometry in Cartesian Coordinates

```
C         1.159949      0.491007     -1.045466
C        -0.031557      0.794183     -0.297182
C        -1.126768     -0.079737     -0.362287
O        -2.235569      0.290284      0.423964
O        -1.309242     -1.138639     -1.011844
C        -3.366259     -0.552252      0.336448
H        -3.115220     -1.587353      0.674752
H        -4.120211     -0.083864      1.017585
H        -3.750057     -0.592561     -0.712294
H         0.975231     -0.254201     -1.854373
H         1.662338      1.401407     -1.454747
C        -0.061208      2.007079      0.532633
H         0.398645      2.873614     -0.010647
H        -1.107136      2.288141      0.810796
H         0.519768      1.867510      1.482337
C         1.840469     -1.210432      1.154111
H         0.742093     -1.004850      1.188691
H         2.004477     -2.288990      0.939564
H         2.294214     -0.963564      2.138549
S         2.585412     -0.222972     -0.089170
```

4_methylmethacrylate_2_am1_HEI_reopt

Datum	Value
AM1 Energy	-0.196996
AM1 Free Energy (Quasiharmonic)	-0.068232
Number of Imaginary Frequencies	0

Frequencies (Top 3 out of 54)

```
1.     19.1799 cm-1
2.     65.9393 cm-1
3.     76.1965 cm-1
```

AM1 Molecular Geometry in Cartesian Coordinates

```
C        -0.935043      0.214973     -1.135635
C        -0.001331      0.938820     -0.318900
C         1.256733      0.430276      0.037026
O         1.557162     -0.846290     -0.474270
```

```
O          2.182510       0.920362       0.729439
C          2.827995      -1.366178      -0.141569
H          2.859067      -2.370353      -0.633785
H          3.640146      -0.706326      -0.533912
H          2.938065      -1.463577       0.966238
H         -0.458545      -0.608987      -1.715422
H         -1.525272       0.879047      -1.813853
C         -0.377127       2.276322       0.169610
H          0.454133       2.737896       0.758200
H         -0.623933       2.961232      -0.683729
H         -1.285465       2.225065       0.825156
C         -1.718604      -0.998531       1.345036
H         -2.429503      -0.625423       2.114080
H         -1.601161      -2.097764       1.463777
H         -0.725758      -0.503278       1.485915
S         -2.342181      -0.624637      -0.250464
```

4_methylmethacrylate_2_am1

Datum	Value
AM1 Energy	-0.1223
AM1 Free Energy (Quasiharmonic)	-0.027164
Number of Imaginary Frequencies	0

Frequencies (Top 3 out of 39)

```
1.      34.7745 cm-1
2.     103.2036 cm-1
3.     109.2900 cm-1
```

AM1 Molecular Geometry in Cartesian Coordinates

```
C         -2.163182      -0.903750       0.000155
C         -1.204730       0.031332       0.000012
C          0.210566      -0.384471      -0.000048
O          1.100867       0.658045       0.000094
O          0.670531      -1.530662      -0.000187
C          2.486301       0.310784       0.000078
H          3.005348       1.300680       0.000093
H          2.731143      -0.280898       0.914689
H          2.731168      -0.280864      -0.914546
H         -1.928767      -1.978442       0.000225
H         -3.229900      -0.642613       0.000206
C         -1.474896       1.487123      -0.000107
```

```
  H          -1.016848        1.960980       -0.904560
  H          -2.572401        1.694559        0.000777
  H          -1.015292        1.961422        0.903323
```

4_methylmethacrylate_3_am1_HEI

Datum	Value
AM1 Energy	-0.196619
AM1 Free Energy (Quasiharmonic)	-0.068186
Number of Imaginary Frequencies	0

Frequencies (Top 3 out of 54)

```
    1.      20.4133 cm-1
    2.      30.8755 cm-1
    3.      60.5212 cm-1
```

AM1 Molecular Geometry in Cartesian Coordinates

```
  C          -1.164496        0.534608        1.012152
  C           0.036488        0.813710        0.268472
  C           1.119898       -0.071896        0.351063
  O           2.254385        0.293444       -0.400029
  O           1.271106       -1.142867        0.989836
  C           3.360632       -0.581296       -0.316708
  H           3.088603       -1.599994       -0.687020
  H           4.138378       -0.115156       -0.972184
  H           3.724893       -0.659670        0.736848
  H          -0.992714       -0.193265        1.839326
  H          -1.662609        1.458779        1.394944
  C           0.085857        2.032381       -0.551758
  H          -0.808273        2.096750       -1.225241
  H           0.080586        2.952762        0.091121
  H           1.004055        2.066193       -1.187901
  C          -1.837221       -1.320377       -1.052900
  H          -2.226148       -1.141205       -2.078814
  H          -0.728605       -1.178110       -1.045166
  H          -2.074317       -2.364412       -0.752807
  S          -2.584670       -0.185630        0.056657
```

4_methylmethacrylate_3_am1

Datum	Value
AM1 Energy	-0.112769
AM1 Free Energy (Quasiharmonic)	-0.01791
Number of Imaginary Frequencies	0

Frequencies (Top 3 out of 39)

```
1.      40.1635 cm-1
2.      95.7542 cm-1
3.     102.2911 cm-1
```

AM1 Molecular Geometry in Cartesian Coordinates

```
C         -1.313309       -0.738686       -1.232310
C         -0.908889       -0.090703       -0.137136
C          0.340309        0.703759       -0.124118
O          1.553579        0.089796        0.082517
O          0.447794        1.920629       -0.268788
C          1.544747       -1.318469        0.283471
H          2.623290       -1.574985        0.436036
H          0.942240       -1.579022        1.187670
H          1.135895       -1.837773       -0.617443
H         -0.734476       -0.742898       -2.165885
H         -2.257317       -1.299900       -1.260281
C         -1.678134       -0.048079        1.129351
H         -1.919872        1.013062        1.392074
H         -2.633407       -0.620528        1.039068
H         -1.075675       -0.488294        1.963383
```

4_methylmethacrylate_4_reopt_am1_HEI

Datum	Value
AM1 Energy	-0.196996
AM1 Free Energy (Quasiharmonic)	-0.068231
Number of Imaginary Frequencies	0

Frequencies (Top 3 out of 54)

```
1.      19.1912 cm-1
2.      65.9432 cm-1
```

```
3.      76.2419 cm-1
```

AM1 Molecular Geometry in Cartesian Coordinates

```
C          0.935023        0.214900        1.135661
C          0.001335        0.938791        0.318936
C         -1.256734        0.430276       -0.037018
O         -1.557184       -0.846304        0.474233
O         -2.182502        0.920396       -0.729418
C         -2.828024       -1.366162        0.141515
H         -2.859074       -2.370392        0.633625
H         -3.640164       -0.706354        0.533959
H         -2.938129       -1.463441       -0.966301
H          0.458498       -0.609067        1.715420
H          1.525267        0.878939        1.813903
C          0.377158        2.276296       -0.169545
H          0.624164        2.961128        0.683801
H          1.285377        2.225014       -0.825255
H         -0.454166        2.737984       -0.757961
C          1.718662       -0.998378       -1.345100
H          1.601085       -2.097586       -1.463953
H          0.725886       -0.502990       -1.486007
H          2.429676       -0.625285       -2.114045
S          2.342159       -0.624689        0.250475
```

4_methylmethacrylate_5_am1_HEI

Datum	Value
AM1 Energy	-0.192043
AM1 Free Energy (Quasiharmonic)	-0.063896
Number of Imaginary Frequencies	0

Frequencies (Top 3 out of 54)

```
1.      33.8345 cm-1
2.      42.6979 cm-1
3.      55.4077 cm-1
```

AM1 Molecular Geometry in Cartesian Coordinates

```
C         -1.043620       0.100774       0.801787
C          0.232708       0.614864       0.384118
C          1.304153      -0.275162       0.207584
O          2.497099       0.314277      -0.254555
O          1.408496      -1.506838       0.423875
C          3.601216      -0.554469      -0.401557
H          3.881461      -1.007768       0.580846
H          3.369089      -1.370575      -1.128773
H          4.424725       0.098097      -0.786050
H         -0.980599      -0.928755       1.226540
H         -1.578058       0.775915       1.512989
C          0.380013       2.053928       0.123608
H         -0.217545       2.654543       0.858110
H          1.449570       2.372702       0.203033
H          0.019647       2.329989      -0.901744
C         -3.630131      -0.702051       0.040365
H         -3.428086      -1.689387       0.513320
H         -4.071462      -0.033190       0.813245
H         -4.367516      -0.839158      -0.781232
S         -2.175751      -0.015576      -0.650018
```

4_methylmethacrylate_6_am1_HEI

Datum	Value
AM1 Energy	-0.192364
AM1 Free Energy (Quasiharmonic)	-0.064092
Number of Imaginary Frequencies	0

Frequencies (Top 3 out of 54)

```
1.      35.6308 cm-1
2.      56.7394 cm-1
3.      69.2218 cm-1
```

AM1 Molecular Geometry in Cartesian Coordinates

```
C         -0.866514       0.167550       0.779214
C          0.267188       0.903195       0.295654
C          1.497508       0.296047      -0.000526
O          1.568278      -1.084273       0.266039
O          2.573486       0.775378      -0.434540
C          2.793144      -1.715014      -0.045791
H          3.622673      -1.293871       0.573789
```

```
H          2.624936      -2.793928       0.197966
H          3.041720      -1.586393      -1.127559
H         -1.473513       0.738823       1.523016
H         -0.605937      -0.833819       1.193514
C          0.136889       2.355945       0.092691
H          1.076342       2.786742      -0.334381
H         -0.708368       2.593716      -0.603390
H         -0.076600       2.879675       1.062333
C         -3.348596      -1.010085       0.162413
H         -4.108956      -1.247627      -0.614128
H         -2.996201      -1.959482       0.624807
H         -3.825848      -0.395563       0.958736
S         -2.036380      -0.150184      -0.613039
```

5_methyltiglate_1_am1_HEI

Datum	Value
AM1 Energy	-0.201554
AM1 Free Energy (Quasiharmonic)	-0.046251
Number of Imaginary Frequencies	0

Frequencies (Top 3 out of 63)

```
1.      28.4092 cm-1
2.      54.3013 cm-1
3.      71.6830 cm-1
```

AM1 Molecular Geometry in Cartesian Coordinates

```
C         -1.094249      -0.527325       0.640770
C          0.118997      -0.663644      -0.130527
C          1.289571      -0.046585       0.336660
O          2.428084      -0.228256      -0.472212
O          1.514610       0.638565       1.364936
C          3.618793       0.376468      -0.010682
H          3.495624       1.484437       0.067131
H          3.907965      -0.031260       0.988773
H          4.384821       0.115165      -0.783140
H         -0.882518      -0.128913       1.665747
C         -1.966413      -1.753096       0.705549
C          0.104303      -1.440186      -1.378423
H          1.029525      -1.267723      -1.981618
H          0.032684      -2.541390      -1.170960
H         -0.780519      -1.164116      -2.009316
```

```
H          -1.422418       -2.571883        1.238020
H          -2.913788       -1.536507        1.258000
H          -2.225781       -2.113143       -0.319348
S          -2.328840        0.735146       -0.033759
C          -1.330618        2.023455       -0.679124
H          -1.670031        2.270925       -1.708545
H          -1.415329        2.930918       -0.042084
H          -0.262659        1.694154       -0.709651
```

5_methyltiglate_1_am1_reopt

Datum	Value
AM1 Energy	-0.137258
AM1 Free Energy (Quasiharmonic)	-0.016343
Number of Imaginary Frequencies	0

Frequencies (Top 3 out of 48)

```
 1.      33.8530 cm-1
 2.      49.3734 cm-1
 3.      81.7650 cm-1
```

AM1 Molecular Geometry in Cartesian Coordinates

```
C           1.737310       -0.682463       -0.000079
C           0.704516        0.180786        0.000003
C          -0.665525       -0.363081       -0.000018
O          -1.648799        0.593123        0.000071
O          -1.020128       -1.546764       -0.000105
C          -2.995787        0.118814        0.000055
H          -3.604650        1.056137        0.000137
H          -3.184923       -0.493029       -0.914507
H          -3.184895       -0.493170        0.914527
H           1.533617       -1.769879       -0.000161
C           3.157348       -0.287238       -0.000070
C           0.831973        1.654839        0.000116
H           0.328261        2.081291        0.904136
H           1.902902        1.972173        0.000128
H           0.328240        2.081431       -0.903826
H           3.820021       -1.187259       -0.000156
H           3.396898        0.325826       -0.905928
H           3.396928        0.325675        0.905882
```

5_methyltiglate_2_am1_HEI

Datum	Value
AM1 Energy	-0.201848
AM1 Free Energy (Quasiharmonic)	-0.04645
Number of Imaginary Frequencies	0

Frequencies (Top 3 out of 63)

```
1.      23.8674 cm-1
2.      56.1355 cm-1
3.      79.1135 cm-1
```

AM1 Molecular Geometry in Cartesian Coordinates

```
C          0.906420        0.316693        0.771465
C         -0.055146        0.858367       -0.153343
C         -1.391405        0.427761       -0.188782
O         -1.738696       -0.550383        0.761544
O         -2.351034        0.773341       -0.920977
C         -3.084866       -0.979482        0.749474
H         -3.143114       -1.745695        1.562550
H         -3.773869       -0.124872        0.958747
H         -3.347876       -1.428883       -0.239288
H          0.415797       -0.251195        1.601332
C          1.913750        1.295497        1.315308
C          0.357624        1.896038       -1.113891
H         -0.457886        2.108972       -1.849115
H          0.609455        2.855929       -0.589758
H          1.270949        1.577094       -1.680061
H          1.389255        2.078692        1.916575
H          2.653889        0.777009        1.973302
H          2.466678        1.800479        0.486814
S          2.068602       -0.995278        0.044069
C          1.139490       -1.767190       -1.225339
H          1.735732       -1.778362       -2.163745
H          0.895570       -2.813792       -0.939678
H          0.190425       -1.200688       -1.396678
```

5_methyltiglate_2_am1

Datum	Value

Datum	Value
AM1 Energy	-0.137423
AM1 Free Energy (Quasiharmonic)	-0.016722
Number of Imaginary Frequencies	0

Frequencies (Top 3 out of 48)

```
1.      19.4043 cm-1
2.      32.6796 cm-1
3.      96.7783 cm-1
```

AM1 Molecular Geometry in Cartesian Coordinates

```
C          1.364640      -0.876664       0.000011
C          0.729531       0.308878       0.000001
C         -0.742585       0.380451       0.000010
O         -1.372773      -0.839823      -0.000020
O         -1.459273       1.385871       0.000041
C         -2.800259      -0.819552      -0.000013
H         -3.174268      -0.299627       0.914551
H         -3.174275      -0.299609      -0.914564
H         -3.079346      -1.901862      -0.000023
H          0.780862      -1.816058       0.000027
C          2.828141      -1.049065       0.000010
C          1.423422       1.619851      -0.000031
H          0.690066       2.465074      -0.000031
H          2.071415       1.715134       0.907044
H          2.071387       1.715113      -0.907126
H          3.139903      -1.628240      -0.906387
H          3.373371      -0.073468       0.000004
H          3.139908      -1.628231       0.906412
```

5_methyltiglate_3_am1

Datum	Value
AM1 Energy	-0.127248
AM1 Free Energy (Quasiharmonic)	-0.006773
Number of Imaginary Frequencies	0

Frequencies (Top 3 out of 48)

```
 1.      29.8969 cm-1
 2.      43.9998 cm-1
 3.      73.7279 cm-1
```

AM1 Molecular Geometry in Cartesian Coordinates

```
C          1.303213        0.321701       -0.707128
C          0.455774       -0.345133        0.091259
C         -0.956751       -0.542711       -0.305307
O         -1.880204        0.460472       -0.122191
O         -1.455955       -1.554893       -0.795277
C         -1.421782        1.667514        0.474356
H         -2.344160        2.296465        0.551775
H         -0.992290        1.465144        1.485779
H         -0.659625        2.158375       -0.178806
H          0.940053        0.751904       -1.657388
C          2.736229        0.524782       -0.421861
C          0.823205       -0.987331        1.374499
H          0.425926       -2.033517        1.407419
H          1.932508       -1.024502        1.504690
H          0.383143       -0.415688        2.230473
H          3.187024        1.243971       -1.148980
H          2.889029        0.926832        0.611157
H          3.288338       -0.446560       -0.501276
```

5_methyltiglate_3_reopt_am1_HEI

Datum	Value
AM1 Energy	-0.196861
AM1 Free Energy (Quasiharmonic)	-0.0418
Number of Imaginary Frequencies	0

Frequencies (Top 3 out of 63)

```
 1.      35.4307 cm-1
 2.      49.9067 cm-1
 3.      65.3242 cm-1
```

AM1 Molecular Geometry in Cartesian Coordinates

```
C          0.922663        0.220380        0.583743
C         -0.356600        0.609963        0.034633
C         -1.459125       -0.234875        0.239408
O         -2.661509        0.196671       -0.353965
O         -1.581045       -1.300242        0.892101
C         -3.782846       -0.640402       -0.160066
H         -3.589577       -1.662967       -0.567078
H         -4.033674       -0.719521        0.926191
H         -4.609928       -0.138450       -0.722080
H          0.823673       -0.630388        1.306201
C          1.720065        1.341485        1.198441
C         -0.475708        1.862724       -0.724396
H          0.395227        1.998055       -1.416589
H         -1.412283        1.883920       -1.334570
H         -0.495275        2.752963       -0.039356
H          1.156523        1.774053        2.061654
H          2.707364        0.970688        1.568818
H          1.900522        2.155361        0.455547
S          1.916822       -0.470320       -0.828540
C          3.341042       -1.166761       -0.086144
H          4.074454       -0.386592        0.219241
H          3.075250       -1.763865        0.814846
H          3.822062       -1.834649       -0.834991
```

5_methyltiglate_4_am1_HEI

Datum	Value
AM1 Energy	-0.201554
AM1 Free Energy (Quasiharmonic)	-0.046244
Number of Imaginary Frequencies	0

Frequencies (Top 3 out of 63)

```
1.      28.5346 cm-1
2.      54.3709 cm-1
3.      71.7317 cm-1
```

AM1 Molecular Geometry in Cartesian Coordinates

```
C          1.094263        0.527201        0.640782
C         -0.119003        0.663678       -0.130474
C         -1.289561        0.046439        0.336580
O         -2.428230        0.228706       -0.471931
```

```
O          -1.514428       -0.639369        1.364457
C          -3.618824       -0.376457       -0.010669
H          -3.495663       -1.484524        0.065895
H          -3.907657        0.030193        0.989331
H          -4.385085       -0.114281       -0.782613
H           0.882498        0.128519        1.665665
C           1.966422        1.752950        0.706017
C          -0.104436        1.440589       -1.378138
H          -0.033732        2.541811       -1.170376
H           0.780863        1.165384       -2.008745
H          -1.029307        1.267579       -1.981730
H           1.422471        2.571460        1.238981
H           2.913882        1.536099        1.258239
H           2.225649        2.113543       -0.318720
S           2.328870       -0.734946       -0.034189
C           1.330676       -2.023548       -0.679007
H           1.416244       -2.931044       -0.042126
H           0.262558       -1.694617       -0.708800
H           1.669401       -2.270774       -1.708725
```

5_methyltiglate_4_am1_reopt

Datum	Value
AM1 Energy	-0.127411
AM1 Free Energy (Quasiharmonic)	-0.006509
Number of Imaginary Frequencies	0

Frequencies (Top 3 out of 48)

```
1.      33.5100 cm-1
2.      49.9769 cm-1
3.      85.2701 cm-1
```

AM1 Molecular Geometry in Cartesian Coordinates

```
C          -1.422894        0.323270       -0.671827
C          -0.465814        0.143337        0.251445
C           0.890660        0.691545        0.029487
O           1.931363       -0.144173       -0.304017
O           1.245872        1.865884        0.123024
C           1.625428       -1.517213       -0.513385
H           2.609649       -1.972689       -0.789750
H           1.225071       -1.975075        0.424187
H           0.888320       -1.630852       -1.344792
```

```
H          -1.191493        0.860175       -1.609640
C          -2.816458       -0.140217       -0.546739
C          -0.672989       -0.528962        1.556253
H           0.277647       -0.575525        2.143048
H          -1.430509        0.030938        2.160826
H          -1.046377       -1.573026        1.405310
H          -3.083993       -0.786721       -1.420587
H          -2.987596       -0.723816        0.390904
H          -3.506198        0.742346       -0.542965
```

5_methyltiglate_5_am1_HEI

Datum	Value
AM1 Energy	-0.201848
AM1 Free Energy (Quasiharmonic)	-0.046452
Number of Imaginary Frequencies	0

Frequencies (Top 3 out of 63)

```
1.      23.8831 cm-1
2.      56.1363 cm-1
3.      79.0712 cm-1
```

AM1 Molecular Geometry in Cartesian Coordinates

```
C           0.906471        0.316753        0.771372
C          -0.055176        0.858469       -0.153326
C          -1.391420        0.427812       -0.188729
O          -1.738630       -0.550415        0.761535
O          -2.351082        0.773399       -0.920877
C          -3.084774       -0.979599        0.749452
H          -3.142960       -1.745881        1.562461
H          -3.773826       -0.125054        0.958809
H          -3.347774       -1.428931       -0.239341
H           0.415910       -0.251154        1.601254
C           1.913909        1.295512        1.315089
C           0.357466        1.896350       -1.113704
H          -0.458214        2.109582       -1.848649
H           0.609545        2.856048       -0.589341
H           1.270595        1.577471       -1.680222
H           1.389481        2.078968        1.916068
H           2.653872        0.777068        1.973308
H           2.467031        1.800160        0.486518
S           2.068627       -0.995274        0.043932
```

```
C          1.139333       -1.767558       -1.225120
H          0.895565       -2.814124       -0.939203
H          0.190178       -1.201189       -1.396416
H          1.735400       -1.778891       -2.163632
```

5_methyltiglate_6_am1_HEI

Datum	Value
AM1 Energy	-0.197033
AM1 Free Energy (Quasiharmonic)	-0.041777
Number of Imaginary Frequencies	0

Frequencies (Top 3 out of 63)

```
  1.      36.8326 cm-1
  2.      50.6583 cm-1
  3.      74.9279 cm-1
```

AM1 Molecular Geometry in Cartesian Coordinates

```
C          0.766257        0.189549        0.583586
C         -0.366529        0.878841        0.017740
C         -1.627666        0.272612       -0.104775
O         -1.733961       -1.022023        0.437150
O         -2.704874        0.699906       -0.587261
C         -2.990230       -1.652748        0.297079
H         -2.847673       -2.667797        0.745550
H         -3.780174       -1.084422        0.846940
H         -3.277100       -1.731042       -0.779983
H          0.469902       -0.733460        1.143514
C          1.673799        1.042851        1.432306
C         -0.214151        2.259275       -0.472531
H          0.677966        2.354078       -1.143571
H         -1.120959        2.582975       -1.041661
H         -0.066612        2.976790        0.378714
H          2.532239        0.442806        1.822855
H          2.077734        1.901246        0.843382
H          1.101726        1.452338        2.301099
S          1.773041       -0.443194       -0.852267
C          3.011143       -1.453333       -0.137515
H          3.821713       -0.849293        0.329360
H          2.585320       -2.122692        0.643256
H          3.452192       -2.075756       -0.947636
```

7_isobutylacrylate_10_reopt_am1_HEI

Datum	Value
AM1 Energy	-0.212947
AM1 Free Energy (Quasiharmonic)	-0.029409
Number of Imaginary Frequencies	0

Frequencies (Top 3 out of 72)

```
1.      10.7078 cm-1
2.      24.7508 cm-1
3.      36.5580 cm-1
```

AM1 Molecular Geometry in Cartesian Coordinates

```
C          0.142274        1.352974        0.304439
C          1.534312        1.354842        0.211394
C          2.293361        0.720926       -0.819145
O         -0.615785        1.862999        1.167196
O         -0.528763        0.693945       -0.744626
C         -1.942045        0.652464       -0.717724
C         -2.425525       -0.717968       -0.232000
C         -3.803255       -1.011796       -0.782869
C         -2.425520       -0.774102        1.279742
H          2.066199        1.871896        1.016003
H          1.687329        0.455021       -1.715637
H          3.200778        1.299618       -1.120376
H         -2.371186        1.467935       -0.079116
H         -2.251083        0.805394       -1.788561
H         -1.704827       -1.492183       -0.619411
H         -4.175086       -1.991319       -0.396651
H         -3.778722       -1.056733       -1.898337
H         -4.525296       -0.216136       -0.478020
H         -2.555736       -1.823788        1.635471
H         -1.456276       -0.365607        1.664836
H         -3.253536       -0.151862        1.696843
S          3.124782       -0.879199       -0.341481
C          2.078406       -1.578951        0.879011
H          1.296576       -0.835331        1.175633
H          2.684297       -1.852519        1.770044
H          1.584397       -2.493072        0.483333
```

7_isobutylacrylate_11_am1_HEI

Datum	Value
AM1 Energy	-0.213929
AM1 Free Energy (Quasiharmonic)	-0.030259
Number of Imaginary Frequencies	0

Frequencies (Top 3 out of 72)

```
1.      23.7348 cm-1
2.      29.4288 cm-1
3.      43.2580 cm-1
```

AM1 Molecular Geometry in Cartesian Coordinates

```
C          0.118121       -0.972085       -0.049999
C          1.346615       -0.961780       -0.711543
C          2.587998       -1.066685       -0.010041
O         -0.199146       -1.096411        1.158215
O         -0.983708       -0.833638       -0.922991
C         -2.275927       -0.894132       -0.352225
C         -2.705416        0.459067        0.217406
C         -4.024698        0.323030        0.943332
C         -2.794927        1.500038       -0.875264
H          1.356308       -0.850154       -1.798259
H          3.348614       -1.686173       -0.545046
H          2.465665       -1.417680        1.041060
H         -2.944171       -1.188594       -1.206303
H         -2.318315       -1.670631        0.456589
H         -1.907518        0.767969        0.952574
H         -4.821051       -0.042052        0.250324
H         -3.929747       -0.402526        1.786798
H         -4.344400        1.309543        1.357380
H         -1.833353        1.525726       -1.444259
H         -3.621261        1.256938       -1.586216
H         -2.988449        2.510204       -0.441361
S          3.566648        0.512061        0.141048
C          2.355958        1.777537        0.225008
H          2.314639        2.205443        1.250360
H          2.618953        2.584034       -0.493477
H          1.354202        1.355427       -0.038762
```

7_isobutylacrylate_12_am1_HEI

Datum	Value
AM1 Energy	-0.211393
AM1 Free Energy (Quasiharmonic)	-0.027833
Number of Imaginary Frequencies	0

Frequencies (Top 3 out of 72)

```
1.      18.5178 cm-1
2.      27.8360 cm-1
3.      34.1218 cm-1
```

AM1 Molecular Geometry in Cartesian Coordinates

```
C       -0.054539       1.041476       -0.198894
C        1.086301       0.804163       -0.966524
C        1.713529      -0.467621       -1.139033
O       -0.685386       2.106588        0.014899
O       -0.597505      -0.095303        0.433497
C       -1.792166       0.054510        1.174078
C       -3.009389      -0.448528        0.391432
C       -2.788027      -1.865265       -0.089305
C       -3.312878       0.476208       -0.766071
H        1.529933       1.681120       -1.447754
H        1.063701      -1.323661       -0.843436
H        2.118492      -0.624233       -2.168958
H       -1.645429      -0.589756        2.084469
H       -1.955659       1.120639        1.482829
H       -3.885830      -0.441031        1.099757
H       -1.861667      -1.910076       -0.713552
H       -2.658825      -2.553766        0.780014
H       -3.655036      -2.216941       -0.698194
H       -2.393905       0.611046       -1.389912
H       -4.133905       0.064237       -1.399724
H       -3.615589       1.481782       -0.386698
S        3.285991      -0.735043       -0.173581
C        3.093967       0.240610        1.270480
H        3.997327       0.873575        1.408815
H        2.193414       0.895825        1.163286
H        2.969461      -0.411673        2.162204
```

7_isobutylacrylate_13_reopt_am1_HEI

Datum	Value

Datum	Value
AM1 Energy	-0.21279
AM1 Free Energy (Quasiharmonic)	-0.029863
Number of Imaginary Frequencies	0

Frequencies (Top 3 out of 72)

```
1.      17.7803 cm-1
2.      25.8102 cm-1
3.      38.7977 cm-1
```

AM1 Molecular Geometry in Cartesian Coordinates

```
C        -0.292293      1.409674      0.170120
C        -1.570880      1.158595      0.669951
C        -2.024988     -0.096818      1.175732
O         0.213653      2.460279     -0.297598
O         0.597623      0.318723      0.204653
C         1.911374      0.573714     -0.270715
C         2.682207     -0.743928     -0.182188
C         2.935481     -1.118434      1.261290
C         3.981620     -0.628447     -0.948207
H        -2.268010      2.001651      0.644623
H        -2.710341      0.000649      2.053058
H        -1.197653     -0.807027      1.406009
H         2.395218      1.366651      0.357348
H         1.866166      0.935783     -1.331667
H         2.047940     -1.547462     -0.651818
H         3.604592     -0.370441      1.750753
H         1.964163     -1.141425      1.813689
H         3.418010     -2.122884      1.328149
H         3.781830     -0.411080     -2.025027
H         4.609323      0.196767     -0.533184
H         4.559484     -1.581324     -0.878396
S        -3.150946     -1.069365      0.047084
C        -2.686126     -0.587463     -1.573171
H        -3.593775     -0.293335     -2.143720
H        -1.983121      0.281317     -1.518808
H        -2.187267     -1.431385     -2.097663
```

7_isobutylacrylate_14_am1_HEI

Datum	Value

Datum	Value
AM1 Energy	-0.21395
AM1 Free Energy (Quasiharmonic)	-0.030183
Number of Imaginary Frequencies	0

Frequencies (Top 3 out of 72)

```
1.      24.3687 cm-1
2.      34.5569 cm-1
3.      47.1779 cm-1
```

AM1 Molecular Geometry in Cartesian Coordinates

```
C         -0.088828       -0.098421       -0.120196
C         -1.177591       -0.899420        0.226086
C         -2.308012       -1.058416       -0.634829
O          0.181328        0.578353       -1.142402
O          0.911362       -0.060096        0.876584
C          2.065172        0.712315        0.609616
C          3.079162       -0.060421       -0.235851
C          3.574902       -1.283625        0.501413
C          4.229839        0.841242       -0.621720
H         -1.171386       -1.402436        1.195853
H         -2.722780       -2.095967       -0.634716
H         -2.112880       -0.714443       -1.677234
H          1.793055        1.667241        0.087042
H          2.491631        0.935216        1.625038
H          2.537211       -0.390244       -1.168536
H          4.218198       -1.909386       -0.162500
H          4.173154       -0.989683        1.397555
H          2.701320       -1.891968        0.841712
H          4.974794        0.280228       -1.235865
H          3.858706        1.709117       -1.218332
H          4.747269        1.232315        0.287697
S         -3.847105       -0.139695       -0.125555
C         -3.256287        1.312475        0.659680
H         -3.780257        1.442227        1.631498
H         -2.156018        1.219943        0.838834
H         -3.449998        2.202531        0.022175
```

7_isobutylacrylate_15_am1_HEI

Datum	Value

Datum	Value
AM1 Energy	-0.213477
AM1 Free Energy (Quasiharmonic)	-0.029461
Number of Imaginary Frequencies	0

Frequencies (Top 3 out of 72)

```
1.      23.5176 cm-1
2.      32.8543 cm-1
3.      35.9108 cm-1
```

AM1 Molecular Geometry in Cartesian Coordinates

```
C        -0.097914       0.158015      -0.022999
C        -1.191710       0.397995      -0.855417
C        -2.333413       1.138069      -0.414930
O         0.165472       0.504200       1.154777
O         0.907571      -0.614543      -0.644122
C         2.081914      -0.900923       0.088541
C         3.218202       0.039347      -0.328684
C         4.554996      -0.652705      -0.178826
C         3.177730       1.313850       0.485581
H        -1.180930      -0.012467      -1.867810
H        -2.758941       1.806143      -1.203109
H        -2.145298       1.710002       0.523477
H         1.907378      -0.837475       1.194363
H         2.340741      -1.958527      -0.195025
H         3.064107       0.301764      -1.413202
H         4.701884      -0.998114       0.873044
H         4.615286      -1.540938      -0.852835
H         5.385836       0.046653      -0.438733
H         2.124503       1.693100       0.528284
H         3.520615       1.123715       1.531145
H         3.837090       2.092025       0.032661
S        -3.855692       0.130462      -0.045811
C        -3.240283      -1.400622       0.547228
H        -3.430300      -1.496725       1.638463
H        -3.752983      -2.231606       0.015804
H        -2.139404      -1.460345       0.358246
```

7_isobutylacrylate_16_am1_HEI

Datum	Value

Datum	Value
AM1 Energy	-0.213534
AM1 Free Energy (Quasiharmonic)	-0.029605
Number of Imaginary Frequencies	0

Frequencies (Top 3 out of 72)

```
1.      24.7370 cm-1
2.      31.5078 cm-1
3.      34.6348 cm-1
```

AM1 Molecular Geometry in Cartesian Coordinates

```
C         -0.124568      -0.987326       0.196901
C         -1.323215      -1.160604      -0.495896
C         -2.594531      -1.061251       0.150653
O          0.132388      -0.758255       1.404359
O          1.013207      -1.117091      -0.629480
C          2.287818      -0.932496      -0.047502
C          2.829847       0.460831      -0.384035
C          4.340914       0.463593      -0.316794
C          2.245640       1.496270       0.551374
H         -1.285361      -1.350698      -1.571038
H         -2.525898      -1.140466       1.260615
H         -3.352117      -1.778465      -0.249172
H          2.265754      -1.085048       1.063142
H          2.937358      -1.716433      -0.526094
H          2.511179       0.707867      -1.435678
H          4.688361       0.148405       0.696753
H          4.769013      -0.240314      -1.070554
H          4.737157       1.487147      -0.522248
H          1.138147       1.345102       0.626545
H          2.678685       1.390545       1.575091
H          2.456946       2.527228       0.180156
S         -3.526480       0.525546      -0.141481
C         -2.281682       1.759356      -0.204617
H         -1.274102       1.275313      -0.250093
H         -2.431473       2.386152      -1.110438
H         -2.336067       2.407465       0.697183
```

7_isobutylacrylate_17_reopt_am1_HEI

Datum	Value

Datum	Value
AM1 Energy	-0.211393
AM1 Free Energy (Quasiharmonic)	-0.027833
Number of Imaginary Frequencies	0

Frequencies (Top 3 out of 72)

```
1.      18.5304 cm-1
2.      27.8448 cm-1
3.      34.1081 cm-1
```

AM1 Molecular Geometry in Cartesian Coordinates

```
C         -0.054573        1.041468        0.198829
C          1.086301        0.804286        0.966447
C          1.713597       -0.467457        1.139069
O         -0.685489        2.106522       -0.015040
O         -0.597500       -0.095401       -0.433441
C         -1.792135        0.054308       -1.174084
C         -3.009391       -0.448598       -0.391405
C         -3.312937        0.476353        0.765913
C         -2.788046       -1.865250        0.089594
H          1.529905        1.681311        1.447577
H          2.118547       -0.623981        2.169013
H          1.063818       -1.323549        0.843518
H         -1.955613        1.120392       -1.482996
H         -1.645373       -0.590096       -2.084373
H         -3.885795       -0.441233       -1.099773
H         -2.393980        0.611356        1.389741
H         -3.615682        1.481839        0.386339
H         -4.133955        0.064474        1.399637
H         -1.861719       -1.909950        0.713898
H         -3.655084       -2.216824        0.698500
H         -2.658796       -2.553903       -0.779595
S          3.286132       -0.734823        0.173725
C          3.093833        0.240241       -1.270703
H          3.997277        0.872963       -1.409582
H          2.968908       -0.412370       -2.162127
H          2.193437        0.895670       -1.163483
```

7_isobutylacrylate_18_reopt_am1_HEI

Datum	Value

Datum	Value
AM1 Energy	-0.208569
AM1 Free Energy (Quasiharmonic)	-0.02636
Number of Imaginary Frequencies	0

Frequencies (Top 3 out of 72)

```
1.      19.7007 cm-1
2.      25.9521 cm-1
3.      39.6052 cm-1
```

AM1 Molecular Geometry in Cartesian Coordinates

```
C       -0.025621       1.620132       -0.056012
C        1.337837       1.561994        0.240370
C        2.002847       0.436584        0.813550
O       -0.714867       2.537043       -0.567340
O       -0.776023       0.488192        0.317361
C       -2.158103       0.524279       -0.005819
C       -2.729705      -0.856400        0.319883
C       -2.221750      -1.887382       -0.663586
C       -4.241402      -0.799008        0.314376
H        1.927016       2.442006       -0.033949
H        2.789381       0.710776        1.557862
H        1.316462      -0.322325        1.254364
H       -2.662730       1.316076        0.608619
H       -2.295529       0.771279       -1.090873
H       -2.376912      -1.143435        1.350086
H       -2.607573      -1.675773       -1.689793
H       -1.104529      -1.854318       -0.691249
H       -2.551523      -2.911339       -0.365322
H       -4.616822      -0.465208       -0.683063
H       -4.669127      -1.806876        0.533604
H       -4.608199      -0.080781        1.086592
S        2.932968      -0.410495       -0.535912
C        3.788192      -1.705330        0.272803
H        3.083965      -2.432841        0.735307
H        4.406455      -2.235383       -0.485077
H        4.455528      -1.315039        1.073920
```

7_isobutylacrylate_19_am1_HEI_reopt

Datum	Value

Datum	Value
AM1 Energy	-0.208959
AM1 Free Energy (Quasiharmonic)	-0.026827
Number of Imaginary Frequencies	0

Frequencies (Top 3 out of 72)

```
1.      17.7746 cm-1
2.      28.7232 cm-1
3.      38.3194 cm-1
```

AM1 Molecular Geometry in Cartesian Coordinates

```
C         -0.051803      -0.644463       0.150248
C         -1.127178      -0.148865       0.891126
C         -2.466671      -0.590299       0.659579
O          0.043414      -1.537702      -0.725798
O          1.181464      -0.049911       0.493321
C          2.316579      -0.530156      -0.210508
C          3.518836       0.285196       0.267906
C          3.436602       1.706038      -0.244515
C          4.800704      -0.379946      -0.182709
H         -0.940925       0.627715       1.636034
H         -3.059157      -0.740002       1.594354
H         -2.533622      -1.499615       0.018258
H          2.463812      -1.619792       0.013465
H          2.162443      -0.410146      -1.314708
H          3.494185       0.310853       1.393479
H          3.520155       1.728297      -1.357755
H          2.452085       2.148267       0.045770
H          4.258476       2.327566       0.185031
H          4.882333      -1.405212       0.251863
H          4.826408      -0.466634      -1.295838
H          5.684938       0.217608       0.145729
S         -3.350708       0.761535      -0.226994
C         -4.985506       0.164263      -0.408238
H         -5.450756      -0.057538       0.578563
H         -5.583714       0.949991      -0.920425
H         -5.013731      -0.765637      -1.019430
```

7_isobutylacrylate_1_am1_HEI_reopt

Datum	Value

Datum	Value
AM1 Energy	-0.212914
AM1 Free Energy (Quasiharmonic)	-0.029285
Number of Imaginary Frequencies	0

Frequencies (Top 3 out of 72)

```
1.      19.1516 cm-1
2.      38.7064 cm-1
3.      48.4052 cm-1
```

AM1 Molecular Geometry in Cartesian Coordinates

```
C        -0.302540      1.561418      0.243327
C        -1.653792      1.440819     -0.084330
C        -2.193224      0.509075     -1.021224
O         0.283020      2.341397      1.034752
O         0.560299      0.676379     -0.432970
C         1.933701      0.728370     -0.083455
C         2.540317     -0.632499     -0.432580
C         2.046700     -1.694318      0.525148
C         4.050019     -0.541504     -0.411736
H        -2.336878      2.104312      0.454676
H        -3.085377      0.899375     -1.569084
H        -1.436719      0.113540     -1.737487
H         2.423993      1.540303     -0.683794
H         2.064576      0.955656      1.007014
H         2.202683     -0.906476     -1.471405
H         2.423282     -1.498744      1.557958
H         0.927624     -1.684157      0.547334
H         2.396569     -2.704960      0.205576
H         4.409291     -0.220371      0.595779
H         4.502155     -1.534774     -0.647898
H         4.407445      0.200463     -1.165702
S        -2.967085     -1.028765     -0.295069
C        -1.929429     -1.435694      1.058570
H        -1.229700     -0.586707      1.261550
H        -2.554221     -1.623227      1.958606
H        -1.338417     -2.350207      0.831407
```

7_isobutylacrylate_1_am1

Datum	Value

Datum	Value
AM1 Energy	-0.1392
AM1 Free Energy (Quasiharmonic)	0.010338
Number of Imaginary Frequencies	0

Frequencies (Top 3 out of 57)

```
1.      41.4341 cm-1
2.      49.6935 cm-1
3.      61.1915 cm-1
```

AM1 Molecular Geometry in Cartesian Coordinates

```
C          1.324298      -0.204028       0.154408
C          2.573738       0.565604       0.088372
C          3.664801       0.076184      -0.500778
O          1.089224      -1.335209      -0.281121
O          0.325001       0.470380       0.804670
C         -0.945028      -0.180659       0.935797
C         -1.783113      -0.018178      -0.327842
C         -2.144610       1.433259      -0.554056
C         -3.026546      -0.874354      -0.224588
H          2.536731       1.562121       0.556913
H          4.601838       0.647467      -0.553003
H          3.686090      -0.921652      -0.964358
H         -0.795826      -1.266479       1.172205
H         -1.412213       0.353086       1.806373
H         -1.162321      -0.379109      -1.197141
H         -2.668342       1.553874      -1.532402
H         -2.818625       1.802070       0.256381
H         -1.220961       2.061266      -0.559055
H         -3.651198      -0.753353      -1.142097
H         -2.752114      -1.951803      -0.120933
H         -3.638110      -0.575819       0.660851
```

7_isobutylacrylate_20_am1_HEI

Datum	Value
AM1 Energy	-0.213477
AM1 Free Energy (Quasiharmonic)	-0.029461
Number of Imaginary Frequencies	0

Frequencies (Top 3 out of 72)

```
1.      23.4908 cm-1
2.      32.8467 cm-1
3.      35.8887 cm-1
```

AM1 Molecular Geometry in Cartesian Coordinates

```
C          0.097940       0.157968       0.023105
C          1.191668       0.398277       0.855520
C          2.333411       1.138168       0.414830
O         -0.165364       0.503710      -1.154819
O         -0.907577      -0.614383       0.644427
C         -2.081892      -0.900979      -0.088198
C         -3.218151       0.039504       0.328613
C         -4.554971      -0.652533       0.178938
C         -3.177562       1.313694      -0.486131
H          1.180822      -0.011821       1.868060
H          2.759019       1.806376       1.202851
H          2.145309       1.709902      -0.523700
H         -1.907294      -0.837958      -1.194034
H         -2.340792      -1.958457       0.195771
H         -3.064089       0.302317       1.413041
H         -4.615334      -1.540535       0.853246
H         -5.385786       0.046956       0.438575
H         -4.701834      -0.998292      -0.872821
H         -3.836703       2.092163      -0.033396
H         -2.124262       1.692711      -0.529141
H         -3.520643       1.123246      -1.531572
S          3.855612       0.130361       0.045904
C          3.240104      -1.400542      -0.547473
H          3.752619      -2.231673      -0.016099
H          2.139178      -1.460118      -0.358642
H          3.430246      -1.496553      -1.638694
```

7_isobutylacrylate_21_am1_HEI_reopt

Datum	Value
AM1 Energy	-0.211853
AM1 Free Energy (Quasiharmonic)	-0.028319
Number of Imaginary Frequencies	0

Frequencies (Top 3 out of 72)

```
 1.      22.3017 cm-1
 2.      29.7033 cm-1
 3.      35.8978 cm-1
```

AM1 Molecular Geometry in Cartesian Coordinates

```
C          0.006444      -0.086753      -0.183758
C         -0.985354      -0.405664       0.744845
C         -2.135068      -1.179439       0.392279
O          0.163676      -0.379724      -1.393928
O          1.034814       0.712840       0.363529
C          2.124198       1.060544      -0.466264
C          3.356873       0.197503      -0.178796
C          3.120057      -1.227876      -0.625033
C          3.720372       0.252832       1.288101
H         -0.888607      -0.029864       1.766076
H         -2.450438      -1.893337       1.191974
H         -2.020965      -1.708620      -0.582416
H          1.855713       0.983181      -1.553058
H          2.352944       2.130445      -0.204108
H          4.209489       0.627235      -0.777460
H          2.185915      -1.619797      -0.150152
H          2.978199      -1.266183      -1.731944
H          3.980667      -1.880528      -0.344572
H          3.976056       1.298178       1.585374
H          2.851136      -0.085689       1.903736
H          4.596673      -0.404317       1.503726
S         -3.727079      -0.228017       0.218730
C         -3.238258       1.349896      -0.369344
H         -3.727951       2.136922       0.244334
H         -2.127234       1.451953      -0.288913
H         -3.541841       1.477508      -1.431271
```

7_isobutylacrylate_22_am1_HEI

Datum	Value
AM1 Energy	-0.20925
AM1 Free Energy (Quasiharmonic)	-0.026027
Number of Imaginary Frequencies	0

Frequencies (Top 3 out of 72)

```
   1.      15.1042 cm-1
   2.      32.1771 cm-1
   3.      41.4179 cm-1
```

AM1 Molecular Geometry in Cartesian Coordinates

```
C         -0.251031        1.036795        0.102987
C         -0.955925        0.206458       -0.770894
C         -2.325527        0.456049       -1.104654
O         -0.590283        2.076655        0.716186
O          1.100639        0.779868        0.427544
C          1.711969       -0.340792       -0.180566
C          3.160968       -0.389426        0.312493
C          3.959996        0.754373       -0.271331
C          3.780228       -1.722333       -0.044742
H         -0.484210       -0.676733       -1.205249
H         -2.570271        0.236780       -2.172766
H         -2.657581        1.484248       -0.829752
H          1.175197       -1.281227        0.117571
H          1.685112       -0.241756       -1.298244
H          3.146685       -0.277985        1.433002
H          4.987054        0.779660        0.165394
H          4.048814        0.647693       -1.379278
H          3.442570        1.719518       -0.047680
H          4.842581       -1.760881        0.296915
H          3.219479       -2.556610        0.441379
H          3.758881       -1.880951       -1.150032
S         -3.566339       -0.671666       -0.297366
C         -2.945109       -0.905372        1.326375
H         -2.949373       -1.990409        1.568080
H         -1.899600       -0.513587        1.386831
H         -3.580181       -0.367796        2.063828
```

7_isobutylacrylate_2_am1

Datum	Value
AM1 Energy	-0.1392
AM1 Free Energy (Quasiharmonic)	0.010338
Number of Imaginary Frequencies	0

Frequencies (Top 3 out of 57)

```
1.      41.4341 cm-1
2.      49.6935 cm-1
3.      61.1914 cm-1
```

AM1 Molecular Geometry in Cartesian Coordinates

```
C          -1.324298       -0.204028        0.154408
C          -2.573738        0.565605        0.088372
C          -3.664802        0.076184       -0.500778
O          -1.089225       -1.335209       -0.281121
O          -0.325001        0.470380        0.804670
C           0.945028       -0.180659        0.935797
C           1.783113       -0.018178       -0.327842
C           3.026546       -0.874355       -0.224588
C           2.144610        1.433259       -0.554056
H          -2.536731        1.562121        0.556913
H          -4.601838        0.647467       -0.553003
H          -3.686090       -0.921652       -0.964357
H           1.412213        0.353086        1.806373
H           0.795826       -1.266479        1.172205
H           1.162322       -0.379109       -1.197141
H           3.651198       -0.753353       -1.142097
H           3.638110       -0.575819        0.660852
H           2.752114       -1.951803       -0.120933
H           2.668342        1.553874       -1.532401
H           1.220961        2.061267       -0.559055
H           2.818625        1.802070        0.256382
```

7_isobutylacrylate_2_reopt3_am1_HEI

Datum	Value
AM1 Energy	-0.21279
AM1 Free Energy (Quasiharmonic)	-0.029863
Number of Imaginary Frequencies	0

Frequencies (Top 3 out of 72)

```
1.      17.7782 cm-1
2.      25.8091 cm-1
3.      38.7969 cm-1
```

AM1 Molecular Geometry in Cartesian Coordinates

```
C          0.292299       1.409685       0.170127
C          1.570891       1.158599       0.669942
C          2.024994      -0.096812       1.175731
O         -0.213639       2.460287      -0.297606
O         -0.597632       0.318749       0.204702
C         -1.911381       0.573736      -0.270671
C         -2.682196      -0.743921      -0.182198
C         -3.981604      -0.628433      -0.948225
C         -2.935478      -1.118484       1.261263
H          2.268029       2.001648       0.644595
H          2.710360       0.000659       2.053046
H          1.197655      -0.807010       1.406028
H         -2.395243       1.366641       0.357419
H         -1.866172       0.935845      -1.331609
H         -2.047912      -1.547429      -0.651852
H         -4.559454      -1.581321      -0.878454
H         -4.609324       0.196757      -0.533178
H         -3.781807      -0.411024      -2.025035
H         -1.964165      -1.141482       1.813671
H         -3.604602      -0.370518       1.750748
H         -3.417993      -2.122943       1.328082
S          3.150922      -1.069386       0.047077
C          2.686122      -0.587462      -1.573177
H          1.983127       0.281326      -1.518812
H          3.593779      -0.293339      -2.143716
H          2.187257      -1.431372      -2.097681
```

7_isobutylacrylate_3_am1_HEI

Datum	Value
AM1 Energy	-0.213176
AM1 Free Energy (Quasiharmonic)	-0.030286
Number of Imaginary Frequencies	0

Frequencies (Top 3 out of 72)

```
1.      17.6107 cm-1
2.      26.6245 cm-1
3.      41.6626 cm-1
```

AM1 Molecular Geometry in Cartesian Coordinates

```
C          -0.236485       -0.768383       -0.007012
C          -1.305542       -0.556379       -0.878830
C          -2.631782       -0.993722       -0.574506
O          -0.139114       -1.345480        1.103359
O           0.983622       -0.241548       -0.483159
C           2.115301       -0.455773        0.346564
C           3.317141        0.168489       -0.363787
C           4.598127       -0.297891        0.292192
C           3.220429        1.677985       -0.350032
H          -1.121818       -0.020742       -1.813041
H          -2.666851       -1.729478        0.262318
H          -3.185981       -1.382356       -1.463458
H           1.948752        0.019627        1.348450
H           2.271528       -1.556863        0.495354
H           3.304965       -0.179093       -1.434897
H           4.612214       -0.011351        1.371504
H           4.690784       -1.408368        0.221858
H           5.482103        0.165816       -0.208155
H           2.239354        1.990856       -0.784086
H           3.285932        2.067028        0.694555
H           4.046552        2.130703       -0.949079
S          -3.826878        0.343208       -0.062465
C          -2.837446        1.523497        0.775556
H          -3.054731        1.505586        1.865787
H          -1.756033        1.289176        0.611402
H          -3.061251        2.537418        0.378461
```

7_isobutylacrylate_3_am1

Datum	Value
AM1 Energy	-0.1375
AM1 Free Energy (Quasiharmonic)	0.01151
Number of Imaginary Frequencies	0

Frequencies (Top 3 out of 57)

```
1.      30.1834 cm-1
2.      44.3443 cm-1
3.      71.1433 cm-1
```

AM1 Molecular Geometry in Cartesian Coordinates

```
C          -1.397278        0.266866        0.000036
C          -2.334627       -0.863310        0.000098
C          -3.654426       -0.675570       -0.000018
O          -1.640581        1.477766       -0.000107
O          -0.087017       -0.130771        0.000169
C           0.894811        0.920616        0.000083
C           2.271749        0.267168       -0.000078
C           2.480613       -0.564179       -1.246714
C           2.480952       -0.564091        1.246561
H          -1.867325       -1.861065        0.000247
H          -4.360275       -1.517526        0.000029
H          -4.106241        0.327881       -0.000166
H           0.743755        1.550448       -0.915265
H           0.743938        1.550393        0.915497
H           3.017908        1.113199       -0.000206
H           3.501749       -1.015229       -1.244399
H           1.725606       -1.386010       -1.292839
H           2.371137        0.068930       -2.159774
H           3.502118       -1.015069        1.244040
H           2.371640        0.069066        2.159608
H           1.726014       -1.385973        1.292913
```

7_isobutylacrylate_4_am1_HEI

Datum	Value
AM1 Energy	-0.212829
AM1 Free Energy (Quasiharmonic)	-0.029871
Number of Imaginary Frequencies	0

Frequencies (Top 3 out of 72)

```
1.      17.5501 cm-1
2.      25.7708 cm-1
3.      38.8576 cm-1
```

AM1 Molecular Geometry in Cartesian Coordinates

```
C          -0.265342        1.481332       -0.097672
C          -1.572690        1.349611        0.373059
C          -2.052770        0.274022        1.179352
O           0.262525        2.368880       -0.813248
O           0.628779        0.469615        0.302257
C           1.963269        0.599952       -0.166099
```

```
C          2.749762       -0.588716        0.387655
C          4.231928       -0.355744        0.193447
C          2.311343       -1.875076       -0.276483
H         -2.270339        2.137178        0.072296
H         -1.239622       -0.327647        1.646695
H         -2.793349        0.595222        1.952060
H          1.974696        0.603354       -1.287319
H          2.398073        1.567672        0.198974
H          2.530944       -0.665263        1.489726
H          4.815614       -1.225040        0.581263
H          4.469324       -0.225866       -0.890064
H          4.557591        0.563273        0.737548
H          2.819177       -2.752054        0.191732
H          1.204947       -1.990827       -0.166838
H          2.560491       -1.860916       -1.364819
S         -3.099676       -0.998750        0.298520
C         -2.566052       -0.944716       -1.370457
H         -2.029814       -1.882544       -1.633402
H         -3.450006       -0.835468       -2.035723
H         -1.880032       -0.073028       -1.517326
```

7_isobutylacrylate_4_am1

Datum	Value
AM1 Energy	-0.138514
AM1 Free Energy (Quasiharmonic)	0.010869
Number of Imaginary Frequencies	0

Frequencies (Top 3 out of 57)

```
1.      34.1150 cm-1
2.      44.8433 cm-1
3.      70.8052 cm-1
```

AM1 Molecular Geometry in Cartesian Coordinates

```
C         -1.335133       -0.624328       -0.074587
C         -2.692616       -0.122573       -0.322418
C         -3.134130        1.068389        0.079064
O         -0.870221       -1.714139       -0.420386
O         -0.531620        0.236067        0.626659
C          0.808536       -0.184211        0.912672
C          1.732408        0.065942       -0.274470
C          1.873445        1.547102       -0.547822
```

```
C          3.082007      -0.564313      -0.007493
H         -3.321446      -0.836644      -0.881168
H         -4.159550       1.405641      -0.127717
H         -2.503665       1.777017       0.636089
H          1.090596       0.456014       1.791046
H          0.816778      -1.269036       1.196056
H          1.270216      -0.428933      -1.176079
H          0.865266       2.010113      -0.676808
H          2.390869       2.056661       0.300562
H          2.468622       1.714302      -1.477258
H          3.543746      -0.131964       0.912831
H          3.768219      -0.377138      -0.868153
H          2.977979      -1.667509       0.130740
```

7_isobutylacrylate_5_am1

Datum	Value
AM1 Energy	-0.138514
AM1 Free Energy (Quasiharmonic)	0.010869
Number of Imaginary Frequencies	0

Frequencies (Top 3 out of 57)

```
1.      34.1146 cm-1
2.      44.8429 cm-1
3.      70.8071 cm-1
```

AM1 Molecular Geometry in Cartesian Coordinates

```
C          1.335129      -0.624326      -0.074588
C          2.692614      -0.122577      -0.322420
C          3.134135       1.068380       0.079066
O          0.870210      -1.714134      -0.420390
O          0.531622       0.236070       0.626663
C         -0.808535      -0.184205       0.912677
C         -1.732406       0.065941      -0.274469
C         -1.873440       1.547099      -0.547832
C         -3.082006      -0.564311      -0.007488
H          3.321439      -0.836650      -0.881175
H          4.159557       1.405628      -0.127716
H          2.503676       1.777009       0.636096
H         -0.816778      -1.269028       1.196068
H         -1.090596       0.456027       1.791046
H         -1.270212      -0.428942      -1.176073
```

```
H          -2.468611       1.714292       -1.477272
H          -2.390870       2.056664        0.300545
H          -0.865260       2.010108       -0.676814
H          -3.768217      -0.377140       -0.868150
H          -2.977980      -1.667506        0.130751
H          -3.543745      -0.131956        0.912833
```

7_isobutylacrylate_5_reopt2_am1_HEI_reopt

Datum	Value
AM1 Energy	-0.213827
AM1 Free Energy (Quasiharmonic)	-0.029359
Number of Imaginary Frequencies	0

Frequencies (Top 3 out of 72)

```
1.      32.4226 cm-1
2.      41.1468 cm-1
3.      60.3505 cm-1
```

AM1 Molecular Geometry in Cartesian Coordinates

```
C           0.091111      -1.400213        0.272546
C           1.485424      -1.447612        0.259313
C           2.321610      -0.806519       -0.704209
O          -0.739115      -1.931980        1.051486
O          -0.496034      -0.644900       -0.762595
C          -1.908365      -0.581225       -0.803717
C          -2.458387       0.460485        0.171937
C          -3.970074       0.446957        0.146611
C          -1.923625       1.837562       -0.149094
H           1.952925      -2.003119        1.078006
H           3.250757      -1.382562       -0.935625
H           1.786293      -0.536010       -1.643454
H          -2.140354      -0.286389       -1.862935
H          -2.358212      -1.582890       -0.573574
H          -2.101103       0.161631        1.199433
H          -4.350060       0.692594       -0.874659
H          -4.354015      -0.561508        0.433749
H          -4.377841       1.199688        0.863526
H          -2.262460       2.578450        0.614113
H          -0.804770       1.809543       -0.158762
H          -2.278760       2.175598       -1.152330
S           3.114699       0.793695       -0.161561
```

```
C          1.893693       1.567167       0.831156
H          1.075558       0.837109       1.054221
H          2.355225       1.908032       1.783191
H          1.464509       2.446134       0.301707
```

7_isobutylacrylate_6_am1

Datum	Value
AM1 Energy	-0.136809
AM1 Free Energy (Quasiharmonic)	0.011946
Number of Imaginary Frequencies	0

Frequencies (Top 3 out of 57)

```
1.      21.2965 cm-1
2.      43.8258 cm-1
3.      63.0068 cm-1
```

AM1 Molecular Geometry in Cartesian Coordinates

```
C         -1.535515      -0.563535      -0.000019
C         -2.597972       0.449159       0.000085
C         -2.376372       1.762751      -0.000103
O         -1.670939      -1.790672       0.000004
O         -0.263649      -0.055035      -0.000140
C          0.805465      -1.017491      -0.000228
C          2.120179      -0.246360       0.000066
C          2.255390       0.600014       1.246775
C          2.255852       0.600198      -1.246469
H         -3.611298       0.012566       0.000355
H         -3.200767       2.489539       0.000012
H         -1.363371       2.191809      -0.000372
H          0.709575      -1.658120       0.915056
H          0.709750      -1.657721      -0.915807
H          2.937605      -1.023721       0.000155
H          3.232703       1.139476       1.244400
H          1.430712       1.351959       1.293237
H          2.202233      -0.040339       2.159807
H          3.233256       1.139495      -1.243747
H          2.202827      -0.039996      -2.159621
H          1.431317       1.352294      -1.293029
```

7_isobutylacrylate_6_reopt2_am1_HEI

Datum	Value
AM1 Energy	-0.213501
AM1 Free Energy (Quasiharmonic)	-0.029693
Number of Imaginary Frequencies	0

Frequencies (Top 3 out of 72)

```
1.      25.1123 cm-1
2.      28.6981 cm-1
3.      44.6081 cm-1
```

AM1 Molecular Geometry in Cartesian Coordinates

```
C         0.066229        0.692909       -0.708954
C         1.287494        0.252276       -1.219690
C         1.988262       -0.914851       -0.787951
O        -0.628262        1.697554       -1.004606
O        -0.500813       -0.121820        0.291452
C        -1.740830        0.284615        0.837570
C        -2.914621       -0.155093       -0.039277
C        -4.207017        0.420016        0.494542
C        -2.988404       -1.662761       -0.123892
H         1.733479        0.877480       -1.999269
H         1.357071       -1.615839       -0.194576
H         2.496697       -1.459322       -1.621105
H        -1.784545       -0.227850        1.836539
H        -1.770678        1.398007        0.972500
H        -2.721410        0.260196       -1.070066
H        -4.391394        0.073108        1.540183
H        -4.163876        1.535822        0.494420
H        -5.067665        0.097459       -0.139593
H        -3.246603       -2.102303        0.869827
H        -3.764593       -1.977948       -0.861703
H        -1.996226       -2.066246       -0.443159
S         3.473996       -0.613111        0.299764
C         3.116584        0.882387        1.141985
H         2.933813        0.682911        2.220402
H         3.981005        1.574514        1.043928
H         2.207409        1.356933        0.694684
```

7_isobutylacrylate_7_am1

Datum	Value
AM1 Energy	-0.138224
AM1 Free Energy (Quasiharmonic)	0.010482
Number of Imaginary Frequencies	0

Frequencies (Top 3 out of 57)

```
1.      25.2871 cm-1
2.      41.4666 cm-1
3.      58.4299 cm-1
```

AM1 Molecular Geometry in Cartesian Coordinates

```
C        -1.592057      -0.609504       0.103870
C        -2.819726       0.180990      -0.044615
C        -2.842688       1.468882      -0.384221
O        -1.502831      -1.801723       0.411897
O        -0.434273       0.082716      -0.133557
C         0.790667      -0.659843       0.001749
C         1.923170       0.318237      -0.292712
C         3.193461      -0.457241      -0.568823
C         2.115783       1.280500       0.859023
H        -3.735226      -0.401892       0.154582
H        -3.785601       2.024513      -0.484882
H        -1.927250       2.045747      -0.581922
H         0.853866      -1.070636       1.042385
H         0.777171      -1.507368      -0.732168
H         1.646421       0.908939      -1.211436
H         3.465024      -1.092688       0.308448
H         3.064639      -1.118251      -1.459444
H         4.035224       0.248737      -0.768269
H         2.435340       0.735068       1.779581
H         2.897849       2.035661       0.605494
H         1.157709       1.812087       1.075282
```

7_isobutylacrylate_7_reopt_am1_HEI

Datum	Value
AM1 Energy	-0.213176
AM1 Free Energy (Quasiharmonic)	-0.030285
Number of Imaginary Frequencies	0

Frequencies (Top 3 out of 72)

```
1.      17.6442 cm-1
2.      26.6172 cm-1
3.      41.6175 cm-1
```

AM1 Molecular Geometry in Cartesian Coordinates

```
C          0.236542       -0.768195        0.006851
C          1.305510       -0.556803        0.878917
C          2.631779       -0.993871        0.574353
O          0.139345       -1.344360       -1.104030
O         -0.983680       -0.241919        0.483309
C         -2.115227       -0.455529       -0.346762
C         -3.317155        0.168302        0.363823
C         -4.598087       -0.297973       -0.292331
C         -3.220619        1.677814        0.350659
H          1.121719       -0.021727        1.813437
H          2.666893       -1.729122       -0.262909
H          3.186059       -1.382904        1.463078
H         -1.948450        0.020551       -1.348289
H         -2.271469       -1.556517       -0.496326
H         -3.304937       -0.179693        1.434804
H         -4.612181       -0.011084       -1.371553
H         -4.690649       -1.408483       -0.222358
H         -5.482112        0.165496        0.208155
H         -2.239529        1.990619        0.784731
H         -3.286285        2.067272       -0.693766
H         -4.046725        2.130198        0.949987
S          3.826792        0.343508        0.063142
C          2.837504        1.522883       -0.776294
H          3.060701        2.537193       -0.379843
H          3.055426        1.504246       -1.866383
H          1.756075        1.288303       -0.612568
```

7_isobutylacrylate_8_am1_HEI_reopt

Datum	Value
AM1 Energy	-0.21395
AM1 Free Energy (Quasiharmonic)	-0.030183
Number of Imaginary Frequencies	0

Frequencies (Top 3 out of 72)

```
1.      24.3809 cm-1
2.      34.5613 cm-1
3.      47.1808 cm-1
```

AM1 Molecular Geometry in Cartesian Coordinates

```
C          0.088814        0.098434        0.120166
C          1.177566        0.899439       -0.226140
C          2.308010        1.058420        0.634746
O         -0.181297       -0.578364        1.142368
O         -0.911412        0.060137       -0.876577
C         -2.065206       -0.712297       -0.609606
C         -3.079189        0.060405        0.235901
C         -4.229847       -0.841282        0.621770
C         -3.574960        1.283621       -0.501325
H          1.171329        1.402475       -1.195896
H          2.112905        0.714405        1.677142
H          2.722766        2.095980        0.634663
H         -2.491683       -0.935174       -1.625026
H         -1.793069       -1.667235       -0.087063
H         -2.537225        0.390213        1.168584
H         -4.747289       -1.232340       -0.287646
H         -3.858691       -1.709167        1.218355
H         -4.974798       -0.280292        1.235943
H         -2.701391        1.891981       -0.841628
H         -4.173228        0.989693       -1.397460
H         -4.218247        1.909359        0.162618
S          3.847129        0.139801        0.125366
C          3.256403       -1.312653       -0.659405
H          2.156184       -1.220123       -0.838862
H          3.780605       -1.442826       -1.631042
H          3.449897       -2.202476       -0.021506
```

7_isobutylacrylate_8_am1

Datum	Value
AM1 Energy	-0.137869
AM1 Free Energy (Quasiharmonic)	0.011569
Number of Imaginary Frequencies	0

Frequencies (Top 3 out of 57)

```
```

```
1.      23.8069 cm-1
2.      43.7627 cm-1
3.      77.9295 cm-1
```

AM1 Molecular Geometry in Cartesian Coordinates

```
C         -1.376338        0.471636       -0.267850
C         -2.717759        0.122524        0.218286
C         -3.093882       -1.110216        0.554943
O         -0.972519        1.593342       -0.586827
O         -0.521651       -0.593057       -0.372266
C          0.814723       -0.359910       -0.836287
C          1.778418       -0.321416        0.347375
C          3.146414       -0.796189       -0.093581
C          1.858870        1.071199        0.934197
H         -3.394078        0.992220        0.279253
H         -4.109272       -1.327667        0.915097
H         -2.416205       -1.974368        0.492396
H          1.018963       -1.252560       -1.488204
H          0.872932        0.582029       -1.440028
H          1.387616       -1.024236        1.136969
H          3.101011       -1.854905       -0.445455
H          3.533386       -0.161090       -0.926680
H          3.864811       -0.735757        0.759084
H          2.365957        1.768675        0.224472
H          2.434617        1.056959        1.890106
H          0.830953        1.462646        1.133236
```

7_isobutylacrylate_9_reopt_am1_HEI_reopt

Datum	Value
AM1 Energy	-0.21186
AM1 Free Energy (Quasiharmonic)	-0.027182
Number of Imaginary Frequencies	0

Frequencies (Top 3 out of 72)

```
1.      32.3257 cm-1
2.      46.2902 cm-1
3.      62.7763 cm-1
```

AM1 Molecular Geometry in Cartesian Coordinates

```
C          0.071843       1.479740       0.158521
C         -1.323202       1.505899       0.155938
C         -2.160122       0.782981      -0.747218
O          0.897710       2.085226       0.886134
O          0.656223       0.653993      -0.823448
C          2.066518       0.591521      -0.895507
C          2.617412      -0.671045      -0.225356
C          1.900793      -1.904094      -0.728353
C          2.511554      -0.562157       1.279459
H         -1.790694       2.115863       0.935017
H         -3.098351       1.329076      -1.012597
H         -1.629909       0.448002      -1.668411
H          2.291600       0.553068      -1.997220
H          2.542293       1.499550      -0.439594
H          3.706215      -0.746203      -0.506495
H          0.808504      -1.825791      -0.497021
H          2.019650      -1.997743      -1.834283
H          2.309747      -2.823282      -0.244644
H          1.454856      -0.328131       1.565076
H          2.825977      -1.514087       1.769615
H          3.155474       0.269883       1.652998
S         -2.929576      -0.778209      -0.075299
C         -1.674546      -1.481565       0.927207
H         -0.841689      -0.745453       1.057318
H         -2.098018      -1.739535       1.922083
H         -1.275401      -2.405311       0.453322
```

8_ethylmethacrylate_10_reopt_am1_HEI_reopt

Datum	Value
AM1 Energy	-0.205588
AM1 Free Energy (Quasiharmonic)	-0.050228
Number of Imaginary Frequencies	0

Frequencies (Top 3 out of 63)

```
1.      15.6004 cm-1
2.      28.4583 cm-1
3.      45.9895 cm-1
```

AM1 Molecular Geometry in Cartesian Coordinates

```
```

```
C         -0.740093      -0.039724      -0.204234
C          0.366071       0.821131      -0.235393
C          0.406150       2.070501       0.537895
C          1.502048       0.484167      -1.054030
O         -0.954918      -1.130735      -0.788839
O         -1.804392       0.386405       0.614072
C         -2.945832      -0.450697       0.679471
C         -3.873751      -0.214348      -0.492227
H          1.344891       2.132427       1.147960
H          0.394492       2.965415      -0.139860
H         -0.465946       2.156528       1.231335
H          1.990721       1.380850      -1.507881
H          1.252420      -0.268792      -1.837828
H         -2.644214      -1.530697       0.721372
H         -3.441510      -0.157222       1.645132
H         -4.796374      -0.831664      -0.383024
H         -4.162016       0.861591      -0.553523
H         -3.349595      -0.496680      -1.437765
C          2.283822      -1.313475       1.022615
H          1.180850      -1.145535       1.089634
H          2.750146      -1.107849       2.010611
H          2.476519      -2.372628       0.744491
S          2.972100      -0.236027      -0.178569
```

8_ethylmethacrylate_11_am1_HEI

Datum	Value
AM1 Energy	-0.201127
AM1 Free Energy (Quasiharmonic)	-0.04653
Number of Imaginary Frequencies	0

Frequencies (Top 3 out of 63)

```
1.      31.9297 cm-1
2.      36.0158 cm-1
3.      55.6455 cm-1
```

AM1 Molecular Geometry in Cartesian Coordinates

```
C         -1.031177       0.942680      -0.014550
C          0.316916       1.162045       0.310201
C          0.861347       2.521471       0.154322
C          1.184275       0.118863       0.778917
```

```
O          -1.915302       1.724637       -0.442214
O          -1.496763      -0.365703        0.210037
C          -2.852389      -0.610177       -0.139702
C          -3.109501      -2.082467        0.097185
H           0.092299       3.215406       -0.267054
H           1.199065       2.934762        1.141844
H           1.751230       2.525861       -0.526561
H           0.641144      -0.776522        1.160416
H           1.917902       0.471252        1.544200
H          -3.523759       0.026137        0.495800
H          -3.021915      -0.336361       -1.214555
H          -2.419344      -2.701342       -0.523926
H          -4.164077      -2.325788       -0.173664
H          -2.939243      -2.340519        1.169237
C           3.233836      -1.704146        0.143490
H           3.854598      -1.274571        0.961813
H           3.906335      -2.128272       -0.634618
H           2.617003      -2.524946        0.573775
S           2.235340      -0.482886       -0.614279
```

8_ethylmethacrylate_12_reopt2_am1_HEI

Datum	Value
AM1 Energy	-0.205894
AM1 Free Energy (Quasiharmonic)	-0.050127
Number of Imaginary Frequencies	0

Frequencies (Top 3 out of 63)

```
1.      17.7858 cm-1
2.      40.4684 cm-1
3.      64.8397 cm-1
```

AM1 Molecular Geometry in Cartesian Coordinates

```
C           0.818398       0.847456       -0.033231
C          -0.552633       1.088232       -0.209687
C          -1.167030       2.200579        0.534807
C          -1.380300       0.302264       -1.082021
O           1.673147       1.434819        0.674831
O           1.338729      -0.221312       -0.786560
C           2.728490      -0.476470       -0.677852
C           3.044101      -1.313564        0.542475
H          -0.404150       2.749311        1.140908
```

```
H          -1.651536       2.932436       -0.163611
H          -1.965337       1.828022        1.228648
H          -0.793792      -0.285181       -1.825697
H          -2.166947       0.907600       -1.596140
H           3.306890       0.484572       -0.647593
H           2.974514      -1.038438       -1.620147
H           2.452977      -2.259627        0.531979
H           4.130762      -1.564307        0.566115
H           2.777669      -0.737778        1.462155
C          -1.632912      -1.422368        1.198943
H          -2.333787      -1.347000        2.058675
H          -1.262392      -2.468027        1.124674
H          -0.767006      -0.734155        1.364437
S          -2.471348      -0.970766       -0.273199
```

8_ethylmethacrylate_1_am1_reopt

Datum	Value
AM1 Energy	-0.131525
AM1 Free Energy (Quasiharmonic)	-0.010168
Number of Imaginary Frequencies	0

Frequencies (Top 3 out of 48)

```
1.      28.3179 cm-1
2.      55.2522 cm-1
3.      98.8160 cm-1
```

AM1 Molecular Geometry in Cartesian Coordinates

```
C          -0.367699      -0.535342        0.000003
C          -1.634983       0.221165       -0.000009
C          -1.533535       1.698268        0.000022
C          -2.796385      -0.445161       -0.000044
O          -0.213440      -1.761177       -0.000056
O           0.752275       0.251343        0.000083
C           2.015293      -0.439720        0.000148
C           3.080279       0.629515       -0.000141
H          -2.544572       2.172984        0.000046
H          -0.970866       2.042818        0.903925
H          -0.970891       2.042857       -0.903882
H          -3.764252       0.073723       -0.000051
H          -2.837325      -1.544349       -0.000068
H           2.070208      -1.087854        0.913461
```

```
H          2.070109       -1.088254       -0.912888
H          4.084134        0.141690       -0.000172
H          2.987375        1.276210       -0.905080
H          2.987573        1.276499        0.904612
```

8_ethylmethacrylate_1_reopt_am1_HEI

Datum	Value
AM1 Energy	-0.205365
AM1 Free Energy (Quasiharmonic)	-0.050376
Number of Imaginary Frequencies	0

Frequencies (Top 3 out of 63)

```
1.      20.6391 cm-1
2.      31.5845 cm-1
3.      46.9909 cm-1
```

AM1 Molecular Geometry in Cartesian Coordinates

```
C         -0.651924       -0.104553       -0.556650
C          0.424402        0.780377       -0.392495
C          0.299235        2.005790        0.409499
C          1.689547        0.475046       -1.007058
O         -0.756350       -1.174265       -1.205827
O         -1.836261        0.269354        0.106533
C         -2.952249       -0.589005       -0.089092
C         -4.106785        0.005737        0.687473
H          0.824832        2.862906       -0.086875
H         -0.771769        2.292875        0.554505
H          0.759785        1.879623        1.424858
H          2.225151        1.382695       -1.378605
H          1.595519       -0.285834       -1.817025
H         -3.190489       -0.655138       -1.183613
H         -2.703534       -1.619350        0.279407
H         -5.009523       -0.638460        0.566000
H         -3.851441        0.076460        1.771339
H         -4.337843        1.032345        0.316497
C          2.149069       -1.174596        1.287567
H          1.052083       -0.974868        1.206179
H          2.499650       -0.904270        2.307357
H          2.338081       -2.256449        1.114119
S          3.012663       -0.209127        0.105297
```

8_ethylmethacrylate_2_am1_HEI

Datum	Value
AM1 Energy	-0.205745
AM1 Free Energy (Quasiharmonic)	-0.050626
Number of Imaginary Frequencies	0

Frequencies (Top 3 out of 63)

```
1.      18.0684 cm-1
2.      38.3243 cm-1
3.      59.5883 cm-1
```

AM1 Molecular Geometry in Cartesian Coordinates

```
C          0.699674        0.988181        0.063309
C         -0.624854        1.015353       -0.399245
C         -1.445185        2.200181       -0.095777
C         -1.213163       -0.056666       -1.152536
O          1.362024        1.826650        0.722952
O          1.428957       -0.166167       -0.274977
C          2.778190       -0.201273        0.171089
C          3.359449       -1.520009       -0.289918
H         -0.852301        2.968740        0.459459
H         -1.834615        2.670986       -1.036451
H         -2.335050        1.924534        0.528426
H         -0.455752       -0.736723       -1.606338
H         -1.931670        0.301788       -1.930113
H          3.341435        0.665847       -0.264822
H          2.807767       -0.113336        1.289387
H          4.420258       -1.598284        0.046563
H          3.322481       -1.594044       -1.402611
H          2.776530       -2.371358        0.134912
C         -1.759459       -1.179833        1.432636
H         -0.992790       -0.373586        1.546845
H         -1.305284       -2.159246        1.698491
H         -2.611653       -0.978338        2.117615
S         -2.340320       -1.210403       -0.221407
```

8_ethylmethacrylate_2_am1_reopt

Datum	Value
AM1 Energy	-0.131638
AM1 Free Energy (Quasiharmonic)	-0.009679
Number of Imaginary Frequencies	0

Frequencies (Top 3 out of 48)

```
1.      30.9608 cm-1
2.      66.2601 cm-1
3.      94.7362 cm-1
```

AM1 Molecular Geometry in Cartesian Coordinates

```
C       -0.211723     -0.386726     -0.167803
C       -1.608458      0.033754      0.061462
C       -1.870752      1.490133      0.109099
C       -2.557371     -0.898607      0.213300
O        0.229515     -1.537854     -0.244611
O        0.663738      0.655581     -0.315097
C        2.044647      0.336745     -0.541110
C        2.743866      0.008468      0.754937
H       -2.953044      1.700466      0.287874
H       -1.269668      1.960359      0.927589
H       -1.563319      1.965681     -0.856154
H       -3.609316     -0.634744      0.385926
H       -2.329350     -1.973876      0.172364
H        2.125466     -0.511791     -1.269696
H        2.443404      1.282347     -0.998512
H        3.833156     -0.140712      0.563873
H        2.615110      0.836090      1.492607
H        2.320279     -0.928235      1.192486
```

8_ethylmethacrylate_3_am1_reopt

Datum	Value
AM1 Energy	-0.131485
AM1 Free Energy (Quasiharmonic)	-0.009882
Number of Imaginary Frequencies	0

Frequencies (Top 3 out of 48)

```
1.      19.3063 cm-1
2.      64.6572 cm-1
3.      69.4035 cm-1
```

AM1 Molecular Geometry in Cartesian Coordinates

```
C         -0.208438      -0.301546      -0.125805
C         -1.570122       0.239049       0.057507
C         -2.638681      -0.788687       0.087633
C         -1.799528       1.549948       0.190604
O          0.150598      -1.480709      -0.065091
O          0.737644       0.650466      -0.402203
C          2.090316       0.210813      -0.590258
C          2.779366      -0.003279       0.734850
H         -3.613520      -0.344947       0.404189
H         -2.762464      -1.240247      -0.928789
H         -2.363912      -1.609428       0.797224
H         -2.812240       1.949459       0.338226
H         -0.990945       2.294010       0.159114
H          2.104013      -0.724862      -1.208183
H          2.548812       1.062082      -1.162533
H          3.853195      -0.252319       0.561068
H          2.717768       0.916359       1.364236
H          2.295876      -0.845954       1.286613
```

8_ethylmethacrylate_3_reopt_am1_HEI

Datum	Value
AM1 Energy	-0.205589
AM1 Free Energy (Quasiharmonic)	-0.049979
Number of Imaginary Frequencies	0

Frequencies (Top 3 out of 63)

```
1.      22.9668 cm-1
2.      35.7995 cm-1
3.      41.8504 cm-1
```

AM1 Molecular Geometry in Cartesian Coordinates

```
C          0.743580        0.340734        0.493238
C         -0.445043        0.988677        0.128481
C         -0.492408        1.977912       -0.957642
C         -1.660966        0.690598        0.840198
O          0.979799       -0.513528        1.383523
O          1.878723        0.715174       -0.251840
C          3.109752        0.104174        0.093384
C          3.251644       -1.257839       -0.550539
H         -0.640326        3.015297       -0.554637
H          0.450024        1.977626       -1.558453
H         -1.348742        1.768251       -1.650120
H         -2.326844        1.580880        0.953111
H         -1.468163        0.213508        1.829539
H          3.885314        0.814834       -0.303801
H          3.210873        0.017069        1.207518
H          4.253718       -1.690047       -0.319803
H          2.456591       -1.935589       -0.154138
H          3.134954       -1.182702       -1.657452
C         -1.836041       -1.681097       -0.740736
H         -0.766793       -1.358763       -0.694433
H         -2.136810       -1.813683       -1.802731
H         -1.949460       -2.650665       -0.208403
S         -2.852851       -0.459259        0.001377
```

8_ethylmethacrylate_4_am1

Datum	Value
AM1 Energy	-0.122023
AM1 Free Energy (Quasiharmonic)	-0.000901
Number of Imaginary Frequencies	0

Frequencies (Top 3 out of 48)

```
  1.      42.7633 cm-1
  2.      51.2104 cm-1
  3.      93.9389 cm-1
```

AM1 Molecular Geometry in Cartesian Coordinates

```
C          0.424345        0.849227       -0.106357
C          1.286675       -0.354652       -0.109912
C          1.901623       -0.674584        1.200445
C          1.519679       -1.044011       -1.229431
```

```
O          0.793466       2.022451      -0.151508
O         -0.941602       0.731154      -0.030730
C         -1.478187      -0.596127       0.047813
C         -2.977796      -0.443652       0.142901
H          2.579476      -1.559259       1.121352
H          2.496466       0.201099       1.564975
H          1.107970      -0.899538       1.956629
H          2.188182      -1.915573      -1.246946
H          1.066220      -0.778204      -2.193968
H         -1.180810      -1.166911      -0.871517
H         -1.059257      -1.108928       0.953831
H         -3.445330      -1.454949       0.203740
H         -3.253129       0.144745       1.050680
H         -3.372739       0.091479      -0.753629
```

8_ethylmethacrylate_4_reopt_am1_HEI

Datum	Value
AM1 Energy	-0.205605
AM1 Free Energy (Quasiharmonic)	-0.049898
Number of Imaginary Frequencies	0

Frequencies (Top 3 out of 63)

```
1.      19.5074 cm-1
2.      45.5679 cm-1
3.      47.5816 cm-1
```

AM1 Molecular Geometry in Cartesian Coordinates

```
C         -0.745838      -0.063138      -0.221061
C          0.363458       0.794830      -0.263573
C          0.410082       2.034339       0.525209
C          1.494050       0.447470      -1.084058
O         -0.982639      -1.140414      -0.821162
O         -1.785815       0.354799       0.631925
C         -2.946031      -0.457056       0.684430
C         -3.881642      -0.162214      -0.467702
H          0.863901       2.869178      -0.070048
H         -0.612126       2.353721       0.847084
H          1.035551       1.909110       1.448269
H          1.978199       1.335547      -1.559144
H          1.241036      -0.320749      -1.851977
H         -2.669136      -1.544492       0.688551
```

```
H         -3.423316      -0.183170       1.664949
H         -4.816573      -0.762275      -0.367406
H         -4.146458       0.921231      -0.490771
H         -3.374953      -0.425095      -1.428237
C          2.306295      -1.191000       1.118271
H          1.216493      -0.965552       1.224806
H          2.833824      -0.919837       2.058558
H          2.438343      -2.278676       0.929193
S          2.973788      -0.257712      -0.208064
```

8_ethylmethacrylate_5_am1

Datum	Value
AM1 Energy	-0.121952
AM1 Free Energy (Quasiharmonic)	-7e-06
Number of Imaginary Frequencies	0

Frequencies (Top 3 out of 48)

```
1.      43.3626 cm-1
2.      62.3907 cm-1
3.      91.3185 cm-1
```

AM1 Molecular Geometry in Cartesian Coordinates

```
C          0.358645       0.891489      -0.006354
C          1.130388      -0.361685       0.154796
C          1.625731      -0.946540      -1.114450
C          1.416181      -0.860770       1.360364
O          0.808514       1.970821      -0.394425
O         -0.978506       0.973070       0.295385
C         -1.706203      -0.218631       0.594386
C         -2.212278      -0.873439      -0.668596
H          0.768881      -1.282503      -1.750782
H          2.204567      -0.175660      -1.684422
H          2.289751      -1.823994      -0.920908
H          2.033503      -1.760814       1.485242
H          1.062528      -0.398907       2.292215
H         -1.083843      -0.927396       1.200171
H         -2.561406       0.158633       1.221019
H         -2.781994      -0.137945      -1.286279
H         -1.361948      -1.260999      -1.279832
H         -2.884881      -1.724086      -0.404980
```

8_ethylmethacrylate_5_reopt_am1_HEI

Datum	Value
AM1 Energy	-0.205351
AM1 Free Energy (Quasiharmonic)	-0.050516
Number of Imaginary Frequencies	0

Frequencies (Top 3 out of 63)

```
1.      22.2570 cm-1
2.      31.8113 cm-1
3.      32.6136 cm-1
```

AM1 Molecular Geometry in Cartesian Coordinates

```
C         -0.646962       -0.083326        0.536383
C          0.418738        0.808379        0.349552
C          0.281439        2.030685       -0.455064
C          1.689165        0.532214        0.968549
O         -0.725672       -1.158217        1.181757
O         -1.851017        0.282679       -0.095043
C         -2.944151       -0.605656        0.095470
C         -4.120127       -0.024814       -0.659195
H         -0.682795        2.048080       -1.019830
H          0.315108        2.947318        0.192329
H          1.120620        2.118267       -1.193377
H          1.604075       -0.199963        1.805350
H          2.218508        1.457379        1.304150
H         -2.675215       -1.623323       -0.293423
H         -3.169745       -0.696297        1.190932
H         -5.006753       -0.691220       -0.538867
H         -4.370178        0.990146       -0.268975
H         -3.878252        0.069220       -1.744373
C          2.161647       -1.312767       -1.159735
H          1.058638       -1.177235       -1.040366
H          2.433653       -2.356115       -0.888201
H          2.444645       -1.128636       -2.218921
S          3.010170       -0.176350       -0.127244
```

8_ethylmethacrylate_6_am1_HEI

Datum	Value
AM1 Energy	-0.205964
AM1 Free Energy (Quasiharmonic)	-0.050138
Number of Imaginary Frequencies	0

Frequencies (Top 3 out of 63)

```
1.      18.7711 cm-1
2.      46.3003 cm-1
3.      58.0547 cm-1
```

AM1 Molecular Geometry in Cartesian Coordinates

```
C        0.819077       0.602482      -0.187843
C       -0.438231       0.955843       0.325179
C       -0.984146       2.280313      -0.016529
C       -1.213275       0.086623       1.166176
O        1.614304       1.233965      -0.926604
O        1.282687      -0.670502       0.193128
C        2.550040      -1.073457      -0.296299
C        3.673638      -0.511399       0.547199
H       -0.263325       2.863809      -0.641356
H       -1.945386       2.184724      -0.586032
H       -1.206001       2.874387       0.908685
H       -1.788262       0.639228       1.949395
H       -0.606548      -0.725487       1.629316
H        2.522352      -2.195267      -0.226612
H        2.678398      -0.763048      -1.366939
H        4.655891      -0.896930       0.185553
H        3.542446      -0.799154       1.617019
H        3.664813       0.603540       0.475005
C       -2.138001      -1.008789      -1.320998
H       -1.931356      -2.078243      -1.544120
H       -1.216579      -0.405671      -1.516064
H       -2.958415      -0.653317      -1.981754
S       -2.621160      -0.821623       0.354025
```

8_ethylmethacrylate_6_am1

Datum	Value
AM1 Energy	-0.122031
AM1 Free Energy (Quasiharmonic)	2.3e-05

Datum	Value
Number of Imaginary Frequencies	0

Frequencies (Top 3 out of 48)

```
1.      54.2624 cm-1
2.      64.5481 cm-1
3.      91.1780 cm-1
```

AM1 Molecular Geometry in Cartesian Coordinates

```
C         -0.388705        0.863092       -0.199515
C         -1.082049       -0.443231       -0.118030
C         -1.871183       -0.647119        1.120807
C         -1.046936       -1.317770       -1.126438
O         -0.850765        1.908633       -0.657290
O          0.880470        1.032358        0.296146
C          1.592529       -0.107539        0.781008
C          2.341859       -0.791156       -0.337665
H         -1.224963       -0.519234        2.024989
H         -2.696428        0.108122        1.171352
H         -2.321998       -1.669207        1.145913
H         -1.591269       -2.271246       -1.087593
H         -0.482708       -1.134898       -2.051173
H          2.305986        0.337531        1.528562
H          0.900333       -0.818703        1.302614
H          1.627977       -1.259629       -1.058167
H          2.969583       -0.053392       -0.893121
H          3.002759       -1.584929        0.084778
```

8_ethylmethacrylate_7_reopt_am1_HEI_reopt

Datum	Value
AM1 Energy	-0.205745
AM1 Free Energy (Quasiharmonic)	-0.050627
Number of Imaginary Frequencies	0

Frequencies (Top 3 out of 63)

```
1.      18.0680 cm-1
2.      38.3065 cm-1
```

```
3.      59.5772 cm-1
```

AM1 Molecular Geometry in Cartesian Coordinates

```
C        -0.699657        0.988204        0.063143
C         0.624862        1.015305       -0.399429
C         1.445181        2.200191       -0.096153
C         1.213169       -0.056836       -1.152550
O        -1.362012        1.826785        0.722641
O        -1.428927       -0.166216       -0.274921
C        -2.778146       -0.201254        0.171197
C        -3.359425       -1.520047       -0.289621
H         2.334959        1.924694        0.528244
H         1.834752        2.670741       -1.036895
H         0.852235        2.968902        0.458802
H         1.931703        0.301493       -1.930158
H         0.455751       -0.736935       -1.606274
H        -2.807680       -0.113170        1.289483
H        -3.341403        0.665811       -0.264804
H        -4.420224       -1.598270        0.046899
H        -3.322489       -1.594227       -1.402304
H        -2.776502       -2.371345        0.135303
C         1.759412       -1.179635        1.432800
H         2.611599       -0.978044        2.117758
H         1.305235       -2.159007        1.698801
H         0.992747       -0.373366        1.546879
S         2.340278       -1.210463       -0.221238
```

8_ethylmethacrylate_8_am1_HEI

Datum	Value
AM1 Energy	-0.200791
AM1 Free Energy (Quasiharmonic)	-0.046298
Number of Imaginary Frequencies	0

Frequencies (Top 3 out of 63)

```
1.      29.3135 cm-1
2.      33.8012 cm-1
3.      43.7940 cm-1
```

AM1 Molecular Geometry in Cartesian Coordinates

```
C          0.825259      -0.341057      -0.340645
C         -0.232504       0.572136      -0.480496
C         -0.032335       2.013889      -0.276347
C         -1.544758       0.079581      -0.801537
O          0.883039      -1.581200      -0.523783
O          2.056472       0.232703       0.029209
C          3.151022      -0.667455       0.136475
C          4.352701       0.148403       0.561048
H         -0.297275       2.327157       0.767236
H          1.033531       2.303802      -0.455206
H         -0.676788       2.608126      -0.975319
H         -1.533886      -0.964019      -1.195399
H         -2.105656       0.746213      -1.500273
H          2.912977      -1.462593       0.891626
H          3.328282      -1.164712      -0.853811
H          5.240735      -0.519348       0.662084
H          4.574076       0.938636      -0.194882
H          4.157317       0.647580       1.539632
C         -4.096927      -0.631744       0.144654
H         -4.570114       0.023303      -0.621170
H         -4.784258      -0.723861       1.014506
H         -3.948196      -1.638415      -0.306814
S         -2.586349       0.038974       0.720343
```

8_ethylmethacrylate_9_reopt2_am1_HEI

Datum	Value
AM1 Energy	-0.205598
AM1 Free Energy (Quasiharmonic)	-0.049918
Number of Imaginary Frequencies	0

Frequencies (Top 3 out of 63)

```
1.      19.6338 cm-1
2.      42.1230 cm-1
3.      46.6461 cm-1
```

AM1 Molecular Geometry in Cartesian Coordinates

```
C          0.750853       0.349814      -0.491983
C         -0.450656       0.976364      -0.128253
C         -0.520859       1.912688       1.002709
C         -1.651306       0.684001      -0.866577
```

```
O          1.019601      -0.448110      -1.423487
O          1.859183       0.681430       0.311399
C          3.112410       0.131198      -0.055734
C          3.279573      -1.272558       0.483993
H          0.492287       2.294555       1.282222
H         -1.167446       2.793737       0.751681
H         -0.965026       1.425300       1.910530
H         -1.437680       0.219004      -1.857535
H         -2.319129       1.572310      -0.983626
H          3.235583       0.133198      -1.171034
H          3.861615       0.828290       0.410213
H          4.298562      -1.657943       0.244470
H          3.136866      -1.287028       1.590310
H          2.513218      -1.938664       0.017540
C         -1.859932      -1.607707       0.837930
H         -0.808554      -1.229537       0.877317
H         -1.871044      -2.609227       0.355163
H         -2.259100      -1.699081       1.871511
S         -2.856307      -0.484643      -0.068410
```

---

ethylcrotonate_10_reopt_am1_HEI

Datum	Value
AM1 Energy	-0.196854
AM1 Free Energy (Quasiharmonic)	-0.041197
Number of Imaginary Frequencies	0

Frequencies (Top 3 out of 63)

```
1.      36.1406 cm-1
2.      44.8047 cm-1
3.      52.6454 cm-1
```

AM1 Molecular Geometry in Cartesian Coordinates

```
C         -1.089811      -0.044241       0.051194
C          0.052341       0.531600      -0.509965
C          1.285078       0.621280       0.220055
C          2.032726       1.915408       0.028104
O         -1.344155      -0.486947       1.197659
O         -2.181999      -0.092578      -0.841006
C         -3.387353      -0.656201      -0.357989
C         -4.205563       0.360458       0.408946
H          1.163097       0.390828       1.308247
```

```
H          2.182121       2.129541       -1.057256
H          1.448293       2.757295        0.474743
H          3.033197       1.876904        0.524943
H         -3.929631      -0.981648       -1.287704
H         -3.176810      -1.548413        0.289373
H         -4.402755       1.260561       -0.219864
H         -5.179765      -0.084770        0.720358
H         -3.637298       0.678269        1.316877
C          3.803804      -0.781143        0.486911
H          3.581839      -0.705140        1.574836
H          4.318157      -1.747780        0.288881
H          4.492230       0.050813        0.215407
S          2.334543      -0.750404       -0.463913
H          0.016542       0.883248       -1.543003
```

---

ethylcrotonate_11_am1_HEI

Datum	Value
AM1 Energy	-0.197016
AM1 Free Energy (Quasiharmonic)	-0.041493
Number of Imaginary Frequencies	0

Frequencies (Top 3 out of 63)

```
1.      18.9487 cm-1
2.      44.0715 cm-1
3.      71.2223 cm-1
```

AM1 Molecular Geometry in Cartesian Coordinates

```
C         -0.836709       0.074763       -0.886008
C          0.062944       0.602726        0.043390
C          1.446721       0.800455       -0.280154
C          2.062288       2.048237        0.296353
O         -0.683740      -0.279017       -2.079328
O         -2.194446      -0.129824       -0.555439
C         -2.598613       0.193284        0.761318
C         -4.073377      -0.137099        0.870089
H          1.639986       0.717103       -1.378790
H          1.836320       2.136324        1.386186
H          3.172132       2.045872        0.161840
H          1.644609       2.945961       -0.222935
H         -2.007150      -0.405856        1.504948
H         -2.426969       1.285524        0.959722
```

```
H          -4.437293        0.103990        1.896609
H          -4.658648        0.453181        0.125920
H          -4.243505       -1.220747        0.666274
C           1.692692       -2.001724        0.343355
H           2.127338       -2.670108       -0.431717
H           0.630396       -1.774483        0.078115
H           1.725649       -2.519323        1.326759
S           2.609238       -0.510745        0.441385
H          -0.260852        0.861356        1.052978
```

---

ethylcrotonate_12_reopt_am1_HEI_reopt

Datum	Value
AM1 Energy	-0.197016
AM1 Free Energy (Quasiharmonic)	-0.041493
Number of Imaginary Frequencies	0

Frequencies (Top 3 out of 63)

```
1.      18.9461 cm-1
2.      44.0681 cm-1
3.      71.2196 cm-1
```

AM1 Molecular Geometry in Cartesian Coordinates

```
C           0.836705        0.074718        0.886006
C          -0.062943        0.602727       -0.043371
C          -1.446719        0.800454        0.280179
C          -2.062280        2.048253       -0.296298
O           0.683734       -0.279111        2.079310
O           2.194439       -0.129869        0.555428
C           2.598618        0.193317       -0.761306
C           4.073385       -0.137051       -0.870083
H          -1.639984        0.717080        1.378812
H          -1.644621        2.945963        0.223033
H          -1.836287        2.136383       -1.386122
H          -3.172127        2.045877       -0.161813
H           2.426969        1.285566       -0.959651
H           2.007166       -0.405785       -1.504975
H           4.658645        0.453191       -0.125876
H           4.243517       -1.220710       -0.666326
H           4.437308        0.104097       -1.896585
C          -1.692694       -2.001709       -0.343431
H          -0.630394       -1.774477       -0.078202
```

```
H          -2.127325       -2.670120        0.431626
H          -1.725673       -2.519271       -1.326854
S          -2.609242       -0.510726       -0.441388
H           0.260859        0.861403       -1.052945
```

---

ethylcrotonate_14_reopt_am1_HEI

Datum	Value
AM1 Energy	-0.200668
AM1 Free Energy (Quasiharmonic)	-0.045294
Number of Imaginary Frequencies	0

Frequencies (Top 3 out of 63)

```
1.      25.8539 cm-1
2.      44.1519 cm-1
3.      56.1580 cm-1
```

AM1 Molecular Geometry in Cartesian Coordinates

```
C           0.860656        0.864341       -0.673359
C          -0.498169        1.100529       -0.456660
C          -1.265206        0.560248        0.623225
C          -2.314940        1.485153        1.181161
O           1.625654        1.271536       -1.583199
O           1.499196        0.054936        0.284392
C           2.886514       -0.173865        0.079302
C           3.356734       -1.069980        1.204258
H          -0.628368        0.129853        1.434851
H          -2.997742        0.940954        1.879281
H          -1.821800        2.315863        1.744300
H          -2.926680        1.931759        0.360968
H           3.044908       -0.659856       -0.919701
H           3.434255        0.805537        0.084210
H           2.789587       -2.030900        1.198509
H           4.444239       -1.285859        1.080148
H           3.192716       -0.575128        2.190734
C          -1.515721       -1.680381       -1.196159
H          -2.229149       -1.825669       -2.036423
H          -1.107585       -2.669575       -0.893985
H          -0.675873       -1.021850       -1.532701
S          -2.348662       -0.932454        0.151480
H          -0.997924        1.736081       -1.194030
```

---

ethylcrotonate_1_am1_HEI

Datum	Value
AM1 Energy	-0.200831
AM1 Free Energy (Quasiharmonic)	-0.044807
Number of Imaginary Frequencies	0

Frequencies (Top 3 out of 63)

```
1.      29.4746 cm-1
2.      40.4139 cm-1
3.      59.1007 cm-1
```

AM1 Molecular Geometry in Cartesian Coordinates

```
C          0.927833      -0.771149      -0.570216
C         -0.454114      -0.944334      -0.664874
C         -1.389225      -0.697610       0.389801
C         -2.544078      -1.662750       0.452008
O          1.830850      -0.948833      -1.425783
O          1.402825      -0.351042       0.686719
C          2.804098      -0.207173       0.837069
C          3.281359       1.133428       0.322425
H         -0.897452      -0.586538       1.387546
H         -3.327585      -1.304129       1.164222
H         -3.009494      -1.789937      -0.554775
H         -2.179997      -2.661196       0.799731
H          2.968089      -0.288318       1.946486
H          3.346346      -1.037594       0.312114
H          4.373073       1.253807       0.517191
H          3.093438       1.192609      -0.777351
H          2.729291       1.964030       0.822299
C         -1.251633       2.000625      -0.652483
H         -0.380093       1.411466      -1.034014
H         -0.882421       2.816413       0.006738
H         -1.797129       2.449297      -1.511129
S         -2.332538       0.946961       0.236731
H         -0.830297      -1.278503      -1.636622
```

---

ethylcrotonate_1_am1

Datum	Value
AM1 Energy	-0.137799
AM1 Free Energy (Quasiharmonic)	-0.016604
Number of Imaginary Frequencies	0

Frequencies (Top 3 out of 48)

```
1.      41.5939 cm-1
2.      62.6763 cm-1
3.      93.7381 cm-1
```

AM1 Molecular Geometry in Cartesian Coordinates

```
C          0.018633        0.365276       -0.000004
C          1.254538       -0.422035        0.000001
C          2.455458        0.170958       -0.000002
C          3.730879       -0.568335        0.000004
O         -0.122838        1.592889       -0.000011
O         -1.108267       -0.413253       -0.000001
C         -2.365953        0.285343       -0.000007
C         -3.438474       -0.776473        0.000017
H          1.122017       -1.515251        0.000008
H          2.535574        1.274044       -0.000008
H          4.328291       -0.293704        0.906341
H          3.576515       -1.675296        0.000011
H          4.328292       -0.293715       -0.906336
H         -2.416873        0.934247        0.913064
H         -2.416881        0.934213       -0.913102
H         -4.439044       -0.282044        0.000012
H         -3.349772       -1.424012       -0.904753
H         -3.349765       -1.423977        0.904811
```

---

ethylcrotonate_2_am1

Datum	Value
AM1 Energy	-0.137914
AM1 Free Energy (Quasiharmonic)	-0.016141
Number of Imaginary Frequencies	0

Frequencies (Top 3 out of 48)

```
  1.      49.4426 cm-1
  2.      65.7857 cm-1
  3.      92.0254 cm-1
```

AM1 Molecular Geometry in Cartesian Coordinates

```
C          0.077044        0.135040       -0.218328
C         -1.251320       -0.453854       -0.024012
C         -2.346009        0.313553        0.056748
C         -3.705672       -0.221786        0.251596
O          0.388432        1.325212       -0.331476
O          1.068359       -0.808637       -0.283752
C          2.413888       -0.348716       -0.470532
C          3.017052        0.105514        0.835900
H         -1.278528       -1.552307        0.048996
H         -2.265989        1.413772       -0.022718
H         -4.147083        0.197244        1.191445
H         -4.359331        0.082537       -0.605069
H         -3.713262       -1.337101        0.323697
H          2.933529       -1.260908       -0.870876
H          2.432947        0.474801       -1.231437
H          2.947815       -0.701926        1.603323
H          4.090657        0.367161        0.680558
H          2.475009        1.005625        1.215674
```

---

ethylcrotonate_2_reopt_am1_HEI_reopt

Datum	Value
AM1 Energy	-0.200668
AM1 Free Energy (Quasiharmonic)	-0.045294
Number of Imaginary Frequencies	0

Frequencies (Top 3 out of 63)

```
  1.      25.8691 cm-1
  2.      44.1632 cm-1
  3.      56.1652 cm-1
```

AM1 Molecular Geometry in Cartesian Coordinates

```
C          0.860710       -0.864154        0.673508
C         -0.498114       -1.100350        0.456826
C         -1.265114       -0.560284       -0.623200
C         -2.314708       -1.485379       -1.181098
O          1.625663       -1.271139        1.583481
O          1.499314       -0.055026       -0.284434
C          2.886608        0.173870       -0.079292
C          3.356832        1.069920       -1.204300
H         -0.628241       -0.129973       -1.434847
H         -2.997309       -0.941417       -1.879598
H         -1.821415       -2.316292       -1.743802
H         -2.926674       -1.931678       -0.360904
H          3.044934        0.659948        0.919681
H          3.434397       -0.805507       -0.084102
H          2.789636        2.030813       -1.198649
H          4.444323        1.285861       -1.080161
H          3.192878        0.574982       -2.190744
C         -1.516214        1.680375        1.196082
H         -1.108053        2.669582        0.893974
H         -0.676421        1.021914        1.532899
H         -2.229888        1.825656        2.036141
S         -2.348755        0.932324       -0.151733
H         -0.997907       -1.735749        1.194304
```

---

ethylcrotonate_3_am1_HEI

Datum	Value
AM1 Energy	-0.200903
AM1 Free Energy (Quasiharmonic)	-0.04484
Number of Imaginary Frequencies	0

Frequencies (Top 3 out of 63)

```
  1.      30.8552 cm-1
  2.      44.0455 cm-1
  3.      54.8769 cm-1
```

AM1 Molecular Geometry in Cartesian Coordinates

```
C         -0.950317        0.125730        0.759958
C          0.346307        0.634679        0.852813
C          1.231593        0.838454       -0.252652
C          2.104024        2.061981       -0.149509
```

```
O          -1.800171       -0.105629        1.656164
O          -1.396660       -0.165109       -0.543163
C          -2.709195       -0.679802       -0.680846
C          -3.741087        0.427187       -0.676910
H           0.709669        0.792509       -1.240139
H           2.896177        2.056390       -0.938304
H           1.479469        2.980050       -0.281612
H           2.597740        2.114126        0.850456
H          -2.692366       -1.204752       -1.674948
H          -2.932666       -1.418170        0.134079
H          -3.515411        1.176494       -1.471966
H          -4.758469        0.005527       -0.854065
H          -3.722023        0.943036        0.313994
C           1.845226       -1.950112        0.234540
H           2.549801       -2.396420        0.969743
H           1.637143       -2.695144       -0.564232
H           0.888388       -1.686137        0.751274
S           2.561261       -0.507620       -0.455271
H           0.697716        0.871615        1.861682
```

---

ethylcrotonate_3_am1

Datum	Value
AM1 Energy	-0.137102
AM1 Free Energy (Quasiharmonic)	-0.01605
Number of Imaginary Frequencies	0

Frequencies (Top 3 out of 48)

```
1.      44.6530 cm-1
2.      59.7331 cm-1
3.      93.7881 cm-1
```

AM1 Molecular Geometry in Cartesian Coordinates

```
C           0.012474        0.900161        0.000002
C          -1.428876        0.639219        0.000002
C          -1.964377       -0.587356       -0.000002
C          -3.417133       -0.841587       -0.000002
O           0.574113        2.000307        0.000003
O           0.800007       -0.221179       -0.000001
C           2.222072       -0.002293       -0.000001
C           2.860268       -1.369925       -0.000000
H          -2.039379        1.557852        0.000005
```

```
H          -1.319381      -1.485134      -0.000005
H          -3.698174      -1.436004       0.906298
H          -4.010843       0.105392       0.000001
H          -3.698175      -1.435999      -0.906305
H           2.494721       0.588719      -0.913099
H           2.494721       0.588721       0.913096
H           3.970239      -1.252510      -0.000001
H           2.553370      -1.946690       0.904956
H           2.553370      -1.946691      -0.904955
```

---

ethylcrotonate_4_am1_HEI

Datum	Value
AM1 Energy	-0.196351
AM1 Free Energy (Quasiharmonic)	-0.040645
Number of Imaginary Frequencies	0

Frequencies (Top 3 out of 63)

```
1.      35.5541 cm-1
2.      44.0472 cm-1
3.      54.5815 cm-1
```

AM1 Molecular Geometry in Cartesian Coordinates

```
C           1.177909       0.587743      -0.539085
C          -0.114718       1.083338      -0.356908
C          -1.118678       0.470260       0.463021
C          -1.972093       1.442472       1.236571
O           2.122712       1.017511      -1.246716
O           1.504609      -0.551052       0.222339
C           2.793124      -1.108051       0.035479
C           3.829135      -0.401212       0.882998
H          -0.713725      -0.322535       1.139871
H          -1.347280       1.964615       2.002590
H          -2.409935       2.212671       0.557384
H          -2.805847       0.914137       1.760922
H           2.678039      -2.178477       0.359780
H           3.088925      -1.069591      -1.046268
H           3.533213      -0.416487       1.958487
H           4.821337      -0.898960       0.773053
H           3.910149       0.662576       0.551188
C          -3.404983      -1.293057       0.214594
H          -4.189802      -0.615445       0.620440
```

```
H          -2.927089      -1.819765       1.070648
H          -3.890983      -2.044153      -0.447100
S          -2.220740      -0.424588      -0.737785
H          -0.371904       1.984189      -0.921426
```

---

ethylcrotonate_4_am1

Datum	Value
AM1 Energy	-0.137224
AM1 Free Energy (Quasiharmonic)	-0.015608
Number of Imaginary Frequencies	0

Frequencies (Top 3 out of 48)

```
1.      43.0055 cm-1
2.      69.3824 cm-1
3.      90.5986 cm-1
```

AM1 Molecular Geometry in Cartesian Coordinates

```
C           0.122711       0.688254      -0.099292
C          -1.331197       0.647946       0.078617
C          -2.063130      -0.468485      -0.016178
C          -3.526349      -0.500682       0.165255
O           0.856804       1.677650      -0.016863
O           0.695130      -0.521638      -0.396690
C           2.116806      -0.557998      -0.582772
C           2.835033      -0.613867       0.743060
H          -1.771933       1.634133       0.301381
H          -1.585290      -1.439605      -0.240973
H          -4.015332      -0.888926      -0.764146
H          -3.788942      -1.187969       1.009230
H          -3.945735       0.510831       0.389358
H           2.265039      -1.502040      -1.173633
H           2.445226       0.331088      -1.182156
H           2.467248      -1.471977       1.354607
H           3.930593      -0.735594       0.569332
H           2.660406       0.330958       1.313287
```

---

ethylcrotonate_5_am1

Datum	Value
AM1 Energy	-0.137799
AM1 Free Energy (Quasiharmonic)	-0.016604
Number of Imaginary Frequencies	0

Frequencies (Top 3 out of 48)

```
1.      41.5942 cm-1
2.      62.6763 cm-1
3.      93.7373 cm-1
```

AM1 Molecular Geometry in Cartesian Coordinates

```
C          0.018633       0.365278       -0.000029
C          1.254538      -0.422034        0.000013
C          2.455459       0.170958        0.000003
C          3.730878      -0.568337        0.000043
O         -0.122838       1.592891       -0.000072
O         -1.108267      -0.413251       -0.000016
C         -2.365953       0.285342       -0.000061
C         -3.438473      -0.776475        0.000098
H          1.122016      -1.515250        0.000051
H          2.535576       1.274044       -0.000036
H          4.328300      -0.293745       -0.906300
H          4.328282      -0.293679        0.906377
H          3.576513      -1.675298        0.000081
H         -2.416855       0.934343        0.912942
H         -2.416903       0.934117       -0.913223
H         -4.439044      -0.282047        0.000058
H         -3.349787      -1.424112       -0.904603
H         -3.349747      -1.423881        0.904960
```

---

ethylcrotonate_5_reopt_am1_HEI

Datum	Value
AM1 Energy	-0.201313
AM1 Free Energy (Quasiharmonic)	-0.045343
Number of Imaginary Frequencies	0

Frequencies (Top 3 out of 63)

```
1.      32.2044 cm-1
2.      42.0531 cm-1
3.      59.4919 cm-1
```

AM1 Molecular Geometry in Cartesian Coordinates

```
C        -0.885440      -0.559895       0.159643
C         0.345614      -0.787008      -0.458500
C         1.577798      -0.756438       0.271665
C         2.596386      -1.778382      -0.159534
O        -1.212528      -0.343717       1.352209
O        -1.973738      -0.604865      -0.737464
C        -3.269678      -0.438819      -0.191708
C        -3.613214       1.024712      -0.017139
H         1.417288      -0.777918       1.378393
H         3.590131      -1.575854       0.310897
H         2.258942      -2.798128       0.151120
H         2.719679      -1.772136      -1.269119
H        -3.948828      -0.918755      -0.948538
H        -3.358384      -0.975014       0.790145
H        -4.661054       1.133146       0.349567
H        -2.912790       1.479618       0.725158
H        -3.509115       1.570342      -0.984574
C         1.383104       2.083786      -0.230876
H         1.328235       2.767815       0.644034
H         0.381615       1.612023      -0.392518
H         1.664096       2.671141      -1.132081
S         2.584079       0.838615       0.047058
H         0.367628      -0.973195      -1.534679
```

---

ethylcrotonate_6_am1_HEI

Datum	Value
AM1 Energy	-0.201058
AM1 Free Energy (Quasiharmonic)	-0.04578
Number of Imaginary Frequencies	0

Frequencies (Top 3 out of 63)

```
1.      28.7430 cm-1
2.      41.0021 cm-1
3.      58.4175 cm-1
```

AM1 Molecular Geometry in Cartesian Coordinates

```
C         -0.829318      0.310491      0.361087
C          0.297026      0.667820     -0.382552
C          1.589700      0.806397      0.218625
C          2.428210      1.930105     -0.330859
O         -1.010851      0.086588      1.582856
O         -1.999346      0.197665     -0.418564
C         -3.186195     -0.128097      0.290201
C         -4.306291     -0.187127     -0.725653
H          1.538738      0.839523      1.335443
H          2.001137      2.910632     -0.004610
H          3.480844      1.861786      0.039475
H          2.441069      1.906598     -1.446969
H         -3.056865     -1.115110      0.808445
H         -3.387002      0.653700      1.069865
H         -4.088985     -0.960471     -1.500032
H         -5.263228     -0.443009     -0.212355
H         -4.418837      0.797272     -1.238694
C          1.695814     -2.050789     -0.210329
H          1.813849     -2.709228      0.677867
H          0.632934     -1.707738     -0.275299
H          1.954032     -2.627630     -1.125034
S          2.756963     -0.662426     -0.081721
H          0.188811      0.825676     -1.458020
```

---

ethylcrotonate_6_am1

Datum	Value
AM1 Energy	-0.129237
AM1 Free Energy (Quasiharmonic)	-0.007566
Number of Imaginary Frequencies	0

Frequencies (Top 3 out of 48)

```
1.      23.9699 cm-1
2.      72.6289 cm-1
3.      98.5637 cm-1
```

AM1 Molecular Geometry in Cartesian Coordinates

```
C         -0.161903       0.928094        0.033567
C          0.790411      -0.184876       -0.026162
C          2.112043       0.039176       -0.005127
C          3.125904      -1.029561       -0.052014
O          0.100058       2.102795        0.313587
O         -1.498928       0.765723       -0.237314
C         -1.996475      -0.514106       -0.623225
C         -2.293945      -1.377385        0.579648
H          0.377885      -1.203872       -0.069625
H          2.501448       1.073412        0.050985
H          3.783822      -0.889703       -0.947264
H          3.769862      -0.980145        0.862885
H          2.666146      -2.046749       -0.106226
H         -2.947566      -0.256012       -1.168656
H         -1.294332      -1.018971       -1.335623
H         -2.994576      -0.848278        1.270114
H         -2.766316      -2.332241        0.246857
H         -1.361624      -1.613640        1.146245
```

---

ethylcrotonate_7_am1_HEI

Datum	Value
AM1 Energy	-0.201318
AM1 Free Energy (Quasiharmonic)	-0.04533
Number of Imaginary Frequencies	0

Frequencies (Top 3 out of 63)

```
  1.      33.7312 cm-1
  2.      44.1270 cm-1
  3.      55.9031 cm-1
```

AM1 Molecular Geometry in Cartesian Coordinates

```
C         -0.909618       0.075677        0.086898
C          0.252825       0.570626       -0.507164
C          1.434733       0.847185        0.253734
C          2.199725       2.068353       -0.183716
O         -1.212292      -0.187793        1.276403
O         -1.949570      -0.157427       -0.837989
C         -3.177981      -0.638037       -0.323408
C         -4.033118       0.489760        0.212546
H          1.239264       0.857285        1.354987
```

```
H          3.197892       2.115915       0.317534
H          2.356113       2.063809      -1.289135
H          1.623881       2.988016       0.086240
H         -3.672304      -1.125874      -1.207694
H         -2.999927      -1.401579       0.479587
H         -5.021058       0.097041       0.550127
H         -3.510216       0.967264       1.076821
H         -4.199632       1.263659      -0.573475
C          1.923174      -1.971937      -0.158046
H          2.004145      -2.625066       0.738192
H          0.844370      -1.752410      -0.356986
H          2.359306      -2.502150      -1.032560
S          2.791921      -0.472874       0.103218
H          0.263885       0.732066      -1.587505
```

---

ethylcrotonate_7_am1_reopt

Datum	Value
AM1 Energy	-0.129262
AM1 Free Energy (Quasiharmonic)	-0.008283
Number of Imaginary Frequencies	0

Frequencies (Top 3 out of 48)

```
1.      17.9374 cm-1
2.      90.7399 cm-1
3.      91.6844 cm-1
```

AM1 Molecular Geometry in Cartesian Coordinates

```
C          0.015470       0.830349      -0.048342
C         -0.979582      -0.243884      -0.117883
C         -2.279735       0.007784       0.091248
C         -3.334580      -1.019614       0.028394
O         -0.211748       2.043330       0.007508
O          1.363911       0.568746      -0.044963
C          1.791909      -0.796869      -0.041046
C          3.296760      -0.765831       0.100088
H         -0.619557      -1.254151      -0.362641
H         -2.617793       1.034789       0.326787
H         -4.085234      -0.743197      -0.755381
H         -2.926033      -2.031102      -0.214445
H         -3.866068      -1.078696       1.012236
H          1.317405      -1.338662       0.818850
```

```
H          1.488717       -1.280675       -1.007029
H          3.686707       -1.811146        0.093797
H          3.754923       -0.196245       -0.743546
H          3.588174       -0.269144        1.056255
```

---

ethylcrotonate_8_am1_HEI

Datum	Value
AM1 Energy	-0.196858
AM1 Free Energy (Quasiharmonic)	-0.041232
Number of Imaginary Frequencies	0

Frequencies (Top 3 out of 63)

```
1.      37.0873 cm-1
2.      44.9695 cm-1
3.      50.7675 cm-1
```

AM1 Molecular Geometry in Cartesian Coordinates

```
C          1.108514        0.409942        0.202439
C         -0.083828        0.788423       -0.419062
C         -1.351696        0.649357        0.239492
C         -2.302995        1.795779        0.012949
O          1.372254        0.001451        1.359460
O          2.240271        0.569629       -0.624942
C          3.499963        0.240984       -0.068546
C          3.763491       -1.247666       -0.136426
H         -1.251730        0.426199        1.331441
H         -2.427333        1.997934       -1.077854
H         -3.307137        1.574969        0.451372
H         -1.900991        2.719574        0.497533
H          4.235628        0.804772       -0.705241
H          3.569333        0.596634        0.993630
H          3.685927       -1.612035       -1.187923
H          4.783236       -1.476891        0.253192
H          3.001420       -1.782414        0.481758
C         -3.596607       -1.175573        0.352744
H         -3.921547       -2.214062        0.120121
H         -3.452348       -1.078811        1.452041
H         -4.404133       -0.472706        0.046675
S         -2.104914       -0.873602       -0.511787
H         -0.052962        1.158368       -1.445844
```

ethylcrotonate_9_reopt_am1_HEI_reopt

Datum	Value
AM1 Energy	-0.196573
AM1 Free Energy (Quasiharmonic)	-0.04164
Number of Imaginary Frequencies	0

Frequencies (Top 3 out of 63)

```
1.      29.5496 cm-1
2.      41.8175 cm-1
3.      57.4621 cm-1
```

AM1 Molecular Geometry in Cartesian Coordinates

```
C        1.025541      0.127897      0.369639
C       -0.076021      0.633753     -0.324584
C       -1.392742      0.625082      0.246441
C       -2.200921      1.867333     -0.026027
O        1.170597     -0.314393      1.535094
O        2.221499      0.169375     -0.376956
C        3.381767     -0.310083      0.286522
C        4.526908     -0.213121     -0.698168
H       -1.390896      0.384199      1.339173
H       -2.229169      2.089810     -1.119589
H       -3.250076      1.750579      0.341283
H       -1.737919      2.739727      0.497794
H        3.578814      0.313619      1.198583
H        3.218581     -1.370595      0.615257
H        5.464360     -0.582503     -0.219153
H        4.312898     -0.827630     -1.604557
H        4.674385      0.844544     -1.021414
C       -3.820396     -0.952608      0.182998
H       -3.741666     -0.879389      1.290730
H       -4.236067     -1.949257     -0.085449
H       -4.527043     -0.166178     -0.166264
S       -2.250037     -0.803815     -0.575651
H        0.062801      1.004740     -1.342003
```

n-propylacrylate_10_am1

Datum	Value
AM1 Energy	-0.129842
AM1 Free Energy (Quasiharmonic)	-0.007164
Number of Imaginary Frequencies	0

Frequencies (Top 3 out of 48)

```
1.      26.4896 cm-1
2.      52.1392 cm-1
3.      84.3643 cm-1
```

AM1 Molecular Geometry in Cartesian Coordinates

```
C        -0.886165       0.513925      -0.232791
C        -2.263487       0.378264       0.259177
C        -2.846651      -0.786486       0.537956
O        -0.294122       1.563622      -0.498875
O        -0.231985      -0.675723      -0.409997
C         1.121033      -0.650880      -0.886000
C         2.091748      -0.779152       0.274852
C         2.498048       0.561266       0.829238
H        -2.776342       1.347959       0.378195
H        -3.881048      -0.842868       0.905175
H        -2.331452      -1.750861       0.417731
H         1.170424      -1.557244      -1.549134
H         1.315347       0.276704      -1.484239
H         1.619464      -1.399467       1.081749
H         3.000054      -1.330337      -0.085311
H         3.108378       1.127559       0.084593
H         3.103738       0.431217       1.757814
H         1.593140       1.172522       1.069803
```

---

n-propylacrylate_10_reopt_am1_HEI

Datum	Value
AM1 Energy	-0.200215
AM1 Free Energy (Quasiharmonic)	-0.044669
Number of Imaginary Frequencies	0

Frequencies (Top 3 out of 63)

```
1.      20.8342 cm-1
2.      37.3115 cm-1
3.      42.5867 cm-1
```

AM1 Molecular Geometry in Cartesian Coordinates

```
C          0.491749        1.432972        0.130566
C         -0.823585        1.370282        0.595524
C         -1.538536        0.164844        0.866456
O          1.213561        2.420684       -0.154410
O          1.148456        0.194511       -0.002913
C          2.485326        0.249229       -0.479585
C          2.965970       -1.184906       -0.642326
C          3.211353       -1.849597        0.686616
H         -2.185756        0.220886        1.775304
H         -0.895743       -0.743739        0.918589
H          2.513651        0.789738       -1.462635
H          3.123347        0.810138        0.253102
H          2.195343       -1.764342       -1.214801
H          3.910209       -1.175065       -1.246618
H          2.282467       -1.804908        1.306639
H          3.501637       -2.918641        0.546412
H          4.029281       -1.331258        1.243052
S         -2.727141       -0.102819       -0.518252
C         -3.615986       -1.537412       -0.055908
H         -4.124553       -1.403456        0.925361
H         -2.947588       -2.424240        0.020906
H         -4.384225       -1.732697       -0.836457
H         -1.337707        2.328662        0.713701
```

---

n-propylacrylate_11_am1_HEI

Datum	Value
AM1 Energy	-0.204625
AM1 Free Energy (Quasiharmonic)	-0.047329
Number of Imaginary Frequencies	0

Frequencies (Top 3 out of 63)

```
1.      25.4900 cm-1
2.      28.4538 cm-1
3.      60.3737 cm-1
```

AM1 Molecular Geometry in Cartesian Coordinates

```
C         -0.383861      -0.750677       0.242666
C          0.810016      -1.146503      -0.361568
C          1.623627      -0.322904      -1.197976
O         -1.164020      -1.386797       0.995322
O         -0.795088       0.565353      -0.042831
C         -1.998928       1.032572       0.536294
C         -3.152860       0.934830      -0.454996
C         -3.869811      -0.385534      -0.348354
H          2.084547      -0.877705      -2.051702
H          1.099464       0.589153      -1.565586
H         -2.241448       0.480484       1.481893
H         -1.792306       2.113449       0.770920
H         -3.870020       1.775153      -0.263462
H         -2.748837       1.063634      -1.493151
H         -3.120107      -1.217883      -0.316889
H         -4.554849      -0.536307      -1.216654
H         -4.473925      -0.431046       0.589841
S          3.183911       0.336059      -0.415006
C          2.808931       0.462473       1.292945
H          3.611245      -0.032081       1.882506
H          2.744403       1.530499       1.595154
H          1.831081      -0.040075       1.500566
H          1.138353      -2.168208      -0.147334
```

---

n-propylacrylate_11_am1

Datum	Value
AM1 Energy	-0.122369
AM1 Free Energy (Quasiharmonic)	0.000568
Number of Imaginary Frequencies	0

Frequencies (Top 3 out of 48)

```
1.      26.8371 cm-1
2.      48.8846 cm-1
3.      75.8234 cm-1
```

AM1 Molecular Geometry in Cartesian Coordinates

```
```

```
C          1.010863      -0.575344      -0.008242
C          1.506752       0.805254      -0.088605
C          2.810219       1.069925      -0.190810
O          1.619980      -1.593368      -0.350545
O         -0.234446      -0.875501       0.486975
C         -1.068502       0.171502       0.980735
C         -1.864280       0.829226      -0.133195
C         -2.935415      -0.084666      -0.669407
H          0.750097       1.605233      -0.084402
H          3.192699       2.097461      -0.261988
H          3.570730       0.274305      -0.210046
H         -1.754395      -0.368889       1.691375
H         -0.466367       0.924864       1.551225
H         -1.172841       1.122048      -0.966744
H         -2.328247       1.767251       0.270279
H         -3.455501       0.392553      -1.534365
H         -2.484677      -1.049369      -1.008792
H         -3.693587      -0.309885       0.119159
```

---

n-propylacrylate_12_am1_HEI

Datum	Value
AM1 Energy	-0.205481
AM1 Free Energy (Quasiharmonic)	-0.048559
Number of Imaginary Frequencies	0

Frequencies (Top 3 out of 63)

```
1.      26.9471 cm-1
2.      31.1441 cm-1
3.      55.8602 cm-1
```

AM1 Molecular Geometry in Cartesian Coordinates

```
C         -0.279415      -0.938975       0.019257
C          0.892445      -0.924017      -0.738547
C          2.182601      -1.089371      -0.145660
O         -0.501383      -1.113973       1.242463
O         -1.443578      -0.736677      -0.753705
C         -2.686516      -0.789319      -0.079286
C         -3.029426       0.536996       0.582997
C         -3.260251       1.630211      -0.425746
H          2.882741      -1.697481      -0.768920
```

```
H          2.136175       -1.488022        0.894444
H         -3.428645       -1.030491       -0.888375
H         -2.683158       -1.603415        0.693229
H         -3.944602        0.400802        1.215491
H         -2.177522        0.812716        1.260471
H         -2.374384        1.712384       -1.102488
H         -3.416848        2.611422        0.083496
H         -4.159494        1.410404       -1.050965
S          3.210297        0.459508        0.000125
C          2.042206        1.745153        0.238551
H          2.093852        2.125494        1.282019
H          2.266658        2.578616       -0.462010
H          1.012370        1.357003        0.038283
H          0.817930       -0.760420       -1.816129
```

---

n-propylacrylate_12_am1

Datum	Value
AM1 Energy	-0.122371
AM1 Free Energy (Quasiharmonic)	6.6e-05
Number of Imaginary Frequencies	0

Frequencies (Top 3 out of 48)

```
1.      27.4728 cm-1
2.      48.4342 cm-1
3.      80.6858 cm-1
```

AM1 Molecular Geometry in Cartesian Coordinates

```
C          1.097846        0.525998       -0.158793
C          1.816985       -0.753874       -0.104235
C          2.998733       -0.864350        0.504172
O          1.585516        1.652733       -0.033381
O         -0.256579        0.576532       -0.378812
C         -0.974361       -0.660744       -0.446760
C         -2.451372       -0.303776       -0.454198
C         -2.944965        0.085675        0.914246
H          1.342156       -1.607393       -0.612696
H          3.549511       -1.814388        0.540361
H          3.484870       -0.011145        1.001456
H         -0.717245       -1.300632        0.437192
H         -0.691467       -1.189133       -1.395649
H         -3.019533       -1.193890       -0.831594
```

```
H          -2.618705       0.542910      -1.171375
H          -2.840070      -0.763820       1.631680
H          -2.356532       0.952039       1.303841
H          -4.021675       0.377755       0.867745
```

---

n-propylacrylate_13_am1_HEI

Datum	Value
AM1 Energy	-0.205496
AM1 Free Energy (Quasiharmonic)	-0.04848
Number of Imaginary Frequencies	0

Frequencies (Top 3 out of 63)

```
1.      26.8593 cm-1
2.      36.8890 cm-1
3.      51.5998 cm-1
```

AM1 Molecular Geometry in Cartesian Coordinates

```
C           0.299459      -0.051795       0.186338
C          -0.721745      -0.771283      -0.435940
C          -1.875480      -1.223608       0.276593
O           0.482634       0.315484       1.372796
O           1.335823       0.319558      -0.697860
C           2.429460       1.032459      -0.151843
C           3.444761       0.104443       0.498737
C           4.116550      -0.792713      -0.506138
H          -2.225359      -2.235717      -0.042270
H          -1.747333      -1.183495       1.383336
H           2.883606       1.557873      -1.035677
H           2.074275       1.785209       0.601043
H           4.212002       0.722957       1.032505
H           2.903591      -0.513523       1.264168
H           3.343495      -1.352757      -1.087714
H           4.785638      -1.526654       0.004061
H           4.730592      -0.195903      -1.223502
S          -3.446521      -0.271021      -0.040153
C          -2.913928       1.370647      -0.349385
H          -3.189217       2.032629       0.500400
H          -1.803392       1.384838      -0.482943
H          -3.403079       1.746405      -1.274237
H          -0.642609      -0.974750      -1.506382
```

---

n-propylacrylate_13_am1_reopt

Datum	Value
AM1 Energy	-0.123305
AM1 Free Energy (Quasiharmonic)	-0.000501
Number of Imaginary Frequencies	0

Frequencies (Top 3 out of 48)

```
1.      33.3563 cm-1
2.      40.2945 cm-1
3.      64.0806 cm-1
```

AM1 Molecular Geometry in Cartesian Coordinates

```
C          1.215335       -0.517948       -0.066200
C          1.301477        0.947908       -0.020387
C          2.473772        1.571515        0.106503
O          2.126754       -1.296942       -0.360542
O          0.055724       -1.189492        0.234961
C         -1.093705       -0.459581        0.659221
C         -1.923727       -0.012866       -0.531045
C         -3.186166        0.670157       -0.076144
H          0.355729        1.502678       -0.122003
H          2.551609        2.667223        0.130381
H          3.424155        1.022972        0.193124
H         -0.808533        0.403944        1.313113
H         -1.661183       -1.213798        1.272810
H         -1.322055        0.678905       -1.176909
H         -2.173385       -0.909602       -1.157776
H         -3.791218        0.988113       -0.959349
H         -3.806789       -0.019760        0.545733
H         -2.950070        1.575679        0.533840
```

---

n-propylacrylate_14_am1_HEI

Datum	Value
AM1 Energy	-0.204959
AM1 Free Energy (Quasiharmonic)	-0.048694

Datum	Value
Number of Imaginary Frequencies	0

Frequencies (Top 3 out of 63)

```
1.      19.7896 cm-1
2.      33.9470 cm-1
3.      45.8985 cm-1
```

AM1 Molecular Geometry in Cartesian Coordinates

```
C        0.172349     -0.805023      0.057155
C       -0.869577     -0.597258     -0.848139
C       -2.214801     -0.983776     -0.559531
O        0.225931     -1.340297      1.191341
O        1.418324     -0.333648     -0.408655
C        2.523303     -0.546261      0.457405
C        3.755741      0.017126     -0.233947
C        3.751792      1.523079     -0.249226
H       -2.759821     -1.391038     -1.445793
H       -2.291491     -1.684039      0.304490
H        2.339358     -0.034929      1.438799
H        2.644961     -1.645668      0.647516
H        3.792781     -0.368273     -1.286180
H        4.665193     -0.359013      0.303014
H        4.629581      1.914890     -0.817302
H        2.813681      1.892369     -0.731780
H        3.793717      1.928722      0.790462
S       -3.381692      0.406654     -0.129308
C       -2.376701      1.591697      0.683022
H       -2.618470      1.624906      1.767723
H       -2.561579      2.594409      0.240082
H       -1.299318      1.319894      0.553718
H       -0.648216     -0.104631     -1.797742
```

---

n-propylacrylate_14_am1

Datum	Value
AM1 Energy	-0.123248
AM1 Free Energy (Quasiharmonic)	-0.001099
Number of Imaginary Frequencies	0

Frequencies (Top 3 out of 48)

```
1.      30.4558 cm-1
2.      46.2206 cm-1
3.      87.9001 cm-1
```

AM1 Molecular Geometry in Cartesian Coordinates

```
C         -1.271601       -0.522852       -0.074265
C         -1.726152        0.868347       -0.200186
C         -2.940609        1.243878        0.203683
O         -1.983032       -1.524970        0.037679
O          0.063889       -0.842949       -0.081306
C          1.012566        0.228182       -0.113151
C          2.372773       -0.408717        0.117569
C          3.457554        0.634553        0.074113
H         -1.023501        1.576311       -0.666560
H         -3.294089        2.279404        0.103765
H         -3.652611        0.537734        0.657597
H          0.776371        0.973045        0.690915
H          0.969447        0.727081       -1.117114
H          2.371672       -0.930761        1.110016
H          2.550484       -1.190671       -0.666487
H          4.452800        0.157593        0.245721
H          3.478428        1.145146       -0.919165
H          3.296952        1.408124        0.863746
```

---

n-propylacrylate_15_am1_HEI

Datum	Value
AM1 Energy	-0.205101
AM1 Free Energy (Quasiharmonic)	-0.048061
Number of Imaginary Frequencies	0

Frequencies (Top 3 out of 63)

```
1.      20.0890 cm-1
2.      34.6396 cm-1
3.      48.6326 cm-1
```

AM1 Molecular Geometry in Cartesian Coordinates

```
C          0.340254      -0.918990       0.184124
C         -0.872855      -1.183569      -0.452874
C         -2.122909      -1.116038       0.237273
O          0.628600      -0.621192       1.369435
O          1.452109      -1.030535      -0.678302
C          2.736427      -0.760513      -0.150960
C          3.188580       0.645512      -0.527692
C          2.645783       1.677600       0.425518
H         -2.858110      -1.886258      -0.100912
H         -2.010189      -1.140195       1.346167
H          2.763611      -0.898230       0.961871
H          3.408493      -1.517228      -0.642516
H          4.309251       0.677780      -0.530706
H          2.838685       0.871445      -1.568792
H          1.550764       1.500969       0.585723
H          2.799745       2.708027       0.025185
H          3.152781       1.602819       1.417660
S         -3.143786       0.409234      -0.088081
C         -1.965303       1.697647      -0.252471
H         -0.936964       1.261229      -0.312577
H         -2.176986       2.273194      -1.179696
H         -2.021358       2.384054       0.620419
H         -0.864680      -1.421415      -1.519108
```

---

n-propylacrylate_15_am1

Datum	Value
AM1 Energy	-0.121381
AM1 Free Energy (Quasiharmonic)	0.001935
Number of Imaginary Frequencies	0

Frequencies (Top 3 out of 48)

```
1.      38.3873 cm-1
2.      54.7171 cm-1
3.      80.3617 cm-1
```

AM1 Molecular Geometry in Cartesian Coordinates

```
C         -1.013821      -0.556856      -0.191527
C         -1.247569       0.868892       0.078358
C         -2.461963       1.341273       0.358754
O         -1.793461      -1.335722      -0.746960
```

```
O          0.158220       -1.179350        0.158156
C          1.133317       -0.487344        0.939397
C          2.335124       -0.128207        0.083167
C          2.133673        1.124005       -0.727267
H         -0.365941        1.525701       -0.005897
H         -2.643683        2.409565        0.540633
H         -3.347049        0.690673        0.422616
H          1.431922       -1.245937        1.716832
H          0.705178        0.409672        1.452910
H          3.217258        0.002233        0.764527
H          2.559077       -0.988482       -0.602580
H          3.052300        1.350160       -1.321206
H          1.279865        0.997812       -1.437220
H          1.920438        1.998590       -0.065471
```

---

n-propylacrylate_16_reopt_am1_HEI

Datum	Value
AM1 Energy	-0.206377
AM1 Free Energy (Quasiharmonic)	-0.049595
Number of Imaginary Frequencies	0

Frequencies (Top 3 out of 63)

```
1.      27.1800 cm-1
2.      36.2781 cm-1
3.      55.6342 cm-1
```

AM1 Molecular Geometry in Cartesian Coordinates

```
C          0.188203       -0.823799        0.092752
C         -1.056596       -1.060541       -0.492117
C         -2.266495       -1.056928        0.269179
O          0.547725       -0.613106        1.276943
O          1.251849       -0.846895       -0.835667
C          2.560925       -0.682550       -0.325582
C          2.894453        0.789033       -0.132614
C          4.330547        0.977045        0.275668
H         -2.995962       -1.839459       -0.053283
H         -2.089682       -1.114708        1.368403
H          2.685119       -1.233772        0.643569
H          3.223752       -1.137727       -1.111175
H          2.687855        1.344033       -1.083953
H          2.205431        1.198913        0.653409
```

```
H           4.538607        0.443657        1.234908
H           4.557981        2.060892        0.419857
H           5.021566        0.574335       -0.504480
S          -3.352216        0.443751        0.055552
C          -2.235170        1.758126       -0.258675
H          -2.580481        2.327322       -1.148955
H          -1.214821        1.343356       -0.454164
H          -2.190518        2.444492        0.614853
H          -1.105183       -1.233665       -1.569694
```

---

n-propylacrylate_17_reopt_am1_HEI

Datum	Value
AM1 Energy	-0.204961
AM1 Free Energy (Quasiharmonic)	-0.048655
Number of Imaginary Frequencies	0

Frequencies (Top 3 out of 63)

```
1.      19.7366 cm-1
2.      36.4652 cm-1
3.      43.1163 cm-1
```

AM1 Molecular Geometry in Cartesian Coordinates

```
C          -0.192358       -0.370834        0.432619
C           0.795820       -0.809810       -0.450082
C           2.089665       -1.216966        0.000044
O          -0.235524       -0.261445        1.682389
O          -1.388441        0.008838       -0.213053
C          -2.446305        0.423806        0.638413
C          -3.633580        0.767073       -0.248613
C          -4.272278       -0.465122       -0.833358
H           2.500393       -2.091295       -0.561397
H           2.131036       -1.401298        1.098726
H          -2.121582        1.321674        1.228417
H          -2.700890       -0.399339        1.356707
H          -3.288592        1.439408       -1.076908
H          -4.381759        1.332815        0.365422
H          -3.506517       -1.056772       -1.392668
H          -5.097785       -0.188581       -1.532499
H          -4.693949       -1.112152       -0.026634
S           3.474184       -0.007218       -0.315451
C           2.715100        1.570123       -0.215062
```

```
H          3.009582       2.175518       -1.099698
H          1.603068       1.449709       -0.199190
H          3.039734       2.097068        0.708560
H          0.575667      -0.820033       -1.520060
```

---

n-propylacrylate_18_am1_HEI

Datum	Value
AM1 Energy	-0.205809
AM1 Free Energy (Quasiharmonic)	-0.049817
Number of Imaginary Frequencies	0

Frequencies (Top 3 out of 63)

```
  1.      23.0521 cm-1
  2.      32.9976 cm-1
  3.      45.7298 cm-1
```

AM1 Molecular Geometry in Cartesian Coordinates

```
C         -0.100384      -0.610575        0.139020
C          1.001071      -0.732510       -0.709595
C          2.289894      -1.121189       -0.229773
O         -0.260272      -0.812228        1.367580
O         -1.273365      -0.193776       -0.526116
C         -2.437102      -0.088247        0.279727
C         -3.566073       0.355068       -0.638231
C         -4.854523       0.499632        0.127365
H          2.841214      -1.792899       -0.932060
H          2.259325      -1.562901        0.793334
H         -2.665785      -1.080618        0.750308
H         -2.265806       0.659412        1.098449
H         -3.289289       1.329379       -1.118236
H         -3.687045      -0.395256       -1.462031
H         -4.746800       1.258350        0.940097
H         -5.678743       0.825202       -0.552199
H         -5.145934      -0.471951        0.595224
S          3.552337       0.246253       -0.102417
C          2.616134       1.678625        0.279732
H          1.527127       1.460222        0.146562
H          2.914490       2.504191       -0.402348
H          2.801121       1.992182        1.330249
H          0.873726      -0.502146       -1.769852
```

n-propylacrylate_19_reopt_am1_HEI

Datum	Value
AM1 Energy	-0.206383
AM1 Free Energy (Quasiharmonic)	-0.049541
Number of Imaginary Frequencies	0

Frequencies (Top 3 out of 63)

```
1.      27.7128 cm-1
2.      39.1012 cm-1
3.      48.9275 cm-1
```

AM1 Molecular Geometry in Cartesian Coordinates

```
C         -0.222492      -0.242093       0.044719
C          0.921582      -0.708033       0.694020
C          2.045743      -1.222815      -0.023410
O         -0.551884      -0.183343      -1.165260
O         -1.206963       0.242897       0.933261
C         -2.411457       0.722131       0.367304
C         -3.368939      -0.420421       0.064235
C         -4.702266       0.092016      -0.409107
H          2.527914      -2.098243       0.476176
H          1.812578      -1.458004      -1.087937
H         -2.839510       1.396865       1.157935
H         -2.208774       1.310180      -0.566362
H         -2.895508      -1.068234      -0.721041
H         -3.500618      -1.047738       0.983402
H         -5.180326       0.734640       0.369862
H         -5.391213      -0.757762      -0.633799
H         -4.581081       0.701189      -1.337633
S          3.530619      -0.098996      -0.114472
C          2.859483       1.520780      -0.129472
H          3.389931       2.144056       0.622832
H          2.983892       1.981294      -1.133754
H          1.770736       1.476998       0.123599
H          0.962925      -0.657139       1.784531
```

n-propylacrylate_1_am1

Datum	Value
AM1 Energy	-0.131141
AM1 Free Energy (Quasiharmonic)	-0.008325
Number of Imaginary Frequencies	0

Frequencies (Top 3 out of 48)

```
1.      44.6269 cm-1
2.      59.5380 cm-1
3.      72.8066 cm-1
```

AM1 Molecular Geometry in Cartesian Coordinates

```
C        -0.950036      -0.224406       0.118118
C        -2.096068       0.688721       0.220301
C        -3.251770       0.429768      -0.391523
O        -0.865518      -1.293726      -0.493621
O         0.138722       0.216178       0.822495
C         1.321670      -0.594271       0.797718
C         2.131943      -0.357016      -0.462317
C         2.725771       1.026099      -0.505719
H        -1.926096       1.588792       0.832614
H        -4.112024       1.109290      -0.318478
H        -3.406177      -0.474204      -0.999814
H         1.875218      -0.252692       1.713572
H         1.041399      -1.676369       0.888191
H         2.948508      -1.124953      -0.503439
H         1.468722      -0.518945      -1.353412
H         1.922449       1.796107      -0.405631
H         3.259204       1.189659      -1.472789
H         3.454101       1.170332       0.328729
```

---

n-propylacrylate_1_reopt_am1_HEI

Datum	Value
AM1 Energy	-0.205351
AM1 Free Energy (Quasiharmonic)	-0.047634
Number of Imaginary Frequencies	0

Frequencies (Top 3 out of 63)

```
1.      36.6906 cm-1
2.      47.2792 cm-1
3.      65.2763 cm-1
```

AM1 Molecular Geometry in Cartesian Coordinates

```
C          0.374854      -1.346332      -0.275244
C         -1.012997      -1.488235      -0.243078
C         -1.878357      -0.894255       0.724459
O          1.229169      -1.829185      -1.060089
O          0.922306      -0.543225       0.744859
C          2.326923      -0.368176       0.753061
C          2.774905       0.693148      -0.240822
C          2.201117       2.047445       0.079447
H         -2.766637      -1.526108       0.970052
H         -1.351952      -0.581718       1.655621
H          2.846554      -1.336661       0.526665
H          2.554891      -0.045617       1.805425
H          2.441499       0.369334      -1.263045
H          3.894714       0.739920      -0.239305
H          1.083707       1.985676       0.109752
H          2.499796       2.795625      -0.693606
H          2.559720       2.405738       1.074607
S         -2.778741       0.648327       0.179093
C         -1.614557       1.497974      -0.819670
H         -1.245358       2.406719      -0.295268
H         -0.748994       0.824081      -1.040397
H         -2.099315       1.801949      -1.772652
H         -1.451902      -2.082298      -1.050427
```

---

n-propylacrylate_20_reopt_am1_HEI

Datum	Value
AM1 Energy	-0.19481
AM1 Free Energy (Quasiharmonic)	-0.037956
Number of Imaginary Frequencies	0

Frequencies (Top 3 out of 63)

```
1.      38.9388 cm-1
2.      43.6855 cm-1
3.      56.6971 cm-1
```

AM1 Molecular Geometry in Cartesian Coordinates

```
C          0.960653        1.387609       -0.051589
C         -0.394354        1.622412        0.196781
C         -1.482627        0.992651       -0.473356
O          1.972410        1.945555        0.431435
O          1.356202        0.468391       -1.062089
C          1.027047       -0.886144       -0.814156
C          2.258146       -1.660441       -0.359091
C          2.652105       -1.304817        1.049870
H         -2.356234        1.664660       -0.646243
H         -1.201648        0.482364       -1.424261
H          0.677925       -1.292879       -1.803777
H          0.194421       -0.979616       -0.062122
H          3.108084       -1.431463       -1.053581
H          2.039211       -2.757425       -0.434715
H          2.759951       -0.191892        1.136858
H          3.619843       -1.789279        1.323661
H          1.870857       -1.639152        1.774246
S         -2.138379       -0.331147        0.644366
C         -3.523280       -0.987777       -0.201011
H         -4.276075       -0.197739       -0.420431
H         -3.226762       -1.459894       -1.164364
H         -3.989524       -1.760074        0.449900
H         -0.621028        2.358211        0.975522
```

---

n-propylacrylate_22_am1_HEI

Datum	Value
AM1 Energy	-0.194728
AM1 Free Energy (Quasiharmonic)	-0.038536
Number of Imaginary Frequencies	0

Frequencies (Top 3 out of 63)

```
1.      36.9086 cm-1
2.      46.4624 cm-1
3.      49.4458 cm-1
```

AM1 Molecular Geometry in Cartesian Coordinates

```
C        -0.725133      1.455893      0.035134
C         0.656464      1.638719     -0.057051
C         1.631030      0.841026      0.610925
O        -1.664614      2.108355     -0.472660
O        -1.231944      0.437401      0.895111
C        -1.148233     -0.866996      0.348925
C        -2.288985     -1.138503     -0.622353
C        -3.589176     -1.395506      0.092041
H         2.536745      1.407352      0.931781
H         1.227860      0.249091      1.465420
H        -1.215149     -1.552266      1.237048
H        -0.155506     -1.012213     -0.165669
H        -2.023580     -2.019378     -1.261811
H        -2.398513     -0.239678     -1.287943
H        -3.526517     -2.321672      0.713574
H        -4.424108     -1.518905     -0.639088
H        -3.824644     -0.534282      0.764231
S         2.269550     -0.399403     -0.607982
C         3.513122     -1.277270      0.256161
H         3.959368     -2.019307     -0.442217
H         4.314375     -0.594616      0.618086
H         3.094878     -1.816205      1.135654
H         0.999928      2.452293     -0.703650
```

---

n-propylacrylate_23_am1_HEI

Datum	Value
AM1 Energy	-0.195563
AM1 Free Energy (Quasiharmonic)	-0.03955
Number of Imaginary Frequencies	0

Frequencies (Top 3 out of 63)

```
1.      36.4425 cm-1
2.      46.2210 cm-1
3.      46.7766 cm-1
```

AM1 Molecular Geometry in Cartesian Coordinates

```
C         0.798367     -1.607760     -0.066741
C        -0.572812     -1.698292     -0.315802
C        -1.581187     -1.001056      0.411963
O         1.762123     -2.189983     -0.612739
```

```
O           1.250339       -0.810438        1.026364
C           1.269872        0.578209        0.750756
C           2.518479        0.957090       -0.032563
C           2.484005        2.400293       -0.456361
H          -2.531151       -1.573350        0.530698
H          -1.243193       -0.612287        1.400749
H           1.277264        1.071429        1.760334
H           0.340310        0.882999        0.190670
H           2.589094        0.282042       -0.927462
H           3.424864        0.760455        0.596474
H           1.598817        2.596101       -1.109291
H           3.407883        2.663967       -1.025837
H           2.416250        3.072770        0.433253
S          -2.059591        0.487507       -0.581272
C          -3.354318        1.235294        0.328844
H          -3.708730        2.122129       -0.241602
H          -4.206713        0.533992        0.472475
H          -3.007327        1.567697        1.332838
H          -0.878032       -2.347352       -1.142528
```

---

n-propylacrylate_24_am1_HEI

Datum	Value
AM1 Energy	-0.205054
AM1 Free Energy (Quasiharmonic)	-0.048004
Number of Imaginary Frequencies	0

Frequencies (Top 3 out of 63)

```
1.      25.7886 cm-1
2.      34.5827 cm-1
3.      53.2019 cm-1
```

AM1 Molecular Geometry in Cartesian Coordinates

```
C          -0.324806        0.829712        0.537339
C           0.852328        0.418688        1.164016
C           1.520030       -0.821685        0.930539
O          -0.986379        1.891276        0.658578
O          -0.881874       -0.092579       -0.370129
C          -2.080219        0.281794       -1.024463
C          -3.304487        0.020815       -0.159464
C          -3.521849       -1.448715        0.084009
H           1.967540       -1.264649        1.853847
```

```
H          0.886559      -1.573943       0.406568
H         -2.101479      -0.362188      -1.945399
H         -2.050227       1.365561      -1.314093
H         -4.204047       0.462576      -0.661266
H         -3.155071       0.554255       0.817092
H         -4.365330      -1.610847       0.797377
H         -2.594120      -1.901186       0.513129
H         -3.760676      -1.978277      -0.870095
S          3.065155      -0.727911      -0.113155
C          2.811735       0.651132      -1.165567
H          3.700315       1.317495      -1.118111
H          2.668826       0.313546      -2.215219
H          1.905097       1.215757      -0.832196
H          1.289767       1.128462       1.872796
```

---

n-propylacrylate_25_am1_HEI

Datum	Value
AM1 Energy	-0.205083
AM1 Free Energy (Quasiharmonic)	-0.047855
Number of Imaginary Frequencies	0

Frequencies (Top 3 out of 63)

```
1.      24.0656 cm-1
2.      38.4797 cm-1
3.      48.5997 cm-1
```

AM1 Molecular Geometry in Cartesian Coordinates

```
C         -0.337617      -0.074825      -0.061511
C          0.759496      -0.395449      -0.862263
C          1.847519      -1.186951      -0.378377
O         -0.645292      -0.376765       1.117787
O         -1.284901       0.735610      -0.723205
C         -2.454906       1.106591      -0.020166
C         -3.624460       0.203315      -0.394812
C         -3.716430      -0.994615       0.513665
H          2.253496      -1.895609      -1.140955
H          1.607137      -1.725017       0.568042
H         -2.664359       2.157671      -0.363056
H         -2.288096       1.098040       1.088984
H         -3.493872      -0.135323      -1.455848
H         -4.574685       0.796145      -0.342331
```

```
H          -2.701180       -1.453940        0.632618
H          -4.420615       -1.754531        0.098247
H          -4.078616       -0.694006        1.526418
S           3.415875       -0.256516        0.004959
C           2.873754        1.320602        0.546544
H           3.443586        2.108552        0.007806
H           1.782353        1.435976        0.330125
H           3.044386        1.433486        1.639377
H           0.793879       -0.009968       -1.883903
```

---

n-propylacrylate_26_am1_HEI

Datum	Value
AM1 Energy	-0.194728
AM1 Free Energy (Quasiharmonic)	-0.038542
Number of Imaginary Frequencies	0

Frequencies (Top 3 out of 63)

```
1.      36.8434 cm-1
2.      46.3863 cm-1
3.      49.3490 cm-1
```

AM1 Molecular Geometry in Cartesian Coordinates

```
C           0.724920        1.455874        0.035129
C          -0.656657        1.638752       -0.057199
C          -1.631337        0.841228        0.610862
O           1.664442        2.108348       -0.472567
O           1.231635        0.437281        0.895042
C           1.148339       -0.866974        0.348439
C           2.289547       -1.138096       -0.622403
C           3.589402       -1.395404        0.092498
H          -2.537201        1.407606        0.931184
H          -1.228399        0.249707        1.465735
H           0.155849       -1.012169       -0.166594
H           1.214963       -1.552491        1.236391
H           2.399401       -0.239023       -1.287593
H           2.024441       -2.018732       -1.262308
H           4.424683       -1.518460       -0.638283
H           3.526460       -2.321855        0.713573
H           3.824559       -0.534506        0.765196
S          -2.269259       -0.399814       -0.607745
C          -3.513428       -1.277169        0.256007
```

```
H          -4.315488       -0.594475        0.616058
H          -3.095986       -1.814736        1.136713
H          -3.958440       -2.020345       -0.441945
H          -1.000035        2.452203       -0.703995
```

---

n-propylacrylate_27_reopt_am1_HEI

Datum	Value
AM1 Energy	-0.205054
AM1 Free Energy (Quasiharmonic)	-0.048004
Number of Imaginary Frequencies	0

Frequencies (Top 3 out of 63)

```
1.      25.7450 cm-1
2.      34.5727 cm-1
3.      53.2109 cm-1
```

AM1 Molecular Geometry in Cartesian Coordinates

```
C          -0.324827        0.829628       -0.537327
C           0.852269        0.418564       -1.164016
C           1.520007       -0.821776       -0.930477
O          -0.986505        1.891111       -0.658770
O          -0.881758       -0.092470        0.370423
C          -2.080235        0.281910        1.024529
C          -3.304355        0.020828        0.159355
C          -3.521710       -1.448734       -0.083948
H           1.967584       -1.264703       -1.853780
H           0.886491       -1.574085       -0.406620
H          -2.101605       -0.361999        1.945513
H          -2.050331        1.365701        1.314056
H          -4.203994        0.462694        0.660922
H          -3.154739        0.554124       -0.817251
H          -2.593909       -1.901289       -0.512824
H          -4.365057       -1.610951       -0.797456
H          -3.760736       -1.978154        0.870187
S           3.065006       -0.727964        0.113261
C           2.811846        0.651405        1.165281
H           2.669220        0.314136        2.215078
H           3.700413        1.317751        1.117350
H           1.905091        1.215864        0.832000
H           1.289611        1.128250       -1.872947
```

n-propylacrylate_2_am1_HEI

Datum	Value
AM1 Energy	-0.200821
AM1 Free Energy (Quasiharmonic)	-0.044523
Number of Imaginary Frequencies	0

Frequencies (Top 3 out of 63)

```
1.      26.2695 cm-1
2.      42.2035 cm-1
3.      44.6195 cm-1
```

AM1 Molecular Geometry in Cartesian Coordinates

```
C         -0.703078       -1.316367       -0.091847
C          0.674199       -1.546585       -0.105909
C          1.639062       -0.779698        0.614638
O         -1.635657       -1.910435       -0.687997
O         -1.135003       -0.283765        0.763535
C         -2.523669       -0.011569        0.804652
C         -2.966309        0.861174       -0.360119
C         -2.347153        2.232157       -0.305953
H          2.465080       -1.392495        1.050623
H          1.202898       -0.122828        1.401804
H         -2.667064        0.527806        1.780399
H         -3.113500       -0.966295        0.804200
H         -2.666183        0.343205       -1.310152
H         -4.084034        0.943435       -0.347463
H         -2.616818        2.823214       -1.213960
H         -1.233930        2.140033       -0.252316
H         -2.697978        2.791328        0.595194
S          2.485948        0.327622       -0.592913
C          3.702882        1.153375        0.355403
H          3.243303        1.774838        1.156446
H          4.402608        0.433510        0.836669
H          4.280009        1.815666       -0.327146
H          1.020112       -2.354694       -0.757179
```

n-propylacrylate_2_am1

Datum	Value
AM1 Energy	-0.130945
AM1 Free Energy (Quasiharmonic)	-0.008672
Number of Imaginary Frequencies	0

Frequencies (Top 3 out of 48)

```
1.      33.5849 cm-1
2.      46.0600 cm-1
3.      70.4542 cm-1
```

AM1 Molecular Geometry in Cartesian Coordinates

```
C        1.090837      0.238738      0.003208
C        2.104540     -0.810011     -0.165290
C        3.394761     -0.579485      0.078230
O        1.237878      1.410517      0.364937
O       -0.173783     -0.192297     -0.295171
C       -1.227805      0.777459     -0.154913
C       -2.519743      0.066844     -0.511504
C       -2.992266     -0.840262      0.594123
H        1.719321     -1.785369     -0.503280
H        4.157316     -1.360596     -0.046231
H        3.764192      0.400747      0.415433
H       -1.232983      1.156653      0.899943
H       -1.020154      1.629950     -0.853242
H       -2.367367     -0.528254     -1.450226
H       -3.294112      0.850328     -0.723377
H       -3.926513     -1.369738      0.288289
H       -2.210131     -1.602569      0.828926
H       -3.204278     -0.256620      1.522508
```

---

n-propylacrylate_3_am1_HEI_reopt

Datum	Value
AM1 Energy	-0.204623
AM1 Free Energy (Quasiharmonic)	-0.047672
Number of Imaginary Frequencies	0

Frequencies (Top 3 out of 63)

```
1.      23.4579 cm-1
2.      40.3786 cm-1
3.      51.4430 cm-1
```

AM1 Molecular Geometry in Cartesian Coordinates

```
C          0.223539        1.500911        0.230199
C         -1.146149        1.511133       -0.038062
C         -1.810217        0.645143       -0.957579
O          0.914487        2.215003        0.998716
O          0.968739        0.543877       -0.485809
C          2.355041        0.465301       -0.193794
C          2.832623       -0.912667       -0.629827
C          2.326234       -1.993193        0.289270
H         -2.690046        1.120530       -1.455849
H         -1.126866        0.196111       -1.714678
H          2.541717        0.628521        0.900412
H          2.888234        1.266178       -0.772231
H          3.953472       -0.911590       -0.644527
H          2.474328       -1.110961       -1.673547
H          2.605236       -3.002898       -0.096682
H          1.211188       -1.929667        0.367402
H          2.756121       -1.874316        1.313101
S         -2.681484       -0.831413       -0.213487
C         -1.601798       -1.371014        1.058576
H         -0.814673       -0.595828        1.234318
H         -2.182548       -1.527339        1.993371
H         -1.113577       -2.328313        0.771350
H         -1.740298        2.227453        0.537404
```

---

n-propylacrylate_3_am1

Datum	Value
AM1 Energy	-0.132055
AM1 Free Energy (Quasiharmonic)	-0.00936
Number of Imaginary Frequencies	0

Frequencies (Top 3 out of 48)

```
1.      49.9859 cm-1
2.      54.7140 cm-1
3.      67.3563 cm-1
```

AM1 Molecular Geometry in Cartesian Coordinates

```
C          1.046244      0.027881     -0.167577
C          2.355936     -0.496513      0.241323
C          3.429022      0.290358      0.321135
O          0.738392      1.181834     -0.480804
O          0.076877     -0.939461     -0.193875
C         -1.246455     -0.547528     -0.580851
C         -1.989253      0.067737      0.589937
C         -3.425273      0.339622      0.228354
H          2.379047     -1.573520      0.472622
H          4.410907     -0.096648      0.626521
H          3.389910      1.364818      0.086548
H         -1.712582     -1.518885     -0.898548
H         -1.199775      0.164434     -1.445475
H         -1.936906     -0.625911      1.469475
H         -1.475849      1.021987      0.882868
H         -3.959977      0.796493      1.095793
H         -3.488220      1.045056     -0.635513
H         -3.950034     -0.606146     -0.050783
```

---

n-propylacrylate_4_am1

Datum	Value
AM1 Energy	-0.131813
AM1 Free Energy (Quasiharmonic)	-0.009744
Number of Imaginary Frequencies	0

Frequencies (Top 3 out of 48)

```
1.     36.0398 cm-1
2.     46.0196 cm-1
3.     71.0388 cm-1
```

AM1 Molecular Geometry in Cartesian Coordinates

```
C          1.185643      0.187932      0.000001
C          2.346513     -0.710999     -0.000001
C          3.593769     -0.240424     -0.000003
O          1.158494      1.422642     -0.000001
O         -0.006605     -0.485181      0.000004
C         -1.195671      0.324050      0.000006
```

```
C          -2.362814       -0.645363       -0.000004
C          -3.668099        0.106156       -0.000001
H           2.107461       -1.786561        0.000001
H           4.465967       -0.908550       -0.000004
H           3.816401        0.837290       -0.000005
H          -1.191762        0.973767        0.913473
H          -1.191757        0.973780       -0.913453
H          -2.295309       -1.307606        0.902384
H          -2.295305       -1.307593       -0.902402
H          -4.523152       -0.612197       -0.000008
H          -3.751854        0.754932        0.905436
H          -3.751850        0.754945       -0.905430
```

---

n-propylacrylate_4_reopt_am1_HEI

Datum	Value
AM1 Energy	-0.204563
AM1 Free Energy (Quasiharmonic)	-0.048245
Number of Imaginary Frequencies	0

Frequencies (Top 3 out of 63)

```
1.      18.8754 cm-1
2.      32.9022 cm-1
3.      44.8952 cm-1
```

AM1 Molecular Geometry in Cartesian Coordinates

```
C           0.178178        1.316783       -0.241137
C          -1.059661        1.068637       -0.836327
C          -1.546530       -0.217936       -1.217465
O           0.704600        2.393511        0.136278
O           0.992526        0.186382       -0.038498
C           2.266441        0.434169        0.539462
C           2.962525       -0.909899        0.690277
C           3.399052       -1.468345       -0.638518
H          -2.134682       -0.205903       -2.167606
H          -0.751423       -0.997372       -1.262256
H           2.853668        1.124200       -0.121759
H           2.135557        0.926275        1.539525
H           3.850746       -0.774416        1.360770
H           2.261343       -1.630216        1.187061
H           2.517254       -1.547881       -1.320842
H           3.851092       -2.481406       -0.511922
```

```
H          4.154989      -0.801154      -1.118775
S         -2.842051      -0.962448      -0.096191
C         -2.506408      -0.286651       1.486247
H         -1.743712       0.526652       1.392416
H         -3.443302       0.132694       1.913129
H         -2.120190      -1.075590       2.167824
H         -1.697119       1.943591      -0.995981
```

---

n-propylacrylate_5_am1

Datum	Value
AM1 Energy	-0.130516
AM1 Free Energy (Quasiharmonic)	-0.007686
Number of Imaginary Frequencies	0

Frequencies (Top 3 out of 48)

```
1.      32.3001 cm-1
2.      52.8609 cm-1
3.      80.9206 cm-1
```

AM1 Molecular Geometry in Cartesian Coordinates

```
C         -0.889901      -0.082400       0.220465
C         -2.170546      -0.392716      -0.429989
C         -3.221019       0.421387      -0.326513
O         -0.594617       0.896134       0.912971
O          0.054470      -1.048202      -0.000040
C          1.358027      -0.877132       0.573541
C          2.317308      -0.306808      -0.456445
C          2.353032       1.198890      -0.430313
H         -2.191598      -1.335485      -0.999778
H         -4.180760       0.194610      -0.810873
H         -3.184206       1.359975       0.246829
H          1.649500      -1.926642       0.851556
H          1.308314      -0.234049       1.490040
H          3.341687      -0.712937      -0.246183
H          2.016001      -0.664676      -1.476331
H          2.962691       1.588765      -1.280224
H          1.317344       1.613231      -0.507687
H          2.800799       1.566435       0.524725
```

---

n-propylacrylate_6_am1

Datum	Value
AM1 Energy	-0.13046
AM1 Free Energy (Quasiharmonic)	-0.007794
Number of Imaginary Frequencies	0

Frequencies (Top 3 out of 48)

```
1.      38.5759 cm-1
2.      53.5204 cm-1
3.      75.4435 cm-1
```

AM1 Molecular Geometry in Cartesian Coordinates

```
C          -0.999925       -0.584231       -0.071074
C          -2.287595        0.071803       -0.330543
C          -2.588176        1.309106        0.060929
O          -0.663615       -1.722945       -0.408616
O          -0.105184        0.179003        0.631766
C           1.175869       -0.395592        0.925018
C           2.119321       -0.284491       -0.257318
C           2.503962        1.142077       -0.547866
H          -2.992848       -0.567082       -0.889077
H          -3.565582        1.763259       -0.154489
H          -1.881872        1.942366        0.617728
H           1.530756        0.220746        1.794494
H           1.049111       -1.467713        1.228686
H           1.625872       -0.740623       -1.156569
H           3.036812       -0.888898       -0.031132
H           3.146509        1.193097       -1.459511
H           1.590875        1.762657       -0.719021
H           3.070019        1.581695        0.308810
```

---

n-propylacrylate_6_reopt_am1_HEI

Datum	Value
AM1 Energy	-0.205054
AM1 Free Energy (Quasiharmonic)	-0.048001
Number of Imaginary Frequencies	0

Frequencies (Top 3 out of 63)

```
1.      25.7749 cm-1
2.      34.5970 cm-1
3.      53.2374 cm-1
```

AM1 Molecular Geometry in Cartesian Coordinates

```
C        -0.324812       0.829676      -0.537381
C         0.852248       0.418542      -1.164128
C         1.519948      -0.821793      -0.930525
O        -0.986414       1.891213      -0.658722
O        -0.881783      -0.092458       0.370299
C        -2.080184       0.281924       1.024526
C        -3.304379       0.020779       0.159477
C        -3.521537      -1.448779      -0.083983
H         1.967513      -1.264781      -1.853802
H         0.886489      -1.574050      -0.406513
H        -2.101456      -0.361963       1.945538
H        -2.050270       1.365738       1.314027
H        -4.204025       0.462434       0.661234
H        -3.154960       0.554220      -0.817090
H        -4.364918      -1.611028      -0.797454
H        -3.760418      -1.978349       0.870111
H        -2.593693      -1.901148      -0.512983
S         3.064952      -0.727996       0.113348
C         2.811746       0.651440       1.165260
H         1.904855       1.215749       0.832039
H         3.700198       1.317944       1.117235
H         2.669276       0.314208       2.215096
H         1.289591       1.128196      -1.873092
```

---

n-propylacrylate_7_am1

Datum	Value
AM1 Energy	-0.130254
AM1 Free Energy (Quasiharmonic)	-0.008157
Number of Imaginary Frequencies	0

Frequencies (Top 3 out of 48)

```
1.      29.9373 cm-1
2.      49.0840 cm-1
3.      59.6302 cm-1
```

AM1 Molecular Geometry in Cartesian Coordinates

```
C          1.219650       -0.569462        0.079674
C          2.376107        0.333944        0.101348
C          2.307112        1.641857       -0.141838
O          1.216678       -1.785805        0.291602
O          0.026266        0.035706       -0.212259
C         -1.131328       -0.818997       -0.242687
C         -2.318941        0.071083       -0.556418
C         -2.753696        0.879465        0.637750
H          3.321930       -0.181123        0.341534
H          3.198987        2.283586       -0.115606
H          1.361786        2.150830       -0.380975
H         -0.977238       -1.595507       -1.036940
H         -1.234773       -1.323810        0.752810
H         -3.158986       -0.587987       -0.900567
H         -2.053126        0.758070       -1.402488
H         -3.607577        1.545245        0.365115
H         -3.078558        0.211285        1.471730
H         -1.909422        1.512872        1.003663
```

---

n-propylacrylate_8_am1_HEI

Datum	Value
AM1 Energy	-0.20541
AM1 Free Energy (Quasiharmonic)	-0.049359
Number of Imaginary Frequencies	0

Frequencies (Top 3 out of 63)

```
1.      22.5118 cm-1
2.      38.8966 cm-1
3.      41.4007 cm-1
```

AM1 Molecular Geometry in Cartesian Coordinates

```
C         -0.053856        1.321536        0.025128
C          1.264812        1.268653       -0.428935
C          1.838585        0.190748       -1.168194
O         -0.659406        2.205146        0.681881
O         -0.858966        0.217976       -0.316162
C         -2.207296        0.272679        0.126400
C         -2.869711       -1.016764       -0.333866
C         -4.312492       -1.060980        0.094557
H          2.559669        0.525772       -1.953461
H          1.081329       -0.500127       -1.605003
H         -2.715959        1.168959       -0.316731
H         -2.237503        0.365747        1.243921
H         -2.794388       -1.091676       -1.449659
H         -2.311144       -1.889838        0.092955
H         -4.395019       -1.004781        1.207134
H         -4.792632       -2.009547       -0.247232
H         -4.879358       -0.202101       -0.339942
S          2.975819       -0.938694       -0.208278
C          2.392813       -0.862996        1.443329
H          1.932106       -1.832251        1.733923
H          3.245026       -0.649389        2.124483
H          1.630409       -0.049144        1.534802
H          1.894199        2.125249       -0.169007
```

---

n-propylacrylate_8_am1

Datum	Value
AM1 Energy	-0.131387
AM1 Free Energy (Quasiharmonic)	-0.008852
Number of Imaginary Frequencies	0

Frequencies (Top 3 out of 48)

```
1.      40.6913 cm-1
2.      55.8677 cm-1
3.      74.5144 cm-1
```

AM1 Molecular Geometry in Cartesian Coordinates

```
C         -1.063782        0.541398        0.080150
C         -2.467504        0.241224       -0.228135
C         -2.985416       -0.986084       -0.233279
O         -0.524847        1.651705        0.091434
```

```
O          -0.304043       -0.555552        0.391712
C           1.078004       -0.342222        0.707056
C           1.904901       -0.214176       -0.558169
C           3.372993       -0.142386       -0.231978
H          -3.061023        1.142222       -0.459308
H          -4.043156       -1.167213       -0.470694
H          -2.389798       -1.881499       -0.002206
H           1.349230       -1.267291        1.283562
H           1.189316        0.567101        1.353140
H           1.700113       -1.090709       -1.226871
H           1.584169        0.710575       -1.107752
H           3.590238        0.739352        0.418574
H           3.706298       -1.064759        0.302709
H           3.970553       -0.043522       -1.170191
```

---

n-propylacrylate_9_am1_HEI

Datum	Value
AM1 Energy	-0.205947
AM1 Free Energy (Quasiharmonic)	-0.04905
Number of Imaginary Frequencies	0

Frequencies (Top 3 out of 63)

```
1.      27.6090 cm-1
2.      36.7615 cm-1
3.      50.9911 cm-1
```

AM1 Molecular Geometry in Cartesian Coordinates

```
C           0.238211        0.878711        0.213438
C          -1.019517        1.203666       -0.296085
C          -1.797128        0.375200       -1.160937
O           0.998780        1.520026        0.981189
O           0.760278       -0.360527       -0.206449
C           2.032226       -0.732129        0.290270
C           3.147564       -0.086642       -0.517958
C           4.499096       -0.585776       -0.083522
H          -2.355939        0.949250       -1.940144
H          -1.211282       -0.448123       -1.630553
H           2.056764       -1.850012        0.175666
H           2.134611       -0.457264        1.372992
H           3.075770        1.025109       -0.379256
H           2.988474       -0.301033       -1.606298
```

```
H         5.307186     -0.098693     -0.681001
H         4.577813     -1.691526     -0.222728
H         4.673449     -0.356795      0.995865
S        -3.243068     -0.496962     -0.364406
C        -2.767476     -0.716676      1.308642
H        -1.833261     -0.135548      1.512299
H        -3.581463     -0.352609      1.972512
H        -2.583750     -1.792848      1.518993
H        -1.429598      2.167356      0.021142
```

---

n-propylacrylate_9_am1

Datum	Value
AM1 Energy	-0.131132
AM1 Free Energy (Quasiharmonic)	-0.009217
Number of Imaginary Frequencies	0

Frequencies (Top 3 out of 48)

```
1.     41.1743 cm-1
2.     44.8987 cm-1
3.     61.0097 cm-1
```

AM1 Molecular Geometry in Cartesian Coordinates

```
C         1.274591      0.592567      0.000017
C         2.571881     -0.093840      0.000009
C         2.716700     -1.418101     -0.000028
O         1.073300      1.810776      0.000047
O         0.187182     -0.239615     -0.000013
C        -1.102947      0.396702     -0.000009
C        -2.124418     -0.725187     -0.000008
C        -3.521300     -0.161605     -0.000008
H         3.428295      0.602022      0.000036
H         3.708320     -1.892194     -0.000033
H         1.859826     -2.107847     -0.000055
H        -1.189187      1.040695     -0.913489
H        -1.189184      1.040694      0.913472
H        -1.966224     -1.371599     -0.902517
H        -1.966223     -1.371599      0.902501
H        -4.268666     -0.991431     -0.000006
H        -3.693929      0.469381      0.905470
H        -3.693930      0.469380     -0.905486
```

Created using ESIgen v0.0.5

Ketone Structures (AM1)

ESIgen is scientific software, funded by public research grants and published as:

```
J Rodriguez-Guerra, P Gomez-Orellana, JD Marechal.
J. Chem. Inf. Model., 2018, 58 (3), pp 561564.
DOI: 10.1021/acs.jcim.7b00714.
```

If you make use of ESIgen in scientific publications, please cite us in the main text! References only mentioned in SI documents are not indexed by citation engines.

1_pentene-3-one_trunc_1_am1_HEI

Datum	Value
AM1 Energy	-0.107567
AM1 Free Energy (Quasiharmonic)	-0.008916
Number of Imaginary Frequencies	0

Frequencies (Top 3 out of 42)

```
1.      32.4007 cm-1
2.      62.2793 cm-1
3.      86.1511 cm-1
```

AM1 Molecular Geometry in Cartesian Coordinates

```
C         -1.616430      -0.068980       0.147056
C         -0.570036      -0.728070      -0.505451
C          0.598501      -1.208081       0.162576
O         -1.703099       0.185953       1.383681
H         -0.631803      -0.866667      -1.589517
H          0.474504      -1.251983       1.269344
H          0.981424      -2.179784      -0.233699
C          1.570740       1.468948      -0.063517
H          1.909136       1.981123       0.863456
H          0.452899       1.473890      -0.100371
H          1.974114       2.013324      -0.944842
S          2.145614      -0.187842      -0.088248
```

```
C          -2.766002       0.360323      -0.741923
H          -3.206050      -0.528946      -1.251697
H          -3.560819       0.862561      -0.141621
H          -2.399065       1.069484      -1.520983
```

1_pentene-3-one_trunc_2_am1_HEI_reopt

Datum	Value
AM1 Energy	-0.107838
AM1 Free Energy (Quasiharmonic)	-0.008631
Number of Imaginary Frequencies	0

Frequencies (Top 3 out of 42)

```
  1.      29.7432 cm-1
  2.      63.8808 cm-1
  3.      98.0853 cm-1
```

AM1 Molecular Geometry in Cartesian Coordinates

```
C          -1.672574       0.007273      -0.148658
C          -0.588639      -0.562816      -0.831811
C           0.506875      -1.237297      -0.223205
O          -2.646266       0.592161      -0.706548
H          -0.580367      -0.462750      -1.923499
H           0.290374      -1.660092       0.785335
H           0.967418      -2.015963      -0.876580
C           1.532793       1.463685      -0.055396
H           1.620475       1.999038       0.915199
H           0.467402       1.492472      -0.399336
H           2.176656       1.970455      -0.806619
S           2.045374      -0.201424       0.132438
C          -1.685214      -0.086849       1.360470
H          -1.776089      -1.153772       1.674669
H          -0.734968       0.327523       1.774477
H          -2.546208       0.484617       1.781329
```

1_pentene-3-one_trunc_3_am1_HEI

Datum	Value

Datum	Value
AM1 Energy	-0.107567
AM1 Free Energy (Quasiharmonic)	-0.008918
Number of Imaginary Frequencies	0

Frequencies (Top 3 out of 42)

```
1.      32.3700 cm-1
2.      62.1027 cm-1
3.      86.0803 cm-1
```

AM1 Molecular Geometry in Cartesian Coordinates

```
C         -1.616560       -0.069176       -0.146961
C         -0.569948       -0.727128        0.506351
C          0.598446       -1.208171       -0.161149
O         -1.703653        0.183642       -1.383983
H         -0.631521       -0.864109        1.590633
H          0.981307       -2.179369        0.236423
H          0.474313       -1.253630       -1.267832
C          1.570973        1.469014        0.062149
H          1.975149        2.014147        0.942632
H          0.453165        1.474162        0.099882
H          1.908662        1.980253       -0.865595
S          2.145716       -0.187799        0.088141
C         -2.765888        0.361542        0.741654
H         -2.398569        1.071045        1.520214
H         -3.560399        0.863781        0.140956
H         -3.206473       -0.527116        1.252027
```

1_pentene-3-one_trunc_4_am1_HEI

Datum	Value
AM1 Energy	-0.107838
AM1 Free Energy (Quasiharmonic)	-0.008635
Number of Imaginary Frequencies	0

Frequencies (Top 3 out of 42)

```
1.      29.6658 cm-1
2.      63.8543 cm-1
3.      98.0570 cm-1
```

AM1 Molecular Geometry in Cartesian Coordinates

```
C          1.672606        0.007506        0.148707
C          0.588556       -0.561799        0.832319
C         -0.506863       -1.237015        0.224387
O          2.646108        0.593160        0.706128
H          0.580099       -0.460421        1.923890
H         -0.290252       -1.660931       -0.783654
H         -0.967391       -2.015003        0.878581
C         -1.533080        1.463709        0.053957
H         -0.467653        1.492936        0.397735
H         -2.176910        1.971027        0.804836
H         -1.620991        1.998215       -0.917082
S         -2.045431       -0.201634       -0.132367
C          1.685749       -0.088531       -1.360295
H          1.776596       -1.155869       -1.673100
H          0.735751        0.325396       -1.775252
H          2.546976        0.482293       -1.781555
```

1_pentene-3-one_trunc_5_am1_HEI

Datum	Value
AM1 Energy	-0.103359
AM1 Free Energy (Quasiharmonic)	-0.005383
Number of Imaginary Frequencies	0

Frequencies (Top 3 out of 42)

```
1.      41.0630 cm-1
2.      57.9909 cm-1
3.      63.2968 cm-1
```

AM1 Molecular Geometry in Cartesian Coordinates

```
C          1.870114        0.191019        0.005622
C          0.764806       -0.452331        0.574273
```

```
C          -0.484072       0.201908       0.808166
O           1.962252       1.413050      -0.307008
H           0.841457      -1.515470       0.821564
H          -0.955470      -0.039394       1.791395
H          -0.437385       1.306443       0.667531
C          -3.198480       0.327091      -0.040283
H          -3.126210       1.432180      -0.152569
H          -3.512135       0.106886       1.005012
H          -3.975803      -0.055405      -0.738085
S          -1.683420      -0.455681      -0.432910
C           3.085819      -0.686059      -0.214531
H           3.394654      -1.162351       0.745696
H           2.840342      -1.491004      -0.946866
H           3.938145      -0.085152      -0.610532
```

1_pentene-3-one_trunc_6_am1_HEI

Datum	Value
AM1 Energy	-0.103484
AM1 Free Energy (Quasiharmonic)	-0.004987
Number of Imaginary Frequencies	0

Frequencies (Top 3 out of 42)

```
1.      44.0599 cm-1
2.      58.1489 cm-1
3.      68.7773 cm-1
```

AM1 Molecular Geometry in Cartesian Coordinates

```
C          -1.925115      -0.078648      -0.010929
C          -0.827496      -0.859016       0.383214
C           0.412499      -0.349941       0.861087
O          -3.032108      -0.545064      -0.407465
H          -0.922782      -1.945529       0.275332
H           0.367869       0.673947       1.300538
H           0.938187      -1.035085       1.567950
C           3.076940       0.372633       0.101999
H           3.468458      -0.325524       0.875744
H           2.951624       1.373327       0.573359
H           3.822344       0.453562      -0.719803
S           1.573813      -0.207140      -0.580389
C          -1.787071       1.425171       0.065929
H          -1.682214       1.743873       1.130439
```

```
  H          -0.880256        1.751693       -0.496549
  H          -2.685898        1.923295       -0.368864
```

1_pentene-3-one_truncated_1_am1

Datum	Value
AM1 Energy	-0.036892
AM1 Free Energy (Quasiharmonic)	0.028009
Number of Imaginary Frequencies	0

Frequencies (Top 3 out of 27)

```
    1.      41.9253 cm-1
    2.     133.5319 cm-1
    3.     291.5239 cm-1
```

AM1 Molecular Geometry in Cartesian Coordinates

```
  C           0.763983        1.306900        0.000001
  H           0.296234        1.758732       -0.908307
  H           1.855888        1.543992       -0.000032
  C           0.549061       -0.173380       -0.000000
  H           0.296293        1.758720        0.908346
  C          -0.842608       -0.663936        0.000002
  O           1.499809       -0.966019       -0.000002
  C          -1.921469        0.116608       -0.000002
  H          -2.938795       -0.299623        0.000000
  H          -1.871040        1.214527       -0.000006
  H          -0.930852       -1.765349        0.000007
```

1_pentene-3-one_truncated_2_am1

Datum	Value
AM1 Energy	-0.038392
AM1 Free Energy (Quasiharmonic)	0.026408
Number of Imaginary Frequencies	0

Frequencies (Top 3 out of 27)

```
1.      61.4137 cm-1
2.      97.6091 cm-1
3.     268.2836 cm-1
```

AM1 Molecular Geometry in Cartesian Coordinates

```
C        1.716368      -0.620130       0.000000
H        1.758423      -1.270485       0.907535
H        2.600296       0.062476       0.000029
C        0.441693       0.162629       0.000000
H        1.758448      -1.270441      -0.907564
C       -0.790273      -0.646001      -0.000000
O        0.432545       1.400584      -0.000000
C       -2.009741      -0.105925       0.000000
H       -2.923026      -0.716141       0.000000
H       -2.164034       0.983886       0.000001
H       -0.638750      -1.737405      -0.000002
```

2_2cyclopentene1one_1_am1_HEI

Datum	Value
AM1 Energy	-0.100657
AM1 Free Energy (Quasiharmonic)	0.008225
Number of Imaginary Frequencies	0

Frequencies (Top 3 out of 45)

```
1.      55.2692 cm-1
2.      83.1112 cm-1
3.     118.9315 cm-1
```

AM1 Molecular Geometry in Cartesian Coordinates

```
C        1.619492       0.855764       0.723714
C        0.366788       1.586648       0.246688
C       -0.325765       0.648907      -0.749059
C        0.561136      -0.475746      -0.946068
C        1.688011      -0.430424      -0.110190
H        2.538424       1.467698       0.562430
H        1.558002       0.602002       1.808783
```

```
H          0.640052        2.545273       -0.261635
H         -0.310051        1.834359        1.100076
O          2.651504       -1.229922        0.005939
H          0.372357       -1.256284       -1.673966
H         -0.653472        1.175305       -1.677393
C         -1.837942       -1.343109        0.683599
H         -1.998931       -1.245186        1.779358
H         -0.808472       -1.738438        0.493782
H         -2.588030       -2.052558        0.271177
S         -2.024515        0.215935       -0.096389
```

2_2-cyclopentene-1-one_1_am1

Datum	Value
AM1 Energy	-0.034795
AM1 Free Energy (Quasiharmonic)	0.040428
Number of Imaginary Frequencies	0

Frequencies (Top 3 out of 30)

```
1.     127.7337 cm-1
2.     306.6302 cm-1
3.     477.1257 cm-1
```

AM1 Molecular Geometry in Cartesian Coordinates

```
C         -0.041540       -1.190483       -0.000000
C         -1.464463       -0.618833        0.000000
C         -1.284748        0.872058       -0.000000
C          0.014630        1.227361       -0.000000
C          0.879040        0.018810        0.000000
H          0.150883       -1.816894        0.905044
H          0.150882       -1.816892       -0.905045
H         -2.034751       -0.946417        0.904887
H         -2.034752       -0.946418       -0.904886
O          2.107655       -0.011860       -0.000000
H          0.440764        2.231702        0.000001
H         -2.151784        1.536317        0.000000
```

2_2cyclopentene1one_2_am1_HEI

Datum	Value
AM1 Energy	-0.100657
AM1 Free Energy (Quasiharmonic)	0.008227
Number of Imaginary Frequencies	0

Frequencies (Top 3 out of 45)

```
1.      55.3106 cm-1
2.      83.1191 cm-1
3.     118.9355 cm-1
```

AM1 Molecular Geometry in Cartesian Coordinates

```
C         -1.619315        0.855731       -0.723828
C         -0.366713        1.586681       -0.246628
C          0.325859        0.648880        0.749053
C         -0.561120       -0.475741        0.946083
C         -1.687979       -0.430372        0.110206
H         -2.538285        1.467685       -0.562834
H         -1.557571        0.601820       -1.808850
H         -0.640137        2.545193        0.261826
H          0.310160        1.834589       -1.099931
O         -2.651549       -1.229784       -0.005874
H         -0.372356       -1.256300        1.673957
H          0.653494        1.175266        1.677432
C          1.837742       -1.343147       -0.683637
H          2.587918       -2.052584       -0.271347
H          1.998532       -1.245200       -1.779424
H          0.808318       -1.738461       -0.493622
S          2.024467        0.215879        0.096393
```

2_2cyclopentene1one_3_am1_HEI

Datum	Value
AM1 Energy	-0.096283
AM1 Free Energy (Quasiharmonic)	0.011941
Number of Imaginary Frequencies	0

Frequencies (Top 3 out of 45)

```
1.      57.7568 cm-1
2.      73.0557 cm-1
3.      85.1042 cm-1
```

AM1 Molecular Geometry in Cartesian Coordinates

```
C         -1.588592       1.183259       -0.335832
C         -0.180337       1.384743        0.217895
C          0.288319       0.008809        0.704892
C         -0.842951      -0.885324        0.587973
C         -1.948921      -0.276705       -0.026899
H         -2.321212       1.872984        0.146182
H         -1.622285       1.354093       -1.438158
H         -0.192324       2.108662        1.071138
H          0.508546       1.789845       -0.562732
O         -3.089793      -0.732015       -0.294079
H         -0.816620      -1.917153        0.917693
H          0.771468       0.060382        1.708868
C          3.038430       0.268817       -0.052567
H          2.903338       1.331166       -0.357212
H          3.879656      -0.167815       -0.635154
H          3.300644       0.251194        1.029171
S          1.606965      -0.683052       -0.381246
```

3_3methyl3pentene2one_1_am1_HEI_reopt

Datum	Value
AM1 Energy	-0.123756
AM1 Free Energy (Quasiharmonic)	0.027973
Number of Imaginary Frequencies	0

Frequencies (Top 3 out of 60)

```
1.      30.9094 cm-1
2.      69.3381 cm-1
3.      78.5955 cm-1
```

AM1 Molecular Geometry in Cartesian Coordinates

```
```

```
C          -1.658843       -0.252656       -0.395450
C          -0.589192        0.510045        0.109173
C           0.655057        0.568678       -0.623329
O          -1.639438       -0.938258       -1.459798
H           0.538253        0.106728       -1.637770
C           1.276727       -1.943874        0.603985
H           1.626694       -2.779883       -0.040255
H           1.527121       -2.174445        1.662358
H           0.169058       -1.835871        0.498344
S           2.059596       -0.447765        0.129666
C          -2.950022       -0.238471        0.392597
H          -3.249538        0.810340        0.627277
H          -3.765959       -0.727575       -0.190961
H          -2.814047       -0.793482        1.351505
C           1.313341        1.918794       -0.723750
H           2.300566        1.840980       -1.242853
H           0.658005        2.609331       -1.310098
H           1.479166        2.367428        0.285093
C          -0.719988        1.250027        1.373201
H           0.283350        1.466282        1.820321
H          -1.245908        2.231741        1.227779
H          -1.307282        0.673476        2.134435
```

3_3methyl3pentene2one_2_reopt_am1_HEI

Datum	Value
AM1 Energy	-0.124587
AM1 Free Energy (Quasiharmonic)	0.02787
Number of Imaginary Frequencies	0

Frequencies (Top 3 out of 60)

```
1.      34.5395 cm-1
2.      55.6552 cm-1
3.      85.3637 cm-1
```

AM1 Molecular Geometry in Cartesian Coordinates

```
C          -1.729752        0.168570        0.172735
C          -0.606189       -0.651825       -0.072488
C           0.600604       -0.559192        0.698282
O          -2.822926        0.107210       -0.459038
H           0.439992       -0.139964        1.723390
```

```
C          1.088769       1.618957      -1.203592
H          1.676564       1.548520      -2.144596
H          0.064479       1.200690      -1.374061
H          1.003580       2.688099      -0.909557
S          1.893826       0.716710       0.063572
C         -1.623070       1.203557       1.269236
H         -2.522405       1.864403       1.267763
H         -1.551923       0.701884       2.263500
H         -0.709860       1.827969       1.113947
C          1.441019      -1.805052       0.770319
H          0.861318      -2.624816       1.263498
H          1.735097      -2.148416      -0.250636
H          2.370633      -1.619856       1.362729
C         -0.673719      -1.629629      -1.174077
H         -1.647165      -1.544450      -1.718549
H          0.156309      -1.470235      -1.911178
H         -0.580383      -2.681185      -0.793587
```

3_3methyl3pentene2one_3_am1_HEI

Datum	Value
AM1 Energy	-0.119269
AM1 Free Energy (Quasiharmonic)	0.032372
Number of Imaginary Frequencies	0

Frequencies (Top 3 out of 60)

```
1.      37.6852 cm-1
2.      59.1108 cm-1
3.      74.5371 cm-1
```

AM1 Molecular Geometry in Cartesian Coordinates

```
C          1.832533      -0.573360       0.160722
C          0.807891       0.386647       0.051706
C         -0.499905       0.094198       0.596143
O          1.741793      -1.688633       0.751316
H         -0.494178      -0.875524       1.159153
C         -3.110918      -0.813665      -0.179085
H         -3.693491      -1.282640      -1.002969
H         -2.909768      -1.578183       0.604435
H         -3.725255      -0.004212       0.276113
S         -1.620080      -0.197758      -0.859979
C          3.168709      -0.222150      -0.455277
```

```
H          3.936268       -0.984514       -0.181363
H          3.073344       -0.191937       -1.566965
H          3.508541        0.779407       -0.099655
C         -1.119945        1.177738        1.439727
H         -0.484475        1.357909        2.341836
H         -1.202295        2.136056        0.873323
H         -2.142017        0.880142        1.781744
C          1.048993        1.673214       -0.617500
H          1.649698        2.370810        0.026930
H          1.619284        1.542413       -1.574192
H          0.087137        2.187731       -0.867872
```

3_3methyl3pentene2one_4_am1_HEI

Datum	Value
AM1 Energy	-0.120395
AM1 Free Energy (Quasiharmonic)	0.032087
Number of Imaginary Frequencies	0

Frequencies (Top 3 out of 60)

```
1.      34.0990 cm-1
2.      60.3615 cm-1
3.      80.8549 cm-1
```

AM1 Molecular Geometry in Cartesian Coordinates

```
C         -1.593844       -0.137567       -0.351845
C         -0.677463        0.249769        0.647839
C          0.583980       -0.375748        0.939338
O         -2.722198        0.405425       -0.536825
H          1.004661        0.029736        1.897865
C          1.437726        1.417664       -1.195631
H          1.620166        1.209977       -2.272574
H          0.340414        1.557096       -1.024525
H          1.974767        2.348432       -0.910289
S          2.018543        0.065979       -0.243473
C         -1.214564       -1.254270       -1.294491
H         -1.886643       -1.251192       -2.185945
H         -0.156535       -1.127191       -1.630670
H         -1.319153       -2.237730       -0.777236
C          0.665253       -1.879027        0.963318
H         -0.272898       -2.305373        1.395980
H          0.809296       -2.303472       -0.059578
```

```
H          1.527581       -2.210827        1.593723
C         -1.011438        1.433126        1.470244
H         -0.234211        2.234949        1.363190
H         -1.999234        1.861047        1.167857
H         -1.065218        1.171800        2.559729
```

3_3methyl3pentene2one_5_am1_HEI

Datum	Value
AM1 Energy	-0.124587
AM1 Free Energy (Quasiharmonic)	0.027863
Number of Imaginary Frequencies	0

Frequencies (Top 3 out of 60)

```
1.      34.4574 cm-1
2.      55.6426 cm-1
3.      85.3394 cm-1
```

AM1 Molecular Geometry in Cartesian Coordinates

```
C          1.729817       -0.168913        0.172735
C          0.606453        0.651792       -0.072430
C         -0.600330        0.559366        0.698331
O          2.823131       -0.107540       -0.458779
H         -0.439822        0.140062        1.723409
C         -1.089655       -1.618554       -1.203950
H         -1.004772       -2.687817       -0.910281
H         -1.677757       -1.547593       -2.144720
H         -0.065285       -1.200629       -1.374673
S         -1.893950       -0.716461        0.063818
C          1.622646       -1.204302        1.268812
H          2.521174       -1.866233        1.266410
H          1.552902       -0.702930        2.263330
H          0.708556       -1.827522        1.113977
C         -1.440547        1.805355        0.770316
H         -0.860661        2.625109        1.263284
H         -1.734672        2.148603       -0.250660
H         -2.370105        1.620403        1.362877
C          0.674225        1.629642       -1.173956
H          0.580487        2.681171       -0.793518
H          1.647907        1.544663       -1.718023
H         -0.155457        1.470100       -1.911407
```

3_3methyl3pentene2one_6_am1_HEI

Datum	Value
AM1 Energy	-0.124587
AM1 Free Energy (Quasiharmonic)	0.027873
Number of Imaginary Frequencies	0

Frequencies (Top 3 out of 60)

```
1.      34.6264 cm-1
2.      55.7980 cm-1
3.      85.3654 cm-1
```

AM1 Molecular Geometry in Cartesian Coordinates

```
C        -1.729712      -0.168819      -0.172867
C        -0.606376       0.651946       0.072302
C         0.600480       0.559438      -0.698332
O        -2.823208      -0.107125       0.458293
H         0.439996       0.140218      -1.723470
C         1.089061      -1.618621       1.203937
H         1.004035      -2.687854       0.910181
H         1.676905      -1.547862       2.144893
H         0.064708      -1.200442       1.374389
S         1.893793      -0.716621      -0.063593
C        -1.622262      -1.204632      -1.268501
H        -1.552383      -0.703575      -2.263190
H        -0.708137      -1.827733      -1.113320
H        -2.520742      -1.866644      -1.265976
C         1.440906       1.805274      -0.770241
H         0.861307       2.625020      -1.263591
H         1.734779       2.148710       0.250753
H         2.370644       1.620039      -1.362454
C        -0.674263       1.629838       1.173787
H        -0.580854       2.681390       0.793304
H        -1.647867       1.544642       1.717984
H         0.155578       1.470482       1.911120
```

3_3methyl3pentene2one_7_reopt_am1_HEI

Datum	Value

Datum	Value
AM1 Energy	-0.122068
AM1 Free Energy (Quasiharmonic)	0.029907
Number of Imaginary Frequencies	0

Frequencies (Top 3 out of 60)

```
1.      27.1684 cm-1
2.      55.9809 cm-1
3.      65.3658 cm-1
```

AM1 Molecular Geometry in Cartesian Coordinates

```
C         -1.534483       0.401679       0.208378
C         -0.623237      -0.364855      -0.540269
C          0.659022       0.121692      -0.990705
O         -1.383529       1.594564       0.602684
H          0.996499      -0.398641      -1.927495
C          1.533988      -0.265571       1.740525
H          2.078162       0.549497       2.265874
H          0.440947      -0.032453       1.731017
H          1.704353      -1.221218       2.282533
S          2.118256      -0.416470       0.093487
C         -2.839939      -0.273931       0.575905
H         -3.514406       0.445034       1.099052
H         -3.352469      -0.648933      -0.341399
H         -2.643458      -1.139794       1.252032
C          0.796371       1.610190      -1.145650
H         -0.015118       1.997994      -1.809833
H          0.695590       2.117831      -0.154885
H          1.786380       1.871856      -1.592318
C         -0.961975      -1.760225      -0.880776
H         -1.231419      -2.356837       0.030017
H         -1.841527      -1.811435      -1.576722
H         -0.105890      -2.279764      -1.379587
```

3-methyl-3-pentene-2-one_1_am1_reopt

Datum	Value
AM1 Energy	-0.062835
AM1 Free Energy (Quasiharmonic)	0.054279

Datum	Value
Number of Imaginary Frequencies	0

Frequencies (Top 3 out of 45)

```
1.      31.9436 cm-1
2.      66.1701 cm-1
3.      87.0970 cm-1
```

AM1 Molecular Geometry in Cartesian Coordinates

```
C          2.579068      -0.550332      -0.091608
H          2.868254       0.135788       0.744303
H          2.929924      -0.090094      -1.051058
C          1.118019      -0.748493      -0.131471
H          3.110924      -1.523096       0.047167
C          0.226667       0.247432       0.016989
C         -1.232960      -0.000558      -0.051475
C         -1.703315      -1.392589       0.236421
O         -2.035433       0.900376      -0.327373
H         -2.809104      -1.403109       0.397363
H         -1.464444      -2.054786      -0.631837
H         -1.198195      -1.794312       1.147155
H          0.782594      -1.784472      -0.308912
C          0.643938       1.652213       0.245616
H         -0.205088       2.357052       0.061168
H          0.991506       1.781451       1.301703
H          1.488586       1.926535      -0.434890
```

3-methyl-3-pentene-2-one_2_am1_reopt

Datum	Value
AM1 Energy	-0.063721
AM1 Free Energy (Quasiharmonic)	0.053586
Number of Imaginary Frequencies	0

Frequencies (Top 3 out of 45)

```
1.      18.5974 cm-1
2.      61.6858 cm-1
3.      88.6111 cm-1
```

AM1 Molecular Geometry in Cartesian Coordinates

```
C         -2.699981      -0.245194       0.021283
H         -2.930863       0.377503      -0.880064
H         -2.928793       0.365078       0.931854
C         -1.286969      -0.664194       0.018511
H         -3.378526      -1.133370       0.016087
C         -0.230119       0.170720      -0.006619
C          1.138563      -0.395364      -0.005668
C          2.278465       0.572054       0.049068
O          1.340704      -1.617736      -0.044671
H          3.252923       0.029641      -0.020727
H          2.206242       1.299649      -0.795262
H          2.248062       1.136405       1.012999
H         -1.105723      -1.755861       0.039220
C         -0.355618       1.645466      -0.041497
H          0.213241       2.108626       0.803393
H          0.055190       2.047180      -1.002041
H         -1.423431       1.966109       0.041443
```

4_4-methyl-3-pentene-2-one_1_am1

Datum	Value
AM1 Energy	-0.066135
AM1 Free Energy (Quasiharmonic)	0.051166
Number of Imaginary Frequencies	0

Frequencies (Top 3 out of 45)

```
1.     44.2906 cm-1
2.     94.5686 cm-1
3.     99.8147 cm-1
```

AM1 Molecular Geometry in Cartesian Coordinates

```
C         -2.413898      -0.976257      -0.000012
H         -2.170965      -2.066738      -0.000248
H         -3.030345      -0.749510      -0.906172
C         -1.191997      -0.134689      -0.000003
H         -3.030126      -0.749857       0.906382
C          0.035676      -0.690014      -0.000026
```

```
C          1.294828       0.060350      -0.000018
C          2.531493      -0.785102       0.000046
O          1.359654       1.298533      -0.000056
H          3.446911      -0.145579       0.000284
H          2.542138      -1.436590       0.907425
H          2.542411      -1.436295      -0.907542
C         -1.415258       1.329206       0.000048
H          0.147655      -1.787008      -0.000057
H         -0.932783       1.791618       0.899540
H         -0.933771       1.791545      -0.899999
H         -2.503418       1.579184       0.000629
```

4_4methyl3pentene2one_1_reopt_am1_HEI

Datum	Value
AM1 Energy	-0.115023
AM1 Free Energy (Quasiharmonic)	0.035808
Number of Imaginary Frequencies	0

Frequencies (Top 3 out of 60)

```
1.      33.1546 cm-1
2.      52.4727 cm-1
3.      74.9287 cm-1
```

AM1 Molecular Geometry in Cartesian Coordinates

```
C         -1.827197      -0.055535       0.040976
C         -0.656600      -0.388901      -0.650534
C          0.601891      -0.764636      -0.079277
O         -2.023822      -0.052980       1.289769
C          0.896510       2.157678       0.106316
H          1.140627       2.895188      -0.689261
H         -0.185040       1.880142       0.035975
H          1.090995       2.620596       1.098543
S          1.883365       0.726132      -0.100566
C         -2.995137       0.332623      -0.846398
H         -2.730468       1.234240      -1.447561
H         -3.237318      -0.501929      -1.545582
H         -3.896216       0.560769      -0.229760
C          0.581457      -1.184945       1.366296
H         -0.068345      -2.087237       1.486025
H          1.610184      -1.428187       1.726479
H          0.150876      -0.368997       1.997182
```

```
H          -0.684916      -0.320050      -1.745807
C           1.405836      -1.722346      -0.929866
H           2.460250      -1.790654      -0.564915
H           0.948014      -2.741318      -0.880201
H           1.417538      -1.390456      -1.995299
```

4_4-methyl-3-pentene-2-one_2_am1

Datum	Value
AM1 Energy	-0.066135
AM1 Free Energy (Quasiharmonic)	0.051173
Number of Imaginary Frequencies	0

Frequencies (Top 3 out of 45)

```
  1.      44.4700 cm-1
  2.      94.7425 cm-1
  3.     100.0168 cm-1
```

AM1 Molecular Geometry in Cartesian Coordinates

```
C          -2.414016      -0.976139      -0.000106
H          -2.171178      -2.066646      -0.000788
H          -3.030575      -0.748987      -0.906093
C          -1.192023      -0.134711       0.000058
H          -3.030101      -0.750034       0.906466
C           0.035612      -0.690090       0.000010
C           1.294781       0.060323      -0.000063
C           2.531434      -0.785144       0.000094
O           1.359560       1.298492      -0.000209
H           2.541978      -1.437046      -0.906991
H           3.446864      -0.145624      -0.000431
H           2.542425      -1.435937       0.907968
C          -1.414948       1.329249       0.000150
H           0.147721      -1.787068       0.000004
H          -0.932649       1.791263       0.899955
H          -0.932987       1.791592      -0.899647
H          -2.503018       1.579622       0.000374
```

4_4methyl3pentene2one_2_reopt_am1_HEI

Datum	Value
AM1 Energy	-0.115023
AM1 Free Energy (Quasiharmonic)	0.035806
Number of Imaginary Frequencies	0

Frequencies (Top 3 out of 60)

```
1.      33.1104 cm-1
2.      52.4500 cm-1
3.      74.8423 cm-1
```

AM1 Molecular Geometry in Cartesian Coordinates

```
C          1.827164        0.055554       -0.040981
C          0.656603        0.389099        0.650488
C         -0.601933        0.764648        0.079189
O          2.023720        0.052631       -1.289788
C         -0.896411       -2.157696       -0.106122
H         -1.091046       -2.620694       -1.098283
H          0.185137       -1.880144       -0.035986
H         -1.140381       -2.895151        0.689549
S         -1.883286       -0.726168        0.100757
C          2.995175       -0.332319        0.846428
H          2.730445       -1.233559        1.448127
H          3.896127       -0.560959        0.229789
H          3.237606        0.502569        1.545123
C         -1.405902        1.722493        0.929611
H         -0.948168        2.741491        0.879685
H         -2.460345        1.790642        0.564714
H         -1.417508        1.390851        1.995121
H          0.684974        0.320564        1.745778
C         -0.581537        1.184666       -1.366472
H         -1.610284        1.427753       -1.726701
H          0.068187        2.086987       -1.486394
H         -0.150891        0.368615       -1.997182
```

4_4methyl3pentene2one_3_am1_HEI

Datum	Value
AM1 Energy	-0.112158
AM1 Free Energy (Quasiharmonic)	0.039878

Datum	Value
Number of Imaginary Frequencies	0

Frequencies (Top 3 out of 60)

```
1.      35.4631 cm-1
2.      53.0691 cm-1
3.      91.3905 cm-1
```

AM1 Molecular Geometry in Cartesian Coordinates

```
C         1.871231        0.079067        0.234142
C         0.642736        0.516813        0.762880
C        -0.582931        0.816449        0.110199
O         2.902212       -0.098315        0.946082
C        -0.754237       -2.173892        0.315030
H        -0.552438       -2.870895       -0.527938
H         0.219472       -1.797375        0.720640
H        -1.296549       -2.723986        1.114827
S        -1.719037       -0.826618       -0.244321
C         1.996322       -0.223228       -1.238283
H         2.974110       -0.719127       -1.450143
H         1.168246       -0.901857       -1.556593
H         1.943941        0.722103       -1.828940
C        -1.561601        1.566443        0.986209
H        -1.218597        2.623612        1.114440
H        -2.580484        1.576268        0.527859
H        -1.631954        1.092061        1.993872
H         0.629491        0.600182        1.861312
C        -0.560827        1.384646       -1.284483
H        -1.569124        1.778173       -1.566497
H         0.174191        2.225833       -1.340850
H        -0.277563        0.609621       -2.035660
```

4_4methyl3pentene2one_4_am1_HEI

Datum	Value
AM1 Energy	-0.112158
AM1 Free Energy (Quasiharmonic)	0.039874
Number of Imaginary Frequencies	0

Frequencies (Top 3 out of 60)

```
1.      35.4895 cm-1
2.      53.1511 cm-1
3.      91.3619 cm-1
```

AM1 Molecular Geometry in Cartesian Coordinates

```
C         -1.871308        0.078893       -0.234098
C         -0.642931        0.517051       -0.762860
C          0.582573        0.816891       -0.110144
O         -2.902360       -0.098334       -0.945937
C          0.754635       -2.173742       -0.315468
H          1.296980       -2.723537       -1.115448
H          0.552877       -2.871041        0.527270
H         -0.219099       -1.797199       -0.720942
S          1.719339       -0.826567        0.244245
C         -1.996062       -0.223976        1.238233
H         -2.973740       -0.720076        1.450119
H         -1.167818       -0.902592        1.556139
H         -1.943681        0.721143        1.829231
C          1.561315        1.566825       -0.986042
H          1.218034        2.623837       -1.114822
H          2.580030        1.577118       -0.527340
H          1.632164        1.092082       -1.993500
H         -0.629730        0.600547       -1.861282
C          0.560461        1.384512        1.284738
H         -0.175614        2.224734        1.341838
H          0.278566        0.608806        2.035737
H          1.568391        1.779211        1.566423
```

4_4methyl3pentene2one_5_reopt_am1_HEI

Datum	Value
AM1 Energy	-0.111043
AM1 Free Energy (Quasiharmonic)	0.040029
Number of Imaginary Frequencies	0

Frequencies (Top 3 out of 60)

```
1.      45.1109 cm-1
2.      57.3391 cm-1
3.      71.7226 cm-1
```

AM1 Molecular Geometry in Cartesian Coordinates

```
C          2.047840      -0.078629      -0.133989
C          0.889993      -0.103425       0.652192
C         -0.374880       0.502477       0.331005
O          2.245651       0.547694      -1.214109
C         -3.023287      -0.565188      -0.521918
H         -3.534350      -1.448698      -0.965129
H         -3.575444      -0.255251       0.393927
H         -3.061450       0.276625      -1.249136
S         -1.373987      -1.028027      -0.161560
C          3.205834      -0.884695       0.423775
H          2.922503      -1.962223       0.476995
H          4.105776      -0.778969      -0.226782
H          3.459268      -0.532536       1.451317
C         -1.087677       1.093753       1.528524
H         -0.542895       2.005966       1.875826
H         -2.134105       1.386084       1.266922
H         -1.120770       0.359977       2.368583
H          0.922159      -0.677677       1.586436
C         -0.378218       1.462666      -0.829900
H         -1.408135       1.849707      -1.026781
H          0.296067       2.325543      -0.605656
H          0.012336       0.956579      -1.746820
```

4_4methyl3pentene2one_6_am1_HEI_reopt

Datum	Value
AM1 Energy	-0.112158
AM1 Free Energy (Quasiharmonic)	0.039874
Number of Imaginary Frequencies	0

Frequencies (Top 3 out of 60)

```
1.      35.4956 cm-1
2.      53.1673 cm-1
3.      91.3641 cm-1
```

AM1 Molecular Geometry in Cartesian Coordinates

```
C          1.871333      -0.078953      -0.234007
C          0.642984      -0.517188      -0.762786
```

```
C          -0.582573       -0.816900       -0.110105
O           2.902492        0.097965       -0.945764
C          -0.754687        2.173707       -0.315941
H          -1.297116        2.723158       -1.116103
H          -0.552979        2.871326        0.526545
H           0.219074        1.797120       -0.721315
S          -1.719202        0.826628        0.244324
C           1.995850        0.224441        1.238230
H           2.973252        0.721121        1.450024
H           1.167216        0.902716        1.555865
H           1.943947       -0.720559        1.829472
C          -1.561304       -1.566871       -0.985998
H          -1.218104       -2.623937       -1.114569
H          -2.580069       -1.576985       -0.527404
H          -1.632000       -1.092281       -1.993541
H           0.629881       -0.600939       -1.861190
C          -0.560593       -1.384520        1.284782
H          -1.568769       -1.778451        1.566665
H           0.174857       -2.225300        1.341700
H          -0.277951       -0.609058        2.035740
```

4-hexene-3-one_10_am1_HEI

Datum	Value
AM1 Energy	-0.119434
AM1 Free Energy (Quasiharmonic)	0.032076
Number of Imaginary Frequencies	0

Frequencies (Top 3 out of 60)

```
1.      34.7078 cm-1
2.      51.5953 cm-1
3.      67.4812 cm-1
```

AM1 Molecular Geometry in Cartesian Coordinates

```
C          -3.151346       -1.326966        0.420160
C          -1.792792       -0.704095        0.595145
C          -1.617053        0.569682       -0.215298
C          -0.335229        1.143235       -0.241176
C           0.811691        0.639025        0.444247
C           1.849392        1.677505        0.781589
O          -2.624565        1.055901       -0.803116
H          -3.253589       -2.242080        1.052706
```

```
H          -3.950042       -0.599273        0.702825
H          -3.311201       -1.607835       -0.648624
H          -0.994003       -1.428570        0.283404
H          -1.626270       -0.460204        1.677263
H           0.579469        0.017272        1.345355
H           1.436064        2.396284        1.531665
H           2.764040        1.202395        1.213970
H           2.144035        2.251987       -0.129129
C           2.934985       -1.341112        0.096302
H           3.237518       -2.243620       -0.479849
H           3.810775       -0.657911        0.173474
H           2.646218       -1.649171        1.126147
S           1.594782       -0.587508       -0.739916
H          -0.200898        2.030002       -0.871434
```

4-hexene-3-one_11_reopt_am1_HEI

Datum	Value
AM1 Energy	-0.123899
AM1 Free Energy (Quasiharmonic)	0.028109
Number of Imaginary Frequencies	0

Frequencies (Top 3 out of 60)

```
   1.      37.2129 cm-1
   2.      38.3433 cm-1
   3.      75.2718 cm-1
```

AM1 Molecular Geometry in Cartesian Coordinates

```
C           3.150207       -0.660788       -0.821076
C           1.735585       -0.148121       -0.844977
C           1.342980        0.566322        0.437905
C           0.003888        0.969391        0.565543
C          -1.015389        0.783930       -0.412606
C          -2.142985        1.778731       -0.357260
O           2.231328        0.764511        1.314928
H           3.410308       -1.154058       -1.788910
H           3.862407        0.179921       -0.638647
H           3.279399       -1.399428        0.006261
H           1.021495       -0.999488       -1.004977
H           1.607244        0.565289       -1.700749
H          -0.634133        0.667500       -1.457136
H          -2.543724        1.866278        0.680999
```

```
H          -1.774728       2.785201       -0.677825
H          -2.977585       1.474618       -1.035551
C          -1.101726      -1.849149        0.873014
H          -0.251180      -1.252723        1.291394
H          -1.773725      -2.163136        1.701103
H          -0.699987      -2.756119        0.370264
S          -1.995877      -0.876560       -0.276094
H          -0.277744       1.453119        1.508603
```

4-hexene-3-one_12_reopt_am1_HEI

Datum	Value
AM1 Energy	-0.123899
AM1 Free Energy (Quasiharmonic)	0.02811
Number of Imaginary Frequencies	0

Frequencies (Top 3 out of 60)

```
1.      37.2735 cm-1
2.      38.3712 cm-1
3.      75.3480 cm-1
```

AM1 Molecular Geometry in Cartesian Coordinates

```
C          -3.150230      -0.660651       -0.821153
C          -1.735476      -0.148345       -0.844875
C          -1.342980       0.566324        0.437909
C          -0.003909       0.969499        0.565522
C           1.015366       0.783996       -0.412607
C           2.142996       1.778751       -0.357320
O          -2.231350       0.764538        1.314898
H          -3.410107      -1.154468       -1.788769
H          -3.279888      -1.398726        0.006620
H          -3.862299       0.180343       -0.639526
H          -1.606751       0.564793       -1.700818
H          -1.021549      -0.999927       -1.004482
H           0.634146       0.667429       -1.457132
H           2.977620       1.474513       -1.035526
H           1.774804       2.785201       -0.678030
H           2.543684       1.866410        0.680950
C           1.101602      -1.849231        0.872923
H           0.251012      -1.252826        1.291237
H           0.699913      -2.756156        0.370048
H           1.773490      -2.163287        1.701077
```

```
S          1.995926       -0.876562       -0.275985
H          0.277686        1.453337        1.508536
```

4-hexene-3-one_13_am1_HEI

Datum	Value
AM1 Energy	-0.124298
AM1 Free Energy (Quasiharmonic)	0.028038
Number of Imaginary Frequencies	0

Frequencies (Top 3 out of 60)

```
1.      39.5920 cm-1
2.      52.9595 cm-1
3.      65.5670 cm-1
```

AM1 Molecular Geometry in Cartesian Coordinates

```
C         -2.804718        0.961476       -0.858173
C         -2.676931       -0.427138       -0.289231
C         -1.347487       -0.602118        0.437273
C         -0.222689       -0.720179       -0.384671
C          1.110570       -0.846885        0.124745
C          2.009987       -1.778574       -0.642475
O         -1.377274       -0.644230        1.700651
H         -3.769352        1.078455       -1.409854
H         -2.770655        1.721209       -0.040076
H         -1.961761        1.170005       -1.561611
H         -2.748039       -1.183044       -1.112937
H         -3.516117       -0.622083        0.427672
H          1.123444       -1.067837        1.220863
H          1.959627       -1.568129       -1.737810
H          3.072285       -1.676692       -0.309127
H          1.686559       -2.835452       -0.472524
C          0.951250        2.039177        0.223224
H          1.110957        2.808368       -0.563609
H          1.061660        2.515427        1.221910
H         -0.082369        1.619065        0.129811
S          2.130400        0.758085        0.025547
H         -0.354349       -0.689372       -1.470824
```

4-hexene-3-one_14_am1_HEI

Datum	Value
AM1 Energy	-0.124298
AM1 Free Energy (Quasiharmonic)	0.028034
Number of Imaginary Frequencies	0

Frequencies (Top 3 out of 60)

```
1.      39.5357 cm-1
2.      52.9262 cm-1
3.      65.5481 cm-1
```

AM1 Molecular Geometry in Cartesian Coordinates

```
C         2.804738       0.961269      -0.858509
C         2.676999      -0.427148      -0.289073
C         1.347521      -0.601952       0.437411
C         0.222767      -0.720298      -0.384555
C        -1.110509      -0.846927       0.124829
C        -2.009912      -1.778733      -0.642270
O         1.377237      -0.643678       1.700806
H         3.769335       1.078068      -1.410284
H         1.961736       1.169539      -1.561966
H         2.770710       1.721292      -0.040686
H         3.516147      -0.621778       0.427954
H         2.748218      -1.183345      -1.112498
H        -1.123425      -1.067689       1.220977
H        -1.959551      -1.568434      -1.737629
H        -1.686470      -2.835580      -0.472177
H        -3.072211      -1.676825      -0.308940
C        -0.951471       2.039238       0.223284
H        -1.111130       2.808412      -0.563572
H        -1.062152       2.515457       1.221950
H         0.082227       1.619284       0.130080
S        -2.130413       0.758008       0.025272
H         0.354483      -0.689788      -1.470708
```

4-hexene-3-one_16_am1_HEI_reopt

Datum	Value
AM1 Energy	-0.124298

Datum	Value
AM1 Free Energy (Quasiharmonic)	0.028035
Number of Imaginary Frequencies	0

Frequencies (Top 3 out of 60)

```
1.      39.5471 cm-1
2.      53.0407 cm-1
3.      65.5582 cm-1
```

AM1 Molecular Geometry in Cartesian Coordinates

```
C          2.804235       0.961406      -0.858928
C          2.677088      -0.426819      -0.288895
C          1.347615      -0.601928       0.437520
C          0.222911      -0.720180      -0.384532
C         -1.110384      -0.846989       0.124745
C         -2.009672      -1.778757      -0.642528
O          1.377293      -0.643954       1.700915
H          2.769862       1.721772      -0.041440
H          3.768791       1.078387      -1.410736
H          1.961146       1.168995      -1.562483
H          2.748688      -1.183346      -1.111984
H          3.516268      -0.620770       0.428277
H         -1.123352      -1.067883       1.220865
H         -1.959244      -1.568326      -1.737859
H         -1.686176      -2.835605      -0.472537
H         -3.072002      -1.676955      -0.309264
C         -0.951603       2.039187       0.223640
H         -1.062371       2.515183       1.222402
H         -1.111301       2.808515      -0.563057
H          0.082140       1.619351       0.130389
S         -2.130415       0.757890       0.025291
H          0.354703      -0.689450      -1.470670
```

4-hexene-3-one_17_reopt_am1_HEI_reopt

Datum	Value
AM1 Energy	-0.124298
AM1 Free Energy (Quasiharmonic)	0.028032
Number of Imaginary Frequencies	0

Frequencies (Top 3 out of 60)

```
1.      39.4758 cm-1
2.      52.8730 cm-1
3.      65.5259 cm-1
```

AM1 Molecular Geometry in Cartesian Coordinates

```
C          2.804636        0.961428       -0.858411
C          2.677042       -0.427042       -0.289069
C          1.347552       -0.602020        0.437337
C          0.222794       -0.720237       -0.384619
C         -1.110465       -0.846944        0.124790
C         -2.009869       -1.778709       -0.642352
O          1.377271       -0.643959        1.700673
H          3.769117        1.078293       -1.410376
H          1.961489        1.169757       -1.561679
H          2.770781        1.721365       -0.040503
H          3.516161       -0.621583        0.428017
H          2.748442       -1.183185       -1.112522
H         -1.123346       -1.067777        1.220923
H         -3.072164       -1.676832       -0.309002
H         -1.959526       -1.568344       -1.737699
H         -1.686415       -2.835564       -0.472328
C         -0.951529        2.039246        0.223355
H          0.082180        1.619342        0.130053
H         -1.062158        2.515409        1.222052
H         -1.111279        2.808455       -0.563448
S         -2.130430        0.757977        0.025357
H          0.354467       -0.689622       -1.470773
```

4-hexene-3-one_18_am1_HEI

Datum	Value
AM1 Energy	-0.119728
AM1 Free Energy (Quasiharmonic)	0.032039
Number of Imaginary Frequencies	0

Frequencies (Top 3 out of 60)

```
1.      43.1140 cm-1
2.      44.5036 cm-1
```

```
3.      55.8289 cm-1
```

AM1 Molecular Geometry in Cartesian Coordinates

```
C          3.056573       -1.283073       -0.612178
C          2.945288        0.161174       -0.201350
C          1.592079        0.453858        0.438073
C          0.508145        0.511939       -0.443988
C         -0.841974        0.728693       -0.004494
C         -1.642881        1.680952       -0.853512
O          1.571657        0.659997        1.685163
H          2.974895       -1.948829        0.280681
H          2.234127       -1.547973       -1.320519
H          4.037222       -1.479454       -1.111174
H          3.076815        0.821296       -1.097178
H          3.758132        0.411231        0.528955
H         -0.899557        1.012048        1.076305
H         -1.608459        1.380528       -1.928129
H         -2.711378        1.709756       -0.526404
H         -1.218739        2.711502       -0.763609
C         -3.258492       -0.791232        0.511500
H         -3.647247       -1.814032        0.713504
H         -3.261546       -0.208536        1.459656
H         -3.942484       -0.285848       -0.207246
S         -1.637286       -0.948006       -0.129862
H          0.679115        0.352568       -1.512674
```

4-hexene-3-one_19_am1_HEI

Datum	Value
AM1 Energy	-0.119728
AM1 Free Energy (Quasiharmonic)	0.032041
Number of Imaginary Frequencies	0

Frequencies (Top 3 out of 60)

```
1.      43.1394 cm-1
2.      44.5840 cm-1
3.      55.8456 cm-1
```

AM1 Molecular Geometry in Cartesian Coordinates

```
C          -3.056136       -1.283195       -0.612356
C          -2.945293        0.161018       -0.201292
C          -1.592118        0.453929        0.438094
C          -0.508193        0.511986       -0.443988
C           0.841921        0.728826       -0.004524
C           1.642902        1.680829       -0.853749
O          -1.571710        0.660231        1.685152
H          -2.974432       -1.949058        0.280420
H          -4.036643       -1.479767       -1.111554
H          -2.233486       -1.547769       -1.320581
H          -3.076995        0.821248       -1.097013
H          -3.758190        0.410715        0.529073
H           0.899524        1.012383        1.076219
H           1.608485        1.380157       -1.928297
H           1.218832        2.711432       -0.764096
H           2.711395        1.709634       -0.526622
C           3.258502       -0.791237        0.511360
H           3.261812       -0.208888        1.459728
H           3.942193       -0.285507       -0.207430
H           3.647425       -1.814073        0.712863
S           1.637089       -0.947988       -0.129534
H          -0.679174        0.352526       -1.512660
```

4-hexene-3-one_1_am1_HEI

Datum	Value
AM1 Energy	-0.123899
AM1 Free Energy (Quasiharmonic)	0.02811
Number of Imaginary Frequencies	0

Frequencies (Top 3 out of 60)

```
1.      37.2648 cm-1
2.      38.3638 cm-1
3.      75.3311 cm-1
```

AM1 Molecular Geometry in Cartesian Coordinates

```
C          -3.150182        0.660649        0.821215
C          -1.735441        0.148307        0.844878
C          -1.342970       -0.566305       -0.437950
C          -0.003896       -0.969452       -0.565571
C           1.015349       -0.783954        0.412598
```

```
C        2.142954    -1.778742     0.357349
O       -2.231356    -0.764507    -1.314926
H       -3.410081     1.154274     1.788922
H       -3.279801     1.398901    -0.006404
H       -3.862257    -0.180294     0.639379
H       -1.606709    -0.564879     1.700777
H       -1.021485     0.999872     1.004482
H        0.634065    -0.667409     1.457102
H        2.543732    -1.866338    -0.680891
H        2.977524    -1.474581     1.035654
H        1.774696    -2.785200     0.677952
C        1.101595     1.849190    -0.872932
H        0.699751     2.756035    -0.370039
H        0.251113     1.252718    -1.291373
H        1.773533     2.163378    -1.700995
S        1.995911     0.876541     0.275995
H        0.277733    -1.453244    -1.508595
```

4-hexene-3-one_1_am1

Datum	Value
AM1 Energy	-0.064717
AM1 Free Energy (Quasiharmonic)	0.052613
Number of Imaginary Frequencies	0

Frequencies (Top 3 out of 45)

```
1.      31.6560 cm-1
2.      64.4819 cm-1
3.     109.8221 cm-1
```

AM1 Molecular Geometry in Cartesian Coordinates

```
C       -1.113796     2.802617     0.000000
C       -1.301171     1.308733     0.000000
C        0.000000     0.554699     0.000000
C       -0.142966    -0.910186     0.000000
C        0.912846    -1.734961     0.000000
C        0.811270    -3.205489     0.000000
O        1.101037     1.121045     0.000000
H       -2.104884     3.315987     0.000000
H       -0.539979     3.123760     0.902900
H       -0.539979     3.123760    -0.902900
H       -1.887197     0.993360    -0.904710
```

```
H        -1.887197      0.993360      0.904710
H        -1.176331     -1.291226      0.000000
H         1.939066     -1.321994      0.000000
H         1.320751     -3.621653      0.906036
H        -0.250400     -3.554531      0.000000
H         1.320751     -3.621653     -0.906036
```

4-hexene-3-one_2_am1_HEI

Datum	Value
AM1 Energy	-0.123899
AM1 Free Energy (Quasiharmonic)	0.02811
Number of Imaginary Frequencies	0

Frequencies (Top 3 out of 60)

```
1.     37.2091 cm-1
2.     38.3677 cm-1
3.     75.3292 cm-1
```

AM1 Molecular Geometry in Cartesian Coordinates

```
C         3.150310      0.660385      0.821400
C         1.735505      0.148214      0.844898
C         1.342978     -0.566025     -0.438121
C         0.003917     -0.969197     -0.565759
C        -1.015262     -0.783969      0.412537
C        -2.142751     -1.778889      0.357205
O         2.231317     -0.763933     -1.315211
H         3.410208      1.153848      1.789189
H         3.862298     -0.180619      0.639512
H         3.280070      1.398730     -0.006114
H         1.021653      0.999843      1.004684
H         1.606646     -0.565161      1.700616
H        -0.633870     -0.667615      1.457028
H        -2.543568     -1.866391     -0.681027
H        -1.774353     -2.785346      0.677647
H        -2.977328     -1.474932      1.035593
C        -1.102064      1.849016     -0.873029
H        -0.699889      2.755831     -0.370346
H        -1.774394      2.163257     -1.700753
H        -0.251842      1.252476     -1.291889
S        -1.995888      0.876444      0.276352
H        -0.277758     -1.452786     -1.508873
```

4-hexene-3-one_2_am1

Datum	Value
AM1 Energy	-0.062711
AM1 Free Energy (Quasiharmonic)	0.054608
Number of Imaginary Frequencies	0

Frequencies (Top 3 out of 45)

```
1.      27.6098 cm-1
2.      67.8338 cm-1
3.     107.3319 cm-1
```

AM1 Molecular Geometry in Cartesian Coordinates

```
C          2.707784       -0.778347        0.000001
C          1.202035       -0.819763        0.000000
C          0.582582        0.550568       -0.000001
C         -0.889243        0.628796        0.000000
C         -1.711633       -0.425923        0.000000
C         -3.182988       -0.312566        0.000001
O          1.266036        1.582784       -0.000002
H          3.121812       -1.814846        0.000002
H          3.083174       -0.238496       -0.902896
H          3.083173       -0.238495        0.902898
H          0.835180       -1.372650        0.905910
H          0.835181       -1.372651       -0.905909
H         -1.284007        1.660806        0.000000
H         -1.324228       -1.460077       -0.000000
H         -3.603915       -0.817447        0.906196
H         -3.521963        0.752437        0.000001
H         -3.603916       -0.817447       -0.906194
```

4-hexene-3-one_3_am1_HEI

Datum	Value
AM1 Energy	-0.119434
AM1 Free Energy (Quasiharmonic)	0.03207

Datum	Value
Number of Imaginary Frequencies	0

Frequencies (Top 3 out of 60)

```
1.      34.7006 cm-1
2.      51.4062 cm-1
3.      67.2224 cm-1
```

AM1 Molecular Geometry in Cartesian Coordinates

```
C        -3.150935      -1.327289       0.419939
C        -1.792646      -0.703961       0.595311
C        -1.617009       0.569813      -0.215157
C        -0.335212       1.143434      -0.241070
C         0.811732       0.639206       0.444224
C         1.849541       1.677641       0.781368
O        -2.624566       1.056003      -0.802919
H        -3.253276      -2.242094       1.052902
H        -3.949961      -0.599633       0.701743
H        -3.310176      -1.608804      -0.648752
H        -0.993481      -1.428189       0.283927
H        -1.626578      -0.459872       1.677447
H         0.579623       0.017497       1.345381
H         1.436386       2.396439       1.531512
H         2.764244       1.202500       1.213575
H         2.144036       2.252100      -0.129404
C         2.934506      -1.341530       0.096381
H         2.645461      -1.649657       1.126121
H         3.810434      -0.658547       0.173803
H         3.236928      -2.244028      -0.479830
S         1.594623      -0.587489      -0.739983
H        -0.200944       2.030212      -0.871321
```

4-hexene-3-one_3_am1

Datum	Value
AM1 Energy	-0.064537
AM1 Free Energy (Quasiharmonic)	0.053255
Number of Imaginary Frequencies	0

Frequencies (Top 3 out of 45)

```
1.      45.3104 cm-1
2.      71.1392 cm-1
3.     112.8596 cm-1
```

AM1 Molecular Geometry in Cartesian Coordinates

```
C          2.327082       -1.121168       -0.458703
C          1.892331       -0.081596        0.542740
C          0.605872        0.582091        0.127667
C         -0.600216       -0.254514        0.215355
C         -1.818583        0.206116       -0.098978
C         -3.050370       -0.599624       -0.021451
O          0.580832        1.761701       -0.248690
H          3.271145       -1.608936       -0.114852
H          1.544640       -1.909233       -0.579589
H          2.514116       -0.653559       -1.455664
H          2.691412        0.699832        0.648970
H          1.741705       -0.556063        1.548531
H         -0.441578       -1.289298        0.557942
H         -1.945647        1.250349       -0.441015
H         -3.533802       -0.652822       -1.029989
H         -2.850888       -1.641569        0.330134
H         -3.774460       -0.120136        0.685276
```

4-hexene-3-one_4_am1_HEI

Datum	Value
AM1 Energy	-0.119434
AM1 Free Energy (Quasiharmonic)	0.032075
Number of Imaginary Frequencies	0

Frequencies (Top 3 out of 60)

```
1.      34.7078 cm-1
2.      51.5933 cm-1
3.      67.4713 cm-1
```

AM1 Molecular Geometry in Cartesian Coordinates

```
C          3.151343      -1.326967       0.420168
C          1.792770      -0.704127       0.595107
C          1.617051       0.569680      -0.215294
C          0.335231       1.143252      -0.241157
C         -0.811692       0.639041       0.444249
C         -1.849397       1.677515       0.781598
O          2.624569       1.055908      -0.803094
H          3.253646      -2.241986       1.052839
H          3.311176      -1.607989      -0.648580
H          3.950016      -0.599197       0.702697
H          1.626182      -0.460278       1.677223
H          0.994005      -1.428600       0.283297
H         -0.579477       0.017266       1.345343
H         -1.436086       2.396266       1.531711
H         -2.144010       2.252029      -0.129108
H         -2.764059       1.202395       1.213936
C         -2.934929      -1.341176       0.096326
H         -2.646075      -1.649293       1.126129
H         -3.237476      -2.243655      -0.479862
H         -3.810733      -0.658005       0.173606
S         -1.594802      -0.587473      -0.739942
H          0.200915       2.030036      -0.871394
```

4-hexene-3-one_4_am1

Datum	Value
AM1 Energy	-0.062134
AM1 Free Energy (Quasiharmonic)	0.055589
Number of Imaginary Frequencies	0

Frequencies (Top 3 out of 45)

```
1.      21.7747 cm-1
2.      85.3458 cm-1
3.     120.8433 cm-1
```

AM1 Molecular Geometry in Cartesian Coordinates

```
C          1.720980      -1.497425      -0.454134
C          1.427579      -0.502929       0.641251
C          0.756022       0.730194       0.096272
C         -0.711053       0.739901      -0.031865
C         -1.499637      -0.333795       0.086990
```

```
C          -2.967285       -0.287191       -0.059541
O           1.420933        1.725167       -0.222695
H           2.243213       -2.388320       -0.028864
H           0.777985       -1.840371       -0.944878
H           2.378133       -1.039365       -1.232801
H           2.390075       -0.197275        1.133953
H           0.779720       -0.970349        1.427645
H          -1.134069        1.735893       -0.256072
H          -1.083390       -1.334647        0.299202
H          -3.455456       -0.655277        0.878243
H          -3.336018        0.747363       -0.266812
H          -3.287303       -0.951524       -0.901898
```

4-hexene-3-one_5_am1_HEI

Datum	Value
AM1 Energy	-0.123442
AM1 Free Energy (Quasiharmonic)	0.028927
Number of Imaginary Frequencies	0

Frequencies (Top 3 out of 60)

```
1.      28.7070 cm-1
2.      42.3874 cm-1
3.      83.1903 cm-1
```

AM1 Molecular Geometry in Cartesian Coordinates

```
C           2.562674        0.772825       -1.311191
C           1.828821       -0.444515       -0.813440
C           1.437654       -0.301186        0.650252
C           0.225515        0.327588        0.969932
C          -0.697648        0.893625        0.042412
C          -1.545399        2.010414        0.589627
O           2.239235       -0.774745        1.506806
H           2.863912        0.642027       -2.379317
H           1.916230        1.680544       -1.235224
H           3.482742        0.946383       -0.702106
H           2.491134       -1.344084       -0.918664
H           0.911036       -0.625174       -1.430980
H          -0.244721        1.180476       -0.938835
H          -2.348570        2.293439       -0.134134
H          -2.024953        1.709369        1.551720
H          -0.907880        2.909435        0.781119
```

```
C          -1.586870       -1.897251       -0.020710
H          -1.412594       -2.578216       -0.882344
H          -0.646514       -1.814524        0.581911
H          -2.394737       -2.320132        0.615352
S          -2.056868       -0.313872       -0.602407
H          -0.037563        0.371370        2.034286
```

4-hexene-3-one_5_am1

Datum	Value
AM1 Energy	-0.064717
AM1 Free Energy (Quasiharmonic)	0.052613
Number of Imaginary Frequencies	0

Frequencies (Top 3 out of 45)

```
  1.      31.6514 cm-1
  2.      64.5198 cm-1
  3.     109.8337 cm-1
```

AM1 Molecular Geometry in Cartesian Coordinates

```
C           3.012646       -0.138433       -0.000000
C           1.661649       -0.802966        0.000008
C           0.524344        0.180980       -0.000004
C          -0.813733       -0.432110       -0.000011
C          -1.937853        0.296826        0.000008
C          -3.294768       -0.278980        0.000001
O           0.700461        1.406547       -0.000005
H           3.821287       -0.907786        0.000034
H           3.129003        0.508732       -0.902920
H           3.128985        0.508792        0.902879
H           1.554737       -1.459805        0.904729
H           1.554740       -1.459835       -0.904691
H          -0.836766       -1.533248       -0.000033
H          -1.882309        1.401626        0.000030
H          -3.854389        0.066860       -0.906026
H          -3.278319       -1.396433       -0.000024
H          -3.854382        0.066821        0.906047
```

4-hexene-3-one_6_am1_HEI

Datum	Value
AM1 Energy	-0.123442
AM1 Free Energy (Quasiharmonic)	0.028927
Number of Imaginary Frequencies	0

Frequencies (Top 3 out of 60)

```
1.      28.7066 cm-1
2.      42.3871 cm-1
3.      83.1905 cm-1
```

AM1 Molecular Geometry in Cartesian Coordinates

```
C         -2.562672        0.772822       -1.311195
C         -1.828821       -0.444517       -0.813439
C         -1.437654       -0.301183        0.650253
C         -0.225514        0.327591        0.969932
C          0.697650        0.893624        0.042410
C          1.545400        2.010415        0.589621
O         -2.239236       -0.774738        1.506809
H         -2.863909        0.642020       -2.379321
H         -3.482740        0.946383       -0.702112
H         -1.916227        1.680541       -1.235230
H         -0.911035       -0.625180       -1.430977
H         -2.491134       -1.344086       -0.918659
H          0.244722        1.180473       -0.938837
H          2.024954        1.709373        1.551716
H          2.348572        2.293437       -0.134140
H          0.907881        2.909436        0.781111
C          1.586865       -1.897253       -0.020709
H          1.412583       -2.578215       -0.882343
H          0.646511       -1.814523        0.581915
H          2.394733       -2.320137        0.615350
S          2.056868       -0.313874       -0.602404
H          0.037564        0.371376        2.034286
```

4-hexene-3-one_7_am1_HEI

Datum	Value
AM1 Energy	-0.119092
AM1 Free Energy (Quasiharmonic)	0.032972

Datum	Value
Number of Imaginary Frequencies	0

Frequencies (Top 3 out of 60)

```
1.      38.9491 cm-1
2.      52.8933 cm-1
3.      71.6777 cm-1
```

AM1 Molecular Geometry in Cartesian Coordinates

```
C          -2.387497       -1.312068        1.194473
C          -1.855940       -1.056307       -0.191237
C          -1.715679        0.432885       -0.471069
C          -0.519594        1.077739       -0.122770
C           0.599519        0.473536        0.528734
C           1.452708        1.428063        1.322858
O          -2.703249        1.011613       -1.009212
H          -2.510809       -2.408201        1.374150
H          -1.689589       -0.904990        1.965726
H          -3.380524       -0.817823        1.326070
H          -2.562217       -1.494063       -0.945009
H          -0.861638       -1.555804       -0.327631
H           0.347913       -0.424533        1.147404
H           1.772774        2.291208        0.691353
H           0.869942        1.824956        2.190546
H           2.365689        0.915981        1.714691
C           2.990350       -1.079520       -0.113757
H           2.642766       -1.727748        0.721598
H           3.756093       -0.378767        0.289091
H           3.465677       -1.716523       -0.892407
S           1.653340       -0.225931       -0.853502
H          -0.426728        2.132333       -0.409257
```

4-hexene-3-one_8_am1_HEI

Datum	Value
AM1 Energy	-0.119092
AM1 Free Energy (Quasiharmonic)	0.032972
Number of Imaginary Frequencies	0

Frequencies (Top 3 out of 60)

```
1.      38.9492 cm-1
2.      52.8932 cm-1
3.      71.6780 cm-1
```

AM1 Molecular Geometry in Cartesian Coordinates

```
C         2.387497      -1.312069       1.194473
C         1.855938      -1.056308      -0.191236
C         1.715679       0.432885      -0.471068
C         0.519594       1.077740      -0.122770
C        -0.599519       0.473537       0.528734
C        -1.452709       1.428065       1.322857
O         2.703249       1.011612      -1.009212
H         2.510809      -2.408202       1.374149
H         3.380524      -0.817823       1.326069
H         1.689591      -0.904990       1.965727
H         0.861637      -1.555803      -0.327629
H         2.562215      -1.494064      -0.945010
H        -0.347914      -0.424532       1.147405
H        -0.869944       1.824957       2.190546
H        -1.772772       2.291211       0.691351
H        -2.365691       0.915985       1.714687
C        -2.990346      -1.079524      -0.113756
H        -3.756091      -0.378774       0.289095
H        -3.465674      -1.716528      -0.892406
H        -2.642759      -1.727753       0.721598
S        -1.653341      -0.225930      -0.853501
H         0.426729       2.132333      -0.409257
```

4-hexene-3-one_9_am1_HEI

Datum	Value
AM1 Energy	-0.119434
AM1 Free Energy (Quasiharmonic)	0.03207
Number of Imaginary Frequencies	0

Frequencies (Top 3 out of 60)

```
1.      34.7033 cm-1
2.      51.4319 cm-1
3.      67.2477 cm-1
```

AM1 Molecular Geometry in Cartesian Coordinates

```
C          3.151045      -1.327185       0.420061
C          1.792678      -0.703983       0.595267
C          1.617001       0.569772      -0.215222
C          0.335208       1.143406      -0.241065
C         -0.811719       0.639160       0.444251
C         -1.849490       1.677593       0.781518
O          2.624521       1.055929      -0.803075
H          3.253387      -2.241992       1.053022
H          3.310450      -1.608664      -0.648616
H          3.949972      -0.599464       0.701981
H          1.626453      -0.459917       1.677386
H          0.993614      -1.428283       0.283798
H         -0.579580       0.017382       1.345353
H         -1.436285       2.396327       1.531697
H         -2.144011       2.252127      -0.129199
H         -2.764185       1.202441       1.213730
C         -2.934544      -1.341522       0.096354
H         -3.237038      -2.243939      -0.479949
H         -3.810442      -0.658516       0.173922
H         -2.645436      -1.649787       1.126035
S         -1.594691      -0.587425      -0.740015
H          0.200920       2.030195      -0.871299
```

5_3methyl2cyclopentene1one_1_am1_HEI

Datum	Value
AM1 Energy	-0.104149
AM1 Free Energy (Quasiharmonic)	0.030617
Number of Imaginary Frequencies	0

Frequencies (Top 3 out of 54)

```
  1.      51.7338 cm-1
  2.      81.3257 cm-1
  3.     112.9466 cm-1
```

AM1 Molecular Geometry in Cartesian Coordinates

```
C          1.682115       0.341868       1.118383
C          0.337659       1.065151       1.118203
```

```
C          -0.346901        0.701063       -0.209634
C           0.627092       -0.037593       -0.985165
C           1.805259       -0.301433       -0.268387
H           2.528136        1.048583        1.291211
H           1.722398       -0.446777        1.907181
H           0.486764        2.172633        1.180629
H          -0.291746        0.755836        1.987553
O           2.843227       -0.924572       -0.607307
H           0.455275       -0.339060       -2.011986
C          -1.404572       -1.993717        0.081843
H          -1.436659       -2.493979        1.074360
H          -0.363166       -2.044788       -0.325674
H          -2.096939       -2.521306       -0.609902
S          -1.898965       -0.319818        0.235951
C          -1.008268        1.868614       -0.889439
H          -0.256914        2.676735       -1.071895
H          -1.432331        1.554339       -1.873142
H          -1.831492        2.287721       -0.259913
```

5_3-methyl-2-cyclopentene-1-one_1_am1

Datum	Value
AM1 Energy	-0.049065
AM1 Free Energy (Quasiharmonic)	0.051502
Number of Imaginary Frequencies	0

Frequencies (Top 3 out of 39)

```
    1.      67.7079 cm-1
    2.     126.1479 cm-1
    3.     167.7430 cm-1
```

AM1 Molecular Geometry in Cartesian Coordinates

```
C           0.597357        1.263721        0.000000
C          -0.933821        1.201964       -0.000000
C          -1.278810       -0.277908       -0.000000
C           0.000349       -1.027390       -0.000000
C           1.048216       -0.174040       -0.000000
H           0.983934        1.795014       -0.905010
H           0.983934        1.795014        0.905010
H          -1.365843        1.694914       -0.904963
H          -1.365843        1.694914        0.904963
O          -2.414029       -0.750246        0.000000
```

```
H          0.024054      -2.117969      -0.000000
C          2.477681      -0.504080       0.000000
H          2.640047      -1.610181      -0.000006
H          2.973062      -0.071662      -0.906320
H          2.973059      -0.071672       0.906326
```

5_3methyl2cyclopentene1one_2_am1_HEI

Datum	Value
AM1 Energy	-0.104149
AM1 Free Energy (Quasiharmonic)	0.030616
Number of Imaginary Frequencies	0

Frequencies (Top 3 out of 54)

```
1.      51.7329 cm-1
2.      81.3425 cm-1
3.     112.9533 cm-1
```

AM1 Molecular Geometry in Cartesian Coordinates

```
C         -1.682130       0.342000      -1.118343
C         -0.337632       1.065210      -1.118169
C          0.346887       0.701131       0.209680
C         -0.627111      -0.037504       0.985210
C         -1.805258      -0.301390       0.268389
H         -2.528118       1.048785      -1.291069
H         -1.722507      -0.446579      -1.907201
H         -0.486659       2.172698      -1.180631
H          0.291774       0.755802      -1.987490
O         -2.843195      -0.924596       0.607268
H         -0.455308      -0.338987       2.012028
C          1.404398      -1.993774      -0.081840
H          2.096761      -2.521474       0.609826
H          0.363026      -2.044679       0.325788
H          1.436294      -2.494019      -1.074372
S          1.898956      -0.319935      -0.236005
C          1.008445       1.868584       0.889434
H          0.257062       2.676546       1.072494
H          1.433084       1.554124       1.872830
H          1.831270       2.287971       0.259576
```

5_3methyl2cyclopentene1one_3_am1_HEI

Datum	Value
AM1 Energy	-0.099761
AM1 Free Energy (Quasiharmonic)	0.034794
Number of Imaginary Frequencies	0

Frequencies (Top 3 out of 54)

```
1.      64.7178 cm-1
2.      81.7464 cm-1
3.      85.3463 cm-1
```

AM1 Molecular Geometry in Cartesian Coordinates

```
C         -1.680197        0.228123       -1.250399
C         -0.267864        0.797580       -1.145837
C          0.221231        0.473822        0.275654
C         -0.916538       -0.080804        0.988082
C         -2.031977       -0.271565        0.157107
H         -2.411009        1.004159       -1.580092
H         -1.724458       -0.621821       -1.972430
H         -0.276782        1.905098       -1.307569
H          0.408966        0.347393       -1.912080
O         -3.174710       -0.726752        0.415972
H         -0.882287       -0.336411        2.040364
C          2.888995       -0.436764       -0.539094
H          3.460229        0.274220        0.099381
H          2.692467        0.059067       -1.516181
H          3.512672       -1.341907       -0.712485
S          1.404851       -0.956167        0.230449
C          0.925318        1.628384        0.934932
H          0.229422        2.499201        1.020898
H          1.259806        1.343445        1.961193
H          1.817237        1.947590        0.341364
```

6_3pentene2one_1_am1_HEI

Datum	Value
AM1 Energy	-0.114108
AM1 Free Energy (Quasiharmonic)	0.010621

Datum	Value
Number of Imaginary Frequencies	0

Frequencies (Top 3 out of 51)

```
1.      35.7916 cm-1
2.      61.1649 cm-1
3.      77.7044 cm-1
```

AM1 Molecular Geometry in Cartesian Coordinates

```
C          2.941365      -0.083746      -0.770233
C          1.754826       0.058388       0.161067
C          0.605982       0.609324      -0.415621
C         -0.615114       0.815119       0.304057
O          1.902087      -0.311220       1.362376
C         -1.372614       2.065486      -0.054689
H          3.817300      -0.511632      -0.227996
H          2.678059      -0.757000      -1.619902
H          3.223729       0.913445      -1.182746
H          0.626325       0.879913      -1.475979
H         -0.473961       0.717060       1.408935
H         -2.392915       2.062415       0.402438
H         -0.818965       2.960232       0.323812
H         -1.479570       2.161794      -1.161925
S         -1.957313      -0.491558      -0.045274
C         -1.060917      -1.988663      -0.208659
H          0.034788      -1.766465      -0.241011
H         -1.272803      -2.659601       0.652352
H         -1.362818      -2.500922      -1.148128
```

6_3pentene2one_2_reopt2_am1_HEI

Datum	Value
AM1 Energy	-0.114108
AM1 Free Energy (Quasiharmonic)	0.010618
Number of Imaginary Frequencies	0

Frequencies (Top 3 out of 51)

```
1.      35.8520 cm-1
2.      60.7911 cm-1
3.      77.6917 cm-1
```

AM1 Molecular Geometry in Cartesian Coordinates

```
C          -2.941415       -0.083677        0.770112
C          -1.754754        0.058472       -0.161039
C          -0.605917        0.609398        0.415657
C           0.615210        0.815139       -0.304020
O          -1.901941       -0.311132       -1.362375
C           1.372749        2.065472        0.054765
H          -3.816984       -0.512371        0.227939
H          -2.677952       -0.756213        1.620288
H          -3.224458        0.913614        1.181935
H          -0.626261        0.880086        1.475990
H           0.474093        0.717090       -1.408902
H           2.393099        2.062305       -0.402235
H           0.819208        2.960236       -0.323843
H           1.479574        2.161815        1.162008
S           1.957294       -0.491726        0.045187
C           1.060599       -1.988649        0.208786
H           1.362509       -2.500970        1.148214
H          -0.035054       -1.766211        0.241287
H           1.272211       -2.659640       -0.652249
```

6_3pentene2one_3_am1_HEI

Datum	Value
AM1 Energy	-0.114344
AM1 Free Energy (Quasiharmonic)	0.01104
Number of Imaginary Frequencies	0

Frequencies (Top 3 out of 51)

```
1.      38.5784 cm-1
2.      57.3498 cm-1
3.      96.4266 cm-1
```

AM1 Molecular Geometry in Cartesian Coordinates

```
C          1.893935      -0.383355       1.293979
C          1.805245       0.138643      -0.121861
C          0.595118       0.717514      -0.535257
C         -0.563888       0.870688       0.278924
O          2.830875       0.025665      -0.853655
C         -1.468349       2.011662      -0.099877
H          1.061436      -1.103144       1.483683
H          2.868747      -0.900016       1.460634
H          1.808421       0.461325       2.018183
H          0.537889       1.061312      -1.574788
H         -0.354989       0.868971       1.377130
H         -0.967540       2.984173       0.134215
H         -2.430743       1.963994       0.466479
H         -1.698035       1.989452      -1.192129
S         -1.814489      -0.604831       0.217110
C         -0.952584      -1.905356      -0.577517
H          0.047029      -1.538509      -0.924664
H         -0.806388      -2.751711       0.128944
H         -1.537863      -2.262652      -1.452547
```

6_3pentene2one_4_am1_HEI

Datum	Value
AM1 Energy	-0.109674
AM1 Free Energy (Quasiharmonic)	0.014792
Number of Imaginary Frequencies	0

Frequencies (Top 3 out of 51)

```
1.      44.2824 cm-1
2.      58.7359 cm-1
3.      69.7666 cm-1
```

AM1 Molecular Geometry in Cartesian Coordinates

```
C          3.206808      -0.221637      -0.697569
C          1.981979      -0.142166       0.190533
C          0.865720       0.473550      -0.387031
C         -0.399913       0.578757       0.281806
O          2.075957      -0.600349       1.365578
C         -1.093386       1.907428       0.131532
H          3.489699       0.796666      -1.054350
H          4.067765      -0.662102      -0.141584
```

```
H         2.985492      -0.859928      -1.585324
H         0.940514       0.863302      -1.406390
H        -0.342410       0.276945       1.357483
H        -0.506946       2.698832       0.660519
H        -2.120623       1.876832       0.571285
H        -1.176164       2.193134      -0.944457
S        -1.459952      -0.708250      -0.546358
C        -2.992249      -0.730450       0.299628
H        -3.524239      -1.667179       0.021118
H        -2.843246      -0.712543       1.402368
H        -3.632019       0.137935       0.023045
```

6_3pentene2one_5_reopt_am1_HEI

Datum	Value
AM1 Energy	-0.111805
AM1 Free Energy (Quasiharmonic)	0.013072
Number of Imaginary Frequencies	0

Frequencies (Top 3 out of 51)

```
1.      32.5276 cm-1
2.      52.3406 cm-1
3.      72.8465 cm-1
```

AM1 Molecular Geometry in Cartesian Coordinates

```
C        -2.835950      -0.882046      -0.413971
C        -1.686750       0.000993       0.035997
C        -0.614855       0.065187      -0.860135
C         0.602398       0.799322      -0.694725
O        -1.811440       0.589104       1.148709
C         0.589968       1.923274       0.300744
H        -3.659011      -0.867459       0.338744
H        -3.233864      -0.521847      -1.391682
H        -2.480367      -1.931765      -0.540487
H        -0.687386      -0.537346      -1.774705
H         1.026668       1.142296      -1.675586
H         1.597633       2.398687       0.378874
H        -0.151052       2.698544      -0.016787
H         0.279415       1.548884       1.307267
S         2.086735      -0.302174      -0.207011
C         1.392752      -1.600992       0.742773
H         1.714907      -1.517452       1.803701
```

```
H          0.276516      -1.546699       0.691270
H          1.734918      -2.578309       0.337802
```

6_3pentene2one_6_am1_HEI

Datum	Value
AM1 Energy	-0.114344
AM1 Free Energy (Quasiharmonic)	0.011039
Number of Imaginary Frequencies	0

Frequencies (Top 3 out of 51)

```
1.      38.5423 cm-1
2.      57.3249 cm-1
3.      96.4231 cm-1
```

AM1 Molecular Geometry in Cartesian Coordinates

```
C         -1.894029      -0.383239      -1.294027
C         -1.805269       0.138563       0.121881
C         -0.595138       0.717409       0.535301
C          0.563848       0.870703      -0.278888
O         -2.830865       0.025479       0.853708
C          1.468268       2.011682       0.100002
H         -2.868773      -0.900049      -1.460631
H         -1.808773       0.461573      -2.018109
H         -1.061432      -1.102851      -1.483963
H         -0.537888       1.061087       1.574871
H          0.354925       0.869091      -1.377091
H          0.967413       2.984191      -0.133998
H          2.430656       1.964103      -0.466371
H          1.697970       1.989385       1.192250
S          1.814497      -0.604772      -0.217256
C          0.952798      -1.905319       0.577559
H          0.806817      -2.751822      -0.128770
H          1.538090      -2.262354       1.452686
H         -0.046909      -1.538612       0.924581
```

6_3pentene2one_7_am1_HEI

Datum	Value
AM1 Energy	-0.109425
AM1 Free Energy (Quasiharmonic)	0.016477
Number of Imaginary Frequencies	0

Frequencies (Top 3 out of 51)

```
1.      30.0031 cm-1
2.      48.7938 cm-1
3.      92.8518 cm-1
```

AM1 Molecular Geometry in Cartesian Coordinates

```
C        -1.720138       0.304379       1.276996
C        -1.731501      -0.200440      -0.145528
C        -0.638010       0.062074      -0.985022
C         0.547190       0.810502      -0.737677
O        -2.747671      -0.862027      -0.511621
C         0.492550       2.036124       0.130184
H        -1.823492       1.415583       1.287402
H        -2.569880      -0.136151       1.851836
H        -0.760876       0.022730       1.773684
H        -0.692364      -0.415327      -1.975305
H         1.070485       1.053399      -1.699438
H        -0.383263       2.669463      -0.156763
H         1.423475       2.643229       0.004735
H         0.401302       1.771953       1.210897
S         1.988510      -0.184748       0.090952
C         1.356813      -1.800765       0.327898
H         1.430449      -2.076246       1.402683
H         0.283885      -1.838081       0.009083
H         1.944060      -2.529608      -0.272178
```

6_cis_3pentene2one_1_am1

Datum	Value
AM1 Energy	-0.053035
AM1 Free Energy (Quasiharmonic)	0.037881
Number of Imaginary Frequencies	0

Frequencies (Top 3 out of 36)

```
   1.      32.5662 cm-1
   2.      81.6430 cm-1
   3.      96.2466 cm-1
```

AM1 Molecular Geometry in Cartesian Coordinates

```
 C          -2.292372       -0.249725        0.000011
 C          -0.850720        0.154604        0.000000
 C           0.105898       -0.959401       -0.000006
 C           1.440118       -0.822285       -0.000006
 O          -0.511504        1.346630       -0.000013
 C           2.154847        0.466124        0.000009
 H          -2.955035        0.649150        0.000241
 H          -2.511234       -0.863231       -0.907615
 H          -2.511128       -0.863624        0.907395
 H          -0.349053       -1.963776       -0.000017
 H           2.076733       -1.725275       -0.000018
 H           3.261311        0.318237       -0.000139
 H           1.866792        1.069878       -0.899455
 H           1.867015        1.069703        0.899660
```

6_cis_3pentene2one_2_am1

Datum	Value
AM1 Energy	-0.050569
AM1 Free Energy (Quasiharmonic)	0.039896
Number of Imaginary Frequencies	0

Frequencies (Top 3 out of 36)

```
   1.      27.9741 cm-1
   2.      76.1613 cm-1
   3.      96.0563 cm-1
```

AM1 Molecular Geometry in Cartesian Coordinates

```
 C          -1.401905        1.167645        0.373562
 C          -0.937914       -0.204114       -0.004796
 C           0.246449       -0.694267        0.728355
 C           1.511936       -0.371950        0.453848
```

```
O          -1.531088       -0.890664       -0.842966
C           1.952142        0.518506       -0.637156
H          -1.654270        1.190471        1.461701
H          -0.585075        1.905702        0.181592
H          -2.304689        1.458196       -0.216136
H          -0.000440       -1.388536        1.549784
H           2.328933       -0.793806        1.066071
H           2.560490        1.361820       -0.223467
H           2.592012       -0.051663       -1.357561
H           1.087494        0.948207       -1.201138
```

---

methane_thiolate_am1

Datum	Value
AM1 Energy	-0.027104
AM1 Free Energy (Quasiharmonic)	-0.011189
Number of Imaginary Frequencies	0

Frequencies (Top 3 out of 9)

```
1.     818.8846 cm-1
2.     947.8763 cm-1
3.     947.9178 cm-1
```

AM1 Molecular Geometry in Cartesian Coordinates

```
C          0.000006       -1.059692       -0.000000
H         -1.039520       -1.466710        0.000000
H          0.519698       -1.466703        0.900290
H          0.519698       -1.466703       -0.900290
S          0.000006        0.672392       -0.000000
```

---

methane_thiolate_DFT

Datum	Value
M06-2X/def2tzvpp-IEFPCM(water) Energy	-438.210295
M06-2X/def2tzvpp-IEFPCM(water) Free Energy (Quasiharmonic)	-438.193051
Number of Imaginary Frequencies	0

Frequencies (Top 3 out of 9)

```
1.     715.6670 cm-1
2.     943.2388 cm-1
3.     947.1466 cm-1
```

M06-2X/def2tzvpp-IEFPCM(water) Molecular Geometry in Cartesian Coordinates

```
C          0.000000      -0.000000      -1.124868
H          0.000000       1.015083      -1.523719
H         -0.879088      -0.507542      -1.523719
H          0.879088      -0.507542      -1.523719
S         -0.000000       0.000000       0.707523
```

Created using ESIgen v0.0.5

ESIgen is scientific software, funded by public research grants and published as:

```
J Rodriguez-Guerra, P Gomez-Orellana, JD Marechal.
J. Chem. Inf. Model., 2018, 58 (3), pp 561564.
DOI: 10.1021/acs.jcim.7b00714.
```

If you make use of ESIgen in scientific publications, please cite us in the main text! References only mentioned in SI documents are not indexed by citation engines.

1-pentene-3-one_protonation_TS_10_reopt

Datum	Value
M06-2X/def2tzvpp-IEFPCM(water) Energy	-1108.136987
M06-2X/def2tzvpp-IEFPCM(water) Free Energy (Quasiharmonic)	-1107.999495
Number of Imaginary Frequencies	1

Frequencies (Top 3 out of 60)

```
  1.    -944.2083 cm-1
  2.      29.0860 cm-1
  3.      38.4127 cm-1
```

M06-2X/def2tzvpp-IEFPCM(water) Molecular Geometry in Cartesian Coordinates

```
C          0.192136        1.583794       -0.330020
C          0.036459        0.197623       -0.508830
C          0.566970       -0.836216        0.425294
O         -0.071000        2.434107       -1.214840
H         -0.123245       -0.124806       -1.534874
H          0.670620       -0.452860        1.440419
H         -0.095860       -1.703684        0.483703
C          3.231965       -0.131533       -0.015906
H          2.785943        0.662698       -0.613627
H          4.191877       -0.407151       -0.446417
H          3.385296        0.216455        1.004170
S          2.176333       -1.589213       -0.052071
C          0.552747        2.098462        1.057612
H         -0.201017        1.785757        1.785185
```

```
H          1.512326       1.712671       1.402341
H          0.592551       3.184633       1.034662
H         -1.475746       0.227360       0.022057
S         -2.909721      -0.054931       0.431581
C         -2.934976      -1.641212      -0.458291
H         -3.889937      -1.771793      -0.961500
H         -2.144770      -1.644438      -1.209065
H         -2.777634      -2.476898       0.220367
```

1-pentene-3-one_protonation_TS_11_reopt

Datum	Value
M06-2X/def2tzvpp-IEFPCM(water) Energy	-1108.136984
M06-2X/def2tzvpp-IEFPCM(water) Free Energy (Quasiharmonic)	-1107.999708
Number of Imaginary Frequencies	1

Frequencies (Top 3 out of 60)

```
1.   -1018.9924 cm-1
2.      40.3976 cm-1
3.      47.7463 cm-1
```

M06-2X/def2tzvpp-IEFPCM(water) Molecular Geometry in Cartesian Coordinates

```
C          0.524569       1.449650      -0.126588
C         -0.004097       0.323727      -0.790195
C         -1.049117      -0.550619      -0.177316
O          1.179713       2.357211      -0.689948
H          0.006646       0.371012      -1.875639
H         -1.085944      -1.524240      -0.671884
H         -0.855568      -0.730670       0.881621
C         -3.698580      -1.134646       0.451967
H         -4.742696      -0.830556       0.435909
H         -3.389555      -1.291473       1.484052
H         -3.590381      -2.063445      -0.105821
S         -2.722979       0.179000      -0.310253
C          0.375139       1.505621       1.387555
H         -0.676571       1.535075       1.678631
H          0.813150       0.613878       1.844465
H          0.880821       2.389350       1.769028
H          1.188509      -0.692634      -0.462688
S          2.275838      -1.672246      -0.044577
C          3.501480      -0.345641       0.177024
```

```
H          4.385917      -0.757189       0.657983
H          3.790135       0.082663      -0.780233
H          3.095719       0.443932       0.806757
```

1-pentene-3-one_protonation_TS_12_reopt

Datum	Value
M06-2X/def2tzvpp-IEFPCM(water) Energy	-1108.139805
M06-2X/def2tzvpp-IEFPCM(water) Free Energy (Quasiharmonic)	-1108.003151
Number of Imaginary Frequencies	1

Frequencies (Top 3 out of 60)

```
1.    -999.8773 cm-1
2.      17.4858 cm-1
3.      32.8648 cm-1
```

M06-2X/def2tzvpp-IEFPCM(water) Molecular Geometry in Cartesian Coordinates

```
C          0.074315       1.694339       0.046949
C         -0.085462       0.333682       0.367873
C         -0.744466      -0.591117      -0.597515
O         -0.100858       2.176299      -1.095819
H         -0.091206       0.060265       1.416662
H         -0.472306      -0.319650      -1.617831
H         -0.440195      -1.626941      -0.433926
C         -2.881585      -1.135349       1.079163
H         -2.479127      -0.422661       1.796244
H         -2.432573      -2.113995       1.243955
H         -3.957864      -1.209588       1.216448
S         -2.579956      -0.577580      -0.607276
C          0.631714       2.610300       1.120292
H          0.723011       2.120834       2.088096
H          1.617401       2.961401       0.806793
H         -0.010127       3.486601       1.218641
H          1.405665       0.027183      -0.031110
S          2.766423      -0.565343      -0.429299
C          2.466014      -2.070931       0.546802
H          1.635854      -1.893874       1.231456
H          2.214381      -2.912326      -0.095395
H          3.347302      -2.326424       1.130329
```

1-pentene-3-one_protonation_TS_1

Datum	Value
M06-2X/def2tzvpp-IEFPCM(water) Energy	-1108.140321
M06-2X/def2tzvpp-IEFPCM(water) Free Energy (Quasiharmonic)	-1108.002562
Number of Imaginary Frequencies	1

Frequencies (Top 3 out of 60)

```
  1.   -1068.1219 cm-1
  2.      34.3722 cm-1
  3.      45.7723 cm-1
```

M06-2X/def2tzvpp-IEFPCM(water) Molecular Geometry in Cartesian Coordinates

```
C        -0.034372        1.441537        0.121225
C         0.112922        0.257499       -0.627875
C         0.664816       -0.976073        0.003489
O         0.031273        1.522155        1.370481
H         0.267474        0.369374       -1.696476
H         0.540978       -0.925660        1.084914
H         0.166346       -1.882889       -0.349538
C         3.148146        0.208842        0.355027
H         2.969209        0.259333        1.427309
H         2.712139        1.082755       -0.126179
H         4.218954        0.186550        0.165823
S         2.437810       -1.293676       -0.338301
C        -0.413434        2.700459       -0.638981
H        -0.453620        2.549913       -1.716152
H        -1.389928        3.043400       -0.290975
H         0.307146        3.487929       -0.413311
H        -1.412914       -0.105210       -0.726410
S        -2.835920       -0.676871       -0.595620
C        -2.540075       -1.138555        1.141254
H        -1.768990       -0.489578        1.553850
H        -3.453656       -1.013696        1.718265
H        -2.211571       -2.172956        1.222941
```

1-pentene-3-one_protonation_TS_2_reopt

Datum	Value
M06-2X/def2tzvpp-IEFPCM(water) Energy	-1108.13699
M06-2X/def2tzvpp-IEFPCM(water) Free Energy (Quasiharmonic)	-1107.999273
Number of Imaginary Frequencies	1

Frequencies (Top 3 out of 60)

```
1.   -1004.4300 cm-1
2.      27.0457 cm-1
3.      44.1047 cm-1
```

M06-2X/def2tzvpp-IEFPCM(water) Molecular Geometry in Cartesian Coordinates

```
C          0.161010        1.283707       -0.273744
C         -0.150573       -0.032645       -0.667526
C         -0.915569       -0.994107        0.179832
O          0.517594        2.187577       -1.068094
H         -0.231705       -0.185694       -1.740936
H         -0.583866       -2.024380        0.018893
H         -0.791829       -0.786442        1.242757
C         -3.204041        0.593501        0.254553
H         -4.244828        0.718413       -0.035142
H         -2.589880        1.302374       -0.299482
H         -3.106570        0.777689        1.323159
S         -2.719509       -1.090781       -0.156165
C          0.167708        1.608771        1.215583
H          0.688439        0.838301        1.787957
H          0.662860        2.565463        1.365592
H         -0.848559        1.675430        1.608321
H          1.261141       -0.696877       -0.342495
S          2.563542       -1.366549        0.076313
C          3.469339        0.211514        0.069202
H          3.762249        0.493248       -0.939858
H          2.845804        1.000205        0.486630
H          4.364217        0.114480        0.679581
```

1-pentene-3-one_protonation_TS_3_reopt

Datum	Value
M06-2X/def2tzvpp-IEFPCM(water) Energy	-1108.13646
M06-2X/def2tzvpp-IEFPCM(water) Free Energy (Quasiharmonic)	-1108.000059

Datum	Value
Number of Imaginary Frequencies	1

Frequencies (Top 3 out of 60)

```
1.    -990.0203 cm-1
2.       3.6263 cm-1
3.      35.7754 cm-1
```

M06-2X/def2tzvpp-IEFPCM(water) Molecular Geometry in Cartesian Coordinates

```
C         0.193836       1.352855      -0.397617
C        -0.141741      -0.002648      -0.581041
C        -0.847871      -0.841253       0.431836
O         0.641340       2.083206      -1.314973
H        -0.285661      -0.290730      -1.619369
H        -0.805728      -1.892684       0.142926
H        -0.396817      -0.771352       1.422430
C        -3.256412      -0.661778      -0.934976
H        -3.091070      -1.678481      -1.288813
H        -2.772527       0.042710      -1.608613
H        -4.324856      -0.460667      -0.912460
S        -2.625287      -0.461030       0.739764
C         0.050070       1.994256       0.974394
H        -0.064561       1.283844       1.790258
H         0.911980       2.633198       1.165040
H        -0.837244       2.631142       0.959696
H         1.316197      -0.665351      -0.580799
S         2.697909      -1.264987      -0.448582
C         3.095858      -0.179689       0.952103
H         3.914579       0.492508       0.705741
H         2.213592       0.417352       1.186707
H         3.367003      -0.761334       1.829942
```

1-pentene-3-one_protonation_TS_4_reopt

Datum	Value
M06-2X/def2tzvpp-IEFPCM(water) Energy	-1108.139826
M06-2X/def2tzvpp-IEFPCM(water) Free Energy (Quasiharmonic)	-1108.002525
Number of Imaginary Frequencies	1

Frequencies (Top 3 out of 60)

```
1.   -1008.3677 cm-1
2.      21.7681 cm-1
3.      29.8230 cm-1
```

M06-2X/def2tzvpp-IEFPCM(water) Molecular Geometry in Cartesian Coordinates

```
C          0.275518        1.304277       -0.214150
C         -0.170308        0.130591        0.425843
C         -1.005414       -0.849434       -0.327806
O          0.273159        1.471841       -1.455153
H         -0.283526        0.162205        1.503363
H         -0.632227       -0.953637       -1.347337
H         -0.983247       -1.836424        0.139231
C         -3.281067       -0.352440        1.176692
H         -2.736142        0.423971        1.709909
H         -3.112364       -1.314722        1.658250
H         -4.343908       -0.123171        1.201210
S         -2.772924       -0.410341       -0.550638
C          0.851099        2.418441        0.642726
H          0.957965        2.135023        1.688610
H          1.820667        2.722837        0.247228
H          0.187246        3.283419        0.580355
H          1.140396       -0.719416        0.229979
S          2.399135       -1.546816       -0.038081
C          3.457420       -0.115313        0.330703
H          3.674544        0.457808       -0.568607
H          2.948565        0.533751        1.042917
H          4.393898       -0.448609        0.771566
```

1-pentene-3-one_protonation_TS_5_reopt

Datum	Value
M06-2X/def2tzvpp-IEFPCM(water) Energy	-1108.140587
M06-2X/def2tzvpp-IEFPCM(water) Free Energy (Quasiharmonic)	-1108.003371
Number of Imaginary Frequencies	1

Frequencies (Top 3 out of 60)

```
1.   -1002.6925 cm-1
2.      29.2582 cm-1
3.      38.3473 cm-1
```

M06-2X/def2tzvpp-IEFPCM(water) Molecular Geometry in Cartesian Coordinates

```
C     -0.227127     1.536592     0.005008
C     -0.060425     0.212857     0.451832
C     -0.575726    -0.929890    -0.356194
O     -0.562512     1.862542    -1.158167
H      0.054275     0.053942     1.518769
H     -0.629620    -0.642027    -1.405853
H      0.066179    -1.810851    -0.283160
C     -3.194886    -0.049376    -0.052057
H     -3.181202     0.286198    -1.086865
H     -2.796201     0.736975     0.586375
H     -4.216092    -0.276629     0.245504
S     -2.225372    -1.554677     0.140547
C      0.161855     2.650317     0.960350
H      0.435680     2.284801     1.948274
H      1.005722     3.200086     0.537956
H     -0.668590     3.351434     1.054216
H      1.427712     0.186910    -0.051338
S      2.851258    -0.162950    -0.511497
C      2.925799    -1.603518     0.597128
H      2.779260    -2.531077     0.047420
H      3.888531    -1.642940     1.101176
H      2.143339    -1.516999     1.351663
```

1-pentene-3-one_protonation_TS_7_reopt

Datum	Value
M06-2X/def2tzvpp-IEFPCM(water) Energy	-1108.137601
M06-2X/def2tzvpp-IEFPCM(water) Free Energy (Quasiharmonic)	-1107.998458
Number of Imaginary Frequencies	1

Frequencies (Top 3 out of 60)

```
1.   -1153.5855 cm-1
2.      52.2678 cm-1
3.      63.0314 cm-1
```

M06-2X/def2tzvpp-IEFPCM(water) Molecular Geometry in Cartesian Coordinates

```
C        -0.812307      1.577666      0.056336
C         0.054042      0.955759     -0.872641
C         1.534929      1.106739     -0.702448
O        -0.471349      2.021690      1.175479
H        -0.307675      0.923088     -1.896226
H         2.013305      1.453415     -1.618726
H         1.751836      1.828379      0.086139
C         1.782289     -0.710492      1.357479
H         2.095176      0.063457      2.056030
H         2.139564     -1.678215      1.703400
H         0.695844     -0.730792      1.288772
S         2.476646     -0.410536     -0.276384
C        -2.277264      1.674016     -0.338526
H        -2.553112      0.903826     -1.059422
H        -2.906127      1.587871      0.546574
H        -2.463480      2.650159     -0.791900
H        -0.463365     -0.526162     -0.724302
S        -1.128464     -1.924327     -0.628128
C        -2.101135     -1.467947      0.839100
H        -3.131285     -1.235496      0.575884
H        -2.096096     -2.281902      1.560782
H        -1.658025     -0.587799      1.305566
```

1-pentene-3-one_protonation_TS_8

Datum	Value
M06-2X/def2tzvpp-IEFPCM(water) Energy	-1108.140072
M06-2X/def2tzvpp-IEFPCM(water) Free Energy (Quasiharmonic)	-1108.002551
Number of Imaginary Frequencies	1

Frequencies (Top 3 out of 60)

```
1.    -995.8042 cm-1
2.      27.4723 cm-1
3.      49.9270 cm-1
```

M06-2X/def2tzvpp-IEFPCM(water) Molecular Geometry in Cartesian Coordinates

```
C          -0.525852       1.384653        0.170331
C          -0.001606       0.356638       -0.640138
C           1.079894      -0.525720       -0.107719
O          -0.381137       1.448457        1.412663
H          -0.039038       0.500576       -1.714306
H           0.928531      -0.692300        0.959191
H           1.097090      -1.496197       -0.610028
C           3.758092      -1.084187        0.370429
H           3.508533      -1.252970        1.416573
H           4.796818      -0.769718        0.298770
H           3.627277      -2.009204       -0.188836
S           2.730004       0.226512       -0.323688
C          -1.371311       2.457956       -0.494507
H          -1.610242       2.225185       -1.531039
H          -2.293074       2.603224        0.069225
H          -0.820208       3.400496       -0.467494
H          -1.113616      -0.721672       -0.385480
S          -2.155282      -1.788503       -0.048771
C          -3.451749      -0.523528        0.105855
H          -3.539546      -0.170366        1.131411
H          -3.204753       0.322764       -0.534460
H          -4.409036      -0.930510       -0.210994
```

1-pentene-3-one_protonation_TS_9_reopt

Datum	Value
M06-2X/def2tzvpp-IEFPCM(water) Energy	-1108.133934
M06-2X/def2tzvpp-IEFPCM(water) Free Energy (Quasiharmonic)	-1107.996042
Number of Imaginary Frequencies	1

Frequencies (Top 3 out of 60)

```
1.   -1094.7929 cm-1
2.      25.9374 cm-1
3.      39.2682 cm-1
```

M06-2X/def2tzvpp-IEFPCM(water) Molecular Geometry in Cartesian Coordinates

```
C           1.454989      -1.333301        0.304246
C           0.244849      -0.779136        0.774128
C          -1.082005      -1.430155        0.514139
O           2.582157      -1.027160        0.751607
```

```
H          0.331378      -0.245900       1.717355
H         -0.982316      -2.360124      -0.043474
H         -1.588935      -1.675340       1.449134
C         -2.502659       0.928958       0.648998
H         -3.229542       1.609089       0.210294
H         -1.551161       1.447256       0.759520
H         -2.865755       0.593720       1.619775
S         -2.308617      -0.471576      -0.465631
C          1.398502      -2.265784      -0.897590
H          0.656126      -1.939959      -1.627792
H          2.378714      -2.300465      -1.367352
H          1.129455      -3.277091      -0.584231
H          0.441590       0.462186      -0.184394
S          0.742544       1.794414      -0.921597
C          1.360069       2.611239       0.580526
H          0.832739       2.222322       1.451849
H          2.426520       2.443314       0.716821
H          1.178624       3.681943       0.518610
```

3-methyl-2-butenal_protonation_TS_10_reopt

Datum	Value
M06-2X/def2tzvpp-IEFPCM(water) Energy	-1147.436455
M06-2X/def2tzvpp-IEFPCM(water) Free Energy (Quasiharmonic)	-1147.26978
Number of Imaginary Frequencies	1

Frequencies (Top 3 out of 69)

```
1.   -1175.9246 cm-1
2.      52.6194 cm-1
3.      69.6868 cm-1
```

M06-2X/def2tzvpp-IEFPCM(water) Molecular Geometry in Cartesian Coordinates

```
C          0.631021       1.879727      -0.936571
C          0.251488       0.524988      -1.028848
C          1.061690      -0.638488      -0.486732
O          1.445552       2.426495      -0.173372
H         -0.270800       0.298995      -1.956939
C          0.783854      -1.878892      -1.338412
C          2.567984      -0.370466      -0.483054
C          0.645088       0.383897       2.131422
H         -0.035305       1.113496       1.698497
```

```
H          1.656026       0.783683       2.133116
H          0.340162       0.163299       3.153032
S          0.550030      -1.162087       1.217626
H          0.038376       2.534331      -1.609387
H         -1.084699       0.698966      -0.300513
S         -2.583622       0.682877       0.217404
C         -2.899504      -0.920968      -0.574631
H         -2.419143      -1.724957      -0.018695
H         -3.970219      -1.108726      -0.617146
H         -2.509101      -0.921917      -1.592568
H          1.296678      -2.754502      -0.936603
H         -0.285121      -2.088984      -1.383377
H          1.145368      -1.706235      -2.353801
H          3.109678      -1.267419      -0.179157
H          2.887421      -0.093037      -1.491032
H          2.824030       0.449629       0.182035
```

3-methyl-2-butenal_protonation_TS_1_reopt

Datum	Value
M06-2X/def2tzvpp-IEFPCM(water) Energy	-1147.441623
M06-2X/def2tzvpp-IEFPCM(water) Free Energy (Quasiharmonic)	-1147.27582
Number of Imaginary Frequencies	1

Frequencies (Top 3 out of 69)

```
1.   -1093.8698 cm-1
2.      39.6484 cm-1
3.      59.0033 cm-1
```

M06-2X/def2tzvpp-IEFPCM(water) Molecular Geometry in Cartesian Coordinates

```
C          0.137943       1.834641       0.130609
C         -0.010766       0.522834      -0.346179
C          0.721243      -0.660137       0.226618
O         -0.271253       2.876863      -0.421246
H         -0.258055       0.440248      -1.404183
C          0.017318      -1.956686      -0.177835
C          0.842830      -0.613783       1.748944
C          3.161747       0.757901      -0.194060
H          2.602207       1.542896      -0.698793
H          4.173464       0.727950      -0.593430
H          3.213507       0.971530       0.872042
```

```
S          2.427271       -0.853056       -0.511794
H          0.582924        1.932618        1.139048
H         -1.462148        0.387888        0.198025
S         -2.974866        0.154358        0.494718
C         -3.232493       -0.874287       -0.982285
H         -3.259846       -1.932212       -0.728242
H         -4.168122       -0.606108       -1.467398
H         -2.416728       -0.709112       -1.686171
H          0.591786       -2.829878        0.136810
H         -0.963341       -1.995944        0.298789
H         -0.123156       -2.001224       -1.258659
H         -0.148798       -0.476832        2.187862
H          1.261920       -1.549908        2.118093
H          1.479012        0.199450        2.094525
```

3-methyl-2-butenal_protonation_TS_2_reopt

Datum	Value
M06-2X/def2tzvpp-IEFPCM(water) Energy	-1147.438764
M06-2X/def2tzvpp-IEFPCM(water) Free Energy (Quasiharmonic)	-1147.272518
Number of Imaginary Frequencies	1

Frequencies (Top 3 out of 69)

```
1.   -1078.3241 cm-1
2.      48.2433 cm-1
3.      63.7634 cm-1
```

M06-2X/def2tzvpp-IEFPCM(water) Molecular Geometry in Cartesian Coordinates

```
C         -0.226682        1.661898       -0.749050
C          0.221069        1.021339        0.411730
C          1.450980        0.136991        0.500770
O         -1.019884        2.630967       -0.806920
H          0.055238        1.584159        1.331930
C          1.340491       -0.780109        1.719980
C          2.724969        0.982383        0.632250
C          0.208873       -1.765001       -1.226700
H         -0.069376       -2.312671       -0.329080
H          0.372044       -2.470321       -2.039270
H         -0.593378       -1.083132       -1.495800
S          1.758181       -0.876449       -1.003460
H          0.106578        1.207279       -1.701640
```

```
H        -1.070460        0.165557        0.710130
S        -2.403119       -0.520075        1.062360
C        -3.033119       -0.446256       -0.639940
H        -3.019338       -1.428836       -1.107820
H        -4.051820       -0.064777       -0.648960
H        -2.406990        0.229885       -1.222860
H         2.250292       -1.366548        1.850630
H         1.188510       -0.171780        2.615700
H         0.490462       -1.456161        1.633610
H         2.649618        1.601673        1.527770
H         3.614710        0.354804        0.723730
H         2.842508        1.640043       -0.229340
```

3-methyl-2-butenal_protonation_TS_3_reopt

Datum	Value
M06-2X/def2tzvpp-IEFPCM(water) Energy	-1147.437211
M06-2X/def2tzvpp-IEFPCM(water) Free Energy (Quasiharmonic)	-1147.270935
Number of Imaginary Frequencies	1

Frequencies (Top 3 out of 69)

```
1.   -1121.9394 cm-1
2.      30.2048 cm-1
3.      54.1747 cm-1
```

M06-2X/def2tzvpp-IEFPCM(water) Molecular Geometry in Cartesian Coordinates

```
C        -0.247427        1.743800       -0.768360
C         0.014032        0.933240        0.349750
C         1.360381        0.282278        0.591930
O        -1.236726        2.483791       -0.935580
H        -0.520648        1.222210        1.253710
C         1.520621        0.010478        2.087400
C         2.544742        1.122357        0.107910
C         1.023290       -1.110521       -1.876570
H        -0.008570       -0.765660       -1.922440
H         1.103269       -2.062011       -2.398830
H         1.680351       -0.390122       -2.361250
S         1.512809       -1.413642       -0.172150
H         0.466953        1.652039       -1.609220
H        -1.011759       -0.159289        0.063280
S        -2.196010       -1.181068       -0.007040
```

```
C        -3.404569    -0.036537     0.725470
H        -4.109200    -0.587456     1.344620
H        -2.886258     0.687773     1.353330
H        -3.957249     0.503634    -0.040510
H         2.443820    -0.533433     2.294640
H         1.553632     0.960458     2.623820
H         0.678420    -0.570121     2.466130
H         2.492263     2.117017     0.556530
H         3.486892     0.654726     0.397220
H         2.546692     1.244697    -0.974550
```

3-methyl-2-butenal_protonation_TS_4

Datum	Value
M06-2X/def2tzvpp-IEFPCM(water) Energy	-1147.441559
M06-2X/def2tzvpp-IEFPCM(water) Free Energy (Quasiharmonic)	-1147.275817
Number of Imaginary Frequencies	1

Frequencies (Top 3 out of 69)

```
1.   -1025.3236 cm-1
2.      30.8185 cm-1
3.      56.9754 cm-1
```

M06-2X/def2tzvpp-IEFPCM(water) Molecular Geometry in Cartesian Coordinates

```
C        -0.291049     1.589295    -0.365816
C        -0.025977     0.247715    -0.697514
C         0.846128    -0.700445     0.088689
O        -0.197974     2.166915     0.735629
H        -0.037847     0.052570    -1.767421
C         0.694128    -0.564502     1.602126
C         0.546899    -2.144890    -0.319629
C         2.880424     1.282180    -0.158759
H         2.604329     1.604689     0.842733
H         2.289958     1.833096    -0.888103
H         3.937312     1.480369    -0.325238
S         2.642952    -0.489708    -0.360599
H        -0.711331     2.167668    -1.214857
H        -1.435107    -0.305371    -0.293817
S        -2.838197    -0.819074     0.104507
C        -3.633725     0.740207    -0.376552
H        -4.478299     0.553409    -1.035677
```

```
H          -3.980775        1.291153        0.494903
H          -2.908061        1.357035       -0.909788
H          -0.361170       -0.660006        1.867739
H           1.256309       -1.354194        2.102627
H           1.035642        0.404595        1.954294
H           1.255439       -2.835940        0.140910
H          -0.456686       -2.417208        0.011364
H           0.597025       -2.264015       -1.402483
```

3-methyl-2-butenal_protonation_TS_6

Datum	Value
M06-2X/def2tzvpp-IEFPCM(water) Energy	-1147.439336
M06-2X/def2tzvpp-IEFPCM(water) Free Energy (Quasiharmonic)	-1147.273047
Number of Imaginary Frequencies	1

Frequencies (Top 3 out of 69)

```
 1.   -1097.5178 cm-1
 2.      35.3099 cm-1
 3.      65.1184 cm-1
```

M06-2X/def2tzvpp-IEFPCM(water) Molecular Geometry in Cartesian Coordinates

```
C          -0.701520        1.776022       -0.786328
C           0.078401        0.607338       -0.792468
C           1.258349        0.452112        0.158456
O          -0.803602        2.638444        0.111896
H           0.198269        0.157922       -1.774345
C           2.247492        1.613593        0.000403
C           0.823883        0.360713        1.621525
C           1.022807       -2.352875       -0.257120
H           0.263302       -2.201762       -1.021010
H           0.545937       -2.427366        0.718585
H           1.557113       -3.278817       -0.459938
S           2.247496       -1.032510       -0.290981
H          -1.339457        1.888821       -1.686515
H          -1.061986       -0.365211       -0.270660
S          -2.215513       -1.283224        0.206814
C          -3.490373       -0.014211       -0.040984
H          -3.713960        0.512897        0.884264
H          -3.141630        0.709302       -0.778131
H          -4.403484       -0.471564       -0.415121
```

```
H          3.112671        1.489158        0.655707
H          2.595307        1.688224       -1.030449
H          1.738250        2.540042        0.264911
H          1.695490        0.323895        2.276627
H          0.228523        1.241696        1.863935
H          0.208496       -0.519203        1.802752
```

3-methyl-2-butenal_protonation_TS_7_reopt

Datum	Value
M06-2X/def2tzvpp-IEFPCM(water) Energy	-1147.440189
M06-2X/def2tzvpp-IEFPCM(water) Free Energy (Quasiharmonic)	-1147.275746
Number of Imaginary Frequencies	1

Frequencies (Top 3 out of 69)

```
1.   -1059.8766 cm-1
2.      22.1064 cm-1
3.      44.1965 cm-1
```

M06-2X/def2tzvpp-IEFPCM(water) Molecular Geometry in Cartesian Coordinates

```
C          0.515550        1.784650        0.389145
C          0.244806        0.599362       -0.311217
C         -0.898999       -0.321133        0.048926
O          1.298895        2.692993        0.044515
H          0.515701        0.608922       -1.365999
C         -0.906315       -0.703246        1.528724
C         -0.819376       -1.587569       -0.799477
C         -3.780232       -0.587397       -0.133245
H         -4.713740       -0.046935       -0.278443
H         -3.771871       -0.998733        0.874018
H         -3.726847       -1.392961       -0.861985
S         -2.448079        0.612144       -0.369094
H          0.038291        1.872802        1.384261
H          1.490747       -0.183197        0.215504
S          2.788388       -0.972638        0.510589
C          3.639830       -0.331540       -0.963288
H          2.901138       -0.010725       -1.697480
H          4.274652        0.516369       -0.714867
H          4.253560       -1.110870       -1.408839
H          0.024965       -1.221035        1.771925
H         -0.997392        0.167816        2.174371
```

```
H          -1.731689       -1.380172        1.752713
H           0.124805       -2.095476       -0.592055
H          -1.633191       -2.275483       -0.568734
H          -0.856824       -1.345142       -1.861844
```

3-methyl-2-butenal_protonation_TS_8_reopt

Datum	Value
M06-2X/def2tzvpp-IEFPCM(water) Energy	-1147.441318
M06-2X/def2tzvpp-IEFPCM(water) Free Energy (Quasiharmonic)	-1147.277115
Number of Imaginary Frequencies	1

Frequencies (Top 3 out of 69)

```
1.   -1054.1596 cm-1
2.       5.3900 cm-1
3.      46.4428 cm-1
```

M06-2X/def2tzvpp-IEFPCM(water) Molecular Geometry in Cartesian Coordinates

```
C          -0.244041        1.718214        0.076830
C          -0.060428        0.421258       -0.432575
C           0.826787       -0.625051        0.189517
O           0.002966        2.153342        1.218264
H          -0.150120        0.361449       -1.514687
C           0.486467       -0.915995        1.651991
C           0.730849       -1.917611       -0.619227
C           2.861834        0.542873       -1.425693
H           2.666742       -0.218477       -2.179063
H           3.903102        0.849871       -1.498863
H           2.219692        1.403922       -1.601059
S           2.619096       -0.095587        0.238985
H          -0.731375        2.408707       -0.642738
H          -1.475967       -0.173507       -0.069267
S          -2.890196       -0.721365        0.170437
C          -3.619614        0.530716       -0.924762
H          -4.449033        1.033567       -0.433308
H          -2.856558        1.274356       -1.163292
H          -3.976449        0.086837       -1.851339
H           0.502828       -0.002361        2.240436
H          -0.513036       -1.351127        1.711352
H           1.192870       -1.634542        2.073494
H          -0.309828       -2.250393       -0.647170
```

```
H          1.060974       -1.767422       -1.647990
H          1.338913       -2.702799       -0.169858
```

3-methyl-2-butenal_protonation_TS_9

Datum	Value
M06-2X/def2tzvpp-IEFPCM(water) Energy	-1147.439548
M06-2X/def2tzvpp-IEFPCM(water) Free Energy (Quasiharmonic)	-1147.273434
Number of Imaginary Frequencies	1

Frequencies (Top 3 out of 69)

```
1.   -1027.5393 cm-1
2.      48.4677 cm-1
3.      57.2637 cm-1
```

M06-2X/def2tzvpp-IEFPCM(water) Molecular Geometry in Cartesian Coordinates

```
C          0.745222        1.708499       -0.735187
C          0.220939        1.146326        0.435186
C         -1.134227        0.464168        0.494539
O          1.722336        2.487070       -0.817035
H          0.496105        1.653444        1.359598
C         -2.280891        1.425948        0.174898
C         -1.332626       -0.119068        1.893205
C         -2.811763       -1.466268       -0.785851
H         -3.182914       -1.699252        0.210307
H         -2.823223       -2.377526       -1.380436
H         -3.459596       -0.731457       -1.257652
S         -1.094515       -0.911460       -0.740191
H          0.278866        1.362855       -1.678434
H          1.334887        0.048380        0.702521
S          2.459691       -0.967734        0.869283
C          2.565500       -1.241649       -0.920868
H          3.564741       -1.019500       -1.288657
H          2.311869       -2.268423       -1.175188
H          1.852873       -0.578522       -1.416410
H         -3.258253        0.956426        0.302078
H         -2.226824        2.276056        0.858240
H         -2.200361        1.801014       -0.845622
H         -1.362985        0.694007        2.623073
H         -2.270306       -0.670152        1.970499
H         -0.509314       -0.784537        2.151369
```

4-methyl-3-pentene-2-one_protonation_TS_10

Datum	Value
M06-2X/def2tzvpp-IEFPCM(water) Energy	-1186.755256
M06-2X/def2tzvpp-IEFPCM(water) Free Energy (Quasiharmonic)	-1186.562928
Number of Imaginary Frequencies	1

Frequencies (Top 3 out of 78)

```
1.   -1007.9453 cm-1
2.      40.3662 cm-1
3.      58.0755 cm-1
```

M06-2X/def2tzvpp-IEFPCM(water) Molecular Geometry in Cartesian Coordinates

```
C         0.676669       1.751809      -0.282253
C        -0.083019       0.593433      -0.521588
C        -1.420954       0.360898       0.162037
O         0.510334       2.558061       0.665056
H         0.035409       0.145844      -1.502637
C        -1.271655       0.132065       1.667235
C        -2.387650       1.526020      -0.080285
C        -1.058524      -2.401759      -0.426873
H        -1.488119      -3.282211      -0.900851
H        -0.856885      -2.622105       0.618778
H        -0.125288      -2.141937      -0.922524
S        -2.270663      -1.081104      -0.604062
C         1.900669       1.954721      -1.160530
H         1.890455       1.336401      -2.056564
H         1.979787       3.005252      -1.440534
H         2.787821       1.697107      -0.575018
H         1.000345      -0.258283       0.313165
S         2.105141      -1.092117       0.973237
C         2.918608      -1.399501      -0.622843
H         2.221543      -1.165131      -1.428881
H         3.803857      -0.777877      -0.742734
H         3.207015      -2.444801      -0.706705
H        -2.249305       0.039397       2.142480
H        -0.694136      -0.768132       1.877459
H        -0.741896       0.982392       2.098337
H        -3.358124       1.339494       0.385730
```

```
H          -1.955244        2.427373        0.352366
H          -2.536420        1.688153       -1.148526
```

4-methyl-3-pentene-2-one_protonation_TS_11_reopt

Datum	Value
M06-2X/def2tzvpp-IEFPCM(water) Energy	-1186.756016
M06-2X/def2tzvpp-IEFPCM(water) Free Energy (Quasiharmonic)	-1186.564588
Number of Imaginary Frequencies	1

Frequencies (Top 3 out of 78)

```
1.   -1082.7908 cm-1
2.      26.8571 cm-1
3.      47.7604 cm-1
```

M06-2X/def2tzvpp-IEFPCM(water) Molecular Geometry in Cartesian Coordinates

```
C           0.648202        1.603335       -0.062976
C           0.150390        0.445777       -0.700631
C          -0.939621       -0.433921       -0.138744
O           0.397620        1.984550        1.103399
H           0.192655        0.469002       -1.786353
C          -1.149131       -1.628458       -1.066866
C          -0.620531       -0.935061        1.269189
C          -3.795771       -0.583406        0.294529
H          -3.964500       -1.293918       -0.511488
H          -4.703639       -0.001561        0.442712
H          -3.575622       -1.118901        1.216092
S          -2.487875        0.595859       -0.112205
C           1.672053        2.412061       -0.845977
H           1.784986        2.079349       -1.875985
H           1.390233        3.465481       -0.832293
H           2.638394        2.324602       -0.343127
H           1.461128       -0.463169       -0.649744
S           2.714006       -1.331026       -0.490347
C           3.019712       -0.696541        1.188378
H           2.295355        0.088803        1.401232
H           4.022765       -0.282566        1.265068
H           2.906876       -1.485745        1.928972
H          -0.213330       -2.187056       -1.141082
H          -1.914614       -2.303918       -0.683780
H          -1.439708       -1.301785       -2.066049
```

```
H          -1.440609       -1.533636        1.669463
H           0.267376       -1.570835        1.223840
H          -0.418625       -0.100581        1.934745
```

4-methyl-3-pentene-2-one_protonation_TS_13

Datum	Value
M06-2X/def2tzvpp-IEFPCM(water) Energy	-1186.752871
M06-2X/def2tzvpp-IEFPCM(water) Free Energy (Quasiharmonic)	-1186.561331
Number of Imaginary Frequencies	1

Frequencies (Top 3 out of 78)

```
1.   -1098.5444 cm-1
2.      41.3053 cm-1
3.      47.2732 cm-1
```

M06-2X/def2tzvpp-IEFPCM(water) Molecular Geometry in Cartesian Coordinates

```
C          -1.353209       -1.629216       -0.189830
C          -0.381777       -0.678955       -0.558493
C           0.953020       -0.586731        0.166702
O          -1.373970       -2.281376        0.880175
H          -0.365465       -0.414926       -1.611688
C           0.793894       -0.150330        1.623765
C           1.711331       -1.914659        0.105632
C           3.542665        0.598962       -0.075185
H           4.104206        1.431544       -0.493940
H           4.033569       -0.330509       -0.353596
H           3.531457        0.700187        1.008240
S           1.880110        0.708331       -0.764866
C          -2.558713       -1.759358       -1.105847
H          -2.435266       -1.236403       -2.052177
H          -2.759610       -2.813969       -1.295756
H          -3.429033       -1.345271       -0.590104
H          -1.348020        0.487016       -0.061049
S          -2.248234        1.675395        0.372186
C          -1.038012        2.940598       -0.115447
H          -0.211372        2.986399        0.591141
H          -0.632690        2.717529       -1.101020
H          -1.528947        3.910756       -0.148895
H           1.763964       -0.058142        2.115302
H           0.273025        0.804501        1.685742
```

```
H          0.208242      -0.900555       2.154541
H          2.656702      -1.867742       0.649642
H          1.090730      -2.684486       0.565265
H          1.915054      -2.196395      -0.927942
```

4-methyl-3-pentene-2-one_protonation_TS_1_reopt

Datum	Value
M06-2X/def2tzvpp-IEFPCM(water) Energy	-1186.755951
M06-2X/def2tzvpp-IEFPCM(water) Free Energy (Quasiharmonic)	-1186.564489
Number of Imaginary Frequencies	1

Frequencies (Top 3 out of 78)

```
1.   -1076.6895 cm-1
2.      16.3528 cm-1
3.      58.4288 cm-1
```

M06-2X/def2tzvpp-IEFPCM(water) Molecular Geometry in Cartesian Coordinates

```
C          0.322113       1.401187       0.013155
C         -0.032735       0.161489      -0.561277
C         -0.982502      -0.845667       0.031572
O          0.168823       1.748906       1.206635
H         -0.003225       0.140226      -1.647860
C         -0.768029      -2.209286      -0.630917
C         -0.860390      -1.004695       1.545397
C         -2.876657       1.284232       0.109662
H         -2.220899       1.902287      -0.500443
H         -2.611970       1.409912       1.156937
H         -3.909620       1.589376      -0.045087
S         -2.757876      -0.440909      -0.388386
C          1.015032       2.393194      -0.914262
H          1.408370       1.926111      -1.816129
H          0.291608       3.157001      -1.210813
H          1.822497       2.892525      -0.379894
H          1.364762      -0.554424      -0.332436
S          2.726083      -1.237333      -0.090772
C          3.572945       0.349772       0.184606
H          4.534202       0.165674       0.659188
H          3.741545       0.874423      -0.753591
H          2.974423       0.981768       0.837738
H         -1.531154      -2.923458      -0.315249
```

```
H          -0.796677       -2.126213       -1.717924
H           0.206720       -2.604677       -0.339404
H          -1.473065       -1.843406        1.879811
H           0.182445       -1.205868        1.802592
H          -1.160520       -0.101981        2.068386
```

4-methyl-3-pentene-2-one_protonation_TS_2

Datum	Value
M06-2X/def2tzvpp-IEFPCM(water) Energy	-1186.754312
M06-2X/def2tzvpp-IEFPCM(water) Free Energy (Quasiharmonic)	-1186.561718
Number of Imaginary Frequencies	1

Frequencies (Top 3 out of 78)

```
1.   -1150.2111 cm-1
2.      67.9011 cm-1
3.      69.8893 cm-1
```

M06-2X/def2tzvpp-IEFPCM(water) Molecular Geometry in Cartesian Coordinates

```
C           0.952568        1.543033       -0.452405
C          -0.082801        0.605029       -0.637223
C          -1.251808        0.522792        0.329955
O           1.168465        2.212525        0.585828
H          -0.302315        0.353807       -1.670183
C          -0.819968        0.006610        1.703535
C          -1.952615        1.876852        0.495438
C          -1.686928       -2.099834       -0.702764
H          -1.239173       -2.509676        0.199318
H          -0.911279       -1.950052       -1.451137
H          -2.425390       -2.801037       -1.086660
S          -2.565780       -0.564687       -0.363316
C           1.958417        1.677967       -1.585764
H           1.691354        1.095080       -2.465172
H           2.048493        2.729176       -1.863274
H           2.937186        1.348387       -1.230278
H           0.867509       -0.660678       -0.414344
S           1.836866       -1.841272       -0.154920
C           2.758492       -0.939007        1.127509
H           3.551641       -1.576585        1.512487
H           3.201225       -0.031483        0.722796
H           2.096717       -0.667057        1.947660
```

```
H          -1.663048       -0.006952        2.396107
H          -0.400454       -0.997455        1.633450
H          -0.046023        0.669763        2.092100
H          -2.807566        1.801399        1.171625
H          -1.236112        2.585330        0.908815
H          -2.299999        2.252523       -0.467831
```

4-methyl-3-pentene-2-one_protonation_TS_3_reopt

Datum	Value
M06-2X/def2tzvpp-IEFPCM(water) Energy	-1186.753875
M06-2X/def2tzvpp-IEFPCM(water) Free Energy (Quasiharmonic)	-1186.562041
Number of Imaginary Frequencies	1

Frequencies (Top 3 out of 78)

```
1.   -1090.9089 cm-1
2.      29.4148 cm-1
3.      58.7516 cm-1
```

M06-2X/def2tzvpp-IEFPCM(water) Molecular Geometry in Cartesian Coordinates

```
C           0.897985        1.588577       -0.357969
C          -0.038766        0.550947       -0.515565
C          -1.087081        0.260626        0.543288
O           1.103709        2.253547        0.689192
H          -0.313767        0.329565       -1.541560
C          -0.444482       -0.194374        1.857763
C          -1.986570        1.469132        0.806987
C          -3.058150       -0.524946       -1.363371
H          -2.401876       -0.061954       -2.097895
H          -3.807001        0.192722       -1.037004
H          -3.559829       -1.371315       -1.827622
S          -2.115399       -1.180453        0.026901
C           1.815147        1.863023       -1.539839
H           1.539226        1.305408       -2.433007
H           1.811767        2.930708       -1.763204
H           2.835522        1.589569       -1.259696
H           0.976228       -0.697005       -0.538716
S           1.993495       -1.833009       -0.477921
C           2.924953       -1.008678        0.850503
H           3.980582       -0.947427        0.594870
H           2.531476       -0.000691        0.976702
```

```
H          2.820685      -1.544830       1.791712
H          0.236758       0.585969       2.193904
H         -1.203516      -0.365863       2.623291
H          0.126461      -1.112721       1.712623
H         -2.475791       1.804862      -0.107437
H         -2.752150       1.239560       1.551208
H         -1.362212       2.284605       1.173823
```

4-methyl-3-pentene-2-one_protonation_TS_4_reopt

Datum	Value
M06-2X/def2tzvpp-IEFPCM(water) Energy	-1186.752635
M06-2X/def2tzvpp-IEFPCM(water) Free Energy (Quasiharmonic)	-1186.56083
Number of Imaginary Frequencies	1

Frequencies (Top 3 out of 78)

```
1.    -981.7047 cm-1
2.      31.9115 cm-1
3.      46.8750 cm-1
```

M06-2X/def2tzvpp-IEFPCM(water) Molecular Geometry in Cartesian Coordinates

```
C         -0.101879       1.689222      -0.153751
C         -0.055399       0.294950      -0.346138
C          0.792639      -0.729399       0.359803
O         -0.704587       2.456688      -0.945415
H         -0.334550       0.007287      -1.357969
C          0.603536      -0.803141       1.877502
C          0.450118      -2.102285      -0.224231
C          2.743779      -0.105070      -1.615547
H          3.788973       0.097312      -1.839795
H          2.144649       0.769498      -1.861245
H          2.414106      -0.953679      -2.212490
S          2.637715      -0.455579       0.146689
C          0.483040       2.316051       1.100033
H          1.468073       1.914738       1.334124
H          0.548242       3.392606       0.957840
H         -0.172497       2.120206       1.952294
H         -1.529721       0.016236       0.249859
S         -2.970522      -0.377071       0.526467
C         -3.263615      -0.773876      -1.222671
H         -3.249340       0.126818      -1.833292
```

```
H          -2.497647       -1.457556       -1.586291
H          -4.234863       -1.252161       -1.324269
H          -0.445268       -1.013298        2.095580
H           1.206846       -1.612897        2.293370
H           0.883569        0.117946        2.381125
H           0.647618       -2.135921       -1.296254
H           1.025345       -2.889603        0.262487
H          -0.615244       -2.297354       -0.072256
```

4-methyl-3-pentene-2-one_protonation_TS_5_reopt

Datum	Value
M06-2X/def2tzvpp-IEFPCM(water) Energy	-1186.752721
M06-2X/def2tzvpp-IEFPCM(water) Free Energy (Quasiharmonic)	-1186.561277
Number of Imaginary Frequencies	1

Frequencies (Top 3 out of 78)

```
1.   -1132.6521 cm-1
2.      37.4035 cm-1
3.      49.0152 cm-1
```

M06-2X/def2tzvpp-IEFPCM(water) Molecular Geometry in Cartesian Coordinates

```
C           0.839904        1.808139       -0.285898
C           0.059498        0.648626       -0.446887
C          -1.048802        0.291286        0.531182
O           0.921117        2.498918        0.758437
H          -0.072560        0.311300       -1.468791
C          -0.502278       -0.096890        1.908338
C          -2.050157        1.437172        0.691220
C          -2.790610       -0.653027       -1.518820
H          -3.187599       -1.538687       -2.010299
H          -2.105522       -0.152230       -2.200533
H          -3.614446        0.012380       -1.272148
S          -1.929208       -1.222682       -0.040877
C           1.785013        2.157863       -1.423128
H           1.615459        1.562891       -2.318503
H           1.685348        3.216200       -1.666060
H           2.810816        1.993176       -1.083535
H           1.341832       -0.233692       -0.122802
S           2.562030       -1.159318        0.158029
C           1.641924       -2.697912       -0.141514
```

```
H          0.918034       -2.883957        0.650170
H          1.109775       -2.646879       -1.089762
H          2.342129       -3.529646       -0.178464
H         -1.317912       -0.284983        2.608736
H          0.123741       -0.987532        1.845627
H          0.108722        0.724174        2.281244
H         -2.862048        1.160421        1.366767
H         -1.521528        2.298990        1.099016
H         -2.475291        1.727183       -0.269557
```

4-methyl-3-pentene-2-one_protonation_TS_7_reopt

Datum	Value
M06-2X/def2tzvpp-IEFPCM(water) Energy	-1186.750717
M06-2X/def2tzvpp-IEFPCM(water) Free Energy (Quasiharmonic)	-1186.559093
Number of Imaginary Frequencies	1

Frequencies (Top 3 out of 78)

```
1.   -1159.4093 cm-1
2.      31.3621 cm-1
3.      39.1516 cm-1
```

M06-2X/def2tzvpp-IEFPCM(water) Molecular Geometry in Cartesian Coordinates

```
C         -0.396061        1.735589        0.316451
C          0.103659        0.889009       -0.696309
C          1.405779        0.113699       -0.614419
O         -0.023781        1.780859        1.511111
H         -0.125691        1.230249       -1.702909
C          1.817229       -0.280231       -2.036139
C          2.537379        0.914229        0.030841
C          0.974849       -1.120561        1.962121
H          0.812199       -2.066821        2.476241
H          0.100059       -0.485471        2.056201
H          1.833869       -0.619641        2.403931
S          1.273419       -1.533951        0.234591
C         -1.572531        2.622149       -0.068469
H         -1.843861        2.545909       -1.119349
H         -2.435631        2.340299        0.538451
H         -1.331701        3.659789        0.167501
H         -1.127471       -0.077491       -0.835029
S         -2.376241       -0.988781       -0.950289
```

```
C         -2.383731       -1.304361        0.838801
H         -1.625051       -2.038691        1.103841
H         -2.175261       -0.379451        1.376441
H         -3.361131       -1.673221        1.142371
H          1.973299        0.623809       -2.627379
H          2.742689       -0.857221       -2.035439
H          1.035939       -0.870781       -2.518299
H          3.463819        0.337049        0.041341
H          2.701379        1.826439       -0.548619
H          2.278539        1.204949        1.045721
```

4-methyl-3-pentene-2-one_protonation_TS_8_reopt

Datum	Value
M06-2X/def2tzvpp-IEFPCM(water) Energy	-1186.747652
M06-2X/def2tzvpp-IEFPCM(water) Free Energy (Quasiharmonic)	-1186.55511
Number of Imaginary Frequencies	1

Frequencies (Top 3 out of 78)

```
1.    -930.4322 cm-1
2.      36.6042 cm-1
3.      57.9506 cm-1
```

M06-2X/def2tzvpp-IEFPCM(water) Molecular Geometry in Cartesian Coordinates

```
C         -0.747668        1.800857       -0.701103
C         -0.021201        0.609841       -0.538436
C          1.226671        0.389591        0.296029
O         -1.618168        1.943256       -1.601263
H         -0.097346       -0.032131       -1.411206
C          2.452968        1.106821       -0.276137
C          1.078722        0.743431        1.781602
C          1.643847       -1.939507       -1.357057
H          0.651641       -1.961502       -1.802804
H          2.050751       -2.948988       -1.361232
H          2.299771       -1.299438       -1.943521
S          1.591711       -1.427372        0.369129
C         -0.622499        2.938494        0.301958
H         -1.022090        2.633420        1.271826
H          0.410227        3.249518        0.455863
H         -1.198701        3.786380       -0.060954
H         -1.229566       -0.102460        0.297169
```

```
S          -2.335596       -0.965172        0.858711
C          -2.071763       -2.232760       -0.415157
H          -1.105580       -2.714791       -0.274581
H          -2.857858       -2.981044       -0.346385
H          -2.102334       -1.783854       -1.407107
H           2.268301        2.183035       -0.296153
H           3.341943        0.918669        0.329065
H           2.652280        0.790354       -1.299999
H           1.070867        1.821549        1.924552
H           0.157423        0.328134        2.190406
H           1.923331        0.347205        2.349529
```

4-methyl-3-pentene-2-one_protonation_TS_9_reopt

Datum	Value
M06-2X/def2tzvpp-IEFPCM(water) Energy	-1186.753897
M06-2X/def2tzvpp-IEFPCM(water) Free Energy (Quasiharmonic)	-1186.563242
Number of Imaginary Frequencies	1

Frequencies (Top 3 out of 78)

```
1.   -1089.6460 cm-1
2.      12.8769 cm-1
3.      33.7368 cm-1
```

M06-2X/def2tzvpp-IEFPCM(water) Molecular Geometry in Cartesian Coordinates

```
C           1.207621        1.530293       -0.241002
C           0.198520        0.634326       -0.643652
C          -1.072231        0.456292        0.170408
O           1.279252        2.124830        0.862798
H           0.093548        0.513393       -1.718479
C          -0.774093       -0.101852        1.563096
C          -1.857601        1.763531        0.292843
C          -3.615781       -0.851487        0.079977
H          -4.197007       -1.638342       -0.396373
H          -4.164368        0.084228        0.002345
H          -3.474506       -1.111404        1.126952
S          -2.044273       -0.775758       -0.800521
C           2.369771        1.717363       -1.204276
H           2.199285        1.252958       -2.173803
H           2.565256        2.781725       -1.339102
H           3.263052        1.272091       -0.758667
```

```
H          1.040084       -0.739837       -0.563761
S          1.891704       -1.993090       -0.386235
C          2.654225       -1.335945        1.129774
H          2.370398       -1.928566        1.997083
H          3.738758       -1.333710        1.043971
H          2.308738       -0.313128        1.276107
H         -1.691399       -0.256906        2.133722
H         -0.237735       -1.047838        1.489835
H         -0.150311        0.613386        2.098646
H         -2.757239        1.642585        0.899677
H         -1.216307        2.501844        0.775393
H         -2.145757        2.135320       -0.690841
```

---

methylacrolein_protonation_TS_1_reopt

Datum	Value
M06-2X/def2tzvpp-IEFPCM(water) Energy	-1108.136422
M06-2X/def2tzvpp-IEFPCM(water) Free Energy (Quasiharmonic)	-1107.998439
Number of Imaginary Frequencies	1

Frequencies (Top 3 out of 60)

```
1.    -930.2421 cm-1
2.      24.0723 cm-1
3.      38.8007 cm-1
```

M06-2X/def2tzvpp-IEFPCM(water) Molecular Geometry in Cartesian Coordinates

```
C          0.120017        1.460805        0.217421
C         -0.078499        0.085202        0.417730
C         -0.824682       -0.684596       -0.626272
O         -0.018282        2.107941       -0.843206
C         -0.056996       -0.471197        1.815479
H         -0.741167       -0.176565       -1.586201
H         -0.429565       -1.699353       -0.739027
C         -3.102021        0.743278        0.075542
H         -2.885814        1.389003       -0.773103
H         -2.578425        1.117720        0.954022
H         -4.172643        0.735462        0.267325
S         -2.603473       -0.951146       -0.274883
H          0.510906        1.996083        1.107536
H          1.297310       -0.363856       -0.120332
S          2.655332       -0.891162       -0.587454
```

```
C          3.511631        0.426923        0.322537
H          4.143228        0.015168        1.106315
H          4.124712        1.025787       -0.346926
H          2.763621        1.076498        0.782989
H          0.436547       -1.448589        1.842950
H          0.493332        0.192304        2.485763
H         -1.062218       -0.608736        2.227114
```

---

methylacrolein_protonation_TS_2_reopt_reopt

Datum	Value
M06-2X/def2tzvpp-IEFPCM(water) Energy	-1108.137199
M06-2X/def2tzvpp-IEFPCM(water) Free Energy (Quasiharmonic)	-1107.999759
Number of Imaginary Frequencies	1

Frequencies (Top 3 out of 60)

```
1.    -922.9743 cm-1
2.      14.9769 cm-1
3.      38.7436 cm-1
```

M06-2X/def2tzvpp-IEFPCM(water) Molecular Geometry in Cartesian Coordinates

```
C         -0.107166        1.415810       -0.463421
C          0.117002        0.272627        0.321045
C          0.752084       -0.914999       -0.323892
O         -0.481712        2.542063       -0.073947
C          0.254239        0.415914        1.813086
H          0.335875       -1.851676        0.058149
H          0.591877       -0.896946       -1.404126
C          3.114379        0.506213       -0.620799
H          2.690416        1.300983       -0.009040
H          2.827097        0.657017       -1.660508
H          4.198901        0.528326       -0.541824
S          2.558151       -1.103251       -0.036505
H          0.002658        1.250677       -1.555554
H         -1.346737       -0.192606        0.382836
S         -2.777587       -0.732841        0.476178
C         -3.049274       -0.390066       -1.286579
H         -3.868151        0.312132       -1.425102
H         -2.139856        0.051329       -1.701144
H         -3.271507       -1.303654       -1.833058
H         -0.370553        1.234283        2.170126
```

```
H          -0.059004       -0.501043        2.322925
H           1.286084        0.619141        2.116492
```

---

methylacrolein_protonation_TS_3_reopt

Datum	Value
M06-2X/def2tzvpp-IEFPCM(water) Energy	-1108.133857
M06-2X/def2tzvpp-IEFPCM(water) Free Energy (Quasiharmonic)	-1107.996697
Number of Imaginary Frequencies	1

Frequencies (Top 3 out of 60)

```
1.    -926.3897 cm-1
2.       3.7999 cm-1
3.      47.5535 cm-1
```

M06-2X/def2tzvpp-IEFPCM(water) Molecular Geometry in Cartesian Coordinates

```
C          -0.606630        1.673269       -0.444912
C           0.036536        0.578645        0.154004
C           0.841129       -0.332855       -0.713729
O          -1.196259        2.614497        0.127012
C           0.370185        0.628567        1.620725
H           0.723727       -1.381641       -0.422613
H           0.519464       -0.247072       -1.753104
C           3.200786       -1.128842        0.587739
H           2.807695       -0.803251        1.547645
H           4.287615       -1.090528        0.615766
H           2.888236       -2.152903        0.391537
S           2.658778       -0.043391       -0.749255
H          -0.639371        1.633881       -1.553671
H          -1.229979       -0.270795        0.358879
S          -2.431389       -1.193912        0.584127
C          -3.053262       -0.832477       -1.083526
H          -3.094412       -1.734127       -1.690169
H          -2.376305       -0.120803       -1.562228
H          -4.045847       -0.389979       -1.041289
H           0.345874       -0.373329        2.063356
H           1.366626        1.043580        1.803094
H          -0.353937        1.250000        2.146949
```

---

methylacrolein_protonation_TS_4_reopt

Datum	Value
M06-2X/def2tzvpp-IEFPCM(water) Energy	-1108.131732
M06-2X/def2tzvpp-IEFPCM(water) Free Energy (Quasiharmonic)	-1107.993454
Number of Imaginary Frequencies	1

Frequencies (Top 3 out of 60)

```
1.   -1041.7608 cm-1
2.      37.3803 cm-1
3.      48.8750 cm-1
```

M06-2X/def2tzvpp-IEFPCM(water) Molecular Geometry in Cartesian Coordinates

```
C          0.513586       1.888465       0.464100
C         -0.006162       0.589246       0.594844
C         -0.640309      -0.043146      -0.602799
O          0.687265       2.548642      -0.581494
C         -0.438342       0.107954       1.955353
H         -0.273493       0.442351      -1.505862
H         -0.405342      -1.109857      -0.673863
C         -2.941310      -1.481675       0.101026
H         -2.604121      -1.510535       1.134376
H         -2.526090      -2.328150      -0.442913
H         -4.027159      -1.544300       0.078068
S         -2.472142       0.066750      -0.700212
H          0.864061       2.329402       1.420122
H          1.337229      -0.158579       0.591819
S          2.540185      -1.115132       0.410269
C          2.633600      -0.701022      -1.361000
H          2.211410       0.290195      -1.520900
H          3.670826      -0.698339      -1.688295
H          2.077602      -1.418221      -1.961956
H         -0.236121      -0.962817       2.076795
H          0.105755       0.635886       2.741336
H         -1.507741       0.259005       2.133188
```

---

methylacrolein_protonation_TS_5

Datum	Value

Datum	Value
M06-2X/def2tzvpp-IEFPCM(water) Energy	-1108.135022
M06-2X/def2tzvpp-IEFPCM(water) Free Energy (Quasiharmonic)	-1107.997976
Number of Imaginary Frequencies	1

Frequencies (Top 3 out of 60)

```
1.    -946.9167 cm-1
2.      19.8091 cm-1
3.      44.4485 cm-1
```

M06-2X/def2tzvpp-IEFPCM(water) Molecular Geometry in Cartesian Coordinates

```
C          -0.549770        1.408555       -0.881528
C          -0.047499        0.652678        0.189303
C           0.935866       -0.432455       -0.109925
O          -1.251957        2.439229       -0.819135
C          -0.047941        1.236379        1.578819
H           0.846219       -1.254904        0.605990
H           0.775224       -0.836898       -1.111639
C           3.524246       -1.413745       -0.274716
H           3.263344       -1.841329       -1.241326
H           3.270048       -2.117695        0.515905
H           4.594132       -1.220648       -0.248318
S           2.669716        0.154626       -0.022523
H          -0.339611        0.977982       -1.882925
H          -1.314533       -0.183582        0.426415
S          -2.538232       -1.055338        0.723956
C          -2.859202       -1.264701       -1.051599
H          -2.805007       -2.312497       -1.337803
H          -3.839433       -0.876176       -1.318089
H          -2.102524       -0.710903       -1.612023
H          -0.906822        1.895845        1.703127
H          -0.117672        0.443680        2.330947
H           0.854340        1.814407        1.797763
```

---

methylacrolein_protonation_TS_6

Datum	Value
M06-2X/def2tzvpp-IEFPCM(water) Energy	-1108.130865
M06-2X/def2tzvpp-IEFPCM(water) Free Energy (Quasiharmonic)	-1107.991997

Datum	Value
Number of Imaginary Frequencies	1

Frequencies (Top 3 out of 60)

```
1.    -942.8719 cm-1
2.      38.3853 cm-1
3.      62.9647 cm-1
```

M06-2X/def2tzvpp-IEFPCM(water) Molecular Geometry in Cartesian Coordinates

```
C        -1.000302      -1.813533       0.025984
C         0.082808      -1.009233       0.395765
C         1.146458      -0.841372      -0.670801
O        -1.305411      -2.177780      -1.134652
C         0.449702      -0.897705       1.852061
H         1.558456      -1.810342      -0.971562
H         0.714673      -0.400524      -1.572976
C         1.892210       1.778756      -0.095519
H         1.193155       1.837479       0.736190
H         1.376687       2.042943      -1.017995
H         2.711084       2.475359       0.071414
S         2.608446       0.131116      -0.230282
H        -1.676217      -2.095225       0.858625
H        -0.734940       0.321313       0.126427
S        -1.524169       1.599374      -0.169067
C        -3.123299       0.797989       0.137974
H        -3.633649       0.562220      -0.793501
H        -2.955745      -0.130197       0.686059
H        -3.759458       1.444096       0.738247
H         0.732982       0.120736       2.133401
H        -0.405736      -1.178460       2.470153
H         1.288102      -1.544400       2.129533
```

---

methylacrolein_protonation_TS_7

Datum	Value
M06-2X/def2tzvpp-IEFPCM(water) Energy	-1108.130916
M06-2X/def2tzvpp-IEFPCM(water) Free Energy (Quasiharmonic)	-1107.991613
Number of Imaginary Frequencies	1

Frequencies (Top 3 out of 60)

```
1.    -948.0305 cm-1
2.      49.9439 cm-1
3.      67.8978 cm-1
```

M06-2X/def2tzvpp-IEFPCM(water) Molecular Geometry in Cartesian Coordinates

```
C         -0.018582        1.222970        1.169594
C         -0.173211        1.175408       -0.219399
C         -1.380755        0.582251       -0.905635
O          0.837390        1.881524        1.810611
C          0.531968        2.198670       -1.073506
H         -2.054250        1.372386       -1.255093
H         -1.060742        0.037657       -1.802348
C         -1.364371       -1.917536        0.322918
H         -0.609674       -1.664120        1.062942
H         -0.873779       -2.215062       -0.602504
H         -1.972551       -2.739759        0.693531
S         -2.471321       -0.527695        0.020690
H         -0.688821        0.553971        1.738662
H          0.897097        0.084564       -0.463439
S          2.035962       -0.861830       -0.874399
C          2.096380       -1.638886        0.766894
H          3.128119       -1.734574        1.097615
H          1.560637       -1.011701        1.480588
H          1.636966       -2.625504        0.754834
H          1.413541        2.576705       -0.557836
H          0.857227        1.760105       -2.023521
H         -0.115722        3.048272       -1.314182
```

---

methylacrolein_protonation_TS_8

Datum	Value
M06-2X/def2tzvpp-IEFPCM(water) Energy	-1108.130689
M06-2X/def2tzvpp-IEFPCM(water) Free Energy (Quasiharmonic)	-1107.992128
Number of Imaginary Frequencies	1

Frequencies (Top 3 out of 60)

```
1.   -1104.2330 cm-1
2.      19.4964 cm-1
3.      51.0753 cm-1
```

M06-2X/def2tzvpp-IEFPCM(water) Molecular Geometry in Cartesian Coordinates

```
C          0.407338        1.810054        0.715651
C         -0.273551        1.192797       -0.354575
C         -1.677688        0.721618       -0.093465
O          0.123905        1.770489        1.928422
C          0.003948        1.661808       -1.763102
H         -1.965769        0.944225        0.934078
H         -2.380701        1.229045       -0.758742
C         -1.237835       -1.704547        1.127162
H         -1.751137       -1.346271        2.017982
H         -0.192546       -1.401607        1.147016
H         -1.294021       -2.790464        1.089773
S         -2.028741       -1.054150       -0.354668
H          1.335254        2.338328        0.412378
H          0.692092        0.017517       -0.472138
S          1.786401       -1.076936       -0.811672
C          2.697585       -0.744228        0.725739
H          3.737038       -1.043328        0.608970
H          2.671092        0.321250        0.952565
H          2.271789       -1.289326        1.567361
H         -0.706127        2.427176       -2.091977
H          1.011553        2.073300       -1.846490
H         -0.061094        0.828595       -2.471182
```

---

methylacrylate_protonation_TS_10

Datum	Value
M06-2X/def2tzvpp-IEFPCM(water) Energy	-1183.375966
M06-2X/def2tzvpp-IEFPCM(water) Free Energy (Quasiharmonic)	-1183.233336
Number of Imaginary Frequencies	1

Frequencies (Top 3 out of 63)

```
1.    -965.9366 cm-1
2.      18.0541 cm-1
3.      46.1626 cm-1
```

M06-2X/def2tzvpp-IEFPCM(water) Molecular Geometry in Cartesian Coordinates

```
C        -0.048542     0.983074     0.509377
C         0.207931    -0.060711    -0.404834
C         1.095219    -1.177656     0.030203
O         0.171735     0.969952     1.725991
H         0.201697     0.158453    -1.463485
H         0.799112    -2.129512    -0.419438
H         1.049471    -1.292721     1.112542
C         3.261206     0.535787     0.365162
H         4.297338     0.769621     0.130980
H         3.139586     0.453214     1.443098
H         2.617484     1.328844    -0.011203
S         2.861169    -1.023306    -0.441868
O        -0.681474     2.126278     0.062699
C        -1.080102     2.222045    -1.290804
H        -1.158869    -0.896182    -0.295455
S        -2.417116    -1.709141    -0.106827
C        -3.380899    -0.239796     0.368806
H        -4.069246    -0.493586     1.171390
H        -2.703275     0.536114     0.722712
H        -3.951172     0.147774    -0.472661
H        -1.733153     1.392286    -1.569395
H        -1.625488     3.157620    -1.385356
H        -0.219308     2.240927    -1.961598
```

---

methylacrylate_protonation_TS_11_reopt

Datum	Value
M06-2X/def2tzvpp-IEFPCM(water) Energy	-1183.375213
M06-2X/def2tzvpp-IEFPCM(water) Free Energy (Quasiharmonic)	-1183.231854
Number of Imaginary Frequencies	1

Frequencies (Top 3 out of 63)

```
1.   -1139.4638 cm-1
2.      48.2610 cm-1
3.      55.9449 cm-1
```

M06-2X/def2tzvpp-IEFPCM(water) Molecular Geometry in Cartesian Coordinates

```
C          1.480766       -0.774308       -0.144426
C          0.372081       -0.699264       -1.016659
C         -0.696705       -1.736619       -0.893438
O          1.575425       -1.399536        0.914404
H          0.584336       -0.328559       -2.012171
H         -0.371524       -2.556962       -0.252316
H         -0.967082       -2.156697       -1.862300
C         -1.777290       -0.774710        1.462434
H         -1.398575       -1.663017        1.964809
H         -2.639578       -0.392752        2.005399
H         -0.998048       -0.014212        1.429674
S         -2.310849       -1.179767       -0.209801
O          2.506257        0.057387       -0.531851
C          3.581354        0.183191        0.383101
H          0.019045        0.773979       -0.477673
S         -0.075171        2.249784       -0.036130
C         -1.882183        2.437630       -0.053786
H         -2.350131        1.813969        0.705265
H         -2.123827        3.478772        0.150191
H         -2.291897        2.168185       -1.024835
H          4.070004       -0.775547        0.553359
H          4.284245        0.878292       -0.068398
H          3.237755        0.575950        1.340097
```

---

methylacrylate_protonation_TS_12_reopt

Datum	Value
M06-2X/def2tzvpp-IEFPCM(water) Energy	-1183.376546
M06-2X/def2tzvpp-IEFPCM(water) Free Energy (Quasiharmonic)	-1183.234796
Number of Imaginary Frequencies	1

Frequencies (Top 3 out of 63)

```
1.    -862.5931 cm-1
2.      32.5350 cm-1
3.      43.0538 cm-1
```

M06-2X/def2tzvpp-IEFPCM(water) Molecular Geometry in Cartesian Coordinates

```
C          0.536903        1.029010        0.701114
C         -0.144751       -0.171089        0.987178
C         -1.193936       -0.724097        0.086896
```

```
O          1.289909       1.658944       1.453958
H         -0.226765      -0.396997       2.043851
H         -1.352953      -1.787778       0.277333
H         -0.916096      -0.607907      -0.960825
C         -3.815054      -0.869092      -0.847906
H         -3.818814      -1.920842      -0.565763
H         -3.429161      -0.766313      -1.860834
H         -4.833953      -0.489828      -0.816316
S         -2.821567       0.094338       0.311117
O          0.375598       1.459862      -0.597214
C          1.186566       2.550256      -0.992964
H          1.070296      -1.211942       0.595125
S          2.184961      -2.074704       0.086910
C          2.923054      -0.699595      -0.848923
H          2.266025      -0.382274      -1.655812
H          3.106056       0.148248      -0.191538
H          3.869386      -1.029199      -1.271249
H          2.245387       2.299171      -0.917805
H          0.932404       2.757839      -2.029261
H          0.993129       3.430879      -0.381667
```

---

methylacrylate_protonation_TS_13

Datum	Value
M06-2X/def2tzvpp-IEFPCM(water) Energy	-1183.375235
M06-2X/def2tzvpp-IEFPCM(water) Free Energy (Quasiharmonic)	-1183.233378
Number of Imaginary Frequencies	1

Frequencies (Top 3 out of 63)

```
1.   -1047.0807 cm-1
2.      33.2710 cm-1
3.      42.1065 cm-1
```

M06-2X/def2tzvpp-IEFPCM(water) Molecular Geometry in Cartesian Coordinates

```
C          0.882790      -1.231648      -0.185473
C         -0.056263      -0.531671      -0.971850
C         -1.444488      -1.075025      -1.076604
O          0.665796      -2.064732       0.699482
H          0.346183      -0.032404      -1.845743
H         -1.511922      -2.051590      -0.595968
H         -1.762643      -1.189776      -2.113308
```

```
C          -2.331470       -0.218761        1.398098
H          -2.465730       -1.244200        1.738440
H          -2.971502        0.441608        1.979216
H          -1.291448        0.072991        1.533204
S          -2.791524       -0.060599       -0.336263
O           2.178109       -0.830665       -0.427326
C           3.160291       -1.342580        0.455549
H           0.032284        0.814420       -0.060190
S           0.354994        2.136679        0.618381
C           1.510672        2.625334       -0.697608
H           0.975704        2.981079       -1.576017
H           2.154265        3.423724       -0.335457
H           2.125163        1.771339       -0.975454
H           4.107424       -0.914893        0.136978
H           2.953691       -1.050632        1.485438
H           3.212592       -2.429670        0.405069
```

---

methylacrylate_protonation_TS_14

Datum	Value
M06-2X/def2tzvpp-IEFPCM(water) Energy	-1183.377904
M06-2X/def2tzvpp-IEFPCM(water) Free Energy (Quasiharmonic)	-1183.236272
Number of Imaginary Frequencies	1

Frequencies (Top 3 out of 63)

```
1.    -915.0001 cm-1
2.      44.4193 cm-1
3.      48.6996 cm-1
```

M06-2X/def2tzvpp-IEFPCM(water) Molecular Geometry in Cartesian Coordinates

```
C           0.512692       -0.994254        0.034327
C          -0.203481       -0.052385       -0.729296
C          -1.403423        0.593431       -0.132266
O           0.454540       -1.177456        1.255068
H          -0.165294       -0.164867       -1.805636
H          -1.630424        1.546480       -0.615741
H          -1.243742        0.781002        0.930454
C          -4.120214        0.645648        0.485265
H          -3.853540        0.846890        1.521500
H          -5.086977        0.148062        0.457691
H          -4.188806        1.584981       -0.061269
```

```
S          -2.905897       -0.447132       -0.282480
O           1.426960       -1.708150       -0.711241
C           2.316757       -2.526642        0.025062
H           0.810393        1.214290       -0.477921
S           1.759436        2.316085       -0.113981
C           3.062550        1.129646        0.339834
H           3.314104        0.493830       -0.506649
H           2.733404        0.501394        1.165062
H           3.950612        1.679059        0.643137
H           1.779573       -3.276057        0.605345
H           2.955698       -3.015818       -0.705677
H           2.927075       -1.930315        0.704902
```

---

methylacrylate_protonation_TS_15

Datum	Value
M06-2X/def2tzvpp-IEFPCM(water) Energy	-1183.37358
M06-2X/def2tzvpp-IEFPCM(water) Free Energy (Quasiharmonic)	-1183.230813
Number of Imaginary Frequencies	1

Frequencies (Top 3 out of 63)

```
1.   -1033.2368 cm-1
2.      38.2393 cm-1
3.      49.6542 cm-1
```

M06-2X/def2tzvpp-IEFPCM(water) Molecular Geometry in Cartesian Coordinates

```
C          -1.470792       -0.736636       -0.551259
C          -0.272684       -0.215115       -1.086370
C          -0.075147        1.252717       -1.301032
O          -1.824660       -1.921001       -0.543300
H           0.193466       -0.872873       -1.811146
H          -1.006637        1.807201       -1.193378
H           0.317547        1.457385       -2.297475
C           0.273402        1.903484        1.368936
H           0.074052        0.848433        1.546502
H          -0.662937        2.458695        1.365388
H           0.920680        2.286067        2.155425
S           1.135043        2.098946       -0.199387
O          -2.252434        0.197514        0.088408
C          -3.418282       -0.297751        0.721966
H           0.665802       -0.830327        0.079340
```

```
S          1.737537      -1.530603       0.901394
C          2.928521      -1.417798      -0.465528
H          3.939229      -1.523387      -0.078143
H          2.755500      -2.196094      -1.206293
H          2.834119      -0.442761      -0.942779
H         -4.094045      -0.758647       0.001950
H         -3.169893      -1.033470       1.486198
H         -3.901519       0.560784       1.181164
```

---

methylacrylate_protonation_TS_1_reopt

Datum	Value
M06-2X/def2tzvpp-IEFPCM(water) Energy	-1183.376885
M06-2X/def2tzvpp-IEFPCM(water) Free Energy (Quasiharmonic)	-1183.234779
Number of Imaginary Frequencies	1

Frequencies (Top 3 out of 63)

```
1.    -878.4689 cm-1
2.      29.0385 cm-1
3.      41.8408 cm-1
```

M06-2X/def2tzvpp-IEFPCM(water) Molecular Geometry in Cartesian Coordinates

```
C          0.126431       0.840243       0.678655
C         -0.194605      -0.530739       0.700669
C         -0.961880      -1.196652      -0.384775
O          0.619328       1.504719       1.599395
H         -0.283108      -0.948313       1.697665
H         -0.697474      -2.252926      -0.474408
H         -0.765166      -0.726617      -1.347141
C         -3.157159       0.499864      -0.049937
H         -2.589163       0.942496       0.766693
H         -4.220805       0.602492       0.153165
H         -2.911794       1.011425      -0.978243
S         -2.793978      -1.259583      -0.170656
O         -0.076021       1.446407      -0.544845
C          0.419396       2.766121      -0.669361
H          1.322887      -1.128462       0.437610
S          2.698962      -1.590943       0.086219
C          3.116624      -0.006507      -0.703805
H          3.527955       0.697201       0.016784
H          3.848004      -0.174062      -1.490703
```

```
H          2.218606        0.423782       -1.144412
H          1.500127        2.795488       -0.521616
H          0.179615        3.086596       -1.680084
H         -0.048719        3.436337        0.050592
```

---

methylacrylate_protonation_TS_2

Datum	Value
M06-2X/def2tzvpp-IEFPCM(water) Energy	-1183.378412
M06-2X/def2tzvpp-IEFPCM(water) Free Energy (Quasiharmonic)	-1183.23601
Number of Imaginary Frequencies	1

Frequencies (Top 3 out of 63)

```
1.    -778.6910 cm-1
2.      37.6889 cm-1
3.      50.6403 cm-1
```

M06-2X/def2tzvpp-IEFPCM(water) Molecular Geometry in Cartesian Coordinates

```
C         -0.159654        0.894876        0.041148
C          0.289642       -0.262466       -0.620446
C          1.204716       -1.196264        0.087991
O         -0.175835        1.116782        1.257638
H          0.352616       -0.212523       -1.700720
H          1.045089       -2.237186       -0.206385
H          1.043426       -1.129998        1.164131
C          3.181410        0.746956        0.349582
H          2.508545        1.396910       -0.207275
H          4.209764        1.050115        0.166373
H          2.964057        0.825397        1.413000
S          3.003752       -0.953360       -0.215626
O         -0.703745        1.831784       -0.818210
C         -1.368813        2.918204       -0.201166
H         -1.052091       -1.192403       -0.340076
S         -2.263188       -1.979340        0.012354
C         -3.279001       -0.479226        0.177541
H         -2.843123        0.314894       -0.427427
H         -4.287634       -0.676164       -0.177391
H         -3.325763       -0.147326        1.212399
H         -0.691621        3.493599        0.429055
H         -1.741922        3.544685       -1.007374
H         -2.203526        2.572183        0.410711
```

methylacrylate_protonation_TS_3_reopt

Datum	Value
M06-2X/def2tzvpp-IEFPCM(water) Energy	-1183.376267
M06-2X/def2tzvpp-IEFPCM(water) Free Energy (Quasiharmonic)	-1183.234537
Number of Imaginary Frequencies	1

Frequencies (Top 3 out of 63)

```
1.    -857.6460 cm-1
2.      28.9630 cm-1
3.      38.3391 cm-1
```

M06-2X/def2tzvpp-IEFPCM(water) Molecular Geometry in Cartesian Coordinates

```
C          0.296431        0.907042        0.695218
C         -0.312173       -0.359648        0.601763
C         -1.102310       -0.774430       -0.586333
O          0.860463        1.396111        1.681555
H         -0.561983       -0.806311        1.556694
H         -1.204127       -1.860712       -0.626246
H         -0.623538       -0.455257       -1.511614
C         -3.526887       -0.819581        0.761137
H         -3.475599       -1.907215        0.729157
H         -4.568954       -0.513586        0.817012
H         -3.000653       -0.455259        1.641404
S         -2.819787       -0.107474       -0.734676
O          0.298243        1.609783       -0.491597
C          1.083731        2.787383       -0.509311
H          1.063580       -1.261216        0.385531
S          2.335744       -1.970772        0.062113
C          3.100247       -0.464038       -0.611403
H          2.336398        0.145012       -1.092055
H          3.575625        0.121075        0.173066
H          3.848959       -0.737291       -1.351022
H          2.134261        2.560140       -0.321295
H          0.973342        3.212210       -1.503727
H          0.743487        3.502817        0.238018
```

methylacrylate_protonation_TS_6_reopt

Datum	Value
M06-2X/def2tzvpp-IEFPCM(water) Energy	-1183.374595
M06-2X/def2tzvpp-IEFPCM(water) Free Energy (Quasiharmonic)	-1183.232044
Number of Imaginary Frequencies	1

Frequencies (Top 3 out of 63)

```
1.    -999.3999 cm-1
2.      29.1736 cm-1
3.      52.6002 cm-1
```

M06-2X/def2tzvpp-IEFPCM(water) Molecular Geometry in Cartesian Coordinates

```
C          1.410618       -0.906462        0.086721
C          0.121516       -0.990884       -0.480275
C         -0.967389       -1.620133        0.325770
O          1.729731       -0.976450        1.277750
H          0.097779       -1.147343       -1.552460
H         -0.616368       -1.817963        1.338490
H         -1.305514       -2.566776       -0.101953
C         -2.941102       -0.380588       -1.158223
H         -3.870711        0.183641       -1.187014
H         -3.089164       -1.338301       -1.656041
H         -2.162285        0.186969       -1.664859
S         -2.494310       -0.626808        0.568363
O          2.380586       -0.629859       -0.851800
C          3.668898       -0.334543       -0.342439
H         -0.024887        0.637001       -0.575199
S         -0.095093        2.152406       -0.577814
C         -0.029285        2.266318        1.235751
H         -0.507317        3.188204        1.559775
H         -0.568360        1.421818        1.662177
H          0.997138        2.256712        1.596092
H          4.080431       -1.178352        0.210528
H          4.294969       -0.121685       -1.205068
H          3.642661        0.534729        0.315318
```

---

methylacrylate_protonation_TS_7

Datum	Value

Datum	Value
M06-2X/def2tzvpp-IEFPCM(water) Energy	-1183.376105
M06-2X/def2tzvpp-IEFPCM(water) Free Energy (Quasiharmonic)	-1183.232545
Number of Imaginary Frequencies	1

Frequencies (Top 3 out of 63)

```
1.   -1058.2632 cm-1
2.      50.5981 cm-1
3.      59.1614 cm-1
```

M06-2X/def2tzvpp-IEFPCM(water) Molecular Geometry in Cartesian Coordinates

```
C          0.933606      -1.145730      -0.123768
C         -0.129623      -0.797812      -0.986886
C         -1.470853      -1.406507      -0.731095
O          0.868542      -1.653453       1.000054
H          0.148120      -0.649287      -2.023641
H         -1.403065      -2.190813       0.024025
H         -1.896310      -1.845986      -1.633382
C         -2.101276       0.144984       1.459928
H         -2.714303       0.927439       1.902350
H         -1.085483       0.513961       1.328167
H         -2.096565      -0.726499       2.112480
S         -2.796929      -0.269738      -0.147653
O          2.164580      -0.796331      -0.634550
C          3.265977      -0.971485       0.237943
H         -0.081007       0.807600      -0.760223
S          0.147834       2.301565      -0.570453
C          1.423742       2.055386       0.701756
H          2.407977       1.928706       0.255997
H          1.443312       2.914324       1.368808
H          1.192149       1.165379       1.287528
H          3.382331      -2.016146       0.524442
H          4.144085      -0.639897      -0.310268
H          3.149862      -0.372762       1.142122
```

---

methylacrylate_protonation_TS_9_reopt

Datum	Value
M06-2X/def2tzvpp-IEFPCM(water) Energy	-1183.377721

Datum	Value
M06-2X/def2tzvpp-IEFPCM(water) Free Energy (Quasiharmonic)	-1183.236608
Number of Imaginary Frequencies	1

Frequencies (Top 3 out of 63)

```
1.    -770.8678 cm-1
2.      25.0740 cm-1
3.      31.7548 cm-1
```

M06-2X/def2tzvpp-IEFPCM(water) Molecular Geometry in Cartesian Coordinates

```
C          0.299820       1.205280       0.078228
C          0.047022      -0.078924      -0.433247
C          0.510225      -1.273751       0.318711
O          0.620336       1.515520       1.232234
H         -0.070768      -0.165310      -1.506770
H         -0.167073      -2.123572       0.203030
H          0.572552      -1.045061       1.382727
C          3.187333      -0.534115       0.102527
H          2.816961       0.317929      -0.464991
H          4.190767      -0.784034      -0.234648
H          3.212336      -0.281391       1.160744
S          2.139071      -1.968942      -0.188753
O          0.051841       2.206211      -0.840086
C          0.153482       3.531582      -0.351608
H         -1.523026      -0.089988       0.118827
S         -2.920815      -0.386733       0.523374
C         -3.060045      -1.757956      -0.662656
H         -2.296882      -1.641332      -1.431911
H         -2.921217      -2.718612      -0.171560
H         -4.037835      -1.742190      -1.137549
H         -0.079775       4.182919      -1.189975
H         -0.552008       3.710324       0.459669
H          1.159430       3.744570       0.009566
```

---

methyltiglate_protonation_TS_1_reopt

Datum	Value
M06-2X/def2tzvpp-IEFPCM(water) Energy	-1261.99563
M06-2X/def2tzvpp-IEFPCM(water) Free Energy (Quasiharmonic)	-1261.800001

Datum	Value
Number of Imaginary Frequencies	1

Frequencies (Top 3 out of 81)

```
1.    -693.8495 cm-1
2.      33.4657 cm-1
3.      43.7981 cm-1
```

M06-2X/def2tzvpp-IEFPCM(water) Molecular Geometry in Cartesian Coordinates

```
C          0.310638        0.988741       -0.251810
C         -0.248789       -0.156170        0.351040
C         -1.272408       -0.890723       -0.452600
O          0.311498        1.293361       -1.454590
C         -0.315179       -0.263281        1.852440
H         -1.118698       -0.652532       -1.503930
C         -1.277585       -2.406543       -0.277950
C         -2.833452        1.435144       -0.108510
H         -2.146123        1.758956        0.671700
H         -3.814323        1.870402        0.070740
H         -2.458563        1.765745       -1.074910
S         -3.022479       -0.355366       -0.076550
O          0.985307        1.805512        0.635230
C          1.708265        2.877394        0.061660
H          1.112303       -1.102888        0.077250
S          2.367884       -1.872855       -0.090760
C          3.395661       -0.376353       -0.201280
H          3.369240        0.182697        0.731440
H          4.421412       -0.671041       -0.409780
H          3.041630        0.264256       -1.006380
H          2.173834        3.407185        0.889200
H          2.478325        2.515465       -0.620570
H          1.051003        3.553072       -0.484790
H         -1.132150        0.322568        2.293630
H         -0.468737       -1.300241        2.160670
H          0.613640        0.073401        2.311760
H         -2.063114       -2.867604       -0.878440
H         -0.320054       -2.820691       -0.596030
H         -1.443464       -2.687453        0.762330
```

---

methyltiglate_protonation_TS_2_reopt

Datum	Value
M06-2X/def2tzvpp-IEFPCM(water) Energy	-1261.995398
M06-2X/def2tzvpp-IEFPCM(water) Free Energy (Quasiharmonic)	-1261.800611
Number of Imaginary Frequencies	1

Frequencies (Top 3 out of 81)

```
1.    -819.7602 cm-1
2.      17.6361 cm-1
3.      38.8570 cm-1
```

M06-2X/def2tzvpp-IEFPCM(water) Molecular Geometry in Cartesian Coordinates

```
C          0.283495        0.989773        0.514684
C         -0.196050       -0.338950        0.501949
C         -1.026111       -0.825645       -0.639927
O          0.822556        1.582585        1.459174
C         -0.391926       -1.013170        1.834289
H         -0.751768       -0.292401       -1.546307
C         -0.918758       -2.326814       -0.890915
C         -2.877752        1.130785        0.300456
H         -3.919753        1.401552        0.458582
H         -2.410508        1.854219       -0.363554
H         -2.357342        1.129812        1.257047
S         -2.863579       -0.518484       -0.423252
O          0.194963        1.635067       -0.703644
C          0.813699        2.904888       -0.775092
H          1.300303       -0.967027        0.218704
S          2.695598       -1.476280        0.045834
C          3.254417        0.105409       -0.659605
H          3.245476        0.886353        0.097843
H          4.266558       -0.013453       -1.038918
H          2.603590        0.405656       -1.478580
H          0.635137        3.273279       -1.782326
H          0.387424        3.596229       -0.048757
H          1.887274        2.830438       -0.596859
H          0.020932       -2.028625        1.847861
H          0.108907       -0.447091        2.617446
H         -1.452974       -1.097578        2.098924
H         -1.557314       -2.634628       -1.720336
H          0.112321       -2.586725       -1.133650
H         -1.216810       -2.892657       -0.007701
```

---

methyltiglate_protonation_TS_3_reopt

Datum	Value
M06-2X/def2tzvpp-IEFPCM(water) Energy	-1261.991964
M06-2X/def2tzvpp-IEFPCM(water) Free Energy (Quasiharmonic)	-1261.795405
Number of Imaginary Frequencies	1

Frequencies (Top 3 out of 81)

```
1.    -407.1640 cm-1
2.      40.1474 cm-1
3.      46.2775 cm-1
```

M06-2X/def2tzvpp-IEFPCM(water) Molecular Geometry in Cartesian Coordinates

```
C        -0.223820       0.946490       0.685121
C         0.182719      -0.397790       0.618361
C         0.952799      -1.021370      -0.501849
O        -0.753180       1.506890       1.660341
C         0.286739      -1.120620       1.936861
H         0.776689      -2.100600      -0.437169
C         0.631189      -0.599070      -1.932429
C         3.033690       0.768269       0.128651
H         2.687900       1.391789      -0.693139
H         4.097640       0.931978       0.285911
H         2.487670       1.032349       1.033351
S         2.805199      -0.978641      -0.243519
O        -0.064930       1.677820      -0.476329
C        -0.671310       2.955100      -0.488799
H        -1.355571      -1.036320       0.284561
S        -2.687941      -1.553549      -0.008499
C        -3.217541       0.029351      -0.731209
H        -3.684840       0.665711       0.017051
H        -2.347151       0.543141      -1.137699
H        -3.927121      -0.155299      -1.533819
H        -0.262669       3.601130       0.287501
H        -0.461770       3.380270      -1.467619
H        -1.750720       2.881380      -0.345939
H        -0.482021      -0.776220       2.627371
H         0.152759      -2.198820       1.795621
H         1.259479      -0.983831       2.425481
H         1.060669      -1.316671      -2.631409
H        -0.451241      -0.573090      -2.076549
H         1.022269       0.388319      -2.161599
```

methyltiglate_protonation_TS_4_reopt

Datum	Value
M06-2X/def2tzvpp-IEFPCM(water) Energy	-1261.992008
M06-2X/def2tzvpp-IEFPCM(water) Free Energy (Quasiharmonic)	-1261.796031
Number of Imaginary Frequencies	1

Frequencies (Top 3 out of 81)

```
1.    -478.5907 cm-1
2.      20.9729 cm-1
3.      49.6857 cm-1
```

M06-2X/def2tzvpp-IEFPCM(water) Molecular Geometry in Cartesian Coordinates

```
C         -0.229769       1.025769      -0.182711
C          0.212720      -0.113752       0.514549
C          1.124090      -1.108342      -0.130911
O         -0.152608       1.291409      -1.390981
C          0.256490      -0.101992       2.022099
H          0.994729      -2.059992       0.397029
C          0.927059      -1.370282      -1.619271
C          2.995891       0.983476      -0.177901
H          4.035032       1.298165      -0.110041
H          2.397002       1.548927       0.534779
H          2.621541       1.169306      -1.182551
S          2.932440      -0.774434       0.209959
O         -0.891228       1.931529       0.639609
C         -1.491467       3.030010      -0.016581
H         -1.194220      -1.043151       0.424779
S         -2.440961      -1.812779       0.396799
C         -3.231700      -0.602019      -0.705471
H         -4.306610      -0.764968      -0.697381
H         -3.024439       0.406541      -0.352541
H         -2.865570      -0.702879      -1.724751
H         -0.753837       3.628319      -0.550831
H         -2.250497       2.702980      -0.729171
H         -1.957597       3.631120       0.760579
H          1.146441       0.398308       2.424509
H         -0.618409       0.386799       2.447299
H          0.276640      -1.129602       2.400929
H          1.479169      -2.262343      -1.915511
H         -0.133921      -1.524731      -1.828661
H          1.261690      -0.528093      -2.218411
```

---

methyltiglate_protonation_TS_5

Datum	Value
M06-2X/def2tzvpp-IEFPCM(water) Energy	-1261.99038
M06-2X/def2tzvpp-IEFPCM(water) Free Energy (Quasiharmonic)	-1261.795169
Number of Imaginary Frequencies	1

Frequencies (Top 3 out of 81)

```
1.    -725.4892 cm-1
2.      39.1792 cm-1
3.      43.0756 cm-1
```

M06-2X/def2tzvpp-IEFPCM(water) Molecular Geometry in Cartesian Coordinates

```
C          1.515532      -0.694738      -0.319497
C          0.353065      -0.643826       0.460380
C         -0.927940      -0.907891      -0.296733
O          1.590941      -0.679845      -1.560865
C          0.370546      -0.932486       1.939050
H         -0.893887      -0.358996      -1.239822
C         -1.148637      -2.390666      -0.594368
C         -3.598498      -0.218283      -0.674322
H         -4.488591       0.261765      -0.273590
H         -3.854183      -1.230717      -0.978437
H         -3.250232       0.346122      -1.538825
S         -2.349127      -0.205606       0.625721
O          2.690798      -0.660213       0.411082
C          3.880300      -0.528554      -0.343622
H          0.481950       1.033732       0.493567
S          0.515241       2.521351       0.490242
C         -0.561105       2.685855      -0.966265
H         -0.788341       3.738952      -1.115609
H         -0.071232       2.303565      -1.859127
H         -1.490006       2.141709      -0.803466
H          4.016836      -1.369174      -1.023667
H          3.880395       0.392465      -0.927050
H          4.694969      -0.504777       0.376206
H          0.022677      -1.944085       2.181724
H          1.374829      -0.825714       2.342727
H         -0.276052      -0.239748       2.489858
H         -2.084731      -2.578084      -1.122389
```

```
H          -0.328730       -2.748882       -1.219356
H          -1.156985       -2.966050        0.332357
```

---

methyltiglate_protonation_TS_6_reopt

Datum	Value
M06-2X/def2tzvpp-IEFPCM(water) Energy	-1261.995126
M06-2X/def2tzvpp-IEFPCM(water) Free Energy (Quasiharmonic)	-1261.799784
Number of Imaginary Frequencies	1

Frequencies (Top 3 out of 81)

```
1.    -741.6843 cm-1
2.      30.0098 cm-1
3.      40.2793 cm-1
```

M06-2X/def2tzvpp-IEFPCM(water) Molecular Geometry in Cartesian Coordinates

```
C          -0.144500        1.277300       -0.279488
C          -0.130199       -0.036990        0.227772
C          -0.871418       -1.062590       -0.566018
O          -0.393300        1.647310       -1.437048
C           0.034711       -0.260080        1.710402
H          -0.976199       -0.697250       -1.586438
C          -0.239548       -2.450740       -0.596248
C          -3.176209        0.375269        0.171762
H          -2.588780        0.913619        0.914022
H          -4.219079        0.355208        0.480932
H          -3.090289        0.881639       -0.787568
S          -2.615378       -1.330971        0.040972
O           0.273200        2.219120        0.639342
C           0.449919        3.532970        0.141692
H           1.464871       -0.176969       -0.290028
S           2.907561       -0.376768       -0.597078
C           3.161862       -1.622628        0.702412
H           2.366042       -2.364649        0.673702
H           4.111402       -2.123178        0.528042
H           3.181892       -1.163028        1.687882
H           0.782439        4.134271        0.984152
H           1.201479        3.559831       -0.647378
H          -0.482281        3.936380       -0.252678
H          -0.833879        0.064990        2.297472
H           0.183982       -1.319430        1.926552
```

```
H          0.903541       0.273981        2.097842
H         -0.847997      -3.148250       -1.174078
H          0.747912      -2.390929       -1.055668
H         -0.126888      -2.858910        0.408922
```

---

methyltiglate_protonation_TS_7_reopt

Datum	Value
M06-2X/def2tzvpp-IEFPCM(water) Energy	-1261.994203
M06-2X/def2tzvpp-IEFPCM(water) Free Energy (Quasiharmonic)	-1261.798487
Number of Imaginary Frequencies	1

Frequencies (Top 3 out of 81)

```
1.    -768.7556 cm-1
2.      34.4900 cm-1
3.      44.7793 cm-1
```

M06-2X/def2tzvpp-IEFPCM(water) Molecular Geometry in Cartesian Coordinates

```
C         -0.631339      -1.056821       -0.111015
C          0.092307      -0.065563        0.578511
C          1.307117       0.472371       -0.116941
O         -0.529914      -1.360068       -1.309533
C          0.021994       0.079082        2.077397
H          1.110982       0.522022       -1.189236
C          1.739003       1.842945        0.388037
C          3.995878       0.017431       -0.927060
H          4.432810       0.878545       -0.426621
H          3.601450       0.320345       -1.896575
H          4.770966      -0.729999       -1.082591
S          2.689984      -0.747910        0.060557
O         -1.604822      -1.677966        0.647937
C         -2.466766      -2.553248       -0.052497
H         -0.954078       1.131002        0.121458
S         -1.925418       2.189206       -0.276259
C         -3.232031       0.980285       -0.653335
H         -4.103884       1.511684       -1.027476
H         -2.889759       0.279685       -1.412124
H         -3.512275       0.423751        0.238393
H         -3.158346      -2.953074        0.685120
H         -3.023520      -2.024225       -0.827014
H         -1.913756      -3.368355       -0.518052
```

```
H          0.914816       -0.309802        2.581401
H         -0.073173        1.129314        2.374819
H         -0.840113       -0.448684        2.477954
H          0.909403        2.548968        0.305035
H          2.044484        1.795898        1.433553
H          2.571857        2.237564       -0.192622
```

---

methyltiglate_protonation_TS_8_reopt

Datum	Value
M06-2X/def2tzvpp-IEFPCM(water) Energy	-1261.991981
M06-2X/def2tzvpp-IEFPCM(water) Free Energy (Quasiharmonic)	-1261.796391
Number of Imaginary Frequencies	1

Frequencies (Top 3 out of 81)

```
  1.    -854.1472 cm-1
  2.      12.8854 cm-1
  3.      37.4884 cm-1
```

M06-2X/def2tzvpp-IEFPCM(water) Molecular Geometry in Cartesian Coordinates

```
C          0.396893        0.995141        0.644963
C         -0.219863       -0.269602        0.565895
C         -0.996953       -0.661136       -0.657079
O          0.944212        1.512026        1.630295
C         -0.440747       -1.025360        1.852881
H         -0.432276       -0.389626       -1.551000
C         -1.296707       -2.156234       -0.701360
C         -3.352642        0.223366        0.669766
H         -4.292111        0.766841        0.594056
H         -2.719602        0.705582        1.412262
H         -3.562642       -0.800872        0.973555
S         -2.572596        0.290689       -0.952702
O          0.444758        1.659351       -0.562964
C          1.188374        2.862111       -0.577472
H          1.178645       -1.003551        0.177157
S          2.471835       -1.692770       -0.152662
C          3.284510       -0.134318       -0.626549
H          2.821258        0.291450       -1.514021
H          3.217692        0.587167        0.185233
H          4.332646       -0.334631       -0.836431
H          1.111551        3.254479       -1.588558
```

```
H          0.786502       3.586482        0.130207
H          2.236099       2.682231       -0.333119
H         -0.053266      -2.050160        1.793366
H          0.092137      -0.525523        2.660236
H         -1.490716      -1.106021        2.146624
H         -1.747974      -2.432809       -1.652733
H         -0.373794      -2.726676       -0.573317
H         -1.980914      -2.445890        0.097403
```

---

methyltiglate_protonation_TS_9_reopt

Datum	Value
M06-2X/def2tzvpp-IEFPCM(water) Energy	-1261.989248
M06-2X/def2tzvpp-IEFPCM(water) Free Energy (Quasiharmonic)	-1261.792692
Number of Imaginary Frequencies	1

Frequencies (Top 3 out of 81)

```
1.   -1009.6959 cm-1
2.      38.6576 cm-1
3.      45.4220 cm-1
```

M06-2X/def2tzvpp-IEFPCM(water) Molecular Geometry in Cartesian Coordinates

```
C          0.938151      -1.236577        0.277975
C         -0.138681      -0.468832        0.775850
C         -1.531645      -0.710002        0.255500
O          2.040404      -1.409486        0.812877
C         -0.024012      -0.008120        2.208526
H         -1.491118      -1.380486       -0.599819
C         -2.479583      -1.303730        1.297463
C         -1.598357       0.830108       -2.047313
H         -0.520590       0.730111       -1.938821
H         -1.822384       1.785202       -2.517625
H         -1.987018       0.022137       -2.665034
S         -2.369219       0.804517       -0.417072
O          0.725455      -1.766974       -0.979912
C          1.828674      -2.435526       -1.561601
H          0.418315       0.931133        0.186205
S          0.972537       2.320574       -0.103712
C          2.708976       1.927870        0.275044
H          2.828260       1.664565        1.323716
H          3.057566       1.096740       -0.333048
```

```
H           3.319889        2.802691        0.060915
H           2.141490       -3.287112       -0.957884
H           1.494184       -2.780895       -2.536678
H           2.679390       -1.764271       -1.681738
H           0.986212        0.345245        2.414548
H          -0.704184        0.829321        2.390373
H          -0.260288       -0.786151        2.943497
H          -3.436506       -1.568930        0.848844
H          -2.034862       -2.200040        1.733104
H          -2.669453       -0.600192        2.109603
```
